# Improved Wound Healing of Airway Epithelial Cells Is Mediated by Cold Atmospheric Plasma: A Time Course-Related Proteome Analysis

**DOI:** 10.1155/2019/7071536

**Published:** 2019-05-19

**Authors:** Christian Scharf, Christine Eymann, Philipp Emicke, Jörg Bernhardt, Martin Wilhelm, Fabian Görries, Jörn Winter, Thomas von Woedtke, Katrin Darm, Georg Daeschlein, Leif Steil, Werner Hosemann, Achim Beule

**Affiliations:** ^1^Department of Otorhinolaryngology, Head and Neck Surgery, University Medicine Greifswald, Germany; ^2^Institute for Microbiology, University of Greifswald, Germany; ^3^Leibniz Institute for Plasma Science and Technology (INP), Greifswald, Germany; ^4^Department of Hygiene and Environmental Medicine, University Medicine Greifswald, Greifswald, Germany; ^5^Department of Dermatology, University Medicine Greifswald, Greifswald, Germany; ^6^Interfaculty Institute of Genetics and Functional Genomics, University of Greifswald, Germany; ^7^Department of Otorhinolaryngology, University Hospital Münster, Münster, Germany

## Abstract

The promising potential of cold atmospheric plasma (CAP) treatment as a new therapeutic option in the field of medicine, particularly in Otorhinolaryngology and Respiratory medicine, demands primarily the assessment of potential risks and the prevention of any direct and future cell damages. Consequently, the application of a special intensity of CAP that is well tolerated by cells and tissues is of particular interest. Although improvement of wound healing by CAP treatment has been described, the underlying mechanisms and the molecular influences on human tissues are so far only partially characterized. In this study, human S9 bronchial epithelial cells were treated with cold plasma of atmospheric pressure plasma jet that was previously proven to accelerate the wound healing in a clinically relevant extent. We studied the detailed cellular adaptation reactions for a specified plasma intensity by time-resolved comparative proteome analyses of plasma treated vs. nontreated cells to elucidate the mechanisms of the observed improved wound healing and to define potential biomarkers and networks for the evaluation of plasma effects on human epithelial cells. K-means cluster analysis and time-related analysis of fold-change factors indicated concordantly clear differences between the short-term (up to 1 h) and long-term (24-72 h) adaptation reactions. Thus, the induction of Nrf2-mediated oxidative and endoplasmic reticulum stress response, PPAR-alpha/RXR activation as well as production of peroxisomes, and prevention of apoptosis already during the first hour after CAP treatment are important cell strategies to overcome oxidative stress and to protect and maintain cell integrity and especially microtubule dynamics. After resolving of stress, when stress adaptation was accomplished, the cells seem to start again with proliferation and cellular assembly and organization. The observed strategies and identification of marker proteins might explain the accelerated wound healing induced by CAP, and these indicators might be subsequently used for risk assessment and quality management of application of nonthermal plasma sources in clinical settings.

## 1. Background

The prospective application of cold atmospheric plasma (CAP) on the human body for the therapy of several diseases (e.g., cancer) and nonhealing, chronic infected wounds of several locations requires an exact evaluation of possible risks and long-term effects and therefore a deep but comprehensive analysis and description of molecular effects on human cells and tissues in response to cold plasma treatment.

The term plasma in physics is defined as the fourth state of matter and is generated almost completely or partly by ionization of gas. According to the thermal equilibrium state of electrons and heavy particles (e.g., atoms and ions), two temperature plasma forms can be classified: (I) thermal plasmas can reach several thousand degrees Kelvin and were used for instance for the ablation and cauterisation of biological tissue [1–4]. (II) Nonequilibrium plasmas can be created artificially under low and atmospheric pressure conditions and are characterized by a strong imbalance of electron and heavy particle temperature. Whereas electrons can reach temperatures of several 10,000 K, the heavy particles can be kept close to body or room temperature. In contrast to the damaging effects of thermal plasma, nonequilibrium or cold plasma is assumed to trigger more nondamaging or beneficial effects [5–9]. Several medical applications of CAP can be envisioned. One of the most innovative fields is the direct application onto the patient's skin for some therapeutic purposes ranging from skin disinfection via treatment of infective skin diseases [10–13] and stimulation of wound healing [14–18]. Further promising are applications in cancer treatment [19–23].

Plasma is composed of charged particles (electrons, ions), excited atoms and molecules, radicals, and UV photons. Therefore, CAP was shown to be able to affect various complex responses in biological systems by interaction between plasma and different cell lines or tissues. In the strict sense, exposure of cells to plasma means an exposition of cells to charged particles, reactive oxygen and nitrogen species (ROS, RNS), and to a minor degree several types of radiation [24]. In this context, both direct and indirect effects of CAP on living matter were subject of analysis [25, 26]. Here, direct and indirect effects resulted from direct treatment of cell cultures or treatment of culture medium before cultivation, respectively. Examples for these effects are (I) inactivation of a broad spectrum of microorganisms, (II) stimulation of cell proliferation and tissue regeneration, (III) inactivation of cells by initialization of programmed cell death (apoptosis), and (IV) promotion of angiogenesis [9, 27–32]. These effects could be probably referred to at least two major principles: (1) changes of the liquid environment of cells and (2) the dominating role of ROS and RNS generated in and/or transferred into liquid phases (for review, see [9]). Both ROS and RNS play a central role in redox biology as normal components of the cellular metabolism. Under normal conditions, an excess of ROS is neutralized by an antioxidant system and it was shown that ROS triggers the programmed cell death (apoptosis) of highly stressed cells [33]. The supposed sublethal and therefore selective properties of CAP could be used for some therapeutic methods in a variety of medical disciplines treating diseases of the surface of a patient including internal medicine, surgery, otorhinolaryngology, and dermatology. However, a future application of plasma requires comprehensive risk estimation because oxidative stress with severe consequences, e.g., DNA damages or apoptosis, must be avoided in healthy cells. Therefore, the application of an appropriate intensity or plasma treatment time is very important because in the most cases damaging effects should be avoided. A plasma intensity that is well tolerated by cells and tissues should be favoured.

Many *in vitro* attempts were made to modulate cell repair and regeneration, using CAP as an effector of wound healing acceleration [17, 18, 34, 35]. But the underlying molecular mechanisms mediating the effects of nonthermal plasma have not been completely revealed yet. Most of previous studies have examined skin treatment, neglecting the potential of inner surfaces including airway and gastrointestinal surface. As the mucosa of the mouth and upper airway are easily accessible, we evaluated the specific requirements for otorhinolaryngologic applications: the mucosa of the upper airways and the oral cavity is different from skin surfaces elsewhere on the human body, less resistant to mechanic trauma, and therefore might be much more sensitive to external plasma applications. Moreover, we have to take into account a partial indirect plasma effect elucidated by produced liquids such as saliva or mucus as well as the difficult accessibility of different cavities. Furthermore, especially the mucosa of the oral cavity displays a different microbiome [36]. Indeed, plasma treatment has recently induced proliferative effects in mammalian epithelial and endothelial cells [30–32]. However, the molecular mechanisms involved have not yet been explored in all details. Here, proteomic analyses as state of the art can provide new insights by providing comprehensive protein level information of global adaptation reactions. In a previous proteome study, we analysed “dose”-dependent plasma effects on upper airway epithelial cells and identified one “tissue-tolerable” plasma (TTP) intensity (120 s) that accelerated the wound healing of S9 cells in a clinically relevant extent based on extensive proteome changes [17]. Now, by performing experiments in a more time-resolved fashion, using this specified CAP intensity (120 s), direct and secondary effects, including potential positive and harmful molecular modifications induced by plasma treatment, might be identified. As a surrogate model for human mucosa of the throat or upper airway medical treatment in general, CAP effects on S9-human bronchial epithelial cells were investigated by time-resolved comparative proteome analyses of CAP treated vs. nontreated cells in a standardised wound model [17]. Thus, the current proteome study now provides an overview of how CAP affects the protein pattern and consequently the wound healing of human S9 cells observed after a short term and long term by unravelling the different cellular adaptation reactions in a time-scheduled manner.

## 2. Methods

### 2.1. Cell Line and Cell Cultivation

S9 epithelial cells (ATCC CRL-2778) [37, 38] were incubated with a 10 ml standard cell cultivation medium (93% MEM Earl Standard w/o L-glutamine, 4% FCS, 2% glutamine, and 1% nonessential amino acids) in a cell culture plate at atmospheric conditions of 95% air and 5% CO_2_ at 37°C. Subcultivation was performed at an approximate cell density of 1 × 10^7^ cells/cm^2^ by removing the cell cultivation medium, washing with 5.0 ml PBS (8.0 g NaCl, 0.2 g KCl, 1.44 g Na_2_HPO_4_^∗^2H_2_O, 0.24 g KH_2_PO_4_, and Aqua Dest ad 1000 ml), and overlaying with 1.0 ml trypsin solution (0.05% trypsin, 0.02% EDTA) for 5 to 15 minutes to detach cells from the surface [39, 40]. Then, a 3 ml standard cell cultivation medium was added, and cells were repeatedly pipetted and afterwards divided into aliquots in new cell culture plates each with a 9 ml fresh cell cultivation medium with a subcultivation ratio of 1 : 4, which corresponded to approximately 1 × 10^7^ viable cells per cell culture plate. These subcultures were incubated as described above until 95% confluency of cells was reached. Substitution of the medium against fresh medium was performed after 60 h.

### 2.2. Cold Atmospheric Plasma (CAP) Treatment of S9 Epithelial Cells

For plasma treatment, atmospheric plasma jets kINPen 08 and 09 (INP Greifswald, neoplas Greifswald) were used [17, 27]. To generate argon plasma, a high-frequency voltage signal of 2-6 kVpp and 1.1 MHz was applied to a pin-pointed metal electrode within a ceramic capillary. At the tip of the generated plasma jet, the measured temperatures are in the range from 37°C to 42°C. A treatment time of 120 s for an area of about 58 cm^2^ (corresponds to 2.07 sec/cm^2^) previously determined as the most suitable intensity for improving the wound healing [17] was therefore applied to the cell cultures. The cell culture plates of two independent experiments covered with 1 ml standard cell medium were plasma treated in a meander-like pattern. In this setting, the visible tip of the plasma jet touches the surface layer of the cell culture medium. After plasma treatment, cell cultures were incubated for several time periods enabling assessment of proteome analysis (incubation of 0, 0.5, 1, 24, 48, and 72 h before cell harvesting). Nontreated control cells were incubated accordingly.

### 2.3. Sample Preparation for Proteome Analysis

For cell harvesting, the medium was removed and cells were washed with phosphate-buffered saline (PBS). After PBS removal, cells were incubated with 1.8 ml sample buffer (8 M urea, 2 M thiourea) and detached with a cell scraper (Greiner Bio-One). Prior to disruption, cells were shock-frozen in liquid nitrogen and stored at -70°C. For disruption, cells were initially shock-frozen in liquid nitrogen and then defrosted in a thermomixer (Eppendorf, Hamburg, Germany) at 1400 rpm at 30°C for 10 minutes. After five freeze and thaw-cycles, samples were centrifuged (20,000 × *g*, 60 min, 4°C) to remove cell debris. Supernatants were transferred into new tubes and stored at −70°C prior to further processing. Protein concentrations were determined using a Bradford assay (Bio-Rad, Munich, Germany) as previously described [41].

### 2.4. Two-Dimensional Difference in Gel Electrophoresis (2D-DIGE)

Proteome analysis was performed for two separate experimental series of two conditions (control, 120 s of plasma treatment) and six time points (0, 0.5, 1, 24, 48, and 72 h). Protein lysates were labelled with Cy dyes (Cy3 or Cy5) according to the manufacturer's instructions (GE Healthcare, Munich, Germany). In order to reduce inter-gel variations, an internal standard pool consisting of 50 *μ*g aliquots of all samples was generated and labelled with the Cy2 dye. 50 *μ*g of all samples or internal standard was labelled with 400 pmol of corresponding dye on ice in the dark for 30 min. Reaction was quenched with 10 mM L-lysine for 10 min under the same conditions. From all samples, four technical replicates labelled either with Cy3 dye or with Cy5 dye were analysed. For a two-dimensional difference gel electrophoresis (2D-DIGE) approach, two labelled samples (Cy3 and Cy5, each 50 *μ*g) were mixed with the internal standard (Cy2, 50 *μ*g) in rehydration buffer [42] and applied to IPG strips (pH 4-7, 24 cm, GE Healthcare, Munich, Germany) by in-gel rehydration. After rehydration overnight, isoelectric focusing (IEF) was carried out as described earlier [42]. Subsequently, the second dimension (SDS-PAGE) was carried out using low-fluorescent glass plates (GE Healthcare) in the PROTEAN Plus Dodeca Cell system (Bio-Rad, Munich, Germany). After 2D-PAGE, the Cy2- (internal standard), Cy3-, and Cy5-labelled proteins in each gel were visualized by using a Typhoon 9400 laser scanner (GE Healthcare) at 100 microns by using the following excitation/emission wavelengths: 488/520 nm (Cy2), 532/670 nm (Cy3), and 633/670 nm (Cy5). The resulting images (3 per gel) were processed with dedicated software (Delta2D, Version 4.2, DECODON GmbH, Greifswald, Germany) as described below in detail. Time-resolved proteome analysis (0, 0.5, 1, 24, 48, and 72 h) was performed for untreated (control) and CAP-treated samples in two separate experimental series including short-time observation (0, 0.5, and 1 h) and long-time observation (24, 48, and 72 h).

### 2.5. Statistical Analysis

Analyses of the scanned gel images and data analyses were performed with the software package Delta2D (Version 4.2, DECODON GmbH, Greifswald, Germany). After aligning all image sets in position by using the internal standard (Cy2), all samples of a set were merged in order to create a fused image (2D proteome map) in union fusion mode [43]. Spot detection and editing were performed with the Delta2D software on the union fused image, and then spots and spot labels were transferred onto all other images included in the analyses. TMeV software (implemented in Delta 2D) was used for statistical analysis and graphical display of expression profiles. Acquired spot expression values were automatically normalized, first by correcting differences between intensities of all gel images and then by calibrating spot volumes (Cy3 or Cy5) to the internal standard (Cy2). In order to identify differences in spot intensity, the values of time-resolved CAP-treated samples were divided by the corresponding baseline values of the untreated control. Stringent selection criteria were employed in order to reduce false-positive results, and changes were only considered significant when the following three criteria were fulfilled: (i) the change of the intensity ratio had to exceed a factor of 1.5, (ii) the *p* value of the corresponding analysis of variance test had to be lower than 0.05 (one-way-ANOVA), and (iii) raw values of each spot had to exceed 0.3, avoiding calculation of ratios of spots close to background intensities. After TMeV analysis, principle component analysis (PCA) [44] was performed to validate reproducibility of experimental conditions and identify possible outliers. Each subset of sample (four technical replicates per group) was defined as point in an *n*-dimensional Cartesian coordinate system, described by 1504 vectors, which represent the amount of expression values of every detected protein spot. Those samples were transferred into a 2-dimensional space by focusing on the principal components with the largest variances. A *k*-means clustering [45] into 10 different groups, representing different representative expression patterns observed in the time-resolved proteome analysis, was done. For that purpose, the *z*-scores (mean centring) of expression values were calculated as follows: *z* − score = (single expression value–average of all expression values of one protein)/standard deviation. The resulting spot-ID lists were exported and analysed using the Ingenuity Pathway Analysis (IPA) software package (Qiagen, Hilden, Germany) for network analysis. This approach enabled creating protein networks and pathways, indicating changes in intensity and thereby placing individual affected proteins into its physiological context.

### 2.6. Preparative 2D Gel Electrophoresis and Sample Preparation for Mass Spectrometry

Preparative two-dimensional gel electrophoresis was performed as previously described [42]. Briefly, 450 *μ*g of protein was pooled from treated and untreated samples of each condition (75 *μ*g each) and added to the rehydration buffer. Resulting 2D-PAGE gels were stained with colloidal Coomassie Brilliant Blue according to the manufacturer's instructions (GE Healthcare). Digital documentation of the gel images was performed by a transmission light scan. Gel image analyses were performed with the Delta2D software package (DECODON GmbH, Greifswald, Germany) as described above. Spots of interest were processed for identification as described by Eymann et al. [46]. Briefly, spots were excised by using a spot cutter (Protein Works, Bio-Rad, USA) with a 2 mm picking head and transferred into 96-well-microplates, which were loaded with 100 *μ*l of distilled water (LiChrosolv, Merck KGaA, Darmstadt) in each well. Tryptic digestion was performed automatically in an Ettan Spot Handling Workstation (Amersham Biosciences) as well as the subsequent spotting of peptide solution onto MALDI targets. In detail, the gel pieces were washed twice with 100 *μ*l 50 mM ammonium bicarbonate/50% *v*/*v* methanol for 30 min and once with 100 *μ*l 75% *v*/*v* ACN for 10 min. Then, samples were dried for 17 min and incubated with 10 *μ*l trypsin solution (20 ng/ml trypsin (Promega, Madison, WI, USA) in 20 mM ammonium bicarbonate at 37°C for 120 min. For peptide extraction, gel pieces were covered with 60 *μ*l 50% *v*/*v* ACN/0.1% *w*/*v* TFA and incubated for 30 min at 37°C. Supernatants containing peptides were transferred into new microtiter plates. Peptide extraction was performed again with 40 *μ*l of the same solution. Joined supernatants were now completely dried at 40°C for 220 min. Peptides were dissolved in 2.2 *μ*l of 0.5% *w*/*v* TFA/50% *v*/*v* ACN, and 0.7 *μ*l of each solution was spotted directly onto MALDI targets. Then, this sample solution was mixed with 0.4 *μ*l of matrix solution by aspirating five times. After drying for 10-15 min, samples were measured using the MALDI-TOF/TOF instrument.

### 2.7. Matrix-Assisted Laser Desorption-Ionization Time-of-Flight Mass Spectrometry (MALDI-TOF-MS)

MALDI-TOF-MS measurements were performed with a Proteome Analyzer 4800 (Applied Biosystems, Foster City, CA, USA). Reflector mode was used in order to record the spectra in a mass range from 900 to 3700 Da with a mass focus on 2000 Da. Twenty-five subspectra with 100 shots per subspectrum were accumulated for one main spectrum using a random search pattern. An automatic internal two-point calibration was performed, when the autolytic fragments of trypsin with the monoisotopic (M+H)^1+^ *m*/*z* at 1045.564 and 2211.104 reached a signal-to-noise ratio (S/N) of at least 10. The standard peptide search tolerance was set to 50 ppm. The peak lists were created and searched automatically by using GPS Explorer software package (Applied Biosystems, Foster City, CA, USA). These peak lists were compared to a UniProt Swiss-Prot database (Rel. 51.5 restricted to human taxonomy) by the MASCOT search engine (Version 2.1). Positive identifications had to reach all of the following specifications: (i) sequence coverage of at least 30% and (ii) twice a MOWSE score of at least 49. Proteins and peptides, which failed the 30% sequence requirement, were reanalysed with manually measured MALDI-TOF-MS. The MALDI-TOF-MS/MS analyses were used for the five strongest peaks of the previous MS spectrum. Here, 20 subspectra with 125 shots per subspectrum were accumulated using a random search pattern. For peak list interpretation, the same database tools were used as mentioned before. Results reaching a MOWSE score of at least 49 in reflector mode (MALDI-TOF-MS) and being confirmed by subsequent measurement of the strongest peaks (MS/MS) were regarded as positively identified proteins. The confirmation by subsequent measurements (MS/MS) was particularly useful for protein identification in spots, which contained multiple proteins. Protein identifications and statistically relevant data were combined via unique spot IDs using the MS Repo database software (DECODON, Greifswald, Germany). Categorization of the identified proteins was achieved by using the PANTHER Classification System (http://www.pantherdb.org/) [47].

### 2.8. Network and Protein Functional Analysis Using Specialized Software

Expression profiles of statistically significant changing spots were exported from TMeV. These data were combined with identification lists from mass spectrometry and then imported into Ingenuity Pathway Analysis (Qiagen, Hilden, Germany). Using this program, it was possible to create protein networks and pathways, which contained the proteins displaying changes in intensity and thereby placing individual protein data into the physiological context. Additionally, Voronoi treemaps [48] were used for data analysis and visualization. Identified proteins were assigned to adapted KEGG BRITE orthology hierarchies and subsequently displayed with corresponding normalized expression values for each time point.

### 2.9. Immunoblot Analysis

For immune blotting, ten micrograms of protein from each time point (0 h, 0.5 h, 1 h, 24 h, 48 h, and 72 h) was separated by 12.5% SDS-PAGE and transferred onto an Immobilon-P PVDF membrane (Millipore) using a conventional semidry blotting device (Milliblot Graphic Electroblotter II, Millipore, Billerica, MA, USA) for 2 hours. Prior to antibody binding, membranes were blocked in a solution of 5% nonfat dry milk in TBS-Tween buffer (137 mM Tris-HCl, 2.68 mM NaCl, and 0.1% Tween 20) for 90 min at room temperature. Immediately after this step, the membranes were incubated with the different primary antibodies overnight at 4°C. To detect Hsp27, nuclear factor erythroid 2-related factor 2 (Nrf2), interleukin 1 beta (IL-1*β*), interleukin 33 (IL-33), and Kelch-like ECH-associated protein 1 (Keap1) as well as rabbit polyclonal IgG anti-Hsp27, anti-Nrf2, anti-IL-1*β*, anti-IL-33, and anti-Keap1 antibodies (Santa Cruz Biotechnology, USA) were used in a dilution of 1 : 100 (2 *μ*g/ml) in TBS-T buffer containing 5% BSA. Membranes were washed six times in TBS-T buffer before the incubation with the secondary antibodies at room temperature for 1 h. Stabilized goat anti-rabbit HRP-conjugated secondary antibody (Santa Cruz Biotechnology) was used in dilution 1 : 2,500 in 5% nonfat dry milk in TBS-T buffer. After six washing steps with TBS-T buffer, membranes were incubated with SuperSignal West Femto Maximum Sensitivity Substrate (Pierce; Thermo Fisher) for 5 min. Signals were detected with a Fusion-SL-3500WL instrument for fluorescence and chemoluminescence applications (Vilber Lourmat, Eberhardzell, Germany). Band intensities were quantified using the ImageQuant software version 5.0 (GE Healthcare). Statistical significance was determined using a two-sided *t*-test.

## 3. Results

### 3.1. Two-Dimensional Difference Gel Electrophoresis (2D-DIGE) Revealed Time Course-Specific Proteome Alterations of S9 Airway Epithelial Cells Triggered by Cold Atmospheric Plasma (CAP) Treatment

**Figure 1:**
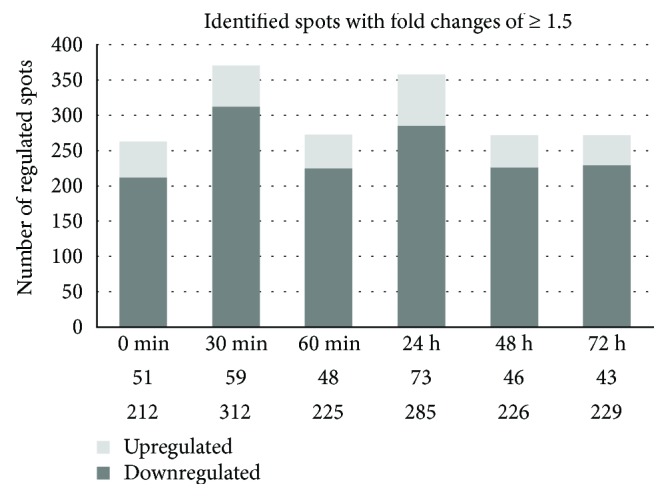
Number of CAP time-dependent (0, 30, 60 min, 24, 48, and 72 h) up- and downregulated protein spots in S9 epithelial cells. The number of identified significantly up- and downregulated protein spots (≥1.5-fold, *p* ≤ 0.05) is shown.

**Figure 2:**
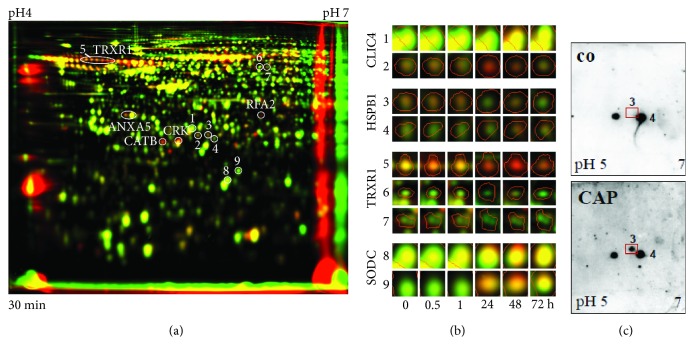
(a) Dual-channel image of whole proteomes separated by 2D-DIGE. Images of false-coloured separated proteins of untreated (green) and 120 s CAP-treated (red) S9 cells after 30 min were overlaid to create a so-called dual-channel image. Protein spots with increased amounts induced by CAP exposure appear as red or orange spots, and those with decreased amounts as green spots. Spots, which are expressed with the same intensity under both conditions, appear in yellow. Furthermore, several upregulated proteins are indicated (e.g., TRXR1, CATB) as well as the number of some examples for multiple protein spots as a result of potential protein modification. These examples are presented in detail and time schedule in (b); see CLIC4, HSPB1 (Hsp27), TRXR1, and SODC. (c) 2D-Western blot analysis of proteins from untreated (co) and plasma-treated S9 cells. Monoclonal antibodies raised against HSPB1 (Hsp27) was used. Note the appearance of HSPB1 spot no. 3 after CAP treatment.

As recently shown by Lendeckel et al. [17], S9 airway epithelial cells were clearly affected by the 120 s intensity of nonthermal plasma as demonstrated by (I) an increased wound healing rate in an *in vitro* wound healing model comprising plasma intensities (corresponding to treatment times) in the range of 30 s to 360 s and (II) the highest number of regulated protein spots revealed by a plasma intensity-dependent 2D-DIGE approach. This follow-up study was designed to provide a more detailed and time-resolved view on the proteome alterations of that specified CAP intensity (120 s) which seems to be tissue-tolerable and trigger more beneficial than damaging effects. Thus, CAP-treated S9 cells and nontreated control cells were harvested either directly after the end of treatment (0 h) or at different time points after subsequent cultivation in MEM (0.5 h-72 h). To analyse possible changes in protein pattern caused by this time course-related CAP treatment, a 2D-DIGE approach comparatively analysing time match samples of either treated or nontreated S9 cells immediately after CAP treatment or after subsequent 0.5 h, 1 h, 24 h, 48 h, and 72 h cultivation in MEM was performed. These time points including short-term (0 h–1 h) and long-term (24 h–72 h) observations should allow the evaluation and comparison of early and late molecular changes on proteomic level. Across the 48 resulting 2D-DIGE gel images, the average abundances of all detected spots were quantified, and for 1504 protein spots significant differences in intensity could be detected for at least one time point (one-way ANOVA) (Supplemental Table S-1). Those with relative changes in abundance greater than 1.5 times after CAP treatment (up- and downregulated proteins calculated as fold changes) at 95% confidence level (*p* ≤ 0.05) were further considered (1188 protein spots) (Supplementary Table S-2). Of those, 548 protein spots could be identified by MALDI-TOF-MS/MS and hence further analysed. Due to the facts that (I) many proteins were represented by more than one protein spot and (II) some protein spots represent mixed proteins containing more than one identifiable protein, our proteome analysis covered in total 422 different unique identified proteins/genes. Particularly for the short time course, immediately after CAP treatment, the amount of 263 identified protein spots was changed at least 1.5-fold. After an incubation of 30 min, 371 identified protein spots showed 1.5-fold altered levels, whereas 1 hour after CAP treatment 273 protein spots were changed 1.5-fold in comparison to the respective control for each time point. Representing the long-term effects, 358 identified protein spots were 1.5-fold up- or downregulated after 24 hours of incubation, 272 protein spots after 48 hours, and 272 protein spots which showed 1.5-fold changed intensity after 72 hours of incubation. The highest number of regulated protein spots was detected after 30 min and after 24 h; the highest number of upregulated protein spots was found after 24 h (Figure 1; Table S-2). In summary, our established protein index represented in Table S-2 contains 548 identified protein spots (= 422 different unique proteins) which displayed changes in intensity of at least 1.5-fold for at least one time point. The majority of the regulated protein spots displayed lower intensity after treatment and about a quarter increased in intensity. Alterations in protein amounts between untreated and treated samples could be also demonstrated in dual view mode by Delta2D software (DECODON) as shown for one example of the short time observation (30 min) in Figure 2(a). The appearance of the same protein in several spots is probably due to posttranslational modification (e.g., TRXR1 in Figure 2(a); see also below).

### 3.2. Posttranslational Modifications due to CAP Treatment Already Happened after a Short Time Probably in Consequence of Oxidative Stress

Compared to peptide-based LC-MS proteomic approaches, 2D gel electrophoresis offers the ability to monitor protein mobility and charge shifts resulting from putative posttranslational modifications like processing, degradation, and chemical reactions at individual positions (e.g., phosphorylation, oxidation). The appearance of intensity changes in multiple spots with the same protein identification by MALDI-TOF-MS indicates the existence of protein modifications due to the CAP treatment.  Figure 2(b) illustrates four different examples for possible protein modifications triggered by the CAP treatment resulting in changes of multiple spot patterns. The cytosolic thioredoxin reductase 1 (TRXR1) reduces thioredoxin (TRX), with both forming the thioredoxin system. In this system, electrons are transferred from NADPH via TRXR to the active site of TRX by reducing disulphide bonds of each protein itself, which are then used to reduce disulphide bonds of other protein substrates. A typical chain of protein spots and at least two further forms of the TRXR1 protein with specific *pI* values and/or molecular weights were separated on the 2D gel probably representing at least reduced and reoxidized TRXR states, whereat spot no. 5 as part of a whole TRXR1 protein chain was upregulated in comparison to the respective untreated control in contrast to the TRXR1 spots 6 and 7, which were downregulated. Further proteins, like CLIC4 (chloride intracellular channel protein 4), HSPB1 (HSP27, heat shock protein beta-1), and SODC (superoxide dismutase), appeared in two separated protein spots, respectively, in which one of them seemed to be upregulated and the other one downregulated. Probably, this protein shift is due to an oxidation of methionine or cysteine, leading to the formation of sulfonic acids and the creation of disulphide bonds. In case of CLIC4, posttranslational modification by cysteine oxidation has been identified by MALDI-MS (CLIC4: spot no. 1: AGSDGESIGNCPFSQR (*m*/*z* 1680.725) vs. spot no. 2: AGSDGESIGN**C**_**3ox**_PFSQR (*m*/*z* 1728.695)). Such a modification might be a result of oxidative stress triggered by the CAP treatment. As mentioned above, the observed TRXR1 modifications could occur due to its natural protein function in the thioredoxin system and therefore also in response to oxidative stress. For the heat shock protein HSPB1, the appearance of an additional protein spot (no. 3) was also confirmed by 2D Western blotting (Figure 2(c)).

To filter relevant processes and functions, which are affected by CAP and observed after a short or long term, further statistical and bioinformatics methods were used and are described in the following sections.

### 3.3. Principal Component Analysis (PCA) Uncovers Significant Short-Term and Long-Term Effects after CAP Treatment

**Figure 3:**
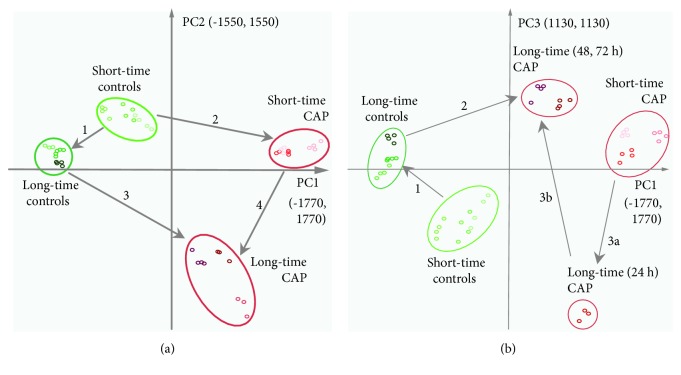
Principal component analysis (PCA). 2D plots of the first three principal components from the protein expression data are displayed, separating all analysed samples. Comparisons between the first and the second principal component (a) and the comparison between the first and the third principal component (b) are displayed. Ellipses subsume long- and short-time incubated samples and furthermore separate controls from the 120 s CAP treatment. Separation between controls and CAP-treated samples and separation between short-time and long-time observations are clearly detectable. Arrows indicate distances between the separated conditions. Arrows 1 and 4 represent separation between short-time and long-time samples. Separation between untreated controls and CAP-treated samples are represented by arrows 2 and 3 (a). Equal colour shades represent technical replicates of every condition (controls—green, 120 s CAP-treated samples—red; light shaded early: 0 h, 0.5 h, and 1 h; and dark-shaded late: 24 h, 48 h, and 72 h).

PCA was used to reduce dimensionality of data and to visualize significant differences between the proteomes of untreated and CAP-treated S9 epithelial cells or short- and long-term effects of CAP treatment. After reduction of dimensionality, the first three principal components described the highest variances of the data cloud from *n*-dimensional space. They represented 61.2% of data variance (PC1: 36.5%, PC2: 15.8%, and PC3: 8.9%) and distinguished the analysed samples in a very impressive way and further showed that replicates of the same sample are clustered together underlining the sufficient experimental quality (Figures 3(a) and 3(b)). In fact, the projection of only the first and second principal components in a 2D chart was sufficient to demonstrate that the proteomes of untreated and CAP-treated S9 cells showed significant differences. Also, a distinct effect of short- or long-term incubation could be observed (Figure 3(a), A). Thus, the cellular proteomes of the untreated control groups were indeed closely clustered on the upper left side of the 2D chart, but with a clear distance between the short (0 h, 30 min, and 1 h) and long-term (24 h, 48 h, and 72 h) incubated samples (arrow 1). Untreated controls were clearly separated from their respective CAP-treated samples (arrows 2 and 3). There was also a significant difference between short- and long-term incubated CAP-treated samples (arrow 4). An additional comparison between the first and the third principal components highlighted the pivotal role of the 24 h time point for CAP-treated long-term samples (Figure 3(b), arrows 3a and 3b).

### 3.4. PANTHER Analysis of Time Course-Dependently Regulated Proteins

The gene ontology (GO) terms associated with all identified time course-dependently regulated proteins after CAP treatment were examined using PANTHER. For 420 of the 422 identified proteins/genes, 586 hits for 11 different main molecular functions and 1071 hits for 16 main groups of biological processes could be found (Figure S-1). Catalytic, binding, and structural molecule activity appeared to be the most affected molecular functions, while metabolic and cellular processes followed by cell communication and developmental process are the most influenced biological processes. Consequently, PANTHER analyses revealed that CAP treatment caused proteome alterations after short- and long-time incubations including mainly proteins which are involved in common metabolic and cellular processes of catalytic and binding activity. There were only minor ranking differences between the PANTHER classifications of proteins affected by (I) that specified CAP intensity (120 s) over the short and long terms compared to (II) the proteins affected by three different plasma intensities over the long term only (see Lendeckel et al. [17]).

### 3.5. IPA Analysis Revealed Differences of Affected Cellular Functions and Pathways between Short-Term and Long-Term Incubated S9 Epithelial Cells Treated with CAP

**Figure 4:**
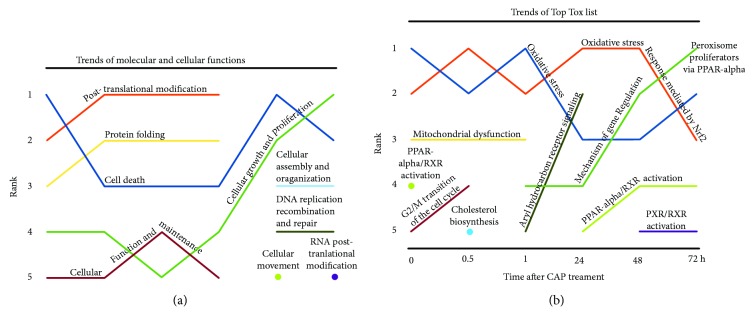
Molecular function and toxicity analysis assessed by Ingenuity Pathway Analysis (IPA). Statistically approved differentially expressed proteins (≥1.5 fold) from each time condition were assigned to networks. Top 5 rankings of molecular and cellular functions (a) and Top Tox lists (b) were plotted against the time points after CAP treatment. Relevance ranking shows time-dependent developments and importance for cell status at the specific time points. Detailed information including *p* values (<0.05) are shown in supplementary Table S-3.

Pathways and networks, in which CAP time-course-dependently regulated proteins are involved, were further analyzed using Ingenuity Pathway Analysis (IPA) to determine the precise affected cellular pathways in response to CAP and to uncover possible differences between the short-term and long-term incubated S9 cells. The IPA network analysis was performed with the protein index containing all identified proteins which were up- or downregulated ≥1.5-fold for at least one time point (see Table S-2). A ranking of the five most affected molecular functions (“Top molecular and cellular function”) and the five most determining stress factors (“Top Tox List”) was established (Figures 4(a) and 4(b)). Interestingly, also by means of these ranking trends, distinct differences between the short and long times could be observed.

Posttranslational modification belongs to the top molecular function which was affected early after CAP treatment (up to 24 h) and therefore could confirm the observations of multiple protein spots on 2D gel even under these conditions. Cell death-related proteins with altered expression dominated at the beginning of incubation (*t*_0_) became less important for the following hour and dominated again after 48 h. Regulation of some genes belonging to the category “Mitochondrial dysfunction” was only found during the short time (rank 3). The long-time effect after 48 h and 72 h was characterized by the increased influence of “cellular growth and proliferation”-related proteins in addition to the contrary functioning of cell death proteins. Simultaneously, proteins related to “Cellular assembly and organization” and “DNA replication, recombination and repair” were mostly regulated after long-term incubation only (Figure 4(a)). A ranking of significantly affected, functionally related protein sets showed that the most influenced toxicity responses during the short-term incubation (0 h, 0.5 h, and 1 h) are “oxidative stress” and “oxidative stress response mediated by Nrf2” as demonstrated by the reciprocal alternation of ranks 1 and 2 (Figure 4(b)). As recently shown for the long-term incubation after three different plasma doses [17], it is conspicuous that these oxidative stress pathways are the most influenced toxic responses after plasma treatment. In this time course-related study, we could clearly show that oxidative stress proteins belong to the most regulated proteins even at the beginning of incubation after CAP exposure. Later, not until 48 h, the expression of proteins involved in the “mechanism of gene regulation by peroxisome proliferators via PPAR-Alpha” was mostly affected. The strong influence of CAP on “PPAR-Alpha/RXR-activation” directly after the CAP impact might be a precondition for that. Finally, enhanced influence on “G2/M Transition of the cell cycle” only at early time points (0 h and 0.5 h) and not during the late times confirmed the determination of early- and late-term observations. The strong regulation of proteins involved in “DNA replication, recombination and repair” at later times (48 h and 72 h) might be an indicator for at least marginal DNA damage or quarrelling with DNA damages caused by oxidative stress.

### 3.6. Allocation of Different Protein Expression Patterns by *k*-Means-Clustering

**Figure 5:**
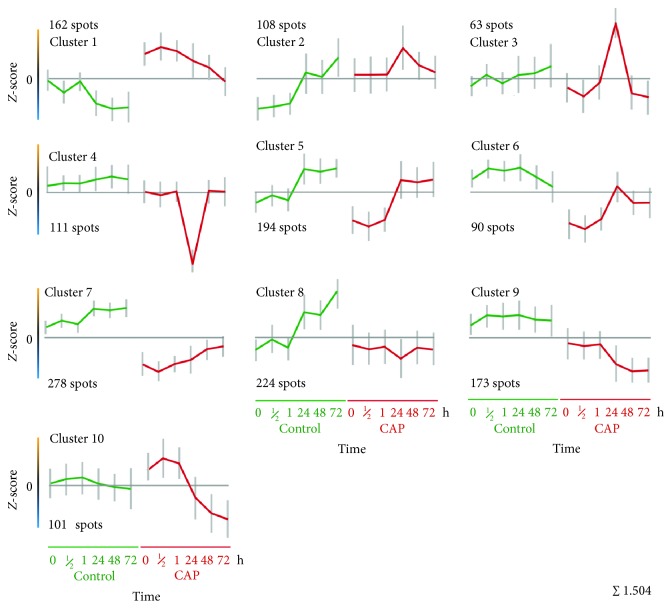
Protein expression profile analysis by *k*-means clustering. Ten different *k*-means clusters were computed by TMeV included in Delta2D. Expression profiles from every spot were compared with these given clusters and assorted to the best-fitting one. The *x*-axis shows the different groups of untreated and CAP-treated samples. From the left to the middle, controls in ascending incubation time from 0 h to 72 h are plotted. From the middle to the right, CAP-treated samples are plotted in ascending incubation time from 0 h to 72 h. On the *y*-axis, the expression rate (*z*-score) is displayed. Each value is marked with the standard deviation of assorted protein expression values for this specific time point. The horizontal line marks value “0.” Values above that line show higher expression, values below that line lower expression, respectively.

To find protein groups containing proteins with similar expression kinetics over all time points in untreated and CAP-treated S9 cells, a so-called *k*-means clustering was performed using TMeV. In this way, proteins might be classified that probably work together in concert and/or belong to similar pathways catalysing or managing similar reactions. A *k*-means clustering into 10 clusters (Figure 5, Table S-4) was calculated by the means of the Euclidean distance of the protein spots. Consequently, several expression features could be differentiated as follows: (I) the expression profiles between untreated controls and CAP-treated samples showed a typical offset supporting the results from PCA. (II) Four clusters include proteins with significant increased (cluster 1) or decreased expression (clusters 6, 7, and 9) after CAP treatment in comparison to the control. (III) Several clusters showed clear differences between the short- and long-term incubations after plasma treatment (clusters 6, 9, and 10) which are clearly separated from the control expression levels too. These profiles support the results from PCA as well as from IPA analysis concerning all regulated proteins (see above). (IV) Genes of cluster 5 showed a typical offset between short and long terms in the untreated as well as plasma-treated samples showing an increase in expression after 24 h. (V) Again, the particular features of the 24 h time point of CAP-treated samples are clearly represented by the two specific clusters 3 and 4 and to a minor degree also demonstrated in clusters 2 and 6. The single spot-ID lists of all proteins of each respective cluster with appending identifications (Table S-4) were exported and analysed in the following by using the IPA software package for network analysis. Supporting information like scores and *p* values are given in Table S-3.

### 3.7. Network Analysis of Calculated *k*-Means Clusters Using Ingenuity Pathway Analysis (IPA) Reveals Underlying Molecular Functions of Similar Expressed Proteins

Identified proteins resulting from respective *k*-means clusters were used for IPA network analysis to characterize pathways and networks. In contrast to IPA analysis based on all CAP-affected proteins (≥1.5-fold, 1187 proteins), whether induced or repressed by CAP at certain time points (see above), the following IPA analysis used all proteins of each specific *k*-means cluster (see Figure 5, in sum 1504 proteins). A ranking of the most determining stress factors “Top Tox Lists” and the most affected molecular functions “top molecular and cellular function lists” was visualized (see Table S-5), and their possible relationships to potential reactions during control or plasma-treated conditions or during short- and long-term observations after CAP were summarized and discussed. According to the expression kinetics of each *k*-means cluster (Figure 5), we could presume which pathways/reactions are relevant at a certain time point, in untreated or CAP-treated cells and consequently how cells might react to the CAP treatment after short and long terms. Summaries of cluster network analyses as well as scores and *p* values can be found in the supplementary material (Table S-5).

Cluster 1 contains proteins, which have significantly higher expression values at any time in comparison to the specific controls. Cluster 1 proteins correlate with functions associated to cell death, posttranslational modifications, and protein folding (ranks 1-3). The most striking influenced toxicity responses are “mechanism of gene regulation by peroxisome proliferators via PPAR alpha,” “PPAR alpha/RXR alpha activation,” and “Nrf2 mediated oxidative stress response.” The expression maximum of cluster 1 proteins is at 30 min after CAP treatment. Identified proteins with the highest *z*-score at 30 min with descending order are TCPA, 2AAA, CATB, GDIA, NUCB1, and PDIA3. TCPA is a molecular chaperone that is a member of the chaperonin-containing TCP1 complex (CCT), also known as the TCP1 ring complex (TRiC). This complex folds various proteins, including actin and tubulin which were also found as upregulated proteins by our analysis (ACTB, ACTG, and TBA3). Microtubule dynamics is an essential and highly regulated process required for cell viability, architecture, and division. Regulation of the microtubule network also depends on the maintenance of the *α*/*ß*-tubulin heterodimers' pool resulting from complex folding and assembly events, requiring the TCP1 ring complex [49].

Clusters 2 and 3 are characterized by an induction peak at 24 h after CAP, and their proteins seem to have functions concerning posttranslational modification and protein folding. Proteins of cluster 2 have an increased expression level after CAP treatment nearly over the whole time compared to the control (except for 72 h) and match to “cellular function and maintenance.” The cluster 2 proteins with the highest *z*-score are PDIA1, GRP78, GLU2B, and RCN1. PDIA1 is a multifunctional protein and catalyses the formation, breakage, and rearrangement of disulphide bonds. At high concentrations, it works as a chaperone that inhibits aggregation of misfolded proteins. As a potential cell surface protein disulphide isomerase, PDIA1 could alter the redox status at the plasma membrane [50]. Proteins of cluster 3 with the highest *z*-score are HYOU1, ATPB, and a second spot of GRP78. The protein HYOU1 belongs to the heat shock protein 70 family and is thought to play an important role in protein folding and secretion in the ER. Since suppression of this protein is associated with accelerated apoptosis, it is also suggested to play an important cytoprotective role in hypoxia-induced cellular perturbation (NCBI; [51]). As recently shown by Sepehrimanesh et al. [52], HYOU1 and ATP synthase beta subunit (ATPB) were also found to be upregulated after exposure to the radiofrequency-electromagnetic field in the rat testicular proteome. These proteins affect signalling pathways in rat testes and spermatogenesis and might therefore influence signalling pathways after CAP treatment too. HYOU1 fulfils together with the proteins GRP78 (HSPA5), PDIA3, and P4HB (PDIA1) the endoplasmic reticulum stress response. In patients with polyQ diseases caused by misfolded polyQ proteins resulting in severe oxidative stress and cell death, these ER stress proteins were downregulated [53].

Clusters 4-8 comprise proteins functioning predominantly in “Nrf2-mediated oxidative stress response” (rank 1). The expression is decreased after CAP in comparison to the controls. A more clear decrease over the whole time course could be observed in cluster 9 whose proteins also possess functions in oxidative stress response via Nrf2 (rank 2). Proteins of clusters 7 to 9 are linked to “cellular growth and proliferation” but also to “cell death.” The expression behaviour of cluster 9 proteins is comparable with that of clusters 7 and 8, also demonstrated by the correlation with “aryl hydrocarbon receptor signalling” in cluster 8. Examples of typically repressed protein spots of cluster 7 are HNRL2 and HNRPC which influence pre-mRNA processing and NP1L1 which is responsible for nucleosome assembly. Furthermore, several spots of the chaperone HS90A and the co-chaperone SGTA belong to the most decreased protein spots after 30 min of plasma treatment.

Cluster 10 is characterized by an increased expression during the first hour after CAP, and its proteins might be involved in “cellular growth and proliferation,” “posttranslational modification,” protein folding, cell death, and “cellular assembly and organization.” The main toxicity responses are linked to “cell death,” “decrease permeability transition of mitochondria and mitochondrial membrane,” and “oxidative stress.” All the proteins seem to have an important function during the first hour after CAP treatment. Later, the expression was switched off. The proteins CALM and HSPB1 (Hsp27) belong to the highest expressed proteins in this cluster. Calmodulin mediates the control of a large number of enzymes, ion channels, aquaporins, and other proteins through calcium-binding (http://www.uniprot.org). The water channel aquaporin-1 (AQP1) is expressed widely in mammalian epithelial and endothelial plasma membranes and seems to have an important role in migration and wound healing [54].

Table 1 summarizes the effects of CAP treatment of human epithelial cells and combines these effects with the detected and affected proteins of the cluster analyses. Thus, IPA analysis indicated that typical cellular functions and toxicity responses can be attributed substantially to either short-term (up to 1 h) or long-term (24-72 h) observations in response to CAP. On the other side, there is some evidence for a clear differentiation of relevant cellular responses that happened in untreated or CAP-treated S9 cells. During the first hour posttranslational modification, protein folding, cell death, “cell function and maintenance,” and “cellular assembly and organization” might play a dominant role in CAP-treated cells as long as these cells try to overcome cellular stress factors. CAP-treated cells have to deal with oxidative stress since proteins involved in “mechanism of gene regulation by peroxisome proliferators via PPAR alpha” and “Nrf2-mediated oxidative stress response” as well as oxidative stress possess higher expression values in CAP-treated cells compared to the untreated cells (clusters 1 & 10). In contrast, the untreated cells are characterized by a higher expression of genes whose proteins function in “cellular assembly and organization,” “cell growth and proliferation,” protein degradation, “cell-to-cell signalling interaction,” “cellular compromise,” and cell death (clusters 4, 6, 7, and 9).

Summarizing both IPA approaches, the first being obtained from all regulated proteins, the second based on *k*-means clustering, resulted in similar conclusions, but from two different points of view. While the first approach is related to the comparison between CAP-treated samples and their corresponding controls for each time point, respectively, the second approach is exclusively related to the time-dependent expression profile in untreated as well as CAP-treated cells.

### 3.8. Identification of Proteins That Were Specifically Induced after Short-Term or Long-Term Incubation in Response to CAP

**Figure 6:**
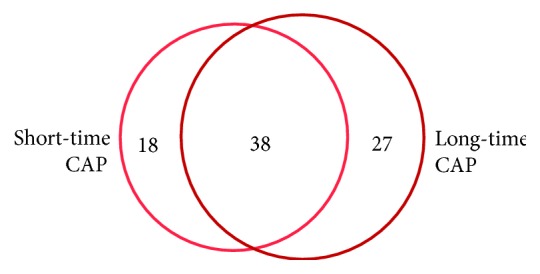
Comparison of upregulated proteins after short-term and long-term incubation of 120 s CAP by VENN diagram. The VENN diagram visualizes the overlap of upregulated single (identified) proteins induced in response to either short-term (0, 30, and 60 min) or long-term (24, 48, and 72 h) CAP treatment. Proteins that are solely induced by one respective time period could be considered as marker proteins.

To exemplify that there are distinct short-time and long-time specific protein alterations, we focused also on the upregulated unique proteins (fold changes of at least 1.5) and compared them by means of VENN diagrams (Figure 6, Table S-6). In contrast to our PANTHER and IPA analyses comprising all regulated proteins dominated by downregulated proteins, we filtered for proteins with increased expression compared to their respective controls. Providing that mainly the upregulated proteins contribute to the most needed and functionally active proteins, we wanted to know (I) which proteins are solely induced by the short-term incubation after CAP and could therefore be marker proteins for that time-period, (II) which proteins are specifically induced during the long-term incubation and could be considered as marker proteins for that period, and (III) which proteins were upregulated both during the short- and long-time incubation. As shown in Figure 6, 38 single proteins showed an enhanced expression either during the first hour and after 24 h and later. 18 proteins were solely induced during the first hour after CAP treatment and could therefore be considered as marker proteins of the early CAP response. These marker proteins are listed in Table S-6 and include for instance calmodulin-1, MARCS, and the peptidyl-prolyl *cis*-trans isomerase FKBP9, which bind calcium; proteins with chaperone function (CH60, HSPB1, PRS4, and TCPA); components of the nuclear lamina (LMNA, LMNB1), as well as an interferon-induced GTP-binding protein (MX1); and vimentin, a class III intermediate filament. 27 proteins are solely induced in at least one late time point (24, 48, and 72 h). Examples for late-time-induced proteins are ATPB, CALD1, GELS, HYOU1, and THIO as well as PSME1 and 2. Proteins that were induced both during short time and long time are for example the actin-related protein ARP3, the fructose-bisphosphate aldolase A (ALDOA) functioning also as a scaffolding protein, dynactin DCTN1, ubiquilin-1 (UBQL1), and the UV excision repair protein RD23B. Furthermore, the antiapoptotic factors ANXA5 and GDIA were found to be induced.

### 3.9. Time-Resolved Mediated Proteomic Changes after CAP Treatment Displayed by Voronoi Treemaps Confirmed the Appearance of Oxidative Stress after Plasma Treatment

In order to visualize imposing data, we used also Voronoi treemaps. Originally used to visualize hierarchical structures of software systems [55], an adapted derivative, previously described by Bernhardt et al. [56] and Otto et al. [48], can serve as a powerful tool for visualization of hierarchically structured protein functional classifications and expression data. In this study, KEGG BRITE orthology was used and adapted according to the proteins identified in this study. Approximately 550 identified protein species that correspond to 422 unique proteins/genes could be allocated to KEGG BRITE functional classes. First, the whole expression data set from Delta2D-analysis was used for expression-related colouring of Voronoi cells (first panel in Figure S-2A). The section “oxidative stress” including several oxidative stress subclasses like “Detoxification of ROS” or “Protein damage” was extracted in the following panel (Figure S-2A) and coloured separately for each time point (Figure S-2B). Colourization followed log fold changes of normalized expression values of CAP-treated samples divided by the normalized expression values of untreated controls. Negative fold changes (downregulation) are coloured in shades of blue, and positive fold changes (upregulation) are coloured in shades of orange.

As shown in Figure S-2B, the expression patterns of oxidative stress-related proteins changed impressively during the cultivation after CAP treatment. For example, members of the proteasome like PSME2 (proteasome activator complex subunit 2) were induced after 24 h. Protein disulphide isomerases like PDIA3 were induced during the first 24 h, and some heat shock proteins like HS90A as well as some proteins involved in the detoxification of ROS (e.g., TXND5) were induced for the whole time. These visualized results further suggest that CAP exposure caused significant expression changes in CAP-treated cells compared to the untreated controls. These effects and responses apparently affect metabolic pathways, which are related to oxidative stress. Furthermore, the 24 h time point after CAP treatment seems to be crucial for cell status and determination of cell fate, supporting the PCA results as well as IPA analysis that were described above.

All putative intracellular effects associated with oxidative stress like damages on proteins or DNA are summarized in Table 2. The represented proteins can serve as markers for the evaluation of the dimension of oxidative stress. For our analysed case, we can assume that the resulting oxidative stress is more moderate than hurtful but it is required to stimulate the wound healing.

### 3.10. Western Blot Analysis of Cytosolic Nrf2 Expression Confirmed the Pivotal Role of This Oxidative Stress-Related Regulator

**Figure 7:**
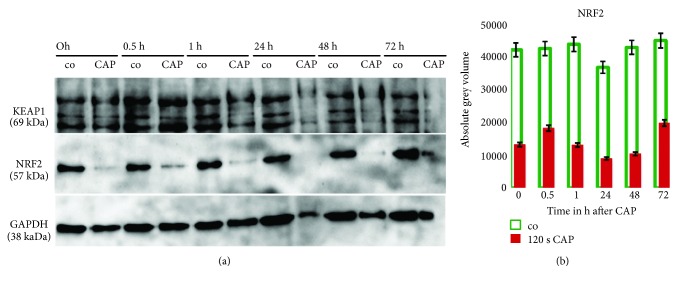
Validation of affected NRF2 and KEAP1 cytosolic concentration after CAP treatment by immunoblot analyses. (a) Cytosolic concentrations of NRF2 and KEAP1 in nontreated control (co) and CAP-treated S9 epithelial cells are displayed for each time point 0 h up to 72 h after treatment. Immunoblotting of NRF2 in S9 epithelial cell extracts shows that NRF2 disappears after CAP treatment while KEAP1 decreases after 1 h of CAP treatment. The membranes were re-probed with an anti-GAPDH Ab to ensure equal loading of the gels. (b) Results from quantitative image analysis of signal densities. Student's *t*-test opened statistical significance (*p* < 0.005) between untreated controls and CAP-treated samples regarding NRF2 expression for all time points.

As shown before, statistical analyses of the proteomic experiments revealed a pivotal role of oxidative stress response mediated by Nrf2 (master regulator of the antioxidant response) since genes that are linked to this pathway are induced (cluster 1 and 2) during the short-time and decreased during the short-time (clusters 5-7 and 9) or long-time incubation (clusters 4, 8). Although Nrf2 itself was not detected by the 2D-DIGE approach, some Nrf2-related network partners or Nrf2 targets could be detected. Thus, the Nrf2 targets GSTO1, SODC, and NADPH-quinone oxidoreductase 1 (NDUS1=NQO1) were identified by our analysis and found as downregulated. However, thioredoxin (TRX) was found to be significantly induced in response to CAP. The catalase CATA could not be found because of being out of the *pI* range in our 2D system. To confirm the effects of CAP treatment on Nrf2 expression in S9 epithelial cells, we performed an immunoblot analysis of the same protein extracts, which were used for the 2D-DIGE experiment using antibodies against Nrf2 and its trapping protein Keap1 which binds to cytosolic Nrf2 during the absence of oxidative stress (Figure 7). Levels of Nrf2 were significantly reduced after CAP treatment compared to control samples. During all time points analysed (0 min up to 72 h), the signal intensities of Nrf2 were far lower in treated samples compared to the corresponding nontreated controls (Figure 7(a)). Quantitative image analyses confirmed these observations with a *p* value of <0.005 for all time points (Figure 7(b)).

Because of the oxidative stress presumably triggered by CAP treatment, Nrf2 probably dissociates from the complex formed by Keap1 with Nrf2 and migrates into the nucleus, where it binds to other proteins and subsequently to the DNA [57, 58]. Therefore, the reduction of cytosolic Nrf2 levels in CAP-treated cells affirmed further findings concerning Nrf2-mediated oxidative stress response [59–66].

### 3.11. Cytosolic Concentrations of Full-Length IL-1 Beta and IL-33 Are Decreased in S9 Epithelial Cells after CAP Treatment, Suggesting Processes of Modulation of Immune-Related Cytokines

**Figure 8:**
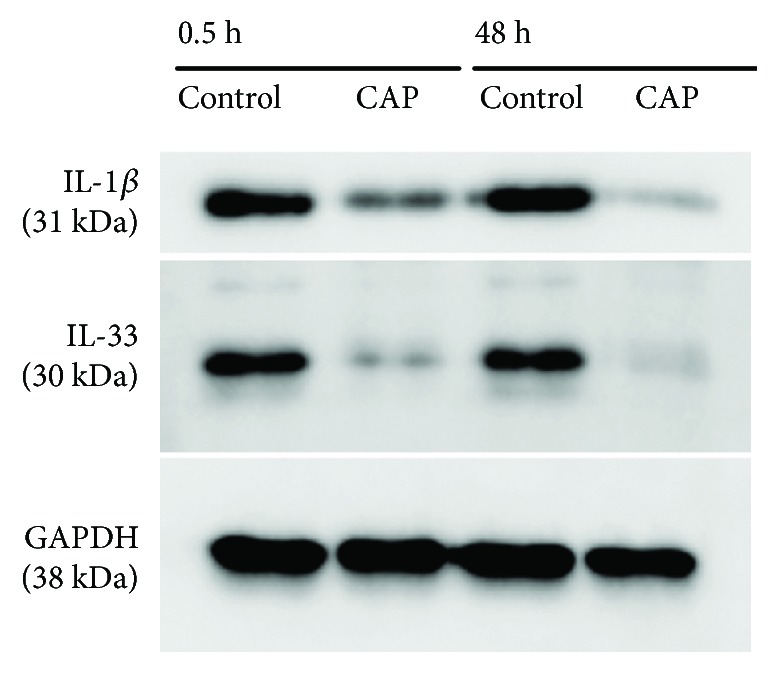
Influence of CAP treatment on cytosolic IL-1 beta and IL-33 levels observed by immunoblotting. Expression of IL-1 beta and IL-33 in nontreated and CAP-treated S9 epithelial cells for the time points 0.5 h and 48 h is displayed. Immunoblotting of S9 epithelial cell extracts show that both, IL-1*β* and IL-33 cytosolic protein amounts, are decreased dramatically after CAP treatment. The membranes were re-probed with an anti-GAPDH Ab to ensure equal loading of the gels.

We analysed interleukins IL-1 and IL-33 as potential immune modulators which could also play an important role in wound healing and its inflammation phase. They were described as proinflammatory cytokines, which furthermore are associated with immunological responses [67, 68]. They are known to be processed under proinflammatory conditions via caspase-1 to the particular mature forms, which function as intracellular transcription factors as well as secreted cytokines, which target T-cells and other immune-related targets [67–74]. Immunoblotting of intracellular cytosolic full-length IL-1 beta and IL-33 revealed a decrease of these prointerleukins in S9 bronchial epithelial cells 30 minutes and 48 hours after CAP treatment in comparison to nontreated control cells (Figure 8).

## 4. Discussion

The promising potential of cold plasma treatment as a new therapeutic option in the field of medicine, particularly in otorhinolaryngology or surgery of the chest and generally in wound medicine, demands primarily the assessment of potential risks and the prevention of any future cell damages. Several effects of nonthermal plasma onto human cells regarding the wound healing stimulation, reduction of microbial decontamination [10–18], or destruction of cancer cells have been studied so far [19–23]. But there is still a need of an extensive, all-encompassing analysis on the molecular level to monitor the entity of plasma effects happening in the cells caused by interaction of plasma with cells and tissues. The aim of such a detailed molecular analysis is to unravel dose- and time-dependent beneficial and damaging effects to develop the optimal therapeutic way. As previously shown, wound healing of S9 epithelial cells was accelerated by a specified CAP intensity (120 s) in a clinically relevant extent suggesting a beneficial role in the postoperative period [17]. The current proteome study might now provide an overview of how this special CAP intensity affects the protein pattern of human S9 cells after the short and long term and might explain the previously observed improved wound healing [17] on the basis of particular protein regulations.

The need for the exploration of the interactions between nonthermal plasma treatment and living cells was recently emphasized [31]. Kalghatgi et al. investigated specific and hypothesis-derived markers for effects like increases in intracellular level of reactive oxygen species (ROS) or DNA damage [31]. Our proteomic approach complements and extends such a targeted approach. While ROS cannot be observed directly by our method, molecular and metabolic reactions of the cells may indicate the presence of ROS and demonstrate the influence of ROS on cellular structures. Furthermore, we monitored the strategies of living cells to respond to cold plasma stimulation and could define associated biomarkers and networks. These indicators might be subsequently used for risk assessment and quality management of application of nonthermal plasma sources in clinical settings.

Our results indicate that oxidative stress resulting from plasma generated ROS/RNS plays a dominant role after treatment with cold plasma. The time-related *k*-means cluster analysis and the control-related fold-change approach showed concordantly that oxidative stress and responses to oxidative stress, mediated by Nrf2, occur primarily during the first hour since respective upregulated proteins were found during that short time. However, the Nrf2-mediated oxidative stress response is also linked to clusters whose expression is downregulated during the short term and primarily after 24 h. Indeed, we could demonstrate by Western blotting that the transcription factor Nrf2 vanished immediately after CAP treatment from the cytosolic protein fraction. This disappearance is presumably caused by its release from KEAP1 and subsequent move to the nucleus (57-59, 61, 63, 64, 66, and 67). In our analysis, the function of Nrf2 as transcriptional activator was shown indirectly by the induction of the Nrf2-related proteins thioredoxin, thioredoxin reductase 1, peroxiredoxin 1, HS90A, actin, and TCPA. The Nrf2-related proteins GSTO1, NDUS1, and SODC were found as downregulated proteins. The H_2_O_2_-detoxifying catalase was out of our *pI* range and could therefore not be identified. Important consequences of oxidative stress are different damages and modifications on proteins, nucleic acids, and lipids. The observed potential modifications of some proteins (see Figure 2) must not play an obligatory physiological role but may result in permanent and sometimes irreversible structural changes, which may affect protein function and necessitate replenishment by new synthesis. Although we did not detect all damages of oxidative stress in detail, the identified mechanisms and networks support already described stress responses like heat shock protein interactions and DNA repair (see Table 2) [75, 76].

Besides the already mentioned Nrf2-mediated oxidative stress response (rank 3 in clusters 1 & 2), also gene regulation by peroxisome proliferators via PPAR alpha and PPAR alpha/RXR alpha activation (rank 1 & 2, cluster 1) are symptomatic for the first hour. Probably, the production of peroxisomes is induced to overcome oxidative stress, and the peroxisome proliferator-activated receptors (PPARs) of the nucleus might function as transcription factors for several subsequent expression events, for instance anti-inflammatory functions [77]. Obviously, the cells deal with stress and try to overcome this stress. The ultimate ambition is the protection of the cell integrity. That is why cellular processes linked to cell growth and proliferation are mostly downregulated during the first hour (clusters 5, 7, and 9). However, there might be also moribund cells since also cell death was found as a relevant cellular function in cluster 1 (rank 1). A plausible explanation for this observation could be that a portion of cells might be stimulated by CAP treatment to induce stress adaptation responses. On the other hand, some cells might be severely damaged by the acting stressor and activate the cell death signalling pathway.

Although 28 proteins could be classified by PANTHER analysis as somehow involved in apoptosis processes (see Figure S-1), we did not find clear hints for activation of apoptosis. Instead, prevention of apoptosis seems to happen since “decrease permeability transition of mitochondria and mitochondrial membrane” (MPT) represents the first Top Tox rank in cluster 2 and the second in cluster 10, and an increase of MPT was considered to be one of the key events in mitochondrial-dependent apoptosis. On the other hand, a decrease of MPT is needed for protection of cell homeostasis [78]. Furthermore, antiapoptotic factors (e.g., HSP27, ANXA5) are induced (this work, [17]) confirming the assumption of apoptosis prevention. Interestingly, there are lots of hints for the maintenance of microtubule dynamics. The molecular chaperone TCPA represents the protein with the highest *z*-score in cluster 1 and is induced 2.1-fold 30 min after CAP treatment. TCPA is a member of the chaperonin-containing TCP1 complex, also known as the TCP1 ring complex that folds various proteins including actin and tubulin (NCBI). Since actin and tubulin proteins (ACTB, AGTG, and TBA3) were also found to be upregulated in our analysis after CAP treatment, we assume that the cells try to preserve the microtubule dynamics which is an essential and highly regulated process required for cell viability, architecture, division, and also wound healing [79, 80]. Regulation of the microtubule network also depends on the maintenance of the *α*/*ß*-tubulin heterodimers' pool resulting from complex folding and assembly events, requiring the TCP1 ring complex [49]. Both actin and tubulin contribute to different cell processes, for instance, cell migration. Thus, lamellipodia as a two-dimensional mesh of actin builds the motor in the process of cell migration which is also a prerequisite for wound healing [79]. The positive influence of CAP on wound healing of S9 epithelial cells was demonstrated by our previous analysis [17]. Now, we can suggest that plasma induces migration and therefore wound healing also by induced expression of actin. Not only lamellipodia-dependent cell crawling over the wound gap heals epithelial wounds but also a contraction of a supercellular actin cable (“purse string”) that surrounds the wound, or some combination of these two mechanisms [81]. During the “purse string” closure, actin filaments associated with myosin II form a supercellular cable around the wound circumference, presumably linking cells through adherent junctions [81]. Further processes that were found in this actual time-dependent analysis might be responsible for the observed improved wound healing [17]. The wound healing process can be divided at least into 3 phases—(I) inflammatory, (II) proliferation, and (III) remodelling phase [82]. In addition to cell migration, we found also some hints for inflammation control since “PPAR alpha/RXR alpha activation” and “Hypoxia inducible factor signalling” were induced during the short time. “PPAR alpha/RXR alpha activation” was described as a mediator of proliferation induction in the early phase of wound healing [83]. Furthermore, the cytosolic amounts of interleukin 1 beta (IL-1 beta) and IL-33 belonging to the IL-1 family are decreased dramatically in response to CAP as demonstrated by Western blotting. It might be that these proteins move to the nucleus where they function as nuclear factors which are involved in wound healing [84]. Extracellularly, they function as cytokines. For IL-1, it was shown that low extracellular levels of this mediator are necessary for wound healing since abnormal levels prevent the healing process and promote the development of a chronic wound [85]. Undisputed, there is an indication for the ability of CAP treatment to modulate the immune response resulting in improved wound healing.

The upregulated proteins CALM and HSPB1 (HSP27) of cluster 10 are further indicators for stimulation of wound healing during the first hour. Calmodulin mediates the control of a large number of enzymes, ion channels, aquaporins, and other proteins through calcium binding and prevent mitochondrial permeability transition pore (MPT) building [86]. The water channel aquaporin-1 (AQP1) is expressed widely in mammalian epithelial and endothelial plasma membranes and seems to play an important role in migration and wound healing [54]. Future studies on the membrane proteome of S9 cells will analyse the expression behaviour of, e.g., AQP1. HSPB1 seems to play a prominent role in response to plasma treatment. It has been shown that (1) during oxidative stress, this protein functions as an antioxidant and (2) interacts with Bax and cytochrome c, thereby preventing mitochondrial-dependent apoptosis, and (3) HSPB1 has been characterized with the ability to regulate actin cytoskeletal dynamics during heat shock and other stress conditions [87].

Remarkably, our analysis indicates for the first time that cold plasma causes endoplasmic reticulum (ER) stress and triggers the “Unfolded protein response” (UPR) [88]. ER stress is elicited by misfolded proteins, which for their part are caused by the existence of ROS since the protein folding process depends strongly on redox homeostasis [89]. The ER stress response is indicated by the upregulation of the following proteins: HYOU1, GRP78 (HSPA5), PDIA3, and P4HB (PDIA1) whereby GRP78, PDIA3, and P4HB are induced during the short time but HYOU1 is induced first after 24 h but also present before. The increase in these protein amounts after CAP indicates the quarrelling of S9 epithelial cells with misfolded proteins and oxidative stress leading to the ER stress response or UPR whose primary aim is to restore this organelle's physiological activity [88]. This response is also elucidated by hypoxia, which was shown to be induced by cold plasma since hypoxia-inducible factor 1*α* (HIF1 *α*) was induced by cold plasma [90]. HIF-1 *α* is a subunit of a heterodimeric transcription factor and forms together with the aryl hydrocarbon receptor nuclear translocator (ARNT), the hypoxia-inducible factor 1 (HIF-1). Proteins linked to “Aryl hydrocarbon receptor signalling” were found as downregulated proteins after CAP indicating that HIF-1 target genes that encode proteins involved in the regulation of processes in response to aryl hydrocarbons are repressed.

Summarizing, our findings suggest that there must be a mechanism that either drives cells into cell death or mounts adaptive responses allowing survival after cold plasma treatment. These observations may also explain that cold plasma treatment can turn a chronic wound into an acute wound with a better healing probability [13, 91, 92].

Our statistical and subsequent network analysis also confirmed the results obtained from HaCaT keratinocyte cells by transcriptomic analyses [67, 93]. Thus, NRF2 was also revealed as a probable key player in cellular oxidative stress response in epithelial cells [67]. Both transcriptomic studies analysing the effect of the CAP-treated medium on HaCaT cells showed the upregulation of “oxidative stress response” genes, e.g., oxidoreductases, and the induction of several genes encoding transcription factors and regulators as well as nucleic acid-binding proteins [67, 93]. These factors (e.g., NRF2, AHR) could mostly not be captured by our cytosolic proteomic analysis because their functions and locations are restricted to the nucleus.

Oxidative stress, regardless if caused internally or externally, is often linked with ageing, disease, and cell death [57, 59, 94], but at least moderate levels seem to be important for wound healing maybe by inducing alterations in redox-sensitive cell signalling pathways. The conflict with oxidative stress is not only concerned with negative stress and damage but also a good training for cells and prerequisite for wound healing. Induction of proliferation that is also triggered by NRF2-signalling [59, 95, 96] further supports our previous result of accelerated wound healing in CAP-treated epithelial cells [17].

Our results concerning the wound healing also confirm previous results based on animal skin wound models. Here, some basic plasma effects on wound healing mainly transmitted via ROS and RNS were identified: (I) promotion of re-epithelialization and acceleration of wound closure, (II) reduction of inflammation by activation of body-protective mechanisms (*ß*-defensins and NRF2 signalling) as well as by recruitment of immune cells into the wound area, (III) activation of fibroblasts to induce the re-arrangement of the actin cytoskeleton and to promote the collagen matrix synthesis, (IV) activation of wound healing-relevant cytokines and growth factors in fibroblasts and keratinocytes, and (V) induction of neovascularization [18, 97].

Nevertheless, all observed effects need to be studied in more detail. The different proteins identified by our network- and time-related statistical analysis have to be verified by further targeted proteomic analyses (e.g., Western blot or MRM analysis) and could then be used as possible biomarkers in further studies to evaluate effectiveness and toxicities of cold plasma treatments or even for the design of new plasma sources. The hypotheses-free approach leading to these candidates is a clear advantage in biomarker screening. Those defined biomarkers will also help to characterize newly developed endoscope-based plasma sources for internal medicine treatments, for instance, in Otorhinolaryngology or Respiratory medicine.

Because of the detection of antiapoptotic activity in the presence of possible marginal DNA damages and the suggestion of plasma dose-dependency of these effects [17, 31], tools for the assessment of the associated risks are necessary before any application in daily medical routine can be envisioned. DNA damages can be mutagenic and may result in carcinogenesis. We could show by IPA analysis that the “cell cycle: G2/M DNA damage checkpoint regulation” is both down- and upregulated (clusters 7, 3). The G2/M transition of the cell cycle can be blocked if DNA is damaged, thus preventing mitosis with damaged chromosomes [98]. But the DNA damages seem to be marginally since the mitogen-activated protein kinase 2 (MAP2K2) and MAD1 (mitotic arrest deficient-like 1) are downregulated. Even if we did not detect direct damages of the ultraviolet fraction of cold plasma or of ROS interactions with the DNA, Kalghatgi et al. [31] provided evidence that nonthermal plasma treatment of only the cell culture medium caused in subsequent cell cultivations an increase in DNA strand scissions. This underlines the pivotal role of ROS-dependent DNA interactions. This fact may be considered for risk assessment and future toxicity valuation.

A therapeutic treatment with nonthermal plasma would be conceivable for different respiratory chronic diseases such as bronchial asthma, chronic obstructive pulmonal disease, and chronic rhinosinusitis as well as allergic conditions of the nose and lungs. Besides, the experimental setup enables translation for all various cell lines to extend areas for possible plasma treatment to all parts of chronic medical conditions.

A proteomic approach monitoring over 1000 protein spots can be considered as an in-depth method for characterization of nonthermal plasma-related effects and risks. Even fine adjustments in development of plasma sources and follow-up quality controls can be performed using proteomic approaches. Costs and complexity of analysis have to be reduced in order to establish a method that is suitable for daily use and routine. Multiple reaction monitoring (MRM) assays based on mass spectrometry for up to 50 target proteins derived from an initial in-depth analysis might be a compromise, fulfilling at least some of the requirements. Ultimately, a plasma treatment in cancer therapy has to be applied selectively without or only minor effects on surrounding healthy cells.

## Figures and Tables

**Table 1:** Assignment of protein clusters to short-term and/or long-term responses.

Response	Cluster	Protein spots with highest/lowest *z*-score	Molecular & cellular functions	Top Tox	Overall conclusions/remarks
Short-term increased	1	TCPA, 2AAA, CATB, GDIA, NUCB1, PDIA3 (↑)	Cell death	Mechanism of gene regulation by peroxisome proliferators via PPAR alpha	(i) Induction of peroxisome production to overcome oxidative stress (ii) Activation of transcription factors (PPARs) involved in anti-inflammatory functions (iii) Induction of Nrf2 and their target genes to deal with oxidative stress (iv) Prevention of apoptosis (v) Some cell death events in damaged cells (vi) Posttranslational modifications caused by oxidative stress (vii) Beginning of protein folding and repair (viii) Protection of cell integrity and cell homeostasis (ix) ER stress response (x) Maintenance of microtubule dynamics (xi) Control of proteins through Ca^2+^ binding (xii) Stop of cell growth and proliferation (xiii) Protein synthesis stop, elimination (→) Present enzymes have to function
			Posttranslational modifications	PPAR alpha/RXR alpha activation	
			Protein folding	Nrf2-mediated oxidative stress response	
Short-term increased	2	PDIA1 (P4HB), GRP78, GLU2B, RCN1, PDIA3, CH60 (↑)	Posttranslational modifications	Decrease permeability transition of mitochondria and mitochondrial membrane	
			Protein folding	Hypoxia-inducible factor signalling	
			Cellular function and maintenance	Nrf2-mediated oxidative stress response	
Short-term increased	10	CALM, HSPB1, PDIA3, PSB7, RD23B, RCN2, PRS4 (↑)	Cellular growth and proliferation	Liver necrosis/cell death	
			Posttranslational modification	Decrease permeability transition of mitochondria and mitochondrial membrane	
			Protein folding	Oxidative stress	
Short-term decreased	5	SET, BASP, SPEE, HS90A, FSTL1, EF2, CBX1 (↓)	Cellular growth and proliferation	Nrf2-mediated oxidative stress response	
			Posttranslational modification	Liver necrosis/cell death	
			Protein folding	Decrease permeability transition of mitochondria and mitochondrial membrane	
Short-term decreased	6	SSRD, CBX3, NPM, C1QBP, ERP29, ANXA2 (↓)	Protein degradation	Nrf2-mediated oxidative stress response	
			Cell death	Decrease permeability transition of mitochondria and mitochondrial membrane	
			Gene expression	Oxidative stress	
Short-term decreased	7	HNRL2, HNRPC, HS90A, SGTA, NP1L1, CNDP2 (↓)	Cellular growth and proliferation	Nrf2-mediated oxidative stress response	
			Cell death	Cell cycle: G2/M DNA damage checkpoint regulation	
			Posttranslational modification	Oxidative stress	
Short-term decreased	9	PPT1, ANXA3, VINC, SCMC1, TPM1, K1C17 (↓)	Cellular growth and proliferation	Hypoxia-inducible factor signalling	
			Cellular assembly and organization	Nrf2-mediated oxidative stress response	
			Cell death	Aryl hydrocarbon receptor signalling	
Long-term increased	3	HYOU1, ATPB, GRP78, SYG, PDIA3, SF3B2, PSME1 (↑)	Posttranslational modifications	PPAR alpha/RXR alpha activation	(i) ER stress response (ii) Refolding of misfolded proteins replenishment by new synthesis (iii) Stop of mitosis to ensure the repair of oxidative or DNA damages (iv) Some Nrf2-related proteins are downregulated (v) Characterized more by cell metabolism, e.g., AA metabolism
			Protein folding	Mechanism of gene regulation by peroxisome proliferators via PPAR alpha	
			Amino acid metabolism	Cell cycle: G2/M DNA damage checkpoint regulation	
Long-term decreased	4	U2AF2, CALD1, GSTO1, CTNA1, PNPO, RFC2 (↓)	Posttranslational modifications	Nrf2-mediated oxidative stress response	
			Protein folding	Aryl hydrocarbon receptor signalling	
			Cell morphology	Hypoxia-inducible factor signalling	
Long-term decreased	8	EGF1, GELS, SODC, SF3B2, GANAB, PARK7 (↓)	Cellular growth and proliferation	Nrf2-mediated oxidative stress response	
			Cell death	Increase transmembrane potential of mitochondria and mitochondrial membrane	
			Cellular function and maintenance	Aryl hydrocarbon receptor signalling	

Relevant cellular and molecular functions and Top Tox responses (ranks 1-3) revealed by IPA analyses are mentioned. Reasoned functions are indicated as well as some proteins with highest or lowest *z*-score.

**Table 2:** Description of (putative) intracellular oxidative stress effects observed after CAP and their representation by corresponding detected proteins.

Oxidative stress-related effects	Consequences	Immediate effects	Long-term responses	Examples from our study
Protein damages	Unfolding	Loss of function	Elimination and subsequent new production	Proteasome-related proteins↑ (PRS4, PSME1, PSME2)
	Misfolding	Heat shock protein interaction	Refolding and/or repair	Translational activity ↑ (CH60, ENPL, GRP78, HS90A, HS71A, HSPB1)
	Decomplexation/dimerization	pI shifts		Heat shock proteins↓ (HS105, HSP74, AHSA1)
		Shifts in molecular weight		PDIA 1, 3, 4, 6↑

DNA damages	Single-strand scissions	Detection mechanisms	SOS repair	MRE11↑
	Double-strand scissions	Damage marking	Apoptosis	PPP6↓
	Base modifications	Cell cycle arrest	Cancer development	MSH2↓
	Hydrolysed bonds between sugar and base			KU86↓, RD23A↓, RD23B↑, RUVB1↓, RUVB2↓

Detoxification of reactive oxygen species (ROS)	H_2_O_2_ detoxification	Catalase active	Regeneration of “exhausted” proteins	(Catalase not found)
	OH^●^ detoxification	Peroxidases active	Appearance of isoforms	PRDX2↓, 3↓, 4↑
	O_2_-^●^ detoxification	Superoxide dismutase active		SODC↓
		Thioredoxin system active		TRXR1↑, TXND5, TXNL5↑
		Glutathione system active		GSTO1↓, GSTP1↓, GLRX3↓

Membrane modifications	Membrane oxidation	Structural changes	Changes in permeability	
	Lipid peroxidation	Increased/decreased protein integration	Changes of the membrane potential	AL1B1↓
	Transporter proteins		Apoptosis	CLIC4↓

Possible damages of proteins, nucleic acids, and lipids may result in cellular responses, which are exemplarily listed as logical consequences. Corresponding identified proteins and their regulation modes (up- or downregulated) are indicated.

## Data Availability

All the data used to support the findings of this study are included within the supplementary information files (see supplemental Tables S-1 to S-6 and supplemental Figures S-1 and S-2).

## Abbreviations

CAP: Cold atmospheric plasma
ROS: Reactive oxygen species
2D-DIGE: Two-dimensional difference gel electrophoresis.

## Supplementary Materials

Supplementary Materials Figure S-1: Gene Ontology Categories Classification (Panther) of all identified regulated proteins according to their cellular functions. Proteins were assigned to 16 biological processes and 11 molecular function categories. Figure S-2: visualization of time-related effects by Voronoi treemaps of CAP-treated vs. untreated S9 epithelial cells. Identified proteins were assorted into the individualised KEGG-BRITE hierarchy followed by the extraction of the subclass “oxidative stress” (**A**). Oxidative stress-related proteins were displayed for the time courses from 0 h up to 72 h (**B**). Every tile in that structural hierarchy, representing one identified protein, was coloured by fold changes (logarithmic normalized expression values from Delta2D of CAP-treated samples divided by expression values of untreated controls). The colour gradient encodes expression changes: white-coloured tiles show expressions, which correspond to a fold change of “1.” Blue-shaded tiles represent proteins with negative fold changes (lower expression in CAP-treated samples in comparison to the untreated controls), and shades of orange represent proteins with fold changes higher than “1.” Saturation of blue and orange is reached at an expression rate five times higher or lower than the corresponding control. The six different time points 0 h, 0.5 h, 1 h, 24 h, 48 h, and 72 h are displayed. Table S-1: register of all 1504 significant protein spots of the S9 epithelial cells passed the one-way ANOVA test (*p* value ≤0.05). Table S-2: established protein spot index obtained from Delta 2D analysis. Table S-3: summary results from network analysis using Ingenuity Pathway Analysis (IPA). Table S-4: detailed information on proteins/genes of all ten *k*-means clusters. Table S-5: summary results from network analysis using Ingenuity Pathway Analysis (IPA) for each single cluster. Table S-6: upregulated proteins (fold changes ≥1.5) in response to either short-term or long-term cultivation after CAP treatment or both.
