# Supplementary material for: Improved Wound Healing of Airway Epithelial Cells Is Mediated by Cold Atmospheric Plasma: A Time Course-Related Proteome Analysis
Source: Oxid Med Cell Longev. 2019 May 19;2019:7071536. doi: 10.1155/2019/7071536 (PMC6541959; doi:10.1155/2019/7071536)
Supplement: Supplementary Materials — Figure S-1: Gene Ontology Categories Classification (Panther) of all identified regulated proteins according to their cellular functions. Proteins were assigned to 16 biological processes and 11 molecular function categories. Figure S-2: visualization of time-related effects by Voronoi treemaps of CAP-treated vs. untreated S9 epithelial cells. Identified proteins were assorted into the individualised KEGG-BRITE hierarchy followed by the extraction of the subclass “oxidative stress” (A). Oxidative stress-related proteins were displayed for the time courses from 0 h up to 72 h (B). Every tile in that structural hierarchy, representing one identified protein, was coloured by fold changes (logarithmic normalized expression values from Delta2D of CAP-treated samples divided by expression values of untreated controls). The colour gradient encodes expression changes: white-coloured tiles show expressions, which correspond to a fold change of “1.” Blue-shaded tiles represent proteins with negative fold changes (lower expression in CAP-treated samples in comparison to the untreated controls), and shades of orange represent proteins with fold changes higher than “1.” Saturation of blue and orange is reached at an expression rate five times higher or lower than the corresponding control. The six different time points 0 h, 0.5 h, 1 h, 24 h, 48 h, and 72 h are displayed. Table S-1: register of all 1504 significant protein spots of the S9 epithelial cells passed the one-way ANOVA test (p value ≤0.05). Table S-2: established protein spot index obtained from Delta 2D analysis. Table S-3: summary results from network analysis using Ingenuity Pathway Analysis (IPA). Table S-4: detailed information on proteins/genes of all ten k-means clusters. Table S-5: summary results from network analysis using Ingenuity Pathway Analysis (IPA) for each single cluster. Table S-6: upregulated proteins (fold changes ≥1.5) in response to either short-term or long-term cultivation after CAP treatment o [file 7071536.f1.pdf]

Figure S-1: Gene Ontology Categories Classification (Panther) of all identified regulated proteins according to their cellular functions. Proteins were assigned to 16 biological processes and 11 molecular function categories.

Figure S-1

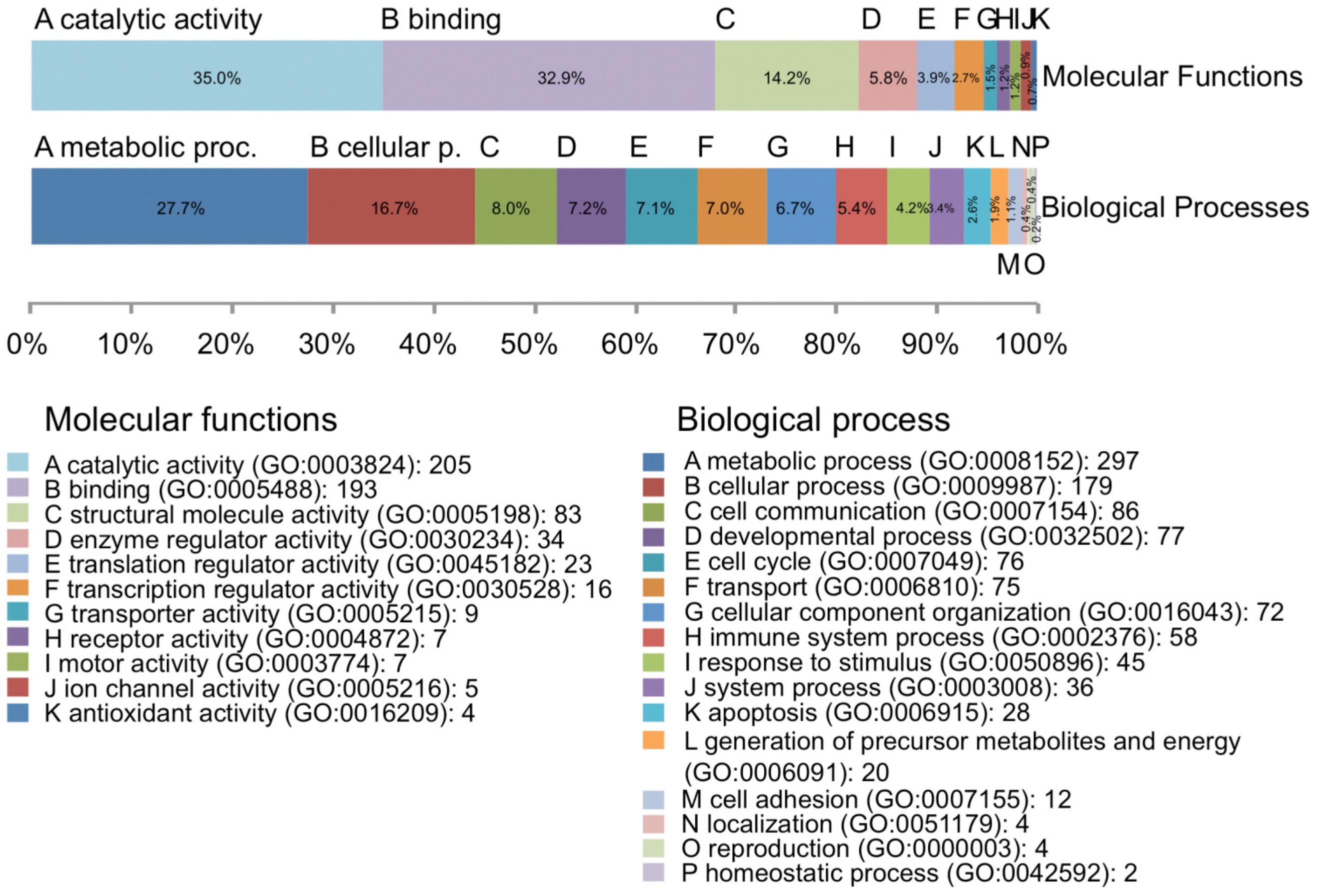
A catalytic activity
B binding
C
D
E
F
GHIJK
35.0%
32.9%
14.2%
5.8%
3.9%
2.7%
1.5%
1.2%
1.2%
0.9%
0.7%
Molecular Functions
A metabolic proc.
B cellular p.
C
D
E
F
G
H
I
J
KL
NP
27.7%
16.7%
8.0%
7.2%
7.1%
7.0%
6.7%
5.4%
4.2%
3.4%
2.6%
1.9%
1.1%
0.4%
0.4%
0.2%
Biological Processes
MO
0%
10%
20%
30%
40%
50%
60%
70%
80%
90%
100%
Molecular functions
A catalytic activity (GO:0003824): 205
B binding (GO:0005488): 193
C structural molecule activity (GO:0005198): 83
D enzyme regulator activity (GO:0030234): 34
E translation regulator activity (GO:0045182): 23
F transcription regulator activity (GO:0030528): 16
G transporter activity (GO:0005215): 9
H receptor activity (GO:0004872): 7
I motor activity (GO:0003774): 7
J ion channel activity (GO:0005216): 5
K antioxidant activity (GO:0016209): 4
Biological process
A metabolic process (GO:0008152): 297
B cellular process (GO:0009987): 179
C cell communication (GO:0007154): 86
D developmental process (GO:0032502): 77
E cell cycle (GO:0007049): 76
F transport (GO:0006810): 75
G cellular component organization (GO:0016043): 72
H immune system process (GO:0002376): 58
I response to stimulus (GO:0050896): 45
J system process (GO:0003008): 36
K apoptosis (GO:0006915): 28
L generation of precursor metabolites and energy (GO:0006091): 20
M cell adhesion (GO:0007155): 12
N localization (GO:0051179): 4
O reproduction (GO:0000003): 4
P homeostatic process (GO:0042592): 2

Figure S-2: Visualization of time-related effects by Voronoi Treemaps of CAP treated vs. untreated S9 epithelial cells. Identified proteins were assorted into individualised KEGG-BRITE hierarchy followed by the extraction of the sub-class “oxidative stress” (**A**). Oxidative stress related proteins were displayed for the time courses from 0 h up to 72 h (**B**). Every tile in that structural hierarchy, representing one identified protein, was coloured by fold-changes (logarithmic normalized expression values from Delta-2D of CAP treated samples divided by expression values of untreated controls). The colour gradient encodes expression changes: white coloured tiles show expressions, which correspond to a fold change of ‘1’. Blue shaded tiles represent proteins with negative fold-changes (lower expression in CAP treated samples in comparison to the untreated controls) and shades of orange represent proteins with fold-changes higher than ‘1’. Saturation of blue and orange is reached at an expression rate five times higher or lower than the corresponding control. The six different time points 0 h, 0.5 h, 1 h, 24 h, 48 h and 72 h are displayed.

Figure S-2

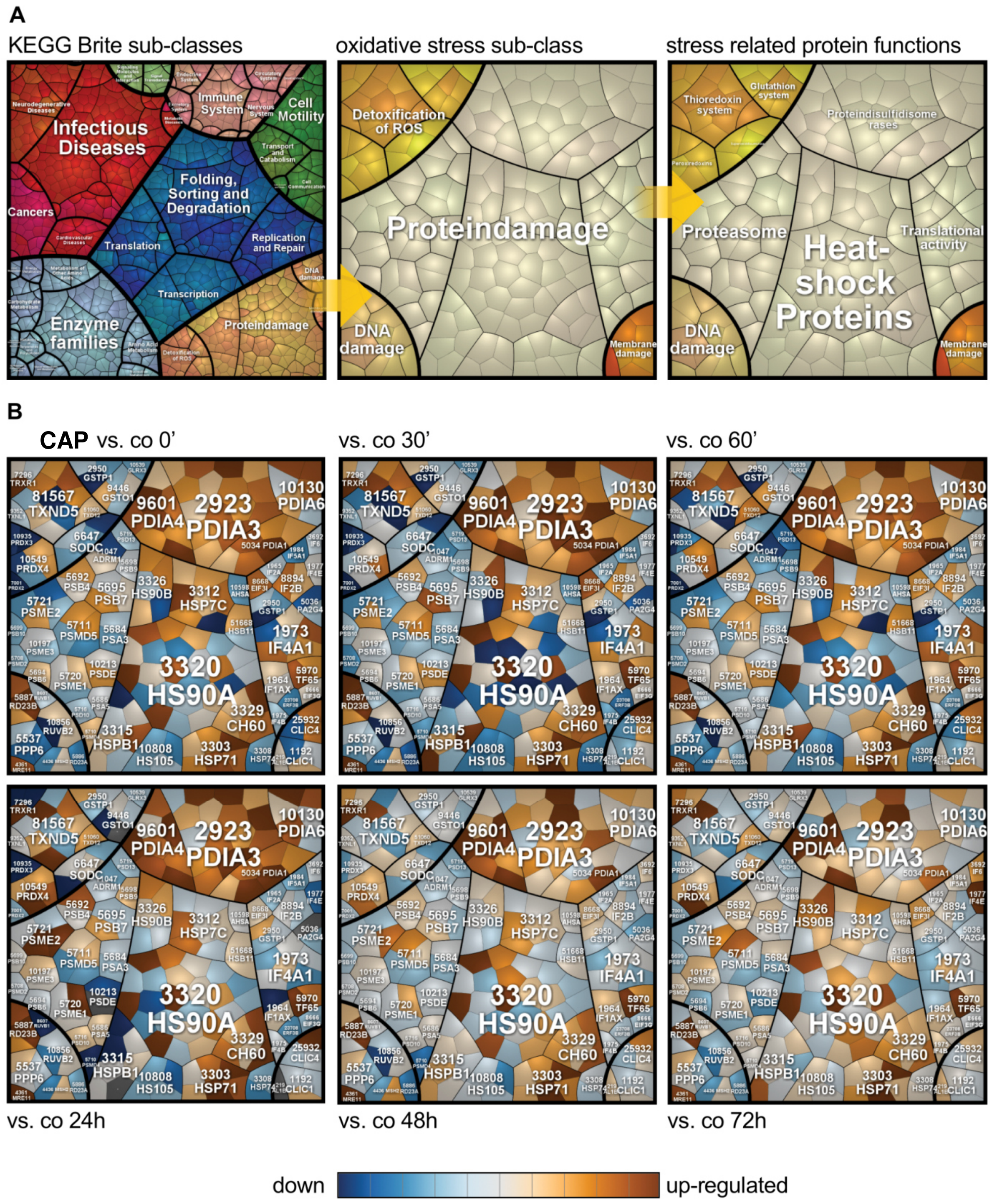
A
KEGG Brite sub-classes
oxidative stress sub-class
stress related protein functions
Infectious Diseases
Immune System
Cell Motility
Folding, Sorting and Degradation
Cancers
Translation
Replication and Repair
Transcription
Enzyme families
Proteindamage
Detoxification of ROS
DNA damage
Membrane damage
Thioredoxin system
Glutathion system
Proteindisulfidisomerases
Proteasome
Heat-shock Proteins
Translational activity
B
CAP vs. co 0'
vs. co 30'
vs. co 60'
vs. co 24h
vs. co 48h
vs. co 72h
down
up-regulated

Table S-1: Register of all 1504 significant protein spots of the S9 epithelial cells passed the One-way ANOVA test (p-value ≤ 0.05)

)1 hit ID obtained from Delta2D analysis (Delta2D statistically software version 4.4, Decodon (Germany, Greifswald))

)2 protein identification corresponding to human proteins (UniProt-SwissProt database; Rel. 51.5 restricted to human taxonomy)

)3 Median of normalized volumes of four technical replicates obtained from Delta2D analysis after 0, 30, 60 min and 24, 48 and 72 h of untreated (co) or plasma treated S9 epithelial cells (Pl)

Table S-1

		Median of normalized volumes)3											
ID)1	Protein)2	co_0 min	co_30 min	co_60 min	co_24 h	co_48 h	co_72 h	PI_0 min	PI_30 min	PI_60 min	PI_24 h	PI_48 h	PI_72 h
ID1449	1433B,1433G,TIF1B	95,583745	99,69177	103,72439	86,402165	88,133715	99,632305	55,551505	53,11662	65,341705	63,92361	60,095935	57,949365
ID1403	1433E	78,885515	83,20139	86,293905	75,572935	77,971885	85,833295	50,179895	48,66315	55,842215	51,6318	49,722	49,761455
ID1391	1433E,CLIC1	96,41815	103,53994	104,064015	123,003225	118,67649	124,557025	72,216435	76,73614	83,320225	79,08991	82,70916	88,41856
ID1425	1433G	120,286435	125,86943	133,46587	121,056245	120,714685	112,44005	72,454745	70,206145	77,769385	84,98135	81,55185	89,012665
ID1438	1433S	86,18513	92,022485	88,261085	107,51618	108,9057	106,98616	56,234135	58,940915	65,79851	71,36245	69,821605	68,70838
ID1429	1433T,1433Z	79,13656	80,325945	88,866215	85,961805	86,448315	89,204765	40,29503	40,772145	47,790725	51,284	51,614215	54,94
ID1430	1433Z	70,63055	73,807055	81,0351	76,965945	79,090805	86,357345	44,81633	44,854425	52,33692	56,42529	55,64717	58,06992
ID801	2AAA	120,62813	104,26214	124,05182	103,117365	108,28765	109,717835	115,563675	138,82353	125,866765	120,78811	118,76194	105,495
ID767	2AAB,UBQL1,IRGQ	110,51536	74,60654	108,66553	52,33244	53,24823	54,261375	198,125935	254,125055	217,184715	219,31879	203,33396	157,712605
ID1399	3HIDH,PSME1,CDV3	111,70553	115,16838	121,444185	101,334735	123,074455	130,02768	66,67329	60,215145	75,13423	77,35806	80,18045	75,344735
ID13892	ACL6A,IF4A1,HNRPF	135,09347	141,983725	136,327515	163,02464	156,33716	155,69761	179,51714	148,353835	164,616595	111,25791	118,072745	109,850905
ID651	ACPH	110,674885	125,908705	117,607055	111,16107	113,738295	118,55773	68,132545	95,18738	81,54194	66,29675	72,930445	70,297855
ID1114	ACTB	119,045045	101,45345	122,71457	86,604115	74,310005	66,790465	165,03791	188,885555	167,619065	164,34858	126,1945	112,739915
ID1070	ACTB	114,797295	115,92455	121,190745	124,20143	102,403455	102,715765	86,439305	84,954845	89,305855	90,99874	96,560005	95,599585
ID3255127	ACTG	92,039815	91,247385	92,56812	102,803955	79,7036	90,54841	81,39246	61,43024	63,371945	119,57176	74,44322	91,77801
ID1067	ACTG	98,787155	96,29072	86,91779	85,56691	128,727165	81,45378	95,13788	95,59521	94,95239	40,09193	91,74688	104,67174
ID1074	ACTG	67,425435	68,75858	73,25127	98,421175	99,354425	105,823015	71,584605	73,053075	68,6988	91,51088	83,33608	91,13239
ID360270	ACTG,ACTB	120,39944	114,79356	138,84618	99,478525	83,90349	70,557745	54,132895	81,26083	45,08136	98,16308	64,370965	60,6808
ID209100	ACTG,ACTB	91,790225	78,60895	99,047455	73,48615	65,5567	74,75669	29,62944	3,14729	17,755585	51,97919	47,170155	48,704125
ID1008	ACTG,ACTB	116,727295	117,34665	131,352825	124,09166	103,249095	135,52755	103,444125	107,958145	107,216935	92,94628	80,289215	85,652305
ID1073	ACTG,ACTB	30,35926	32,209565	35,372305	84,587225	74,45414	98,67245	52,85941	47,703035	46,383105	91,04608	61,559005	64,1947
ID1055	ACTG,IF34	67,595235	75,161285	62,132555	145,82536	94,56551	78,73915	70,44114	111,226575	115,020135	117,42206	118,28154	101,66423
ID420	ACTG,ITA3	104,967375	111,30103	109,34333	107,132515	115,317615	123,67495	97,53957	86,81829	92,08713	138,44188	88,32583	87,923995
ID1034	ACTG,K1C18	98,98775	103,048915	109,55228	96,58122	94,111645	108,484025	157,47854	149,445085	143,937095	83,78375	79,85701	74,247615
ID1064	ACTG,K1C18,ACTB	100,372195	95,29378	87,48099	87,44067	130,167835	85,364765	102,38816	104,31595	104,153215	69,66777	95,69628	105,169695
ID1080	ACTG,K1C18,ACTB	136,81236	144,457625	144,607915	129,20401	116,69489	116,7168	102,417365	102,946195	91,92182	79,06768	68,78114	75,930405
ID1035	ACTG,K1C18,CHD9	153,08116	163,161045	169,448915	130,627775	115,87038	115,567965	179,763815	191,35154	152,22413	91,14517	82,49422	81,313335
ID1053	ACTG,M6PBP	69,607395	68,490365	67,192605	88,57011	91,81142	100,5113	65,02444	63,441405	61,147785	78,34205	76,09358	76,949415
ID1478	ACTG,NNMT,CLIC4	91,397815	101,53196	103,724695	76,432575	87,727105	101,72447	109,40965	140,624935	120,852315	213,25293	97,55407	92,25573
ID1227	ACTG,PP2AB	107,115915	115,53131	111,34801	118,92167	98,315115	115,07728	77,794475	73,206635	78,728595	89,45868	88,628085	86,53644
ID14770	ACTG,SET	42,869405	47,49576	44,297885	93,60357	84,69448	93,89303	62,695645	60,87807	55,236175	81,85278	68,61775	72,515445
ID8247	ACTN4	105,447385	109,45465	107,49598	144,87208	158,033905	137,002435	78,35333	57,754575	59,657675	49,49417	76,701525	75,6417
ID517	ACTN4	80,792015	82,643665	86,575755	103,41759	104,712145	107,1561	57,737045	54,81945	53,03088	59,86058	61,649855	69,39307
ID518	ACTN4,ERO1A	105,99698	109,36112	111,59853	125,46613	122,74742	106,652125	62,475295	57,481755	60,46805	58,75529	64,750735	73,820535
ID507	ACTN4,HS105	121,141285	120,70519	126,96749	135,090325	136,99573	110,02421	62,66819	57,63865	60,728025	58,94369	66,22891	73,355955
ID8700	ACTN4,MVP	153,62179	132,36322	143,315135	50,686505	43,84603	85,17763	70,8429	90,83199	65,92449	20,23373	30,19142	45,755465
ID1047	ADRM1,ACTG,TMOD3,ATPB	90,26858	106,59496	109,499605	156,37931	143,79252	128,19672	76,75899	67,67029	60,72272	105,20716	92,92917	97,20383
ID467907	AGM1	92,48203	97,6184	95,47238	136,318975	95,29444	155,46808	97,438655	96,082995	106,879155	119,70884	101,571735	104,357725
ID1077	AHSA1,ACTG,CSN4	113,359215	120,804145	124,83158	135,16834	111,803955	107,73788	63,97355	63,61545	77,83914	100,56131	84,05431	88,39943
ID169714	AIBP,CI032	116,272925	116,47616	127,276675	110,762045	122,49475	123,885455	71,661415	82,64155	83,706505	86,19316	83,431065	91,61101
ID894	AL1B1	75,661075	82,641315	83,075895	113,56278	101,650555	103,65787	63,32164	53,35733	68,650025	9,772045	88,1461	94,422635
ID939	AL9A1	111,34244	120,0291	113,62564	136,101895	136,745185	147,658415	65,177705	68,173475	77,06819	65,07484	83,42511	88,097555
ID1386	ALBU	121,9733	133,299515	134,950155	129,42683	124,975845	116,95644	66,129895	82,443485	81,248885	82,4658	82,31701	77,699885
ID1828633	ALDOA	174,31809	95,551085	152,082475	57,58039	51,478315	49,50115	256,804925	310,91157	271,38924	200,84968	179,920235	120,50965
ID2003479	AN32A	87,95697	89,944265	95,561025	86,87064	108,04136	57,90857	29,696785	64,851945	61,72417	93,38261	61,050715	59,357695
ID3661699	ANXA1,DCPS	62,945855	74,966975	73,17148	129,504795	106,80768	117,86464	85,56156	84,711825	66,391565	95,046	73,873325	71,37444
ID2588249	ANXA2,NACA	178,926025	296,538395	92,903685	106,563215	93,953675	106,28719	33,303035	61,541355	98,414315	72,85427	72,94783	63,92224
ID1351	ANXA3,CAPZB,IPYR	123,20518	134,675695	131,561745	136,733215	132,39348	122,598085	76,73548	78,09211	79,666015	79,51955	84,43618	90,808865
ID1293	ANXA3,IPYR	114,39635	122,044755	121,3162	123,454015	119,378115	122,04876	116,6875	115,385745	116,81648	110,16942	85,61138	90,877635
ID1343	ANXA4	129,415995	136,974375	141,16159	127,959615	123,699505	114,90777	133,77147	160,1581	143,856265	146,04996	94,30994	92,49936
ID1334	ANXA5	99,50621	102,37519	114,944915	78,842095	83,509185	92,583725	117,45996	113,61239	123,127855	87,41981	70,387995	68,80215
ID213992	ANXA5	98,84342	109,704125	100,98105	115,251765	112,42314	116,65547	57,796965	67,78417	73,48931	69,38365	70,488615	66,2952
ID1345	ANXA5,SFRS2	43,510995	43,1422	53,579875	55,62829	63,885405	86,698595	87,85766	89,655625	88,914375	108,31569	79,491325	74,111725
ID978	APMAP,SNX6	100,26037	103,9828	122,580095	120,0283	122,98922	112,89155	120,64579	101,149555	130,48525	95,96083	85,63032	81,347015
ID341707	APT	69,088775	72,87946	84,173305	532,134375	153,542665	133,72962	7,70409	11,66579	14,6401	122,92756	129,00311	131,9219
ID1569	APT	136,636765	148,4242	152,00712	133,638285	116,35277	115,57886	93,09067	104,107175	107,52269	107,19462	94,097705	92,70482
ID965	ARP3	113,070245	117,56086	116,06469	145,83567	136,58162	130,859235	66,919345	59,205315	70,222725	60,48846	71,148185	78,017115

Table S-1

ID[1]	Protein[2]	Median of normalized volumes[3]											
		co_0 min	co_30 min	co_60 min	co_24 h	co_48 h	co_72 h	PI_0 min	PI_30 min	PI_60 min	PI_24 h	PI_48 h	PI_72 h
ID3329884	ARP3,TADBP	7,431215	29,406965	38,207135	81,171965	67,00959	74,255215	411,49689	255,054145	211,04777	132,89043	101,24879	92,013885
ID1046	ARP3,TADBP	76,13053	91,509395	86,37988	101,16411	104,159365	128,82713	117,585435	108,47282	104,54741	80,24286	72,368795	72,402135
ID1152	ARSA1	111,261865	111,84274	122,934625	106,976005	116,536795	116,683145	59,673215	52,571335	68,09929	73,11252	80,737515	78,141835
ID1560	ATP5H,CBX5	94,912795	108,563165	99,842455	99,38217	97,92969	99,40477	134,91678	108,884015	139,263585	127,1413	91,61694	88,342425
ID3737	ATPB	90,630255	98,14623	86,89253	87,113775	87,288065	90,00916	78,79455	78,30762	90,03048	135,2042	89,662235	86,043835
ID48368	BASP	89,43558	139,240845	75,352395	125,588265	132,23631	167,82339	20,024455	1,378435	7,7186	39,74228	65,55506	56,63564
ID936	BASP	102,15652	133,72475	104,46685	142,32019	173,256145	138,77479	59,244045	39,698055	59,19063	76,22659	87,33546	92,75009
ID2010414	BCCIP,ACTG	85,658335	102,710555	79,659555	107,986	155,148825	165,64524	71,654415	73,602815	75,843175	77,44378	103,695105	68,395235
ID1596	BID,RBM8A	101,157275	117,78074	100,879155	154,1472	127,505955	110,115335	98,850585	69,63507	72,607965	72,64713	83,71618	55,405975
ID1183	BIEA,TALDO	95,855145	107,006315	101,694085	112,80782	109,50053	107,0155	82,452995	90,710355	83,93705	83,39726	81,79053	87,386745
ID951	BLMH,LA,PA2G4	112,21182	122,85627	118,55186	120,20284	111,819225	120,625565	72,72389	81,05263	74,8677	68,05314	82,124965	84,806655
ID1363	C1QBP	93,646195	105,01945	99,1404	93,1786	89,56901	86,34525	92,935285	89,055315	109,64179	89,43267	90,24653	89,737625
ID1354	C1QBP	86,262595	100,725705	85,481795	118,6061	107,718445	113,021425	89,857995	76,882435	83,81682	84,86429	75,940905	77,9249
ID488759	CALD1	108,278785	115,594385	114,709345	151,948465	166,440635	143,75288	72,989075	65,58606	86,825125	912,817445	70,317645	80,763545
ID1702	CALM	144,750755	142,272545	196,118	103,865775	104,25972	105,253535	111,610595	451,18055	170,125345	105,06413	87,68575	91,3685
ID2174	CALR	88,89839	94,143675	90,37074	71,348345	76,74707	88,812945	92,489985	96,61487	100,901525	103,23889	90,310635	82,41663
ID831	CALR	100,3225	109,943195	105,60792	84,763995	90,08078	102,90512	113,82303	114,618445	121,930695	106,41295	90,08304	80,055615
ID745	CALR,KAP0	132,967545	89,222035	110,12773	57,10753	50,25332	52,04415	249,335165	232,62972	249,314635	205,58277	184,166095	134,7817
ID897	CALR,NP1L1	77,30268	84,13628	76,926875	78,783175	91,102765	109,589085	77,384905	75,722675	83,75862	93,63933	87,66965	91,780805
ID925	CALR,TBB2C	90,236555	102,269115	93,182835	86,17494	92,68315	106,089175	85,299285	88,86317	94,208725	99,91644	79,906195	84,38399
ID2006762	CALU	131,90107	160,61074	113,155685	125,703475	361,623485	185,610255	55,06583	55,25619	69,6157	31,61237	9,272725	31,59375
ID54018	CALU	73,039995	85,10613	73,20304	84,805665	87,403155	119,47833	79,58905	77,304305	88,19899	101,90479	61,061495	62,25916
ID53317	CALU	68,39457	77,98631	70,25559	74,501575	80,19322	117,666735	74,495625	72,42241	77,91629	104,61575	67,45907	74,43177
ID1024	CALU	79,533165	92,47806	78,905295	84,125095	87,910255	110,21043	84,63079	77,75105	88,86502	110,32053	74,5384	83,13767
ID1027	CALU,ACTG	87,46535	100,33167	89,160605	78,4288	91,5432	124,496575	89,807705	89,79329	95,7084	113,69301	76,472005	84,447485
ID1037	CALU,ACTG	94,136675	111,832455	95,16477	94,4831	101,503825	102,56451	101,52827	87,060205	108,59487	130,63919	92,50638	94,71852
ID479360	CAP2	89,209255	93,9242	107,47995	94,151685	99,326895	99,25567	105,34914	102,367565	120,713295	0	82,120285	86,134245
ID496907	CAPG	96,244265	108,116155	111,70219	140,647435	126,128155	154,289405	48,730405	51,22292	53,478875	55,47173	70,738865	63,568025
ID1106	CAPG	102,137345	116,431555	108,36414	113,43871	115,91302	123,4474	76,266095	70,222935	81,549555	79,83099	84,30171	90,333885
ID1440	CAPZB	107,60373	121,140435	115,69242	123,671385	125,47666	132,855035	86,451895	87,34504	96,597605	102,4815	85,179945	83,141005
ID1512	CATB	154,356845	107,271995	140,150125	98,02715	87,71859	93,47577	273,2966	362,5067	318,674235	188,69923	137,326105	106,162765
ID152055	CATD,CLIC1	104,374905	113,82983	114,26925	87,19919	129,942165	143,75756	100,785015	102,60089	114,50027	79,25228	103,20238	102,18715
ID1204	CAZA1,NPM	94,428665	102,919515	99,26126	113,728325	99,017555	102,74606	83,31348	79,96957	81,270655	88,57521	84,01674	89,314095
ID17135	CAZA2	105,11475	116,78941	112,503885	120,343655	115,926345	102,243955	85,58128	86,98721	89,57414	88,19298	84,444805	84,454555
ID163898	CBX1	93,215055	104,789565	93,012215	134,625765	116,237895	119,545995	119,528695	83,13456	86,976355	92,68008	81,923875	76,83239
ID1547	CBX1	56,62253	68,34873	70,256205	134,28403	120,640235	118,39233	51,51179	31,806315	38,771245	92,27438	94,91418	104,72887
ID1618	CBX3	72,208945	100,497555	78,154525	102,639545	119,05052	114,346255	34,85149	17,606025	22,09393	10,53659	26,017005	18,93456
ID3270147	CBX5,CATB	103,799235	120,642695	104,428625	76,704825	84,070785	129,937355	118,785705	95,962415	94,111935	86,00869	137,641945	113,6269
ID1530	CBX5,CATB	117,231465	119,9153	118,43505	124,704685	124,870335	131,65322	140,38517	148,577535	156,16844	67,13026	82,238015	72,87787
ID941	CD2B2	97,13261	113,797295	102,233935	107,905815	123,269395	107,06249	62,8173	50,058995	67,98124	73,74442	60,18159	58,71192
ID958	CDC37,PRS6B,DCTN2,PDIA6	103,457955	113,996025	112,08284	118,285485	111,37668	109,321885	70,91879	66,850225	72,371105	77,16692	78,846555	80,47092
ID827	CH60	69,075915	74,129985	73,016465	78,514925	77,69317	83,19507	82,523445	82,092315	102,95863	113,34766	98,05541	94,690205
ID14100	CH60	90,71302	97,21192	94,1139	104,82316	97,240775	100,333255	85,258595	81,609895	87,255565	97,80907	96,477565	93,835465
ID826	CH60,STK3	72,4893	66,440935	78,854895	92,07595	85,39417	99,49253	110,842735	113,62821	123,817905	124,66712	101,05925	91,53991
ID783	CH60,TBAK,ACTB,HNRPK	102,079445	100,95122	97,058375	99,31262	98,454305	94,418835	104,049515	99,99538	103,065085	111,6302	100,98178	100,27718
ID1268	CHM4B,RPB3	101,04462	113,51302	106,531975	120,894105	117,576335	105,915895	80,291835	73,03954	78,92447	99,67449	93,53243	88,590905
ID3763000	CHM4B,RPB3	77,41477	88,20882	72,886755	118,494165	128,44789	106,040315	86,27229	73,629985	73,299765	91,27134	94,04571	82,64234
ID2212082	CHP1	79,845315	95,152035	79,20624	123,63509	120,938245	130,05337	92,67368	70,57802	76,05167	17,73311	75,55471	56,306195
ID1479	CI032	109,279115	112,503875	121,858285	126,11289	128,18463	129,51848	74,98522	86,62129	88,460145	97,46721	91,584615	106,042085
ID1378	CLIC1	122,674045	130,871615	134,21456	134,52677	128,13082	123,94757	58,18018	71,41566	76,815975	92,34422	86,21744	95,272815
ID1318	CLIC4,PPIE	88,29241	103,373755	91,0456	115,828605	110,32626	111,663415	105,64171	98,26085	101,21551	80,16291	88,00653	88,445485
ID909	CNDP2,HNRH1	113,120755	122,587005	120,50033	131,25651	134,838705	128,11857	70,30758	68,366885	67,112135	65,25281	66,455075	71,228855
ID3392731	CNDP2,PEPD	108,340715	113,07423	112,529885	117,330455	107,907405	125,127015	71,750165	54,892025	72,7578	82,41157	84,4601	85,00046
ID901	CNDP2,PEPD	110,853115	115,116135	113,45779	116,62858	120,169585	121,210175	77,20618	75,656265	82,52091	71,78713	71,968725	75,692855
ID1168	CNN3	97,015395	107,11035	100,653755	143,497215	122,86517	115,64696	78,71123	70,2695	71,361435	89,66518	85,359095	92,37834
ID1165	CNN3	88,919775	94,835535	96,582815	134,10254	126,560765	108,960685	76,16567	61,54912	58,51474	86,47401	78,67127	87,59172
ID1154	CNN3,IDH3A	113,43605	128,69911	114,59791	131,9018	110,24202	114,776885	74,215625	66,813525	73,386685	67,66917	85,848465	86,9083

Table S-1

ID)[1]	Protein)[2]	Median of normalized volumes)[3]											
		co_0 min	co_30 min	co_60 min	co_24 h	co_48 h	co_72 h	PI_0 min	PI_30 min	PI_60 min	PI_24 h	PI_48 h	PI_72 h
ID1641	COF1	122,447965	126,15801	118,001095	140,78635	133,709505	134,622725	46,774995	47,800235	62,965135	82,61509	55,289885	62,043005
ID1341	COPE,ANXA5	137,825305	142,317385	135,95785	120,080265	119,34753	129,65528	167,76136	172,11798	152,736225	102,0077	92,33481	92,19387
ID295167	COPE,ANXA5	89,24158	93,5597	97,685625	84,91864	78,547155	81,87899	84,80662	75,223485	89,849425	68,51585	56,224885	56,44962
ID235608	COR1B	102,098415	94,58964	97,15092	108,968995	120,980055	142,204345	131,718105	119,26388	115,27618	112,94874	131,883405	107,02121
ID693	COR1B,CBR4	96,81786	92,74887	85,276405	79,00085	54,756955	73,971875	112,35536	87,414855	91,269195	115,88104	130,413675	132,722105
ID1769	COTL1	105,52968	115,130865	111,996685	112,368385	97,647655	95,515805	55,98173	57,35926	69,30251	87,10998	74,627015	88,691575
ID791	CPNE1	110,407575	114,49396	113,217375	129,20151	124,30859	130,5958	111,926175	115,44013	110,03673	74,87359	88,378495	85,57913
ID668	CPNE1,GRP75,PDIA4	109,50864	110,40697	110,982535	95,7526	81,8089	111,75223	121,18884	125,2212	125,793005	124,48206	100,833235	96,435545
ID808	CPNE3,CPNE1,PDIA3	81,12023	83,426965	86,307905	106,79141	97,66413	106,74998	89,936945	90,96556	93,538025	100,19078	101,31261	91,02602
ID1467	CPNS1	69,94976	83,301215	93,564525	112,464335	107,467165	100,76896	56,012215	92,041025	72,098525	96,12463	82,731155	88,772765
ID2001699	CPNS1	123,133295	128,41244	142,871835	128,10456	135,79822	155,87696	83,659095	98,146975	87,231145	105,20288	107,35467	120,172265
ID1401	CPNS1,GDIR	103,723145	114,38368	110,269175	109,449935	103,160815	105,499375	58,30643	64,76687	75,643815	72,40659	63,37635	65,763465
ID1502	CRK	184,941395	130,164075	172,626375	117,485915	90,30303	75,37607	288,345755	352,225805	311,838755	219,90246	134,48107	107,222015
ID565	CSDE1	97,50041	123,628705	102,050485	202,987585	164,18274	126,45567	72,121025	65,81105	80,66749	0	78,86991	68,4132
ID17270	CSN4	110,767805	116,869365	119,56591	113,37669	115,34023	121,015455	69,724985	58,34468	67,095035	78,6539	81,38041	93,596115
ID1099	CSN4,MPI,BPNT1	120,041935	126,385355	130,64259	128,74411	125,40209	116,78026	99,447705	76,57623	96,227375	123,45649	94,644325	95,94652
ID1723	CT077	42,13797	48,747915	46,449035	111,380025	138,44968	115,063855	50,73928	44,80194	62,962635	10,40025	85,858855	81,48284
ID26959	CTNA1	72,240745	79,18698	93,594185	132,814545	116,886705	125,94826	38,98242	33,24885	32,153585	0	33,45652	46,865125
ID650	DC1I2	108,100625	115,99056	108,17547	137,97351	126,793725	127,10919	80,43997	76,82725	77,8103	65,73129	65,312335	69,094915
ID327583	DC1I2	80,10508	85,900285	77,48305	93,34278	99,389015	104,45233	72,3791	67,333315	68,198715	92,96388	72,002815	69,91419
ID586647	DC1L2,TCPB	101,25123	107,1211	106,636975	143,934275	145,02769	127,574655	87,47939	85,72358	87,229155	41,16627	95,70171	97,209815
ID380472	DCTN1	80,007325	44,55774	76,046225	49,31849	29,241365	24,3214	197,041805	176,29227	207,22101	211,82989	209,285485	162,22811
ID942	DCTN2,PABP2	114,5817	128,771175	124,774395	117,909095	117,311075	119,512325	88,644505	86,514885	85,35819	95,30879	88,051005	87,664525
ID302775	DCTN2,PABP2,PRS6A	103,73667	110,88813	107,852545	120,39203	109,235655	107,460625	67,89339	64,650795	67,34142	87,90626	76,3999	78,899915
ID3348523	DCUP	109,924465	125,08233	119,65323	139,442535	110,65436	144,266435	72,72302	67,37775	76,700145	70,21872	94,097335	95,417735
ID902	DD19A,MPPA	100,025035	111,64799	105,62513	141,675975	132,19846	131,207725	84,41124	81,29284	87,99673	31,94169	70,33323	73,385305
ID893	DD19A,TBG1,PPP5	123,93303	130,411945	138,83092	151,98327	139,093735	132,55551	89,348775	85,70972	94,527055	24,68736	99,50442	108,41284
ID1259	DDAH1,CAZA1,LDHB,RLA0,NPM	78,808115	79,725665	86,885115	88,49741	87,77109	94,417635	50,503715	54,01715	53,13177	96,73163	79,163725	88,53064
ID1188	DDAH1,EIF3I,NPM	97,58566	99,13237	100,52592	107,006555	94,987265	95,62854	122,433025	121,28745	109,0216	86,93099	84,467795	96,192215
ID442	DDB1	91,59023	88,270455	100,536145	116,818305	115,9555	114,586515	94,7544	94,84224	86,94462	78,58566	82,01561	80,350195
ID129069	DDB1	75,71036	70,785965	79,71095	95,67477	98,749875	113,357385	87,91904	87,616485	80,488915	84,58425	81,54952	75,501845
ID436	DDB1,OXRP	105,00318	104,372165	115,524155	127,414485	123,54466	112,987965	98,7828	95,867665	90,46452	77,18807	80,522605	77,383185
ID1169	DJB11,ROAA	119,08758	128,80379	133,14005	137,754385	124,398745	119,650885	89,986795	95,74221	86,730845	106,715505	91,203545	98,02503
ID445851	DNJA2,APMAP,SNX6	81,55171	89,722125	82,35027	100,934595	87,98128	87,86359	69,182995	59,75365	60,661625	74,52982	72,24221	69,816445
ID983	DNJA2,APMAP,SNX6	70,682985	76,173765	68,306085	119,12082	114,43119	106,67182	66,54961	58,28902	59,6025	78,18022	77,76392	76,71447
ID1319	DNJC9	110,246425	139,303495	126,70546	139,43806	120,329435	123,72683	71,260165	89,378035	74,82667	64,0006	84,812295	84,61293
ID435879	DNJC9,PSME3	118,54565	127,88479	131,58246	105,26744	92,945145	83,908875	92,296855	95,62815	99,78669	91,74561	74,820875	76,235115
ID122740	DP13B	13,983775	20,98784	19,04432	72,27588	45,579005	91,71344	12,62676	13,90738	13,924005	296,10633	75,4305	121,36523
ID410	DPOD2	96,308125	111,04463	92,28487	141,04441	134,55087	144,36593	55,268495	36,683605	30,929215	31,18429	42,03278	57,263445
ID244116	DPOD2	99,32258	108,970225	96,57548	147,83193	159,780005	174,500725	51,72215	31,471715	22,97198	0,02609	13,76593	35,37919
ID13335	DPOD2	95,461415	93,799895	93,336795	156,183345	191,094315	211,235135	94,80849	87,697585	117,714785	65,59947	94,2679	96,524465
ID755	DPYL2	152,27617	101,429235	122,8146	57,355415	49,864715	52,102165	280,09564	293,87282	280,448965	208,70962	189,265175	138,3486
ID778	DPYL2,LKHA4	90,53427	104,96045	92,914925	137,670865	121,28035	112,41734	73,53944	67,08022	73,738195	14,64969	91,15042	95,844
ID644	DPYL2,ZYX,EZRI,CALD1,IMMT	56,551265	61,491265	63,334795	101,55699	83,852655	93,777785	46,034635	43,031855	47,401195	194,75407	67,490895	80,445025
ID624	DPYL2,ZYX,EZRI,CALD1,IMMT	90,85454	94,665105	94,371205	115,66195	114,71292	123,993825	92,00611	70,524	90,020855	0	81,28126	86,09103
ID435	DREB	95,156995	102,19118	94,108595	127,08493	108,647875	108,51848	66,5076	48,721645	56,30049	62,14855	72,556135	73,305815
ID381	DYNA	113,64283	115,255485	111,092355	133,363095	153,27024	148,456245	69,02119	55,24977	61,75276	55,04345	83,405095	73,482775
ID1352	ECH1,PSDE	113,253425	119,960115	123,75728	103,930065	121,859765	140,21971	123,98558	118,136165	128,0928	65,68563	86,89513	72,495525
ID1435	ECHM	110,33824	121,868355	117,331375	130,32028	135,769865	138,980625	100,049265	106,8465	120,78406	66,74133	83,807335	77,921885
ID1473	ECHM	77,96155	87,581165	84,52541	96,53631	90,380915	97,75736	59,628175	62,75373	68,79302	65,08193	96,87861	89,52324
ID64437	EF1B	81,36777	90,026875	80,725475	90,89321	88,64752	86,42319	68,371065	64,452695	75,83596	61,36695	68,4041	65,258145
ID59353	EF1B	41,438115	45,03286	42,56405	68,24956	66,183025	63,3929	38,4016	34,1678	41,07757	55,66135	57,21794	58,39774
ID1382	EF1B	85,846695	94,60124	85,082885	108,266295	96,533335	86,10452	74,954745	62,33091	69,259795	91,24666	78,430005	83,49917
ID1251	EF1D	76,54766	101,40915	78,92609	145,92142	103,085705	75,00444	96,95495	71,994865	75,737265	134,52919	90,423475	93,721895
ID1278	EF1D	99,748785	111,267195	99,595535	101,849045	94,559725	87,64875	91,911125	72,767205	87,866615	103,73728	79,081195	74,21261
ID546	EF2	52,90957	56,323265	58,58694	77,743305	81,985405	94,71372	46,92001	32,98245	39,994115	61,39176	67,026335	69,36238
ID610	EFG1	83,28244	86,55753	85,13953	132,506615	120,515245	120,7202	73,03933	61,99981	64,039765	34,819385	79,873605	83,2585

Table S-1

ID[)1]	Protein[)2]	Median of normalized volumes[)3]											
		co_0 min	co_30 min	co_60 min	co_24 h	co_48 h	co_72 h	PI_0 min	PI_30 min	PI_60 min	PI_24 h	PI_48 h	PI_72 h
ID1128	EI2BL,CAPG,ROAA	106,085505	119,03714	107,786985	124,72469	126,24613	132,386395	64,499025	63,730785	74,169605	65,47084	81,290615	93,511535
ID1078	EIF3G,ARP3,TADBP,ACTB	99,93757	108,32973	105,05015	134,456845	119,226245	113,10765	83,306335	81,804565	85,754145	77,95114	74,778045	82,522035
ID1913428	ELOB	125,54448	136,351715	148,61101	342,786665	453,26493	842,602905	99,02928	90,053345	100,050205	65,10389	166,01195	184,63571
ID1015	ENOA	132,289915	87,75459	117,119555	55,833135	51,78509	52,25965	283,46817	292,74734	267,07939	197,684	177,611245	131,75512
ID1001	ENOG	104,94079	117,347145	110,52766	123,06929	130,066275	135,545935	56,408055	66,25472	68,207345	67,91352	83,173545	85,12051
ID15652	ENOG	91,17362	105,172515	100,15314	116,625385	120,79445	125,54896	44,298515	53,879735	53,358265	77,02495	85,471995	82,29215
ID953	ENOG,RBBP7	93,070865	108,95896	94,62498	132,710535	122,6311	116,05562	85,342835	84,341425	84,63007	72,69305	79,301835	71,877025
ID274688	ENPL	70,095525	79,293535	72,56963	78,1079	69,310525	84,78929	85,15835	102,754575	91,42789	139,96314	77,18248	70,027495
ID104582	ENPL	112,07059	128,84972	120,24541	110,568495	116,211945	121,836265	97,154405	79,33534	106,110695	118,72813	92,77252	88,310535
ID132924	ENPL	49,704715	69,089075	44,01625	123,71895	125,15409	127,195985	100,6575	63,594355	108,76752	102,09717	88,48107	83,13858
ID462	ENPL	106,16827	100,00445	98,70222	93,2526	110,55937	88,882765	98,70881	94,29857	94,200995	119,94748	95,990125	87,676265
ID198671	ERO1A	124,46438	132,55691	145,135035	93,665145	104,90495	104,612115	124,69033	128,6047	135,28987	98,10031	75,735275	72,9607
ID2298466	ERP29,IF4E	131,77083	146,703425	131,974465	345,21417	127,13165	79,911775	105,062475	95,65111	96,810665	155,13132	93,03082	174,304465
ID1463	ERP29,TPIS,ETHE1	101,72378	113,874145	107,51804	117,36839	113,58969	106,36462	58,699915	69,014575	72,726655	69,34197	86,14798	82,737005
ID637	EZRI	97,66466	99,13942	99,897575	136,27566	119,81196	104,829325	71,617335	80,87928	81,7882	128,40934	87,390095	89,2336
ID625	EZRI,CALD1,SYK	79,285875	87,194655	86,09288	114,84912	102,08921	108,66978	59,202665	54,722475	62,590635	245,30616	78,58669	83,713995
ID957	F10A1,PDIA6,TXND4	104,159455	115,036325	111,169805	97,938505	99,91222	103,915175	105,084675	109,150775	109,337275	123,06403	87,670875	85,771905
ID191574	FKB10	79,067625	84,529315	83,050145	66,239	74,224265	72,552165	60,05311	50,363305	65,266135	48,57691	49,211575	58,41959
ID387435	FKB10	84,440585	89,33243	89,07973	102,79421	100,921915	144,820775	73,75215	50,81994	57,985715	66,45999	67,12181	74,8812
ID643	FKB10,DC1I2	99,842635	112,16365	101,90194	105,825615	96,834485	101,13478	73,7811	74,295335	76,573575	92,68039	83,25	82,03034
ID871	FKBP4	98,3785	110,715695	103,971095	118,41371	107,891965	110,110655	59,86644	55,44968	63,680325	85,25229	84,032355	86,996925
ID878	FKBP4,HMCS1,HNRPK	107,628655	119,62358	111,346125	132,752755	111,24614	103,11064	75,11219	62,92586	73,219785	93,00876	88,57577	90,886225
ID692	FKBP9	67,79747	80,75614	71,378025	87,49751	70,352895	133,96875	105,4673	99,77974	111,401325	80,76354	65,75608	52,66364
ID1173	FSTL1,ANXA2	81,287805	99,713885	69,131555	150,568805	143,26352	145,16537	99,28505	57,23515	122,96288	142,57138	90,23856	91,79898
ID1137	GALK1,ILEU,DNJBB,ROAA	97,201795	112,003595	101,955585	126,63899	123,56323	139,975575	114,1665	94,42572	87,12581	17,87545	74,668575	80,70339
ID1087	GALK1,ILEU,DNJBB,ROAA,SAHH	113,098385	126,1945	120,8	111,357605	106,402835	111,337395	118,30111	114,595155	125,016035	24,29573	98,495735	92,896145
ID460138	GANAB	81,81818	90,67608	83,717505	107,921125	110,05523	126,748205	73,33469	56,005275	50,71368	26,26938	51,405325	57,678395
ID496	GANAB	108,192685	116,482005	115,846225	105,202515	110,93363	130,688755	92,52415	94,336985	102,03854	73,66799	62,611045	61,292425
ID509	GANAB	85,733875	92,936155	89,46344	90,723555	94,607515	148,945775	82,310035	82,471725	80,782135	72,16065	56,62759	59,338235
ID4596	GANAB	96,92161	109,084265	86,278995	129,238265	120,775155	139,564905	42,0469	47,58142	43,7969	62,73942	64,339175	78,934205
ID510	GANAB,IMMT	126,945265	139,23663	138,328215	109,985715	109,669915	137,18108	89,05143	96,87711	93,19855	48,96262	56,49383	61,026595
ID499	GANAB,IMMT	103,16191	108,028305	116,56342	90,09344	99,472605	121,066615	90,26156	74,950695	92,636445	62,36323	58,801245	58,45663
ID825	GDIA,ANXA3,NUCB1	139,124495	110,925835	140,474555	94,94922	97,505855	92,46145	166,8889	220,9686	193,68113	178,51598	150,3906	121,892105
ID828	GDIA,VTDB,2AAA	133,04655	91,94065	131,00971	70,735475	72,35636	65,18205	208,54159	278,498915	241,357975	207,15806	174,57159	134,51286
ID940	GDIB	116,79346	121,191085	124,183635	148,68798	147,238695	137,913205	49,184155	52,340685	58,27932	74,30319	79,40922	86,84127
ID993	GDIB,SAHH,IDH3A	97,384715	101,933725	107,695385	102,643985	96,25093	97,706565	81,61075	83,186745	83,183835	72,90004	72,28509	78,812385
ID1513	GDIR1,LGUL	106,7343	118,108055	113,22415	124,503985	120,15317	120,878315	63,662515	68,571065	80,21645	87,82653	86,59842	85,575235
ID17482	GELS	135,7546	110,064425	128,194305	80,03332	110,602295	169,826425	161,96474	160,4447	189,96281	145,20878	159,512975	113,549295
ID584	GELS,MX1,HS90A	93,905675	104,93371	99,23075	116,55324	125,044605	135,415535	80,020245	79,48212	77,445565	47,11515	85,72387	80,34639
ID1166	GIPC1	111,700325	129,067155	126,35797	142,025405	125,103295	127,0282	80,380605	74,56105	86,86392	0	109,479615	121,05907
ID1167	GLRX3,TXNL2,SNAG,CPIN1	111,02994	120,57365	113,214015	134,0003	121,546775	122,54552	63,15505	61,324735	69,793785	81,28063	84,456465	91,191915
ID535	GLU2B	105,21014	118,23586	110,94032	92,01201	97,71002	103,788125	106,59636	101,405865	116,46459	124,14016	81,30747	74,519595
ID2111	GLU2B	73,33603	80,300265	73,48993	85,527605	75,631555	85,297665	82,06112	79,34985	89,791725	113,34191	86,11447	88,19912
ID3296127	GMFB	109,93477	133,2015	123,262345	99,74301	93,060155	111,046095	74,622825	76,64651	81,38354	62,75638	87,88126	118,91859
ID505	GRAP1,GANAB	50,595445	58,23906	47,00738	122,122875	107,21568	171,11769	41,515695	45,858845	36,966995	54,06436	57,13417	72,23364
ID309937	GRAP1,GANAB	94,35994	99,878325	72,720085	176,51725	119,636205	118,69415	29,538385	10,356745	9,32518	58,80149	67,71943	76,539465
ID1495	GRB2	101,85374	114,548855	105,25004	129,224815	112,65304	106,38032	78,532295	84,89627	92,5844	108,75762	90,003515	87,871625
ID3763691	GRP75	129,48729	136,84911	126,75166	125,538555	135,22256	106,667005	114,111695	108,4255	108,130785	110,03745	96,0373	94,384645
ID18275	GRP75,PDIA4	102,9421	111,39775	90,720595	92,948445	116,851515	103,973385	94,650085	80,578185	88,12959	114,93111	92,33198	90,673495
ID710	GRP75,TF65,PDIA4,HSP7C,HSP7	89,915825	96,542195	92,431215	100,22445	94,60582	105,34274	99,348005	93,24781	94,23544	114,31484	96,306345	94,08641
ID2845794	GRP78	107,38948	100,17752	117,928275	141,49643	140,22263	154,019205	127,86085	178,622745	129,80813	212,24352	137,90799	133,11296
ID2846419	GRP78	62,90503	49,842765	73,044085	93,237615	108,88093	125,168455	70,10446	53,17403	41,647915	96,10396	80,78925	75,994385
ID10719	GRP78	73,76355	118,049565	80,57081	75,234895	120,709025	68,551485	152,26049	42,659085	238,099685	154,68278	108,95272	88,42386
ID672	GRP78	57,96031	49,519635	55,013195	92,568385	98,241505	100,081785	84,33462	75,426145	66,11048	118,78636	80,91476	77,97782
ID318	GRP78	124,05096	136,49043	123,592255	164,57516	157,32148	227,279	41,869175	45,263005	2,42067	34,90055	71,71889	101,9466
ID594	GRP78	102,642945	100,24216	117,59754	105,86482	97,989405	131,870935	157,8366	154,38678	153,207475	132,38028	115,24759	103,66654
ID658	GRP78	96,196245	96,781145	99,57343	105,957175	105,90628	95,633705	108,177795	101,48222	104,088045	151,24451	99,381655	96,76527

Table S-1

ID)[1]	Protein)[2]	Median of normalized volumes)[3]											
		co_0 min	co_30 min	co_60 min	co_24 h	co_48 h	co_72 h	PI_0 min	PI_30 min	PI_60 min	PI_24 h	PI_48 h	PI_72 h
ID3760	GRP78	20,05108	24,192205	31,12976	37,39717	31,442495	43,916615	34,65171	41,542305	40,901185	216,79369	92,235315	112,103235
ID366771	GRP78	74,446035	84,30294	60,86663	77,621035	98,37569	210,41836	154,20372	89,06232	129,426125	44,63832	60,14983	49,62885
ID661	GRP78	47,27308	53,99535	59,149795	60,49348	52,426145	56,666785	59,879825	73,685425	64,878545	172,12602	91,201035	94,48044
ID662	GRP78	79,99499	87,787145	92,59996	79,45162	76,21625	72,505315	83,511535	110,548635	91,675285	152,38431	92,680245	91,667185
ID105671	GRP78,ENPL	152,69661	143,80528	162,11817	96,56849	99,22915	95,2295	105,425255	179,444515	101,990825	112,12152	90,07343	113,73882
ID120878	GRP78,PDIA4	35,042515	39,446045	36,98826	79,166125	61,489065	81,46732	66,33739	37,53574	46,249085	63,43228	56,673645	47,968515
ID960	GSHB	108,177545	121,67428	115,47211	112,960725	116,52578	121,58411	64,84325	68,30363	71,893775	80,85368	86,274185	89,12308
ID943	GSHB,K2C8,DDX39	113,06965	123,330615	136,1384	102,61881	111,525275	104,08506	95,09351	66,96806	71,04716	51,58723	67,23913	59,62164
ID622	GSPT1,HS90A,ERF3B	115,432645	120,285575	114,035345	150,506235	130,786255	119,359725	60,682775	54,030025	62,204035	77,14953	84,050525	93,1898
ID1393	GSTO1	113,781315	135,040425	127,153835	119,58787	124,697785	124,35265	73,52388	96,864095	95,183985	0	93,72244	104,455755
ID1408	GSTO1,ERP29	100,271225	113,044415	106,12117	116,215125	116,96922	121,052785	100,27156	96,11949	112,81241	15,44478	87,54604	81,098595
ID422947	GSTO1,PSB7	107,92095	118,40206	117,26894	140,779605	112,476775	106,17534	90,948955	82,69164	90,707735	89,37616	68,777755	74,23645
ID1516	GSTP1	111,094495	122,660225	121,913525	126,35216	127,17935	138,43344	62,077905	74,629525	80,8604	85,31845	86,92964	93,907105
ID12547	HDAC1,HNRPK,CH60	115,01545	112,6409	125,244235	120,23893	116,4344	102,965155	135,04692	125,16267	112,493765	98,74317	100,74951	89,628565
ID1231	HDGF	134,47207	142,28801	123,11207	136,03602	109,044535	108,62374	58,57187	63,279845	67,507865	83,53764	96,105765	97,00226
ID1211	HDGF	111,890845	130,82146	112,9552	150,708175	113,938695	120,36501	69,349435	65,171005	72,371185	64,85317	72,41474	58,705505
ID88373	HDGF	109,057575	123,932365	113,0606	121,337105	109,429735	126,346155	59,803055	64,1119	68,3294	53,35838	73,244145	69,420965
ID1194	HDGF	102,20748	114,48601	105,04423	108,451945	110,391575	123,240165	60,04378	64,00371	67,060245	57,94046	74,253765	73,085165
ID217409	HEXB	106,99282	121,48155	116,473845	103,258815	111,88329	120,458055	133,54145	158,00282	139,81586	97,07329	80,423665	80,613375
ID923	HNRH1,CNDP2	96,82487	109,96634	106,45446	112,32273	115,34106	131,771555	104,774675	96,737035	88,812865	63,51034	61,18937	66,68006
ID447263	HNRH1,CNDP2	111,606235	119,558835	114,178925	109,868895	115,37623	105,578435	110,99925	137,06592	118,567915	67,04907	61,08742	72,611925
ID918	HNRH1,CNDP2,SNX6	104,70776	122,56118	115,402315	138,14821	130,40084	133,0849	102,285955	80,819215	70,13008	70,03228	79,360995	82,886735
ID971	HNRH1,GDIB,2ABA	98,20386	104,412135	103,621485	121,53555	114,678505	119,226705	71,024995	68,495505	70,84428	70,42163	80,024475	81,758245
ID29521	HNRH1,HNRH2	107,52559	127,29003	117,772325	142,77507	132,90265	139,46523	94,33878	77,509375	72,09706	68,90861	80,013625	83,917895
ID471051	HNRH1,TIF1B	122,213835	134,62639	125,686185	144,152425	123,81122	128,862425	101,77396	92,81809	89,24436	35,08008	79,917055	84,364065
ID457	HNRL2	89,44624	91,45993	90,83303	112,493875	130,145175	135,259135	83,03438	47,274985	48,72722	33,89547	59,201965	61,39204
ID493	HNRL2	88,828005	85,487245	89,902445	103,87505	84,56136	68,074625	44,0409	15,271045	28,25315	41,24255	64,166915	62,667705
ID441	HNRL2	108,646335	107,68422	104,046305	141,68642	148,5574	139,66163	83,45177	47,32806	53,306735	49,8488	71,337975	67,488605
ID458	HNRL2	98,60034	96,936265	93,248825	121,839605	126,55021	127,6677	78,75278	45,08067	50,526775	41,4018	64,0626	64,21495
ID1203	HNRPC	86,890715	91,10909	94,772765	121,219365	92,34883	122,356345	39,730145	32,18253	38,632485	76,80908	65,516835	81,70471
ID2964442	HNRPC,STRAP	105,425965	111,84221	133,17342	130,768295	103,8956	70,21711	38,562105	17,183005	36,115845	79,06916	76,83554	90,610675
ID305540	HNRPC,STRAP	98,850885	87,61071	112,58245	93,975865	87,45691	51,440365	31,19687	11,50037	26,98523	111,28244	52,65709	65,404985
ID787	HNRPK	71,63349	74,73596	75,79276	105,43141	91,095285	87,64479	83,335785	97,01418	86,78947	126,31511	116,63789	100,972955
ID788	HNRPK	106,363365	118,735715	113,104085	125,25224	120,390495	133,492725	97,039455	76,773445	77,13336	100,78864	105,734605	98,36566
ID770	HNRPK	150,135115	95,891515	147,1807	58,20586	51,5457	49,98533	260,049045	310,480265	271,36329	221,09554	199,715905	142,180875
ID790	HNRPK	96,238185	108,307985	102,00321	121,55767	108,41824	98,309665	94,58733	76,96795	80,284765	112,91596	100,35124	93,75387
ID797	HNRPK,HSP7C	96,375335	105,274025	102,79675	111,623135	106,06876	124,05537	113,02581	83,73214	72,76748	90,15288	89,681195	81,31566
ID879	HNRPK,TBL1R,HMCS1,TBA3	84,902285	93,48237	86,002045	100,809605	103,020475	117,37388	48,70837	44,73838	58,46978	58,89311	56,56334	64,032255
ID447	HNRPU	74,85803	84,787985	76,76482	106,711375	106,11203	131,844675	62,01889	50,844965	48,972265	46,64981	66,740345	64,884805
ID391	HNRPU	103,401705	107,33846	108,232035	123,376135	129,226195	122,094625	90,115435	73,66978	84,302045	72,90708	69,765035	77,93713
ID464	HNRPU,HNRH1	74,595835	80,84048	72,04142	113,016285	124,50058	135,692615	52,49771	61,69535	50,64426	95,96883	105,895225	103,391635
ID446	HNRPU,PPCE	94,586575	96,358865	88,86922	118,519455	114,53813	129,953475	51,869995	23,888905	38,84938	39,45276	41,81824	51,247565
ID415	HNRPU,PPCE	105,356995	117,39996	105,219215	121,5833	121,1973	136,324845	73,03571	56,744405	58,782905	83,92116	50,84441	51,00426
ID486	HS105	105,805465	116,25947	107,61342	150,191575	128,97942	113,58565	60,62429	53,49747	56,734425	81,39342	86,50003	94,69202
ID485	HS105	97,100925	105,670875	99,896355	132,12283	117,2509	107,455415	58,03128	50,132975	52,434265	73,91053	83,02089	89,99386
ID477	HS105,GPIA1	101,95878	112,640365	105,825225	151,31687	129,54797	118,956375	67,836935	58,084195	59,083875	86,20448	83,405575	92,388165
ID573	HS90A	130,598265	151,38603	138,355455	170,489275	131,70239	102,912115	80,145075	63,636385	83,576105	104,61483	85,60073	95,505955
ID10923	HS90A	83,894015	93,26623	70,772775	138,012155	116,15283	104,667235	46,790185	29,574325	38,336135	79,05203	63,787485	77,347505
ID571	HS90A	146,594215	162,285625	157,069695	165,77699	164,43753	107,81048	79,28264	59,98812	73,382865	95,34795	93,26863	80,02381
ID3841	HS90A	34,945105	36,6603	47,63837	58,077875	57,944635	66,021735	32,17012	22,38615	30,748605	62,4076	56,78303	53,65962
ID596190	HS90A	12,23649	12,21398	20,44292	33,57864	33,74787	72,588515	18,029725	12,815975	17,184785	58,41809	42,826555	47,849495
ID553	HS90A	111,247765	104,74967	90,45744	87,449885	100,59974	82,953425	92,07409	82,984535	86,882215	76,51523	97,26508	92,755015
ID575	HS90A	100,71742	107,764475	93,801615	148,62892	135,059015	110,34707	63,1467	55,002585	63,559395	91,4066	83,286115	94,228485
ID21718	HS90A	113,40708	116,185445	112,71467	132,295925	113,7598	114,72872	86,473445	83,137125	81,22768	103,55931	99,771045	98,62234
ID628	HS90A	124,13819	98,01125	120,186715	74,64171	64,8243	80,84788	194,37371	204,763315	189,39077	176,44977	169,846375	132,485205
ID20414	HS90A	106,252805	85,73414	99,12609	95,426975	76,42019	88,9135	137,98746	139,2101	133,74298	137,97763	153,49622	135,10804
ID603	HS90A	85,59486	91,106195	95,020155	116,781935	109,372415	124,97468	99,111025	94,440955	102,192445	130,06831	121,048425	106,026875

Table S-1

ID[1]	Protein[2]	Median of normalized volumes[3]											
		co_0 min	co_30 min	co_60 min	co_24 h	co_48 h	co_72 h	PI_0 min	PI_30 min	PI_60 min	PI_24 h	PI_48 h	PI_72 h
ID3931	HS90A	95,00591	93,913145	88,724355	83,15191	74,86984	69,465475	48,96825	34,91927	44,71234	71,82701	83,6288	69,276115
ID623	HS90A	61,942225	66,063285	65,02128	111,06411	102,771475	119,851215	53,50494	50,47772	51,98419	67,68655	73,41094	72,54872
ID559	HS90A,DP13A,SRC8	127,479655	142,858675	127,439645	147,22616	147,964215	107,43242	77,02518	58,44093	69,661975	85,79541	78,81519	77,2794
ID574	HS90A,HS90B	147,834225	172,8703	152,226155	151,673435	143,18015	117,18765	62,99558	64,429965	70,83512	105,07607	90,835385	106,15672
ID2816455	HS90A,HS90B	118,10404	117,902445	121,06384	116,161435	122,240535	89,437475	77,961155	70,656295	80,937645	95,1014	93,309375	96,13312
ID627	HS90A,MRE11,FETA	106,587365	90,85989	106,294075	96,68341	78,19119	95,386675	144,098335	155,66808	153,99069	146,75998	150,544555	126,86897
ID4106	HS90B,HS90A	98,088415	92,660315	81,495185	82,82879	113,261415	84,209215	112,33307	114,93144	109,947665	80,9868	98,816385	108,180175
ID1672	HSB11	101,9225	111,314965	106,7508	125,42977	114,440435	100,43557	104,84333	90,719715	115,08602	101,32364	79,31445	77,06921
ID1703	HSB11	87,96661	100,666005	95,416845	108,974855	102,216265	96,15531	57,48677	67,711555	70,712475	89,9761	81,816465	87,609135
ID720	HSP71	91,740895	90,808095	90,164555	109,883285	113,02128	97,17259	104,33661	94,59872	95,64972	101,45382	108,574715	101,24557
ID723	HSP71,LMNB2,GRP75	79,392905	87,205455	86,60981	91,88415	92,319615	101,08123	98,94928	88,059575	90,17697	112,94543	99,52631	90,085635
ID717	HSP71,PDIA4	91,73067	65,578725	90,839945	57,44322	47,94298	60,476415	172,48265	168,200155	159,49359	196,47584	178,02231	146,99612
ID491	HSP74	117,387885	131,42829	117,19577	144,4244	134,95091	117,233995	60,07134	57,71747	61,25794	70,88544	84,8948	94,354475
ID490	HSP74	90,771295	101,63518	91,525075	116,785535	113,614755	117,754335	52,37114	49,40412	52,056805	66,12669	81,101055	90,147525
ID686	HSP7C	92,82365	95,532505	84,641885	99,23808	122,995975	92,919495	99,348815	86,733335	89,35226	69,54569	94,659865	100,87749
ID822	HSP7C,AINX	97,661575	106,73824	98,802635	121,679165	116,264655	108,46072	91,90038	74,22735	70,71322	81,2694	84,29945	94,814655
ID680	HSP7C,HSP71,LMNB2	82,8204	85,01673	79,53188	97,247	93,162515	92,50263	76,68602	64,892025	61,75188	75,42187	81,779855	78,397565
ID694	HSP7C,LMNB1	38,43073	41,77555	44,04876	79,20611	69,62381	91,807305	57,05817	44,937255	48,86529	93,77901	71,939555	68,176505
ID1420	HSPB1	90,82275	102,089355	103,15526	97,600895	89,284495	96,73678	83,917425	93,893135	102,95629	72,08921	86,16521	76,8637
ID1455	HSPB1	94,72076	95,783005	132,876265	92,23018	84,44504	69,27203	84,244095	182,63925	119,999255	67,0233	81,16726	84,841955
ID1453	HSPB1,ECHM	93,314105	103,741455	96,96968	127,86187	114,96128	115,372435	68,68163	56,393815	81,247985	6,83023	92,77232	88,54212
ID1492	HSPB1,PIMT	112,735535	124,75466	119,88994	130,042445	128,20654	139,78589	74,81586	71,755155	79,26768	0	99,30635	102,52069
ID5403	HYOU1	104,933805	109,79053	100,441805	128,196315	152,12458	129,49055	62,808955	44,81711	54,219235	51,55463	87,07681	97,178085
ID376	HYOU1	78,692405	85,653525	84,676535	79,434045	81,43829	95,41947	82,843155	83,361105	87,89132	151,59645	88,00485	82,82729
ID5526	HYOU1	125,7966	132,156595	115,974065	153,68373	175,256665	142,99983	65,832945	48,81122	60,526875	55,90508	92,16754	95,041985
ID364	HYOU1,OXRP	107,498435	118,59882	113,856445	100,6835	102,32023	107,93351	97,2608	106,16447	108,65854	139,36953	89,246685	84,58999
ID365	HYOU1,OXRP	122,65144	136,606545	128,02801	129,136635	124,25898	117,644545	101,95997	110,962125	111,558195	131,92476	88,866655	85,918275
ID475	ICAL	81,13175	88,34536	75,710305	115,03918	105,130375	118,265945	51,246025	41,973565	53,25658	44,2227	68,30676	74,53579
ID430	ICAL	118,86964	112,555385	104,101605	132,17739	127,111525	124,72643	84,2293	74,05765	79,850525	79,22365	93,687815	80,843295
ID427	ICAL	90,356435	92,703825	84,24817	111,48376	113,28335	119,219995	64,78627	57,74562	67,53671	64,65539	81,909315	76,82268
ID126195	ICAL	82,661855	87,257905	78,89539	97,618375	102,540295	116,56264	54,771555	51,334475	61,093735	64,0685	73,648785	71,981055
ID428	ICAL,DREB	94,20429	98,859605	86,04793	125,64519	111,98324	112,14956	63,647	50,659825	58,37536	65,6613	75,48431	78,057045
ID1174	IDH3A	94,31733	106,61145	92,286985	104,256595	82,825515	99,24972	66,918565	56,534225	62,070545	63,33772	77,00193	76,61631
ID1458	IDI1,PSB10,EXOS4	113,26455	124,50535	125,860455	113,50116	133,42578	136,298865	66,623215	74,60969	75,955545	83,60937	83,1818	80,91875
ID1656	IF1AX	90,196915	94,327035	89,096555	123,1911	111,416655	106,815595	72,03818	72,75479	78,766555	99,25431	95,33334	92,53559
ID3591281	IF1AX,SORCN	94,05784	102,02258	100,8249	134,331325	165,874425	182,521075	81,98823	85,95383	89,279395	46,87414	146,71883	115,60856
ID1196	IF2A	114,800555	125,5537	123,5164	142,621245	125,47062	122,097485	79,438875	72,8355	79,088445	83,12673	84,7699	91,28181
ID964	IF2B,ARP3,QCR1,PDIA3	99,45949	102,164185	95,83532	97,324985	105,74849	103,138685	98,608305	84,020515	85,99269	60,78503	65,90197	64,27305
ID1240	IF31,ANXA2,HDGF,TPM2	112,258575	121,853625	118,93118	109,93777	99,372405	104,77595	65,589615	66,17816	70,22354	81,21574	77,835675	75,55322
ID1185	IF32,NPM	90,148945	98,551025	91,383845	112,65182	95,680255	90,77182	90,724125	72,685425	73,077985	89,1695	80,654325	88,282805
ID1019	IF4A1,ACTG,HNRPF,NUDC	114,82808	120,09022	125,996965	131,570115	113,36608	92,45218	73,02741	60,04715	74,79233	113,64645	91,98746	86,02946
ID990	IF4A1,HNRPF,ACTG	131,13492	144,9141	144,8117	136,184055	131,43918	122,018655	117,848925	98,79915	101,504385	101,69725	91,82451	84,41809
ID13655	IF4A1,NDRG3,RBBP7,PDIA6,VIME	53,970295	59,6735	60,00808	92,348315	96,52328	123,29103	66,75617	60,025965	73,363395	67,16409	66,967105	71,889075
ID617	IF4B,NEBU	90,349495	96,301365	83,112385	128,707615	111,773775	103,52297	57,92254	50,27002	54,477025	88,84544	79,7504	87,240425
ID1697	IF5A1	125,57819	146,489115	139,50991	138,87106	124,756575	114,183975	66,63088	76,86786	79,98823	74,94364	76,364735	90,134065
ID1465	IF6	96,467605	106,321245	95,37545	119,655005	101,297625	94,115245	69,30174	68,68271	77,717925	83,0792	84,810395	96,055815
ID1139	ILEU,SCMC1,ROAA	83,392185	89,81473	92,7421	93,564795	87,37495	105,008575	76,462895	57,802685	58,74636	5,87952	68,645815	69,017205
ID3406	IMA7	96,587685	77,91618	97,533905	94,26415	92,54253	90,8098	107,433025	140,32007	110,002445	131,18498	107,957165	97,41211
ID577	IMMT,PLOD3,HS90B	121,475975	139,815665	136,578355	127,301095	134,033915	129,59463	116,448085	96,204505	119,38282	76,2547	92,47863	89,24167
ID630	IMMT,PLOD3,HS90B	102,77031	109,848325	111,11025	108,66548	108,50564	102,405015	115,18579	94,920475	117,273365	120,38061	86,134395	83,40469
ID631	IMMT,PLOD3,THOP1	108,12224	111,022605	113,9557	113,180675	120,158985	121,754005	124,95714	100,72033	122,07194	84,60941	86,644135	84,61247
ID1300	IPYR	87,844495	98,36225	89,5496	96,058825	94,7792	109,719145	55,093885	61,73592	70,24407	71,18552	77,038925	79,525625
ID1291	IPYR,GBB1,GBB2	100,5677	111,96815	100,129775	123,49058	111,224865	105,34823	56,09966	63,030795	72,317685	87,38117	77,57249	84,347965
ID195754	ITA3	104,98391	106,416625	105,47851	105,272145	122,2793	119,89359	90,449285	86,25528	87,861965	146,92881	90,3209	78,291055
ID393	ITA3	95,27765	101,54928	100,76714	108,8761	118,521855	128,336745	89,459335	77,87812	83,849485	102,88587	75,698945	77,675315
ID408	ITA3	111,592935	114,188295	113,47922	114,55497	121,915565	109,672645	99,45385	88,754445	92,530485	138,73888	97,16046	84,353605
ID981	K1C17	92,822125	100,18997	100,645075	128,91711	101,51616	96,99223	95,46175	91,544425	85,04494	90,56365	63,458235	71,332155

Table S-1

ID)1	Protein)2	Median of normalized volumes)3											
		co_0 min	co_30 min	co_60 min	co_24 h	co_48 h	co_72 h	PI_0 min	PI_30 min	PI_60 min	PI_24 h	PI_48 h	PI_72 h
ID927	K1C17,ATPB,PDIA6	100,916795	113,499225	105,92225	101,650385	97,506635	104,230525	142,165805	141,75504	159,196665	127,68598	90,40603	83,857635
ID986	K1C18,ACTB,NUP43	116,574	117,4766	118,74429	112,12773	115,11042	106,63233	164,099215	143,409985	144,96664	80,80191	87,08786	81,149675
ID1086	K1C18,ACTB,NUP43	107,39239	109,34952	104,55523	114,58219	108,540025	112,60658	119,05607	111,35651	110,82865	75,78423	74,47764	80,792675
ID417	K1C9	164,60623	107,54659	150,232325	72,883955	63,247785	73,83169	219,81848	235,73018	228,39343	221,54458	201,85055	114,57802
ID892	K2C8	63,454985	67,277035	69,379575	89,4619	94,420455	112,46449	105,376175	100,885655	101,86584	85,36767	83,319925	82,96057
ID900	K2C8	107,49957	106,04761	111,44065	104,041845	100,57192	99,68771	114,402355	99,83268	107,78299	87,94113	87,7491	86,11384
ID899	K2C8	48,56458	46,926475	52,96113	85,53226	85,615055	95,23819	78,663415	72,72335	78,144055	88,66947	86,666705	87,54794
ID913	K2C8,PSMD5	81,4663	80,52095	84,63503	111,181235	92,966555	82,68716	88,122695	75,86764	86,30302	93,77037	101,806085	99,938565
ID884	K2C8,UAP56,IF2B,GSHB	115,47763	116,7006	111,89801	110,091835	117,093035	107,8632	139,152875	115,43405	114,55504	81,33877	93,672635	86,48514
ID989	KAP0,TXND5	106,28601	120,52898	111,58428	106,89814	109,8653	112,75433	87,056825	87,83854	92,44173	157,52055	91,071245	89,40862
ID959	KAP0,TXND5	190,280225	219,773175	242,779945	143,48342	140,191785	137,24832	91,627845	69,197815	93,91855	78,72862	75,17587	71,935755
ID3670	KAP2,IF4A1	72,43548	82,36095	73,03805	141,543565	145,900115	132,79412	76,284215	68,52028	72,238215	70,41165	83,33537	87,77666
ID556498	KBP	34,23585	34,26936	40,758465	85,07427	63,55712	158,587995	56,855275	38,580105	42,135005	102,47817	70,450505	66,82817
ID207376	KCRB	126,959385	146,883025	162,48926	134,855525	115,12609	135,714415	124,71723	111,10026	95,782795	96,95912	67,3643	50,77126
ID3760270	KU86	119,62602	121,50887	121,54567	141,421155	131,283085	118,861545	82,733305	73,637365	76,618035	71,54231	88,33985	80,209215
ID629	KU86,HS90A	113,35748	115,778805	117,61118	126,20227	136,661895	124,61587	82,746765	76,949595	77,58779	75,12675	85,44791	77,10479
ID618	KU86,HS90A	83,064715	88,8023	87,92106	117,796105	125,327175	125,518345	60,811215	50,342545	53,45534	64,47075	77,644125	70,768345
ID1962730	LEG1	111,411355	127,35051	121,050215	136,40812	145,158345	127,826155	81,647245	79,42357	81,915745	76,94335	93,779035	143,15133
ID10509	LG3BP	75,07161	79,59437	71,30266	71,21765	73,970875	83,328055	69,52438	68,73782	72,41708	122,15319	96,077965	84,047895
ID777	LKHA4	114,081935	111,8335	133,659385	112,029695	109,98277	115,471505	63,65709	74,27855	71,718945	96,8233	90,044925	80,763545
ID765	LMNA	74,637825	80,77912	84,16397	130,100125	121,17434	164,284475	121,193275	111,21289	117,899765	46,71914	80,54018	80,472445
ID490428	LMNA,AGM1	72,75195	73,675015	74,95047	121,539965	126,35107	115,195395	111,662615	97,834095	99,50787	10,82725	102,33386	104,96687
ID709	LMNB1	100,045435	87,92174	108,11691	117,16327	105,2859	115,74042	110,28562	99,050155	105,92859	86,0929	75,699795	63,132845
ID712	LMNB1	90,314005	108,165305	90,83236	97,42072	108,52997	146,310635	127,448165	128,0674	121,04468	63,57538	84,505225	68,45556
ID708	LMNB1	84,368835	102,80584	80,974155	93,292345	122,336355	147,712825	141,546215	128,026815	139,40798	79,63217	84,668595	67,24641
ID537545	LMNB1,TKT	66,298595	81,7659	55,797325	89,978975	110,15841	162,78005	139,70436	91,78429	135,395465	76,2726	75,34978	61,424415
ID395	LPPRC	113,24041	120,073235	119,933365	138,41757	138,326715	130,74728	86,664515	75,23873	82,176585	99,55126	75,84155	79,92071
ID1308	LZTL1,SFRS1,SPEE	109,796545	118,521505	112,012725	136,354325	106,280195	101,8972	51,37017	59,558525	62,543365	68,75305	65,362785	70,606305
ID1022	M6PBP,IF35,HNRPF	97,971305	103,78247	106,951335	114,930715	109,069535	106,37448	70,4702	76,47621	80,338155	84,75392	76,875125	81,27927
ID204237	M6PBP,IF35,HNRPF	101,75742	116,198855	117,00938	138,304345	130,878535	118,2404	96,674515	96,48265	95,461405	151,44743	106,43579	112,34828
ID691	MAOX,SWP70,PDIA3	98,192955	92,601085	88,19165	78,424095	67,507345	72,400775	113,8348	97,831575	100,891345	89,2879	133,036205	133,534195
ID44539	MARCS	104,58545	85,799995	105,316545	107,07222	122,638965	172,34661	160,65049	161,2689	166,99454	159,61559	99,31577	199,43289
ID1355	MARE1,EFHD2	107,408675	121,82158	117,40969	124,861425	112,38746	106,274915	65,737195	67,67955	81,198895	94,53999	83,24865	84,657635
ID1360	MARE1,TBCB,ANXA5	90,787785	101,309355	97,368855	100,30194	96,19401	98,240415	59,46612	61,35071	70,643265	77,65007	72,84352	73,913625
ID409	MATR3	104,321075	115,726045	108,062735	125,598945	123,287965	141,95459	82,467955	74,2687	75,96514	113,78984	77,625785	73,63248
ID3316	MCM6,2AAA	133,278485	84,41908	129,07864	52,586285	51,209535	50,48305	234,13061	270,171245	248,70943	202,49276	181,54262	134,66513
ID585	MD1L1,GELS	107,212455	124,91023	117,814535	146,57801	145,51941	136,268315	80,311505	79,0294	78,95004	56,88842	83,718295	84,20695
ID663	MEPD,THOP1	93,18473	111,650335	91,729085	151,06737	165,99488	140,43486	59,157925	68,026255	62,651155	79,37246	90,67455	94,291215
ID2669	MLRM	93,31358	103,02277	99,20782	116,209905	106,180285	99,606505	83,225895	98,483985	92,887735	71,03045	67,107875	76,687285
ID1647	MLRM	102,1496	119,096375	119,273745	124,638365	110,677145	110,22416	97,40734	127,27327	108,47966	65,18016	59,78323	63,76336
ID1953484	MLRN	145,79776	143,79637	166,77627	262,157175	191,160485	158,99619	155,289205	197,226595	176,922915	127,70502	87,18592	98,791675
ID72360	MLRN	128,137075	135,350025	155,94088	145,92414	137,305615	123,77748	82,139625	120,681405	120,046975	95,93662	101,581505	107,4052
ID1041	MP2K2	111,340055	123,36021	122,3375	120,513595	126,07141	125,23612	81,608475	77,785795	90,10729	3,36545	82,256225	84,4028
ID1936868	MRLC2	125,753115	137,66189	134,16837	700,32188	229,910975	2543,86745	163,13195	126,340075	127,57231	52,20932	65,454625	115,706245
ID1706	MRLC2,MLRM	99,327525	114,560545	112,27744	140,56703	127,792305	137,91366	147,65649	127,59733	136,65432	139,83278	117,134055	116,57796
ID638	MSH2	108,798095	112,94434	107,640695	117,84739	110,66633	120,52038	93,49218	96,40357	94,648865	103,86105	112,730165	96,073975
ID508	MSH2,GANAB	111,148445	108,82986	110,66079	137,165195	147,46336	142,67547	58,659815	49,88638	55,93561	67,04125	78,418375	74,46453
ID1630	MTND,SSRD	103,138225	117,090395	92,936195	134,536	126,647215	125,20993	63,757415	79,300695	90,71898	116,12278	96,71208	87,830535
ID540509	MVP	108,028655	107,79714	109,786145	132,79784	141,0097	131,16176	81,875325	78,414595	75,031055	67,99376	88,786665	82,745725
ID487	MVP	86,30209	90,9824	90,970535	111,204475	120,626065	129,34288	57,333375	52,34758	47,90183	55,28965	70,961635	79,540625
ID460	MVP,SYAC,UBA1	109,95449	113,979745	112,70476	129,20204	138,5017	137,188295	51,927635	49,454765	53,39091	50,23711	72,46346	80,69045
ID7894	MVP,TIF1B,MVP	81,89983	87,682655	88,39351	109,671245	114,30139	130,018275	60,040435	54,22059	60,399955	58,58265	77,34056	82,916795
ID197182	MVP,UBA1	100,22145	104,57971	104,657585	113,274935	127,49106	135,666415	63,380285	56,16358	55,95264	52,03997	75,11669	79,41375
ID17545	MX1	121,653295	98,489995	113,63578	84,427715	116,116445	189,11618	121,7989	153,34368	140,545845	110,43978	150,45184	102,260835
ID379418	MYG1,SHLB1	104,582965	125,98932	107,014815	144,532225	128,49453	131,75325	48,52326	36,273975	50,37527	55,75707	75,38283	82,17916
ID31063	MYH13,SEPT15,ACTY,OAT	98,423835	108,665065	109,57591	123,432395	130,645105	133,42723	53,847765	60,536565	66,323445	0	73,45755	80,584805
ID1755	MYL6	96,386965	110,12746	100,13271	121,23284	112,26287	114,54521	93,00502	96,466855	103,599585	87,94402	71,331355	74,480285

Table S-1

		Median of normalized volumes[3]											
ID[1]	Protein[2]	co_0 min	co_30 min	co_60 min	co_24 h	co_48 h	co_72 h	PI_0 min	PI_30 min	PI_60 min	PI_24 h	PI_48 h	PI_72 h
ID1740	MYL6,RLA2	101,275135	114,041695	104,16645	123,387905	111,876525	106,66996	102,23783	94,38348	103,24265	110,42412	87,357555	90,68643
ID1258	NACA	108,153705	114,720635	116,264565	114,27696	106,59744	96,878	92,39461	79,148995	86,199595	135,563	98,867145	105,16279
ID1254	NACA,ANXA2	110,531495	125,410835	112,81694	119,98913	105,965725	88,45767	103,182585	85,29024	99,16714	139,75825	103,52944	105,04718
ID1205	NAGK	95,383125	105,444345	98,81304	127,262605	107,091335	96,402385	105,20668	93,88548	85,70793	81,42103	84,32485	87,382005
ID405	NASP	87,301475	96,411725	85,465645	105,626725	114,42036	143,412605	68,339865	51,3219	59,412095	69,07621	86,15047	66,788105
ID1645	NDKA,BT3L4	98,233775	105,30684	103,70695	113,79399	121,46559	121,370815	83,02861	89,2633	77,93022	96,55462	98,392145	98,23088
ID1632	NDKA,COMD1,SODC	111,395555	118,754355	115,64283	132,200385	113,370335	108,49177	62,15397	70,22153	77,19942	104,51204	88,79843	99,53977
ID659	NDUS1	109,80471	118,37019	104,993635	121,114965	138,28894	141,91276	125,72456	123,832025	127,00302	68,57906	77,726515	66,980565
ID1494	NDUS3,HSPB1,PSB4	116,900165	126,0295	129,98588	140,91627	126,802215	121,265245	96,120015	86,638785	91,511825	103,96412	102,19205	102,18758
ID1524	NDUV2	110,165685	124,493725	117,07831	103,402925	111,81172	111,5179	118,40458	123,520075	133,166505	112,14942	90,474375	81,764615
ID20028	NEBU	103,202395	100,40118	100,99458	133,683785	124,33429	111,907815	83,992845	81,304045	78,141735	94,17094	108,676475	101,523735
ID1432	NNMT	124,986635	126,392925	135,924125	118,519145	124,04656	111,084255	81,523245	91,449505	96,48216	81,02414	77,67003	73,617755
ID926	NP1L1	88,668935	102,3272	93,880775	104,025635	98,436195	86,03111	48,2066	40,49217	55,04854	74,18204	63,60915	70,52472
ID845	NP1L1	125,30821	96,609325	105,626375	61,74539	62,1141	65,630385	202,678215	214,39957	207,41278	173,58807	160,876715	117,163295
ID929	NP1L1,SYAP1	80,279175	94,87891	89,07973	103,77243	87,89002	81,068805	41,303125	35,640165	50,86771	73,83915	65,35227	75,804195
ID2404447	NP1L4	128,573145	96,486605	116,752195	73,15063	69,130185	61,31076	190,99681	210,55482	200,071615	154,38892	159,625835	122,61703
ID846	NP1L4	141,25743	98,36416	116,26769	65,192585	59,887745	59,37413	228,077355	240,96778	235,72471	184,22187	172,824195	128,55674
ID842	NP1L4	136,431835	93,49081	111,072655	61,379925	55,95636	54,147875	238,85271	255,886515	249,29743	186,72666	175,99665	130,26913
ID1209	NPM	97,51595	105,42086	99,985445	110,710765	113,43727	90,425315	115,43896	95,49721	86,55524	98,65601	94,1613	108,627905
ID1200	NPM	93,791825	97,459275	93,28613	108,93663	104,266265	86,66284	84,563105	67,987075	63,490125	93,37123	89,885465	98,43657
ID1224	NPM	92,895745	126,82514	83,775215	143,123	93,94151	59,1223	73,011155	48,259065	73,0794	97,09744	121,25841	124,0548
ID1225	NPM,EF1D	88,18576	87,924315	101,59805	134,091465	96,205255	66,709945	114,294875	84,38836	102,444865	122,17261	91,63221	106,755445
ID50273	NSAP,CALR	69,827135	64,900695	65,528845	82,599285	83,591505	87,435995	186,27014	178,51856	188,296955	104,90164	113,174565	89,519145
ID3227	NUCB1,VIME	123,967605	100,82282	127,7094	91,29247	90,63104	86,736145	193,59683	254,357995	207,841465	193,90176	159,18014	130,508215
ID520	NUCL	86,314545	84,894615	85,16088	118,001505	123,994405	137,204405	55,395805	40,138735	48,574835	59,05825	75,655	72,14311
ID1364	NUDT5,ANXA5,SFRS2	88,125155	97,53695	87,85583	113,730585	116,15961	112,63986	63,639475	60,406385	70,48728	94,84711	87,90766	91,071215
ID1776	OAT	109,769525	121,004375	116,40533	136,2638	113,869205	93,64105	80,7897	93,770815	95,66757	121,61389	86,10186	88,66302
ID970	ODO2	82,16804	92,407425	89,76152	137,653625	108,042095	132,67247	49,06888	51,2309	59,51695	48,87313	59,27437	69,58617
ID1255	ODPB	103,362795	118,11586	113,776855	118,88195	119,313605	116,547765	94,65876	90,357125	91,00923	79,44157	77,010965	76,502115
ID1282	ODPB,CSN6,GBB2,GBB1	114,65877	126,199715	119,954	147,465725	159,52693	156,53016	111,052715	118,0466	108,91506	59,02264	76,60582	78,73447
ID1321	OTUB1	123,90914	137,34954	142,50579	112,60729	120,67965	94,90845	75,03832	73,52663	85,306805	97,94251	94,636945	88,601305
ID1312	OTUB1	96,668125	98,15916	102,58626	123,464905	130,81535	116,763785	54,008625	46,729415	64,78598	73,13809	82,800025	85,811065
ID319168	OXRP	144,960715	162,7822	148,537775	164,82593	143,73832	134,994525	119,705415	115,17928	126,399985	153,77428	114,03666	97,864285
ID2818369	P3H1	131,35512	137,209385	143,3885	134,950935	130,828115	104,582925	68,56556	56,59821	70,173025	102,64138	91,394085	92,11789
ID4295	P3H1	134,702705	146,64593	145,352545	129,55633	125,62167	116,321605	87,64216	71,59371	84,56	127,19621	85,329855	85,34377
ID564	P3H2,KU86,HS90A	109,887175	110,896305	122,61177	130,174895	126,27058	124,993795	110,627995	95,285225	118,66564	100,39144	92,027265	93,341705
ID558	P3H3	89,999765	106,32875	90,528415	115,545045	127,95848	139,911015	94,15387	83,616895	89,00055	19,07457	79,596995	84,567365
ID813	P4HA1,TCPA,PDIA3,CPNE2	125,62708	127,15037	132,924855	125,798515	110,38066	109,616685	160,366465	193,925085	179,8896	153,11399	135,50004	100,633455
ID812	P4HA2,CPNE1	97,04151	108,13129	102,15902	130,894655	119,88048	117,955845	96,10828	94,363625	107,26528	116,10937	90,586295	89,585415
ID1412	PA1B2,PSME1,DDAH2	115,109985	124,54593	122,53049	132,427435	133,458305	130,678155	90,599915	83,260535	103,25084	96,16658	96,13162	99,87751
ID1032	PA2G4,SAHH,ILEU	120,719335	124,012745	124,779495	142,444575	139,96111	130,71813	52,843395	51,24035	65,282805	0,033635	75,802565	87,947015
ID17391	PAK2,PDIA3	98,323055	99,964975	101,654135	105,362865	105,173045	94,81304	92,83751	95,595365	95,7223	93,9124	97,51677	95,88022
ID399832	PAK2,PDIA3	104,125125	107,599825	112,022135	103,96024	98,481235	104,992135	113,241575	123,03186	137,302165	104,87083	98,46351	80,740045
ID1511	PARK7	109,71956	121,44594	115,410455	126,46992	123,144215	124,799205	62,63613	62,01608	70,174465	62,950205	89,342365	89,596745
ID1304	PCNA	105,54681	111,55669	109,888315	125,90615	141,217495	137,888155	69,270035	58,766135	75,4695	85,65131	91,27522	78,982645
ID1328	PCNA,CX026,AN32E	72,27044	73,167855	72,5756	98,275275	114,5453	114,79671	56,72688	44,596565	60,082965	92,75055	92,455015	82,623415
ID99319	PDIA1	116,819235	124,31853	117,35995	114,580575	112,332845	109,54823	111,83056	117,77718	115,53436	107,62788	98,426245	90,027655
ID824	PDIA1	70,154905	73,79069	73,689105	75,6022	83,75515	89,764445	87,177305	88,790195	94,07053	142,71697	87,041065	80,37446
ID230824	PDIA1	90,13302	91,382335	127,79347	90,00562	123,36711	123,83671	117,432135	138,83287	152,35692	103,6119	83,211745	90,96413
ID820	PDIA1	95,878815	98,443725	94,40794	100,54184	112,627945	101,94401	104,44452	97,567935	99,87562	113,26881	92,694895	80,312945
ID2239	PDIA1	39,294685	40,88196	41,606585	59,70128	60,26962	70,42288	63,22694	62,082935	66,71734	183,04104	85,81699	81,54254
ID451640	PDIA3	133,1189	94,897305	120,07308	98,575005	102,451975	131,684325	173,82686	134,38324	133,099545	156,77576	101,71275	95,542965
ID854	PDIA3,HNRPF,VATB2	74,388145	80,87528	75,731335	83,39727	88,76929	103,40568	90,630875	84,1349	93,80482	129,76688	83,39729	81,220285
ID17374	PDIA3,PEPD,TBA1A	122,85605	102,58295	123,045795	113,751565	118,37146	118,00765	154,696065	178,577995	159,34358	134,21085	114,16164	104,763945
ID836	PDIA3,TBA1B	131,56153	99,66647	133,26297	69,006235	73,124045	81,53773	232,58061	271,884915	226,435225	145,36455	126,86215	104,57924
ID865	PDIA3,TBA1B	124,30701	133,454025	126,00208	107,05642	109,52916	116,28257	126,99228	136,11122	132,378945	102,48049	76,744685	78,818355
ID864	PDIA3,VATB2	59,46478	63,608875	63,17621	91,31856	90,841305	111,149555	77,41689	62,64748	72,685395	128,59361	82,353575	78,4674

Table S-1

ID[1]	Protein[2]	Median of normalized volumes[3]											
		co_0 min	co_30 min	co_60 min	co_24 h	co_48 h	co_72 h	PI_0 min	PI_30 min	PI_60 min	PI_24 h	PI_48 h	PI_72 h
ID660	PDIA4	127,5737	102,619925	119,847555	71,495595	50,86444	89,349735	160,54442	157,860255	161,95198	122,14481	146,926555	133,140465
ID370380	PDIA4	81,825065	78,87163	91,003555	88,776805	81,23932	74,98199	120,187095	134,088435	129,86416	101,79991	114,483175	111,634795
ID22159	PDIA4,HSP71,ANXA6,TF65,GRP75	95,29908	83,456825	92,913445	71,463575	57,72285	69,582685	128,770165	133,548695	132,64408	170,31327	172,152395	142,214755
ID982	PDIA6	63,761615	70,711895	69,22459	77,501125	80,36838	88,51096	88,433955	86,652045	99,881655	125,86458	80,951885	86,13748
ID176254	PDIA6,F10A1,PABP2,PRS6B	95,607005	106,632275	95,910525	101,85588	102,45521	103,05947	71,51298	67,164905	73,63045	82,77913	78,62029	79,368625
ID1784	PEA15	105,943585	118,585135	101,64145	124,44321	124,28236	126,399255	35,4076	36,12181	59,8695	72,23945	72,59083	74,691475
ID450085	PEPD,PDIA3,TBA1B	96,342085	102,773985	95,22645	118,89107	109,418215	135,39376	104,0611	92,517505	99,93916	124,63893	119,056235	110,370085
ID810	PEPD,PDIA3,TBA1B	101,830135	102,046225	92,245225	96,99557	108,124385	97,560555	102,785335	95,3944	93,726285	110,95884	90,286355	88,07965
ID1522	PFD3	111,87283	128,11715	118,49048	137,908455	126,595655	115,437325	75,132175	75,642865	78,18218	77,772675	93,710685	89,3352
ID1746	PFD5	113,631105	119,116385	118,23592	131,428415	118,581605	108,97632	86,61102	84,790265	87,597555	121,99891	82,68294	77,413575
ID1468	PHB,HSPB1	83,140805	85,30102	85,14586	107,351535	101,941255	111,62085	111,448325	121,359655	117,956235	86,7754	71,64494	64,841265
ID16655	PHP14	101,25266	116,689285	104,227255	105,43377	186,8931	153,01901	63,85154	74,767685	79,52881	54,94822	121,137135	271,12135
ID25674	PLOD3	91,570605	95,8598	96,91669	89,728695	90,76147	111,489835	102,31248	92,376155	106,252805	121,44448	74,60981	81,72364
ID371745	PLST,AMPM2,CPNE1,COR1B	122,886695	116,88176	128,409955	108,490905	99,964245	126,582195	84,62894	100,46576	93,97371	95,65982	124,027465	105,680125
ID798	PNCB,FKBP4,TCPE,TCPQ,HDAC2	110,82288	117,665355	113,275115	137,404325	125,940875	115,305745	71,55722	65,619035	71,6885	92,163	88,43521	93,778725
ID1443	PNPO	101,68788	110,798545	105,02612	116,29279	107,561865	109,605775	66,19815	77,75611	84,25103	18,437955	81,315765	85,19774
ID534785	PP1A	106,924355	116,794685	116,681045	114,37988	105,16261	111,23662	59,74885	63,197685	69,45707	78,27798	81,28752	86,48426
ID1272	PP1A	66,265805	80,689805	57,655275	116,79723	85,62816	97,064965	59,78323	69,454425	75,07676	27,84084	70,81786	71,494505
ID1331	PP1A,DHC3	118,02139	133,45356	136,287085	102,06771	107,577345	111,02663	80,644675	116,91253	95,620745	68,78877	85,597655	84,402385
ID1186	PP1B,NAGK	111,039105	124,1509	120,07581	129,52815	106,505025	103,19859	102,78647	109,24179	94,07157	95,97127	88,970205	98,31138
ID1030	PP1R7,IF4A1	126,709325	140,328585	136,165815	138,136095	150,71082	146,900185	56,60696	58,514665	62,261735	73,76849	84,132425	84,34823
ID1252	PP2AA,ACTG,ANXA2	96,98864	105,342205	111,598005	114,14415	99,94927	109,857315	87,367735	95,748435	95,029695	87,96512	87,75165	94,305325
ID1241	PP2AB,CT077	101,732535	109,914445	110,82194	126,72802	114,15067	108,50645	83,697105	74,55044	81,62649	92,95755	91,470365	94,03015
ID1316	PP4C,EF1D	109,250655	127,629075	104,67897	96,38084	97,443405	96,53332	83,36981	78,430795	79,74484	99,3726	79,512505	81,799925
ID2382844	PPM1G	106,79038	101,95479	108,897125	121,908615	127,888525	118,65015	66,23745	67,208305	69,983165	94,48362	80,71481	62,337185
ID1060	PPME1	120,37434	126,51582	135,299445	151,71643	136,07957	131,170525	92,00097	82,017065	91,519975	86,67675	80,925825	87,403435
ID844	PPP5,RHG01	107,41728	115,10957	119,76606	114,465585	103,354665	103,989475	72,818805	83,964295	82,29256	99,11488	82,768005	86,589505
ID1249	PPP6	100,4087	130,03249	117,98513	102,903005	99,02036	107,93433	67,1304	85,14055	80,498895	88,49611	88,451395	98,514115
ID1243	PPP6,LDHB,PDXK	120,429275	116,06084	133,9024	105,12008	107,043175	108,45274	57,71698	60,844165	68,28156	78,05838	73,68932	82,98301
ID1296	PPP6,PGP,CAZA2	125,06775	121,805395	139,57516	104,195015	105,089875	111,151865	60,07597	74,49097	72,30736	69,53055	60,199955	66,056865
ID2198480	PPT1	133,577465	150,53858	155,250715	289,995265	729,548225	379,316545	95,87643	79,34198	65,49324	0	0	1,957565
ID3102815	PR56A,TXND5	99,55876	116,8937	108,6874	117,300605	115,985625	128,39217	91,50126	83,061265	78,4633	109,58481	78,32334	77,63937
ID177384	PR56A,TXND5	126,06852	146,475475	150,693655	138,84095	133,57958	116,56032	77,94517	68,482595	68,189505	95,726	87,75522	84,71873
ID1541	PRDX2,GSTP1	105,863845	116,48139	109,39538	116,23451	119,65452	132,67535	39,159575	39,69429	38,013745	66,92868	77,894635	79,38837
ID1510	PRDX3	106,995185	123,206485	122,22205	124,6202	126,64329	133,516975	44,815985	40,86548	48,890525	0,92952	67,38631	74,05431
ID584346	PRDX3,HSPB1	109,69096	120,594015	117,400275	114,001235	119,11324	126,03524	38,596625	47,198395	58,62272	70,94781	85,27138	91,078035
ID1497	PRDX4	123,909655	134,50746	136,59139	123,197605	121,847575	125,7833	52,04898	82,946805	79,140225	116,49895	110,207985	119,71593
ID3056834	PRDX4	137,498445	148,632935	142,824045	101,095135	101,547985	55,222525	77,577945	107,81489	107,785555	148,0632	117,0544	118,957775
ID16864	PRDX4	61,821855	68,65892	61,45066	110,774575	92,639375	105,184615	62,64804	53,60487	67,02808	106,04587	86,3639	91,95951
ID1471	PRDX4	93,83494	108,5148	99,65398	108,89388	95,174535	88,88084	98,53782	120,72301	119,600605	129,33034	86,936285	81,921225
ID1802	PROF2,TR112	128,346875	143,6517	138,119775	147,315955	198,050345	150,458445	85,89075	87,264085	97,31836	87,62207	98,10084	127,53878
ID469498	PRS4,SYWC	110,73022	105,741405	120,123695	98,442745	98,06643	108,35831	148,019895	168,77284	162,95412	111,43591	91,114665	92,177695
ID1002	PRS6A	103,63856	116,416375	106,21356	125,22575	114,995385	110,64495	79,797885	68,51339	70,16351	99,69526	81,207135	81,191295
ID997	PRS7	109,6916	119,273495	116,010395	141,83456	124,018585	121,263735	84,15451	66,44102	76,463305	91,16458	83,743095	87,07761
ID172969	PSA3,TPD54	106,66479	115,199925	111,916265	125,562815	121,14112	123,2719	71,588645	68,220075	71,739115	83,59725	89,40346	100,91025
ID1476	PSA5	94,761985	99,361845	97,977995	111,77623	119,165025	118,768545	73,98328	67,63981	67,890495	93,70505	92,59164	101,79124
ID16938	PSB4	83,21554	93,38514	86,217055	143,634185	117,81884	117,961055	88,912085	85,27848	82,45416	180,11916	102,22785	99,269185
ID3058865	PSB6	123,45137	133,33376	125,961185	99,699495	109,431825	124,71103	112,968965	99,171485	98,37257	69,16042	77,09932	70,03763
ID1402	PSB7	120,83483	136,977925	134,3655	153,079335	128,240345	123,042555	91,184115	79,236685	87,448185	96,40822	96,456185	105,245095
ID1460	PSB7	103,65405	111,916445	115,87671	104,90591	103,32547	111,958385	114,45864	136,25803	126,93489	101,68675	88,90022	85,616405
ID1519	PSD10	110,97222	121,920625	117,36009	129,710955	125,478305	123,936765	72,9118	77,837175	86,798175	110,70106	95,232725	99,72005
ID1140	PSD13	121,08545	114,973585	142,05416	102,513085	108,747465	114,18186	77,976085	62,327215	66,09693	62,14334	63,636645	70,623815
ID1244	PSDE	116,09945	129,47189	124,61628	125,31013	121,787715	121,372725	92,777175	81,8346	87,945085	50,47915	87,587415	87,56264
ID566	PSMD2	89,034875	91,49321	94,12836	109,05688	96,395145	96,666355	53,577355	44,129235	52,56442	74,01722	68,841855	80,10025
ID276677	PSMD4	105,472485	163,67549	114,782925	178,08277	201,07812	142,005535	100,8158	82,76272	79,717305	62,5366	92,0593	107,510875
ID955	PSMD5,SH3G1	130,197995	138,020355	134,112735	161,66785	156,35356	144,12045	68,335015	81,103845	110,60133	83,37508	92,64086	83,26756
ID954	PSMD5,SH3G1	134,946115	123,124285	157,36733	130,795915	138,762	128,998885	81,93008	65,85993	90,83708	65,98606	80,944295	84,809185

Table S-1

ID)1	Protein)2	co_0 min	co_30 min	co_60 min	co_24 h	co_48 h	co_72 h	PI_0 min	PI_30 min	PI_60 min	PI_24 h	PI_48 h	PI_72 h
		Median of normalized volumes)3											
ID907	PSMD5,TXND5	82,69473	82,32863	92,03621	104,041405	101,43464	116,30184	92,26588	87,06506	89,171635	93,64137	84,364135	88,58941
ID1433	PSME1,6PGL,ERP29	99,233445	107,29279	104,865385	89,85611	87,95466	100,752215	91,59247	94,88568	97,60142	136,2215	83,90839	81,95661
ID1392	PSME2	103,89938	101,9394	116,082795	101,7192	122,62001	128,156265	56,57135	50,080195	60,597455	73,84066	77,39895	73,60516
ID3313934	PSME2,1433E	118,107265	113,25819	127,45944	123,891635	120,37339	127,74425	74,65441	60,3621	75,57591	216,95407	141,39646	138,584975
ID1373	PSME2,1433E,PHB,1433Z	110,489895	115,286625	121,352575	97,33692	104,62131	113,67391	114,42304	111,813885	125,29877	177,32055	81,322805	80,61155
ID1409	PSME2,CLIC4	122,83746	124,73231	139,42729	136,560255	144,401065	143,015085	56,517685	62,968625	70,428985	85,3662	88,9922	97,801535
ID1396	PSME3,GSTO1	104,16194	123,888995	116,88715	114,33295	121,681865	124,937145	65,72509	81,868005	84,67264	85,67739	92,89434	100,917235
ID458579	PUR4	107,936225	117,66807	114,512385	152,8932	138,86892	131,65893	82,21557	64,408815	79,00715	71,8888	81,44725	83,48611
ID3435858	QCR1	145,420975	108,176235	124,70041	126,0504	152,89496	95,347375	87,38864	42,12074	69,771685	62,20935	1826,11282	132,539675
ID714	RANB3	148,589345	95,934475	152,867325	62,93775	64,273655	50,44118	252,21722	295,52942	283,92726	173,58004	169,928155	126,074265
ID1446	RANG,PSA3	103,649785	117,59076	103,68525	114,24166	110,50683	117,75525	59,539535	64,759415	72,8355	68,86046	64,63208	67,98964
ID932	RBBP4	95,767265	106,65236	96,308275	124,653805	118,67156	117,350545	113,10438	102,47742	102,76783	72,53909	81,67369	74,33916
ID1604	RBM8A	142,16532	169,91537	136,598975	127,65509	120,009385	114,5316	109,65879	82,951355	81,65969	56,64557	64,23401	27,904685
ID1586	RBM8A	99,097265	119,877035	106,235425	131,101615	127,193205	132,32744	101,07464	96,75225	91,022425	79,50646	93,57226	87,715765
ID2662678	RCN1	78,55588	93,24772	81,93276	71,889365	80,687995	87,594505	90,977335	85,940245	101,97293	112,43859	84,21475	81,43941
ID280677	RCN1	78,870305	82,21975	77,66271	70,419955	80,397275	103,520755	89,174765	102,93082	100,99383	140,64662	91,66706	93,413525
ID2687300	RCN1	70,291485	76,96989	71,336005	50,634905	61,362905	88,72084	87,531655	88,70634	97,85226	110,25728	83,478875	88,254095
ID55507	RCN1	83,155705	92,00353	83,660775	93,2433	91,20634	109,390935	95,93322	93,144365	105,730055	110,64906	80,20923	78,783735
ID459	RCN1	79,99439	84,658605	78,713455	108,05688	100,121615	105,84377	51,268265	34,981155	44,41851	49,71831	70,29365	70,448345
ID1061	RCN1,ACTG	102,1393	120,155515	105,62752	93,820165	100,65686	98,806335	99,536795	93,88778	114,448285	113,69752	80,68619	79,076395
ID1127	RCN1,SPRC	106,20693	112,229	109,77198	105,17646	110,89347	129,624105	95,114125	112,861585	104,10035	123,67739	81,25273	77,970095
ID2680471	RCN1,SPRC	92,13064	103,725555	92,18002	84,221265	87,45377	111,21924	95,24782	103,0901	104,933425	130,74152	81,53274	86,717155
ID1825144	RCN2	105,18952	119,44726	107,52512	105,36874	101,75409	82,133925	129,035375	157,60411	131,59355	84,48335	78,90923	65,86483
ID935	RD23A	99,285	120,26754	108,471965	134,63193	170,60037	136,189935	49,69347	49,8043	62,934325	72,91175	80,6064	73,929695
ID3128108	RD23B	124,880385	69,743005	115,54939	57,87854	49,446775	52,70732	258,54656	311,78497	259,821865	194,45784	191,902815	142,5744
ID94683	RD23B	99,445755	103,067785	107,49125	103,85378	108,48195	128,41767	130,759355	156,88764	137,343295	136,86305	99,245345	85,77356
ID99730	RD23B,PDIA1	103,403635	103,34606	101,44057	101,056135	108,57143	101,878915	109,357985	103,170865	98,239305	105,80378	82,5591	70,109605
ID1367	RFA2	115,26994	122,099375	125,239825	105,470255	123,53351	128,7397	99,073475	98,420235	104,206225	0	102,863685	100,98432
ID1375	RFA2	117,16934	131,04903	130,38104	164,54349	153,57252	155,928945	78,449245	73,08301	85,40367	68,58281	75,161885	79,58611
ID1146	RFC2	131,51075	145,16747	152,012925	135,28749	131,975645	145,858465	81,40249	66,39416	84,559715	0,036235	98,85021	96,931225
ID851	RHG01	141,279365	104,214045	117,157545	71,957845	67,0879	69,775455	234,16479	239,130605	234,85226	181,24806	172,4	130,13306
ID922	RHG01,HNRH2	94,571275	102,906335	106,208895	127,983945	113,88663	121,662575	63,060965	53,880645	59,87421	12,84439	68,416435	74,91266
ID1005	RINI	123,95066	136,285025	126,45136	173,41188	173,933315	148,878005	40,772775	46,606265	59,91757	67,05789	85,76277	103,02331
ID1248	RLA0	106,350945	117,48712	118,89529	123,488875	110,89936	110,425195	94,3698	93,488915	94,297095	103,10273	78,332845	87,963265
ID1098	RSSA	132,905825	143,218705	151,347135	137,45149	119,38672	102,595775	119,65832	115,846995	109,53807	130,7853	96,035725	92,16642
ID1156	RSSA,SET	89,982795	101,671785	88,19172	85,407925	86,22599	99,719545	75,026735	71,98729	80,721185	74,04095	62,739025	58,33773
ID949	RUVB2,NEMO	101,406775	107,875565	110,163765	110,881035	105,673855	107,51892	89,364545	76,25924	85,85567	85,78328	82,36863	84,148175
ID357613	SAE1,ACTG,TMOD3	103,333345	106,59616	107,339505	112,4153	110,101715	105,46554	75,206645	60,05108	64,538825	74,87233	76,27892	74,65236
ID1277	SAE1,EF1D	93,79593	104,387305	94,864155	87,065635	84,35151	87,566875	83,064885	70,866985	73,31561	81,11109	70,22585	68,57242
ID1042	SAHH	88,39115	90,45175	87,79727	109,866185	110,985365	111,541835	46,046855	44,61768	56,79728	64,33193	72,96663	85,357115
ID3228910	SAKS1,SAE1	104,58526	105,28229	103,750055	105,5346	103,040155	96,301505	62,216355	59,88184	72,546305	107,71771	95,128065	104,372905
ID703	SCFD1	112,95461	106,648105	115,730585	104,935605	95,346605	94,45132	144,452205	151,320435	141,903625	332,15153	143,271315	115,84714
ID1093	SCMC1,ILEU	113,99539	125,953935	124,921575	140,88356	143,353865	142,812405	53,739555	49,95845	69,129455	0,08462	90,61496	89,090175
ID418384	SCMC1,TADBP,PPME1,ACTG	100,820095	107,31552	114,47639	87,63967	112,56961	115,142495	99,360355	85,18938	112,23633	76,04436	54,67262	62,937435
ID937	SCRN1	96,684085	109,835335	93,72714	128,88687	135,970935	136,89552	45,213665	44,52698	54,208635	50,53299	72,243875	83,32729
ID697	SDC10,AGM1,NDUS1,WDR72	110,50713	97,549265	99,03971	66,692285	59,8625	74,505115	157,370475	152,676235	141,250835	122,26538	153,92975	135,206625
ID1215	SEC13	115,98761	118,921295	120,60693	119,61696	103,81845	108,605965	104,218885	128,89128	117,24424	81,92575	77,652935	76,73474
ID921	SEPT8,HNRH1	103,34458	110,127325	100,981345	126,845605	135,29366	137,533455	82,850195	68,618535	67,39088	69,96492	73,275985	81,648715
ID1142	SET	92,681865	94,44646	100,51275	127,506515	113,23224	134,1815	67,53447	50,37757	61,34375	80,04426	98,29802	93,325685
ID1157	SET	91,001075	101,152555	87,10979	105,5324	98,534085	95,035435	56,75687	44,50149	57,613325	64,11875	60,40336	57,33314
ID90190	SET	53,800325	45,24246	73,62625	73,243945	87,51933	99,40002	24,228875	7,055915	21,826935	44,49076	75,14704	70,72299
ID279386	SET	97,517155	101,00069	106,192755	131,68405	122,038935	125,47781	73,731165	64,23634	70,48868	92,7133	97,494935	93,791225
ID1052	SET,ADRM1,MYL3	156,330105	179,4491	157,64854	113,75063	117,254505	109,341605	66,74961	88,63489	65,794795	61,88892	75,439265	77,73833
ID2906459	SET,HDGF	95,760165	115,494845	126,740535	113,681495	99,3109	96,761725	64,555545	71,740045	70,157055	46,91325	72,055535	68,766385
ID306795	SET,HDGF	109,508145	142,49929	124,75851	145,30197	177,111745	102,235415	34,32333	40,25473	40,01487	26,02775	82,343155	65,60388
ID92002	SET,SPSY,HSP7C,RSSA	170,98863	275,94127	207,232355	140,38776	176,28179	120,1309	44,10635	120,816445	68,04464	92,8231	118,58032	89,504575
ID3760549	SF3A3	108,074355	115,4459	116,005045	110,57355	112,62266	110,03992	107,931985	96,23625	75,858115	75,68843	73,252985	71,873795

Table S-1

ID)[1]	Protein)[2]	Median of normalized volumes)[3]											
		co_0 min	co_30 min	co_60 min	co_24 h	co_48 h	co_72 h	PI_0 min	PI_30 min	PI_60 min	PI_24 h	PI_48 h	PI_72 h
ID9421	SF3B2	104,00866	97,49692	103,0334	84,832695	104,40951	123,290995	84,082745	99,671755	96,89548	159,39478	103,666445	93,295085
ID354	SF3B2	87,997525	97,73544	97,64113	128,90794	118,956495	134,52382	56,77604	46,608455	45,71159	23,88642	69,292855	67,889055
ID355	SF3B2	101,707945	113,45567	111,854045	143,80814	131,4804	133,6813	64,67802	52,532145	53,3753	33,82847	70,377805	65,34395
ID2995227	SFRS1	84,08593	88,116885	88,33843	104,877195	128,18189	89,963265	83,945595	75,26504	80,10889	45,95296	86,86657	57,8391
ID1536	SFRS3	112,711435	117,55585	123,09675	123,53659	143,41232	130,545755	48,31606	55,72197	66,770565	91,60688	84,943685	86,861395
ID2287478	SFRS3	114,61784	132,61368	126,3033	152,367195	128,036525	113,41255	85,226125	90,295755	96,72478	39,360675	55,29947	53,2745
ID1565	SFRS3	109,023795	115,17442	117,84112	117,541405	136,88622	123,145795	112,986325	97,41383	86,64104	77,82344	81,612425	67,77637
ID1572	SFRS3	97,21524	115,36315	95,409085	136,498045	111,085585	113,62893	117,386245	86,6367	84,49697	4,63867	52,794415	35,30268
ID1267	SGTA,HDGF,ANXA2	114,682475	123,788925	124,954455	120,042045	113,5074	116,00765	64,88041	48,893335	61,306755	94,32573	97,87731	98,89685
ID972	SHLB2	100,89477	111,179645	101,30167	130,193775	139,37981	143,92905	63,82429	60,26081	71,346035	61,19357	78,80873	76,77294
ID408351	SHLB2,AL9A1	101,98137	117,491025	108,152635	132,61745	114,053295	144,20202	58,076795	69,43387	69,560325	82,47422	71,636235	83,29015
ID950	SHLB2,AL9A1	104,74609	123,107975	122,07331	138,3217	107,71413	97,15685	115,028515	118,49549	107,019695	79,37761	75,479165	88,382805
ID1628	SKP1	83,608905	96,5356	93,92058	90,72246	95,92987	101,443955	76,21926	75,524135	85,15592	85,55401	70,08186	58,547825
ID1305	SNAA,SFRS1	118,47806	123,669495	123,36385	132,96543	139,060845	122,81067	90,74157	93,025295	102,52833	77,51945	75,452525	74,20181
ID956	SNX6,HNRH1	105,46485	121,883465	109,485695	136,065405	134,031515	131,568315	62,251745	59,599115	65,42702	67,03359	81,44327	88,4429
ID30752	SODC	77,67357	78,00102	75,803055	144,32041	122,77806	115,986995	32,361365	30,3744	44,382475	79,13615	65,4886	65,4389
ID508072	SODC	103,738	116,5195	100,349145	131,24098	121,695765	127,465295	60,107275	71,384125	74,176945	60,75552	68,031945	61,57637
ID1651	SODC	85,36791	94,749065	83,02438	106,836875	99,22706	131,14779	57,373805	56,38549	71,09711	79,89983	83,38503	87,31184
ID1589	SORCN	104,3072	118,891085	106,1809	134,690835	122,20683	123,5455	115,445605	110,5011	99,56312	65,45002	74,70887	74,499405
ID1143	SPB6	102,28458	104,655155	103,860535	111,48046	117,602525	121,853555	81,02455	78,450195	80,20745	127,69096	90,65249	96,139125
ID1404	SPEE,CATD	94,48072	97,114375	101,794595	114,258525	123,674015	118,53216	91,68496	78,26781	108,704195	125,20421	111,31254	122,248855
ID2666660	SPRC	113,423685	129,07292	117,56307	79,600345	96,851305	148,58536	120,421805	104,8474	142,702305	108,45743	77,73621	88,633575
ID278067	SPRC	130,882095	151,92071	120,236535	151,087745	144,524975	172,78293	129,401585	102,74378	164,102845	157,98175	94,251545	92,151415
ID1083	SPS1	119,199245	139,95812	129,62371	129,392385	114,885485	133,553715	63,65525	62,352105	68,23344	78,36534	78,089475	84,37482
ID1121	SPSY,ARSA1	125,13303	127,616505	145,54515	125,076035	123,43279	116,504205	52,85786	64,183175	69,69096	66,72611	71,07534	80,05603
ID557	SRC8	106,359515	115,362485	105,0986	114,72728	116,66064	120,86302	66,773305	51,71534	63,57755	55,73253	67,9425	73,72771
ID1638	SSRD	131,19717	137,029805	141,74031	142,21968	137,8655	90,965585	140,26525	193,968495	246,96865	75,61905	79,71167	92,93683
ID1660	SSRD	97,313095	103,891705	106,889825	74,90005	90,10301	82,65245	89,49216	70,08023	97,849885	130,10976	88,320245	84,920955
ID853	STABP	143,38385	98,908065	130,66814	64,21926	56,06467	54,707815	256,95702	303,416995	270,457205	190,44948	178,39962	131,319335
ID1553	STAM1,PSB9	119,600405	137,72531	153,631825	87,96797	91,701445	91,33011	89,32822	79,0435	82,89761	78,63461	64,23562	63,38519
ID1119	STML2,ACTG	112,09798	118,926445	123,215135	103,52421	103,2023	104,3759	159,4007	148,51999	167,86284	212,22111	120,784435	109,842685
ID1695	STMN1	53,89669	58,86439	54,60472	83,424385	87,42896	107,19664	41,70146	34,413525	49,341075	52,74773	64,52604	61,27555
ID1686	STMN1	71,832675	82,7885	71,1557	90,25493	84,15384	70,872185	56,30503	68,896445	72,67141	105,35126	78,5817	79,60799
ID1689	STMN1	101,99064	114,49674	99,00122	118,578245	122,14678	145,0595	68,90617	60,104975	79,8009	57,36472	66,296875	64,233385
ID1701	STMN1,1433S	106,32472	114,469185	113,44882	131,00202	124,005985	114,20368	90,22288	83,03169	93,41621	99,24415	91,54071	101,759525
ID1684	STMN1,PTN1	61,867525	74,78417	60,056535	100,070285	91,6976	86,02186	47,03764	74,01922	73,0727	101,7943	78,53073	79,741715
ID1161	STRAP	107,80446	113,13418	118,637645	133,75262	114,37592	104,981175	54,20013	51,287755	61,38095	71,93283	73,772185	81,2601
ID1058	SUCB1,IF34,ACTB,ACTG	105,69806	116,448565	117,773625	147,653575	135,73562	123,773285	71,587265	65,64931	85,95372	63,22818	61,710865	67,95177
ID1214	SUGT1	108,70235	121,185	106,68276	133,848595	110,00017	103,157135	60,503625	51,51347	58,89194	96,87095	77,46692	82,826575
ID144537	SUGT1,NUBP1	103,46753	113,10995	103,6223	135,89852	119,092775	108,4261	64,7285	71,125635	82,228205	73,57395	64,64898	66,68077
ID974	SYDC	86,97485	93,54538	90,65602	117,609455	100,74391	84,81311	59,954665	53,59742	66,175445	79,31747	82,061985	80,66019
ID934	SYDC,HNRH1,PTN1,BLMH	103,665395	119,153085	111,917245	117,515345	111,62218	127,40256	78,96744	70,001	71,208835	1,00922	70,60591	75,976055
ID28319	SYG,CALD1	89,79939	94,18571	94,045285	135,68922	121,63572	116,382375	68,46554	78,825125	68,774035	301,48093	76,935275	99,200035
ID673	SYG,DPYL2	106,008125	114,54211	110,11315	124,787285	122,67128	130,5468	61,760235	66,63099	65,938745	335,18767	87,072685	102,351415
ID908	SYHC	113,43782	129,127785	120,117745	135,25863	128,693185	127,595975	81,602875	92,775735	86,622725	59,12767	74,178405	83,394765
ID377849	SYHC,VATB2,PDIA3,RUVB2	96,59824	103,035705	99,97237	120,030715	122,905535	139,037495	86,75861	80,055765	88,001255	81,67888	78,472365	83,686745
ID683	SYK,TRFL	111,005275	89,99014	109,16068	87,854235	73,35725	79,719485	165,94605	191,65357	177,317635	232,14999	158,334905	131,397135
ID885	SYWC	106,68288	111,54154	112,063995	113,01617	120,3394	135,474865	59,5189	64,15067	70,888065	63,56415	80,750835	94,400315
ID29100	TADBP,ACY1	127,924155	145,49744	146,11859	136,65021	144,913395	143,985205	75,777715	88,00546	85,93414	63,165	64,34347	75,18111
ID1084	TADBP,PPME1	112,75583	120,24165	119,55705	121,93816	134,559595	138,85788	66,901325	51,72873	71,315205	57,40883	68,53442	91,265715
ID3369954	TADBP,PPME1	79,97274	98,467085	101,58603	155,188115	1116,37975	224,34762	61,559015	52,20337	83,63606	50,18611	n.d.	0
ID1270	TALDO	46,037975	55,08936	48,19669	100,39156	80,507775	84,24322	54,89306	58,22396	45,86725	27,73946	81,31807	74,667865
ID1234	TALDO,PP1B,KPYM	111,22697	119,762085	116,246605	116,395715	110,128215	109,876195	70,29214	73,356045	75,823855	92,40881	91,467685	100,006925
ID890	TBA1B	95,264445	99,360535	98,678595	105,62553	122,56673	137,604925	51,899625	51,769355	59,016295	63,57524	69,318835	85,212815
ID402687	TBA1B	117,12177	123,91457	123,967595	135,308125	130,60964	145,656315	56,400345	37,457485	63,953925	65,40134	48,59121	61,24408
ID859	TBA1B,TBA1C,VIME,TBA3,TBA6,D	96,85395	93,464195	91,9938	100,274785	101,45379	108,234675	68,38951	68,65779	73,349875	77,80765	78,322295	79,436965
ID835	TBA1B,TBAK,TBA3,IFIT3	107,545015	105,278375	100,32752	106,006465	130,39683	95,09747	88,198875	85,674685	92,72429	74,9413	90,04079	82,325975

Table S-1

ID)1	Protein)2	Median of normalized volumes)3											
		co_0 min	co_30 min	co_60 min	co_24 h	co_48 h	co_72 h	PI_0 min	PI_30 min	PI_60 min	PI_24 h	PI_48 h	PI_72 h
ID840	TBA1B,TBAK,TBA3,VIME	101,915655	99,636605	98,34748	91,792475	102,26246	96,19998	93,71253	87,60761	84,059275	78,73732	85,75382	87,342205
ID904	TBA1B,TBB5	130,54446	133,56544	178,02536	120,23853	105,505645	87,763375	118,491135	156,160885	72,7684	80,52954	67,92255	85,873845
ID870	TBA1B,VIME,TBB3	74,32024	73,20761	74,88041	91,504015	92,307285	103,37053	58,49804	64,495845	64,35777	88,02015	81,75455	79,757405
ID906	TBA1C,PTRF,TBA4A,TBB5	120,201275	122,359755	136,379455	137,59037	120,529885	109,092265	105,004345	126,366745	93,92511	80,71481	74,47862	82,260945
ID2200	TBA3	147,31423	106,08293	121,26047	70,03221	61,0417	60,978115	258,5224	277,478745	260,80263	192,21894	186,29199	136,83329
ID15904	TBAK	62,582145	69,9074	66,820445	82,112295	99,091245	104,15983	90,8414	88,87464	62,66323	76,88643	71,40536	67,4793
ID876	TBAK	142,786355	141,096415	147,1348	123,776075	122,43476	122,566505	66,52314	76,97641	71,60967	74,06303	69,695805	70,13703
ID3111980	TBB2C	132,392915	135,627895	126,61738	118,231365	115,31196	122,50075	61,26742	73,39976	69,396095	80,21768	76,085185	73,740835
ID875	TBB2C,TBB3,TBB5	102,52119	97,70481	92,55008	119,504385	121,8643	106,14226	54,01545	63,53622	63,397155	64,9898	80,0242	75,78279
ID3104	TBB2C,TBB6,TBB2A,SMCE1	81,803805	83,27809	83,98931	114,81774	107,628795	116,17155	48,358695	50,15844	56,188785	74,46176	86,79016	86,94701
ID2998	TBB2C,TFG	58,41883	60,50244	61,144355	94,745255	84,48609	98,264035	59,442745	62,86605	62,896325	92,57058	80,29891	82,10996
ID3150	TBB3,TBB2C	91,90149	99,78985	93,058955	111,59926	98,79397	98,428675	50,116175	58,22764	57,2915	93,83082	81,773945	73,632975
ID93790	TBB5,TBB2C,ENOG	83,719345	95,068055	81,496645	143,02259	121,84606	105,802255	77,17916	72,479765	75,09299	62,56366	52,08801	54,03951
ID1780	TBCA	87,44634	100,59323	88,42513	107,698435	104,74288	105,34499	54,70108	54,586345	67,235835	89,22143	88,045435	89,28725
ID973	TBG1,MPPB	90,948685	97,33007	92,862345	128,465675	112,52212	118,789665	65,001165	60,659335	61,3763	64,22634	81,72607	81,48226
ID793	TCPA	118,4442	132,46762	121,64892	150,74127	135,705415	121,284705	71,17259	68,904845	73,3631	89,54403	96,906415	103,367765
ID29990	TCPA	128,252835	121,863135	138,87866	121,118085	95,69441	105,18873	188,365255	257,51989	220,91121	120,30771	120,382635	111,18875
ID17458	TCPA,CPNE2	121,3886	133,59307	128,202825	135,124925	129,34916	114,330135	81,75712	98,18757	98,460005	111,20875	101,850515	77,71129
ID888	TCPB,RUVB1	116,071455	130,14983	120,667715	129,81013	120,38592	115,7965	81,20948	78,62589	81,434275	46,59495	88,87793	93,824995
ID804	TCPE	70,23104	78,113725	75,16691	104,659645	101,158505	107,304815	66,396135	52,153985	56,06378	85,72547	85,17935	86,93048
ID405373	TCPE,FKBP4,CPNE1	106,95971	121,302995	115,6175	134,193115	122,4875	140,74468	88,17369	80,571535	81,91775	100,3034	107,60261	114,583735
ID847	TCPE,HNRPK	79,91364	82,684535	82,59949	109,725285	102,800305	121,50367	67,023255	53,42508	60,103795	75,18182	75,22213	78,638965
ID760	TCPG	106,6701	123,62841	111,37225	134,811175	119,877655	119,970525	78,763685	78,360635	77,307315	59,51413	85,22318	89,39036
ID771	TCPG	86,924765	95,184065	90,871005	113,38217	107,666225	113,368925	80,514475	84,00995	82,67404	8,4973	88,07452	95,044245
ID796	TCPQ,HNRPK	119,93508	131,854005	126,953875	139,888865	137,684675	139,13236	121,410645	84,280435	77,43332	87,93408	100,742415	96,371695
ID2000013	TCTP	110,466475	124,97983	126,256405	148,32412	83,999855	56,560215	78,29314	91,23241	90,67366	150,39073	98,63491	76,88326
ID1576	TCTP	105,97151	118,85147	109,524155	127,73556	117,57	110,3025	97,4242	87,38393	91,426205	82,03224	82,040275	75,942345
ID1501	TCTP,CBX1	103,716475	117,796085	106,958135	116,871425	100,53095	89,14123	82,495145	75,33266	86,211285	100,51043	81,344005	81,840165
ID2310	TEBP	94,731895	101,416955	108,854295	120,00349	106,302345	139,28641	56,273185	58,49522	61,71551	51,86479	71,370985	66,065145
ID1610	TEBP	89,17078	96,16524	103,02015	118,67615	96,574585	161,00998	55,218705	58,12572	62,25732	56,6445	60,94245	63,2422
ID542	TERA	79,298635	86,71852	80,04221	103,71703	105,17944	110,9048	61,57492	52,12901	58,17247	65,62233	80,352335	81,879295
ID540	TERA	105,525485	105,642535	108,679135	139,77478	136,626345	110,745185	77,224405	67,864795	71,150385	68,02345	83,722045	86,403375
ID9875	TERA	119,54127	132,83385	118,77012	145,72327	142,24054	135,496795	76,30357	63,97739	68,104795	65,99823	80,272275	88,37103
ID1821	THIO,RFA3,LEG1,COX5A	105,50094	118,3811	110,32622	102,50779	142,294415	107,86662	70,529195	81,415325	86,134135	175,51911	136,73071	213,645755
ID1115	THOC3	118,00706	139,786045	123,76074	118,417415	106,31198	134,065365	94,50324	103,14751	103,59713	74,46585	86,110755	88,40038
ID607	THOP1	106,390985	113,64369	119,15148	114,015905	128,859975	137,4544	114,12254	86,705615	114,26958	80,13941	93,260585	82,67909
ID488	TIF1B	145,87498	161,830615	147,937675	161,718875	185,06094	164,942965	104,41485	60,51613	60,474535	54,08774	97,496385	87,764175
ID1419	TIF1B,1433G	107,39388	112,527045	121,67679	108,170435	113,015455	113,84424	63,69867	64,513815	72,52019	81,6556	77,333185	79,784275
ID479	TIF1B,HNRPU	110,74223	114,960075	113,378275	177,022195	173,22841	144,30218	59,920425	51,134945	55,654905	33,80963	79,909245	86,493745
ID774	TM1L2	127,78688	84,645695	131,60445	56,247235	60,237875	57,60581	233,48109	277,38272	228,942725	215,68806	194,10124	138,725575
ID3085623	TMOD3	97,930885	102,895475	100,729165	81,958625	82,744185	65,35633	57,53561	54,92819	64,880675	78,64657	77,819035	46,59135
ID1131	TMOD3	77,711195	84,45241	74,23272	118,08982	115,418895	114,30412	70,56995	64,001985	67,60251	91,60188	79,68323	80,475485
ID1315	TPM1	101,365075	111,758785	100,38814	113,95565	125,371045	132,556205	104,76019	92,61746	97,46175	75,01152	58,863005	68,803065
ID55610	TPM1,TPM2	82,986375	93,63326	84,37219	119,07904	136,40331	137,12414	87,25874	68,834825	77,66554	58,43241	50,426705	57,60713
ID1210	TPM2	81,584245	92,485725	82,264965	105,05118	94,63098	86,041155	80,1584	64,57861	71,17692	69,48085	63,270005	72,592125
ID56308	TPM2	85,1463	96,30605	87,923295	103,712925	101,32077	111,28414	61,92655	57,77735	61,567945	63,58844	66,82666	84,42606
ID87491	TPM2	90,981105	81,03269	97,02572	125,076125	117,85711	92,60504	52,322035	58,118485	66,34519	114,337	79,622535	97,08293
ID1372	TPM3,TPM4,TPM1,TPM2,TPM3L	118,71201	126,30024	124,77774	120,1063	126,485885	119,447845	75,781595	70,77843	82,186375	69,09847	84,920815	89,197335
ID1374	TPM3,TPM4,TPM2,TPM3L	97,676605	106,100005	96,6471	113,118945	114,091785	117,97073	84,14596	75,757775	79,667005	76,30327	80,216465	85,51914
ID1369	TPM4	90,9618	98,59639	93,724715	114,258025	114,72304	112,859855	74,107035	65,420815	70,5944	74,19477	75,68051	80,016505
ID13616	TPP1,HNRPF	87,665815	93,232335	93,846315	109,49387	97,64739	98,545675	93,662655	82,72806	78,843675	83,72728	69,557625	66,07307
ID1153	TRA2B,CSN4	105,808135	119,748975	115,14961	127,34027	122,18953	120,9683	50,903235	48,01539	60,918375	84,72603	85,7693	88,50387
ID688	TRFL	119,949005	87,835945	113,68177	66,88199	64,408675	65,233095	199,671825	222,852575	219,58604	116,59035	163,649825	130,63994
ID837	TRXR1	113,523995	131,82959	127,635555	124,972565	109,16343	101,74641	95,429955	120,54153	108,702895	15,88342	108,252875	110,481085
ID849	TRXR1	111,94964	114,143085	126,76321	121,061955	110,843655	106,1372	81,28055	78,73571	92,481545	27,71662	92,887255	99,84392
ID751	TRXR1	134,174835	85,3874	134,380105	58,83923	52,720775	49,18618	255,004225	310,419775	255,31109	197,29714	185,58202	133,53722
ID1335	TSNAX	128,70297	123,710595	136,11567	91,542875	96,3565	98,75087	163,537965	176,831045	181,32941	81,89616	146,31031	115,32371

Table S-1

ID[1]	Protein[2]	Median of normalized volumes[3]											
		co_0 min	co_30 min	co_60 min	co_24 h	co_48 h	co_72 h	PI_0 min	PI_30 min	PI_60 min	PI_24 h	PI_48 h	PI_72 h
ID19644	TTL12	109,4187	100,261515	106,1795	124,425725	98,00787	99,95302	111,263125	119,323215	113,178255	137,26886	129,3671	123,68009
ID1693	TXD12,STMN1	55,782925	67,01284	56,565615	77,627035	69,6899	63,36991	53,049035	57,30583	65,00961	74,43592	60,493915	61,272415
ID1033	TXND4,ACTG,PRS6B	100,700625	114,42747	108,644575	118,217035	129,13127	124,850715	101,098185	106,368385	110,42778	131,26682	96,075455	97,018615
ID203683	TXND5,ACL6A	152,928655	146,76655	170,039595	101,19542	102,25094	87,316245	180,268895	195,056605	207,976745	272,79647	139,96844	146,655335
ID992	TXND5,ACL6A	235,52853	238,642075	250,33084	189,80415	178,50368	129,81616	257,009605	248,08958	257,26296	108,72986	92,319725	88,216715
ID977	TXND5,HNRPF,TBAK	106,534615	121,835695	112,283065	110,715365	107,032715	113,78369	143,355735	139,308725	146,516375	128,02817	76,894945	74,90412
ID13083	TXND5,SH3G1	115,78492	119,84733	117,36392	120,71595	129,171925	128,35887	43,693205	41,71864	67,43993	68,61202	72,92071	78,12844
ID1269	TXNL1,ANXA2	114,642035	121,322165	110,08571	141,671065	119,460035	102,82043	78,50654	70,66707	84,87337	106,64523	93,85618	91,698315
ID16669	TXNL5	99,913465	113,716805	101,22761	114,723395	184,9951	132,481935	53,94763	60,422415	71,462315	78,60654	140,77791	282,275225
ID36428	U2AF2	102,359515	105,466185	106,76724	124,87492	113,333885	156,44211	113,0564	124,35203	111,653735	0	119,226365	101,7609
ID811	U2AF2,DPYL2	110,481865	108,47282	112,21673	141,717165	121,38973	123,67223	112,28858	136,3237	121,84194	0	112,959085	118,34513
ID1358	UBA1,3HIDH	123,774175	135,213025	140,202705	134,59448	151,634485	145,5793	87,970475	93,066035	104,8903	70,95778	70,576515	69,423255
ID469	UBA1,TIF1B	115,93437	118,778855	117,12309	139,91161	152,774025	141,644105	64,15116	46,57239	52,2904	59,21721	81,77593	87,81645
ID478	UBA1,TIF1B	119,841685	127,442445	122,45601	136,708115	153,33638	153,16965	72,93023	58,492275	62,187285	54,95988	81,829295	85,724265
ID3542	UBA5	70,840585	83,18012	85,96799	129,9399	123,099415	108,543895	51,65008	45,637315	55,643155	43,18664	61,822665	59,19131
ID1116	UBCP1	101,82225	110,24278	111,613705	122,031605	120,900345	120,44348	59,28245	57,415305	68,949655	39,26686	95,06546	91,129395
ID554833	UBE1	118,444595	120,59193	121,933225	156,662625	152,943605	133,11169	61,55029	46,319475	52,807175	50,75163	73,945325	74,42804
ID466	UBE1,MVP,UBA1	116,71923	122,64151	116,33569	150,466055	142,208825	130,339855	54,85715	50,36247	55,494095	56,43027	78,34836	83,431535
ID1526	UBE2K,RPE,CBX5,GSTP1	114,563135	117,310005	118,789995	121,825895	116,019855	118,62817	76,758485	98,695045	97,89192	117,44636	96,657755	92,92888
ID818	UBP14	111,675885	115,23956	115,723225	119,237815	114,04965	112,10458	59,26781	56,03193	65,109055	81,5256	79,88784	88,946395
ID821	UBP14,IMA2	96,145095	99,033415	101,150525	112,095685	109,7069	109,522825	71,658985	76,68781	80,59193	96,10563	98,20461	99,23621
ID545	UBP5	92,24255	96,318495	92,186215	115,05011	116,555285	110,035425	40,69518	38,485135	46,86629	66,16786	75,129335	79,296595
ID550	UBP5,P3H1	128,3653	139,0131	131,3987	144,45944	137,89354	132,22175	55,46841	53,79609	62,3893	71,2891	80,059605	82,15437
ID764	UBQL1	124,391515	78,49392	120,011975	53,230645	49,52252	50,64605	225,99419	286,0227	244,42998	225,84388	211,407045	158,83212
ID1491	UCHL1	103,449245	120,131415	103,915355	122,710555	110,02661	110,18761	80,425555	89,98743	89,30941	81,32621	84,03044	97,30124
ID809	ULA1	109,55114	106,97498	117,195135	124,64378	127,78734	124,594475	59,93977	49,04721	52,36223	67,58078	78,54373	85,67404
ID1013	UQCR1	108,34266	116,835935	117,606495	121,206755	115,80193	106,887515	129,941135	123,388465	137,95888	83,37747	84,17406	94,21168
ID201269	UQCR1,HNRPK,RUVB2	135,685375	122,781355	158,92029	105,26503	109,38045	100,992205	21,41851	20,189925	26,204505	49,84153	44,396575	48,96495
ID1031	VAT1	115,13403	119,1352	129,73351	126,19932	114,644225	108,125795	91,310995	74,00353	81,326505	3,23786	75,04638	80,42105
ID975	VIME	83,15018	140,06044	96,881215	118,83479	112,790465	70,52669	133,946755	156,328995	130,350435	125,28933	112,55645	94,776095
ID1044	VIME	111,197705	176,29779	114,191915	106,31919	97,51987	61,438865	224,702165	254,699995	265,488905	126,86265	132,036845	91,47436
ID2117397	VINC	92,75261	99,547945	94,565845	165,01641	103,615765	139,061865	50,545035	51,514545	59,007285	24,47317	3,089735	10,739025
ID432	VINC	85,425725	93,377645	87,658055	117,00962	119,91139	119,645315	61,571035	55,069445	59,608	55,97098	76,59903	79,645155
ID433	VINC	112,184425	125,17678	118,099415	131,706135	141,29929	124,652305	72,74277	66,61792	68,42316	0,27231	77,305985	85,00315
ID682	VINC	75,880125	80,48632	74,85928	104,391945	103,09061	111,25333	53,89884	56,996675	58,83679	88,87081	89,123585	99,9335
ID431	VINC	103,46287	109,34878	107,09362	123,61521	128,01261	127,149945	82,67701	63,614915	64,563735	58,25315	70,519515	71,259975
ID1325	WDR61,ANXA5	114,6221	126,03079	119,291135	117,136485	110,72544	111,48382	108,547035	111,272315	104,76369	86,26614	71,854155	66,92673
ID581		6,232935	4,434315	16,111135	10,725475	3,39514	80,43324	19,214065	32,247915	55,385135	39,05009	13,522925	17,01142
ID1730		86,13627	94,20698	83,206125	102,87501	118,39894	151,434285	67,747905	62,6198	78,242945	76,12357	88,31966	91,83385
ID2753		106,80557	113,23479	122,26811	50,84645	53,7365	57,57306	121,246665	118,84761	98,792395	35,06248	49,10178	62,00709
ID1431		99,5535	113,253725	107,259575	113,56929	121,314195	128,35081	85,691455	109,26613	103,0998	97,3635	89,966155	95,694225
ID401		100,66903	115,47809	108,009455	142,418425	145,948555	143,893815	65,80364	53,513195	47,626205	0	69,26853	71,74029
ID1960958		58,051785	64,4183	62,10662	96,162265	91,953705	85,491955	35,72785	39,197205	51,217685	155,91061	85,24778	59,87007
ID802		123,13977	96,14617	103,43489	61,341435	64,309115	79,8477	202,31447	216,40619	214,05071	178,22072	138,192275	109,659615
ID27793		69,263905	70,2387	65,844525	125,77468	151,66914	120,71254	58,231095	76,935725	58,48223	53,10989	93,1551	100,441225
ID16921		88,03633	95,456145	96,73203	103,383615	86,55153	77,762065	77,37105	67,923775	72,530875	134,95141	77,456475	66,51837
ID707		138,72393	167,22494	152,663115	197,69686	156,072125	93,94491	137,598085	160,34473	153,151705	136,72186	112,229335	112,808075
ID112515		135,593845	154,044825	139,802755	190,64652	160,12257	125,87627	140,86908	149,493055	122,896485	74,27103	73,41889	66,72025
ID178566		772,7063	982,716515	562,04185	303,22999	259,182505	146,78453	66,439185	82,12926	91,71515	60,15287	124,06196	146,99415
ID1265		87,06697	100,18235	88,600205	105,782025	87,670195	99,143215	99,75974	76,573725	80,66728	72,20896	88,10755	93,49309
ID102094		108,528315	71,63596	107,318585	48,99068	53,91045	51,81122	154,35961	204,904705	165,75106	213,1786	179,32464	140,70063
ID3231778		97,13968	102,89376	102,29793	126,730005	108,81318	119,62574	58,178235	47,194395	54,87458	68,38321	72,561575	84,49456
ID3765751		461,174385	420,234155	472,21275	387,600745	356,425355	382,6994	524,99671	555,628175	413,39074	318,43144	230,481315	291,95881
ID62715		82,154425	91,746385	79,38405	89,62912	90,72715	105,48491	89,265025	86,85085	85,03472	57,40375	69,55414	74,605865
ID108562		173,12704	139,262065	191,87752	99,43306	120,203275	68,394845	265,681345	312,92367	291,842	181,25774	204,582975	153,185785
ID1816422		230,32637	162,968585	117,47139	159,697895	118,70819	125,8418	103,346465	184,07925	192,412795	309,92182	24,008015	29,29396
ID181505		128,86777	111,06767	136,571065	113,846395	128,69617	102,1403	185,55051	168,10143	170,88952	156,86225	140,769085	117,59678

Table S-1

ID[1]	Protein[2]	Median of normalized volumes[3]											
		co_0 min	co_30 min	co_60 min	co_24 h	co_48 h	co_72 h	PI_0 min	PI_30 min	PI_60 min	PI_24 h	PI_48 h	PI_72 h
ID1841741		161,13132	97,30852	168,172385	83,302995	70,67723	29,20386	247,270375	238,745735	265,786495	103,11101	190,73368	159,24913
ID1570		321,772915	162,11853	200,714745	177,20264	116,36219	92,734045	346,738935	223,230485	254,24002	38,71363	212,16664	101,770695
ID1096		132,607325	116,965855	161,920135	93,82334	75,22922	88,90082	126,603175	134,24826	139,171015	73,45716	57,99393	49,54109
ID356		107,100065	117,515915	105,62896	129,071715	135,46643	140,40222	75,30208	66,54205	67,37492	52,48343	66,84895	76,414845
ID1009		77,09164	81,765005	84,11109	118,97128	112,32407	117,257255	68,729085	57,659385	70,776455	88,18245	79,343135	72,49686
ID315		102,975615	102,91963	94,90443	183,883225	170,890685	287,866025	42,154565	48,37063	11,67087	55,08009	64,74959	15,558195
ID281947		206,34063	261,84463	167,201505	131,895165	113,558145	124,46121	201,31874	292,14533	392,00778	162,82585	132,306385	112,632625
ID349		81,501075	89,914935	85,983305	113,245185	108,99652	137,10855	75,687125	55,708795	57,273075	34,34323	62,013595	61,336335
ID3130260		110,209225	132,91903	108,810875	170,776775	164,767645	144,658345	83,937905	74,248255	73,89623	51,37906	70,036975	80,21871
ID1514		115,85807	120,00256	128,28343	72,10095	96,83936	98,526485	106,432325	123,975595	126,56752	34,9522	127,25456	97,96796
ID546917		104,014925	94,537315	105,334315	99,951165	106,972875	143,83009	100,87437	114,81104	95,79928	157,68661	113,53191	97,055365
ID1666		53,189925	54,62344	51,047455	89,37903	92,508065	108,87227	48,56132	45,18331	55,21435	74,58108	77,78287	77,002795
ID1062		125,52818	121,66159	164,7529	128,43798	120,401805	143,791725	56,966405	104,315115	34,5507	45,69837	68,838985	66,246545
ID1045		73,92558	86,59794	73,658525	120,380835	87,05214	347,083875	19,36439	31,56905	32,417355	107,05958	87,50378	71,867705
ID426		92,84214	90,84209	79,14705	118,39335	102,46039	127,92083	93,366705	78,802435	83,96011	73,00625	106,789255	101,0483
ID516		119,269185	142,68415	105,3186	144,52316	128,99709	185,84614	91,79652	115,360895	92,834655	110,73658	78,910785	81,11365
ID1094		14,554775	13,33029	55,945315	132,24359	119,22258	208,034795	21,58023	24,163605	22,11484	140,85488	68,882795	103,44578
ID2068580		92,05072	99,94649	92,116705	133,44656	108,71277	125,251985	101,57966	92,75868	90,51274	182,36299	81,830255	63,348705
ID1720		198,20317	244,87285	263,080745	302,32105	524,89974	2856,40728	84,588625	765,49122	293,378365	49,17638	16,63086	54,74494
ID528		116,3627	136,38513	117,62669	191,640605	187,643445	149,88271	73,4971	62,012805	59,16886	64,8167	72,06423	75,884635
ID2924537		83,97005	82,17913	105,09132	107,994765	91,281165	63,627115	45,29174	35,009075	44,545325	87,41262	75,41953	91,482785
ID1736		96,11475	114,280235	107,779195	134,287375	130,7525	128,149265	79,51133	71,803365	80,39008	99,97064	123,194675	136,81095
ID742		168,854675	118,915685	171,66444	101,35831	154,828125	84,938925	276,91089	462,274535	352,80751	180,75551	203,142165	171,977215
ID1652		82,91034	89,250685	88,279165	146,53124	122,032365	110,09038	70,09897	66,08466	74,778845	16,27733	101,006225	110,426305
ID86593		60,179555	69,959585	67,27187	100,946295	84,41753	101,80594	71,621085	72,6054	76,616425	118,25527	91,945355	86,85079
ID3432480		119,68745	112,477675	120,68502	96,959205	84,79824	69,321475	115,738205	149,642	123,58664	235,47502	177,798765	130,21372
ID39482		144,043165	106,37228	138,018315	69,113745	59,525705	89,1117	208,47365	186,6025	188,794385	144,97297	139,257835	102,001955
ID52561		80,02076	92,36098	78,22415	86,19754	86,67637	115,9488	82,78556	78,62991	93,58393	103,92597	66,53423	71,250935
ID1847793		79,380635	89,863425	79,829575	163,35009	181,5684	149,521945	75,40394	87,63061	96,579775	82,16631	38,638275	41,54575
ID24119		67,02436	71,94608	71,466245	109,165425	108,107465	141,77515	68,248975	75,10324	70,09376	71,58679	74,91191	66,453955
ID165075		84,941535	137,590315	110,792795	139,31429	133,58415	109,94176	68,921025	62,440385	76,018555	97,22944	86,41604	105,45131
ID429069		15,026135	9,855795	7,28387	119,38847	89,246525	93,435575	4,120895	3,35227	1,407595	130,2833	80,90039	76,34109
ID353686		94,798875	104,55524	93,46616	100,229205	93,20995	121,543605	46,168335	44,733695	59,372825	37,42926	70,51505	64,021545
ID2728784		24,14911	8,906895	36,80894	45,16608	20,2986	27,65649	237,9151	169,61902	202,07841	55,51283	51,262755	34,511425
ID1383		103,1539	116,504405	111,66015	110,14962	94,788145	91,022865	73,829755	77,35495	89,25587	66,41556	69,234935	67,44099
ID1758		128,450955	145,494375	150,435165	130,292445	119,44885	115,97095	112,15519	158,79685	109,295745	88,53301	82,02164	80,780845
ID1359		101,75636	104,10078	123,606605	103,03308	121,13189	122,617355	64,493025	61,51559	69,21603	85,11095	96,37376	98,61623
ID2031934		104,957165	163,394145	113,229085	121,00409	144,07182	201,455875	29,264865	41,847275	46,08488	67,12947	50,359445	75,95036
ID1837103		143,64849	107,900775	146,153465	77,26538	78,55733	30,108705	149,80063	99,776415	114,919815	139,94232	138,70595	173,80362
ID31086		114,5476	130,69207	123,44346	159,488895	130,79704	109,04345	74,34566	80,148685	90,46542	0	78,762555	120,56691
ID1289		105,008845	118,68994	123,29302	117,52078	105,30866	112,9206	131,19465	114,383835	112,19768	124,99535	85,83079	75,92992
ID1683		44,09344	45,927825	98,95058	145,977035	102,92917	56,519115	1054,43011	113,452595	842,861015	471,86426	728,76602	346,367055
ID1782		52,265245	54,30075	56,12268	67,941925	137,27136	126,608925	38,33007	68,793225	40,57865	108,41962	72,56937	70,078145
ID3078712		71,035405	85,311	68,306335	47,643345	36,30943	54,264085	140,76274	169,50461	202,46503	57,54324	43,547595	83,97946
ID3762560		102,01348	109,26164	102,18582	109,191635	98,559415	82,77562	72,72595	62,09902	63,407205	77,9932	54,85159	65,403
ID773		140,81262	97,46507	142,041085	68,21591	70,74776	62,241395	227,302215	271,267475	219,95953	186,80983	182,887775	130,28261
ID7589		106,845415	86,52748	107,106485	68,83235	67,11967	101,437685	184,24436	202,13527	170,721865	153,91605	120,495755	101,012955
ID1007		75,969375	110,21321	106,84387	184,13656	116,934	90,758935	50,46594	57,42326	38,282105	67,91756	53,25103	68,941085
ID1676		156,379075	334,992755	770,55968	58,2499	88,32513	104,358395	279,779975	189,61099	244,60932	117,44204	102,736775	95,866845
ID373664		95,4408	103,19678	102,438935	114,037805	113,275715	116,00423	76,669805	78,670465	80,4436	53,24054	62,275965	72,623085
ID1484		98,899455	112,95765	105,38355	128,846715	116,282765	116,094715	126,03371	121,290175	123,33277	123,65868	100,146415	89,16201
ID2288948		265,247605	167,343935	332,21391	118,614515	118,19521	105,48726	582,683875	59,52059	229,716065	0	56,17431	1,050405
ID2008587		112,52745	135,42539	95,958515	109,22624	245,062905	170,678945	74,54659	80,77098	93,895825	68,54688	74,8272	89,075235
ID750		143,17037	89,198855	158,871965	67,240245	62,161255	38,539685	284,638885	388,179195	309,76899	188,58623	207,752235	154,44043
ID715		99,44551	87,865955	93,00363	58,971685	45,21444	58,1437	143,77526	127,284025	118,892605	175,64656	146,135925	141,375405
ID124470		76,598215	76,78254	71,776385	89,51939	93,52418	108,11386	64,02637	61,540075	68,869855	79,28843	82,173975	77,112295
ID1592		79,28551	126,22626	78,48521	132,383445	93,26168	139,955725	62,06761	82,06586	91,061955	0	81,981515	75,219985

Table S-1

ID[1]	Protein[2]	Median of normalized volumes[3]											
		co_0 min	co_30 min	co_60 min	co_24 h	co_48 h	co_72 h	PI_0 min	PI_30 min	PI_60 min	PI_24 h	PI_48 h	PI_72 h
ID380		88,377105	87,07969	88,62656	120,49765	123,94714	122,691945	62,03486	53,01686	55,608565	58,94388	79,734945	75,28378
ID3179		128,88724	80,81253	121,37852	62,015585	62,28061	58,177865	218,45055	299,81273	250,221615	216,05134	200,93181	156,86926
ID898		110,028805	106,243185	105,24738	84,342925	83,302645	86,274825	132,69782	156,284005	142,833955	150,57505	132,746495	111,17832
ID538		87,364545	98,39366	91,264845	104,249575	123,547235	136,290835	81,376935	75,358815	82,320045	36,2306	72,766755	83,94984
ID254557		103,789145	90,53736	103,270725	90,03832	102,11021	134,68596	87,789255	130,112715	114,248805	128,78028	135,92447	98,80056
ID1739		84,139935	101,56645	81,73432	106,97691	71,85916	78,022805	106,578745	388,475735	152,131535	10,43426	48,14895	25,88684
ID1136		70,429495	70,84655	86,609605	84,27378	86,38481	95,145145	33,35413	37,95928	44,44173	66,56184	71,14884	77,95406
ID636		69,88651	75,45084	69,98012	111,622545	95,260975	103,573375	58,114705	58,1876	66,458355	85,23296	64,021275	73,28544
ID582		6,632855	5,50251	6,41177	20,06145	9,815965	75,764145	55,49522	71,878195	106,61825	70,52905	31,268095	37,38369
ID1603		90,396715	99,766735	104,829595	273,979095	386,89461	178,48715	39,721805	28,29353	51,89632	7,3207	0	0
ID2296834		92,71913	106,782285	81,94796	131,15208	121,37125	44,63881	97,529505	83,132305	98,372525	0	0	0,02363
ID347052		99,69403	103,792005	106,81354	100,859795	101,89727	123,505885	81,42482	99,53706	100,1747	96,38336	87,987925	89,42095
ID2340727		105,38086	96,72409	130,036565	125,427995	90,933005	87,51563	98,36641	179,64739	121,14584	68,83592	108,99871	111,371425
ID1017		102,715735	93,88115	119,70717	93,68617	89,083975	95,87516	59,49395	48,694975	65,318175	100,57102	71,174495	71,849595
ID61022		48,50716	59,355705	54,175665	83,828365	74,908185	68,76726	70,53261	69,06968	86,53181	64,3946	88,582915	87,776405
ID1477		105,090125	120,794285	112,706535	126,588245	111,63741	111,98587	147,54183	145,746045	137,693405	105,89724	88,625395	81,66728
ID351		93,90148	92,965345	85,50367	122,63948	123,9565	143,302115	62,424255	41,13377	46,625425	15,4491	42,23918	57,481415
ID1379		99,30541	103,78633	106,74413	110,74301	125,95388	126,67059	71,754995	79,40071	81,2157	52,65429	63,589695	51,256235
ID1911469		98,6604	113,000005	99,847155	106,9474	113,78101	81,365965	61,86127	70,338835	81,25766	125,39405	134,51135	131,969515
ID749		120,788235	81,971075	121,37984	71,02086	64,182375	53,53123	243,335125	271,19386	240,095925	158,86069	151,539415	118,352005
ID1151		82,605375	99,74517	83,24822	78,587935	76,06042	84,20647	67,34096	59,484855	70,57011	73,21351	66,02352	59,281145
ID425876		79,747625	85,82524	71,67621	111,669315	92,645485	92,309245	70,091375	78,472285	83,002695	99,86491	92,979205	90,896315
ID331		90,55219	85,343945	88,91503	95,816555	98,126105	71,509	95,014245	105,85378	92,393175	75,70371	114,224285	69,41683
ID1648		75,053465	84,833545	70,2534	119,80042	133,311	117,894795	57,51567	62,63197	66,69285	65,45784	87,374925	32,665035
ID1994828		136,161425	151,22044	167,11965	271,094465	175,823865	178,90986	191,18362	189,22415	205,06698	114,2509	228,68546	1182,22776
ID2209869		78,29805	87,168855	82,71662	86,703745	79,98745	51,47825	182,462355	187,498785	168,05393	97,96358	60,487545	43,23857
ID1499		138,217995	200,174875	255,398235	156,693915	161,062335	113,679955	154,04175	234,651115	211,35599	67,0352	90,605135	89,826435
ID3395583		102,85834	97,316395	110,76302	122,12727	122,232335	107,862805	62,86723	70,426635	70,359	135,93695	73,45559	95,34693
ID1181		88,519835	88,26271	91,88925	105,25712	99,505235	100,891575	72,82862	76,314305	78,733415	83,83463	82,32419	91,30174
ID463		69,381215	74,421295	66,27172	115,35364	103,27835	135,41011	36,82093	61,188515	40,2627	105,5385	135,80361	134,887935
ID242682		81,565035	97,31956	90,05243	124,46063	115,97679	123,822785	66,35722	46,02344	50,161415	24,89373	55,21402	60,936635
ID306		56,943745	50,703175	58,384875	220,12324	267,186925	126,90253	53,75623	22,826105	12,8246	62,39053	67,45368	80,95834
ID2110071		100,928565	115,674955	95,32706	178,42056	132,16878	155,346015	17,206025	15,014155	18,324545	21,60165	19,728275	26,71356
ID1486		87,691955	93,018765	98,446945	123,27965	133,20983	73,965725	57,613345	82,9919	73,517625	60,65876	165,68458	178,76286
ID841		124,4516	90,06208	101,14182	55,183705	48,51698	51,16802	246,86804	248,534205	224,7366	237,67207	195,04463	143,550755
ID1233		104,118515	115,202745	105,144555	117,915015	95,789345	80,82898	59,70521	61,049385	68,679905	80,30705	60,22285	62,451785
ID314		96,731355	108,13342	89,15566	164,188675	147,10008	117,2749	55,439955	56,981495	52,66494	168,47583	81,377515	78,703135
ID2108161		98,41121	109,01152	100,257565	174,337115	109,372	108,19436	70,76658	239,0594	78,27965	74,71845	10,89616	28,35581
ID114983		94,363675	119,268345	81,8683	106,72113	84,80699	130,58276	43,875945	69,012525	64,359285	42,85498	72,585965	75,63005
ID473357		112,05864	118,725355	124,966305	115,434815	97,04952	96,78621	86,28712	111,35666	104,743205	23,22176	125,294455	129,235575
ID476		89,00079	93,048975	92,26404	115,35367	98,65896	98,642985	58,397695	49,13252	53,013425	70,36824	77,101035	83,13232
ID1788919		111,269445	119,04348	117,37959	154,470435	139,473575	141,71591	67,454175	74,56153	80,767125	23,13393	51,459055	36,22427
ID402		105,79354	116,143225	106,59456	142,212255	146,651875	141,32827	60,22076	48,50847	51,049265	0	67,864535	70,84734
ID449534		102,488595	106,12051	104,241575	115,23173	114,639905	123,91525	82,828025	79,155015	83,1146	79,21251	72,03713	87,98822
ID16755		135,224315	135,27378	152,97788	74,146345	94,73654	104,793745	93,0135	95,70748	133,903775	74,74198	51,792345	45,53033
ID4808		88,412985	83,140215	80,540625	106,656295	95,38755	96,72629	81,88422	87,54206	87,149555	95,32626	93,12337	87,69992
ID42780		155,41556	94,37891	162,512135	57,390745	67,29188	44,134065	300,922395	429,302975	329,247825	229,14217	226,46821	149,724805
ID3761682		133,702295	89,459375	108,91461	93,824045	76,256275	155,38212	178,15039	254,945435	223,82325	91,95881	127,706325	89,361685
ID6662		110,436265	122,17984	102,669125	141,68698	138,281765	127,64622	82,731375	69,07143	90,25605	201,51808	86,89085	82,129295
ID2300514		147,794165	192,933065	144,531325	93,207805	62,878235	33,57106	78,802035	76,479985	95,376625	388,95431	274,336455	205,42948
ID3163353		82,03348	82,27552	90,51836	121,8001	154,67914	163,41967	156,69698	101,50301	111,60204	106,44448	113,315315	111,77589
ID328907		76,326795	98,269765	44,398375	111,5915	92,062315	146,56415	59,44141	49,757665	52,112475	47,00001	50,616165	68,397605
ID1158		121,629685	143,29404	146,423005	156,759705	131,80607	111,00577	43,51615	33,809155	43,848445	90,51886	97,725775	104,356855
ID1189		92,928645	103,6122	95,706425	112,18819	97,943115	86,652545	105,82626	94,02274	81,4423	89,50998	84,69004	91,19268
ID2035512		13,118245	9,781365	26,64175	45,48058	21,171585	130,634095	12,714145	19,973045	19,858755	36,38572	32,03229	48,66722
ID6678		94,488975	104,616	88,027325	125,270265	119,816075	108,05992	78,525625	63,069135	84,646225	196,02656	88,066465	81,580915
ID305		53,26108	118,48324	121,809095	205,07473	475,077105	135,41761	34,85512	36,35836	0,01695	56,61917	75,42808	94,178445

Table S-1

ID[1]	Protein[2]	Median of normalized volumes[3]											
		co_0 min	co_30 min	co_60 min	co_24 h	co_48 h	co_72 h	PI_0 min	PI_30 min	PI_60 min	PI_24 h	PI_48 h	PI_72 h
ID1982157		92,46122	102,78431	93,391065	75,87785	63,22056	57,76269	54,620555	56,98805	64,545985	21,55481	17,503345	14,954295
ID1991205		107,427295	100,11006	83,81642	657,272855	167,91068	296,81407	81,792685	108,83948	88,200045	107,12638	n.d.	1168,75041
ID33115		109,59824	117,523625	132,40252	105,23216	114,835305	90,636495	98,57807	70,129315	113,55104	0,65859	68,43863	53,436405
ID1266		140,031425	581,87228	112,709085	116,91519	89,383365	77,01924	143,427855	115,2337	91,60699	81,39847	88,125875	83,070055
ID1814733		127,57977	120,099	127,478965	141,787775	113,40309	121,26883	139,04236	175,131945	186,86639	396,53648	86,91187	55,34552
ID2148299		20,03609	23,860425	29,55533	48,379315	19,709955	71,330855	65,31455	60,91062	69,263185	31,59631	34,5504	23,185355
ID1864524		107,9477	125,788155	205,86641	81,85601	112,25786	185,561905	82,167815	85,90324	80,08436	128,02778	92,14427	74,85234
ID1602		66,175675	85,39294	71,21491	114,67009	100,16821	115,222595	69,0283	53,61175	55,88739	59,13485	106,67144	90,108325
ID2094921		119,648145	134,73199	117,65371	115,446685	98,94297	57,548145	132,853285	116,648275	124,57756	157,37972	102,162625	72,633565
ID182136		60,719555	101,088775	138,20964	148,17739	151,797695	134,14895	79,737685	50,44434	52,204415	80,7496	75,10524	110,300915
ID1542		0,162955	0	0,283825	216,862855	211,91494	263,645595	0,940635	1,16339	5,348255	94,53905	60,319765	51,673405
ID855		104,85349	116,02353	121,90988	120,47166	107,610535	102,711065	84,085365	81,728225	101,857375	16,808745	86,999655	96,203705
ID3763676		106,658735	127,56761	98,185085	172,484005	138,64554	109,5893	114,058985	128,119255	163,10038	69,30179	66,1028	36,863245
ID613		109,30221	117,636025	115,428135	159,670695	147,602805	130,14685	20,383165	35,41969	53,68135	28,46991	16,280185	39,53792
ID948		69,80362	85,11734	77,08213	114,702925	107,1164	93,867505	77,619115	67,331455	67,14692	64,25744	74,932865	68,73748
ID1795		36,619975	38,32287	43,123255	70,48327	87,67998	117,42931	42,305155	64,57696	38,70106	86,98667	65,51389	70,700255
ID443		109,73064	99,176065	112,18984	83,344255	94,56566	125,045935	104,029635	109,17279	110,13557	117,82685	120,139055	101,58892
ID393893		99,75348	112,06361	101,98497	120,849525	118,50978	156,54393	67,21992	47,113635	44,11143	23,15296	33,316255	44,85242
ID1707		71,160045	64,31283	82,01623	284,97137	277,20566	667,558595	68,38147	67,913305	73,01447	7,9952	171,21148	113,73413
ID2439918		92,374925	98,54388	101,834995	102,9207	118,73859	88,819055	78,690675	67,842025	66,086955	139,29202	74,59456	72,73219
ID1100		115,64994	117,305765	122,467265	135,488015	113,594555	129,191935	165,197785	86,127985	73,639095	37,44959	72,45817	75,45592
ID216132		107,058665	105,8716	135,140515	103,39589	125,88449	163,916715	58,55061	66,89533	71,73375	72,77259	122,5029	138,961495
ID200032		57,504155	66,74027	64,653435	69,32628	83,575395	94,995905	70,11497	65,0583	83,19979	71,257	68,408335	59,85726
ID519		114,146125	125,621005	102,96131	145,82664	123,14665	146,809655	73,667895	65,106125	56,145225	61,51256	70,029615	70,85437
ID1609		122,00632	130,22599	136,249055	92,328045	108,8592	98,197625	172,55254	186,148445	184,239445	106,86075	77,045985	71,595755
ID1505		120,52237	134,07987	130,99778	132,744005	126,10774	120,17041	62,929245	71,30164	83,38152	105,63365	87,821635	92,325965
ID412		141,1698	93,149695	131,484575	72,14501	63,58292	78,157165	210,469185	214,082645	208,81785	198,67281	155,984185	106,131195
ID1763758		78,546805	73,584475	68,14913	163,8615	116,25375	113,340365	38,86905	22,168675	26,71627	27,67995	6,11086	16,19445
ID1057		118,49537	152,15813	147,66842	115,54432	102,133335	170,132465	45,69288	62,64501	67,145415	80,39359	142,57035	79,262065
ID1283		112,30181	129,105255	108,441575	132,161495	128,368025	118,95788	73,23148	74,707745	78,319795	102,20544	100,217955	89,12382
ID3483954		131,25986	97,005305	106,92226	75,616295	71,24265	67,054125	218,66685	241,14668	224,575515	187,96626	168,047835	125,310705
ID2024619		138,674675	195,98616	138,997905	450,726545	569,105705	182,34976	86,920865	69,223165	118,79651	72,10986	6,69303	32,734665
ID257339		94,703175	109,757075	102,7343	107,92634	120,49249	102,24559	56,76886	53,16074	62,59463	75,94552	68,71879	62,050015
ID313877		94,35545	91,46163	91,535945	118,90834	140,068845	123,55673	57,120545	49,276285	53,99212	35,86646	84,298075	98,021345
ID51068		95,15126	112,502725	97,838955	114,86235	139,15171	112,04902	57,767455	53,40309	66,197935	77,61793	73,429535	64,38711
ID2944561		100,00936	127,351465	86,090155	122,686505	83,29941	50,11967	40,60685	47,318745	56,83289	99,79661	96,81073	58,92048
ID3763623		106,13919	100,49331	110,66636	102,863515	93,16204	88,505715	141,03194	144,33797	135,10668	132,18857	117,23904	92,576375
ID17707		96,412185	82,091935	86,95306	76,985775	69,642465	114,65016	129,005445	246,601	149,730335	183,686	150,73024	124,97325
ID283779		87,88794	97,550505	94,94522	109,08693	98,701495	112,663165	69,348475	54,5822	66,645405	76,29034	58,69218	58,414935
ID338		81,08227	88,085045	77,97247	156,52138	451,81225	n.d.	61,23345	104,90754	135,71778	1,99128	30,52583	16,78423
ID3477702		111,978395	71,690085	87,04425	49,4565	38,559985	44,54373	191,63158	168,36276	183,583535	191,55305	176,415015	118,192085
ID525		88,803885	92,67589	88,03062	141,29972	136,635955	152,50753	50,093375	47,60587	47,220035	46,739	76,987545	75,02778
ID1980208		174,05123	115,82479	113,874775	119,09187	161,4023	122,167165	194,39071	151,58273	180,106145	14,275	156,95651	58,886415
ID1299		10,616345	10,599385	35,850255	545,8594	734,796845	203,71603	23,963795	7,941545	6,48767	22,9532	186,276095	72,546615
ID1523		90,836055	94,51028	104,679625	103,385295	88,1907	70,6622	74,816885	64,624135	72,56329	131,59305	90,953355	84,26218
ID1437		84,079715	92,7382	85,096635	111,239645	102,97431	106,869525	61,996445	62,61052	70,586	70,1053	72,94573	54,237795
ID464690		59,52291	63,30806	58,49845	107,7162	106,19204	133,616755	41,19211	35,62213	40,440925	22,38541	60,89979	73,700835
ID2060826		108,12546	120,415515	100,34692	155,54249	133,544085	219,34177	75,95439	68,896465	67,236175	108,47096	80,269145	79,59704
ID1414		89,36145	91,126605	97,95388	94,540145	109,70883	121,051995	75,891565	72,165225	90,654075	108,21636	80,84028	87,601915
ID2089255		110,24791	118,66529	112,006315	112,554995	110,794145	72,62658	116,965035	113,967565	119,642735	79,61286	86,41638	75,431165
ID484		96,09296	109,29485	96,478975	123,88156	105,891635	115,574275	57,05688	48,79563	50,279525	66,89533	72,044105	78,87754
ID169541		98,871295	110,523485	99,377905	128,68613	103,55603	93,278045	112,09222	90,384795	87,665515	106,56675	55,534335	47,618765
ID1327		408,41921	1297,18522	0,211435	687,88032	1764,04447	482,017065	373,948275	117,10928	733,33164	4,76075	0	0
ID3762278		139,71569	153,547295	161,03456	160,591755	144,31537	175,032515	145,719795	155,272975	121,410745	234,24386	152,664215	126,256775
ID229688		105,756225	153,441915	110,040395	119,23958	98,03825	85,491135	133,296825	115,476755	95,988315	100,22624	116,553355	107,72219
ID30544		114,74408	100,90717	124,44036	128,15279	111,34775	144,32885	120,273955	105,1175	121,459025	94,38579	103,707845	99,306775
ID411894		79,76005	97,58263	86,079835	99,44222	81,28203	101,42455	85,01741	64,14461	63,51668	58,85813	70,52642	79,685575

Table S-1

ID[1]	Protein[2]	Median of normalized volumes[3]											
		co_0 min	co_30 min	co_60 min	co_24 h	co_48 h	co_72 h	PI_0 min	PI_30 min	PI_60 min	PI_24 h	PI_48 h	PI_72 h
ID2308652		113,417885	107,845875	121,572935	150,54395	190,53595	157,25173	87,548255	65,68559	65,5301	35,40971	66,833355	61,22512
ID1000		95,990875	128,172155	93,382295	175,547295	170,825405	99,957335	36,86039	33,05804	50,327515	70,70423	71,374525	81,526345
ID722		119,89624	79,129405	97,965355	52,344305	42,95783	46,925515	197,81941	168,5571	174,932655	198,3249	179,56002	127,948705
ID1989367		118,33517	109,556705	125,90003	90,95702	204,66582	183,5659	177,75638	191,79671	200,911265	147,90195	200,792595	346,410115
ID2889441		13,65284	18,584975	12,483565	53,31529	35,50877	62,19249	54,477245	53,851385	54,072455	72,96074	55,8643	47,32105
ID339		100,499345	111,91526	102,24177	122,13104	140,458105	112,46349	93,67638	89,130565	74,626595	58,81371	83,27151	73,67042
ID83942		107,646215	125,850575	117,598065	101,173875	91,69005	86,621805	67,76375	76,120515	83,627955	61,98	59,275885	58,270715
ID157378		72,213525	76,99236	65,11875	123,517765	100,109835	75,86909	79,612035	69,1428	67,49497	81,53301	69,42125	71,116965
ID2617667		81,19187	83,76411	93,154375	113,7421	192,995575	108,07208	68,66319	97,557	74,68417	103,33284	83,539675	106,77857
ID1617		191,018045	234,79487	262,14189	120,814735	105,081765	115,559525	68,019525	111,42292	142,83006	17,65867	29,169865	28,39733
ID75108		242,630185	299,740795	186,91907	198,396335	166,36731	130,5464	189,36652	252,32276	267,08337	137,19248	138,74396	173,568105
ID388		99,729385	111,406395	96,14418	152,159845	174,022665	178,180825	66,100015	38,54121	27,60137	17,99027	37,73583	38,61153
ID303936		72,74235	65,936595	74,146555	90,22996	93,07594	108,740135	49,99362	54,97748	55,395755	128,84967	105,962175	101,16285
ID403		116,723745	131,53012	115,299385	163,759755	157,6365	151,140495	61,804905	48,80174	44,90327	0	70,03968	71,43442
ID1212		71,6903	183,56593	29,74337	101,6711	33,659775	60,81902	280,56535	1410,96983	184,70646	151,94387	144,29118	144,692025
ID97395		120,87866	105,38471	125,754345	98,32714	98,424595	93,440705	119,297465	140,64678	118,040395	119,79238	118,822305	100,993015
ID483852		97,109585	88,65739	89,839485	109,06343	94,286975	107,07189	99,269005	108,81563	98,29178	95,45093	138,5642	113,82699
ID2042668		99,798995	109,5632	91,437445	120,74907	131,595145	172,34491	86,328725	70,170625	83,2387	33,05122	82,49514	75,83708
ID1762		104,83737	132,74045	114,315325	133,66884	116,3255	113,11707	123,18382	109,480275	107,03903	81,76005	75,810995	72,77433
ID129210		100,764365	108,329405	95,036535	110,798325	109,79579	107,290325	66,002365	72,910455	67,26285	112,07605	88,357255	73,51552
ID423		124,313925	101,54507	120,00813	83,659695	86,293095	90,89053	150,612535	173,00208	165,81178	152,69159	136,421145	102,292545
ID110384		139,798265	169,139745	149,12106	190,202535	113,534985	74,127005	70,02823	60,463355	68,311275	72,42447	71,34397	45,033645
ID3046761		71,10044	76,48113	84,52904	86,38091	76,00527	56,174975	61,31531	73,127605	73,75822	64,52745	41,237565	40,15835
ID1178		81,12985	83,97805	119,78399	67,78683	60,57296	44,26125	85,52406	186,96429	89,0309	72,72006	166,956525	53,69828
ID85707		102,032025	112,278385	107,792365	110,463885	119,081175	120,44338	85,65261	80,854125	86,4756	85,78351	80,789555	84,03698
ID245554		92,90146	140,70943	97,17048	334,13569	115,70277	96,735685	32,60438	29,46911	48,887685	86,30065	207,02777	180,57468
ID1821608		123,623035	114,65175	125,837805	112,285545	58,089115	102,840585	144,83431	149,711665	151,368715	190,53794	82,820085	89,77911
ID23217		99,563525	109,086855	100,63961	144,746065	130,89409	142,744865	63,74182	51,55476	56,38622	53,13143	71,75488	76,432235
ID412768		91,95298	104,73627	96,892115	122,788845	106,81926	147,694895	78,730925	46,010595	48,713875	57,76836	71,84267	75,59148
ID3763670		6,15666	3,126245	1,4085	86,707135	30,714525	91,10262	3,406925	0	0	18,6275	19,066785	22,422705
ID132415		135,847235	83,005205	133,728135	44,476515	43,52462	33,90828	293,211725	336,78432	326,234625	184,61358	184,253405	123,213035
ID1543		150,178135	47,83824	22,144245	170,077925	289,019135	292,408905	24,644275	2,843475	12,37859	6,93089	26,157595	9,961305
ID16834		92,858575	105,9707	106,2982	135,76404	123,181725	127,687845	46,54601	53,85108	62,47343	80,39854	83,92065	83,926165
ID145564		90,48723	81,95474	96,21033	128,995425	104,570915	121,702435	68,754005	58,45473	52,96208	87,89668	80,779585	93,911235
ID1082		5,832335	10,574655	14,711885	99,762895	75,33068	107,69725	48,62756	41,895905	37,999075	127,00698	70,81557	75,604375
ID319		92,787515	98,517865	85,485705	184,15972	151,748945	133,173385	49,48551	43,281305	23,266195	45,46134	0	18,505805
ID66993		43,295785	49,561575	47,617235	77,325625	66,561585	65,568395	36,51888	33,482135	40,81499	60,27496	68,92942	67,626525
ID437		115,8849	103,55278	116,876465	89,591495	87,49445	103,895275	122,92308	142,50286	132,94414	141,57661	137,5722	105,508575
ID2194613		113,743605	138,379535	124,925105	175,34911	117,864485	96,69673	66,970285	41,34032	62,66765	28,98478	16,40252	19,479865
ID2140633		115,49914	104,785045	127,533995	160,721985	150,43366	139,06072	66,78717	56,448205	93,571345	14,09545	79,001605	6,749065
ID1340		96,10713	133,68583	121,027065	99,036485	136,669	100,007715	136,00622	90,35937	86,46295	198,45272	94,57157	109,172355
ID1993029		113,259375	126,611725	125,630225	160,618215	500,94151	149,773015	106,799245	91,665665	103,21757	203,82226	n.d.	n.d.
ID15367		76,084465	95,46457	80,431885	80,673545	36,5609	70,351155	67,33845	74,16664	58,913115	16,74382	7,63457	8,93526
ID5909		68,397905	69,202685	74,370685	93,96609	102,90098	128,00623	71,811395	36,207915	41,46177	29,61348	52,716795	55,95523
ID3184881		132,98616	145,6626	137,1061	108,88398	70,428705	99,89513	93,298875	102,08789	100,10281	114,40052	145,072325	97,877785
ID2302886		95,41636	103,11105	102,258155	122,23338	120,181905	94,715805	65,2971	66,93409	76,146155	71,47992	11,86405	175,89909
ID265686		196,075685	141,552485	232,09298	97,209535	83,252115	49,240805	289,58059	348,45686	313,2265	178,66838	207,518915	170,999665
ID3499167		109,49316	114,896825	129,4829	337,76658	295,148395	715,64671	154,50742	172,40353	165,43831	0	0	0
ID1230		74,94643	76,85744	80,916015	93,875655	74,925665	84,235705	102,17269	107,96794	100,515875	94,86051	93,766885	96,507285
ID535941		110,16886	117,94017	112,658305	103,91939	117,02335	124,33336	98,506095	97,58023	105,02662	135,54466	158,007255	133,794935
ID2021577		103,103415	114,908875	105,071595	110,17457	105,7323	110,56227	79,96107	82,223845	89,953285	97,20933	69,847335	68,604715
ID416		129,61369	83,20653	122,78051	86,027085	64,42794	86,744165	218,643285	234,27919	221,21724	174,10507	220,711565	111,58732
ID674		66,060645	69,616755	71,707455	109,34764	95,3999	109,232255	54,22444	52,99364	58,829355	139,48084	75,565075	84,74179
ID1298		102,340315	109,718985	111,40939	132,82026	137,316495	124,53328	99,40892	87,72809	100,653555	11,14585	95,8318	94,209035
ID1717		83,526945	113,789685	95,613725	102,197435	83,33527	66,12385	57,701585	91,06999	67,65924	21,19669	34,38391	37,336695
ID2447768		135,46063	111,837215	96,370225	65,82755	86,11389	193,907585	80,981185	92,555105	78,89927	89,19382	87,88066	99,8512
ID562964		102,68908	101,72072	105,55125	107,626575	90,485285	118,22309	102,69778	117,841575	108,29101	128,40529	115,55477	95,62231

Table S-1

ID[1]	Protein[2]	Median of normalized volumes[3]											
		co_0 min	co_30 min	co_60 min	co_24 h	co_48 h	co_72 h	PI_0 min	PI_30 min	PI_60 min	PI_24 h	PI_48 h	PI_72 h
ID296837		121,485325	132,38098	138,048165	131,05493	134,14959	144,661265	87,886165	94,247715	89,85351	127,18764	96,86914	107,92445
ID352255		99,155925	79,97076	100,2375	84,22372	63,22364	58,90475	174,28355	205,25715	186,005595	146,1439	157,521015	134,269695
ID1439		93,841245	99,536625	101,307475	98,487875	101,67295	100,01692	100,771325	125,015935	107,019625	114,51049	91,482875	90,745825
ID1709		95,942395	106,878415	94,429415	120,561455	118,306345	119,581335	63,687175	64,545475	75,119375	78,71439	74,053735	75,1848
ID747		127,68576	85,44531	101,818465	55,489505	46,69726	48,593285	206,93763	200,753595	195,748445	204,99229	184,15927	132,70701
ID311244		71,124695	68,87837	72,220415	80,726565	62,765015	92,513005	45,550165	37,020215	39,83151	39,39977	57,61546	57,00398
ID1124		97,936945	193,83753	143,48444	174,586675	126,537845	144,4837	66,180265	70,8032	80,496425	107,80558	103,50847	128,22544
ID3352574		179,464335	200,99637	174,59288	146,3633	135,426455	145,880725	132,584685	118,039235	111,959235	88,68243	86,9678	99,197705
ID7106		54,872685	60,883025	57,25489	64,768585	63,74518	110,423095	58,962655	55,95504	61,74834	146,04922	87,36495	81,177145
ID466049		78,46424	93,164605	92,921865	135,91799	146,286085	161,046395	61,5975	44,536805	36,921595	0	58,23113	55,97565
ID3763800		141,1878	146,5268	168,60673	229,27321	489,894195	663,097245	128,028225	163,06857	165,76453	24,04339	97,79595	33,985975
ID482337		78,111155	79,793565	77,36456	104,759485	94,895755	107,118345	71,722475	66,379	70,69985	274,85099	85,15999	86,58473
ID1785181		113,97463	113,705245	116,138995	175,297335	141,91577	141,525225	42,323465	8,08334	60,01083	27,56462	29,634655	5,24409
ID43156		148,798185	94,292065	143,51259	42,821555	53,87085	48,207505	302,05952	408,70271	335,76833	242,36858	205,01356	141,457885
ID1631		103,359555	122,731765	97,544175	140,947015	136,37136	115,40547	69,41004	59,33449	84,837925	81,97167	92,91114	99,72205
ID748		133,75644	89,325455	107,18966	56,403045	49,253855	50,196665	228,826505	229,73712	224,591065	207,25137	189,50912	136,33873
ID109442		128,95946	182,937135	121,536335	208,684395	85,376465	76,80189	90,57233	63,24082	90,266305	146,36363	67,808915	49,367915
ID1964508		95,392435	107,64023	101,152015	190,3299	203,0251	118,506485	98,964125	86,71229	87,744235	140,93841	183,51852	348,416685
ID337908		101,67667	82,347765	100,61099	94,81932	36,994085	48,322735	138,162105	401,63551	186,87428	209,15083	124,28319	97,132095
ID91144		77,577135	84,403745	79,1149	114,497025	110,672475	156,83609	49,972285	47,69467	53,834895	77,10786	86,219165	83,73022
ID3761685		45,96661	46,65017	43,090195	77,654795	69,98484	68,684035	64,10932	39,56289	68,81837	101,68188	71,9825	53,02381
ID2215889		99,49995	110,17998	110,46249	139,689875	106,466705	131,047275	102,11382	95,212275	87,02183	54,37943	59,38687	64,047125
ID135817		119,48953	109,586895	163,217965	207,16896	147,32437	71,79798	258,783525	409,074365	307,055745	150,374	120,11914	80,982345
ID2575		83,942785	96,94598	94,170795	117,676165	120,176475	112,370705	73,95932	72,21492	82,57299	115,95596	99,590145	89,78905
ID733		69,370025	92,947145	31,104135	131,016855	52,915885	95,21994	117,927185	97,468825	94,60762	23,79307	57,64783	43,35517
ID1601		107,07865	142,53638	98,611425	205,364795	286,492075	216,28352	n.d.	59,48551	9168,95591	57,79218	9,50059	1,159135
ID160578		n.d.	472,01062	24264,69615	122,2863	166,98304	115,26689	123,00083	118,50005	142,86253	115,9354	118,011315	133,08858
ID1464		96,431105	87,97291	167,084165	199,277715	84,637195	64,689435	61,339445	208,784215	117,4721	44,29028	85,72295	152,06338
ID2051821		91,23976	102,339305	83,22725	167,39694	134,211625	121,429525	59,0512	59,034945	54,9479	171,6132	83,00392	74,26399
ID2292906		93,092515	102,845565	93,53902	139,45205	98,968705	71,9136	65,822845	73,909095	68,348795	97,76356	47,743795	48,03282
ID2113599		68,6378	81,121855	69,774225	95,18813	107,65805	139,2689	49,32419	43,851365	29,86131	41,20719	43,52449	55,877305
ID1799192		133,127785	108,69694	126,463705	103,911035	84,48745	83,381485	217,46371	351,602815	226,290025	241,60813	165,76543	60,156735
ID1182		110,46495	88,58362	121,657455	122,730275	136,24451	138,5552	60,850845	54,726255	52,74407	54,58602	54,378305	66,14087
ID500		61,943495	57,270205	56,85363	114,708395	125,02718	175,81731	37,8281	70,31466	48,16008	71,25701	58,576355	77,17108
ID3762592		79,8157	85,04744	89,836335	109,28763	119,094405	136,80685	145,26007	129,520995	118,365165	97,80786	104,50191	118,060055
ID589630		73,4083	74,76779	72,54069	120,227115	122,39068	133,214615	55,02316	41,353695	58,519445	0	71,172225	71,182265
ID233300		48,4171	59,64703	43,11795	119,36665	100,247765	92,810995	45,62118	18,37495	33,343165	101,13292	80,56721	84,814515
ID1125		128,60459	124,544455	155,47471	146,681885	133,516625	137,76168	88,14703	72,624125	74,162975	77,04886	89,40761	99,406205
ID1410		150,47977	162,92156	197,99021	116,721415	114,147955	117,6944	143,186655	243,07556	153,040715	83,17646	56,563355	51,82906
ID2305694		103,888125	152,79827	103,72685	120,59264	110,253505	110,473845	109,2442	110,54522	92,46037	96,18981	106,376545	87,286075
ID1933424		75,884965	80,71388	71,622055	89,926165	217,77776	181,69079	84,274615	64,39741	77,49055	79,55715	163,706215	492,06831
ID196961		65,41883	66,39856	66,538235	112,846005	105,781075	133,14146	53,168915	62,053965	49,177655	64,57115	69,18858	77,62957
ID1721		855,32587	1132,87762	392,49152	112,674615	73,70603	24,19437	1351,253115	14010,96446	875,98422	66,29721	125,264885	94,492565
ID3170569		71,63974	77,052295	80,58867	70,231235	88,01424	103,69971	118,91263	114,011665	115,19002	81,44196	75,028525	88,96313
ID1577		64,51905	67,91477	61,533825	174,77498	102,629625	123,99583	82,832665	46,196665	58,02591	172,08555	106,022895	100,13943
ID3763666		129,32731	127,001115	129,361385	103,1266	108,37119	117,521765	71,343125	67,251575	66,93323	52,01199	58,30262	65,708735
ID1580		100,446195	119,69484	112,45448	104,2439	101,60399	90,000655	92,129385	90,09551	102,700215	98,93	88,47954	80,19711
ID156308		92,831475	98,68756	107,029135	149,069795	116,210815	98,76728	84,14302	82,524925	94,79875	56,94023	73,20246	68,715625
ID566208		66,43684	70,99835	72,262655	110,14034	107,75277	108,84258	53,25413	44,29479	43,17347	58,47637	76,824515	72,902155
ID3413662		61,14072	61,311955	92,527435	97,088405	106,539315	161,80894	113,826695	123,18157	94,358985	98,38011	89,92003	78,789065
ID238040		135,10938	103,04067	121,85885	90,034475	84,95553	94,47865	182,057485	224,620965	193,870375	169,11635	163,69795	124,08009
ID452		90,838295	98,936525	91,923815	120,326445	121,12478	125,466415	39,40416	43,75539	39,041245	5,53274	70,184895	76,06747
ID3761658		107,14084	92,046145	64,70022	181,34114	154,72498	129,093815	24,09295	70,19384	41,492035	70,48171	69,97218	74,91209
ID511		105,71601	106,290995	112,84967	107,692465	125,45618	129,261625	71,69093	53,167045	65,11757	0	56,19328	56,580615
ID3763684		66,791745	72,039005	66,10342	77,44121	85,28793	106,839865	53,44554	69,298285	59,160035	65,37855	65,56417	65,429255
ID1313		101,828095	118,00667	109,4203	200,61873	134,80007	123,741315	118,199565	153,501955	158,07491	23,59595	15,71686	1,878135
ID3763682		51,718405	72,0416	38,86039	92,06026	67,652735	103,21792	48,400835	44,39718	29,27014	118,89464	88,50082	81,036415

Table S-1

ID[1]	Protein[2]	Median of normalized volumes[3]											
		co_0 min	co_30 min	co_60 min	co_24 h	co_48 h	co_72 h	PI_0 min	PI_30 min	PI_60 min	PI_24 h	PI_48 h	PI_72 h
ID1457		82,11244	87,443935	92,67744	90,649645	92,03233	100,04342	53,754745	56,009405	59,77399	61,7965	65,726295	69,874865
ID1521		78,772685	57,2443	88,52847	127,344035	125,26145	110,17496	20,70696	25,93722	38,783065	93,82919	95,11329	88,85915
ID1840030		144,24791	97,4895	150,85411	76,85299	70,354515	37,12203	180,95817	172,592765	196,66067	138,19548	170,853915	327,319515
ID916		131,39893	92,691115	109,62628	56,29047	49,77842	51,11882	256,647455	256,908525	237,760805	216,71157	183,29261	137,446095
ID985		94,46466	109,603655	115,89485	151,68825	162,96622	125,767375	52,909475	31,49838	43,9029	55,39831	79,76176	76,744205
ID364919		132,83325	150,24889	127,539705	107,08499	109,4364	117,152855	80,699915	122,080335	84,0183	77,13706	61,94457	56,250755
ID1229		116,88947	121,043725	106,542445	162,530685	145,11906	119,678305	116,354805	105,46715	108,51483	94,66388	102,52344	118,768065
ID294218		94,84608	103,090455	99,160245	106,631675	120,690475	89,33824	71,66933	75,745445	87,49138	73,03408	72,588115	73,12111
ID1506		127,599415	141,14517	134,98471	141,40721	125,9372	118,96906	75,692585	96,503075	101,618645	91,75474	102,740445	115,57174
ID1481		75,611935	85,046405	77,288425	101,7385	91,950195	104,38641	48,611135	32,276925	60,815045	38,62833	89,549875	81,743945
ID1655		76,065175	74,614835	66,36098	184,705685	227,63113	148,247085	53,368675	55,205745	59,21392	363,04344	201,618145	152,96869
ID66151		25,69484	31,602605	39,318725	56,400905	72,92018	47,149505	29,24491	23,8187	26,644485	92,65449	100,445305	132,36153
ID168476		112,914925	166,952335	169,08426	176,137685	165,47811	141,30391	68,8124	49,28527	73,34026	82,62124	70,560415	102,029775
ID9044		118,72223	98,36306	115,9029	79,914685	79,28156	90,946615	143,35212	164,874345	155,770205	154,20459	139,08662	102,05525
ID2737054		81,953845	77,09723	88,006105	124,67923	34,429245	135,628825	52,03087	16,79496	24,49336	3,40788	23,78227	17,48785
ID24906		108,27368	128,758695	114,28871	161,899015	130,67395	110,69691	26,860545	24,24073	5,63864	5,9796	72,3672	4,62173
ID594301		81,2547	91,487605	83,08087	100,025985	91,09497	86,964505	77,405645	61,690055	75,326765	178,93048	91,50274	95,14092
ID3711681		120,030335	139,24256	122,21746	201,449025	138,902755	90,007925	92,38766	89,457095	82,539255	99,06952	78,392645	92,07796
ID2201032		86,44849	95,22476	90,745845	165,04647	601,728505	3213,762765	45,68449	25,116925	43,497755	1,65398	0	0
ID35204		83,840135	94,66872	84,420395	111,43024	106,967755	109,56515	75,063105	77,98403	89,564985	24,99451	84,985795	103,322425
ID1366		109,4484	130,333455	114,55823	135,90606	150,08756	138,627835	47,359685	23,714195	41,242445	43,82576	105,43213	132,43606
ID731		98,309985	86,042125	95,71367	104,549355	117,01693	99,91961	155,036985	243,931515	156,614205	165,28379	131,642545	44,92325
ID3668269		161,73751	172,663405	157,494885	64,10562	70,46584	89,38207	223,4139	194,67684	197,59984	37,20796	40,54024	30,673345
ID2294		85,466985	94,199075	78,742615	117,397395	104,6506	98,862315	68,996225	53,713605	69,55166	77,05297	82,669505	91,407985
ID1750		98,647565	98,535575	116,162475	77,637345	93,36606	91,75937	77,48461	70,214965	79,0953	69,4789	52,916465	52,84791
ID74132		91,97911	88,039095	122,93519	43,733525	72,423015	70,19386	104,242125	109,449765	118,70395	124,23703	69,396495	62,979525
ID4476		147,80489	151,971805	140,9886	155,0355	142,54781	137,482485	70,70755	64,91488	72,966425	89,33659	85,080375	87,369995
ID593		87,657275	89,189955	92,067365	116,27822	112,77129	121,006915	89,52552	92,06114	95,22314	84,63855	95,24483	89,90564
ID1556		272,20008	423,22454	660,714015	128,393505	100,73591	125,997635	278,829455	227,708105	192,008525	45,99612	75,283155	64,641195
ID759		151,200665	103,36883	125,32549	60,29007	54,28573	56,87881	253,232255	273,060995	264,8523	209,04462	185,26306	137,04818
ID1785527		110,6467	116,59652	116,028205	159,376325	153,56118	144,99496	62,11275	83,67448	76,601775	0,59013	100,148325	10,59052
ID185509		88,56423	100,361785	89,153825	124,1525	114,82702	139,271695	159,823755	204,63616	162,63817	94,90073	80,52794	77,339235
ID2005865		129,546495	147,541885	128,67454	112,26544	177,522675	128,947185	73,44448	69,944335	107,60402	77,02589	93,389595	116,142855
ID1759		89,285245	101,151835	97,09485	113,88103	100,65983	107,27651	72,35317	67,65373	65,16061	90,91395	83,98586	86,703835
ID71445		38,13437	50,87422	50,36578	54,12216	40,935835	45,874305	43,55021	57,303385	65,288405	210,01074	113,00752	126,826125
ID1236		2371,17663	n.d.	11231,5328	109,497575	197,85888	86,247695	2816,11016	28906,63011	n.d.	116,4369	79,120625	131,93148
ID406		105,880965	88,210605	102,08926	100,875905	80,05224	85,412685	138,12899	121,605145	130,92691	124,92737	125,26962	99,997145
ID858		126,535015	95,952705	115,402665	68,711205	62,73355	60,739855	212,54911	247,74472	223,40364	185,2409	166,30984	124,110595
ID1761		34,41894	38,660525	52,130055	52,91507	58,27225	121,90206	46,47426	114,35039	54,07819	116,59343	72,36987	72,29631
ID756		134,3661	85,106795	131,16695	59,60169	55,62887	50,51894	249,000315	279,144975	261,20581	167,88395	159,713505	118,15554
ID371		90,57313	87,671105	88,78887	111,30061	105,691575	118,28221	79,99038	68,625305	69,8609	53,72891	81,6347	69,3212
ID2102478		97,95643	105,271905	100,237065	41,45628	110,88257	94,31956	78,433485	95,814345	97,346435	111,88615	60,11122	67,839535
ID324496		116,6636	107,825695	122,681665	127,94874	136,78097	116,010565	154,28502	125,28152	124,329305	89,10954	99,141075	93,2424
ID246907		101,80755	82,4137	97,55783	11,297955	75,01745	69,31852	104,178495	194,683475	74,08564	188,81791	200,864185	128,733385
ID325		100,32159	110,706495	98,999125	116,39557	108,024975	102,69863	98,510045	74,768415	101,096235	178,43926	98,957155	96,750125
ID338842		104,973885	109,69146	108,051595	153,989435	152,61154	138,71074	111,84289	110,34964	96,464965	139,39997	147,239285	102,846355
ID183880		160,30871	181,892915	225,224125	133,69185	145,07476	134,614405	2,30492	5,161635	3,50311	58,89315	81,03488	85,097395
ID1657		447,729905	270,407825	125,061855	142,13982	171,524645	96,679305	32,791195	27,76612	50,710685	42,44573	194,402595	17,518035
ID1608		67,14316	93,16734	96,608185	98,903925	90,67091	109,5135	90,027855	53,63402	62,85227	21,29585	66,006975	69,523345
ID848		112,91809	103,634815	111,719035	97,776385	94,68336	100,802705	122,798775	138,777835	130,897135	102,05145	98,45339	102,918325
ID1197		62,846075	70,33997	69,91661	125,66771	101,754255	119,34253	71,706	66,783575	62,39679	68,26144	80,28485	85,305055
ID362785		84,82085	95,28204	100,452355	150,2383	114,466575	126,07744	61,58533	51,394225	58,713965	91,68877	89,47785	94,603175
ID1025		137,864105	150,46406	154,947345	130,385635	126,649185	71,883355	67,032255	44,48427	75,84382	86,61891	82,03333	73,086495
ID240147		100,9846	119,307615	103,39284	163,30904	142,029615	167,476005	68,385325	46,99953	47,96812	25,50962	51,959135	55,76686
ID3761675		114,666065	114,818135	106,591675	136,148045	132,011475	119,12534	82,75516	95,53112	83,79276	77,77282	83,36393	80,78137
ID1987569		88,63417	61,63909	134,604595	329,03852	262,4465	127,147885	227,414125	451,547595	716,982825	95,86655	165,88026	457,409465
ID567		77,03523	86,073455	77,06341	96,30648	82,748825	100,156265	48,452225	56,3424	63,72085	86,8921	70,134415	85,793215

Table S-1

ID)[1]	Protein)[2]	Median of normalized volumes)[3]											
		co_0 min	co_30 min	co_60 min	co_24 h	co_48 h	co_72 h	PI_0 min	PI_30 min	PI_60 min	PI_24 h	PI_48 h	PI_72 h
ID2596060		144,086855	163,09586	120,92059	131,48389	110,54722	97,782795	147,28973	131,688835	140,568975	126,72762	123,672965	120,450805
ID1193		27,676625	31,214045	47,120505	69,34894	39,763505	35,284785	192,88311	64,67352	41,413235	15,32282	52,63813	63,85948
ID11367		97,683115	106,318325	98,680295	114,450265	133,933805	141,248975	93,92277	91,89896	96,9937	75,4953	78,2474	75,677715
ID2214019		104,05847	230,39511	133,69945	671,66564	904,18229	374,44238	n.d.	78,63115	60676,90806	0	0	5,845585
ID1615		63,866605	141,70284	43,612345	238,782975	325,008515	105,365375	19,19186	6,66334	22,79773	9,96687	0,236425	7,27908
ID1226		98,35295	109,32747	100,2037	129,15973	106,25779	105,823735	78,594405	73,63852	76,96984	114,9303	92,7103	93,547685
ID80360		99,26888	118,28936	136,777435	135,790315	151,707285	160,265655	73,94719	78,11658	90,51091	62,28571	82,165265	98,940365
ID732		103,999095	91,325465	92,778885	111,364485	114,5738	117,904395	158,85476	269,190515	176,42436	198,41249	154,16075	61,887075
ID1475		420,03199	280,86146	389,11258	123,206	175,804005	95,5199	973,016185	1000,327215	870,38995	192,20288	204,633405	151,83613
ID1461		74,18884	83,536855	79,380885	110,11706	96,959655	141,879345	69,401845	78,221085	82,26684	55,53976	95,275225	86,48192
ID3597623		114,299685	130,480735	130,026985	148,9523	139,08746	127,90507	66,23536	80,32578	83,76695	126,02197	98,7583	122,949045
ID119751		98,26515	107,367935	85,9921	116,56803	114,94243	142,7545	58,467105	65,85379	51,305015	64,8809	68,88814	76,226135
ID60178		58,07145	64,811035	55,61444	129,09059	96,88239	109,58384	62,388175	46,95787	56,93862	99,27879	85,749285	95,552275
ID1130		481,25687	385,521875	800,591535	344,86526	437,93313	166,58528	159,315715	90,13392	49,732425	57,65972	120,745285	213,527145
ID2838327		151,878425	165,71181	170,70167	178,234765	137,661235	110,78775	62,554265	61,88477	69,810045	104,80325	89,336035	101,714475
ID27152		108,59102	109,562405	142,90709	76,237655	104,68315	118,86967	87,83517	55,23929	81,874075	0	52,21811	47,42574
ID2085530		84,87864	92,397355	92,80298	128,09961	121,607265	164,20878	69,680905	34,752725	52,20244	29,89574	67,489605	48,60333
ID1669		112,02831	120,00794	130,740185	113,04134	123,598825	99,43038	65,156855	67,5415	84,302015	101,74027	92,294925	93,532125
ID3403522		97,10555	117,17602	119,557905	675,850135	89,377265	90,68382	426,510615	5054,344695	464,42233	127,69337	99,242505	99,90441
ID1915149		96,166535	62,914825	124,22338	163,9628	15,27455	102,908695	1433,553715	942,47299	1058,04259	70,74936	539,694515	139,30126
ID1582		85,2719	90,451795	64,39127	60,51584	54,293625	69,205735	50,903305	44,344135	62,858855	65,46446	97,66256	105,653615
ID2555205		118,36021	115,387035	186,967025	209,539855	400,1463	263,970735	80,53173	42,21012	71,147475	89,46959	104,191955	64,347015
ID124300		144,906115	116,56102	145,7474	84,19133	90,723005	96,71828	204,130945	303,833845	221,0927	259,82439	225,95421	172,134135
ID2723		120,201155	139,104225	130,256965	124,35953	115,835265	116,780545	69,050705	81,75841	87,24645	97,5881	88,639965	100,68902
ID504850		118,90933	128,954655	124,416245	119,51016	129,18007	119,600015	90,80264	99,80421	97,15779	114,99449	90,665815	85,647175
ID1665		128,08176	122,4768	168,127225	82,54846	83,423565	74,87669	94,308475	97,1834	91,383835	85,41282	63,595995	66,14709
ID1228		252,68093	255,561675	284,96908	116,49016	93,062835	74,327745	311,956285	177,009415	188,189495	131,17482	106,905635	125,12506
ID457100		86,267035	93,09254	91,61034	121,058405	119,578415	130,4324	82,9085	65,75439	69,434785	78,78106	77,254995	81,408985
ID237648		178,268415	105,8965	158,496665	79,117015	62,05137	115,25775	307,205385	325,987715	275,216285	246,28031	196,220765	144,23486
ID320752		101,59175	102,89746	107,808025	128,35555	117,700965	108,41019	119,544885	95,01007	90,07537	75,64958	85,210785	84,11531
ID1488		96,401785	107,4578	107,16046	121,52182	109,64132	115,7314	65,20824	79,918535	78,72027	82,40175	99,519545	99,063965
ID3763695		16,540625	25,059895	10,267545	46,609045	33,511025	18,19321	91,1996	33,32465	98,710815	92,24176	47,603355	46,373325
ID2286		96,90995	105,68605	102,74102	115,22865	106,191655	102,646175	86,600615	78,28975	85,24105	98,63396	87,84947	92,361715
ID114043		104,746975	124,78334	88,248445	110,971805	104,091275	139,288305	40,3165	43,846505	47,900185	36,95797	66,95763	75,380555
ID1800806		118,61783	115,405665	120,937675	158,148505	148,058195	114,3748	25,75982	353,10183	71,41217	0	0,88064	25,66809
ID522		109,69618	118,20915	119,50385	158,97095	163,827845	139,545975	45,30791	45,66676	55,242705	3,01244	34,27124	13,710195
ID1260		127,56261	141,54442	154,222455	135,30499	130,587405	138,875	127,49691	117,48538	111,984695	92,73742	89,842765	95,651815
ID1048		32,892305	62,8308	25,88817	111,000315	38,018805	97,505575	20,511425	20,85154	43,996885	18,33576	63,48758	49,53882
ID1575		114,05376	125,313195	125,329545	147,84669	116,2667	98,6336	84,26998	81,811205	88,809065	157,54345	121,715815	96,76976
ID82135		88,342695	102,083955	97,67905	150,71015	160,296485	120,282445	57,07427	52,99999	65,834435	115,82159	124,225155	135,63858
ID8503		65,4954	68,075475	74,164845	104,15178	98,339825	111,1898	56,41129	55,388875	48,31638	58,03264	63,041495	69,097035
ID2315592		81,65933	98,596955	89,86476	124,503965	99,12256	95,954285	93,513145	92,748435	86,277335	89,18351	89,227925	96,850945
ID2072345		77,491105	84,377335	74,946575	123,34206	119,995955	228,91364	60,992185	36,64791	35,30995	4,27317	51,928225	53,152295
ID776		109,213645	67,714875	105,920285	46,400665	47,477815	47,27422	200,094295	262,826545	216,239395	223,60162	212,81634	163,5151
ID444		104,711555	99,71068	106,689895	99,89583	101,85435	118,15932	102,522185	107,987005	103,06055	121,46846	125,035795	106,165665
ID3761695		11,679385	17,81424	10,66249	81,314565	40,020545	85,62982	41,80873	37,345485	48,792435	55,55077	56,584175	44,765195
ID2330		49,31228	52,645125	40,774395	102,49173	64,967325	98,43861	112,363815	114,48253	97,436565	83,25862	99,31982	161,236075
ID2183		132,986725	97,552555	120,704065	69,277325	60,482585	58,783885	231,248095	259,91344	240,3311	173,76531	169,06966	127,68015
ID2830369		140,743335	154,761755	142,85872	150,192855	125,194975	98,63802	80,144165	62,62128	80,873	102,86752	88,43157	94,10799
ID1705		154,25725	161,500885	157,233505	145,929875	143,178015	103,44416	119,78032	101,32146	121,388565	137,53088	183,43256	93,362615
ID1571		102,59895	116,29828	102,338565	138,80477	131,66483	126,76017	72,85282	69,731725	78,525785	84,40289	88,86066	85,659565
ID43845		102,70802	91,23302	96,361555	85,66004	90,87577	106,3902	153,567395	165,508645	168,509495	178,31559	104,222625	106,102855
ID1983973		148,64861	177,07821	164,387825	104,84455	130,66277	180,3859	168,710295	187,02289	222,25163	117,93709	97,37938	97,72935
ID1470		124,512705	103,516125	254,160785	172,31172	202,85783	188,223405	129,179835	195,042625	166,719865	23,65141	29,175065	13,14241
ID186642		68,982035	68,91716	57,28686	75,9829	83,368265	130,835005	60,328735	43,75006	50,05737	85,93743	67,20209	71,425285
ID3761869		81,159455	91,550315	70,737625	444,85219	227,02021	316,41022	90,348235	83,940795	101,977505	381,72711	n.d.	176,00786
ID1451		68,0005	73,158915	99,20316	163,71689	150,54518	236,35589	71,92395	152,11063	111,90247	14,5049	55,522875	3,043525

Table S-1

ID)1	Protein)2	Median of normalized volumes)3											
		co_0 min	co_30 min	co_60 min	co_24 h	co_48 h	co_72 h	PI_0 min	PI_30 min	PI_60 min	PI_24 h	PI_48 h	PI_72 h
ID262545		120,275805	132,477585	135,145465	96,9308	132,77885	93,56986	55,70883	37,779585	52,00463	59,20916	62,48303	61,21615
ID1998321		25,80449	26,951255	23,745655	93,502625	79,498425	109,60943	27,439045	16,24701	25,693285	29,04275	40,70773	33,41185
ID2087348		90,71811	103,44086	88,159365	111,624745	110,999395	78,154115	74,87742	70,24162	63,97605	25,73166	36,813355	45,876155
ID639		70,76658	79,0745	72,035095	125,57626	103,361065	100,890405	57,630015	59,176035	70,600665	89,40826	65,57534	82,908585
ID2055531		103,15029	110,16742	92,268045	159,08109	117,3333	123,02922	81,758995	66,86711	67,411365	155,41745	90,62614	78,04684
ID268600		25,788605	53,106695	46,097325	68,151945	42,27217	114,678415	150,693035	110,272215	198,23661	103,0694	46,372115	46,582045
ID2083630		94,84738	103,608265	101,125625	127,62404	133,255	157,43235	72,24056	55,381595	58,42789	23,23333	61,766895	53,474475
ID45562		123,83111	110,120575	121,67839	100,522435	92,323825	109,16747	184,117445	166,786035	162,1099	129,06183	131,793005	118,03617
ID1792282		115,913205	123,50017	125,431305	143,381095	144,1363	131,58238	64,0738	81,47582	78,73008	37,12842	51,034465	36,53574
ID2318312		87,24116	100,618205	67,07718	113,561	136,13482	237,274035	44,2854	82,616545	78,99709	89,88915	84,79872	91,244545
ID2880272		58,40824	75,221	62,99453	68,28188	86,056655	134,38691	195,568385	148,517725	163,575985	116,48816	75,620975	54,391735
ID3759858		90,697745	109,1491	75,325655	210,020325	285,360295	257,468465	72,32739	62,05013	75,15501	247,14215	44,35676	89,735715
ID769		143,47688	94,091295	140,01839	58,254465	53,305995	52,214925	247,042675	267,912925	258,182085	219,28288	193,701365	143,952785
ID221306		86,04593	111,068915	102,32477	106,16241	104,331025	95,06027	70,050165	79,25187	61,9731	120,06056	70,59148	71,787845
ID401246		103,324005	94,8642	106,92369	84,16179	62,354075	97,774135	100,781845	92,598975	129,28455	141,32418	97,372035	81,553365
ID57056		61,526995	70,66825	62,27633	89,84065	86,833895	79,695075	47,840085	39,128575	51,34106	62,4531	67,25757	70,352355
ID394		114,845525	94,332575	117,698695	74,86347	80,102285	97,668195	161,383285	190,13609	186,740585	157,16816	139,740735	111,314575
ID2437		40,504315	45,75968	48,386575	125,141295	125,57898	131,379555	55,438965	36,8967	98,43516	56,76696	77,46198	77,65944
ID444662		175,3716	179,63045	192,50107	153,703895	142,32495	115,32567	189,40868	180,41095	190,31474	543,56248	201,10852	217,481395
ID154183		91,095155	108,21531	101,21722	120,410615	135,29746	149,64882	50,75459	66,726815	64,60876	98,26682	119,145435	115,957305
ID1452		77,85469	89,75318	98,63359	109,00543	109,433975	109,970875	65,377735	65,871045	73,868515	89,81063	86,175335	86,70703
ID1600		130,1019	140,47274	164,8684	128,488195	103,402055	85,688355	132,28032	204,18777	173,61803	82,12549	66,468975	78,91421
ID218682		199,436875	212,762925	216,780795	162,17101	144,53205	161,71811	123,34514	93,86857	151,62269	78,54983	87,624695	85,478435
ID1866181		1513,53796	243,584425	111,47018	108,253365	1286,13716	156,813155	90,01925	185,85337	109,764395	41,04196	53,571495	0,01137
ID392		122,502435	101,912625	120,408565	87,60091	85,395965	103,211495	158,966315	184,52941	183,83564	161,78633	140,721895	107,104625
ID1654		102,53134	104,54464	108,586305	201,46277	205,962685	200,34818	130,086925	123,0941	134,57522	106,88181	162,0932	155,95723
ID2917416		128,476125	127,362565	138,17908	180,57991	169,44718	143,741275	28,675085	19,29086	31,922765	59,25728	151,905365	155,069485
ID149908		84,385765	94,889385	85,620765	107,431695	96,60769	107,999575	61,84535	60,730775	69,77199	72,67831	76,17095	81,6
ID2204207		127,903415	158,32853	162,71055	258,340155	653,97441	123,60653	68,67375	53,859125	66,151335	46,42129	1642,25764	71,655545
ID2068313		71,21509	73,88052	64,49273	116,893585	127,741025	154,364635	52,9749	28,671515	35,221845	25,89099	50,44453	52,5218
ID315205		83,4516	75,281865	77,91438	111,56854	123,19847	103,77212	72,864835	54,293115	61,15976	71,62785	75,41248	74,019635
ID2912567		92,84593	133,77259	118,840075	104,879095	124,51268	101,74395	211,8069	n.d.	n.d.	100,96616	128,85948	162,832495
ID1867746		105,01289	94,627525	119,83265	80,47935	62,141175	54,15455	107,573115	178,19904	114,077395	76,44881	175,40296	86,962865
ID1677		77,132035	95,89571	70,15815	112,98754	66,591985	86,599265	56,66474	0,15108	37,39739	526,86271	278,611625	260,84683
ID398		109,04361	112,29329	105,494985	131,25197	141,707835	146,71037	71,197755	63,0649	66,222535	76,86574	86,203435	85,11999
ID290046		72,643875	88,32034	73,130245	99,9602	94,051045	129,349355	109,59784	82,54667	77,296935	67,57772	105,30636	86,98285
ID996		60,14356	253,9599	78,74742	152,14776	220,037025	92,15265	118,45779	277,887995	180,82246	108,36936	102,87172	67,68099
ID3549612		97,163235	50,32229	79,239535	44,26357	52,1301	40,998365	263,82021	260,55422	244,047815	214,25201	213,306225	150,676215
ID2152149		77,80457	95,713685	75,80472	94,247825	97,387315	98,967215	121,13864	108,806385	111,90979	82,92413	90,690455	70,584965
ID327		99,845455	98,905095	100,59717	109,30698	102,15758	93,16255	111,61762	118,826075	100,03491	91,09022	99,689205	78,223625
ID1006		138,98092	318,41288	635,591275	397,225455	256,742845	137,302	62,23049	78,16834	43,101675	79,66433	100,677045	90,6779
ID232052		82,46885	70,66001	118,75207	70,6255	69,53761	48,23947	61,353365	3,2915	36,62968	95,11417	61,003845	45,11657
ID1574		232,724165	558,08563	1323,27202	37,395415	51,830215	48,4013	203,6002	351,59485	195,902265	20,25749	43,780635	84,17945
ID3763685		116,98944	108,31744	108,272715	137,00574	127,10337	83,10986	64,992205	86,23563	74,998785	97,73169	97,41028	94,543485
ID2093028		112,33839	117,551005	109,26759	106,7142	99,35778	56,257045	133,094835	127,222215	143,577475	111,34738	124,02691	72,837125
ID3464756		108,431405	117,47771	118,387215	89,63558	90,58471	51,10978	35,921915	0,513615	30,297725	28,80509	42,89911	68,07995
ID2997219		75,950625	76,91129	75,444595	87,67679	40,234725	37,731975	103,103325	164,254695	119,50514	46,76985	42,561695	26,290005
ID1716		59,211235	76,67799	68,630355	115,68466	114,22531	140,898675	57,57923	42,368145	55,24772	53,10884	77,97805	129,687855
ID523		115,7831	127,564535	111,532165	143,875625	124,38857	156,12105	85,811495	97,220895	78,76842	95,08377	79,180405	81,472525
ID775		126,574805	96,204105	122,450375	68,067835	71,479065	73,13959	191,680175	233,732205	175,95547	191,76918	171,13654	137,69205
ID350		89,34717	100,51646	90,639005	113,598005	117,298615	132,311075	76,08007	58,6121	61,937565	45,78811	65,179125	74,867995
ID3762281		133,891405	109,75618	124,77668	118,88022	105,844635	90,576955	151,00581	143,902185	159,73642	144,1955	118,364695	91,84111
ID463195		81,328945	88,248745	80,890565	113,99908	146,89869	135,799015	60,81427	48,98784	55,77702	4,83931	60,84448	68,665405
ID829		130,793375	149,52392	162,05109	136,086775	126,828875	177,45068	93,4883	105,306025	113,404155	120,37455	168,33673	129,243985
ID3261240		92,67639	103,85793	99,69121	121,43431	126,537865	132,77209	87,865575	90,50975	95,85824	66,00233	90,29572	87,242905
ID587		9,545935	10,0167	9,52973	29,34899	15,78262	80,67157	45,366735	46,78073	76,06348	61,81966	31,52208	49,42629
ID193389		60,539565	67,14238	55,58614	86,87066	89,311945	110,51362	89,347095	70,18502	82,493455	109,30981	82,755875	72,199885

Table S-1

ID)1	Protein)2	Median of normalized volumes)3											
		co_0 min	co_30 min	co_60 min	co_24 h	co_48 h	co_72 h	PI_0 min	PI_30 min	PI_60 min	PI_24 h	PI_48 h	PI_72 h
ID307		109,12255	107,08312	116,47629	233,956665	217,82503	119,24964	201,66453	146,32415	0	0	132,42756	37,47676
ID785		152,06667	100,98235	124,24116	55,97038	48,22547	52,77446	291,303975	298,33929	291,682895	211,67535	184,2662	134,60489
ID1105		123,280205	135,36302	125,41825	135,456675	146,2113	156,961005	37,057635	31,45618	51,52819	56,6596	89,723935	97,113865
ID2990527		235,20914	317,94567	196,16416	593,434585	380,66968	151,32295	346,82253	565,977	308,184275	0,37601	0,01906	0
ID240305		93,002405	109,19972	98,051975	146,76277	133,59583	135,64771	66,79451	50,0941	52,16943	24,55377	55,859725	58,209445
ID3259231		82,34446	90,16495	93,887395	98,11176	89,617995	92,790975	102,319075	102,133295	98,98266	72,06532	83,615035	86,53472
ID1814025		131,66847	124,093595	134,90647	243,214375	130,224115	122,066795	13149,26365	1117,053705	455,22864	804,48277	0	46,340435
ID2350362		77,612655	88,98751	79,176115	78,624575	93,17361	148,65775	75,75814	81,072595	78,55007	96,5064	68,61901	81,17721
ID2028269		74,199685	119,63748	69,84251	150,64091	93,867925	155,04361	21,233075	53,29015	45,83941	30,20786	39,64912	55,433205
ID2074209		97,8073	95,60887	102,442755	97,53061	100,95785	87,28879	121,25296	138,818675	115,72855	113,62947	101,64051	69,151325
ID1772		110,58201	127,47369	118,91154	118,783305	104,57731	97,150305	76,394975	72,45461	80,879225	110,92348	91,014705	94,810595
ID2218268		133,43701	145,631565	133,03421	294,73722	721,17673	63,909935	62,06691	68,583935	83,170635	0,30577	0	0,005025
ID4923		95,828875	107,069465	85,724525	136,404435	152,43765	136,223425	99,928435	58,595595	68,177025	63,64655	89,8759	82,76708
ID2033833		15,86412	8,03953	14,534715	31,412905	16,37726	41,56826	30,260985	19,189685	27,68712	70,08033	38,69845	25,11885
ID2037342		19,10624	16,782005	28,22797	50,717915	38,00465	124,98411	29,279425	30,86486	37,327755	53,79424	42,33107	57,72252
ID754		138,31833	83,573755	137,9919	58,049535	54,115015	41,44375	278,72857	328,273855	297,183415	179,3574	169,683595	121,14855
ID1026		92,40956	133,06908	98,033925	186,55443	187,784405	164,453765	140,862475	161,13155	164,355525	76,34495	61,66745	41,774035
ID210320		117,844005	124,957545	123,27741	102,69081	88,643415	99,091045	84,90953	78,98358	76,059695	78,79659	59,163375	67,262405
ID652		53,842075	55,62667	51,8136	86,15588	70,25813	74,371215	48,44822	44,058025	49,635835	90,60236	61,408955	77,084215
ID1843456		124,42194	87,247535	131,660115	248,171125	95,976015	18,118615	288,2531	246,602615	274,708765	89,3415	170,546785	145,04268
ID1564		115,89132	104,349165	582,758945	188,263395	204,570315	551,01865	32,814535	109,512485	99,837945	22,95144	50,6499	53,509835
ID413		143,076695	94,457045	136,12456	68,473725	61,97	65,117285	235,74247	243,26392	235,76106	207,8227	192,801815	109,051755
ID3040913		148,03133	142,40065	221,93033	118,187155	88,294475	20,27903	119,091095	123,02193	113,09136	73,47756	69,360105	63,111455
ID136093		77,59455	82,80186	81,88253	102,686505	91,5343	101,072045	55,230125	44,960795	45,33363	72,34207	64,81154	84,016005
ID2057395		111,542195	115,57679	99,52309	154,124325	143,52624	202,46863	74,291185	66,55675	63,917735	101,48805	81,99594	83,51848
ID2062951		91,19294	97,31949	85,18465	180,13894	156,49485	217,018005	48,06276	33,720955	42,74015	23,87556	36,97325	37,316175
ID438739		38,464565	51,908405	35,071165	60,449775	51,936885	83,76843	34,38723	44,037405	39,589835	47,13885	49,7032	50,738515
ID578		114,516405	110,516015	129,056265	109,11656	144,87778	137,560305	291,481105	352,330475	392,974145	111,71082	117,877835	99,32337
ID25932		97,35746	99,559625	88,305265	184,485235	169,05112	121,72292	27,65185	16,85235	24,835555	26,06764	44,90586	43,261
ID2105992		100,019025	107,514035	95,54478	130,28088	128,04316	151,79092	53,951755	29,692375	38,33108	18,78976	28,368525	35,53487
ID1804168		28,479055	33,84209	40,338515	22,34771	15,17307	19,95668	804,99368	0	n.d.	41,80688	35,451385	62,517155
ID2202941		12,23298	15,9992	10,03883	63,04164	40,63658	50,724345	98,764585	93,583935	99,7761	29,47598	49,776145	24,388085
ID1793947		101,54421	114,11316	115,4886	149,03885	124,81562	133,93915	59,04356	92,68904	86,08973	74,38409	84,313235	70,910515
ID3763473		35,72331	28,81882	52,878535	128,218865	149,66035	149,39231	96,649175	493,673825	196,51612	77,02407	219,96305	157,505175
ID470		60,91154	76,85696	57,84029	100,93871	109,40541	136,514065	49,820245	59,91333	52,90483	86,3767	91,445155	83,22858
ID1411		65,276685	66,64628	71,175705	81,260355	95,861015	104,88904	57,250405	48,05211	59,14893	78,24356	77,2931	76,10549
ID1532		531,73463	283,129805	652,35658	190,633535	158,351525	829,28714	476,724135	597,075465	536,92003	10,30956	60,322435	63,71678
ID963		73,735185	81,380905	73,425435	76,81312	85,821975	99,91754	78,41656	74,681365	79,862455	87,37791	76,49249	81,307875
ID1819121		129,330485	115,99883	129,19581	108,246295	61,88556	106,447635	126,790615	125,044075	149,80069	280,99527	93,8817	83,06582
ID7425		106,744605	84,757555	106,72511	80,83984	64,744515	94,55462	193,34158	227,45984	190,61004	157,19446	136,005785	105,7358
ID36784		109,1476	122,86271	108,110275	147,86142	142,40074	135,934055	44,375665	29,178465	15,572425	1,7262	65,687915	70,40649
ID2091139		113,45493	117,6113	108,51645	107,92336	98,11152	37,310335	129,34547	141,680025	134,284695	68,1097	93,202585	62,34366
ID1735		112,947465	125,95077	140,216715	137,63251	150,83428	132,576855	97,15208	97,487715	96,46718	97,83675	119,81013	127,870375
ID2076081		96,808085	92,51319	97,2917	95,698065	97,64192	63,35633	115,87363	137,46666	111,030575	110,2173	96,21255	80,26701
ID2190755		120,26998	137,988005	125,33933	216,456295	269,917665	176,525735	42,64605	59,98982	58,41406	56,88017	37,743835	52,36504
ID866		108,8792	74,033445	153,19624	78,482505	74,77579	82,985145	227,15109	211,505855	299,775075	37,90569	126,544755	134,41384
ID121837		50,6045	72,5239	45,29254	82,422295	50,163365	88,111555	65,18407	58,396375	69,64329	50,86741	63,78509	52,87719
ID352		106,69928	95,194945	85,1333	125,250635	125,743765	132,015745	53,85699	24,8213	33,500085	6,56977	39,561005	48,483145
ID2158110		91,39396	176,76618	78,23103	119,903845	134,140415	110,86878	333,665945	636,721995	764,0856	134,22708	115,12845	91,763
ID1566		177,413945	102,47751	106,731185	97,108745	81,02185	101,748805	248,67079	269,15515	280,516515	99,79044	151,62601	136,00911
ID23159		112,827115	116,91289	112,00595	132,85799	137,182865	106,36441	94,41178	69,75188	72,740085	120,28582	88,18255	80,09076
ID1201		89,929215	100,789525	96,861865	117,38972	94,02247	92,51971	105,62123	91,76657	82,50958	91,71055	84,00173	89,003695
ID524		83,297635	96,64711	88,51272	153,371075	143,726395	158,34949	39,195005	56,779395	36,890865	55,37097	56,096775	62,59764
ID1223		139,066785	140,14658	127,080355	133,627715	43,193235	96,643035	26,64868	21,854	16,13246	119,7571	28,896655	107,5582
ID2275539		91,448515	98,13711	102,615185	133,507875	117,224615	116,716785	55,118235	54,337705	74,40083	0,044815	83,39352	65,848565
ID2044505		127,80591	127,971805	114,024195	161,685675	193,20099	187,10851	80,52602	59,73165	75,0134	43,2009	92,72295	81,608285
ID719		158,45342	107,14153	167,43135	75,72458	82,701565	57,776395	279,98584	320,663585	303,310345	190,23729	176,885045	138,590355

Table S-1

		Median of normalized volumes)3											
ID)1	Protein)2	co_0 min	co_30 min	co_60 min	co_24 h	co_48 h	co_72 h	Pl_0 min	Pl_30 min	Pl_60 min	Pl_24 h	Pl_48 h	Pl_72 h
ID1621		80,87756	90,22589	78,998075	165,868695	122,342075	108,431975	32,44845	30,350645	41,615715	101,42878	52,08398	73,629865
ID3068832		95,88613	124,166965	113,658075	130,792485	103,008205	108,04957	78,51962	77,699955	77,288245	94,33156	87,409035	91,18206
ID440482		87,90174	101,7526	98,55691	147,93132	153,817405	177,85072	69,421745	63,56926	70,654455	86,04113	315,94577	313,395625
ID1623		4869,76974	3596,095125	179,96693	201,541225	582,671	103,59811	8535,981155	66317,59775	894,55784	6,91064	56,767295	56,11012
ID2064822		83,73835	85,87047	81,17295	158,189925	134,546335	144,23538	40,15303	33,764495	35,825535	59,85732	43,08702	46,94949
ID1059		83,530105	92,5088	90,766225	92,12436	119,327115	134,98056	106,030705	112,97221	129,15127	103,96222	46,92663	43,97593
ID5273		50,91426	51,71629	52,218555	110,26471	86,77814	122,35192	31,538365	20,069065	25,88078	51,61702	70,18779	72,983235
ID1085		109,79544	423,744	257,064505	846,601775	2335,97252	378,98688	50,008635	136,64122	86,91391	8,76387	0	5,52146
ID2040831		95,28774	106,8871	93,27085	119,8782	113,479305	144,73616	90,77197	69,165645	85,90493	53,54666	96,50449	80,51717
ID2977290		218,640315	372,556525	101,232365	193,30513	98,795075	113,37783	4,72551	3,6414	31,26637	59,60785	47,18274	34,024465
ID41789		124,773025	84,95761	126,11684	63,76998	77,062445	118,019765	190,989195	210,653645	217,13034	148,72614	146,22075	115,71979
ID3091679		2,348455	5,276395	20,64808	52,248815	53,95996	79,984825	31,045795	24,09118	20,481415	107,12254	52,54347	51,409895
ID1394		65,10797	70,434755	71,564145	89,06197	94,08462	102,505645	35,763095	41,309045	48,607035	140,72042	87,785555	88,5143
ID1028		521,53415	278,731385	229,39805	81,402155	125,094615	63,29319	282,313435	295,5869	277,38822	115,3824	145,45026	69,89416
ID140457		42,965135	8,014975	48,346685	173,698205	212,362645	131,687475	250,569075	225,96191	182,418715	104,63099	125,7767	92,27382
ID3616		93,956875	94,845985	94,43191	96,144385	100,6173	106,818415	108,14198	116,88895	109,784545	131,38085	112,38007	99,71805
ID2290995		139,367525	147,28836	118,410085	140,06255	149,375905	112,38135	66,943345	78,807555	68,275855	83,77681	10,24136	3,456835
ID2970570		99,955475	108,4887	100,911265	106,339725	121,019625	121,076765	124,51661	86,308665	76,56317	119,69719	118,50786	96,702845
ID2039001		87,204105	97,09223	87,01725	115,523215	100,059355	126,22123	90,656405	66,610915	84,085375	73,0962	101,313775	84,59334
ID2096810		118,6478	118,22398	108,71145	114,385795	105,754105	55,259035	119,836125	129,57646	111,9153	135,86864	86,18827	69,89709
ID68693		66,41294	69,299185	72,90697	89,973475	91,94017	69,60692	57,89282	50,40004	58,102695	83,01442	69,868105	74,619235
ID1719		50,03129	58,66594	82,81132	67,74883	62,874195	94,32901	125,874655	447,41827	188,923935	50,95042	60,776155	61,123985
ID421481		166,07288	140,37075	193,901695	95,181045	133,887305	147,788235	90,801235	112,607825	96,317775	95,49834	94,13091	94,197175
ID267419		210,73056	209,071725	315,594985	151,2018	194,27791	75,08305	330,93945	425,747005	296,214325	170,05341	242,42297	163,888845
ID113114		77,04733	87,05339	64,94224	115,508265	119,32391	125,34377	36,96043	54,327665	45,35723	82,61067	76,07529	79,537635
ID1692		96,70764	105,35743	99,907675	129,136585	126,190925	120,64387	72,074085	70,703445	79,901985	5,359735	93,02423	99,48357
ID261244		81,990405	115,566765	88,56072	87,077095	231,079445	126,18489	43,977075	21,779775	36,79138	53,33863	55,65291	62,636385
ID2048120		108,035035	122,876945	100,59085	163,143535	317,75288	244,62494	106,761695	99,114535	115,723255	401,87606	136,9142	120,728925
ID782		143,476535	97,94737	139,256845	68,16177	59,352175	55,95336	249,1485	288,49743	236,022565	201,45621	183,027155	132,12531
ID2027812		49,61539	230,45302	48,10608	953,337465	1036,45253	215,24008	0	29,13066	28,9955	39,12558	0	25,098465
ID1550		43,25204	57,96712	50,00215	114,707665	91,84643	121,017445	27,72337	15,718905	30,33942	50,64825	72,77836	83,14027
ID142473		119,163655	125,271345	98,13372	156,472395	147,693895	154,37951	83,29597	99,18377	81,797035	66,69552	104,321175	73,291935
ID576672		61,66639	56,08203	72,960355	146,044405	140,483865	62,96171	61,962115	55,54762	56,298495	43,17264	68,795295	65,97066
ID10303		137,45062	128,552615	132,396925	108,14848	103,159145	102,357855	122,97417	139,014075	122,53367	100,65424	97,50835	92,14941
ID568037		104,01042	110,66767	106,45814	116,744695	128,15347	83,953465	103,73492	103,480185	91,4138	72,98854	73,46926	74,6384
ID1557		185,28578	237,712765	123,391155	476,184275	245,18711	194,691115	164,34846	57,44878	81,506555	209,94425	64,5436	101,384715
ID3763337		163,11122	189,559165	184,46056	213,80634	101,08652	134,26687	111,85789	49,633245	93,66575	26,67726	3,794815	9,846485
ID1996662		112,128755	138,375265	135,246965	315,74782	n.d.	460,12967	110,78219	105,5287	94,795025	275,85962	n.d.	n.d.
ID414		103,45453	109,657095	105,49204	135,3427	127,276145	149,11418	77,393985	60,733735	60,490185	80,43141	70,44357	75,843965
ID3762817		107,925995	110,30224	150,980265	97,166005	91,24055	92,144735	139,2646	187,56419	160,895845	108,42751	118,867165	71,33925
ID1864541		96,619765	107,90839	111,267995	118,399975	143,506555	104,16228	56,142045	100,35566	87,830255	43,05697	21,544025	32,101455
ID2926520		71,93129	73,67041	95,418615	88,07642	46,43828	40,720325	46,78488	23,02707	21,773005	71,95043	57,224595	120,646895
ID2314456		96,94816	93,87295	71,593985	92,996935	126,71973	100,158995	18,71971	4,24926	4,203865	320,38936	122,604575	184,232965
ID1802534		130,304505	107,08659	128,12831	128,29507	112,186925	83,92484	212,51316	417,15651	206,291765	231,36372	196,755715	56,373265
ID3762259		123,11806	114,905215	112,689275	132,255235	132,18247	116,40973	48,031375	58,762555	57,643705	100,88358	72,67013	77,42799
ID258601		54,441505	60,33249	45,79932	117,958065	64,28487	134,010215	69,025795	97,487965	102,17638	49,91151	35,48705	90,212945
ID1619		76,906345	77,114075	89,127975	109,688345	94,41396	89,975975	81,674755	79,03633	86,463795	75,49876	78,334435	92,16954
ID17878		127,807765	127,76742	123,172025	172,950475	154,395395	114,386925	88,417685	87,46315	89,59825	82,87843	93,424465	98,75056
ID2030105		101,66786	179,873375	106,04834	155,705925	164,273085	147,655775	25,993585	51,391055	43,68814	66,73719	41,5977	74,71061
ID341		97,985045	103,43031	89,23193	158,782515	128,14884	168,562705	74,120395	66,961895	59,595395	140,21489	86,265935	88,014635
ID1520		87,40652	97,010565	92,396215	96,045805	94,689755	105,879515	138,217995	155,481645	184,515105	105,99932	94,379275	92,308165
ID206082		88,183385	86,44233	91,222955	87,0192	86,47265	92,490755	82,954505	69,372625	85,36295	76,09478	66,986575	76,13736
ID2138752		10,9347	13,35486	5,27766	28,63507	20,01703	34,936275	23,761515	1,5973	8,35317	125,71459	31,612705	35,125565
ID1472		179,261705	196,747685	193,81066	157,445205	125,523435	109,475885	167,48256	164,58378	167,399985	172,05745	117,570165	107,390285
ID1978481		12,141135	20,57319	15,423105	48,31476	37,82938	61,74145	17,119365	17,803555	12,713895	73,17372	67,419925	69,828485
ID125371		94,996355	103,303455	89,639395	173,52223	226,22947	160,913085	123,05703	62,22152	73,697295	82,24419	125,11241	87,47393
ID3760248		120,792695	119,94087	126,02133	149,81535	142,125125	126,17982	44,933285	46,831205	52,92016	76,92632	79,924365	82,96835

Table S-1

ID)1	Protein)2	Median of normalized volumes)3											
		co_0 min	co_30 min	co_60 min	co_24 h	co_48 h	co_72 h	PI_0 min	PI_30 min	PI_60 min	PI_24 h	PI_48 h	PI_72 h
ID595		122,71854	127,006645	145,660055	241,894735	311,82467	143,353205	111,95792	125,40102	144,844105	117,04446	907,80403	304,666765
ID2329037		81,069265	90,476365	79,821565	91,08425	93,880565	125,60659	68,508355	66,72315	72,60604	91,30161	68,908515	79,35513
ID1302		16,909025	208,079375	33,360975	390,04402	235,11742	212,895325	25,16135	0,820155	45,24657	72,29867	0,29635	222,7962
ID1593		105,442675	119,3645	101,46756	79,33764	81,329935	104,83973	82,978665	74,86094	87,3663	69,58593	72,958695	62,82048
ID1814782		301,064825	122,18967	117,05639	55,47548	55,22172	48,774935	282,191165	277,801665	266,73876	187,69102	195,54004	134,746575
ID453		101,36451	103,040365	95,603635	134,466695	127,472755	127,864965	57,079515	58,821015	39,46676	7,79265	74,082365	77,325125
ID1763762		81,936455	93,720375	100,42191	173,084145	167,10079	99,21116	32,06184	90,757525	50,4493	0	38,12287	54,54517
ID1787194		113,999885	116,937465	114,8336	178,00682	164,62488	160,436955	73,90044	62,44739	78,968245	16,26677	46,061795	11,469245
ID47508		63,96127	71,17758	67,82299	109,78321	107,218495	104,98856	88,172005	78,24274	79,289565	82,32238	84,4708	84,563935
ID309		95,71194	105,01136	95,75853	142,736375	147,386755	145,481435	68,54034	44,898505	56,40886	41,81139	63,568605	69,79333
ID361		105,73546	116,76749	128,78674	138,748615	136,32053	137,269355	78,567385	60,29072	57,2586	0	70,21911	61,534395
ID1931678		110,546535	119,17795	126,52942	156,28411	224,61808	214,714225	87,0706	81,694155	91,34945	158,15838	178,322905	437,535035
ID1985723		109,35187	113,388705	146,754175	203,2356	145,803245	154,86228	230,426875	149,99366	168,25151	72,13297	63,483035	82,133245
ID2070460		79,474685	87,328595	75,39559	148,1525	125,400165	293,890465	63,161595	34,73102	35,01011	0,16914	18,765555	23,904305
ID527		77,52352	79,755985	70,512945	94,216795	102,96867	164,003485	46,80597	66,017175	46,20453	63,73746	62,060555	77,506795
ID729		111,751875	99,23612	116,111805	85,606385	84,602145	84,81423	174,478645	160,15525	156,50853	158,89521	126,948415	114,9116
ID576		133,80121	128,101235	122,349615	165,06066	144,412325	125,05314	63,579265	57,95422	72,758545	105,07744	92,09313	100,096235
ID1563		294,8711	175,30137	130,60154	115,83268	105,15933	125,985435	101,69198	82,903335	94,936185	88,89503	99,40687	73,229125
ID351055		54,71722	62,54463	56,092765	93,842015	83,84246	112,75983	80,96368	64,24676	67,872545	162,33314	83,990985	74,707795
ID1533		135,62441	155,010535	173,986235	109,030955	136,43358	109,854455	68,873095	61,512985	97,4873	60,57087	60,338805	59,509685
ID2016883		74,06542	96,060855	71,442915	95,12271	102,05925	156,9562	74,7127	78,62687	80,513985	75,817	88,93311	75,39483
ID2788379		111,171535	111,22265	107,385805	146,387135	145,27705	144,831045	77,2247	65,99275	70,20317	91,72955	88,99384	86,8693
ID3485928		23,03434	21,638975	25,936115	56,137675	49,616875	65,538505	49,942745	51,095735	53,473395	196,08946	83,03318	81,41056
ID730		224,542815	133,52471	246,34172	116,878025	167,71506	35,925735	374,117815	760,421815	456,886245	263,17911	310,518035	231,257385
ID1187		77,74749	77,283055	74,887245	93,840615	77,54822	81,227255	118,08997	103,27666	92,66694	98,45173	85,81771	95,93594
ID1138		239,82265	224,60466	459,231485	145,33975	132,677185	117,114605	21,24972	46,585025	41,757985	52,23348	71,333235	96,604695
ID523641		118,07828	115,82578	123,63027	88,89288	92,11215	94,1808	97,009305	101,605575	104,09968	52,47845	102,949615	84,19836
ID266132		171,8162	126,844855	175,842485	95,3788	102,499015	62,894345	295,39129	373,130135	306,631285	156,64836	186,69715	140,397045
ID315424		75,358735	68,01601	70,96752	98,98835	111,006955	96,748135	64,669075	46,32334	56,20935	59,49621	74,64824	73,243215
ID1434		90,835915	100,211235	97,90253	96,57477	106,203485	132,35726	90,25473	108,27206	101,580255	90,35395	84,558545	89,37737
ID543746		108,771565	109,07335	117,818385	135,461385	127,62152	119,8244	129,55582	99,506165	91,29744	80,98357	92,82206	87,94466
ID2106278		92,150305	109,38623	90,389275	186,467955	136,322305	161,48618	36,85709	5,241485	37,984815	14,58099	11,990845	22,930915
ID1562		74,51049	83,93114	77,48233	90,844445	89,551455	107,692245	64,450395	56,947425	63,02931	94,02835	80,904645	73,668805
ID2115505		80,8633	79,088775	97,2154	76,70473	74,307815	149,961705	78,255155	72,377805	72,27483	38,73395	56,10328	53,85178
ID418		162,360395	115,35563	148,99276	66,2143	61,85672	73,012245	237,20025	239,570055	230,243015	196,38218	187,794765	109,74165
ID509563		185,402765	101,04226	102,330855	241,26738	120,647435	93,194695	110,663595	232,769725	134,47626	0	54,81564	84,50641
ID483		92,74516	101,21239	88,258835	125,46156	115,62438	121,999325	77,09067	55,475735	59,94055	52,72991	67,79069	76,9909
ID130985		118,93337	116,18067	121,866775	138,725145	144,93303	134,90765	73,271935	56,298545	60,91359	83,16433	92,20025	85,529935
ID65305		101,46035	114,61633	103,412445	138,829515	147,27214	157,32476	67,880185	70,920435	77,576375	75,49778	115,987485	128,456865
ID1219		108,449985	115,23138	118,918085	128,678975	101,1925	69,988135	72,582155	86,587465	79,1875	85,84094	75,923095	59,21469
ID49585		71,39651	64,952875	65,019275	64,19983	54,92391	60,28137	100,42747	99,270795	106,367605	104,84637	99,71037	87,777405
ID1664		108,52798	113,151235	132,20369	129,27327	134,572695	154,8874	102,32147	102,98819	114,315505	161,91953	104,559055	102,34357
ID2742902		61,47621	68,02739	73,172655	75,38951	70,87705	83,65208	51,30752	40,144725	55,140965	104,95774	79,9505	79,19513
ID407		114,788355	89,738095	113,05445	73,716915	69,12344	89,739415	203,64232	229,92179	206,079295	169,28852	148,034865	114,11308
ID1871260		136,38039	103,16221	237,01502	239,789265	124,071065	40,10067	120,83953	197,389995	129,660445	85,43501	166,083605	38,9046
ID419		156,188855	105,74305	144,989245	61,65529	59,85292	79,01422	234,77101	236,49458	222,093025	185,50557	166,373385	103,882375
ID569671		108,07036	115,842185	109,333205	111,91636	104,786855	80,8054	102,88609	104,810795	103,70216	90,78435	88,50102	72,207855
ID5884		70,75479	67,27594	74,78702	87,0976	105,838855	98,85125	26,76637	32,23328	36,80723	39,36775	81,17758	102,922875
ID1036		93,585525	96,546595	114,854495	94,409625	107,78918	100,92311	84,959565	83,67535	90,99164	102,70727	64,271965	66,53752
ID1081		114,958235	112,390395	130,486855	98,29751	88,51492	118,506655	63,798385	65,931605	59,522445	46,21735	55,477315	65,004795
ID1101		133,867195	141,18257	144,316385	142,762235	149,62815	144,93156	47,789215	45,92498	55,894365	59,81239	80,985385	87,06468
ID92901		47,774935	64,34315	63,1642	107,72178	121,43875	104,42345	72,743025	78,94643	77,97792	227,93326	103,965315	108,325085
ID2150207		25,12696	30,394155	34,1329	64,33286	21,76046	98,29184	96,35531	83,13524	103,7608	42,3432	48,67372	36,50564
ID2649893		61,451305	63,68599	61,70526	98,42037	80,103005	105,008825	77,03599	76,14507	82,198875	80,47801	59,76198	58,162335
ID1518		159,5304	151,41849	265,550265	139,308035	95,951405	83,68948	74,669695	37,59171	70,73281	75,97517	97,1296	127,118765
ID1663		100,64788	108,913895	94,8277	133,81593	125,72368	88,08534	96,935085	86,31525	100,93747	147,11407	107,67483	79,12692
ID147614		2,43505	3,501645	9,869415	87,0361	54,16631	116,24403	18,21142	11,47814	12,1504	96,38925	56,947705	57,65576

Table S-1

ID[1]	Protein[2]	Median of normalized volumes[3]											
		co_0 min	co_30 min	co_60 min	co_24 h	co_48 h	co_72 h	PI_0 min	PI_30 min	PI_60 min	PI_24 h	PI_48 h	PI_72 h
ID2352301		88,804605	101,92573	91,98611	91,85327	100,152555	124,667905	85,908245	93,41626	82,818745	101,24446	80,44374	83,73396
ID1559		55,737365	64,313395	55,486035	71,340315	72,837445	75,85233	349,380395	318,55564	399,866615	176,94504	112,138365	91,46191
ID211213		64,73198	69,746895	66,90758	83,274055	92,034775	80,528905	62,709985	67,713315	64,5012	211,81742	88,059895	88,62107
ID1534		117,080535	119,64276	201,812205	113,098765	159,2855	114,687705	62,666995	27,346685	65,32947	60,06085	59,24753	55,12182
ID9232		106,10505	106,371025	108,46782	100,950605	112,366715	101,994315	90,457905	80,28645	85,025095	146,20381	93,63336	79,062075
ID1303		145,55733	172,23936	166,092905	146,09963	125,20006	114,072525	80,572325	110,70315	94,56817	91,77174	73,77506	93,991145
ID529		80,976115	86,46009	77,62198	119,394115	127,921965	128,51136	56,537525	49,4038	56,22581	50,06465	73,24094	81,106735
ID57837		83,01335	83,69646	84,722435	106,423965	126,475945	114,42089	37,74197	41,15351	44,483115	47,17216	87,42897	101,489285
ID29087		112,291455	79,298915	119,231575	62,51018	47,223895	51,69238	205,212775	281,708655	232,71372	249,62082	179,652325	140,74402
ID139104		82,18552	83,68521	92,04534	92,534725	100,3388	142,1263	80,23118	67,917935	76,288485	78,53424	77,54226	69,495155
ID344430		63,04341	63,50533	62,74591	87,95295	117,518565	103,047385	52,9263	50,601375	66,516695	133,48572	90,827135	97,119035
ID1295		85,54462	135,44666	48,78551	141,455385	134,700195	144,338505	60,035895	31,5322	47,166215	56,76264	13,813185	10,520615
ID1846930		3,95659	8,629315	7,66973	17,49456	50,309505	23,129555	9,644265	48,48782	50,033305	34,315	40,84221	44,5395
ID1190		71,829185	71,55657	73,118805	100,395305	79,41341	76,16782	85,52413	42,01113	45,20506	76,21235	76,862955	84,145165
ID1980598		n.d.	n.d.	n.d.	234,675205	145,4583	181,50459	91,087925	198,50671	169,37243	6,19211	0	0
ID1135		98,97814	93,57167	135,441485	88,414505	89,239315	96,466205	82,36368	89,60453	83,413245	59,31517	60,223305	67,91251
ID1935188		121,22397	144,878305	138,16151	142,746715	259,430345	217,3063	152,83108	137,642205	154,652145	120,19567	212,73553	453,84223
ID115953		82,80941	103,429985	67,598085	105,8365	107,96019	124,42344	65,90304	90,372995	89,60677	86,54981	79,46744	87,08314
ID2379		92,58309	90,686835	101,79174	102,494225	91,690845	54,82692	40,83151	36,03316	38,7386	17,42374	113,72794	148,27287
ID137923		98,23699	91,72591	96,08264	89,63839	93,044245	127,9899	84,32849	69,46497	64,481295	91,05298	78,7545	78,94983
ID2019749		121,61963	130,040985	131,986775	126,636805	127,758395	107,60094	37,872835	90,28675	42,04431	113,85609	107,98776	83,76478
ID431675		87,081575	99,092145	78,34474	115,527225	97,62729	99,104575	165,9295	131,72401	171,81153	463,39985	160,99189	142,551735
ID2214		58,69531	61,12139	70,81779	142,625145	85,29751	102,778995	70,527915	90,973485	90,04677	67,31214	57,993985	56,889395
ID83022		112,98355	119,424205	123,97176	101,53757	103,1296	116,242625	71,86962	68,02296	77,0408	79,36153	72,016355	74,279545
ID1954		102,87867	111,92872	115,466275	120,73192	n.d.	6885,750755	59,726145	74,23885	77,291035	174,22498	n.d.	250,0316
ID3763726		117,095445	122,54615	120,61022	126,65894	128,258355	121,14704	85,962155	109,144675	109,228225	99,04305	110,33825	89,531965
ID9000		151,307655	147,760195	150,87107	59,267945	41,85705	66,98712	50,453415	70,50528	53,543285	25,37923	38,28215	58,49482
ID1700		115,01214	98,4996	122,275625	108,129045	124,387085	103,3101	46,24273	33,366755	88,73333	313,96246	206,611915	186,328975
ID1339		126,04406	112,17297	105,627075	111,261395	99,40992	111,65556	80,69504	67,54123	66,45716	57,77957	64,51471	67,662225
ID883		89,109235	80,91299	80,993295	118,6212	117,81798	126,541995	99,114885	110,02108	108,43912	125,39226	97,132965	89,0509
ID1797353		129,589745	153,225755	207,616835	169,989445	168,877675	118,8386	29,850805	21,844015	47,25429	51,1796	45,65573	39,950035
ID1490		80,395825	123,002535	216,888845	181,78645	401,77426	90,647715	51,612665	279,32822	70,007905	108,23317	477,693655	337,856775
ID1195		112,57918	118,73733	122,43396	121,372205	107,554145	95,30217	95,72908	91,675665	83,810385	96,32342	90,85486	97,93571
ID41097		135,160465	85,848455	134,12954	45,9094	45,953285	62,902945	244,012085	287,459625	269,929865	197,29234	163,6089	120,70846
ID4489		51,133575	53,886855	52,26441	89,07747	89,85432	103,539745	24,93503	22,22044	26,121505	63,03838	59,57034	69,3079
ID343050		381,493195	438,53621	528,721805	106,20669	93,8384	69,79804	841,44104	3608,29332	1576,49529	65,40071	86,75559	81,17143
ID12822		50,13933	46,19173	48,53141	133,593915	106,47587	88,070905	57,213635	73,687955	60,430795	85,95148	112,759495	121,849895
ID1820		67,10135	76,267185	71,572435	175,126265	183,524125	94,47787	53,027055	54,6996	60,804595	107,06156	176,27518	176,96325
ID485827		86,55777	88,956095	77,40999	120,68776	109,418445	105,864025	69,533805	77,632865	67,88262	99,05793	94,727925	103,63467
ID1395		68,255055	71,836565	78,163965	114,259295	121,83994	113,441465	46,67643	50,97719	54,551925	92,83993	100,99149	95,11794
ID642		114,940115	120,18981	118,788205	151,536745	134,706235	135,085005	45,53342	35,42586	56,054205	70,57001	48,238595	38,726565
ID713		181,952315	123,36779	175,20564	63,68519	56,697745	54,588305	295,319755	376,03097	311,07443	202,86315	186,34578	135,85062
ID3022983		107,901335	120,90907	114,069265	77,391785	80,12003	83,03768	115,40325	112,19169	113,704825	53,4604	18,132875	19,88209
ID288865		81,34734	90,513305	64,99742	115,72115	122,625185	119,481495	85,2948	132,00012	88,56919	131,43473	128,063325	154,651775
ID2081733		91,138015	100,197935	96,979025	125,08616	127,281355	172,96811	75,7733	49,954335	60,69408	16,57732	60,11664	49,829285
ID1819877		214,94548	136,700315	120,467435	151,88531	95,551485	109,30962	105,33094	111,885365	174,25464	238,84351	42,449815	41,06516
ID3557801		114,97594	137,146945	117,21203	152,538075	161,38254	109,664955	141,564295	145,41808	146,01213	114,8282	111,288175	88,719775
ID348		89,59107	92,636085	92,291035	111,65375	103,65219	124,42482	91,518915	77,018175	72,68601	75,34445	80,30701	69,608715
ID75950		91,53777	111,445615	109,71526	132,38383	116,91312	123,027545	85,901375	87,976775	106,163885	63,46537	78,25755	79,78551
ID1164		107,088265	116,11471	112,31182	108,477165	110,333715	114,301125	82,58357	94,103555	90,047285	108,2264	74,19207	73,51291
ID1175		112,887245	131,985335	124,203805	127,28691	115,62221	121,349855	86,92343	93,92937	85,800905	69,38516	87,29969	91,998825
ID1301		81,26834	86,313355	93,027795	96,78887	139,688345	115,66251	63,896055	60,150265	69,37369	11,921	5,674285	0,9402
ID2304779		94,910655	98,58668	113,479855	121,94473	113,541185	101,980845	67,97641	80,344025	93,465135	86,2275	99,0705	82,82714
ID138687		79,320105	75,256055	75,55895	92,392325	97,243835	123,621705	88,67473	105,62735	89,67909	122,99731	96,00606	86,11583
ID1590		117,28594	109,83218	138,062935	91,17366	95,67413	83,291785	121,58287	121,41036	136,261805	35,17509	90,33574	123,39547
ID300154		182,702325	340,36394	162,217385	280,249365	287,65439	171,92608	44,406995	46,13605	49,896075	66,3655	72,488895	79,01263
ID158651		129,277965	143,675545	134,47381	122,495385	134,448055	103,866635	64,53159	72,749845	53,36934	63,49695	69,085405	55,1719

Table S-1

ID[1]	Protein[2]	Median of normalized volumes[3]											
		co_0 min	co_30 min	co_60 min	co_24 h	co_48 h	co_72 h	PI_0 min	PI_30 min	PI_60 min	PI_24 h	PI_48 h	PI_72 h
ID1540		59,84025	89,360415	79,16127	617,252875	348,3593	460,039225	112,968375	33,9295	21,82921	342,36918	673,92726	547,658955
ID1537		3230,033905	1749,39121	331,68777	217,72824	222,233155	610,85381	2257,421615	1107,11174	1063,25417	2,34912	129,922065	10,39804
ID1398		96,17105	109,24088	106,264745	53,921435	105,4303	129,292965	73,181345	79,162485	72,179065	87,76954	104,661145	78,962035
ID11611		79,490725	102,826445	65,9484	76,82372	155,36683	282,141595	189,25235	138,077145	215,568	62,18937	61,99061	46,10876
ID1780185		112,632495	115,0499	113,062515	164,44894	154,988535	152,418465	61,92563	2,412525	54,46447	8,9967	23,56336	20,53198
ID938		130,00201	154,413455	183,36247	347,274975	421,745675	144,88709	92,10737	85,30033	67,603275	71,79642	110,554645	85,584335
ID3762296		124,521455	100,542815	115,739435	74,41259	65,91915	71,725895	191,45306	213,242065	211,683585	176,26662	176,489915	135,9988
ID1466		66,67105	71,761995	70,639105	75,649045	74,905175	73,72907	183,413775	190,548045	248,361295	152,7956	136,355415	112,14171
ID1568		122,34401	128,51952	126,878615	132,56879	123,39423	120,142175	60,08307	70,71017	81,97941	91,89186	94,648075	122,767445
ID586		54,238855	55,96623	68,726925	48,351615	10,00056	104,60038	22,05916	27,56523	36,428405	38,58667	15,62749	16,99368
ID37898		121,28608	129,11032	116,24601	151,13591	144,2459	146,265685	38,770985	39,32136	32,92366	19,52248	66,82149	70,34162
ID1286		103,32148	111,746505	110,69941	121,522785	113,636475	120,34312	123,18086	97,684835	89,445015	105,48216	86,88816	78,463045
ID2023391		93,40809	107,527385	94,31841	105,19748	97,234105	107,98443	72,76407	71,118975	80,34307	71,59269	67,605405	64,8821
ID389942		68,07212	83,52292	48,783005	109,46375	98,792655	104,58573	44,63804	22,32262	31,89796	86,39612	77,36352	88,70842
ID1281		128,73098	137,074125	128,159295	132,015655	110,15715	134,582615	119,972875	74,39905	57,789085	119,27151	95,86337	83,398965
ID1338		158,26173	92,747145	152,50476	62,72126	45,7101	52,113915	305,49524	349,89047	322,656635	11,71089	244,00662	148,36486
ID1594		113,632765	120,84638	113,178105	144,569465	143,91953	136,781565	54,11036	47,10892	70,427415	57,14373	67,917785	67,711345
ID16882		87,006105	96,542885	94,632185	100,443255	101,03002	116,287405	91,022325	93,48081	110,936545	91,9462	82,0507	76,53579
ID40401		143,42854	87,333105	144,905665	50,674985	53,200645	42,915425	251,22497	300,334095	288,46815	199,29144	174,716735	125,782615
ID317853		64,038725	58,88287	62,684185	79,477445	93,884465	103,61332	62,87604	45,88981	57,16075	65,02554	71,123675	70,83957
ID131133		112,830765	109,61742	113,681565	135,89198	139,001605	145,278175	75,971945	64,525155	67,91503	90,46607	86,906925	80,581165
ID574883		61,90115	67,50103	68,76158	95,358185	77,304335	80,883245	69,216555	68,385775	85,941135	88,17793	52,9095	69,336845
ID2522		67,83769	78,272575	79,279755	82,961795	78,702085	71,376925	57,630865	55,190595	68,635945	172,53386	81,124385	73,060275
ID543455		105,38979	103,06349	106,774365	99,047235	118,42796	122,28969	81,22002	82,90378	85,519605	160,59008	94,07557	77,104455
ID1811207		81,086845	54,455685	66,400125	106,854105	15,26506	44,649545	588,993225	349,37395	290,5182	186,72258	164,19901	97,291955
ID3543802		102,78576	117,7252	115,09785	119,749585	97,02779	87,336135	93,58823	90,95423	85,18291	90,57278	70,031365	74,962965
ID987		164,24297	172,79844	212,50267	172,682945	114,36971	103,379715	54,82221	288,210555	249,13299	89,4846	84,843395	79,363035
ID3497145		112,423025	122,030925	135,847525	68,81591	82,51999	67,5512	159,44832	187,91526	178,850435	23,01285	16,998505	3,736265
ID3062814		82,44445	66,986855	81,134	114,09089	154,23387	128,663105	97,51173	97,516495	90,474945	102,56714	86,225695	76,7923
ID2722863		20,13368	10,31599	30,69113	94,743035	30,431635	108,207145	269,363855	189,62529	296,059515	5,6828	42,23716	11,279455
ID51803		56,853595	77,5689	51,545195	117,5188	178,388735	151,784695	20,89612	8,462775	17,993545	4,93605	52,671965	79,653845
ID501		115,994745	120,319105	133,13864	110,439745	123,014345	112,972965	72,93516	52,657525	76,22423	0	59,66699	58,812545
ID440		93,11234	90,836575	88,949055	127,766315	131,73999	145,56281	47,72763	46,253855	33,221615	24,36247	66,547965	72,673195
ID2549343		123,720935	127,202795	199,638055	142,847885	261,83178	225,48647	77,105255	42,715175	78,82471	64,69418	161,690345	143,073585
ID2784333		90,09745	95,700705	83,23815	123,63955	111,236145	96,56999	58,09983	45,05993	59,381315	190,06871	77,375365	75,44281
ID2740977		38,440355	36,521735	46,31367	58,037015	55,59753	70,34844	47,493965	40,181215	51,017545	117,12123	74,442005	74,39239
ID2221628		103,29689	113,02594	108,5676	134,052405	141,625765	133,61141	60,30146	59,989015	64,37824	31,30066	80,55073	62,71289
ID1535		86,84397	101,360095	87,814495	128,943555	132,08591	134,481215	40,47067	45,11821	60,10822	44,37343	77,96534	72,900655
ID589226		106,15912	109,658475	103,25565	89,7709	82,88338	97,286505	133,808475	156,099955	156,49096	113,49637	149,437815	129,465985
ID574393		107,23047	111,17093	102,89028	133,401065	125,459465	141,630745	64,61717	69,042265	67,36722	72,35268	72,634005	73,085265
ID3019322		200,989615	240,48644	224,31618	170,95251	155,244355	303,89828	100,155345	200,38193	213,76808	76,12425	118,027135	98,394885
ID1809464		5,81851	25,20552	18,21267	161,865735	32,13476	271,83368	n.d.	5,21719	n.d.	0	15,010195	82,65868
ID1765003		129,509245	120,7177	119,537445	83,19121	233,95487	74,00612	98,52272	55,15441	70,329115	23,98704	0	0
ID25129		105,61189	127,13387	93,392255	196,051275	147,27615	89,31077	65,417405	52,543355	74,176245	99,94237	92,714835	104,764
ID706		142,73955	144,89017	157,22193	144,13414	135,13965	79,431385	168,213115	207,92518	187,4109	133,08714	124,00275	98,454175
ID146603		86,29179	93,52475	89,512285	145,95561	140,52023	109,13184	44,503705	2,69478	62,000915	163,40756	111,271835	115,149565
ID141431		103,88165	84,92999	61,12886	177,785905	225,4191	197,862735	132,08672	65,30685	1338,43798	91,00318	76,50653	82,81822
ID1844173		90,54919	68,81938	72,997875	58,435695	44,894505	44,37721	199,46385	170,4485	195,87918	129,10759	119,60712	102,22096
ID2100931		74,517385	81,770905	78,18456	13,74136	66,15909	96,41397	52,21959	79,703105	62,670345	106,30699	201,32169	139,61357
ID58577		124,01995	180,090505	71,79957	169,761015	150,85036	119,51684	0	1,17198	4,21038	79,22636	96,365005	120,00688
ID2099065		98,806975	98,693465	99,73239	184,86294	134,582245	114,42919	85,30784	128,22688	94,58988	131,55675	187,093605	136,43845
ID1129		83,950835	92,18565	79,149215	130,18384	102,075825	118,61245	55,60902	54,4136	56,745245	80,89114	76,6361	77,58956
ID1545		99,84626	111,09121	98,73274	127,489425	121,36521	115,491645	68,83499	65,59699	79,308375	108,89203	81,896145	69,45467
ID1763803		106,353055	114,859555	109,979935	171,44166	140,208405	149,944245	36,79988	7,773065	48,46438	2,15931	9,387805	8,168075
ID461679		99,24852	98,750565	124,371165	85,517565	99,0436	125,270365	74,726955	52,675505	70,70496	67,18041	52,032795	51,532065
ID2592168		337,1304	0,69788	103,34598	231,62483	206,447615	796,621415	n.d.	3090,76636	506,66059	86,18725	0	0
ID2354041		35,519245	41,59888	42,1294	54,439525	69,77037	77,03347	44,331725	54,580725	56,342585	91,95672	70,138265	76,666805

Table S-1

ID[1]	Protein[2]	Median of normalized volumes[3]											
		co_0 min	co_30 min	co_60 min	co_24 h	co_48 h	co_72 h	PI_0 min	PI_30 min	PI_60 min	PI_24 h	PI_48 h	PI_72 h
ID2974603		81,717445	91,756585	80,7711	101,315695	95,148975	115,16436	61,258405	57,53368	62,03811	63,2841	82,61586	77,109835
ID3763073		78,521595	73,23173	68,197775	87,9567	121,30427	122,166245	130,657765	130,530755	122,102505	84,26883	103,679775	58,212175
ID320497		117,465315	122,255185	107,891355	138,81901	162,05816	207,06571	72,06987	74,185645	61,080245	76,82252	86,944955	72,8495
ID1538		106,997105	131,1965	108,0989	120,512455	113,334685	127,087195	121,95233	96,931045	121,152965	98,787	83,21353	80,657145
ID89291		71,013065	80,615175	69,113	168,72176	102,889975	113,466115	75,90456	20,203685	50,81431	79,13009	66,50221	47,34136
ID377		101,932	113,093125	113,181805	174,955015	163,915485	151,576445	42,852375	34,081655	33,55918	0	51,051415	41,74678
ID590		94,15706	103,264805	97,823085	138,55836	158,07679	150,608385	64,30915	46,56995	74,85335	8,02359	73,73256	69,931355
ID31044		58,94079	66,878775	62,801225	99,69369	88,45773	98,56271	58,937585	43,497305	53,872505	66,726155	92,146675	85,99744
ID323167		99,91281	98,24606	106,744515	116,195315	123,705675	121,50091	105,420835	90,28489	80,21844	73,74985	79,933555	77,23258
ID386066		67,398735	73,658675	79,627625	113,73428	95,075565	110,643405	59,439455	36,74814	49,726705	104,34857	70,291655	74,431945
ID57776		89,52877	94,808145	88,511615	65,60779	84,58441	94,20617	119,66903	126,041295	121,21231	135,7756	102,868035	91,150665
ID3275754		103,93434	119,70719	114,96195	119,353125	107,569415	153,460095	166,495015	129,516395	109,452585	15,62739	41,96846	20,246255
ID1712		122,93802	138,137915	135,41243	119,03486	117,09945	94,339565	108,39386	100,935905	118,49554	97,84574	92,752885	94,382895
ID1650		75,13102	83,599	74,08376	87,758365	98,674515	105,928135	62,70311	122,09045	70,433805	83,96955	89,655035	80,3937
ID2729506		135,90269	79,654595	135,836555	43,218965	43,776285	48,577345	279,803805	363,489075	275,841615	273,38475	241,31047	157,50152
ID495		65,87788	73,6995	71,99423	101,97527	93,916825	106,60332	39,76124	30,54457	34,247785	60,40968	75,97479	84,181905
ID16614		100,390515	104,500075	111,41293	86,71997	87,90825	103,82946	76,372205	70,427585	96,574465	109,42147	84,97401	74,655315
ID167310		131,31765	151,26069	138,22415	146,18736	138,46675	122,610145	70,714955	84,714565	93,477885	88,83764	85,543655	90,303525
ID185058		71,313225	73,702855	73,73822	92,55473	97,7565	110,853025	100,736125	82,65474	86,72345	95,66108	88,47859	76,456395
ID1544		97,038565	103,092075	91,68229	119,97317	109,26788	116,33504	70,70789	65,823785	76,21448	84,07149	73,625665	75,643255
ID2142601		21,921225	6,8382	39,94863	14,57335	7,66057	17,149775	89,76496	45,896795	80,509775	20,28775	7,27023	1,266965
ID3479720		97,898195	52,11595	73,04462	43,399565	22,4133	40,45059	237,64551	202,856365	264,22523	182,37411	170,061935	106,207835

Table S-2: Established protein spot index obtained from Delta 2D analysis. Only protein spots, which show fold-changes ≤(-1.5) and ≥1.5 for at least one time point are indicated (1187).

Protein identifications as well as informations obtained from Ingenuity Pathway Analysis (IPA)-software package were appended.

Fold-changes were calculated by the division of the Medians of normalized values of each time point after plasma treatment by the Medians of normalized values of the respective time point of untreated (control) cells.

)[1] hit ID obtained from Delta2D analysis (Delta2D statistically software version 4.4, Decodon (Germany, Greifswald))

)[2] protein identification corresponding to human proteins (UniProt-SwissProt database; Rel. 51.5 restricted to human taxonomy)

)[3] Fold-changes (fc) after 0, 30, 60 min and 24, 48 and 72 h of plasma treatment of S9 epithelial cells

)[4] IPA (Ingenuity pathway analysis) symbol

)[5] Entrez gene name (protein function) obtained from IPA

)[6] location in the cell obtained from IPA

)[7] type(s) of protein function obtained from IPA

Table S-2

ID)[1]	Protein)[2]	0 min)[3]	30 min)[3]	60 min)[3]	24 h)[3]	48 h)[3]	72 h)[3]	IPA symbol)[4]	Entrez gene name)[5]	location)[6]	type(s))[7]
ID1449	**1433B**,1433G	-1,721	-1,877	-1,587	-1,352	-1,467	-1,719	YWHAB	tyrosine 3-monooxygenase/tryptophan 5-monooxygenase activation protein, beta polypeptide	cytoplasm	other
ID1403	1433E	-1,572	-1,710	-1,545	-1,464	-1,568	-1,725	YWHAE	tyrosine 3-monooxygenase/tryptophan 5-monooxygenase activation protein, epsilon polypeptide	cytoplasm	other
ID1391	**1433E**,CLIC1	-1,335	-1,349	-1,249	-1,555	-1,435	-1,409	YWHAE	tyrosine 3-monooxygenase/tryptophan 5-monooxygenase activation protein, epsilon polypeptide	cytoplasm	other
ID1425	1433G	-1,660	-1,793	-1,716	-1,425	-1,480	-1,263	YWHAG	tyrosine 3-monooxygenase/tryptophan 5-monooxygenase activation protein, gamma polypeptide	cytoplasm	other
ID1438	1433S	-1,533	-1,561	-1,341	-1,507	-1,560	-1,557	SFN	14-3-3 protein sigma		
ID1429	**1433T**,1433Z	-1,964	-1,970	-1,859	-1,676	-1,675	-1,624	YWHAQ	tyrosine 3-monooxygenase/tryptophan 5-monooxygenase activation protein, theta polypeptide	cytoplasm	other
ID1430	1433Z	-1,576	-1,645	-1,548	-1,364	-1,421	-1,487	YWHAZ	tyrosine 3-monooxygenase/tryptophan 5-monooxygenase activation protein,zeta polypeptide	cytoplasm	other
ID767	**2AAB**,UBQL1	1,793	3,406	1,999	4,191	3,819	2,907	PPP2R1B	protein phosphatase 2, regulatory subunit A, beta	unknown	phosphatase
ID1399	**3HIDH**,PSME1	-1,675	-1,913	-1,616	-1,310	-1,535	-1,726	HIBADH	3-hydroxyisobutyrate dehydrogenase	cytoplasm	enzyme
ID651	ACPH	-1,624	-1,323	-1,442	-1,677	-1,560	-1,687	APEH	N-acylaminoacyl-peptide hydrolase	cytoplasm	peptidase
ID1114	ACTB	1,386	1,862	1,366	1,898	1,698	1,688	ACTB	actin, beta	cytoplasm	other
ID1067	ACTG	-1,038	-1,007	1,092	-2,134	-1,403	1,285	ACTG1	actin, gamma 1	cytoplasm	other
ID1073	**ACTG**,ACTB	1,741	1,481	1,311	1,076	-1,209	-1,537	ACTG1	actin, gamma 1	cytoplasm	other
ID1008	**ACTG**,ACTB	-1,128	-1,087	-1,225	-1,335	-1,286	-1,582	ACTG1	actin, gamma 1	cytoplasm	other
ID360270	**ACTG**, ACTB	-2,224	-1,413	-3,080	-1,013	-1,303	-1,163	ACTG1	actin, gamma 1	cytoplasm	other
ID209100	**ACTG**, ACTB	-3,098	-24,977	-5,578	-1,414	-1,390	-1,535	ACTG1	actin, gamma 1	cytoplasm	other
ID1055	**ACTG**, IF34	1,042	1,480	1,851	-1,242	1,251	1,291	ACTG1	actin, gamma 1	cytoplasm	other
ID1034	**ACTG**, K1C18	1,591	1,450	1,314	-1,153	-1,179	-1,461	ACTG1	actin, gamma 1	cytoplasm	other
ID1080	**ACTG**, K1C18	-1,336	-1,403	-1,573	-1,634	-1,697	-1,537	ACTG1	actin, gamma 1	cytoplasm	other
ID1227	**ACTG**, PP2AB	-1,377	-1,578	-1,414	-1,329	-1,109	-1,330	ACTG1	actin, gamma 1	cytoplasm	other
ID517	ACTN4	-1,399	-1,508	-1,633	-1,728	-1,698	-1,544	ACTN4	actinin, alpha 4	cytoplasm	other
ID8247	ACTN4	-1,346	-1,895	-1,802	-2,927	-2,060	-1,811	ACTN4	actinin, alpha 4	cytoplasm	other
ID518	**ACTN4**, ERO1A	-1,697	-1,903	-1,846	-2,135	-1,896	-1,445	ACTN4	actinin, alpha 4	cytoplasm	other
ID507	**ACTN4**, HS105	-1,933	-2,094	-2,091	-2,292	-2,069	-1,500	ACTN4	actinin, alpha 4	cytoplasm	other
ID8700	**ACTN4**, MVP	-2,168	-1,457	-2,174	-2,505	-1,452	-1,862	ACTN4	actinin, alpha 4	cytoplasm	other
ID1047	**ADRM1**, ACTG	-1,176	-1,575	-1,803	-1,486	-1,547	-1,319	ADRM1	adhesion regulating molecule 1	plasma membrane	other
ID1077	**AHSA1**, ACTG	-1,772	-1,899	-1,604	-1,344	-1,330	-1,219	AHSA1	AHA1, activator of heat shock 90kDa protein ATPase homolog 1 (yeast)	cytoplasm	other
ID169714	**AIBP**, CI032	-1,623	-1,409	-1,521	-1,285	-1,468	-1,352	NAXE	NAD(P)H-hydrate epimerase		
ID894	AL1B1	-1,195	-1,549	-1,210	-11,621	-1,153	-1,098	ALDH1B1	aldehyde dehydrogenase 1 family, member B1	cytoplasm	enzyme
ID939	AL9A1	-1,708	-1,761	-1,474	-2,091	-1,639	-1,676	ALDH9A1	aldehyde dehydrogenase 9 family, member A1	cytoplasm	enzyme
ID1386	ALBU	-1,844	-1,617	-1,661	-1,569	-1,518	-1,505	ALB	serum albumin		
ID1828633	ALDOA	1,473	3,254	1,784	3,488	3,495	2,434	ALDOA	fructose-bisphosphate aldolase A		
ID2003479	AN32A	-2,962	-1,387	-1,548	1,075	-1,770	1,025	ANP32A	acidic leucine-rich nuclear phosphoprotein 32 family member A		
ID3661699	**ANXA1**, DCPS	1,359	1,130	-1,102	-1,363	-1,446	-1,651	ANXA1	annexin A1		
ID2588249	**ANXA2**, NACA	-5,373	-4,819	1,059	-1,463	-1,288	-1,663	ANXA2	annexin A2	plasma membrane	other
ID1351	**ANXA3**	-1,606	-1,725	-1,651	-1,719	-1,568	-1,350	ANXA3	annexin A3	cytoplasm	enzyme
ID213992	ANXA5	-1,710	-1,618	-1,374	-1,661	-1,595	-1,760	ANXA5	annexin A5	plasma membrane	other
ID1345	**ANXA5**, SFRS2	2,019	2,078	1,659	1,947	1,244	-1,170	ANXA5	annexin A5	plasma membrane	other
ID341707	APT	-8,968	-6,247	-5,750	-4,329	-1,190	-1,014	APRT	adenine phosphoribosyltransferase		
ID965	ARP3	-1,690	-1,986	-1,653	-2,411	-1,920	-1,677	ACTR3	ARP3 actin-related protein 3	plasma membrane	other
ID3329884	**ARP3**, TADBP	55,374	8,673	5,524	1,637	1,511	1,239	ACTR3	ARP3 actin-related protein 3	plasma membrane	other
ID1046	**ARP3**, TADBP	1,545	1,185	1,210	-1,261	-1,439	-1,779	ACTR3	ARP3 actin-related protein 3	plasma membrane	other
ID1152	ASNA1	-1,865	-2,127	-1,805	-1,463	-1,443	-1,493	ARSA1	ATPase ASNA1	nucleus	enzyme
ID3737	ATPB	-1,150	-1,253	1,036	1,552	1,027	-1,046	ATP5B	ATP synthase, H+ transporting, mitochondrial F1 complex, beta polypeptide	cytoplasm	transporter
ID936	BASP	-1,724	-3,369	-1,765	-1,867	-1,984	-1,496	BASP1	brain acid soluble protein 1		
ID48368	BASP	-4,466	-101,014	-9,762	-3,160	-2,017	-2,963	BASP1	brain acid soluble protein 1		
ID2010414	**BCCIP**, ACTG	-1,195	-1,395	-1,050	-1,394	-1,496	-2,422	BCCIP	BRCA2 and CDKN1A-interacting protein	nucleus	
ID1596	**BID**, RBM8A	-1,023	-1,691	-1,389	-2,122	-1,523	-1,987	BID	BH3 interacting domain death agonist	cytoplasm	other
ID951	BLMH, LA	-1,543	-1,516	-1,583	-1,766	-1,362	-1,422	BLMH	bleomycin hydrolase	cytoplasm	peptidase
ID488759	CALD1	-1,483	-1,762	-1,321	6,007	-2,367	-1,780	CALD1	caldesmon 1	cytoplasm	other
ID1702	CALM	-1,297	3,171	-1,153	1,012	-1,189	-1,152	CALM1	calmodulin 1 (phosphorylase kinase, delta)	plasma membrane	other
ID745	**CALR**, KAP0	1,875	2,607	2,264	3,600	3,665	2,590	CALR	calreticulin	cytoplasm	transcription regulator
ID53317	CALU	1,089	-1,077	1,109	1,404	-1,189	-1,581	CALU	calumenin	cytoplasm	other
ID54018	CALU	1,090	-1,101	1,205	1,202	-1,431	-1,919	CALU	calumenin	cytoplasm	other
ID2006762	CALU	-2,395	-2,907	-1,625	-3,976	-38,999	-5,875	CALU	calumenin	cytoplasm	other
ID1106	CAPG	-1,339	-1,658	-1,329	-1,421	-1,375	-1,367	CAPG	capping protein (actin filament), gelsolin-like	nucleus	other
ID496907	CAPG	-1,975	-2,111	-2,089	-2,535	-1,783	-2,427	CAPG	capping protein (actin filament), gelsolin-like	nucleus	other
ID1440	CAPZB	-1,245	-1,387	-1,198	-1,207	-1,473	-1,598	CAPZB	F-actin-capping protein subunit beta	cytoskeleton	
ID1512	CATB	1,771	3,379	2,274	1,925	1,566	1,136	CTSB	cathepsin B	cytoplasm	peptidase

Table S-2

ID)[1]	Protein)[2]	0 min)[3]	30 min)[3]	60 min)[3]	24 h)[3]	48 h)[3]	72 h)[3]	IPA symbol)[4]	Entrez gene name)[5]	location)[6]	type(s))[7]
ID163898	CBX1	1,282	-1,260	-1,069	-1,453	-1,419	-1,556	CBX1	chromobox protein homolog 1	nucleus	other
ID1547	CBX1	-1,099	-2,149	-1,812	-1,455	-1,271	-1,130	CBX1	chromobox protein homolog 1	nucleus	other
ID1618	CBX3	-2,072	-5,708	-3,537	-9,741	-4,576	-6,039	CBX3	chromobox protein homolog 3	nucleus	other
ID1530	**CBX5**, CATB	1,198	1,239	1,319	-1,858	-1,518	-1,806	CBX5	chromobox homolog 5	nucleus	other
ID3270147	**CBX5**, CATB	1,144	-1,257	-1,110	1,121	1,637	-1,144	CBX5	chromobox homolog 5	nucleus	other
ID941	CD2B2	-1,546	-2,273	-1,504	-1,463	-2,048	-1,824	CD2BP2	CD2 antigen cytoplasmic tail-binding protein 2	nucleus	other
ID958	**CDC37**, PRS6B	-1,459	-1,705	-1,549	-1,533	-1,413	-1,359	CDC37	Hsp90 co-chaperone Cdc37		
ID826	**CH60**, STK3	1,529	1,710	1,570	1,354	1,183	-1,087	HSPD1	heat shock 60kDa protein 1 (chaperonin)	cytoplasm	enzyme
ID1268	**CHM4B**, RPB3	-1,258	-1,554	-1,350	-1,213	-1,257	-1,196	CHMP4B	charged multivesicular body protein 4b	cytoplasm	
ID2212082	CHP1	1,161	-1,348	-1,041	-6,972	-1,601	-2,310	CHP1	calcineurin B homologous protein 1		
ID1378	CLIC1	-2,109	-1,833	-1,747	-1,457	-1,486	-1,301	CLIC1	chloride intracellular channel 1	nucleus	ion channel
ID1478	**CLIC4**, ACTG, NNMT	1,197	1,385	1,165	2,790	1,112	-1,103	CLIC4	chloride intracellular channel protein 4	nucleus	ion channel
ID909	**CNDP2**, HNRH1	-1,609	-1,793	-1,796	-2,012	-2,029	-1,799	CNDP2	CNDP dipeptidase 2 (metallopeptidase M20 family)	cytoplasm	peptidase
ID901	**CNDP2**, PEPD	-1,436	-1,522	-1,375	-1,625	-1,670	-1,601	CNDP2	CNDP dipeptidase 2 (metallopeptidase M20 family)	cytoplasm	peptidase
ID3392731	**CNDP2**, PEPD	-1,510	-2,060	-1,547	-1,424	-1,278	-1,472	CNDP2	CNDP dipeptidase 2 (metallopeptidase M20 family)	cytoplasm	peptidase
ID1168	CNN3	-1,233	-1,524	-1,410	-1,600	-1,439	-1,252	CNN3	calponin 3, acidic	cytoplasm	other
ID1165	CNN3	-1,167	-1,541	-1,651	-1,551	-1,609	-1,244	CNN3	calponin 3, acidic	cytoplasm	other
ID1154	**CNN3**, IDH3A	-1,528	-1,926	-1,562	-1,949	-1,284	-1,321	CNN3	calponin 3, acidic	cytoplasm	other
ID1641	COF1	-2,618	-2,639	-1,874	-1,704	-2,418	-2,170	CFL1	cofilin 1 (non-muscle)	nucleus	other
ID693	**COR1B**, CBR4	1,160	-1,061	1,070	1,467	2,382	1,794	CORO1B	coronin, actin binding protein, 1B	cytoplasm	other
ID1769	COTL1	-1,885	-2,007	-1,616	-1,290	-1,308	-1,077	COTL1	coactosin-like 1 (Dictyostelium)	cytoplasm	other
ID791	CPNE1	1,014	1,008	-1,029	-1,726	-1,407	-1,526	CPNE1	copine I	unknown	transporter
ID2001699	CPNS1	-1,472	-1,308	-1,638	-1,218	-1,265	-1,297	CAPNS1	calpain, small subunit 1	cytoplasm	peptidase
ID1401	**CPNS1**, GDIR	-1,779	-1,766	-1,458	-1,512	-1,628	-1,604	CAPNS1	calpain, small subunit 1	cytoplasm	peptidase
ID1502	CRK	1,559	2,706	1,806	1,872	1,489	1,422	CRK	adapter molecule crk	cytoplasm	other
ID17270	CSN4	-1,589	-2,003	-1,782	-1,441	-1,417	-1,293	COPS4	COP9 constitutive photomorphogenic homolog subunit 4 (Arabidopsis)	cytoplasm	other
ID1099	**CSN4**, MPI, BPNT1	-1,207	-1,650	-1,358	-1,043	-1,325	-1,217	COPS4	COP9 constitutive photomorphogenic homolog subunit 4 (Arabidopsis)	cytoplasm	other
ID1723	CT077	1,204	-1,088	1,356	-10,709	-1,613	-1,412				
ID327583	DC1I2	-1,107	-1,276	-1,136	-1,004	-1,380	-1,494	DYNC1I2	dynein, cytoplasmic 1, intermediate chain 2	cytoplasm	other
ID650	DC1I2	-1,344	-1,510	-1,390	-2,099	-1,941	-1,840	DYNC1I2	dynein, cytoplasmic 1, intermediate chain 2	cytoplasm	other
ID586647	**DC1L2**, TCPB	-1,157	-1,250	-1,222	-3,496	-1,515	-1,312	DYNC1LI2	dynein, cytoplasmic 1, light intermediate chain 2	cytoplasm	other
ID380472	DCTN1	2,463	3,956	2,725	4,295	7,157	6,670	DCTN1	dynactin 1	cytoplasm	other
ID302775	**DCTN2**, PABP2	-1,528	-1,715	-1,602	-1,370	-1,430	-1,362	DCTN2	dynactin 2 (p50)	cytoplasm	other
ID3348523	DCUP	-1,512	-1,856	-1,560	-1,986	-1,176	-1,512	UROD	uroporphyrinogen decarboxylase		
ID902	**DD19A**, MPPA	-1,185	-1,373	-1,200	-4,435	-1,880	-1,788	DDX19A	DEAD (Asp-Glu-Ala-As) box polypeptide 19A	nucleus	enzyme
ID893	**DD19A**, TBG1	-1,387	-1,522	-1,469	-6,156	-1,398	-1,223	DDX19A	DEAD (Asp-Glu-Ala-As) box polypeptide 19A	nucleus	enzyme
ID1259	**DDAH1**, CAZA1	-1,560	-1,476	-1,635	1,093	-1,109	-1,066	DDAH1	dimethylarginine dimethylaminohydrolase 1	cytoplasm	enzyme
ID129069	DDB1	1,161	1,238	1,010	-1,131	-1,211	-1,501	DDB1	DNA damage-binding protein 1	nucleus	
ID436	**DDB1**, OXRP	-1,063	-1,089	-1,277	-1,651	-1,534	-1,460	DDB1	DNA damage-binding protein 1	nucleus	
ID1169	**DJB11**, ROAA	-1,323	-1,345	-1,535	-1,291	-1,364	-1,221	DNAJB11	DnaJ homolog subfamily B member 11	ER	
ID983	**DNJA2**, APMAP	-1,062	-1,307	-1,146	-1,524	-1,472	-1,391	DNAJA2	DnaJ (Hsp40) homolog, subfamily A, member 2	nucleus	enzyme
ID445851	**DNJA2**, APMAP	-1,179	-1,502	-1,358	-1,354	-1,218	-1,258	DNAJA2	DnaJ (Hsp40) homolog, subfamily A, member 2	nucleus	enzyme
ID1319	DNJC9	-1,547	-1,559	-1,693	-2,179	-1,419	-1,462	DNAJC9	DnaJ homolog subfamily C member 9	nucleus	enzyme
ID122740	DP13B	-1,107	-1,509	-1,368	4,097	1,655	1,323	APPL2	adaptor protein, phosphotyrosine interaction, PH domain and leucine zipper containing 2	cytoplasm	other
ID13335	DPOD2	-1,007	-1,070	1,261	-2,381	-2,027	-2,188	POLD2	polymerase (DNA directed), delta 2, regulatory subunit 50kDa	nucleus	enzyme
ID410	DPOD2	-1,743	-3,027	-2,984	-4,523	-3,201	-2,521	POLD2	polymerase (DNA directed), delta 2, regulatory subunit 50kDa	nucleus	enzyme
ID244116	DPOD2	-1,920	-3,462	-4,204	-5666,230	-11,607	-4,932	POLD2	polymerase (DNA directed), delta 2, regulatory subunit 50kDa	nucleus	enzyme
ID755	DPYL2	1,839	2,897	2,284	3,639	3,796	2,655	DPYSL2	dihydropyrimidinase-related protein 2	cytoplasm	enzyme
ID778	**DPYL2**, LKHA4	-1,231	-1,565	-1,260	-9,398	-1,331	-1,173	DPYSL2	dihydropyrimidinase-related protein 2	cytoplasm	enzyme
ID644	**DPYL2**, ZYX, EZRI	-1,228	-1,429	-1,336	1,918	-1,242	-1,166	DPYSL2	dihydropyrimidinase-related protein 2	cytoplasm	enzyme
ID435	DREB	-1,431	-2,097	-1,672	-2,045	-1,497	-1,480	DBN1	drebrin 1	cytoplasm	other
ID381	DYNA	-1,646	-2,086	-1,799	-2,423	-1,838	-2,020	DCTN1	dynactin 1	cytoplasm	other
ID1352	**ECH1**, PSDE	1,095	-1,015	1,035	-1,582	-1,402	-1,934	ECH1	enoyl CoA hydratase 1, peroxisomal	cytoplasm	enzyme
ID1435	ECHM	-1,103	-1,141	1,029	-1,953	-1,620	-1,784	ECHS1	enoyl CoA hydratase, short chain, 1, mitochondrial	cytoplasm	enzyme
ID1382	EF1B	-1,145	-1,518	-1,228	-1,187	-1,231	-1,031	EEF1B2	elongation factor 1-beta	cytoplasm	enzyme
ID1278	EF1D	-1,085	-1,529	-1,133	1,019	-1,196	-1,181	EEF1D	elongation factor 1-delta	nucleus	enzyme
ID546	EF2	-1,128	-1,708	-1,465	-1,266	-1,223	-1,365	EEF2	elongation factor 2	nucleus	enzyme
ID610	EFG1	-1,140	-1,396	-1,329	-3,806	-1,509	-1,450	GFM1	elongation factor G, mitochondrial	mitochondrium	enzyme
ID1128	**EI2BL**, CAPG	-1,645	-1,868	-1,453	-1,905	-1,553	-1,416				

Table S-2

ID)[1]	Protein)[2]	0 min)[3]	30 min)[3]	60 min)[3]	24 h)[3]	48 h)[3]	72 h)[3]	IPA symbol)[4]	Entrez gene name)[5]	location)[6]	type(s))[7]
ID1078	**EIF3G**, ARP3	-1,200	-1,324	-1,225	-1,725	-1,594	-1,371	EIF3G	eukaryotic translation initiation factor 3, subunit G	cytoplasm	translation regulator
ID1913428	ELOB	-1,268	-1,514	-1,485	-5,265	-2,730	-4,564	ELOB	elongin-B	nucleus	
ID1015	ENOA	2,143	3,336	2,280	3,541	3,430	2,521	ENO1	enolase 1, (alpha)	cytoplasm	enzyme
ID1001	ENOG	-1,860	-1,771	-1,620	-1,812	-1,564	-1,592	ENO2	enolase 2 (gamma, neuronal)	cytoplasm	enzyme
ID15652	ENOG	-2,058	-1,952	-1,877	-1,514	-1,413	-1,526	ENO2	enolase 2 (gamma, neuronal)	cytoplasm	enzyme
ID953	**ENOG**, RBBP7	-1,091	-1,292	-1,118	-1,826	-1,546	-1,615	ENO2	enolase 2 (gamma, neuronal)	cytoplasm	enzyme
ID274688	ENPL	1,215	1,296	1,260	1,792	1,114	-1,211	HSP90B1	endoplasmin	cytoplasm	other
ID132924	ENPL	2,025	-1,086	2,471	-1,212	-1,414	-1,530	HSP90B1	endoplasmin	cytoplasm	other
ID104582	ENPL	-1,154	-1,624	-1,133	1,074	-1,253	-1,380	HSP90B1	endoplasmin	cytoplasm	other
ID2298466	**ERP29**, IF4E	-1,254	-1,534	-1,363	-2,225	-1,367	2,181	ERP29	endoplasmic reticulum protein 29	cytoplasm	transporter
ID1463	**ERP29**, TPIS	-1,733	-1,650	-1,478	-1,693	-1,319	-1,286	ERP29	endoplasmic reticulum protein 29	cytoplasm	transporter
ID625	**EZRI**, CALD1, SYK	-1,339	-1,593	-1,375	2,136	-1,299	-1,298	EZR	ezrin	plasma membrane	other
ID191574	FKB10	-1,317	-1,678	-1,272	-1,364	-1,508	-1,242	FKBP10	peptidyl-prolyl cis-trans isomerase FKBP10	ER	enzyme
ID387435	FKB10	-1,145	-1,758	-1,536	-1,547	-1,504	-1,934	FKBP10	peptidyl-prolyl cis-trans isomerase FKBP10	ER	enzyme
ID643	**FKB10**, DC1I2	-1,353	-1,510	-1,331	-1,142	-1,163	-1,233	FKBP10	peptidyl-prolyl cis-trans isomerase FKBP10	ER	enzyme
ID871	FKBP4	-1,643	-1,997	-1,633	-1,389	-1,284	-1,266	FKBP4	FK506 binding protein 4, 59kDa	nucleus	enzyme
ID878	**FKBP4**, HMCS1	-1,433	-1,901	-1,521	-1,427	-1,256	-1,135	FKBP4	FK506 binding protein 4, 59kDa	nucleus	enzyme
ID692	FKBP9	1,556	1,236	1,561	-1,083	-1,070	-2,544	FKBP9	FK506 binding protein 9, 63 kDa	cytoplasm	enzyme
ID1173	**FSTL1**, ANXA2	1,221	-1,742	1,779	-1,056	-1,588	-1,581	FSTL1	follistatin-related protein 1	extracellular space	
ID1137	**GALK1**, ILEU	1,175	-1,186	-1,170	-7,085	-1,655	-1,734	GALK1	galactokinase 1	cytoplasm	kinase
ID1087	**GALK1**, ILEU	1,046	-1,101	1,035	-4,583	-1,080	-1,199	GALK1	galactokinase 1	cytoplasm	kinase
ID509	GANAB	-1,042	-1,127	-1,107	-1,257	-1,671	-2,510	GANAB	glucosidase, alpha; neutral AB	cytoplasm	enzyme
ID496	GANAB	-1,169	-1,235	-1,135	-1,428	-1,772	-2,132	GANAB	glucosidase, alpha; neutral AB	cytoplasm	enzyme
ID460138	GANAB	-1,116	-1,619	-1,651	-4,108	-2,141	-2,197	GANAB	glucosidase, alpha; neutral AB	cytoplasm	enzyme
ID4596	GANAB	-2,305	-2,293	-1,970	-2,060	-1,877	-1,768	GANAB	glucosidase, alpha; neutral AB	cytoplasm	enzyme
ID510	**GANAB**, IMMT	-1,426	-1,437	-1,484	-2,246	-1,941	-2,248	GANAB	glucosidase, alpha; neutral AB	cytoplasm	enzyme
ID499	**GANAB**, IMMT	-1,143	-1,441	-1,258	-1,445	-1,692	-2,071	GANAB	glucosidase, alpha; neutral AB	cytoplasm	enzyme
ID825	**GDIA**, ANXA3	1,200	1,992	1,379	1,880	1,542	1,318	GDI1	GDP dissociation inhibitor 1	cytoplasm	other
ID828	**GDIA**, VTDB	1,567	3,029	1,842	2,929	2,413	2,064	GDI1	GDP dissociation inhibitor 1	cytoplasm	other
ID940	GDIB	-2,375	-2,315	-2,131	-2,001	-1,854	-1,588	GDI2	GDP dissociation inhibitor 2	cytoplasm	other
ID1513	**GDIR1**, LGUL	-1,677	-1,722	-1,411	-1,418	-1,387	-1,413	ARHGDIA	Rho GDP-dissociation inhibitor 1		
ID17482	GELS	1,193	1,458	1,482	1,814	1,442	-1,496	GSN	gelsolin	extracellular space	other
ID584	**GELS**, MX1	-1,174	-1,320	-1,281	-2,474	-1,459	-1,685	GSN	gelsolin	extracellular space	other
ID1167	GLRX3, TXNL2	-1,758	-1,966	-1,622	-1,649	-1,439	-1,344	GLRX3	glutaredoxin 3	cytoplasm	enzyme
ID3296127	GMFB	-1,473	-1,738	-1,515	-1,589	-1,059	1,071	GMFB	glia maturation factor beta		
ID505	**GRAP1**, GANAB	-1,219	-1,270	-1,272	-2,259	-1,877	-2,369	GRIPAP1	GRIP1 associated protein 1	plasma membrane	other
ID309937	**GRAP1**, GANAB	-3,194	-9,644	-7,798	-3,002	-1,767	-1,551	GRIPAP1	GRIP1 associated protein 1	plasma membrane	other
ID2845794	GRP78 (BIP)	1,191	1,783	1,101	1,500	-1,017	-1,157	HSPA5	heat shock 70kDa protein 5 (glucose-regulated protein, 78kDa)	cytoplasm	other
ID3760	GRP78 (BIP)	1,728	1,717	1,314	5,797	2,933	2,553	HSPA5	heat shock 70kDa protein 5 (glucose-regulated protein, 78kDa)	cytoplasm	other
ID594	GRP78 (BIP)	1,538	1,540	1,303	1,250	1,176	-1,272	HSPA5	heat shock 70kDa protein 5 (glucose-regulated protein, 78kDa)	cytoplasm	other
ID672	GRP78 (BIP)	1,455	1,523	1,202	1,283	-1,214	-1,283	HSPA5	heat shock 70kDa protein 5 (glucose-regulated protein, 78kDa)	cytoplasm	other
ID661	GRP78 (BIP)	1,267	1,365	1,097	2,845	1,740	1,667	HSPA5	heat shock 70kDa protein 5 (glucose-regulated protein, 78kDa)	cytoplasm	other
ID662	GRP78 (BIP)	1,044	1,259	-1,010	1,918	1,216	1,264	HSPA5	heat shock 70kDa protein 5 (glucose-regulated protein, 78kDa)	cytoplasm	other
ID2846419	GRP78 (BIP)	1,114	1,067	-1,754	1,031	-1,348	-1,647	HSPA5	heat shock 70kDa protein 5 (glucose-regulated protein, 78kDa)	cytoplasm	other
ID366771	GRP78 (BIP)	2,071	1,056	2,126	-1,739	-1,636	-4,240	HSPA5	heat shock 70kDa protein 5 (glucose-regulated protein, 78kDa)	cytoplasm	other
ID10719	GRP78 (BIP)	2,064	-2,767	2,955	2,056	-1,108	1,290	HSPA5	heat shock 70kDa protein 5 (glucose-regulated protein, 78kDa)	cytoplasm	other
ID318	GRP78 (BIP)	-2,963	-3,015	-51,057	-4,716	-2,194	-2,229	HSPA5	heat shock 70kDa protein 5 (glucose-regulated protein, 78kDa)	cytoplasm	other
ID105671	**GRP78 (BIP)**, ENPL	-1,448	1,248	-1,590	1,161	-1,102	1,194	HSPA5	heat shock 70kDa protein 5 (glucose-regulated protein, 78kDa)	cytoplasm	other
ID120878	**GRP78 (BIP** , PDIA4	1,893	-1,051	1,250	-1,248	-1,085	-1,698	HSPA5	heat shock 70kDa protein 5 (glucose-regulated protein, 78kDa)	cytoplasm	other
ID960	GSHB	-1,668	-1,781	-1,606	-1,397	-1,351	-1,364	GSS	glutathione synthetase	cytoplasm	enzyme
ID943	**GSHB**, K2C8	-1,189	-1,842	-1,916	-1,989	-1,659	-1,746	GSS	glutathione synthetase	cytoplasm	enzyme
ID622	**GSPT1**, HS90A	-1,902	-2,226	-1,833	-1,951	-1,556	-1,281	GSPT1	G1 to S phase transition 1	cytoplasm	translation regulator
ID1408	**GSTO1**, ERP29	1,000	-1,176	1,063	-7,525	-1,336	-1,493	GSTO1	glutathione S-transferase omega 1	cytoplasm	enzyme
ID422947	**GSTO1**, PSB7	-1,187	-1,432	-1,293	-1,575	-1,635	-1,430	GSTO1	glutathione S-transferase omega 1	cytoplasm	enzyme
ID1516	GSTP1	-1,790	-1,644	-1,508	-1,481	-1,463	-1,474	GSTP1	glutathione S-transferase pi 1	cytoplasm	enzyme
ID1194	HDGF	-1,702	-1,789	-1,566	-1,872	-1,487	-1,686	HDGF	hepatoma-derived growth factor	extracellular space	growth factor
ID88373	HDGF	-1,824	-1,933	-1,655	-2,274	-1,494	-1,820	HDGF	hepatoma-derived growth factor	extracellular space	growth factor
ID1211	HDGF	-1,613	-2,007	-1,561	-2,324	-1,573	-2,050	HDGF	hepatoma-derived growth factor	extracellular space	growth factor
ID1231	HDGF	-2,296	-2,249	-1,824	-1,628	-1,135	-1,120	HDGF	hepatoma-derived growth factor	extracellular space	growth factor

Table S-2

ID)[1]	Protein)[2]	0 min)[3]	30 min)[3]	60 min)[3]	24 h)[3]	48 h)[3]	72 h)[3]	IPA symbol)[4]	Entrez gene name)[5]	location)[6]	type(s))[7]
ID217409	HEXB	1,248	1,301	1,200	-1,064	-1,391	-1,494	HEXB	beta-hexosaminidase subunit beta	lysosome	enzyme
ID447263	**HNRH1**, CNDP2	-1,005	1,146	1,038	-1,639	-1,889	-1,454	HNRNPH1	heterogeneous nuclear ribonucleoprotein H1 (H)	nucleus	other
ID923	**HNRH1**, CNDP2	1,082	-1,137	-1,199	-1,769	-1,885	-1,976	HNRNPH1	heterogeneous nuclear ribonucleoprotein H1 (H)	nucleus	other
ID918	**HNRH1**, CNDP2	-1,024	-1,516	-1,646	-1,973	-1,643	-1,606	HNRNPH1	heterogeneous nuclear ribonucleoprotein H1 (H)	nucleus	other
ID971	**HNRH1**, GDIB	-1,383	-1,524	-1,463	-1,726	-1,433	-1,458	HNRNPH1	heterogeneous nuclear ribonucleoprotein H1 (H)	nucleus	other
ID29521	**HNRH1**, HNRH2	-1,140	-1,642	-1,634	-2,072	-1,661	-1,662	HNRNPH1	heterogeneous nuclear ribonucleoprotein H1 (H)	nucleus	other
ID471051	**HNRH1**, TIF1B	-1,201	-1,450	-1,408	-4,109	-1,549	-1,527	HNRNPH1	heterogeneous nuclear ribonucleoprotein H1 (H)	nucleus	other
ID457	HNRL2	-1,077	-1,935	-1,864	-3,319	-2,198	-2,203	HNRNPUL2	heterogeneous nuclear ribonucleoprotein U-like 2	nucleus	other
ID458	HNRL2	-1,252	-2,150	-1,846	-2,943	-1,975	-1,988	HNRNPUL2	heterogeneous nuclear ribonucleoprotein U-like 2	nucleus	other
ID441	HNRL2	-1,302	-2,275	-1,952	-2,842	-2,082	-2,069	HNRNPUL2	heterogeneous nuclear ribonucleoprotein U-like 2	nucleus	other
ID493	HNRL2	-2,017	-5,598	-3,182	-2,519	-1,318	-1,086	HNRNPUL2	heterogeneous nuclear ribonucleoprotein U-like 2	nucleus	other
ID1203	HNRPC	-2,187	-2,831	-2,453	-1,578	-1,410	-1,498	HNRNPC	heterogeneous nuclear ribonucleoprotein C (C1/C2)	nucleus	other
ID2964442	**HNRPC**, STRAP	-2,734	-6,509	-3,687	-1,654	-1,352	1,290	HNRNPC	heterogeneous nuclear ribonucleoprotein C (C1/C2)	nucleus	other
ID305540	**HNRPC**, STRAP	-3,169	-7,618	-4,172	1,184	-1,661	1,271	HNRNPC	heterogeneous nuclear ribonucleoprotein C (C1/C2)	nucleus	other
ID770	HNRPK	1,732	3,238	1,844	3,799	3,875	2,844	HNRNPK	heterogeneous nuclear ribonucleoprotein K	nucleus	other
ID788	HNRPK	-1,096	-1,547	-1,466	-1,243	-1,139	-1,357	HNRNPK	heterogeneous nuclear ribonucleoprotein K	nucleus	other
ID797	**HNRPK**, HSP7C	1,173	-1,257	-1,413	-1,238	-1,183	-1,526	HNRNPK	heterogeneous nuclear ribonucleoprotein K	nucleus	other
ID879	**HNRPK**, TBL1R	-1,743	-2,090	-1,471	-1,712	-1,821	-1,833	HNRNPK	heterogeneous nuclear ribonucleoprotein K	nucleus	other
ID391	HNRPU	-1,147	-1,457	-1,284	-1,692	-1,852	-1,567	HNRNPU	heterogeneous nuclear ribonucleoprotein U (scaffold attachment factor A)	nucleus	transporter
ID447	HNRPU	-1,207	-1,668	-1,568	-2,287	-1,590	-2,032	HNRNPU	heterogeneous nuclear ribonucleoprotein U (scaffold attachment factor A)	nucleus	transporter
ID415	**HNRPU**, PPCE	-1,443	-2,069	-1,790	-1,449	-2,384	-2,673	HNRNPU	heterogeneous nuclear ribonucleoprotein U (scaffold attachment factor A)	nucleus	transporter
ID446	**HNRPU**, PPCE	-1,824	-4,034	-2,288	-3,004	-2,739	-2,536	HNRNPU	heterogeneous nuclear ribonucleoprotein U (scaffold attachment factor A)	nucleus	transporter
ID485	HS105	-1,673	-2,108	-1,905	-1,788	-1,412	-1,194	HSPH1	heat shock 105kDa/110kDa protein 1	cytoplasm	other
ID486	HS105	-1,745	-2,173	-1,897	-1,845	-1,491	-1,200	HSPH1	heat shock 105kDa/110kDa protein 1	cytoplasm	other
ID477	**HS105**, GPIA1	-1,503	-1,939	-1,791	-1,755	-1,553	-1,288	HSPH1	heat shock 105kDa/110kDa protein 1	cytoplasm	other
ID628	HS90A	1,566	2,089	1,576	2,364	2,620	1,639	HSP90AA1	heat shock protein 90kDa alpha (cytosolic), class A member 1	cytoplasm	other
ID20414	HS90A	1,299	1,624	1,349	1,446	2,009	1,520	HSP90AA1	heat shock protein 90kDa alpha (cytosolic), class A member 1	cytoplasm	other
ID596190	HS90A	1,473	1,049	-1,190	1,740	1,269	-1,517	HSP90AA1	heat shock protein 90kDa alpha (cytosolic), class A member 1	cytoplasm	other
ID623	HS90A	-1,158	-1,309	-1,251	-1,641	-1,400	-1,652	HSP90AA1	heat shock protein 90kDa alpha (cytosolic), class A member 1	cytoplasm	other
ID3841	HS90A	-1,086	-1,638	-1,549	1,075	-1,020	-1,230	HSP90AA1	heat shock protein 90kDa alpha (cytosolic), class A member 1	cytoplasm	other
ID575	HS90A	-1,595	-1,959	-1,476	-1,626	-1,622	-1,171	HSP90AA1	heat shock protein 90kDa alpha (cytosolic), class A member 1	cytoplasm	other
ID573	HS90A	-1,630	-2,379	-1,655	-1,630	-1,539	-1,078	HSP90AA1	heat shock protein 90kDa alpha (cytosolic), class A member 1	cytoplasm	other
ID3931	HS90A	-1,940	-2,689	-1,984	-1,158	1,117	-1,003	HSP90AA1	heat shock protein 90kDa alpha (cytosolic), class A member 1	cytoplasm	other
ID571	HS90A	-1,849	-2,705	-2,140	-1,739	-1,763	-1,347	HSP90AA1	heat shock protein 90kDa alpha (cytosolic), class A member 1	cytoplasm	other
ID10923	HS90A	-1,793	-3,154	-1,846	-1,746	-1,821	-1,353	HSP90AA1	heat shock protein 90kDa alpha (cytosolic), class A member 1	cytoplasm	other
ID559	**HS90A**, DP13A	-1,655	-2,444	-1,829	-1,716	-1,877	-1,390	HSP90AA1	heat shock protein 90kDa alpha (cytosolic), class A member 1	cytoplasm	other
ID2816455	**HS90A**, HS90B	-1,515	-1,669	-1,496	-1,221	-1,310	1,075	HSP90AA1	heat shock protein 90kDa alpha (cytosolic), class A member 1	cytoplasm	other
ID574	**HS90A**, HS90B	-2,347	-2,683	-2,149	-1,443	-1,576	-1,104	HSP90AA1	heat shock protein 90kDa alpha (cytosolic), class A member 1	cytoplasm	other
ID627	HS90A, **MRE11**	1,352	1,713	1,449	1,518	1,925	1,330	MRE11	double-strand break repair protein MRE11		
ID1703	HSB11	-1,530	-1,487	-1,349	-1,211	-1,249	-1,098	HSPB11	heat shock protein family B (small), member 11	unknown	other
ID717	**HSP71**, PDIA4	1,880	2,565	1,756	3,420	3,713	2,431	HS71A	Heat shock 70 kDa protein 1A		
ID490	HSP74	-1,733	-2,057	-1,758	-1,766	-1,401	-1,306	HSPA4	heat shock 70kDa protein 4	cytoplasm	other
ID491	HSP74	-1,954	-2,277	-1,913	-2,037	-1,590	-1,242	HSPA4	heat shock 70kDa protein 4	cytoplasm	other
ID822	**HSP7C**, AINX	-1,063	-1,438	-1,397	-1,497	-1,379	-1,144	HSPA8	heat shock 70kDa protein 8	cytoplasm	enzyme
ID1455	HSPB1	-1,124	1,907	-1,107	-1,376	-1,040	1,225	HSPB1	heat shock 27kDa protein 1	cytoplasm	other
ID1453	**HSPB1**, ECHM	-1,359	-1,840	-1,194	-18,720	-1,239	-1,303	HSPB1	heat shock 27kDa protein 1	cytoplasm	other
ID376	HYOU1	1,053	-1,027	1,038	1,908	1,081	-1,152	HYOU1	hypoxia up-regulated 1	cytoplasm	other
ID5403	HYOU1	-1,671	-2,450	-1,853	-2,487	-1,747	-1,333	HYOU1	hypoxia up-regulated 1	cytoplasm	other
ID5526	HYOU1	-1,911	-2,708	-1,916	-2,749	-1,902	-1,505	HYOU1	hypoxia up-regulated 1	cytoplasm	other
ID430	ICAL	-1,411	-1,520	-1,304	-1,668	-1,357	-1,543	CAST	calpastatin	cytoplasm	peptidase
ID427	ICAL	-1,395	-1,605	-1,247	-1,724	-1,383	-1,552	CAST	calpastatin	cytoplasm	peptidase
ID126195	ICAL	-1,509	-1,700	-1,291	-1,524	-1,392	-1,619	CAST	calpastatin	cytoplasm	peptidase
ID475	ICAL	-1,583	-2,105	-1,422	-2,601	-1,539	-1,587	CAST	calpastatin	cytoplasm	peptidase
ID428	**ICAL**, DREB	-1,480	-1,951	-1,474	-1,914	-1,484	-1,437	CAST	calpastatin	cytoplasm	peptidase
ID1174	IDH3A	-1,409	-1,886	-1,487	-1,646	-1,076	-1,295	IDH3A	isocitrate dehydrogenase 3 (NAD+) alpha	cytoplasm	enzyme
ID1458	**IDI1**, PSB10	-1,700	-1,669	-1,657	-1,358	-1,604	-1,684	IDI1	isopentenyl-diphosphate Delta-isomerase 1	cytoplasm	enzyme
ID3591281	**IF1AX**, SORCN	-1,147	-1,187	-1,129	-2,866	-1,131	-1,579	EIF1AX	eukaryotic translation initiation factor 1A, X-chromosomal	cytoplasm	translation regulator
ID1196	IF2A	-1,445	-1,724	-1,562	-1,716	-1,480	-1,338	EIF2S1	eukaryotic translation initiation factor 2, subunit 1 alpha, 35kDa	cytoplasm	translation regulator
ID964	**IF2B**, ARP3, QCR1	-1,009	-1,216	-1,114	-1,601	-1,605	-1,605	EIF2B1	translation initiation factor eIF-2B subunit alpha	cytoplasm	translation regulator

Table S-2

ID)[1]	Protein)[2]	0 min)[3]	30 min)[3]	60 min)[3]	24 h)[3]	48 h)[3]	72 h)[3]	IPA symbol)[4]	Entrez gene name)[5]	location)[6]	type(s))[7]
ID1240	**IF31**, ANXA2	-1,712	-1,841	-1,694	-1,354	-1,277	-1,387	EIF3J	eukaryotic translation initiation factor 3, subunit J	cytoplasm	translation regulator
ID1019	**IF4A1**, ACTG	-1,572	-2,000	-1,685	-1,158	-1,232	-1,075	EIF4A1	eukaryotic translation initiation factor 4A1	cytoplasm	translation regulator
ID13655	**IF4A1**, NDRG3	1,237	1,006	1,223	-1,375	-1,441	-1,715	EIF4A1	eukaryotic translation initiation factor 4A1	cytoplasm	translation regulator
ID617	**IF4B**, NEBU	-1,560	-1,916	-1,526	-1,449	-1,402	-1,187	EIF4B	eukaryotic translation initiation factor 4B	cytoplasm	translation regulator
ID1697	IF5A1	-1,885	-1,906	-1,744	-1,853	-1,634	-1,267	EIF5A	eukaryotic translation initiation factor 5A	cytoplasm	translation regulator
ID1465	IF6	-1,392	-1,548	-1,227	-1,440	-1,194	1,021	EIF-6	eukaryotic translation initiation factor 6	cytoplasm	translation regulator
ID1139	**ILEU**, SCMC1	-1,091	-1,554	-1,579	-15,914	-1,273	-1,521	SERPINB1	serpin peptidase inhibitor, clade B (ovalbumin), member 1	cytoplasm	other
ID3406	IMA7	1,112	1,801	1,128	1,392	1,167	1,073	KPNA6	karyopherin alpha 6 (importin alpha 7)	nucleus	transporter
ID577	**IMMT**, PLOD3	-1,043	-1,453	-1,144	-1,669	-1,449	-1,452	IMMT	inner membrane protein, mitochondrial	cytoplasm	other
ID1300	IPYR	-1,594	-1,593	-1,275	-1,349	-1,230	-1,380	PPA1	pyrophosphatase (inorganic) 1	cytoplasm	enzyme
ID1291	**IPYR**, GBB1	-1,793	-1,776	-1,385	-1,413	-1,434	-1,249	PPA1	pyrophosphatase (inorganic) 1	cytoplasm	enzyme
ID195754	ITA3	-1,161	-1,234	-1,201	1,396	-1,354	-1,531	ITGA3	integrin, alpha 3 (antigen CD49C, alpha 3 subunit of VLA-3 receptor)	plasma membrane	other
ID393	ITA3	-1,065	-1,304	-1,202	-1,058	-1,566	-1,652	ITGA3	integrin, alpha 3 (antigen CD49C, alpha 3 subunit of VLA-3 receptor)	plasma membrane	other
ID981	K1C17	1,028	-1,094	-1,183	-1,423	-1,600	-1,360	KRT17	keratin 17	cytoplasm	other
ID927	**K1C17**, ATPB	1,409	1,249	1,503	1,256	-1,079	-1,243	KRT17	keratin 17	cytoplasm	other
ID1086	**K1C18**, ACTB, NUP43	1,109	1,018	1,060	-1,512	-1,457	-1,394	K1C18	keratin 18	cytoplasm	other
ID417	K1C9	1,335	2,192	1,520	3,040	3,191	1,552	KRT9	keratin, type I cytoskeletal 9	cytoplasm	other
ID899	K2C8	1,620	1,550	1,475	1,037	1,012	-1,088	KRT8	keratin 8	cytoplasm	other
ID892	K2C8	1,661	1,500	1,468	-1,048	-1,133	-1,356	KRT8	keratin 8	cytoplasm	other
ID959	**KAP0**, TXND5	-2,077	-3,176	-2,585	-1,823	-1,865	-1,908	PRKAR1A	protein kinase, cAMP-dependent, regulatory, type I, alpha (tissue specific extinguisher 1)	cytoplasm	kinase
ID3670	**KAP2**, IF4A1	1,053	-1,202	-1,011	-2,010	-1,751	-1,513	PRKAR2A	cAMP-dependent protein kinase type II-alpha regulatory subunit	cytoplasm	kinase
ID556498	KBP	1,661	1,126	1,034	1,205	1,108	-2,373	KIF1BP	KIF1-binding protein	cytoplasm	other
ID207376	KCRB	-1,018	-1,322	-1,696	-1,391	-1,709	-2,673	CKB	creatine kinase, brain	cytoplasm	kinase
ID3760270	KU86	-1,446	-1,650	-1,586	-1,977	-1,486	-1,482	XRCC5	X-ray repair cross-complementing protein 5		
ID629	**KU86**, HS90A	-1,370	-1,505	-1,516	-1,680	-1,599	-1,616	XRCC5	X-ray repair cross-complementing protein 5		
ID618	**KU86**, HS90A	-1,366	-1,764	-1,645	-1,827	-1,614	-1,774	XRCC5	X-ray repair cross-complementing protein 5		
ID1962730	LEG1	-1,365	-1,603	-1,478	-1,773	-1,548	1,120	LGALS1	galectin-1	extracellular space	other
ID10509	LG3BP	-1,080	-1,158	1,016	1,715	1,299	1,009	LGALS3BP	lectin, galactoside-binding, soluble, 3 binding protein	plasma membrane	transmembrane receptor
ID777	LKHA4	-1,792	-1,506	-1,864	-1,157	-1,221	-1,430	LTA4H	leukotriene A4 hydrolase	cytoplasm	enzyme
ID765	LMNA	1,624	1,377	1,401	-2,785	-1,505	-2,041	LMNA	lamin A/C	nucleus	other
ID490428	**LMNA**, AGM1	1,535	1,328	1,328	-11,225	-1,235	-1,097	LMNA	lamin A/C	nucleus	other
ID708	LMNB1	1,678	1,245	1,722	-1,172	-1,445	-2,197	LMNB1	lamin B1	nucleus	other
ID712	LMNB1	1,411	1,184	1,333	-1,532	-1,284	-2,137	LMNB1	lamin B1	nucleus	other
ID709	LMNB1	1,102	1,127	-1,021	-1,361	-1,391	-1,833	LMNB1	lamin B1	nucleus	other
ID537545	**LMNB1**, TKT	2,107	1,123	2,427	-1,180	-1,462	-2,650	LMNB1	lamin B1	nucleus	other
ID395	LPPRC	-1,307	-1,596	-1,459	-1,390	-1,824	-1,636	LRPPRC	leucine-rich PPR motif-containing protein, mitochondrial	nucleus	other
ID1308	**LZTL1**, SFRS1	-2,137	-1,990	-1,791	-1,983	-1,626	-1,443	LZTFL1	leucine zipper transcription factor-like 1	unknown	other
ID691	**MAOX**, SWP70	1,159	1,056	1,144	1,139	1,971	1,844	ME1	malic enzyme 1, NADP(+)-dependent, cytosolic		
ID44539	MARCS	1,536	1,880	1,586	1,491	-1,235	1,157	MARCKS	myristoylated alanine-rich protein kinase C substrate	plasma membrane	other
ID1355	**MARE1**, EFHD2	-1,634	-1,800	-1,446	-1,321	-1,350	-1,255	MAPRE1	microtubule-associated protein, RP/EB family, member 1	cytoplasm	other
ID1360	**MARE1**, TBCB	-1,527	-1,651	-1,378	-1,292	-1,321	-1,329	MAPRE1	microtubule-associated protein, RP/EB family, member 1	cytoplasm	other
ID409	MATR3	-1,265	-1,558	-1,423	-1,104	-1,588	-1,928	MATR3	Matrin-3	nucleus	other
ID3316	**MCM6**, 2AAA	1,757	3,200	1,927	3,851	3,545	2,668	MCM6	DNA replication licensing factor MCM6	nucleus	enzyme
ID585	**MD1L1**, GELS	-1,335	-1,581	-1,492	-2,577	-1,738	-1,618	MAD1L1	MAD1 mitotic arrest deficient-like 1 (yeast)	nucleus	other
ID663	MEPD, THOP1	-1,575	-1,641	-1,464	-1,903	-1,831	-1,489	THOP1	Thimet oligopeptidase		
ID1647	MLRM	-1,049	1,069	-1,100	-1,912	-1,851	-1,729	MYL12A	myosin regulatory light chain 12A		
ID2669	MLRM	-1,121	-1,046	-1,068	-1,636	-1,582	-1,299	MYL12A	myosin regulatory light chain 12A		
ID1953484	MLRN	1,065	1,372	1,061	-2,053	-2,193	-1,609	MYL9	myosin regulatory light polypeptide 9		
ID72360	MLRN	-1,560	-1,122	-1,299	-1,521	-1,352	-1,152	MYL9	myosin regulatory light polypeptide 9		
ID1041	MP2K2	-1,364	-1,586	-1,358	-35,809	-1,533	-1,484	MAP2K2	mitogen-activated protein kinase 2	cytoplasm	kinase
ID1936868	MRLC2	1,297	-1,090	-1,052	-13,414	-3,513	-21,986	MYL12B	myosin regulatory light chain 12B	cytoplasm	other
ID508	**MSH2**, GANAB	-1,895	-2,182	-1,978	-2,046	-1,880	-1,916	MSH2	mutS homolog 2, colon cancer, nonpolyposis type 1 (E. coli)	nucleus	enzyme
ID1630	**MTND**, SSRD	-1,618	-1,477	-1,024	-1,159	-1,310	-1,426	ADI1	acireductone dioxygenase 1	nucleus	enzyme
ID540509	MVP	-1,319	-1,375	-1,463	-1,953	-1,588	-1,585	MVP	major vault protein	nucleus	other
ID487	MVP	-1,505	-1,738	-1,899	-2,011	-1,700	-1,626	MVP	major vault protein	nucleus	other
ID460	**MVP**, SYAC	-2,117	-2,305	-2,111	-2,572	-1,911	-1,700	MVP	major vault protein	nucleus	other
ID7894	**MVP**, TIF1B, MVP	-1,364	-1,617	-1,463	-1,872	-1,478	-1,568	MVP	major vault protein	nucleus	other
ID197182	**MVP**, UBA1	-1,581	-1,862	-1,870	-2,177	-1,697	-1,708	MVP	major vault protein	nucleus	other
ID17545	MX1	1,001	1,557	1,237	1,308	1,296	-1,849	MX1	myxovirus (influenza virus) resistance 1, interferon-inducible protein p78 (mouse)	nucleus	enzyme

Table S-2

ID)[1]	Protein)[2]	0 min)[3]	30 min)[3]	60 min)[3]	24 h)[3]	48 h)[3]	72 h)[3]	IPA symbol)[4]	Entrez gene name)[5]	location)[6]	type(s))[7]
ID379418	**MYG1**, SHLB1	-2,155	-3,473	-2,124	-2,592	-1,705	-1,603	C12ORF10	chromosome 12 open reading frame 10	unknown	other
ID1755	MYL6	-1,036	-1,142	1,035	-1,379	-1,574	-1,538	MYL6	Myosin light polypeptide 6	cytoplasm	other
ID1205	NAGK	1,103	-1,123	-1,153	-1,563	-1,270	-1,103	NAGK	N-acetyl-D-glucosamine kinase	cytoplasm	kinase
ID405	NASP	-1,277	-1,879	-1,439	-1,529	-1,328	-2,147	NASP	nuclear autoantigenic sperm protein (histone-binding)	nucleus	other
ID1632	**NDKA**, COMD1	-1,792	-1,691	-1,498	-1,265	-1,277	-1,090	NME1	non-metastatic cells 1, protein (NM23A) expressed in	nucleus	kinase
ID659	NDUS1	1,145	1,046	1,210	-1,766	-1,779	-2,119	NDUFS1	NADH dehydrogenase (ubiquinone) Fe-S protein 1, 75kDa (NADHcoenzyme Q reductase)	cytoplasm	enzyme
ID1432	NNMT	-1,533	-1,382	-1,409	-1,463	-1,597	-1,509	NNMT	nicotinamide N-methyltransferase	cytoplasm	enzyme
ID845	NP1L1	1,617	2,219	1,964	2,811	2,590	1,785	NP1L1	nicotinamide N-methyltransferase	cytoplasm	enzyme
ID926	NP1L1	-1,839	-2,527	-1,705	-1,402	-1,548	-1,220	NP1L1	nicotinamide N-methyltransferase	cytoplasm	enzyme
ID929	**NP1L1**, SYAP1	-1,944	-2,662	-1,751	-1,405	-1,345	-1,069	NP1L1	nicotinamide N-methyltransferase	cytoplasm	enzyme
ID842	NP1L4	1,751	2,737	2,244	3,042	3,145	2,406	NP1L4	nucleosome assembly protein 1-like 4	nucleus	other
ID846	NP1L4	1,615	2,450	2,027	2,826	2,886	2,165	NP1L4	nucleosome assembly protein 1-like 4	nucleus	other
ID2404447	NP1L4	1,486	2,182	1,714	2,111	2,309	2,000	NP1L4	nucleosome assembly protein 1-like 4	nucleus	other
ID1224	NPM	-1,272	-2,628	-1,146	-1,474	1,291	2,098	NPM1	nucleophosmin (nucleolar phosphoprotein B23, numatrin)	nucleus	transcription regulator
ID1225	**NPM**, EF1D	1,296	-1,042	1,008	-1,098	-1,050	1,600	NPM1	nucleophosmin (nucleolar phosphoprotein B23, numatrin)	nucleus	transcription regulator
ID50273	**NSAP**, CALR	2,668	2,751	2,873	1,270	1,354	1,024	SYNCRIP	synaptotagmin binding, cytoplasmic RNA interacting protein	nucleus	other
ID3227	**NUCB1**, VIME	1,562	2,523	1,627	2,124	1,756	1,505	NUCB1	nucleobindin 1	cytoplasm	other
ID520	NUCL	-1,558	-2,115	-1,753	-1,998	-1,639	-1,902	NCL	nucleolin	nucleus	other
ID1364	**NUDT5**, ANXA5	-1,385	-1,615	-1,246	-1,199	-1,321	-1,237	NUDT5	ADP-sugar pyrophosphatase	nucleus	other
ID970	ODO2	-1,675	-1,804	-1,508	-2,817	-1,823	-1,907	DLST	dihydrolipoamide S-Ssuccinyltransferase (E2 component of 2-oxo-glutarate complex)	cytoplasm	enzyme
ID1255	ODPB	-1,092	-1,307	-1,250	-1,496	-1,549	-1,523	PDHB	pyruvate dehydrogenase (lipoamide) beta	cytoplasm	enzyme
ID1282	**ODPB**, CSN6	-1,032	-1,069	-1,101	-2,498	-2,082	-1,988	PDHB	pyruvate dehydrogenase (lipoamide) beta	cytoplasm	enzyme
ID1321	OTUB1	-1,651	-1,868	-1,671	-1,150	-1,275	-1,071	OTUB1	ubiquitin thioesterase OTUB1	cytoplasm	enzyme
ID1312	OTUB1	-1,790	-2,101	-1,583	-1,688	-1,580	-1,361	OTUB1	ubiquitin thioesterase OTUB1	cytoplasm	enzyme
ID4295	P3H1	-1,537	-2,048	-1,719	-1,019	-1,472	-1,363	LEPRE1	leucine proline-enriched proteoglycan (leprecan) 1	nucleus	enzyme
ID2818369	P3H1	-1,916	-2,424	-2,043	-1,315	-1,431	-1,135	LEPRE1	leucine proline-enriched proteoglycan (leprecan) 1	nucleus	enzyme
ID558	P3H3	1,046	-1,272	-1,017	-6,058	-1,608	-1,654	LEPREL2	leprecan-like 2	nucleus	enzyme
ID813	**P4HA1**, TCPA	1,277	1,525	1,353	1,217	1,228	-1,089	P4HA1	prolyl 4-hydroxylase, alpha polypeptide I	cytoplasm	enzyme
ID1412	**PA1B2**, PSME1	-1,271	-1,496	-1,187	-1,377	-1,388	-1,308	PAFAH1B2	platelet-activating factor acetylhydrolase IB subunit beta	nucleus	transcription regulator
ID1032	**PA2G4**, SAHH	-2,284	-2,420	-1,911	-4235,010	-1,846	-1,486	PA2G4	proliferation-associated 2G4, 38kDa	nucleus	
ID1511	PARK7	-1,752	-1,958	-1,645	-2,009	-1,378	-1,393	PARK7	Parkinson disease (autosomal recessive, early onset) 7	nucleus	other
ID1304	PCNA	-1,524	-1,898	-1,456	-1,470	-1,547	-1,746	PCNA	proliferating cell nuclear antigen	nucleus	other
ID1328	**PCNA**, CX026	-1,274	-1,641	-1,208	-1,060	-1,239	-1,389	PCNA	proliferating cell nuclear antigen	nucleus	other
ID230824	PDIA1	1,303	1,519	1,192	1,151	-1,483	-1,361	P4HB	prolyl 4-hydroxylase, beta polypeptide	cytoplasm	enzyme
ID2239	PDIA1	1,609	1,519	1,604	3,066	1,424	1,158	P4HB	prolyl 4-hydroxylase, beta polypeptide	cytoplasm	enzyme
ID824	PDIA1	1,243	1,203	1,277	1,888	1,039	-1,117	P4HB	prolyl 4-hydroxylase, beta polypeptide	cytoplasm	enzyme
ID451640	PDIA3	1,306	1,416	1,108	1,590	-1,007	-1,378	PDIA3	protein disulfide-isomerase A3	cytoplasm	peptidase
ID854	**PDIA3**, HNRPF	1,218	1,040	1,239	1,556	-1,064	-1,273	PDIA3	protein disulfide-isomerase A3	cytoplasm	peptidase
ID17374	**PDIA3**, PEPD	1,259	1,741	1,295	1,180	-1,037	-1,126	PDIA3	protein disulfide-isomerase A3	cytoplasm	peptidase
ID836	**PDIA3**, TBA1B	1,768	2,728	1,699	2,107	1,735	1,283	PDIA3	protein disulfide-isomerase A3	cytoplasm	peptidase
ID370380	PDIA4	1,469	1,700	1,427	1,147	1,409	1,489	PDIA4	protein disulfide-isomerase A4	cytoplasm	enzyme
ID660	PDIA4	1,258	1,538	1,351	1,708	2,889	1,490	PDIA4	protein disulfide-isomerase A4	cytoplasm	enzyme
ID22159	**PDIA4**, HSP71	1,351	1,600	1,428	2,383	2,982	2,044	PDIA4	protein disulfide-isomerase A4	cytoplasm	enzyme
ID982	PDIA6	1,387	1,225	1,443	1,624	1,007	-1,028	PDIA6	protein disulfide-isomerase A6	cytoplasm	enzyme
ID176254	**PDIA6**, F10A1	-1,337	-1,588	-1,303	-1,230	-1,303	-1,298	PDIA6	protein disulfide-isomerase A6	cytoplasm	enzyme
ID1784	PEA15	-2,992	-3,283	-1,698	-1,723	-1,712	-1,692	PEA15	astrocytic phosphoprotein PEA-15	cytoplasm	
ID1522	PFD3	-1,489	-1,694	-1,516	-1,773	-1,351	-1,292	VBP1	von Hippel-Lindau binding protein 1	cytoplasm	other
ID1468	**PHB**, HSPB1	1,340	1,423	1,385	-1,237	-1,423	-1,721	PHB	prohibitin	nucleus	transcription regulator
ID16655	PHP14	-1,586	-1,561	-1,311	-1,919	-1,543	1,772	PHPT1	phosphohistidine phosphatase 1	cytoplasm	phosphatase
ID798	**PNCB**, FKBP4	-1,549	-1,793	-1,580	-1,491	-1,424	-1,230	NAPRT1	nicotinate phosphoribosyltransferase domain containing 1	unknown	enzyme
ID1443	PNPO	-1,536	-1,425	-1,247	-6,307	-1,323	-1,286	PNPO	pyridoxamine 5'-phosphate oxidase	unknown	enzyme
ID1272	PP1A	-1,108	-1,162	1,302	-4,195	-1,209	-1,358	PPP1CA	protein phosphatase 1, catalytic subunit, alpha isozyme	cytoplasm	phosphatase
ID534785	PP1A	-1,790	-1,848	-1,680	-1,461	-1,294	-1,286	PPP1CA	protein phosphatase 1, catalytic subunit, alpha isozyme	cytoplasm	phosphatase
ID1030	**PP1R7**, IF4A1	-2,238	-2,398	-2,187	-1,873	-1,791	-1,742	PPP1R7	protein phosphatase 1, regulatory (inhibitor) subunit 7	nucleus	phosphatase
ID1316	**PP4C**, EF1D	-1,310	-1,627	-1,313	1,031	-1,226	-1,180	PPP4C	Serine/threonine-protein phosphatase 4 catalytic subunit	nucleus	phosphatase
ID2382844	PPM1G	-1,612	-1,517	-1,556	-1,290	-1,584	-1,903	PPM1G	protein phosphatase, Mg2+/Mn2+ dependent, 1G	nucleus	phosphatase
ID1060	PPME1	-1,308	-1,543	-1,478	-1,750	-1,682	-1,501	PPME1	protein phosphatase methylesterase 1	unknown	enzyme
ID1249	PPP6	-1,496	-1,527	-1,466	-1,163	-1,119	-1,096	PPP6C	protein phosphatase 6, catalytic subunit	nucleus	phosphatase
ID1243	**PPP6**, LDHB	-2,087	-1,908	-1,961	-1,347	-1,453	-1,307	PPP6C	protein phosphatase 6, catalytic subunit	nucleus	phosphatase

Table S-2

ID)1	Protein)2	0 min)3	30 min)3	60 min)3	24 h)3	48 h)3	72 h)3	IPA symbol)4	Entrez gene name)5	location)6	type(s))7
ID1296	**PPP6**, PGP	-2,082	-1,635	-1,930	-1,499	-1,746	-1,683	PPP6C	protein phosphatase 6, catalytic subunit	nucleus	phosphatase
ID3102815	**PRS6A**, TXND5	-1,088	-1,407	-1,385	-1,070	-1,481	-1,654	PSMC3	proteasome (prosome, macropain) 26S subunit, ATPase, 3	nucleus	transcription regulator
ID177384	**PRS6A**, TXND5	-1,617	-2,139	-2,210	-1,450	-1,522	-1,376	PSMC3	proteasome (prosome, macropain) 26S subunit, ATPase, 3	nucleus	transcription regulator
ID1541	**PRDX2**, GSTP1	-2,703	-2,934	-2,878	-1,737	-1,536	-1,671	PRDX2	peroxiredoxin 2	cytoplasm	enzyme
ID1510	PRDX3	-2,387	-3,015	-2,500	-134,069	-1,879	-1,803	PRDX3	peroxiredoxin 3	cytoplasm	enzyme
ID584346	**PRDX3**, HSPB1	-2,842	-2,555	-2,003	-1,607	-1,397	-1,384	PRDX3	peroxiredoxin 3	cytoplasm	enzyme
ID3056834	PRDX4	-1,772	-1,379	-1,325	1,465	1,153	2,154	PRDX4	peroxiredoxin 4	cytoplasm	enzyme
ID1497	PRDX4	-2,381	-1,622	-1,726	-1,057	-1,106	-1,051	PRDX4	peroxiredoxin 4	cytoplasm	enzyme
ID1802	**PROF2**, TR112	-1,494	-1,646	-1,419	-1,681	-2,019	-1,180	PFN2	profilin-2	cytoplasm	
ID469498	**PRS4**, SYWC	1,337	1,596	1,357	1,132	-1,076	-1,176	PSMC1	proteasome (prosome, macropain) 26S subunit, ATPase, 1	nucleus	peptidase
ID1002	PRS6A	-1,299	-1,699	-1,514	-1,256	-1,416	-1,363	PSMC3	proteasome (prosome, macropain) 26S subunit, ATPase, 3	nucleus	transcription regulator
ID997	PRS7	-1,303	-1,795	-1,517	-1,556	-1,481	-1,393	PSMC2	26S proteasome regulatory subunit 7	cytoplasm	
ID172969	**PSA3**, TPD54	-1,490	-1,689	-1,560	-1,502	-1,355	-1,222	PSMA3	proteasome (prosome, macropain) subunit, alpha type, 3	cytoplasm	peptidase
ID3058865	PSB6	-1,093	-1,344	-1,280	-1,442	-1,419	-1,781	PSMB6	proteasome subunit beta type-6	nucleus	
ID1402	PSB7	-1,325	-1,729	-1,537	-1,588	-1,330	-1,169	PSMB7	proteasome (prosome, macropain) subunit, beta type, 7	cytoplasm	peptidase
ID1519	PSD10	-1,522	-1,566	-1,352	-1,172	-1,318	-1,243	PSMD10	26S proteasome non-ATPase regulatory subunit 10	nucleus	
ID1140	PSD13	-1,553	-1,845	-2,149	-1,650	-1,709	-1,617	PSMD13	proteasome (prosome, macropain) 26S subunit, non-ATPase, 13	cytoplasm	peptidase
ID1244	PSDE	-1,251	-1,582	-1,417	-2,482	-1,390	-1,386	PSMD14	proteasome (prosome, macropain) 26S subunit, non-ATPase, 14	cytoplasm	peptidase
ID566	PSMD2	-1,662	-2,073	-1,791	-1,473	-1,400	-1,207	PSMD2	proteasome (prosome, macropain) 26S subunit, non-ATPase, 2	cytoplasm	other
ID276677	PSMD4	-1,046	-1,978	-1,440	-2,848	-2,184	-1,321	PSMD4	proteasome (prosome, macropain) 26S subunit, non-ATPase, 4	cytoplasm	other
ID955	**PSMD5**, SH3G1	-1,905	-1,702	-1,213	-1,939	-1,688	-1,731	PSMD5	proteasome (prosome, macropain) 26S subunit, non-ATPase, 5	cytoplasm	other
ID954	**PSMD5**, SH3G1	-1,647	-1,869	-1,732	-1,982	-1,714	-1,521	PSMD5	proteasome (prosome, macropain) 26S subunit, non-ATPase, 5	cytoplasm	other
ID1433	**PSME1**, 6PGL	-1,083	-1,131	-1,074	1,516	-1,048	-1,229	PSME1	proteasome (prosome, macropain) activator subunit 1 (PA28 alpha)	cytoplasm	other
ID1392	PSME2	-1,837	-2,036	-1,916	-1,378	-1,584	-1,741	PSME2	proteasome (prosome, macropain) activator subunit 2 (PA28 beta)	cytoplasm	peptidase
ID3313934	**PSME2**, 1433E	-1,582	-1,876	-1,687	1,751	1,175	1,085	PSME2	proteasome (prosome, macropain) activator subunit 2 (PA28 beta)	cytoplasm	peptidase
ID1373	**PSME2**, 1433E	1,036	-1,031	1,033	1,822	-1,286	-1,410	PSME2	proteasome (prosome, macropain) activator subunit 2 (PA28 beta)	cytoplasm	peptidase
ID1409	**PSME2**, CLIC4	-2,173	-1,981	-1,980	-1,600	-1,623	-1,462	PSME2	proteasome (prosome, macropain) activator subunit 2 (PA28 beta)	cytoplasm	peptidase
ID1396	**PSME3**, GSTO1	-1,585	-1,513	-1,380	-1,334	-1,310	-1,238	PSME3	proteasome activator complex subunit 3	nucleus	
ID458579	PUR4	-1,313	-1,827	-1,449	-2,127	-1,705	-1,577	PFAS	phosphoribosylformylglycinamidine synthase	cytoplasm	enzyme
ID3435858	QCR1	-1,664	-2,568	-1,787	-2,026	11,944	1,390	UQCRC1	ubiquinol-cytochrome c reductase core protein I	cytoplasm	enzyme
ID714	RANB3	1,697	3,081	1,857	2,758	2,644	2,499	RANBP3	RAN binding protein 3	nucleus	other
ID1446	**RANG**, PSA3	-1,741	-1,816	-1,424	-1,659	-1,710	-1,732	RANBP1	RAN binding protein 1	nucleus	other
ID932	RBBP4	1,181	-1,041	1,067	-1,718	-1,453	-1,579	RBBP4	retinoblastoma binding protein 4	nucleus	enzyme
ID1586	RBM8A	1,020	-1,239	-1,167	-1,649	-1,359	-1,509	RBM8A	RNA binding motif protein 8A	nucleus	other
ID1604	RBM8A	-1,296	-2,048	-1,673	-2,254	-1,868	-4,104	RBM8A	RNA binding motif protein 8A	nucleus	other
ID280677	RCN1	1,131	1,252	1,300	1,997	1,140	-1,108	RCN1	reticulocalbin 1, EF-hand calcium binding domain	cytoplasm	other
ID2687300	RCN1	1,245	1,152	1,372	2,177	1,360	-1,005	RCN1	reticulocalbin 1, EF-hand calcium binding domain	cytoplasm	other
ID2662678	RCN1	1,158	-1,085	1,245	1,564	1,044	-1,076	RCN1	reticulocalbin 1, EF-hand calcium binding domain	cytoplasm	other
ID459	RCN1	-1,560	-2,420	-1,772	-2,173	-1,424	-1,502	RCN1	reticulocalbin 1, EF-hand calcium binding domain	cytoplasm	other
ID1127	**RCN1**, SPRC	-1,117	1,006	-1,054	1,176	-1,365	-1,662	RCN1	reticulocalbin 1, EF-hand calcium binding domain	cytoplasm	other
ID2680471	**RCN1**, SPRC	1,034	-1,006	1,138	1,552	-1,073	-1,283	RCN1	reticulocalbin 1, EF-hand calcium binding domain	cytoplasm	other
ID935	RD23A	-1,998	-2,415	-1,724	-1,847	-2,116	-1,842	RAD23A	RAD23 homolog A (S. cerevisiae)	nucleus	other
ID3128108	RD23B	2,070	4,470	2,249	3,360	3,881	2,705	RAD23B	UV excision repair protein RAD23 homolog B	nucleus	other
ID94683	RD23B	1,315	1,522	1,278	1,318	-1,093	-1,497	RAD23B	UV excision repair protein RAD23 homolog B	nucleus	other
ID1375	RFA2	-1,494	-1,793	-1,527	-2,399	-2,043	-1,959	RPA2	replication protein A2, 32kDa	nucleus	other
ID1146	RFC2	-1,616	-2,186	-1,798	-3733,614	-1,335	-1,505	RFC2	replication factor C (activator 1) 2, 40kDa	nucleus	other
ID851	RHG01	1,657	2,295	2,005	2,519	2,570	1,865	ARHGAP1	Rho GTPase activating protein 1	cytoplasm	other
ID922	**RHG01**, HNRH2	-1,500	-1,910	-1,774	-9,964	-1,665	-1,624	ARHGAP1	Rho GTPase activating protein 1	cytoplasm	other
ID1005	RINI	-3,040	-2,924	-2,110	-2,586	-2,028	-1,445	RNH1	ribonuclease/angiogenin inhibitor 1	cytoplasm	other
ID1156	**RSSA**, SET	-1,199	-1,412	-1,093	-1,154	-1,374	-1,709	RPSA	ribosomal protein SA	plasma membrane	transmembrane receptor
ID357613	**SAE1**, ACTG	-1,374	-1,775	-1,663	-1,501	-1,443	-1,413	SAE1	SUMO-activating enzyme subunit 1	nucleus	
ID1042	SAHH	-1,920	-2,027	-1,546	-1,708	-1,521	-1,307	AHCY	adenosylhomocysteinase	cytoplasm	enzyme
ID3228910	**SAKS1**, SAE1	-1,681	-1,758	-1,430	1,021	-1,083	1,084	UBXN1	UBX domain-containing protein 1	cytoplasm	
ID703	SCFD1	1,279	1,419	1,226	3,165	1,503	1,227	SCFD1	sec1 family domain containing 1	cytoplasm	transporter
ID1093	**SCMC1**, ILEU	-2,121	-2,521	-1,807	-1664,897	-1,582	-1,603	SLC25A24	solute carrier family 25 (mitochondrial carrier; phosphate carrier), member 24	cytoplasm	other
ID418384	**SCMC1**, TADBP	-1,015	-1,260	-1,020	-1,152	-2,059	-1,829	SLC25A24	solute carrier family 25 (mitochondrial carrier; phosphate carrier), member 24	cytoplasm	other
ID937	SCRN1	-2,138	-2,467	-1,729	-2,551	-1,882	-1,643	SCRN1	secernin 1	cytoplasm	other
ID697	**SDC10**, AGM1	1,424	1,565	1,426	1,833	2,571	1,815	CWC27	spliceosome-associated protein CWC27 homolog	nucleus	
ID921	**SEPT8**, HNRH1	-1,247	-1,605	-1,498	-1,813	-1,846	-1,684	SEPT8	septin 8	extracellular space	other

Table S-2

ID)1	Protein)2	0 min)3	30 min)3	60 min)3	24 h)3	48 h)3	72 h)3	IPA symbol)4	Entrez gene name)5	location)6	type(s))7
ID279386	SET	-1,323	-1,572	-1,507	-1,420	-1,252	-1,338	SET	SET nuclear oncogene	nucleus	phosphatase
ID1142	SET	-1,372	-1,875	-1,639	-1,593	-1,152	-1,438	SET	SET nuclear oncogene	nucleus	phosphatase
ID1157	SET	-1,603	-2,273	-1,512	-1,646	-1,631	-1,658	SET	SET nuclear oncogene	nucleus	phosphatase
ID90190	SET	-2,221	-6,412	-3,373	-1,646	-1,165	-1,405	SET	SET nuclear oncogene	nucleus	phosphatase
ID1052	**SET**, ADRM1	-2,342	-2,025	-2,396	-1,838	-1,554	-1,407	SET	SET nuclear oncogene	nucleus	phosphatase
ID2906459	**SET**, HDGF	-1,483	-1,610	-1,807	-2,423	-1,378	-1,407	SET	SET nuclear oncogene	nucleus	phosphatase
ID306795	**SET**, HDGF	-3,190	-3,540	-3,118	-5,583	-2,151	-1,558	SET	SET nuclear oncogene	nucleus	phosphatase
ID92002	**SET**, SPSY	-3,877	-2,284	-3,046	-1,512	-1,487	-1,342	SET	SET nuclear oncogene	nucleus	phosphatase
ID3760549	SF3A3	-1,001	-1,200	-1,529	-1,461	-1,537	-1,531	SF3A3	splicing factor 3A subunit 3	nucleus	other
ID9421	SF3B2	-1,237	1,022	-1,063	1,879	-1,007	-1,322	SF3B2	splicing factor 3b, subunit 2, 145kDa	nucleus	other
ID354	SF3B2	-1,550	-2,097	-2,136	-5,397	-1,717	-1,982	SF3B2	splicing factor 3b, subunit 2, 145kDa	nucleus	other
ID355	SF3B2	-1,573	-2,160	-2,096	-4,251	-1,868	-2,046	SF3B2	splicing factor 3b, subunit 2, 145kDa	nucleus	other
ID2995227	SFRS1	-1,002	-1,171	-1,103	-2,282	-1,476	-1,555	SRSF1	serine/arginine-rich splicing factor 1	nucleus	other
ID1565	SFRS3	1,036	-1,182	-1,360	-1,510	-1,677	-1,817	SFRS3	serine/arginine-rich splicing factor 3	nucleus	other
ID1572	SFRS3	1,207	-1,332	-1,129	-29,426	-2,104	-3,219	SFRS3	serine/arginine-rich splicing factor 3	nucleus	other
ID2287478	SFRS3	-1,345	-1,469	-1,306	-3,871	-2,315	-2,129	SFRS3	serine/arginine-rich splicing factor 3	nucleus	other
ID1536	SFRS3	-2,333	-2,110	-1,844	-1,349	-1,688	-1,503	SFRS3	serine/arginine-rich splicing factor 3	nucleus	other
ID1267	**SGTA**, HDGF	-1,768	-2,532	-2,038	-1,273	-1,160	-1,173	SGTA	small glutamine-rich tetratricopeptide repeat-containing protein alpha	nucleus	other
ID972	SHLB2	-1,581	-1,845	-1,420	-2,128	-1,769	-1,875	SH3GLB2	SH3-domain GRB2-like endophilin B2	cytoplasm	other
ID950	**SHLB2**, AL9A1	1,098	-1,039	-1,141	-1,743	-1,427	-1,099	SH3GLB2	SH3-domain GRB2-like endophilin B2	cytoplasm	other
ID408351	**SHLB2**, AL9A1	-1,756	-1,692	-1,555	-1,608	-1,592	-1,731	SH3GLB2	SH3-domain GRB2-like endophilin B2	cytoplasm	other
ID1628	SKP1	-1,097	-1,278	-1,103	-1,060	-1,369	-1,733	SKP1	S-phase kinase-associated protein 1	cytoplasm	other
ID1305	**SNAA**, SFRS1	-1,306	-1,329	-1,203	-1,715	-1,843	-1,655	SNAA	alpha-soluble NSF attachment protein		
ID956	**SNX6**, HNRH1	-1,694	-2,045	-1,673	-2,030	-1,646	-1,488	SNX6	sorting nexin 6	cytoplasm	transporter
ID508072	SODC	-1,726	-1,632	-1,353	-2,160	-1,789	-2,070	SOD1	superoxide dismutase 1, soluble	cytoplasm	enzyme
ID1651	SODC	-1,488	-1,680	-1,168	-1,337	-1,190	-1,502	SOD1	superoxide dismutase 1, soluble	cytoplasm	enzyme
ID30752	SODC	-2,400	-2,568	-1,708	-1,824	-1,875	-1,772	SOD1	superoxide dismutase 1, soluble	cytoplasm	enzyme
ID1589	SORCN	1,107	-1,076	-1,066	-2,058	-1,636	-1,658	SRI	sorcin	cytoplasm	transporter
ID2666660	SPRC	1,062	-1,231	1,214	1,363	-1,246	-1,676	SPARC	SPARC	extracellular space	other
ID278067	SPRC	-1,011	-1,479	1,365	1,046	-1,533	-1,875	SPARC	SPARC	extracellular space	other
ID1083	SPS1	-1,873	-2,245	-1,900	-1,651	-1,471	-1,583	SEPHS1	selenophosphate synthetase 1	unknown	enzyme
ID1121	**SPSY**, ARSA1	-2,367	-1,988	-2,088	-1,874	-1,737	-1,455	SMS	spermine synthase	unknown	enzyme
ID557	SRC8	-1,593	-2,231	-1,653	-2,059	-1,717	-1,639	CTTN	cortactin	plasma membrane	other
ID1638	SSRD	1,069	1,416	1,742	-1,881	-1,730	1,022	SSR4	translocon-associated protein subunit delta	cytoplasm	other
ID1660	SSRD	-1,087	-1,482	-1,092	1,737	-1,020	1,027	SSR4	translocon-associated protein subunit delta	cytoplasm	other
ID853	STABP	1,792	3,068	2,070	2,966	3,182	2,400	STAMBP	STAM binding protein	nucleus	enzyme
ID1553	**STAM1**, PSB9	-1,339	-1,742	-1,853	-1,119	-1,428	-1,441	STAM	signal transducing adaptor molecule (SH3 domain and ITAM motif) 1	cytoplasm	other
ID1119	**STML2**, ACTG	1,422	1,249	1,362	2,050	1,170	1,052	STOML2	stomatin (EPB72)-like 2	plasma membrane	other
ID1695	STMN1	-1,292	-1,711	-1,107	-1,582	-1,355	-1,749	STMN1	stathmin 1	cytoplasm	other
ID1689	STMN1	-1,480	-1,905	-1,241	-2,067	-1,842	-2,258	STMN1	stathmin 1	cytoplasm	other
ID1161	STRAP	-1,989	-2,206	-1,933	-1,859	-1,550	-1,292	STRAP	serine/threonine kinase receptor associated protein	plasma membrane	other
ID1058	**SUCB1**, IF34	-1,476	-1,774	-1,370	-2,335	-2,200	-1,821	SUCLA2	succinate-CoA ligase, ADP-forming, beta subunit	cytoplasm	enzyme
ID1214	SUGT1	-1,797	-2,352	-1,812	-1,382	-1,420	-1,245	SUGT1	protein SGT1 homolog	nucleus	other
ID144537	**SUGT1**, NUBP1	-1,598	-1,590	-1,260	-1,847	-1,842	-1,626	SUGT1	protein SGT1 homolog	nucleus	other
ID974	SYDC	-1,451	-1,745	-1,370	-1,483	-1,228	-1,051	DARS	aspartyl-tRNA synthetase	cytoplasm	enzyme
ID934	**SYDC**, HNRH1	-1,313	-1,702	-1,572	-116,442	-1,581	-1,677	DARS	aspartyl-tRNA synthetase	cytoplasm	enzyme
ID28319	**SYG**, CALD1	-1,312	-1,195	-1,367	2,222	-1,581	-1,173	GARS	glycyl-tRNA synthetase	cytoplasm	enzyme
ID673	**SYG**, DPYL2	-1,716	-1,719	-1,670	2,686	-1,409	-1,275	GARS	glycyl-tRNA synthetase	cytoplasm	enzyme
ID908	SYHC	-1,390	-1,392	-1,387	-2,288	-1,735	-1,530	HARS	histidyl-tRNA synthetase	cytoplasm	enzyme
ID377849	**SYHC**, VATB2	-1,113	-1,287	-1,136	-1,470	-1,566	-1,661	HARS	histidyl-tRNA synthetase	cytoplasm	enzyme
ID683	**SYK**, TRFL	1,495	2,130	1,624	2,642	2,158	1,648	KARS	lysyl-tRNA ligase	cytoplasm	enzyme
ID885	SYWC	-1,792	-1,739	-1,581	-1,778	-1,490	-1,435	WARS	tryptophanyl-tRNA synthetase	cytoplasm	enzyme
ID29100	**TADBP**, ACY1	-1,688	-1,653	-1,700	-2,163	-2,252	-1,915	TARDBP	TAR DNA binding protein	nucleus	transcription regulator
ID1084	**TADBP**, PPME1	-1,685	-2,324	-1,676	-2,124	-1,963	-1,521	TARDBP	TAR DNA binding protein	nucleus	transcription regulator
ID1270	TALDO	1,192	1,057	-1,051	-3,619	1,010	-1,128	TALDO1	transaldolase 1	cytoplasm	enzyme
ID1234	**TALDO**, PP1B	-1,582	-1,633	-1,533	-1,260	-1,204	-1,099	TALDO1	transaldolase 1	cytoplasm	enzyme
ID890	TBA1B	-1,836	-1,919	-1,672	-1,661	-1,768	-1,615	TUBA1B	tubulin, alpha 1b	cytoplasm	other
ID402687	TBA1B	-2,077	-3,308	-1,938	-2,069	-2,688	-2,378	TUBA1B	tubulin, alpha 1b	cytoplasm	other
ID904	**TBA1B**, TBB5	-1,102	1,169	-2,446	-1,493	-1,553	-1,022	TUBA1B	tubulin, alpha 1b	cytoplasm	other

Table S-2

ID[1]	Protein[2]	0 min[3]	30 min[3]	60 min[3]	24 h[3]	48 h[3]	72 h[3]	IPA symbol[4]	Entrez gene name[5]	location[6]	type(s)[7]
ID906	**TBA1C**, PTRF	-1,145	1,033	-1,452	-1,705	-1,618	-1,326	TUBA1C	tubulin, alpha 1c	cytoplasm	other
ID2200	TBA3	1,755	2,616	2,151	2,745	3,052	2,244	TUBA1A	tubulin alpha-1A chain	cytoskeleton	other
ID15904	TBAK	1,452	1,271	-1,066	-1,068	-1,388	-1,544	TUBA1B	tubulin, alpha 1b	cytoplasm	other
ID876	TBAK	-2,146	-1,833	-2,055	-1,671	-1,757	-1,748	TUBA1B	tubulin, alpha 1b	cytoplasm	other
ID3111980	TBB2C	-2,161	-1,848	-1,825	-1,474	-1,516	-1,661	TUBB2C	tubulin, beta 2C	cytoplasm	other
ID875	**TBB2C**, TBB3	-1,898	-1,538	-1,460	-1,839	-1,523	-1,401	TUBB2C	tubulin, beta 2C	cytoplasm	other
ID3104	**TBB2C**, TBB6	-1,692	-1,660	-1,495	-1,542	-1,240	-1,336	TUBB2C	tubulin, beta 2C	cytoplasm	other
ID3150	**TBB3**, TBB2C	-1,834	-1,714	-1,624	-1,189	-1,208	-1,337	TUBB3	tubulin, beta 3	cytoplasm	other
ID93790	**TBB5**, TBB2C	-1,085	-1,312	-1,085	-2,286	-2,339	-1,958	TUBB	tubulin, beta	cytoplasm	other
ID1780	TBCA	-1,599	-1,843	-1,315	-1,207	-1,190	-1,180	TBCA	tubulin-specific chaperone A	cytoskeleton	other
ID973	**TBG1**, MPPB	-1,399	-1,605	-1,513	-2,000	-1,377	-1,458	TUBG1	tubulin, gamma 1	cytoplasm	other
ID29990	TCPA	1,469	2,113	1,591	-1,007	1,258	1,057	TCPA	t-complex 1	cytoplasm	other
ID793	TCPA	-1,664	-1,922	-1,658	-1,683	-1,400	-1,173	TCPA	t-complex 1	cytoplasm	other
ID888	**TCPB**, RUVB1	-1,429	-1,655	-1,482	-2,786	-1,355	-1,234	CCT2	chaperonin containing TCP1, subunit 2 (beta)	cytoplasm	kinase
ID804	TCPE	-1,058	-1,498	-1,341	-1,221	-1,188	-1,234	CCT5	chaperonin containing TCP1, subunit 5 (epsilon)	cytoplasm	other
ID405373	**TCPE**, FKBP4	-1,213	-1,506	-1,411	-1,338	-1,138	-1,228	CCT5	chaperonin containing TCP1, subunit 5 (epsilon)	cytoplasm	other
ID847	**TCPE**, HNRPK	-1,192	-1,548	-1,374	-1,459	-1,367	-1,545	CCT5	chaperonin containing TCP1, subunit 5 (epsilon)	cytoplasm	other
ID771	TCPG	-1,080	-1,133	-1,099	-13,343	-1,222	-1,193	CCT3	chaperonin containing TCP1, subunit 3 (gamma)	cytoplasm	other
ID760	TCPG	-1,354	-1,578	-1,441	-2,265	-1,407	-1,342	CCT3	chaperonin containing TCP1, subunit 3 (gamma)	cytoplasm	other
ID796	**TCPQ**, HNRPK	1,012	-1,564	-1,640	-1,591	-1,367	-1,444	CCT8	chaperonin containing TCP1, subunit 8 (theta)	cytoplasm	enzyme
ID1576	TCTP	-1,088	-1,360	-1,198	-1,557	-1,433	-1,452	TPT1	tumor protein, translationally controlled 1	cytoplasm	other
ID1501	**TCTP**, CBX1	-1,257	-1,564	-1,241	-1,163	-1,236	-1,089	TPT1	tumor protein, translationally controlled 1	cytoplasm	other
ID1610	TEBP	-1,615	-1,654	-1,655	-2,095	-1,585	-2,546	PTGES3	prostaglandin E synthase 3 (cytosolic)	cytoplasm	enzyme
ID2310	TEBP	-1,683	-1,734	-1,764	-2,314	-1,489	-2,108	PTGES3	prostaglandin E synthase 3 (cytosolic)	cytoplasm	enzyme
ID540	TERA	-1,366	-1,557	-1,527	-2,055	-1,632	-1,282	VCP	valosin-containing protein	cytoplasm	enzyme
ID542	TERA	-1,288	-1,664	-1,376	-1,581	-1,309	-1,354	VCP	valosin-containing protein	cytoplasm	enzyme
ID9875	TERA	-1,567	-2,076	-1,744	-2,208	-1,772	-1,533	VCP	valosin-containing protein	cytoplasm	enzyme
ID1821	**THIO**, RFA3, LEG1	-1,496	-1,454	-1,281	1,712	-1,041	1,981	TXN	thioredoxin	nucleus	
ID1115	THOC3	-1,249	-1,355	-1,195	-1,590	-1,235	-1,517	THOC3	THO complex 3	nucleus	other
ID607	THOP1	1,073	-1,311	-1,043	-1,423	-1,382	-1,663	THOP1	thimet oligopeptidase	unknown	
ID488	TIF1B	-1,397	-2,674	-2,446	-2,990	-1,898	-1,879	TRIM28	tripartite motif-containing 28	nucleus	transcription regulator
ID1419	**TIF1B**, 1433G	-1,686	-1,744	-1,678	-1,325	-1,461	-1,427	TRIM28	tripartite motif-containing 28	nucleus	transcription regulator
ID479	**TIF1B**, HNRPU	-1,848	-2,248	-2,037	-5,236	-2,168	-1,668	TRIM28	tripartite motif-containing 28	nucleus	transcription regulator
ID774	TM1L2	1,827	3,277	1,740	3,835	3,222	2,408	TOM1L2	TOM1-like protein 2	unknown	transporter
ID3085623	TMOD3	-1,702	-1,873	-1,553	-1,042	-1,063	-1,403	TMOD3	tropomodulin-3	unknown	
ID1315	TPM1	1,033	-1,207	-1,030	-1,519	-2,130	-1,927	TPM1	tropomyosin 1 (alpha)	cytoplasm	other
ID55610	**TPM1**, TPM2	1,051	-1,360	-1,086	-2,038	-2,705	-2,380	TPM1	tropomyosin 1 (alpha)	cytoplasm	other
ID87491	TPM2	-1,739	-1,394	-1,462	-1,094	-1,480	1,048	TPM2	tropomyosin 2 (beta)	cytoplasm	other
ID1210	TPM2	-1,018	-1,432	-1,156	-1,512	-1,496	-1,185	TPM2	tropomyosin 2 (beta)	cytoplasm	other
ID56308	TPM2	-1,375	-1,667	-1,428	-1,631	-1,516	-1,318	TPM2	tropomyosin 2 (beta)	cytoplasm	other
ID1372	**TPM3**, TPM4	-1,567	-1,784	-1,518	-1,738	-1,489	-1,339	TPM3	tropomyosin 3	cytoplasm	other
ID1369	TPM4	-1,227	-1,507	-1,328	-1,540	-1,516	-1,410	TPM4	tropomyosin 4	cytoplasm	other
ID1153	**TRA2B**, CSN4	-2,079	-2,494	-1,890	-1,503	-1,425	-1,367	TRA2B	transformer-2 protein homolog beta	nucleus	
ID688	TRFL	1,665	2,537	1,932	1,743	2,541	2,003	LTF	lactotransferrin	extracellular space	peptidase
ID751	TRXR1	1,901	3,635	1,900	3,353	3,520	2,715	TXNRD1	thioredoxin reductase 1	cytoplasm	enzyme
ID837	TRXR1	-1,190	-1,094	-1,174	-7,868	-1,008	1,086	TXNRD1	thioredoxin reductase 1	cytoplasm	enzyme
ID849	TRXR1	-1,377	-1,450	-1,371	-4,368	-1,193	-1,063	TXNRD1	thioredoxin reductase 1	cytoplasm	enzyme
ID1335	TSNAX	1,271	1,429	1,332	-1,118	1,518	1,168	TSNAX	translin-associated factor X	nucleus	transporter
ID203683	**TXND5**, ACL6A	1,179	1,329	1,223	2,696	1,369	1,680	TXNDC5	thioredoxin domain containing 5 (endoplasmic reticulum)	cytoplasm	enzyme
ID992	**TXND5**, ACL6A	1,091	1,040	1,028	-1,746	-1,934	-1,472	TXNDC5	thioredoxin domain containing 5 (endoplasmic reticulum)	cytoplasm	enzyme
ID977	**TXND5**, HNRPF	1,346	1,143	1,305	1,156	-1,392	-1,519	TXNDC5	thioredoxin domain containing 5 (endoplasmic reticulum)	cytoplasm	enzyme
ID13083	**TXND5**, SH3G1	-2,650	-2,873	-1,740	-1,759	-1,771	-1,643	TXNDC5	thioredoxin domain containing 5 (endoplasmic reticulum)	cytoplasm	enzyme
ID1269	**TXNL1**, ANXA2	-1,460	-1,717	-1,297	-1,328	-1,273	-1,121	TXNL1	thioredoxin-like protein 1	nucleus	
ID16669	TXNL5	-1,852	-1,882	-1,417	-1,459	-1,314	2,131	TXNDC17	thioredoxin domain containing 17	cytoplasm	enzyme
ID1358	**UBA1**, 3HIDH	-1,407	-1,453	-1,337	-1,897	-2,149	-2,097	UBA1	ubiquitin-like modifier activating enzyme 1	cytoplasm	enzyme
ID478	**UBA1**, TIF1B	-1,643	-2,179	-1,969	-2,487	-1,874	-1,787	UBA1	ubiquitin-like modifier activating enzyme 1	cytoplasm	enzyme
ID469	**UBA1**, TIF1B	-1,807	-2,550	-2,240	-2,363	-1,868	-1,613	UBA1	ubiquitin-like modifier activating enzyme 1	cytoplasm	enzyme
ID3542	UBA5	-1,372	-1,823	-1,545	-3,009	-1,991	-1,834	UBA5	ubiquitin-like modifier activating enzyme 5	cytoplasm	enzyme
ID1116	UBCP1	-1,718	-1,920	-1,619	-3,108	-1,272	-1,322	UBLCP1	ubiquitin-like domain containing CTD phosphatase 1	unknown	phosphatase

Table S-2

ID)[1]	Protein)[2]	0 min)[3]	30 min)[3]	60 min)[3]	24 h)[3]	48 h)[3]	72 h)[3]	IPA symbol)[4]	Entrez gene name)[5]	location)[6]	type(s))[7]
ID554833	UBE1	-1,924	-2,603	-2,309	-3,087	-2,068	-1,788	UBA1	ubiquitin-like modifier activating enzyme 1	cytoplasm	enzyme
ID466	**UBE1**, MVP, UBA1	-2,128	-2,435	-2,096	-2,666	-1,815	-1,562	UBA2	ubiquitin-like modifier activating enzyme 2	cytoplasm	enzyme
ID818	UBP14	-1,884	-2,057	-1,777	-1,463	-1,428	-1,260	USP14	ubiquitin specific peptidase 14 (tRNA-guanine transglycosylase)	cytoplasm	peptidase
ID545	UBP5	-2,267	-2,503	-1,967	-1,739	-1,551	-1,388	USP5	ubiquitin specific peptidase 5 (isopeptidase T)	cytoplasm	peptidase
ID550	**UBP5**, P3H1	-2,314	-2,584	-2,106	-2,026	-1,722	-1,609	USP5	ubiquitin specific peptidase 5 (isopeptidase T)	cytoplasm	peptidase
ID764	UBQL1	1,817	3,644	2,037	4,243	4,269	3,136	UBQLN1	ubiquilin 1	cytoplasm	other
ID1491	UCHL1	-1,286	-1,335	-1,164	-1,509	-1,309	-1,132	UCHL1	ubiquitin carboxyl-terminal hydrolase isozyme L1	cytoplasm	enzyme
ID809	ULA1	-1,828	-2,181	-2,238	-1,844	-1,627	-1,454	NAE1	NEDD8 activating enzyme E1 subunit 1	cytoplasm	enzyme
ID201269	**UQCR1**, HNRPK	-6,335	-6,081	-6,065	-2,112	-2,464	-2,063	UQCRC1	ubiquinol-cytochrome c reductase core protein I	cytoplasm	enzyme
ID1031	VAT1	-1,261	-1,610	-1,595	-38,976	-1,528	-1,344	VAT1	vesicle amine transport protein 1 homolog (T. californica)	plasma membrane	transporter
ID1044	VIME	2,021	1,445	2,325	1,193	1,354	1,489	VIM	vimentin	cytoplasm	other
ID975	VIME	1,611	1,116	1,345	1,054	-1,002	1,344	VIM	vimentin	cytoplasm	other
ID432	VINC	-1,387	-1,696	-1,471	-2,091	-1,565	-1,502	VCL	vinculin	plasma membrane	enzyme
ID431	VINC	-1,251	-1,719	-1,659	-2,122	-1,815	-1,784	VCL	vinculin	plasma membrane	enzyme
ID433	VINC	-1,542	-1,879	-1,726	-483,662	-1,828	-1,466	VCL	vinculin	plasma membrane	enzyme
ID2117397	VINC	-1,835	-1,932	-1,603	-6,743	-33,535	-12,949	VCL	vinculin	plasma membrane	enzyme
ID1325	**WDR61**, ANXA5	-1,056	-1,133	-1,139	-1,358	-1,541	-1,666	WDR61	WD repeat-containing protein 61	nucleus	other
ID3403522		4,392	43,135	3,884	-5,293	1,110	1,102				
ID140457		5,832	28,192	3,773	-1,660	-1,688	-1,427				
ID2728784		9,852	19,044	5,490	1,229	2,525	1,248				
ID1623		1,753	18,442	4,971	-29,164	-10,264	-1,846				
ID2722863		13,379	18,382	9,646	-16,672	1,388	-9,593				
ID3763473		2,705	17,130	3,716	-1,665	1,470	1,054				
ID1915149		14,907	14,980	8,517	-2,318	35,333	1,354				
ID582		8,367	13,063	16,629	3,516	3,185	-2,027				
ID1721		1,580	12,368	2,232	-1,700	1,700	3,906				
ID1814025		99,866	9,002	3,374	3,308	n.d.	-2,634				
ID343050		2,206	8,228	2,982	-1,624	-1,082	1,163				
ID1212		3,914	7,686	6,210	1,494	4,287	2,379				
ID1719		2,516	7,627	2,281	-1,330	-1,035	-1,543				
ID1987569		2,566	7,326	5,327	-3,432	-1,582	3,597				
ID581		3,083	7,272	3,438	3,641	3,983	-4,728				
ID2142601		4,095	6,712	2,015	1,392	-1,054	-13,536				
ID1811207		7,264	6,416	4,375	1,747	10,757	2,179				
ID2202941		8,074	5,849	9,939	-2,139	1,225	-2,080				
ID730		1,666	5,695	1,855	2,252	1,851	6,437				
ID1846930		2,438	5,619	6,523	1,961	-1,232	1,926				
ID3549612		2,715	5,178	3,080	4,840	4,092	3,675				
ID1559		6,268	4,953	7,207	2,480	1,540	1,206				
ID337908		1,359	4,877	1,857	2,206	3,360	2,010				
ID587		4,752	4,670	7,982	2,106	1,997	-1,632				
ID3091679		13,220	4,566	-1,008	2,050	-1,027	-1,556				
ID2729506		2,059	4,563	2,031	6,326	5,512	3,242				
ID42780		1,936	4,549	2,026	3,993	3,365	3,392				
ID750		1,988	4,352	1,950	2,805	3,342	4,007				
ID43156		2,030	4,334	2,340	5,660	3,806	2,934				
ID132415		2,158	4,057	2,440	4,151	4,233	3,634				
ID1082		8,338	3,962	2,583	1,273	-1,064	-1,424				
ID754		2,015	3,928	2,154	3,090	3,136	2,923				
ID1802534		1,631	3,896	1,610	1,803	1,754	-1,489				
ID3479720		2,427	3,892	3,617	4,202	7,588	2,626				
ID742		1,640	3,887	2,055	1,783	1,312	2,025				
ID776		1,832	3,881	2,042	4,819	4,482	3,459				
ID1739		1,267	3,825	1,861	-10,252	-1,492	-3,014				
ID1338		1,930	3,773	2,116	-5,356	5,338	2,847				
ID135817		2,166	3,733	1,881	-1,378	-1,226	1,128				
ID3179		1,695	3,710	2,061	3,484	3,226	2,696				
ID2158110		3,651	3,602	9,767	1,119	-1,165	-1,208				
ID1475		2,317	3,562	2,237	1,560	1,164	1,590				

Table S-2

ID[1]	Protein[2]	0 min[3]	30 min[3]	60 min[3]	24 h[3]	48 h[3]	72 h[3]	IPA symbol[4]	Entrez gene name[5]	location[6]	type(s)[7]
ID29087		1,828	3,552	1,952	3,993	3,804	2,723				
ID40401		1,752	3,439	1,991	3,933	3,284	2,931				
ID41097		1,805	3,348	2,012	4,297	3,560	1,919				
ID749		2,015	3,308	1,978	2,237	2,361	2,211				
ID756		1,853	3,280	1,991	2,817	2,871	2,339				
ID147614		7,479	3,278	1,231	1,107	1,051	-2,016				
ID1799192		1,633	3,235	1,789	2,325	1,962	-1,386				
ID578		2,545	3,188	3,045	1,024	-1,229	-1,385				
ID1720		-2,343	3,126	1,115	-6,148	-31,562	-52,177				
ID237648		1,723	3,078	1,736	3,113	3,162	1,251				
ID1800806		-4,605	3,060	-1,694	n.d.	-168,126	-4,456				
ID713		1,623	3,048	1,775	3,185	3,287	2,489				
ID17707		1,338	3,004	1,722	2,386	2,164	1,090				
ID719		1,767	2,993	1,812	2,512	2,139	2,399				
ID1761		1,350	2,958	1,037	2,203	1,242	-1,686				
ID785		1,916	2,954	2,348	3,782	3,821	2,551				
ID732		1,527	2,948	1,902	1,782	1,346	-1,905				
ID782		1,737	2,945	1,695	2,956	3,084	2,361				
ID266132		1,719	2,942	1,744	1,642	1,821	2,232				
ID2889441		3,990	2,898	4,331	1,368	1,573	-1,314				
ID102094		1,422	2,860	1,544	4,351	3,326	2,716				
ID866		2,086	2,857	1,957	-2,070	1,692	1,620				
ID3761682		1,332	2,850	2,055	-1,020	1,675	-1,739				
ID769		1,722	2,847	1,844	3,764	3,634	2,757				
ID731		1,577	2,835	1,636	1,581	1,125	-2,224				
ID1843456		2,317	2,826	2,086	-2,778	1,777	8,005				
ID416		1,687	2,816	1,802	2,024	3,426	1,286				
ID773		1,614	2,783	1,549	2,739	2,585	2,093				
ID916		1,953	2,772	2,169	3,850	3,682	2,689				
ID841		1,984	2,760	2,222	4,307	4,020	2,805				
ID2150207		3,835	2,735	3,040	-1,519	2,237	-2,693				
ID7425		1,811	2,684	1,786	1,945	2,101	1,118				
ID2183		1,739	2,664	1,991	2,508	2,795	2,172				
ID1466		2,751	2,655	3,516	2,020	1,820	1,521				
ID759		1,675	2,642	2,113	3,467	3,413	2,409				
ID1566		1,402	2,626	2,628	1,028	1,871	1,337				
ID124300		1,409	2,607	1,517	3,086	2,491	1,780				
ID858		1,680	2,582	1,936	2,696	2,651	2,043				
ID413		1,648	2,575	1,732	3,035	3,111	1,675				
ID748		1,711	2,572	2,095	3,674	3,848	2,716				
ID352255		1,758	2,567	1,856	1,735	2,491	2,279				
ID407		1,774	2,562	1,823	2,296	2,142	1,272				
ID2148299		3,260	2,553	2,344	-1,531	1,753	-3,077				
ID3483954		1,666	2,486	2,100	2,486	2,359	1,869				
ID41789		1,531	2,480	1,722	2,332	1,897	-1,020				
ID1844173		2,203	2,477	2,683	2,209	2,664	2,303				
ID1683		23,914	2,470	8,518	3,232	7,080	6,128				
ID265686		1,477	2,462	1,350	1,838	2,493	3,473				
ID1841741		1,535	2,453	1,580	1,238	2,699	5,453				
ID775		1,514	2,430	1,437	2,817	2,394	1,883				
ID2033833		1,908	2,387	1,905	2,231	2,363	-1,655				
ID1464		-1,572	2,373	-1,422	-4,499	1,013	2,351				
ID246907		1,023	2,362	-1,317	16,713	2,678	1,857				
ID3485928		2,168	2,361	2,062	3,493	1,673	1,242				
ID747		1,621	2,349	1,923	3,694	3,944	2,731				
ID3477702		1,711	2,348	2,109	3,873	4,575	2,653				
ID7589		1,724	2,336	1,594	2,236	1,795	-1,004				
ID509563		-1,675	2,304	1,314	n.d.	-2,201	-1,103				
ID412		1,491	2,298	1,588	2,754	2,453	1,358				

Table S-2

ID)1	Protein)2	0 min)3	30 min)3	60 min)3	24 h)3	48 h)3	72 h)3	IPA symbol)4	Entrez gene name)5	location)6	type(s))7
ID1814782		-1,067	2,274	2,279	3,383	3,541	2,763				
ID1490		-1,558	2,271	-3,098	-1,680	1,189	3,727				
ID802		1,643	2,251	2,069	2,905	2,149	1,373				
ID108562		1,535	2,247	1,521	1,823	1,702	2,240				
ID419		1,503	2,237	1,532	3,009	2,780	1,315				
ID1178		1,054	2,226	-1,345	1,073	2,756	1,213				
ID2108161		-1,391	2,193	-1,281	-2,333	-10,038	-3,816				
ID238040		1,347	2,180	1,591	1,878	1,927	1,313				
ID2330		2,279	2,175	2,390	-1,231	1,529	1,638				
ID2209869		2,330	2,151	2,032	1,130	-1,322	-1,191				
ID2997219		1,358	2,136	1,584	-1,875	1,058	-1,435				
ID722		1,650	2,130	1,786	3,789	4,180	2,727				
ID3762296		1,538	2,121	1,829	2,369	2,677	1,896				
ID1532		-1,115	2,109	-1,215	-18,491	-2,625	-13,015				
ID3761695		3,580	2,096	4,576	-1,464	1,414	-1,913				
ID1451		1,058	2,079	1,128	-11,287	-2,711	-77,659				
ID418		1,461	2,077	1,545	2,966	3,036	1,503				
ID268600		5,843	2,076	4,300	1,512	1,097	-2,462				
ID1193		6,969	2,072	-1,138	-4,526	1,324	1,810				
ID2035512		-1,032	2,042	-1,342	-1,250	1,513	-2,684				
ID185509		1,805	2,039	1,824	-1,308	-1,426	-1,801				
ID267419		1,570	2,036	-1,065	1,125	1,248	2,183				
ID394		1,405	2,016	1,587	2,099	1,745	1,140				
ID3413662		1,862	2,009	1,020	1,013	-1,185	-2,054				
ID3078712		1,982	1,987	2,964	1,208	1,199	1,548				
ID2880272		3,348	1,974	2,597	1,706	-1,138	-2,471				
ID1871260		-1,129	1,913	-1,828	-2,807	1,339	-1,031				
ID1470		1,037	1,884	-1,524	-7,285	-6,953	-14,322				
ID1867746		1,024	1,883	-1,050	-1,053	2,823	1,606				
ID2340727		-1,071	1,857	-1,073	-1,822	1,199	1,273				
ID2037342		1,532	1,839	1,322	1,061	1,114	-2,165				
ID43845		1,495	1,814	1,749	2,082	1,147	-1,003				
ID1094		1,483	1,813	-2,530	1,065	-1,731	-2,011				
ID392		1,298	1,811	1,527	1,847	1,648	1,038				
ID3763073		1,664	1,782	1,790	-1,044	-1,170	-2,099				
ID2990527		1,475	1,780	1,571	-1578,241	-19972,176	n.d.				
ID1840030		1,254	1,770	1,304	1,798	2,428	8,817				
ID39482		1,447	1,754	1,368	2,098	2,339	1,145				
ID1989367		1,502	1,751	1,596	1,626	-1,019	1,887				
ID423		1,212	1,704	1,382	1,825	1,581	1,125				
ID3762817		1,290	1,700	1,066	1,116	1,303	-1,292				
ID1795		1,155	1,685	-1,114	1,234	-1,338	-1,661				
ID9044		1,207	1,676	1,344	1,930	1,754	1,122				
ID987		-2,996	1,668	1,172	-1,930	-1,348	-1,303				
ID258601		1,268	1,616	2,231	-2,363	-1,812	-1,485				
ID729		1,561	1,614	1,348	1,856	1,501	1,355				
ID1520		1,581	1,603	1,997	1,104	-1,003	-1,147				
ID12822		1,141	1,595	1,245	-1,554	1,059	1,384				
ID3497145		1,418	1,540	1,317	-2,990	-4,855	-18,080				
ID49585		1,407	1,528	1,636	1,633	1,815	1,456				
ID3762592		1,820	1,523	1,318	-1,117	-1,140	-1,159				
ID45562		1,487	1,515	1,332	1,284	1,428	1,081				
ID181505		1,440	1,514	1,251	1,378	1,094	1,151				
ID3499167		1,411	1,501	1,278	n.d.	n.d.	n.d.				
ID1410		-1,051	1,492	-1,294	-1,403	-2,018	-2,271				
ID2214		1,202	1,488	1,272	-2,119	-1,471	-1,807				
ID3170569		1,660	1,480	1,429	1,160	-1,173	-1,166				
ID898		1,206	1,471	1,357	1,785	1,594	1,289				
ID1814733		1,090	1,458	1,466	2,797	-1,305	-2,191				

Table S-2

ID[1]	Protein[2]	0 min[3]	30 min[3]	60 min[3]	24 h[3]	48 h[3]	72 h[3]	IPA symbol[4]	Entrez gene name[5]	location[6]	type(s)[7]
ID3062814		1,183	1,456	1,115	-1,112	-1,789	-1,675				
ID1600		1,017	1,454	1,053	-1,565	-1,556	-1,086				
ID715		1,446	1,449	1,278	2,978	3,232	2,431				
ID589226		1,260	1,424	1,516	1,264	1,803	1,331				
ID406		1,305	1,379	1,282	1,238	1,565	1,171				
ID1570		1,078	1,377	1,267	-4,577	1,823	1,097				
ID437		1,061	1,376	1,137	1,580	1,572	1,016				
ID307		1,848	1,366	n.d.	n.d.	-1,645	-3,182				
ID11611		2,381	1,343	3,269	-1,235	-2,506	-6,119				
ID1187		1,519	1,336	1,237	1,049	1,107	1,181				
ID3432480		-1,034	1,330	1,024	2,429	2,097	1,878				
ID3763695		5,514	1,330	9,614	1,979	1,421	2,549				
ID57776		1,337	1,329	1,369	2,070	1,216	-1,034				
ID431675		1,905	1,329	2,193	4,011	1,649	1,438				
ID1985723		2,107	1,323	1,146	-2,818	-2,297	-1,886				
ID3765751		1,138	1,322	-1,142	-1,217	-1,546	-1,311				
ID2354041		1,248	1,312	1,337	1,689	1,005	-1,005				
ID1980208		1,117	1,309	1,582	-8,343	-1,028	-2,075				
ID1821608		1,172	1,306	1,203	1,697	1,426	-1,145				
ID1313		1,161	1,301	1,445	-8,502	-8,577	-65,885				
ID1782		-1,364	1,267	-1,383	1,596	-1,892	-1,807				
ID1994828		1,404	1,251	1,227	-2,373	1,301	6,608				
ID74132		1,133	1,243	-1,036	2,841	-1,044	-1,115				
ID3163353		1,910	1,234	1,233	-1,144	-1,365	-1,462				
ID500		-1,637	1,228	-1,181	-1,610	-2,134	-2,278				
ID92901		1,523	1,227	1,235	2,116	-1,168	1,037				
ID1059		1,269	1,221	1,423	1,128	-2,543	-3,069				
ID546917		-1,031	1,214	-1,100	1,578	1,061	-1,482				
ID1026		1,524	1,211	1,677	-2,444	-3,045	-3,937				
ID2091139		1,140	1,205	1,237	-1,585	-1,053	1,671				
ID2649893		1,254	1,196	1,332	-1,223	-1,340	-1,805				
ID1654		1,269	1,177	1,239	-1,885	-1,271	-1,285				
ID1499		1,114	1,172	-1,208	-2,337	-1,778	-1,266				
ID2617667		-1,182	1,165	-1,247	-1,101	-2,310	-1,012				
ID61022		1,454	1,164	1,597	-1,302	1,183	1,276				
ID1096		-1,047	1,148	-1,163	-1,277	-1,297	-1,794				
ID2152149		1,557	1,137	1,476	-1,137	-1,074	-1,402				
ID1816422		-2,229	1,130	1,638	1,941	-4,945	-4,296				
ID3668269		1,381	1,127	1,255	-1,723	-1,738	-2,914				
ID71445		1,142	1,126	1,296	3,880	2,761	2,765				
ID281947		-1,025	1,116	2,345	1,235	1,165	-1,105				
ID3763800		-1,103	1,113	-1,017	-9,536	-5,009	-19,511				
ID1590		1,037	1,105	-1,013	-2,592	-1,059	1,481				
ID2740977		1,236	1,100	1,102	2,018	1,339	1,057				
ID27793		-1,189	1,095	-1,126	-2,368	-1,628	-1,202				
ID996		1,970	1,094	2,296	-1,404	-2,139	-1,362				
ID3275754		1,602	1,082	-1,050	-7,637	-2,563	-7,580				
ID1819121		-1,020	1,078	1,159	2,596	1,517	-1,281				
ID1028		-1,847	1,060	1,209	1,417	1,163	1,104				
ID1983973		1,135	1,056	1,352	1,125	-1,342	-1,846				
ID1707		-1,041	1,056	-1,123	-35,643	-1,619	-5,869				
ID1564		-3,532	1,049	-5,837	-8,203	-4,039	-10,298				
ID733		1,700	1,049	3,042	-5,507	1,089	-2,196				
ID193389		1,476	1,045	1,484	1,258	-1,079	-1,531				
ID24119		1,018	1,044	-1,020	-1,525	-1,443	-2,133				
ID1514		-1,089	1,033	-1,014	-2,063	1,314	-1,006				
ID351055		1,480	1,027	1,210	1,730	1,002	-1,509				
ID444662		1,080	1,004	-1,011	3,536	1,413	1,886				
ID3763676		1,069	1,004	1,661	-2,489	-2,097	-2,973				

Table S-2

ID)[1]	Protein)[2]	0 min)[3]	30 min)[3]	60 min)[3]	24 h)[3]	48 h)[3]	72 h)[3]	IPA symbol)[4]	Entrez gene name)[5]	location)[6]	type(s))[7]
ID576672		1,005	-1,010	-1,296	-3,383	-2,042	1,048				
ID595		-1,096	-1,013	-1,006	-2,067	2,911	2,125				
ID401246		-1,025	-1,024	1,209	1,679	1,562	-1,199				
ID1847793		-1,053	-1,025	1,210	-1,988	-4,699	-3,599				
ID200032		1,219	-1,026	1,287	1,028	-1,222	-1,587				
ID2100931		-1,427	-1,026	-1,248	7,736	3,043	1,448				
ID211213		-1,032	-1,030	-1,037	2,544	-1,045	1,100				
ID112515		1,039	-1,030	-1,138	-2,567	-2,181	-1,887				
ID16882		1,046	-1,033	1,172	-1,092	-1,231	-1,519				
ID3763684		-1,250	-1,040	-1,117	-1,185	-1,301	-1,633				
ID1135		-1,202	-1,044	-1,624	-1,491	-1,482	-1,420				
ID3046761		-1,160	-1,046	-1,146	-1,339	-1,843	-1,399				
ID1935188		1,261	-1,053	1,119	-1,188	-1,219	2,088				
ID1197		1,141	-1,053	-1,121	-1,841	-1,267	-1,399				
ID62715		1,087	-1,056	1,071	-1,561	-1,304	-1,414				
ID473357		-1,299	-1,066	-1,193	-4,971	1,291	1,335				
ID1461		-1,069	-1,068	1,036	-1,983	-1,018	-1,641				
ID568037		-1,003	-1,069	-1,165	-1,599	-1,744	-1,125				
ID290046		1,509	-1,070	1,057	-1,479	1,120	-1,487				
ID196961		-1,230	-1,070	-1,353	-1,748	-1,529	-1,715				
ID1864541		-1,721	-1,075	-1,267	-2,750	-6,661	-3,245				
ID2068580		1,104	-1,077	-1,018	1,367	-1,329	-1,977				
ID3022983		1,070	-1,078	-1,003	-1,448	-4,418	-4,177				
ID1837103		1,043	-1,081	-1,272	1,811	1,766	5,773				
ID320752		1,177	-1,083	-1,197	-1,697	-1,381	-1,289				
ID7106		1,075	-1,088	1,078	2,255	1,371	-1,360				
ID323167		1,055	-1,088	-1,331	-1,576	-1,548	-1,573				
ID2115505		-1,033	-1,093	-1,345	-1,980	-1,324	-2,785				
ID543746		1,191	-1,096	-1,290	-1,673	-1,375	-1,362				
ID2350362		-1,024	-1,098	-1,008	1,227	-1,358	-1,831				
ID1664		-1,061	-1,099	-1,156	1,253	-1,287	-1,513				
ID2102478		-1,249	-1,099	-1,030	2,699	-1,845	-1,390				
ID157378		1,102	-1,114	1,036	-1,515	-1,442	-1,067				
ID1486		-1,522	-1,121	-1,339	-2,032	1,244	2,417				
ID523641		-1,217	-1,140	-1,188	-1,694	1,118	-1,119				
ID1286		1,192	-1,144	-1,238	-1,152	-1,308	-1,534				
ID3261240		-1,055	-1,147	-1,040	-1,840	-1,401	-1,522				
ID1229		-1,005	-1,148	1,019	-1,717	-1,415	-1,008				
ID426		1,006	-1,153	1,061	-1,622	1,042	-1,266				
ID1036		-1,102	-1,154	-1,262	1,088	-1,677	-1,517				
ID1978481		1,410	-1,156	-1,213	1,515	1,782	1,131				
ID11367		-1,040	-1,157	-1,017	-1,516	-1,712	-1,866				
ID2215889		1,026	-1,157	-1,269	-2,569	-1,793	-2,046				
ID3040913		-1,243	-1,158	-1,962	-1,608	-1,273	3,112				
ID1062		-2,204	-1,166	-4,768	-2,811	-1,749	-2,171				
ID52561		1,035	-1,175	1,196	1,206	-1,303	-1,627				
ID438739		-1,119	-1,179	1,129	-1,282	-1,045	-1,651				
ID3761685		1,395	-1,179	1,597	1,309	1,029	-1,295				
ID156308		-1,103	-1,196	-1,129	-2,618	-1,588	-1,437				
ID3019322		-2,007	-1,200	-1,049	-2,246	-1,315	-3,089				
ID3761675		-1,386	-1,202	-1,272	-1,751	-1,584	-1,475				
ID482337		-1,089	-1,202	-1,094	2,624	-1,114	-1,237				
ID348		1,022	-1,203	-1,270	-1,482	-1,291	-1,787				
ID527		-1,656	-1,208	-1,526	-1,478	-1,659	-2,116				
ID2447768		-1,673	-1,208	-1,221	1,355	1,021	-1,942				
ID1762		1,175	-1,212	-1,068	-1,635	-1,534	-1,554				
ID35204		-1,117	-1,214	1,061	-4,458	-1,259	-1,060				
ID463		-1,884	-1,216	-1,646	-1,093	1,315	-1,004				
ID2318312		-1,970	-1,218	1,178	-1,263	-1,605	-2,600				

Table S-2

ID[1]	Protein[2]	0 min[3]	30 min[3]	60 min[3]	24 h[3]	48 h[3]	72 h[3]	IPA symbol[4]	Entrez gene name[5]	location[6]	type(s)[7]
ID2016883		1,009	-1,222	1,127	-1,255	-1,148	-2,082				
ID1819877		-2,041	-1,222	1,446	1,573	-2,251	-2,662				
ID169541		1,134	-1,223	-1,134	-1,208	-1,865	-1,959				
ID8503		-1,161	-1,229	-1,535	-1,795	-1,560	-1,609				
ID364919		-1,646	-1,231	-1,518	-1,388	-1,767	-2,083				
ID1793947		-1,720	-1,231	-1,341	-2,004	-1,480	-1,889				
ID139104		-1,024	-1,232	-1,207	-1,178	-1,294	-2,045				
ID1164		-1,297	-1,234	-1,247	-1,002	-1,487	-1,555				
ID516		-1,299	-1,237	-1,134	-1,305	-1,635	-2,291				
ID2048120		-1,012	-1,240	1,150	2,463	-2,321	-2,026				
ID2437		1,369	-1,240	2,034	-2,204	-1,621	-1,692				
ID1964508		1,037	-1,241	-1,153	-1,350	-1,106	2,940				
ID121837		1,288	-1,242	1,538	-1,620	1,272	-1,666				
ID543455		-1,298	-1,243	-1,249	1,621	-1,259	-1,586				
ID421481		-1,829	-1,247	-2,013	1,003	-1,422	-1,569				
ID1717		-1,448	-1,249	-1,413	-4,821	-2,424	-1,771				
ID1298		-1,029	-1,251	-1,107	-11,917	-1,433	-1,322				
ID1933424		1,111	-1,253	1,082	-1,130	-1,330	2,708				
ID344430		-1,191	-1,255	1,060	1,518	-1,294	-1,061				
ID339		-1,073	-1,256	-1,370	-2,077	-1,687	-1,527				
ID3763685		-1,800	-1,256	-1,444	-1,402	-1,305	1,138				
ID1665		-1,358	-1,260	-1,840	1,035	-1,312	-1,132				
ID142473		-1,431	-1,263	-1,200	-2,346	-1,416	-2,106				
ID948		1,112	-1,264	-1,148	-1,785	-1,429	-1,366				
ID75950		-1,066	-1,267	-1,033	-2,086	-1,494	-1,542				
ID371		-1,132	-1,278	-1,271	-2,072	-1,295	-1,706				
ID470		-1,223	-1,283	-1,093	-1,169	-1,196	-1,640				
ID15367		-1,130	-1,287	-1,365	-4,818	-4,789	-7,873				
ID141431		1,272	-1,300	21,895	-1,954	-2,946	-2,389				
ID538		-1,074	-1,306	-1,109	-2,877	-1,698	-1,623				
ID1379		-1,384	-1,307	-1,314	-2,103	-1,981	-2,471				
ID1866181		-16,813	-1,311	-1,016	-2,638	-24,008	-13791,834				
ID3761658		-4,447	-1,311	-1,559	-2,573	-2,211	-1,723				
ID373664		-1,245	-1,312	-1,273	-2,142	-1,819	-1,597				
ID523		-1,349	-1,312	-1,416	-1,513	-1,571	-1,916				
ID137923		-1,165	-1,320	-1,490	1,016	-1,181	-1,621				
ID66151		1,138	-1,327	-1,476	1,643	1,377	2,807				
ID1219		-1,494	-1,331	-1,502	-1,499	-1,333	-1,182				
ID1299		2,257	-1,335	-5,526	-23,781	-3,945	-2,808				
ID639		-1,228	-1,336	-1,020	-1,405	-1,576	-1,217				
ID449534		-1,237	-1,341	-1,254	-1,455	-1,591	-1,408				
ID1652		-1,183	-1,351	-1,181	-9,002	-1,208	1,003				
ID1655		-1,425	-1,352	-1,121	1,966	-1,129	1,032				
ID1538		1,140	-1,354	1,121	-1,220	-1,362	-1,576				
ID1648		-1,305	-1,354	-1,053	-1,830	-1,526	-3,609				
ID2329037		-1,183	-1,356	-1,099	1,002	-1,362	-1,583				
ID294218		-1,323	-1,361	-1,133	-1,460	-1,663	-1,222				
ID1100		1,428	-1,362	-1,663	-3,618	-1,568	-1,712				
ID1398		-1,314	-1,380	-1,472	1,628	-1,007	-1,637				
ID3395583		-1,636	-1,382	-1,574	1,113	-1,664	-1,131				
ID315205		-1,145	-1,387	-1,274	-1,558	-1,634	-1,402				
ID2292906		-1,414	-1,392	-1,369	-1,426	-2,073	-1,497				
ID1785527		-1,781	-1,393	-1,515	-270,070	-1,533	-13,691				
ID1820		-1,265	-1,394	-1,177	-1,636	-1,041	1,873				
ID2021577		-1,289	-1,398	-1,168	-1,133	-1,514	-1,612				
ID221306		-1,228	-1,401	-1,651	1,131	-1,478	-1,324				
ID145564		-1,316	-1,402	-1,817	-1,468	-1,295	-1,296				
ID1750		-1,273	-1,403	-1,469	-1,117	-1,764	-1,736				
ID296837		-1,382	-1,405	-1,536	-1,030	-1,385	-1,340				

Table S-2

ID[1]	Protein[2]	0 min[3]	30 min[3]	60 min[3]	24 h[3]	48 h[3]	72 h[3]	IPA symbol[4]	Entrez gene name[5]	location[6]	type(s)[7]
ID1175		-1,299	-1,405	-1,448	-1,834	-1,324	-1,319				
ID16755		-1,454	-1,413	-1,142	1,008	-1,829	-2,302				
ID457100		-1,041	-1,416	-1,319	-1,537	-1,548	-1,602				
ID1009		-1,122	-1,418	-1,188	-1,349	-1,416	-1,617				
ID2522		-1,177	-1,418	-1,155	2,080	1,031	1,024				
ID855		-1,247	-1,420	-1,197	-7,167	-1,237	-1,068				
ID3184881		-1,425	-1,427	-1,370	1,051	2,060	-1,021				
ID1301		-1,272	-1,435	-1,341	-8,119	-24,618	-123,019				
ID2019749		-3,211	-1,440	-3,139	-1,112	-1,183	-1,285				
ID1228		1,235	-1,444	-1,514	1,126	1,149	1,683				
ID2439918		-1,174	-1,453	-1,541	1,353	-1,592	-1,221				
ID2039001		1,040	-1,458	-1,035	-1,580	1,013	-1,492				
ID1931678		-1,270	-1,459	-1,385	1,012	-1,260	2,038				
ID17878		-1,446	-1,461	-1,375	-2,087	-1,653	-1,158				
ID1506		-1,686	-1,463	-1,328	-1,541	-1,226	-1,029				
ID1864524		-1,314	-1,464	-2,571	1,564	-1,218	-2,479				
ID315424		-1,165	-1,468	-1,263	-1,664	-1,487	-1,321				
ID2087348		-1,212	-1,473	-1,378	-4,338	-3,015	-1,704				
ID1340		1,415	-1,479	-1,400	2,004	-1,445	1,092				
ID325		-1,018	-1,481	1,021	1,533	-1,092	-1,061				
ID1437		-1,356	-1,481	-1,206	-1,587	-1,412	-1,970				
ID594301		-1,050	-1,483	-1,103	1,789	1,004	1,094				
ID129210		-1,527	-1,486	-1,413	1,012	-1,243	-1,459				
ID1692		-1,342	-1,490	-1,250	-24,094	-1,357	-1,213				
ID1759		-1,234	-1,495	-1,490	-1,253	-1,199	-1,237				
ID1730		-1,271	-1,504	-1,063	-1,351	-1,341	-1,649				
ID1383		-1,397	-1,506	-1,251	-1,658	-1,369	-1,350				
ID2023391		-1,284	-1,512	-1,174	-1,469	-1,438	-1,664				
ID80360		-1,342	-1,514	-1,511	-2,180	-1,846	-1,620				
ID1792282		-1,809	-1,516	-1,593	-3,862	-2,824	-3,601				
ID411894		1,066	-1,521	-1,355	-1,690	-1,153	-1,273				
ID567		-1,590	-1,528	-1,209	-1,108	-1,180	-1,167				
ID1575		-1,353	-1,532	-1,411	1,066	1,047	-1,019				
ID31044		-1,000	-1,538	-1,166	-1,494	1,042	-1,146				
ID2302886		-1,461	-1,540	-1,343	-1,710	-10,130	1,857				
ID341		-1,322	-1,545	-1,497	-1,132	-1,486	-1,915				
ID2040831		-1,050	-1,545	-1,086	-2,239	-1,176	-1,798				
ID1303		-1,807	-1,556	-1,756	-1,592	-1,697	-1,214				
ID3711681		-1,299	-1,557	-1,481	-2,033	-1,772	1,023				
ID1457		-1,528	-1,561	-1,550	-1,467	-1,400	-1,432				
ID2042668		-1,156	-1,561	-1,098	-3,653	-1,595	-2,273				
ID149908		-1,364	-1,562	-1,227	-1,478	-1,268	-1,324				
ID1544		-1,372	-1,566	-1,203	-1,427	-1,484	-1,538				
ID186642		-1,143	-1,575	-1,144	1,131	-1,241	-1,832				
ID1537		-1,431	-1,580	3,206	-92,685	-1,711	-58,747				
ID210320		-1,388	-1,582	-1,621	-1,303	-1,498	-1,473				
ID216132		-1,828	-1,583	-1,884	-1,421	-1,028	-1,180				
ID1574		-1,143	-1,587	-6,755	-1,846	-1,184	1,739				
ID1736		-1,209	-1,592	-1,341	-1,343	-1,061	1,068				
ID1602		1,043	-1,593	-1,274	-1,939	1,065	-1,279				
ID1705		-1,288	-1,594	-1,295	-1,061	1,281	-1,108				
ID1593		-1,271	-1,594	-1,161	-1,140	-1,115	-1,669				
ID2974603		-1,334	-1,595	-1,302	-1,601	-1,152	-1,494				
ID1788919		-1,650	-1,597	-1,453	-6,677	-2,710	-3,912				
ID3068832		-1,221	-1,598	-1,471	-1,387	-1,178	-1,185				
ID440482		-1,266	-1,601	-1,395	-1,719	2,054	1,762				
ID113114		-2,085	-1,602	-1,432	-1,398	-1,568	-1,576				
ID566208		-1,248	-1,603	-1,674	-1,884	-1,403	-1,493				
ID1911469		-1,595	-1,607	-1,229	1,172	1,182	1,622				

Table S-2

ID[1]	Protein[2]	0 min[3]	30 min[3]	60 min[3]	24 h[3]	48 h[3]	72 h[3]	IPA symbol[4]	Entrez gene name[5]	location[6]	type(s)[7]
ID574393		-1,659	-1,610	-1,527	-1,844	-1,727	-1,938				
ID349		-1,077	-1,614	-1,501	-3,297	-1,758	-2,235				
ID65305		-1,495	-1,616	-1,333	-1,839	-1,270	-1,225				
ID1182		-1,815	-1,619	-2,307	-2,248	-2,505	-2,095				
ID154183		-1,795	-1,622	-1,567	-1,225	-1,136	-1,291				
ID3763682		-1,069	-1,623	-1,328	1,291	1,308	-1,274				
ID3597623		-1,726	-1,624	-1,552	-1,182	-1,408	-1,040				
ID119751		-1,681	-1,630	-1,676	-1,797	-1,669	-1,873				
ID2308652		-1,295	-1,642	-1,855	-4,251	-2,851	-2,568				
ID380		-1,425	-1,642	-1,594	-2,044	-1,554	-1,630				
ID1960958		-1,625	-1,643	-1,213	1,621	-1,079	-1,428				
ID2055531		-1,262	-1,648	-1,369	-1,024	-1,295	-1,576				
ID320497		-1,630	-1,648	-1,766	-1,807	-1,864	-2,842				
ID83942		-1,589	-1,653	-1,406	-1,632	-1,547	-1,487				
ID1709		-1,506	-1,656	-1,257	-1,532	-1,598	-1,590				
ID6678		-1,203	-1,659	-1,040	1,565	-1,361	-1,325				
ID1998321		1,063	-1,659	1,082	-3,219	-1,953	-3,281				
ID125371		1,295	-1,660	-1,216	-2,110	-1,808	-1,840				
ID1339		-1,562	-1,661	-1,589	-1,926	-1,541	-1,650				
ID1571		-1,408	-1,668	-1,303	-1,645	-1,482	-1,480				
ID33115		-1,112	-1,676	-1,166	-159,784	-1,678	-1,696				
ID23159		-1,195	-1,676	-1,540	-1,105	-1,556	-1,328				
ID2008587		-1,509	-1,677	-1,022	-1,593	-3,275	-1,916				
ID1151		-1,227	-1,677	-1,180	-1,073	-1,152	-1,420				
ID2788379		-1,440	-1,685	-1,530	-1,596	-1,632	-1,667				
ID1359		-1,578	-1,692	-1,786	-1,211	-1,257	-1,243				
ID1545		-1,451	-1,694	-1,245	-1,171	-1,482	-1,663				
ID1129		-1,510	-1,694	-1,395	-1,609	-1,332	-1,529				
ID2742902		-1,198	-1,695	-1,327	1,392	1,128	-1,056				
ID131133		-1,485	-1,699	-1,674	-1,502	-1,599	-1,803				
ID2723		-1,741	-1,701	-1,493	-1,274	-1,307	-1,160				
ID524		-2,125	-1,702	-2,399	-2,770	-2,562	-2,530				
ID3352574		-1,354	-1,703	-1,559	-1,650	-1,557	-1,471				
ID1190		1,191	-1,703	-1,617	-1,317	-1,033	1,105				
ID1081		-1,802	-1,705	-2,192	-2,127	-1,596	-1,823				
ID1394		-1,821	-1,705	-1,472	1,580	-1,072	-1,158				
ID1125		-1,459	-1,715	-2,096	-1,904	-1,493	-1,386				
ID350		-1,174	-1,715	-1,463	-2,481	-1,800	-1,767				
ID1283		-1,534	-1,728	-1,385	-1,293	-1,281	-1,335				
ID114983		-2,151	-1,728	-1,272	-2,490	-1,168	-1,727				
ID2051821		-1,545	-1,734	-1,515	1,025	-1,617	-1,635				
ID2057395		-1,501	-1,737	-1,557	-1,519	-1,750	-2,424				
ID1608		1,341	-1,737	-1,537	-4,644	-1,374	-1,575				
ID2060826		-1,424	-1,748	-1,492	-1,434	-1,664	-2,756				
ID529		-1,432	-1,750	-1,381	-2,385	-1,747	-1,584				
ID453		-1,776	-1,752	-2,422	-17,256	-1,721	-1,654				
ID2294		-1,239	-1,754	-1,132	-1,524	-1,266	-1,082				
ID83022		-1,572	-1,756	-1,609	-1,279	-1,432	-1,565				
ID3759858		-1,254	-1,759	-1,002	1,177	-6,433	-2,869				
ID1772		-1,448	-1,759	-1,470	-1,071	-1,149	-1,025				
ID3762560		-1,403	-1,759	-1,612	-1,400	-1,797	-1,266				
ID356		-1,422	-1,766	-1,568	-2,459	-2,026	-1,837				
ID1676		1,789	-1,767	-3,150	2,016	1,163	-1,089				
ID6662		-1,335	-1,769	-1,138	1,422	-1,591	-1,554				
ID91144		-1,552	-1,770	-1,470	-1,485	-1,284	-1,873				
ID1669		-1,719	-1,777	-1,551	-1,111	-1,339	-1,063				
ID464690		-1,445	-1,777	-1,447	-4,812	-1,744	-1,813				
ID398		-1,532	-1,781	-1,593	-1,708	-1,644	-1,724				
ID167310		-1,857	-1,786	-1,479	-1,646	-1,619	-1,358				

Table S-2

ID)1	Protein)2	0 min)3	30 min)3	60 min)3	24 h)3	48 h)3	72 h)3	IPA symbol)4	Entrez gene name)5	location)6	type(s))7
ID283779		-1,267	-1,787	-1,425	-1,430	-1,682	-1,929				
ID3130260		-1,313	-1,790	-1,472	-3,324	-2,353	-1,803				
ID463195		-1,337	-1,801	-1,450	-23,557	-2,414	-1,978				
ID1982157		-1,693	-1,804	-1,447	-3,520	-3,612	-3,863				
ID414		-1,337	-1,806	-1,744	-1,683	-1,807	-1,966				
ID57056		-1,286	-1,806	-1,213	-1,439	-1,291	-1,133				
ID2275539		-1,659	-1,806	-1,379	-2979,089	-1,406	-1,773				
ID1716		-1,028	-1,810	-1,242	-2,178	-1,465	-1,086				
ID938		-1,411	-1,810	-2,712	-4,837	-3,815	-1,693				
ID1568		-2,036	-1,818	-1,548	-1,443	-1,304	1,022				
ID483		-1,203	-1,824	-1,472	-2,379	-1,706	-1,585				
ID4923		1,043	-1,827	-1,257	-2,143	-1,696	-1,646				
ID136093		-1,405	-1,842	-1,806	-1,419	-1,412	-1,203				
ID1281		-1,073	-1,842	-2,218	-1,107	-1,149	-1,614				
ID2113599		-1,392	-1,850	-2,337	-2,310	-2,474	-2,492				
ID362785		-1,377	-1,854	-1,711	-1,639	-1,279	-1,333				
ID313877		-1,652	-1,856	-1,695	-3,315	-1,662	-1,261				
ID2140633		-1,729	-1,856	-1,363	-11,402	-1,904	-20,604				
ID1556		1,024	-1,859	-3,441	-2,791	-1,338	-1,949				
ID311244		-1,561	-1,861	-1,813	-2,049	-1,089	-1,623				
ID1136		-2,112	-1,866	-1,949	-1,266	-1,214	-1,221				
ID2290995		-2,082	-1,869	-1,734	-1,672	-14,586	-32,510				
ID2083630		-1,313	-1,871	-1,731	-5,493	-2,157	-2,944				
ID1787194		-1,543	-1,873	-1,454	-10,943	-3,574	-13,988				
ID461679		-1,328	-1,875	-1,759	-1,273	-1,903	-2,431				
ID1505		-1,915	-1,880	-1,571	-1,257	-1,436	-1,302				
ID2221628		-1,713	-1,884	-1,686	-4,283	-1,758	-2,131				
ID1233		-1,744	-1,887	-1,531	-1,468	-1,591	-1,294				
ID3763666		-1,813	-1,888	-1,933	-1,983	-1,859	-1,789				
ID476		-1,524	-1,894	-1,740	-1,639	-1,280	-1,187				
ID314		-1,745	-1,898	-1,693	1,026	-1,808	-1,490				
ID5909		1,050	-1,911	-1,794	-3,173	-1,952	-2,288				
ID1007		-1,505	-1,919	-2,791	-2,711	-2,196	-1,316				
ID82135		-1,548	-1,926	-1,484	-1,301	-1,290	1,128				
ID1017		-1,726	-1,928	-1,833	1,073	-1,252	-1,334				
ID519		-1,549	-1,929	-1,834	-2,371	-1,758	-2,072				
ID525		-1,773	-1,947	-1,864	-3,023	-1,775	-2,033				
ID3762259		-2,563	-1,955	-1,955	-1,311	-1,819	-1,503				
ID440		-1,951	-1,964	-2,677	-5,244	-1,980	-2,003				
ID16834		-1,995	-1,968	-1,701	-1,689	-1,468	-1,521				
ID158651		-2,003	-1,975	-2,520	-1,929	-1,946	-1,883				
ID328907		-1,284	-1,975	1,174	-2,374	-1,819	-2,143				
ID182136		1,313	-2,004	-2,647	-1,835	-2,021	-1,216				
ID386066		-1,134	-2,004	-1,601	-1,090	-1,353	-1,487				
ID2081733		-1,203	-2,006	-1,598	-7,546	-2,117	-3,471				
ID586		-2,459	-2,030	-1,887	-1,253	1,563	-6,155				
ID57837		-2,199	-2,034	-1,905	-2,256	-1,447	-1,127				
ID1582		-1,675	-2,040	-1,024	1,082	1,799	1,527				
ID130985		-1,623	-2,064	-2,001	-1,668	-1,572	-1,577				
ID257339		-1,668	-2,065	-1,641	-1,421	-1,753	-1,648				
ID1631		-1,489	-2,068	-1,150	-1,719	-1,468	-1,157				
ID5884		-2,643	-2,087	-2,032	-2,212	-1,304	1,041				
ID9000		-2,999	-2,096	-2,818	-2,335	-1,093	-1,145				
ID51068		-1,647	-2,107	-1,478	-1,480	-1,895	-1,740				
ID1617		-2,808	-2,107	-1,835	-6,842	-3,602	-4,069				
ID2005865		-1,764	-2,109	-1,196	-1,458	-1,901	-1,110				
ID1563		-2,900	-2,115	-1,376	-1,303	-1,058	-1,720				
ID242682		-1,229	-2,115	-1,795	-5,000	-2,100	-2,032				
ID23217		-1,562	-2,116	-1,785	-2,724	-1,824	-1,868				

Table S-2

ID)1	Protein)2	0 min)3	30 min)3	60 min)3	24 h)3	48 h)3	72 h)3	IPA symbol)4	Entrez gene name)5	location)6	type(s))7
ID2784333		-1,551	-2,124	-1,402	1,537	-1,438	-1,280				
ID315		-2,443	-2,128	-8,132	-3,338	-2,639	-18,503				
ID2044505		-1,587	-2,142	-1,520	-3,743	-2,084	-2,293				
ID240305		-1,392	-2,180	-1,879	-5,977	-2,392	-2,330				
ID3231778		-1,670	-2,180	-1,864	-1,853	-1,500	-1,416				
ID528		-1,583	-2,199	-1,988	-2,957	-2,604	-1,975				
ID165075		-1,232	-2,204	-1,457	-1,433	-1,546	-1,043				
ID1521		-3,804	-2,207	-2,283	-1,357	-1,317	-1,240				
ID576		-2,104	-2,210	-1,682	-1,571	-1,568	-1,249				
ID590		-1,464	-2,217	-1,307	-17,269	-2,144	-2,154				
ID306		-1,059	-2,221	-4,553	-3,528	-3,961	-1,568				
ID484		-1,684	-2,240	-1,919	-1,852	-1,470	-1,465				
ID2028269		-3,495	-2,245	-1,524	-4,987	-2,367	-2,797				
ID1535		-2,146	-2,247	-1,461	-2,906	-1,694	-1,845				
ID351		-1,504	-2,260	-1,834	-7,938	-2,935	-2,493				
ID452		-2,305	-2,261	-2,355	-21,748	-1,726	-1,649				
ID218682		-1,617	-2,267	-1,430	-2,065	-1,649	-1,892				
ID412768		-1,168	-2,276	-1,989	-2,126	-1,487	-1,954				
ID2190755		-2,820	-2,300	-2,146	-3,805	-7,151	-3,371				
ID2072345		-1,271	-2,302	-2,123	-28,864	-2,311	-4,307				
ID353686		-2,053	-2,337	-1,574	-2,678	-1,322	-1,898				
ID309		-1,396	-2,339	-1,698	-3,414	-2,319	-2,084				
ID4476		-2,090	-2,341	-1,932	-1,735	-1,675	-1,574				
ID2924537		-1,854	-2,347	-2,359	-1,235	-1,210	1,438				
ID393893		-1,484	-2,379	-2,312	-5,220	-3,557	-3,490				
ID495		-1,657	-2,413	-2,102	-1,688	-1,236	-1,266				
ID25129		-1,614	-2,420	-1,259	-1,962	-1,588	1,173				
ID4489		-2,051	-2,425	-2,001	-1,413	-1,508	-1,494				
ID1057		-2,593	-2,429	-2,199	-1,437	1,396	-2,146				
ID2830369		-1,756	-2,471	-1,766	-1,460	-1,416	-1,048				
ID2070460		-1,258	-2,514	-2,154	-875,916	-6,682	-12,294				
ID2379		-2,267	-2,517	-2,628	-5,882	1,240	2,704				
ID1533		-1,969	-2,520	-1,785	-1,800	-2,261	-1,846				
ID2300514		-1,876	-2,523	-1,515	4,173	4,363	6,119				
ID240147		-1,477	-2,538	-2,155	-6,402	-2,733	-3,003				
ID2064822		-2,085	-2,543	-2,266	-2,643	-3,123	-3,072				
ID3760248		-2,688	-2,561	-2,381	-1,948	-1,778	-1,521				
ID1594		-2,100	-2,565	-1,607	-2,530	-2,119	-2,020				
ID2068313		-1,344	-2,577	-1,831	-4,515	-2,532	-2,939				
ID5273		-1,614	-2,577	-2,018	-2,136	-1,236	-1,676				
ID522		-2,421	-2,589	-2,163	-52,771	-4,780	-10,178				
ID1540		1,888	-2,634	-3,626	-1,803	1,935	1,190				
ID1481		-1,555	-2,635	-1,271	-2,634	-1,027	-1,277				
ID2085530		-1,218	-2,659	-1,778	-4,285	-1,802	-3,379				
ID2838327		-2,428	-2,678	-2,445	-1,701	-1,541	-1,089				
ID2944561		-2,463	-2,691	-1,515	-1,229	1,162	1,176				
ID2555205		-1,470	-2,734	-2,628	-2,342	-3,840	-4,102				
ID1124		-1,480	-2,738	-1,782	-1,619	-1,222	-1,127				
ID1045		-3,818	-2,743	-2,272	-1,124	1,005	-4,829				
ID110384		-1,996	-2,797	-2,183	-2,626	-1,591	-1,646				
ID2288948		2,197	-2,812	-1,446	n.d.	-2,104	-100,425				
ID2024619		-1,595	-2,831	-1,170	-6,251	-85,030	-5,571				
ID114043		-2,598	-2,846	-1,842	-3,003	-1,555	-1,848				
ID2062951		-1,897	-2,886	-1,993	-7,545	-4,233	-5,816				
ID388		-1,509	-2,891	-3,483	-8,458	-4,612	-4,615				
ID109442		-1,424	-2,893	-1,346	-1,426	-1,259	-1,556				
ID2204207		-1,862	-2,940	-2,460	-5,565	2,511	-1,725				
ID429069		-3,646	-2,940	-5,175	1,091	-1,103	-1,224				
ID1700		-2,487	-2,952	-1,378	2,904	1,661	1,804				

Table S-2

ID)1	Protein)2	0 min)3	30 min)3	60 min)3	24 h)3	48 h)3	72 h)3	IPA symbol)4	Entrez gene name)5	location)6	type(s))7
ID1621		-2,492	-2,973	-1,898	-1,635	-2,349	-1,473				
ID2549343		-1,605	-2,978	-2,533	-2,208	-1,619	-1,576				
ID1048		-1,604	-3,013	1,699	-6,054	1,670	-1,968				
ID1101		-2,801	-3,074	-2,582	-2,387	-1,848	-1,665				
ID2926520		-1,537	-3,199	-4,382	-1,224	1,232	2,963				
ID233300		-1,061	-3,246	-1,293	-1,180	-1,244	-1,094				
ID305		-1,528	-3,259	-7186,377	-3,622	-6,298	-1,438				
ID37898		-3,128	-3,283	-3,531	-7,742	-2,159	-2,079				
ID1763758		-2,021	-3,319	-2,551	-5,920	-19,024	-6,999				
ID613		-5,362	-3,321	-2,150	-5,608	-9,066	-3,292				
ID2194613		-1,698	-3,347	-1,993	-6,050	-7,186	-4,964				
ID1025		-2,057	-3,382	-2,043	-1,505	-1,544	1,017				
ID168476		-1,641	-3,387	-2,305	-2,132	-2,345	-1,385				
ID642		-2,524	-3,393	-2,119	-2,147	-2,792	-3,488				
ID985		-1,785	-3,480	-2,640	-2,738	-2,043	-1,639				
ID2030105		-3,911	-3,500	-2,427	-2,333	-3,949	-1,976				
ID262545		-2,159	-3,507	-2,599	-1,637	-2,125	-1,529				
ID2105992		-1,854	-3,621	-2,493	-6,934	-4,514	-4,272				
ID1550		-1,560	-3,688	-1,648	-2,265	-1,262	-1,456				
ID389942		-1,525	-3,742	-1,529	-1,267	-1,277	-1,179				
ID3763337		-1,458	-3,819	-1,969	-8,015	-26,638	-13,636				
ID352		-1,981	-3,835	-2,541	-19,065	-3,178	-2,723				
ID1000		-2,604	-3,877	-1,855	-2,483	-2,393	-1,226				
ID2031934		-3,586	-3,905	-2,457	-1,803	-2,861	-2,652				
ID89291		1,069	-3,990	-1,360	-2,132	-1,547	-2,397				
ID1518		-2,136	-4,028	-3,754	-1,834	1,012	1,519				
ID1006		-2,233	-4,073	-14,746	-4,986	-2,550	-1,514				
ID1557		-1,127	-4,138	-1,514	-2,268	-3,799	-1,920				
ID36784		-2,460	-4,211	-6,942	-85,657	-2,168	-1,931				
ID1158		-2,795	-4,238	-3,339	-1,732	-1,349	-1,064				
ID1130		-3,021	-4,277	-16,098	-5,981	-3,627	1,282				
ID1295		-1,425	-4,296	-1,034	-2,492	-9,752	-13,720				
ID1105		-3,327	-4,303	-2,434	-2,391	-1,630	-1,616				
ID1534		-1,868	-4,375	-3,089	-1,883	-2,688	-2,081				
ID2737054		-1,575	-4,590	-3,593	-36,586	-1,448	-7,756				
ID245554		-2,849	-4,775	-1,988	-3,872	1,789	1,867				
ID1138		-11,286	-4,821	-10,997	-2,783	-1,860	-1,212				
ID1266		1,024	-5,049	-1,230	-1,436	-1,014	1,079				
ID261244		-1,864	-5,306	-2,407	-1,633	-4,152	-2,015				
ID24906		-4,031	-5,312	-20,269	-27,075	-1,806	-23,951				
ID1366		-2,311	-5,496	-2,778	-3,101	-1,424	-1,047				
ID25932		-3,521	-5,908	-3,556	-7,077	-3,765	-2,814				
ID1223		-5,219	-6,413	-7,877	-1,116	-1,495	1,113				
ID2917416		-4,480	-6,602	-4,329	-3,047	-1,115	1,079				
ID1797353		-4,341	-7,015	-4,394	-3,321	-3,699	-2,975				
ID300154		-4,114	-7,377	-3,251	-4,223	-3,968	-2,176				
ID2110071		-5,866	-7,704	-5,202	-8,260	-6,699	-5,815				
ID2138752		2,173	-8,361	1,583	4,390	1,579	1,005				
ID51803		-2,721	-9,166	-2,865	-23,808	-3,387	-1,906				
ID1657		-13,654	-9,739	-2,466	-3,349	1,133	-5,519				
ID1327		-1,092	-11,077	3468,355	-144,490	n.d.	n.d.				
ID178566		-11,630	-11,965	-6,128	-5,041	-2,089	1,001				
ID1785181		-2,693	-14,067	-1,935	-6,360	-4,789	-26,988				
ID1763803		-2,890	-14,777	-2,269	-79,397	-14,935	-18,357				
ID1543		-6,094	-16,824	-1,789	-24,539	-11,049	-29,354				
ID2106278		-2,500	-20,869	-2,380	-12,788	-11,369	-7,042				
ID1615		-3,328	-21,266	-1,913	-23,958	-1374,679	-14,475				
ID232052		-1,344	-21,467	-3,242	1,347	-1,140	-1,069				
ID2314456		-5,179	-22,092	-17,031	3,445	-1,034	1,839				

Table S-2

ID[1]	Protein[2]	0 min[3]	30 min[3]	60 min[3]	24 h[3]	48 h[3]	72 h[3]	IPA symbol[4]	Entrez gene name[5]	location[6]	type(s)[7]
ID146603		-1,939	-34,706	-1,444	1,120	-1,263	1,055				
ID183880		-69,551	-35,239	-64,293	-2,270	-1,790	-1,582				
ID1780185		-1,819	-47,689	-2,076	-18,279	-6,578	-7,423				
ID2977290		-46,268	-102,311	-3,238	-3,243	-2,094	-3,332				
ID3464756		-3,019	-228,727	-3,907	-3,112	-2,112	1,332				
ID1302		1,488	-253,707	1,356	-5,395	-793,377	1,047				
ID1677		-1,361	-634,735	-1,876	4,663	4,184	3,012				

Table S-3: Summary results from network-analysis using Ingenuity Pathway Analysis (IPA) for each time point.
Spot-lists of MALDI-TOF-MS identified proteins from statistical-analyses of each time point were analysed.
Resulting networks, molecular and cellular functions, as well as “Top Tox Lists” and the respective scores are displayed.
The same categories of “Top Tox Lists” or “molecular and cellular functions” were coloured equally.

Table S-3

	rank	Associated Network Functions	score
**0 min**	1	Post-translational modification, protein folding, cancer	46
	2	Cell morphology, cancer, reproductive system disease	43
	3	Cancer, reproductive system disease, cellular assembly and organization	43
	4	Post-translational modification, protein degradation, cell death	42
	5	Carbohydrate metabolism, small molecule biochemistry, immunological disease	41
**30 min**	1	Cancer, reproductive system disease, cardiovascular disease	47
	2	Protein synthesis, cancer, RNA post-translational modification	41
	3	DNA replication, recombination and repair, energy production, nucleic acid metabolism	40
	4	Cell morphology, genetic disorder, neurological disease	39
	5	Post-translational modification, protein folding, amino acid metabolism	39
**60 min**	1	Infection mechanism, organismal development, lipid metabolism	52
	2	Cancer, reproductive system disease, cardiovascular disease	49
	3	Post-translational modification, protein folding, cellular function and maintenance	47
	4	Neurological disease, cancer, genetic disorder	46
	5	Post-translational modification, protein folding, genetic disorder	26
**24 h**	1	Cancer, reproductive system disease, cardiovascular disease	42
	2	Gene expression, post-translational modification, cellular assembly and organization	40
	3	Amino acid metabolism, post-translational modification, small molecule biochemistry	35
	4	Cancer, cellular assembly and organization, small molecule biochemistry	33
	5	Tumor morphology, cell death, DNA replication, recombination and repair	33
**48 h**	1	Post-translational modification, protein folding, infectious disease	52
	2	Cellular assembly and organization, cancer, reproductive system disease	46
	3	Cell death, neurological disease, organismal injury and abnormalities	33
	4	DNA replication, recombination and repair, cardiovascular disease, infection mechanism	31
	5	Protein synthesis, skeletal and muscular system development and function, tissue morphology	28

Table S-3

	rank	Associated Network Functions	score
**72 h**	1	Cellular assembly and organization, cancer, reproductive system disease	51
	2	DNA replication, recombination and repair, post-translational modification, protein folding	49
	3	Infection mechanism, infectious disease, amino acid metabolism	25
	4	Cell-to-cell signalling and interaction, cellular assembly and organization, cellular function and maintenance	22
	5	Cell death, infection mechanism, cell cycle	19

Table S-3

	rank	Top Tox lists	p-value / ratio
0 min	1	Oxidative stress	1.20E-05 / 7/57 (0.123)
	2	Nrf2 mediated oxidative stress response	5.28E-04 / 10/205 (0.049)
	3	Mitochondrial dysfunction	7.42E-03 / 6/125 (0.048)
	4	Mechanism of gene regulation by peroxisome proliferators via PPAR alpha	4.13E-02 / 4/95 (0.042)
	5	G2/M transition of the cell cycle	8.24E-02 / 2/35 (0.057)
30 min	1	Nrf2 mediated oxidative stress response	1.95E-03 / 10/205 (0.049)
	2	Oxidative stress	2.28E-03 / 5/57 (0.088)
	3	Mitochondrial dysfunction	4.31E-03 / 7/125 (0.056)
	4	G2/M transition of the cell cycle	1.89E-02 / 3/35 (0.086)
	5	Cholesterol biosynthesis	2.71E-02 / 2/16 (0.125)
60 min	1	Oxidative stress	7.95E-04 / 5/57 (0.088)
	2	Nrf2 mediated oxidative stress response	4.93E-03 / 8/205 (0.039)
	3	Mitochondrial dysfunction	2.22E-02 / 5/125 (0.04)
	4	Mechanism of gene regulation by peroxisome proliferators via PPAR alpha	1.22E-01 / 3/95 (0.032)
	5	Aryl hydrocarbon receptor signalling	1.29E-01 / 4/151 (0.026)
24 h	1	Nrf2 mediated oxidative stress response	8.63E-05 / 12/205 (0.059)
	2	Aryl hydrocarbon receptor signalling	5.79E-04 / 9/151 (0.06)
	3	Oxidative stress	1.86E-03 / 5/57 (0.088)
	4	Mechanism of gene regulation by peroxisome proliferators via PPAR alpha	1.60E-02 / 5/95 (0.053)
	5	PPAR alpha/RXR alpha activation	4.46E-02 / 6/166 (0.036)
48 h	1	Nrf2 mediated oxidative stress response	6.69E-03 / 6/205 (0.029)
	2	Mechanism of gene regulation by peroxisome proliferators via PPAR alpha	7.57E-03 / 4/95 (0.042)
	3	Oxidative stress	1.12E-02 / 3/57 (0.053)
	4	PPAR alpha/RXR alpha activation	4.59E-02 / 4/166 (0.024)
	5	PXR/RXR activation	1.06E-01 / 2/68 (0.029)

Table S-3

	rank	Top Tox lists		p-value / ratio
**72 h**	1		Mechanism of gene regulation by peroxisome proliferators via PPAR alpha	3.94E-03 / 4/95 (0.042)
	2		Oxidative stress	6.77E-03 / 3/57 (0.053)
	3		Nrf2 mediated oxidative stress response	1.28E-02 / 5/205 (0.024)
	4		PPAR alpha/RXR alpha activation	2.63E-02 / 4/166 (0.024)
	5		PXR/RXR activation	7.76E-02 / 2/68 (0.029)

Table S-3

	rank	Molecular and cellular functions	p-value / molecules
**0 min**	1	cell death	3.26E-11 - 2.71E-02 / 101
	2	post-translational modification	3.26E-11 - 2.71E-02 / 55
	3	protein folding	4.65E-11 - 2.71E-02 / 43
	4	cellular growth and proliferation	2.32E-08 - 1.59E-02 / 40
	5	cellular function and maintainance	2.32E-08 - 1.37E-02 / 15
**30 min**	1	post-translational modification	8.29E-14 - 2.71E-02 / 42
	2	protein folding	8.29E-14 - 1.62E-02 / 16
	3	cell death	4.05E-13 - 2.73E-02 / 110
	4	cellular growth and proliferation	1.18E-07 - 1.46E-02 / 67
	5	cellular function and maintainance	2.71E-07 - 2.70E-02 / 35
**60 min**	1	post-translational modification	1.95E-15 - 2.54E-02 / 37
	2	protein folding	1.95E-15 - 1.28E-02 / 16
	3	cell death	7.86E-10 - 2.63E-02 / 86
	4	cellular function and maintainance	2.89E-08 - 2.54E-02 / 31
	5	cellular growth and proliferation	1.77E-07 - 2.54E-02 / 52
**24 h**	1	post-translational modification	1.11E-11 - 2.48E-02 / 40
	2	protein folding	1.11E-11 - 2.88E-04 / 14
	3	cell death	1.14E-10 - 2.86E-02 / 96
	4	cellular growth and proliferation	1.43E-10 -2.25E-02 / 67
	5	cellular function and maintainance	1.50E-08 - 2.48E-02 / 31
**48 h**	1	cell death	1.90E-06 - 4.01E-02 / 49
	2	cellular growth and proliferation	2.41E-06 - 3.22E-02 / 35
	3	cellular assembly and organization	2.44E-06 - 4.26E-02 / 35
	4	DNA replication, recombination and repair	7.10E-06 - 3.47E-02 / 21
	5	cellular movement	3.79E-05 - 4.01E-02 / 23

Table S-3

**72 h**	1		cellular growth and proliferation	6.58E-07 -4.91E-02 / 32
	2		cell death	7.20E-07 - 4.91E-02 / 43
	3		cellular assembly and organization	3.40E-06 - 4.64E-02 / 33
	4		DNA replication, recombination and repair	3.40E-06 - 3.87E-02 / 20
	5		RNA post-translational modification	1.63E-04 - 6.77E-03 / 6

Table S-3

Table S-4: Detailed information on proteins/genes of all ten K-means clusters

)1 hit ID obtained from Delta2D analysis (Delta2D statistically software version 4.4, Decodon (Germany, Greifswald))

)2 protein identification corresponding to human proteins (UniProt-SwissProt database; Rel. 51.5 restricted to human taxonomy)

)3 mean values of Z-scores of four replicates of each (control, plasma-treated samples, 0, 0.5, 1, 24, 48, 72 h)

Table S-4: cluster 1

Cluster 1 ID[1]	Protein[2]		Z-score[3] co_0 min	co_30 min	co_60 min	co_24 h	co_48 h	co_72 h	PI_0 min	PI_30 min	PI_60 min	PI_24 h	PI_48 h	PI_72 h
ID1212			-0,424318	0,394877	-1,251199	-0,215585	-1,125894	-0,636255	0,700078	2,086237	0,286998	0,057519	0,063910	0,078012
ID337908			-0,314066	-0,503743	-0,171196	-0,306789	-1,639089	-1,056021	0,534570	2,005223	0,786007	1,011595	0,083715	-0,177309
ID17707			-0,481389	-0,931144	-0,840362	-1,098343	-1,370938	0,008203	0,308428	2,003950	0,610216	1,213270	0,730378	0,151048
ID29990	TCPA		-0,237644	-0,426812	0,044905	-0,464847	-1,211697	-0,860936	1,108427	1,989185	1,520625	-0,432785	-0,432816	-0,703801
ID801	2AAA		0,328879	-1,082253	0,690083	-1,170179	-0,612393	-0,769382	-0,023836	1,983072	0,898672	0,605264	0,233176	-0,929787
ID1802534			-0,192365	-0,450083	-0,223509	-0,202945	-0,458889	-0,804559	0,522840	1,884237	0,465373	0,672573	0,391302	-1,435831
ID1799192			-0,058520	-0,439814	-0,213487	-0,675517	-0,961821	-0,971986	0,860352	1,838554	0,975089	0,980608	0,312842	-1,401149
ID1512	CATB		0,045507	-0,732217	-0,175643	-0,930707	-1,138316	-0,998309	1,206237	1,812000	1,500098	0,482647	-0,216759	-0,733876
ID97395			0,449251	-0,279115	0,707949	-0,998109	-0,775392	-1,481416	0,375060	1,767685	0,390921	0,316068	0,340036	-0,733921
ID825	GDIA	ANXA3, NUCB1	0,056478	-0,720333	0,115940	-1,264369	-1,182949	-1,373503	0,731834	1,748653	1,235829	0,903903	0,348437	-0,373945
ID848			0,220537	-0,421408	0,245731	-0,978766	-1,096063	-0,626077	0,898598	1,744874	1,617333	-0,418083	-0,871188	-0,420010
ID742			-0,030712	-0,836273	-0,076461	-1,127388	-0,085182	-1,451461	0,734442	1,736951	1,112893	0,053485	0,163661	-0,180583
ID3227	NUCB1	VIME	-0,295595	-0,851695	-0,181834	-1,139240	-1,127620	-1,297596	0,918562	1,731381	1,226041	0,974006	0,429395	-0,142304
ID836	PDIA3	TBA1B	0,053032	-0,563765	0,078625	-1,289766	-1,251459	-1,035296	1,335647	1,712505	1,238349	0,254068	0,023629	-0,492053
ID238040			0,006071	-0,800709	-0,301228	-1,088713	-1,296255	-1,161291	0,878543	1,706118	1,123447	0,801168	0,584649	-0,251508
ID813	P4HA	TCPA, PDIA3, CPNE2	-0,349540	-0,375865	-0,038907	-0,394431	-0,975094	-1,075585	0,966801	1,701000	1,421626	0,782203	0,028985	-1,495642
ID3406	IMA7		-0,333082	-1,349156	-0,307276	-0,605835	-0,548118	-0,645820	0,297442	1,685392	0,847637	1,304129	0,315071	-0,334353
ID7425			-0,281229	-0,899414	-0,295001	-1,037609	-1,572761	-0,596771	1,273792	1,668055	1,205721	0,687386	0,341761	-0,322083
ID124300			-0,219948	-0,746152	-0,198008	-1,393107	-1,308406	-1,182152	0,612451	1,658265	0,779508	1,186258	0,867612	0,240244
ID266132			0,113911	-0,488887	0,145561	-0,993069	-0,870598	-1,796667	1,133758	1,649396	1,268776	-0,130706	0,295331	-0,359482
ID7589			-0,226846	-0,780329	-0,240707	-1,461114	-1,382639	-0,357816	1,335007	1,616257	1,112189	0,770709	0,194850	-0,386884
ID1502	CRK		0,281826	-0,434042	0,137063	-0,651140	-1,177842	-1,552867	1,186228	1,605012	1,361212	0,622109	-0,377421	-0,844610
ID407			-0,178421	-0,803468	-0,216164	-1,298029	-1,463873	-0,810840	1,223820	1,550390	1,264759	0,716359	0,435138	-0,240582
ID828	GDIA	VTDB, 2AAA	-0,028874	-0,755763	-0,032864	-1,302144	-1,255093	-1,465694	0,912497	1,522687	1,210978	0,861267	0,531189	0,017132
ID1566			0,280451	-0,593643	-0,335127	-0,995199	-1,122451	-0,300839	0,894555	1,520226	1,021427	-0,662210	0,090882	0,036376
ID394			-0,130831	-0,830917	-0,113584	-1,529694	-1,363759	-0,675960	0,905595	1,515186	1,414518	0,882182	0,452450	-0,304642
ID423			0,173682	-0,630169	0,043597	-1,364992	-1,375854	-1,034946	0,813657	1,497389	1,280069	0,905380	0,495880	-0,577347
ID749			-0,029211	-0,774123	-0,012912	-1,051619	-1,232256	-1,588268	1,287859	1,490305	1,228518	0,463473	0,384291	-0,050190
ID392			-0,000845	-0,774155	-0,074563	-1,334440	-1,395788	-0,777703	0,879853	1,487248	1,427588	0,936565	0,430906	-0,570523
ID775			0,042198	-0,674133	-0,086291	-1,491527	-1,378586	-1,295223	0,973104	1,482303	0,760981	0,987662	0,723269	0,203160
ID1114	ACTB		0,027433	-0,425219	0,090603	-0,945817	-1,434845	-1,685103	0,996903	1,476822	1,092980	1,035221	0,178817	-0,148989
ID898			-0,180469	-0,369897	-0,420876	-1,413352	-1,313611	-1,134179	0,717523	1,471557	1,038558	1,414985	0,679022	-0,135514
ID1044	VIME		-0,431599	0,610668	-0,358816	-0,547889	-0,708307	-1,803131	1,174836	1,464905	1,564794	-0,120613	-0,061217	-0,813783
ID750			0,015082	-0,632430	0,162918	-0,915139	-1,208776	-1,935985	0,966564	1,462309	1,089170	0,473780	0,533275	0,107678
ID50273	NSAP	CALR	-0,984051	-1,102674	-1,101256	-0,528087	-0,493707	-0,394492	1,582276	1,462069	1,536800	0,072821	0,305161	-0,336654
ID730			0,112514	-0,462054	0,123357	-0,869185	-0,337408	-2,167037	0,725495	1,442681	0,858009	0,110650	0,469749	0,020890
ID3179			-0,059798	-0,841766	-0,112797	-1,314117	-1,298422	-1,438491	0,857837	1,442568	1,134209	0,857163	0,690765	0,297140
ID713			0,303461	-0,307565	0,229983	-1,324550	-1,488571	-1,581637	1,058741	1,437630	1,130516	0,472347	0,343262	-0,155531
ID773			0,070914	-0,639520	0,091555	-1,335449	-1,320242	-1,548457	1,084207	1,427171	1,033086	0,699289	0,653572	-0,041303
ID802			0,019430	-0,547542	-0,331658	-1,479809	-1,414287	-0,883093	1,132255	1,426670	1,345289	0,876236	0,300521	-0,224953
ID370380	PDIA4		-0,903423	-0,955459	-0,448436	-0,404508	-0,839655	-1,549562	0,933327	1,426016	1,211079	0,303612	0,721531	0,581380
ID858			0,011491	-0,560169	-0,165595	-1,242965	-1,393547	-1,491416	1,078184	1,407996	1,180517	0,844307	0,575854	-0,033580
ID29087			-0,091882	-0,703374	-0,016956	-1,046145	-1,560352	-1,406536	0,861322	1,400934	1,081902	1,161323	0,661749	0,238678
ID719			0,152083	-0,594269	0,190451	-1,229114	-1,122979	-1,697505	1,145868	1,400105	1,287755	0,451834	0,352273	-0,223544
ID2183			0,064492	-0,537649	-0,111600	-1,184458	-1,461016	-1,521066	1,143368	1,399003	1,213705	0,639115	0,522723	-0,006839
ID853	STABP		0,108637	-0,515177	-0,033019	-1,232607	-1,461830	-1,527553	1,107612	1,398929	1,210857	0,655232	0,485883	-0,033156
ID1559			-1,003147	-0,812659	-1,012889	-0,659428	-0,654471	-0,568134	1,522434	1,397402	1,723026	0,583328	-0,049117	-0,320513
ID782			0,123914	-0,557183	0,073696	-1,230177	-1,465371	-1,581325	1,124921	1,391985	1,039323	0,714530	0,562156	-0,017835
ID751	TRXR1		0,061769	-0,653710	0,064303	-1,258639	-1,409264	-1,556086	1,095376	1,389117	1,092846	0,683730	0,607537	0,053954
ID352255			-0,281105	-0,860215	-0,338992	-0,744217	-1,431880	-1,454841	1,041382	1,388892	1,134991	0,567685	0,724256	0,395967
ID42780			0,092786	-0,546944	0,148293	-1,484763	-1,081628	-1,559331	0,943590	1,385020	1,133029	0,581000	0,539427	-0,005229
ID688	TRFL		0,013914	-0,690200	-0,117602	-1,238142	-1,391221	-1,351294	1,139124	1,384077	1,356496	-0,004811	0,709391	0,189066
ID41789			-0,064645	-0,987928	-0,029485	-1,671607	-1,354419	-0,172294	1,088178	1,382414	1,387693	0,293327	0,436224	-0,234127
ID45562			-0,137145	-0,706259	-0,217832	-1,111327	-1,447368	-0,732091	1,787961	1,377955	1,197916	0,066761	0,241520	-0,303401
ID3483954			0,007966	-0,632750	-0,426460	-1,219266	-1,209416	-1,471652	1,153961	1,371646	1,217959	0,869336	0,605865	-0,049855
ID754			0,101660	-0,612958	0,118678	-1,196333	-1,380543	-1,514765	1,134777	1,371110	1,248776	0,605316	0,430736	-0,155124
ID43156			0,050024	-0,547026	0,017744	-1,515339	-1,193995	-1,466306	0,966216	1,368458	1,135044	0,880304	0,467573	0,057381

Table S-4: cluster 1

ID)1	Protein)2		co_0 min	co_30 min	co_60 min	co_24 h	co_48 h	co_72 h	PI_0 min	PI_30 min	PI_60 min	PI_24 h	PI_48 h	PI_72 h
ID756			0,130377	-0,636934	0,103914	-1,254082	-1,402937	-1,499638	1,179558	1,368434	1,225646	0,582376	0,429995	-0,081115
ID1828633	ALDOA		0,390420	-0,501952	0,170260	-1,340836	-1,397950	-1,548516	1,047548	1,362878	1,143230	0,506946	0,477701	-0,182991
ID9044			0,136095	-0,634039	0,033701	-1,451435	-1,512567	-0,911809	0,810623	1,361677	1,129367	1,092847	0,740299	-0,521548
ID3128108	RD23B		-0,056971	-0,773959	-0,142404	-1,222867	-1,354494	-1,233270	1,047704	1,356772	1,121489	0,642093	0,574009	0,202419
ID714	RANB3		0,204615	-0,542766	0,212171	-1,377503	-1,275629	-1,570966	1,066309	1,353081	1,268085	0,484918	0,421529	-0,122615
ID2404447	NP1L4		0,136442	-0,553728	-0,092383	-1,202197	-1,362957	-1,650466	1,063476	1,339245	1,213946	0,583148	0,651601	0,019661
ID774	TM1L2		0,023552	-0,750875	-0,000331	-1,417676	-1,337118	-1,418855	1,037403	1,337101	1,014070	0,907221	0,730915	0,101397
ID437			0,125521	-0,625877	0,125573	-1,517374	-1,563679	-0,594901	0,412378	1,330949	0,910308	1,236332	1,076353	-0,606500
ID1478	ACTG	NNMT, CLIC4	-0,619418	-0,134469	0,001347	-1,363818	-0,814937	-0,161787	0,187644	1,323948	0,622834	2,557703	-0,351682	-0,607937
ID2200	TBA3		0,132219	-0,468359	-0,217526	-1,235387	-1,468645	-1,505381	1,191537	1,317767	1,191010	0,686559	0,546647	0,001200
ID1015	ENOA		0,083409	-0,600092	-0,148791	-1,298000	-1,430219	-1,428476	1,253771	1,315444	1,178128	0,690339	0,514604	0,042470
ID2729506			0,033732	-0,663437	0,043766	-1,491890	-1,477741	-1,310912	0,952189	1,302867	0,955074	0,943466	0,763355	0,185398
ID764	UBQL1		-0,028691	-0,744396	-0,078405	-1,330230	-1,434380	-1,456792	0,907688	1,296266	1,036353	0,898891	0,807483	0,350935
ID40401			0,194568	-0,534625	0,202545	-1,389167	-1,333228	-1,610097	1,021943	1,295456	1,215892	0,661195	0,473480	-0,032664
ID237648			0,174473	-0,741317	0,017635	-1,235239	-1,888328	-0,610874	1,115315	1,294307	1,081792	0,900923	0,347173	-0,230630
ID41097			0,112031	-0,602873	0,108911	-1,546538	-1,449399	-1,179440	1,036858	1,291514	1,213573	0,852887	0,438494	-0,062797
ID767	2AAB	UBQL1, IRGQ	-0,131145	-0,815449	-0,170898	-1,397340	-1,370477	-1,348800	0,840525	1,285299	0,988381	1,011538	0,900915	0,460335
ID1230			-1,342959	-1,088603	-0,711950	0,343723	-1,327994	-0,348821	0,926720	1,284402	0,902745	0,603706	0,264487	0,645471
ID851	RHG01		0,148911	-0,515702	-0,261013	-1,317473	-1,457905	-1,379064	1,223456	1,282259	1,234579	0,738585	0,529011	-0,041000
ID770	HNRPK		0,165325	-0,541379	0,134679	-1,321117	-1,507991	-1,566082	1,018819	1,281315	1,082647	0,766967	0,605257	0,073303
ID1178			0,104181	-0,128195	0,516689	-0,558345	-0,855091	-1,198346	-0,083278	1,281241	0,584900	-0,436689	1,217010	-0,553250
ID108562			0,255526	-0,472497	0,121243	-1,178667	-0,422986	-1,968066	0,920881	1,279872	1,073438	0,129601	0,445417	-0,151363
ID1335	TSNAX		0,119638	-0,106528	0,299211	-1,254097	-1,124018	-1,012865	1,020493	1,277989	1,497196	-1,746881	0,604486	-0,448065
ID776			-0,095253	-0,832138	-0,141700	-1,399576	-1,361404	-1,378568	0,815086	1,265732	0,944440	1,001078	0,929598	0,502975
ID3762296			-0,074753	-0,552880	-0,258081	-1,290046	-1,507248	-1,328186	0,937990	1,265313	1,222678	0,860101	0,799332	0,140805
ID132415			0,159226	-0,476421	0,116088	-1,244317	-1,453703	-1,540055	1,091521	1,264733	1,198444	0,559075	0,510725	-0,045547
ID842	NP1L4		0,149924	-0,525545	-0,201091	-1,265567	-1,465506	-1,512560	1,151374	1,257887	1,205296	0,699834	0,608209	0,072703
ID1466			-1,051077	-0,895959	-0,928217	-0,785497	-0,813528	-0,835110	1,128323	1,254063	1,787144	0,751506	0,506438	0,069791
ID267419			-0,081801	-0,176838	0,578220	-0,404945	0,250941	-1,959388	0,662873	1,248324	0,571516	-0,447983	0,188587	-0,541500
ID846	NP1L4		0,200181	-0,499884	-0,179243	-1,292425	-1,467001	-1,505826	1,139027	1,247381	1,212025	0,723900	0,582378	0,020463
ID845	NP1L1		0,108626	-0,469441	-0,276392	-1,430346	-1,420433	-1,337156	1,108867	1,247198	1,216239	0,844882	0,666683	-0,047506
ID759			0,213883	-0,441607	-0,126638	-1,361307	-1,525676	-1,469731	1,083331	1,233165	1,173337	0,794225	0,571904	0,053673
ID3316	MCM6	2AAA	0,111486	-0,629362	0,057593	-1,404578	-1,450415	-1,471549	1,042238	1,232328	1,138396	0,810157	0,632122	0,134124
ID785			0,194611	-0,427754	-0,101862	-1,332644	-1,563433	-1,434739	1,194963	1,230564	1,185953	0,704452	0,502698	0,023303
ID1475			0,755422	-0,045508	0,402102	-1,093211	-0,593180	-1,462109	1,374737	1,230542	1,103550	-0,553161	-0,437913	-0,819562
ID755	DPYL2		0,202491	-0,432225	-0,127419	-1,326996	-1,553462	-1,468729	1,166901	1,226119	1,169166	0,704141	0,554570	0,061477
ID413			0,169058	-0,627230	0,097099	-1,272226	-1,470104	-1,343889	1,159137	1,216819	1,142593	0,782129	0,671191	-0,329046
ID102094			-0,048672	-0,760897	-0,045898	-1,349677	-1,385184	-1,416990	0,634714	1,213480	0,855920	1,274056	0,897411	0,450251
ID412			0,209533	-0,652613	0,068512	-1,225387	-1,576372	-1,042092	1,187293	1,210177	1,161716	0,704470	0,568633	-0,437753
ID416			-0,021673	-0,953352	-0,118209	-0,841362	-1,525669	-1,075006	1,100951	1,190600	1,122839	0,736987	0,954463	-0,386322
ID916			0,112633	-0,497010	-0,213149	-1,308472	-1,513149	-1,480887	1,180725	1,188996	1,052696	0,925643	0,626758	0,156628
ID769			0,137638	-0,561682	0,094175	-1,357386	-1,502715	-1,538648	1,047960	1,184940	1,082330	0,838281	0,638752	0,145927
ID181505			-0,287443	-1,006664	0,080803	-0,791859	-0,479695	-1,387369	1,614745	1,184320	1,113886	0,634627	0,184442	-0,701135
ID3763623			-0,177041	-0,533866	-0,040323	-0,434310	-0,801478	-1,087709	0,956320	1,178952	1,403758	0,447766	0,214519	-1,014647
ID662	GRP78		-0,619564	-0,139439	0,108500	-0,656662	-0,963329	-1,148933	-0,399128	1,177553	0,083716	2,966097	0,205298	0,127415
ID594	GRP78		-0,576442	-0,820201	-0,216369	-0,781551	-1,028173	0,409619	1,203050	1,169118	1,153571	0,506201	-0,192183	-0,700089
ID683	SYK	TRFL	-0,193313	-0,708576	-0,248869	-0,776111	-1,189151	-1,011670	0,790920	1,168920	0,935017	0,465139	0,678182	0,205795
ID418			0,343000	-0,361664	0,255471	-1,399601	-1,593898	-1,303391	1,124898	1,160623	1,083534	0,572424	0,649630	-0,387925
ID1844173			-0,132014	-0,643179	-0,505030	-0,925802	-1,386912	-1,425390	1,408441	1,158894	1,370104	0,575913	0,493576	0,155378
ID841			0,049114	-0,506739	-0,324925	-1,310988	-1,516691	-1,437100	1,136827	1,152684	0,968667	1,069611	0,740505	0,246437
ID419			0,350313	-0,346157	0,256486	-1,517459	-1,573225	-1,126883	1,218727	1,150507	1,098920	0,597963	0,481911	-0,441613
ID3078712			-0,039548	0,245659	-0,074846	-0,470923	-1,620388	-0,670338	0,857034	1,143785	1,341638	-0,167791	-0,694900	0,108672
ID265686			0,433959	-0,026746	0,500307	-0,950295	-1,082695	-2,032425	0,855500	1,128254	0,937516	0,045480	0,327339	-0,124825
ID417	K1C9		0,361908	-0,506228	0,221497	-1,257205	-1,547369	-1,325425	1,004227	1,123651	1,033632	0,792330	0,706645	-0,409580
ID2076081			-0,017384	-0,373703	-0,004795	-0,157874	-0,039047	-1,445490	0,536148	1,119449	1,118362	0,211970	0,125200	-1,019845
ID589226			-0,312609	-0,301509	-0,483156	-1,057662	-1,417516	-0,738711	0,432868	1,110728	1,146581	0,739663	0,703548	0,362691
ID748			0,163880	-0,525364	-0,182639	-1,300930	-1,551878	-1,508938	1,086615	1,100571	1,051180	0,929526	0,763802	0,206556
ID745	CALR	KAP0	0,123400	-0,561357	-0,185117	-1,302078	-1,518948	-1,462124	1,180566	1,086276	1,174948	0,859414	0,666701	0,153173
ID39482			0,401458	-0,424579	0,296777	-1,409892	-1,728883	-0,887076	1,282131	1,083882	1,038937	0,663153	0,341422	-0,491542

Table S-4: cluster 1

ID[)1]	Protein[)2]		co_0 min	co_30 min	co_60 min	co_24 h	co_48 h	co_72 h	PI_0 min	PI_30 min	PI_60 min	PI_24 h	PI_48 h	PI_72 h
ID729			-0,210013	-0,524211	-0,219550	-1,185680	-1,255215	-1,240521	1,404051	1,067167	1,262727	1,066546	0,229890	-0,128555
ID668	CPNE1	GRP75, PDIA4	-0,178457	0,126805	-0,101334	-0,792066	-1,957800	0,226291	0,954580	1,057943	0,997437	1,055733	-0,503770	-0,621428
ID57776			-0,380779	0,023237	-0,413100	-1,949458	-0,756423	-0,333946	0,826454	1,052364	1,002497	1,505684	0,145323	-0,345432
ID3479720			0,036180	-0,692903	-0,389182	-0,977644	-1,805944	-1,073013	1,230820	1,020911	1,181686	0,845228	0,683778	0,151388
ID43845			-0,463542	-0,776744	-0,582574	-0,988304	-0,800939	-0,290239	0,831649	1,020201	1,089290	2,179064	-0,317712	-0,355384
ID660	PDIA4		0,296611	-0,195235	0,093785	-1,297337	-2,280825	-0,681453	0,916921	1,020081	0,985132	0,160280	0,674375	0,347736
ID747			0,195357	-0,514842	-0,180459	-1,293442	-1,588822	-1,523284	1,056979	1,010542	0,946096	1,030485	0,851059	0,267952
ID20414	HS90A		-0,296922	-1,128648	-0,450060	-0,742138	-1,491897	-0,994312	0,583417	1,002894	0,665285	1,157586	1,258353	0,725838
ID628	HS90A		-0,016201	-0,578391	-0,111003	-1,363913	-1,663706	-1,131882	1,056513	1,000707	1,094448	0,967619	0,816646	0,171067
ID1867746			0,291550	-0,030055	0,373661	-0,388093	-1,036375	-1,570222	0,188980	1,000191	0,693194	-0,263628	1,247836	-0,572947
ID1811207			-0,219877	-0,531917	-0,406545	0,011453	-1,995708	-0,856166	1,609767	0,997056	0,963298	0,382573	0,239599	-0,097889
ID697	SDC10	AGM1, NDUS1, WDR72	-0,015159	-0,310147	-0,215831	-1,441901	-1,802065	-1,119743	1,155543	0,996929	0,776987	0,359594	1,045438	0,660253
ID3549612			-0,276906	-0,709561	-1,151986	-0,871447	-0,781312	-0,940930	1,014176	0,995331	0,938438	0,789621	0,769832	0,422150
ID203683	TXND5	ACL6A	0,082559	-0,004359	0,368571	-1,203443	-1,234896	-1,674754	0,492551	0,975845	0,997281	1,979858	-0,230621	-0,053625
ID49585			-0,467553	-0,825675	-0,802098	-0,802419	-1,501253	-1,072547	0,960325	0,975171	1,205418	1,283909	0,936069	0,431630
ID3762281			0,278271	-0,516944	-0,000305	-0,099197	-0,749779	-1,357675	1,036391	0,946931	1,280323	0,759051	-0,172156	-1,215148
ID1187			-0,477885	-1,065440	-0,365943	0,236184	-0,737791	-0,703611	1,391486	0,906584	0,497698	0,370697	-0,255652	0,296347
ID3477702			0,177167	-0,576922	-0,230421	-1,208607	-1,646608	-1,396702	1,088631	0,900799	0,907946	1,086916	0,942049	0,227479
ID17482	GELS		0,040060	-0,874507	-0,231494	-1,969921	-0,923549	0,913135	0,686311	0,889361	1,303638	0,241142	0,656939	-0,670830
ID717	HSP71	PDIA4	-0,264289	-0,938518	-0,278773	-1,209997	-1,569558	-1,125475	0,995499	0,878662	0,822534	1,247301	1,068435	0,686003
ID627	HS90A	MRE11, FETA	-0,441274	-1,069828	-0,470449	-0,745703	-1,581884	-0,844675	0,819740	0,870661	1,149115	1,150941	1,094202	0,356889
ID722			0,195126	-0,540884	-0,141222	-1,275305	-1,641662	-1,479983	1,093288	0,862310	0,859510	1,108950	0,921582	0,315527
ID1915149			-0,294499	-0,650827	-0,039786	-0,224859	-1,271054	-0,402454	1,247283	0,861979	1,284538	-0,602528	0,341202	-0,399626
ID3432480			-0,024743	-0,199281	-0,004439	-0,582014	-0,946338	-1,444945	-0,103087	0,860939	0,049926	1,606557	1,009379	0,179684
ID866			-0,198115	-0,812088	0,497162	-0,691901	-0,906955	-0,621828	1,003235	0,846430	1,459664	-0,931718	0,018976	0,104208
ID380472	DCTN1		-0,208175	-0,946275	-0,264976	-0,772155	-1,404563	-1,689537	0,932503	0,842356	0,984073	1,076385	1,009982	0,709479
ID2174	CALR		0,051877	0,554703	0,059484	-2,001405	-1,497724	-0,043838	0,391683	0,824401	1,116346	1,579877	0,085933	-0,726367
ID74132			0,242845	0,095946	1,044330	-2,066225	-0,383287	-0,572654	0,732828	0,800799	0,964382	0,894673	-0,578523	-0,951446
ID1119	STML2	ACTG	-0,566440	-0,298743	-0,156058	-0,968145	-1,047588	-0,929875	1,038252	0,760737	1,231883	2,417327	-0,210290	-0,666731
ID1814782	ID919		1,046915	0,010336	-1,180253	-0,871546	-0,801523	-0,973443	0,736028	0,721694	0,682649	0,351133	0,373470	-0,007678
ID22159	GRP75	PDIA4, HSP71	-0,297366	-0,571073	-0,375830	-1,084509	-1,684081	-1,203869	0,577578	0,715427	0,636425	1,392130	1,396348	0,846853
ID406			-0,051827	-0,997425	-0,203662	-0,221364	-1,463288	-1,140570	1,263562	0,640631	1,268625	0,599457	0,832866	-0,377141
ID715			-0,002296	-0,289550	-0,150858	-1,242506	-1,913824	-1,265196	0,922546	0,636680	0,474561	1,369334	0,926600	0,876843
ID1841741			0,183485	-0,434620	0,263366	-0,707233	-0,926939	-2,148283	0,929072	0,623348	1,005159	-0,038959	0,629717	0,816196
ID1821608			0,264857	0,028092	0,237457	0,155629	-1,860496	-0,226301	0,770833	0,583121	0,569931	1,446366	-1,093542	-0,514356
ID1843456			-0,055463	-0,382704	-0,016922	0,783913	-0,333169	-2,393663	0,841167	0,576252	0,825643	-0,338385	0,253906	0,206436
ID2687300	RCN1		-0,551568	-0,117402	-0,458858	-2,169156	-1,175312	0,624434	0,465780	0,527705	0,997925	1,624825	0,222999	0,414834
ID691	MAOX	SWP70, PDIA3	0,142448	-0,084226	-0,360552	-0,910077	-1,615458	-1,246145	0,850878	0,251823	0,278774	-0,418714	1,500114	1,506458
ID443			0,278178	-0,538730	0,395021	-1,990047	-1,025715	1,374878	-0,179205	0,163948	0,407496	0,599602	1,078897	-0,414423
ID1840030			0,208893	-0,399138	0,308887	-0,814702	-0,855481	-2,083886	0,578644	0,161827	0,781773	0,271008	0,405655	1,504272
ID2662678	RCN1		-0,731202	0,570370	-0,511123	-1,686911	-0,646888	0,140730	0,444615	0,058048	1,335605	2,136276	-0,087487	-0,487964
ID401246			0,265826	-0,035797	0,413791	-0,610452	-1,729785	0,038523	0,067969	0,017501	1,325156	1,271707	0,057863	-0,764374
ID693	COR1B	CBR4	0,084141	0,008373	-0,284709	-0,679870	-2,143035	-0,925571	0,734443	-0,107078	-0,012143	0,687190	1,374461	1,435597
ID580	CH60	HNRPK, ACTB, TBAK	0,075308	0,048952	-0,845567	-0,294356	-0,508326	-1,395892	0,549290	-0,115742	0,877287	2,095180	0,154317	-0,116657
ID444662			-0,177649	-0,151608	0,077789	-0,572239	-0,742190	-1,416680	0,018832	-0,190586	-0,036739	3,115427	0,249872	0,604628
ID1683			-0,887195	-0,801006	-0,502811	-0,291966	-0,405383	-0,687885	1,187068	-0,500318	1,100036	0,669786	0,953010	0,334108

Table S-4: cluster 2

Cluster 2 ID[1]	Protein[2]		Z-score[3] co_0 min	co_30 min	co_60 min	co_24 h	co_48 h	co_72 h	PI_0 min	PI_30 min	PI_60 min	PI_24 h	PI_48 h	PI_72 h
ID824	PDIA1		-1,161790	-0,796705	-0,824941	-0,680127	-0,068859	0,352550	0,171206	0,254227	0,607868	3,122829	0,116008	-0,311557
ID431675			-0,867080	-0,684028	-1,165192	-0,274875	-0,642079	-0,612647	0,624346	0,109390	0,643140	2,879720	0,469380	0,239853
ID661	GRP78		-1,093735	-0,686442	-0,400916	-0,407371	-0,769975	-0,501842	-0,406720	0,262389	-0,124723	2,869278	0,941311	1,036065
ID2111	GLU2B		-1,057631	-0,258775	-1,030801	0,297765	-0,827119	0,182188	-0,211071	-0,593630	0,641507	2,833903	0,319307	0,412833
ID2239	PDIA1		-1,330555	-1,203592	-1,005480	-0,172862	-0,109590	0,271340	-0,018601	-0,108087	0,113980	2,810114	0,796577	0,659285
ID280677	RCN1		-0,790352	-0,420589	-0,835915	-1,464612	-0,773972	0,785915	-0,081255	0,765764	0,630455	2,591490	0,061658	0,179286
ID7106			-0,760148	-0,542449	-0,695061	-0,357462	-0,302040	1,547827	-0,672813	-0,801212	-0,517205	2,553799	0,722692	0,462522
ID351055			-1,211569	-0,782630	-1,157768	0,555068	0,260266	1,316291	0,134575	-0,630919	-0,487013	2,518835	0,259034	-0,144461
ID982	PDIA6		-1,610032	-0,912071	-1,046421	-0,395639	-0,187053	0,382693	0,419165	0,309059	1,039428	2,502389	-0,139144	0,263221
ID3760	GRP78		-1,311318	-0,897379	-0,611853	-0,246064	-0,506827	-0,169374	-0,378203	-0,052264	-0,150377	2,418678	1,098909	1,410742
ID92901			-1,586898	-0,847773	-0,815954	0,509914	0,821452	0,412517	-0,521558	-0,254713	-0,424127	2,416390	0,380934	0,513912
ID3485928			-1,387413	-1,529101	-1,146786	0,102861	0,021986	0,436940	-0,045610	0,038244	0,074423	2,337018	0,868767	0,812925
ID2740977			-1,156788	-1,181089	-0,595869	0,086536	0,034692	0,806955	-0,466014	-0,940152	-0,185056	2,322394	0,947693	0,907295
ID3616			-1,013204	-1,094160	-0,892029	-0,795899	-0,352828	0,229479	0,274320	1,232740	0,656862	2,219612	0,567206	-0,477197
ID864	PDIA3	VATB2	-1,263975	-0,966387	-0,959346	0,596354	0,581174	1,529880	-0,116672	-0,804632	-0,433958	2,194944	0,186992	0,004364
ID71445			-1,051772	-0,471800	-0,381339	-0,388641	-0,893719	-0,552486	-0,666859	-0,026242	0,087178	2,178840	1,243960	1,467590
ID2845794	GRP78		-0,960223	-1,524584	-0,661458	0,179850	0,146642	0,626318	-0,241127	1,244573	-0,296148	2,128819	0,015416	-0,125873
ID827	CH60		-1,391783	-0,873379	-1,005551	-0,528177	-0,558934	-0,078398	-0,160607	-0,204127	1,380922	2,098363	1,039882	0,806378
ID723	HSP71	LMNB2, GRP75	-1,656556	-0,591632	-0,901945	0,014589	-0,048563	0,826486	1,072830	-0,522149	-0,286474	2,066098	0,783251	-0,239410
ID122740	DP13B		-0,959009	-0,676984	-0,700237	0,634135	0,237716	0,882766	-1,110150	-0,858481	-0,906139	2,047431	0,721924	1,198885
ID787	HNRPK		-1,431187	-1,151964	-1,044926	0,833210	0,042227	-0,184375	-0,580582	0,258516	-0,272709	2,015931	1,427906	0,591937
ID19644	TTL12		-0,385210	-1,081983	-0,611818	0,699317	-1,202986	-0,924509	-0,243158	0,727516	0,063300	1,810378	1,042129	0,559620
ID535941			-0,212856	0,010225	-0,262119	-0,685589	-0,113913	0,295547	-0,865848	-0,588931	-0,477869	1,749561	1,040380	0,548804
ID562964			-0,200793	-0,524720	-0,083709	0,229725	-1,696112	0,747299	-0,226086	0,485235	0,089847	1,684991	0,823113	-0,907541
ID2354041			-1,575757	-0,974258	-0,971855	-0,136614	0,694364	1,067769	-0,822643	-0,133110	-0,123400	1,682574	0,701724	1,011850
ID444			-0,245657	-0,831524	0,021779	-0,940934	-0,618847	1,014918	-0,505556	-0,248726	-0,230168	1,677865	1,634769	-0,308453
ID86593			-1,532790	-0,786303	-0,961170	0,991277	0,087746	1,340766	-0,645381	-0,467515	-0,274860	1,634500	0,702712	0,319643
ID138687			-0,745369	-1,401360	-1,241053	-0,088626	0,196350	1,757328	-0,190907	0,753109	-0,201047	1,625375	0,272516	-0,329973
ID694	HSP7C	LMNB1	-1,386032	-1,112147	-1,025922	1,000932	0,529395	1,428604	-0,089321	-0,897003	-0,743371	1,517293	0,681416	0,475480
ID1345	ANXA5	SFRS2	-1,568492	-1,536176	-0,842175	-0,706978	-0,349605	0,674438	0,793936	0,820254	0,858464	1,481328	0,602625	0,142714
ID1761			-1,365537	-1,084511	-0,550999	-0,408461	-0,219512	1,704587	-0,768430	1,255207	-0,397632	1,463768	0,360471	0,376991
ID672	GRP78		-1,028770	-1,582243	-1,081194	0,636128	0,869090	0,958393	0,297801	-0,120323	-0,319624	1,431116	0,225970	0,071434
ID826	CH60	STK3	-1,361622	-1,789609	-0,894443	-0,117941	-0,410639	0,325896	0,807200	0,792463	1,329319	1,421914	0,397180	-0,144238
ID1251	EF1D		-0,728700	-0,007527	-0,731919	1,560372	0,388047	-0,697658	0,253067	-0,659646	-0,474413	1,411521	-0,039818	0,079552
ID603	HS90A		-1,358367	-0,788269	-0,793262	0,698590	0,533437	1,286602	-0,331946	-0,924256	-0,300552	1,404035	0,893054	0,031944
ID596190	HS90A		-1,136497	-1,134501	-0,462641	0,295115	0,382825	1,722841	-0,599360	-1,038911	-0,758592	1,331710	0,764747	0,966192
ID193389			-1,268006	-0,697860	-1,415414	0,218982	0,399461	1,188918	1,105989	-0,541901	0,166707	1,302139	0,205767	-0,339247
ID3763695			-0,527880	-0,360055	-1,443300	0,253844	-0,040967	-0,911551	0,982306	-0,538281	1,026812	1,300692	0,304329	0,279225
ID1978481			-1,261859	-0,682243	-0,686154	0,617130	0,451055	0,915336	-0,704101	-0,860560	-0,941085	1,299049	1,079029	1,099166
ID1073	ACTG	ACTB	-1,544467	-1,371092	-1,140403	1,135285	0,720399	1,494302	-0,108743	-0,394912	-0,384265	1,245362	0,284211	0,375665
ID1055	ACTG	IF34	-0,961770	-0,880139	-1,442083	1,406817	-0,017124	-0,478872	-0,775300	0,503251	0,658512	1,244200	0,791213	0,262345
ID883			-1,023766	-1,370637	-1,450093	0,873243	0,849663	1,300983	-0,175368	0,548579	0,595069	1,203188	-0,214858	-0,835206
ID556498	KBP		-1,201483	-1,166364	-0,804632	0,804764	0,184603	2,126115	-0,015303	-0,855465	-0,652650	1,190671	0,407193	0,280220
ID897	CALR	NP1L1	-0,818983	-0,042810	-0,919975	-0,677454	0,551574	2,268502	-0,914064	-0,979204	-0,266014	1,168947	0,243015	0,678703
ID2889441			-1,708776	-1,193289	-1,590044	0,475705	-0,088060	0,773387	0,490601	0,464275	0,507159	1,107100	0,655113	0,383604
ID254557			-0,299959	-0,955300	-0,371440	-1,039180	-0,245505	1,179168	-1,019478	0,973092	0,100823	1,099186	1,375461	-0,522071
ID450085	PEPD	PDIA3, TBA1B	-0,997249	-0,404108	-1,139894	0,831381	0,099149	1,970825	-0,284099	-1,314470	-0,528573	1,042384	0,825226	0,160023
ID1082			-1,815328	-1,300657	-1,307281	0,836728	0,562234	0,983923	0,168217	0,017068	-0,042560	1,040515	0,526914	0,590356
ID2100931			0,030507	0,106260	0,042688	-2,115234	-0,308876	0,399355	-0,752084	0,174939	-0,411384	0,992228	1,337187	0,752471
ID66151			-0,955092	-0,795814	-0,425142	0,198198	0,831599	-0,049029	-0,721039	-1,061703	-0,803432	0,990201	1,315762	1,723042
ID587			-1,344632	-1,284523	-1,494395	0,011168	-0,718720	1,264715	0,494399	0,532480	1,050575	0,966024	0,110605	0,653807
ID147614			-1,638300	-1,025354	-0,870252	0,861467	0,584285	1,071039	-0,091340	-0,373168	-0,412760	0,910190	0,612834	0,598908
ID1795			-1,172989	-1,060981	-0,795935	0,379162	0,983747	1,455321	-0,839151	0,198005	-1,035132	0,872251	0,209697	1,024069
ID808	CPNE3	CPNE1, PDIA3	-1,468988	-1,091227	-0,757794	1,182430	0,664742	1,208280	-0,480453	-0,154130	-0,122117	0,858416	0,662242	-0,286795
ID582			-0,975980	-1,503693	-1,264091	-0,364986	-0,945679	0,980028	0,758247	0,940483	1,272360	0,850982	0,111371	0,353704
ID3091679			-1,540914	-1,263534	-0,836924	0,492773	0,367292	0,855301	0,207707	0,038457	-0,080687	0,843745	0,483721	0,643999
ID3062814			-0,590262	-1,453695	-0,238255	0,570846	1,162827	1,199203	0,076179	0,073142	-0,232094	0,840238	-0,373414	-0,824655

Table S-4: cluster 2

ID[1]	Protein[2]		co_0 min	co_30 min	co_60 min	co_24 h	co_48 h	co_72 h	Pl_0 min	Pl_30 min	Pl_60 min	Pl_24 h	Pl_48 h	Pl_72 h
ID899	K2C8		-1,639164	-1,820062	-1,331968	0,640234	0,620716	1,050564	0,301883	-0,056275	0,244543	0,786410	0,712775	0,686945
ID14770	ACTG	SET	-1,576538	-1,072335	-1,345141	1,335032	0,971400	1,393588	-0,122559	-0,189328	-0,581357	0,710452	0,254532	0,399865
ID1846930			-1,692906	-0,870808	-0,935607	-0,140787	0,881732	0,180376	-0,704714	0,644484	0,736850	0,675950	0,669991	0,724425
ID2649893			-0,702398	-0,833545	-0,913408	1,436382	0,465269	1,382214	0,215675	0,039998	0,624241	0,672210	-1,024278	-1,194308
ID581			-0,650727	-1,194296	-0,305537	-0,589529	-1,372089	1,594932	0,277607	0,691159	1,096379	0,662723	-0,145956	0,101014
ID2037342			-1,102044	-1,318839	-0,590207	0,439899	-0,267816	2,229526	-0,421428	-0,334166	-0,108263	0,655714	0,211223	0,770328
ID1706	MRL	MLRM	-1,785253	-0,784614	-0,920631	0,929147	0,177835	0,905596	1,445279	0,124838	0,639651	0,581859	-0,486112	-0,682130
ID120878	GRP78	PDIA4	-1,043505	-0,818182	-1,186487	1,032561	0,538694	1,380507	0,737286	-0,825670	-0,309414	0,547104	0,276060	-0,192178
ID185058			-1,341989	-0,868082	-1,077716	0,371356	0,674788	1,407457	1,408696	-0,258370	0,070141	0,534494	0,006070	-0,793221
ID3761695			-1,918702	-0,586808	-1,012507	0,800462	0,186424	0,881069	0,031691	-0,069857	0,426408	0,529453	0,526326	0,338403
ID288865			-0,773357	-0,570625	-1,333615	0,169610	0,497711	0,339728	-0,599239	1,047820	-0,606036	0,525785	0,449044	0,984621
ID2035512			-0,573416	-0,925991	-0,115988	0,649323	-0,373023	2,039989	-1,103695	-0,392075	-0,593860	0,495686	0,247937	0,769034
ID1302			-0,887439	-0,853709	0,393435	0,485349	0,476892	0,477717	-0,228826	-0,909379	-0,239198	0,445533	0,402989	0,401765
ID132924	ENPL		-1,366272	-0,722936	-1,720218	1,155855	0,912111	1,114700	0,338603	-0,742321	0,634700	0,442028	0,045059	0,019198
ID1542			-0,359063	-1,686299	-1,005629	0,477161	0,480971	0,488070	-0,313748	0,325497	0,382522	0,438867	0,442599	0,438768
ID720	HSP71		-1,060705	-1,100765	-1,159064	1,154054	1,435559	-0,166077	0,582735	-0,487028	-0,673562	0,389316	0,979281	0,203585
ID2138752			-1,555446	0,138243	-0,073490	0,197309	0,175986	0,175743	0,180400	-0,017771	0,113705	0,346571	0,206249	0,199143
ID1615			0,373764	-0,326070	-1,090171	0,430524	0,436081	0,406641	0,353673	0,323463	-0,353945	0,344617	-1,126638	0,314216
ID2099065			-0,746912	-0,731410	-0,704250	1,659517	0,397080	-0,118620	-1,373243	0,247759	-0,874119	0,317808	1,521044	0,484797
ID2330			-1,113903	-1,282002	-1,236868	0,336930	-0,289009	0,416569	0,536373	0,626935	0,306049	0,281311	0,347788	1,140155
ID1094			-1,042086	-1,429392	0,198087	0,247377	0,241969	0,279985	0,166999	0,173649	0,163207	0,255744	0,218016	0,233032
ID2202941			0,200272	-0,773973	-1,777507	0,271871	0,258268	0,264154	0,290813	0,287705	0,289400	0,254586	0,261975	0,236083
ID429069			0,081483	0,095424	-1,611401	0,253833	0,239612	0,238645	0,063400	0,002217	-0,011336	0,254453	0,230732	0,226551
ID12822			-0,955310	-1,156825	-1,415286	1,315552	0,764028	0,454097	-0,714590	0,076885	-0,521077	0,237837	0,893959	1,080190
ID44539	MARCS		-0,697584	-1,302411	-0,733598	-0,397560	-0,388969	0,879899	0,623400	0,638233	0,864900	0,210463	-0,792258	1,148101
ID3329884	ARP3	TADBP	-0,036091	0,070904	-1,571724	0,146288	0,133651	0,139501	0,248326	0,216035	0,206550	0,175334	0,160755	0,154304
ID15904	TBAK		-1,119401	-0,529518	-0,731832	0,214373	1,075835	1,814919	0,724164	0,721931	-1,106329	0,107307	-0,427319	-0,717303
ID2148299			-0,705510	-0,718430	-0,803080	0,419588	-0,810060	1,015437	0,895312	0,488464	0,918363	0,077274	-0,014335	-0,743705
ID3413662			-1,448990	-1,046274	-0,385931	0,025170	0,362648	1,950327	0,628458	0,754148	-0,003450	0,052116	-0,174044	-0,701150
ID140457			-1,014657	-1,719992	-0,450693	0,479557	0,422352	0,182419	0,877896	0,616677	0,506228	0,031572	0,137580	-0,061046
ID200032			-1,135615	-0,545042	-0,354957	-0,243067	1,060627	1,584078	-0,132111	-0,427466	1,168753	0,016540	-0,021710	-0,965894
ID892	K2C8		-1,721537	-1,376848	-1,224211	0,164619	0,478974	1,443342	1,064572	0,799248	0,809924	0,002316	-0,177976	-0,261844
ID1650			-0,695441	-0,050353	-0,743204	0,190993	0,831996	1,259641	-1,642407	1,868930	-0,989171	-0,004776	0,257422	-0,284823
ID47508			-1,553781	-0,978508	-1,194148	1,537183	1,366449	1,296520	0,264453	-0,392386	-0,293600	-0,059996	0,007781	-0,014965
ID129069	DDB1		-0,850040	-1,242357	-0,537493	0,727637	1,137500	2,073128	0,286106	-0,093094	-0,447807	-0,064382	-0,303662	-0,701631
ID235608	COR1B		-0,946221	-1,458733	-1,213801	-0,299966	0,415698	1,672449	1,070008	0,309385	0,010340	-0,176783	1,067416	-0,493988
ID61022			-1,689689	-0,689682	-1,397576	0,730709	0,272599	-0,104232	0,014549	-0,072171	0,950541	-0,200909	1,139637	0,996000
ID3163353			-1,366256	-1,298710	-0,953662	0,351644	1,226882	1,627397	1,357889	-0,484317	-0,190759	-0,263805	-0,015292	-0,056961
ID1989367			-0,759511	-1,155698	-0,756396	-1,565540	0,672419	0,066960	0,250891	0,476378	0,570167	-0,290071	0,596173	1,821711
ID17545	MX1		-0,031203	-1,034900	-0,354199	-1,506540	-0,269046	2,001076	0,051539	0,962335	0,452063	-0,319063	0,883022	-0,914850
ID3763473			-1,194950	-1,465314	-0,960445	0,250267	0,384297	0,469019	-0,117763	1,666427	0,607293	-0,336332	0,766767	-0,153351
ID595			-0,565275	-0,526821	-0,378324	0,376141	0,567469	-0,395053	-0,818103	-0,627062	-0,431626	-0,336437	2,470522	0,580460
ID1490			-0,649155	-0,612302	-0,184487	0,191021	0,681107	-0,373250	-0,951835	0,711670	-0,627256	-0,356757	0,915609	1,166445
ID1933424			-0,704930	-0,566660	-0,833043	-0,432968	1,061739	0,737056	-0,496084	-0,820521	-0,657916	-0,402344	0,566802	2,448285
ID1935188			-0,872011	-0,389031	-0,339018	-0,772963	0,991361	0,131530	-0,327699	-0,615229	-0,296869	-0,453541	0,437768	2,392316
ID3762592			-1,676157	-1,344182	-1,095019	-0,055962	0,383433	1,350788	1,415935	0,832841	0,387234	-0,500835	-0,179754	0,356469
ID121837			-0,533516	0,204156	-0,800621	0,778830	-0,657176	1,135144	-0,097699	-0,330304	0,804694	-0,536949	0,194167	-0,294962
ID1654			-0,972929	-0,923826	-0,907657	1,231698	1,261294	1,148175	-0,229092	-0,304762	-0,127055	-0,673291	0,235884	0,093237
ID483852			-0,444549	-1,124195	-1,035568	0,534399	-0,600689	0,300432	-0,336264	0,590853	-0,400269	-0,677095	2,250869	0,772804
ID1994828			-0,578754	-0,472995	-0,318739	0,241603	-0,178965	-0,221664	-0,163340	-0,137750	0,000608	-0,885289	0,194559	3,260432
ID290046			-1,072439	-0,070946	-1,037173	0,609524	0,184578	1,880625	1,096086	-0,459540	-0,771907	-1,357735	0,833971	-0,174477

Table S-4: cluster 3

Cluster 3			Z-score[3]											
ID[1]	Protein[2]		co_0 min	co_30 min	co_60 min	co_24 h	co_48 h	co_72 h	PI_0 min	PI_30 min	PI_60 min	PI_24 h	PI_48 h	PI_72 h
ID376	HYOU1		-0,682113	-0,114028	-0,238009	-0,583871	-0,454718	0,629152	-0,395685	-0,385885	0,048431	3,497170	-0,030543	-0,415609
ID482337			-0,419584	-0,330757	-0,433454	0,374246	0,138109	0,442937	-0,611321	-0,816345	-0,664648	3,394501	-0,141428	-0,083631
ID211213			-0,740160	-0,489291	-0,482426	0,109191	0,285855	0,029006	-0,872581	-0,437135	-0,567158	3,249910	0,362945	0,364321
ID3737	ATPB		-0,027307	0,586098	-0,171628	-0,256554	-0,235577	-0,002926	-0,974208	-0,921806	0,021032	3,205411	-0,114563	-0,306620
ID658	GRP78		-0,564444	-0,630420	-0,423998	0,303187	0,231778	-0,642790	0,368837	-0,070073	-0,102734	3,202358	-0,332415	-0,538697
ID2522			-0,442667	0,123526	0,204894	0,415185	0,183491	-0,254611	-1,000001	-1,233507	-0,427327	3,181302	0,223829	-0,178789
ID594301			-0,383024	0,258418	-0,365593	0,546862	0,138334	-0,146502	-0,620406	-1,502312	-0,697192	3,023984	0,122799	0,380628
ID673	SYG	DPYL2	0,041329	0,217918	0,122692	0,416898	0,370466	0,526268	-1,259262	-1,069098	-1,088482	2,942558	-0,427790	-0,057857
ID854	PDIA3	HNRPF, VATB2	-1,146338	-0,565011	-0,993779	-0,315253	0,085405	1,206453	0,278475	-0,287360	0,471103	2,903679	-0,346206	-0,565248
ID325			-0,124958	0,439594	-0,302873	0,662178	0,235357	-0,120455	-0,239682	-1,733035	-0,110051	2,847197	-0,174685	-0,666789
ID9421	SF3B2		0,074335	-0,277232	0,031493	-1,010045	0,137133	1,163784	-1,107459	-0,161003	-0,345590	2,788676	0,070558	-0,667479
ID1433	PSME1	6PGL, ERP29	0,229130	0,884715	0,706377	-0,627165	-0,752765	0,347797	-0,410589	-0,124819	0,085167	2,777781	-1,095060	-1,326125
ID274688	ENPL		-0,609945	-0,328620	-0,545800	-0,347265	-0,880492	0,183231	0,164287	1,095042	0,485802	2,770275	-0,501398	-0,792547
ID3762278			-0,460783	0,008776	0,354719	0,310103	-0,273256	0,820839	-0,304505	0,235230	-1,361092	2,677408	-0,219096	-1,118991
ID1037	CALU	ACTG	-0,605903	1,083832	-0,596194	-0,561682	0,146655	0,263655	0,143425	-1,384669	0,842091	2,635858	-0,822712	-0,485390
ID10509	LG3BP		-0,424498	0,322287	-0,464287	-0,686155	-0,346705	0,337241	-0,736289	-0,830920	-0,519959	2,598696	1,009928	0,390337
ID6678			-0,156348	0,312952	-0,435789	0,877398	0,769139	0,551810	-0,809275	-1,577511	-0,521227	2,563620	-0,274106	-0,659758
ID543455			0,258511	0,134841	0,259410	-0,082826	0,836027	0,976028	-0,971619	-0,938610	-0,769597	2,562312	-0,303681	-1,320217
ID625	EZRI	CALD1, SYK	-0,232128	0,024604	-0,017983	0,751288	0,447759	0,625510	-1,047103	-1,241922	-0,876201	2,559498	-0,265988	-0,087457
ID1373	PSME2	1433E	0,067351	0,283399	0,532166	-0,625861	-0,242545	0,188856	0,234268	0,097358	0,740930	2,539775	-1,587447	-1,593307
ID16921			0,225392	0,642914	0,737566	1,111596	0,050964	-0,408335	-0,423229	-1,192696	-0,854766	2,462580	-0,458355	-1,277987
ID2680471	RCN1	SPRC	-0,336860	0,499883	-0,313602	-1,025741	-0,702289	1,113358	-0,100760	0,499930	0,632701	2,406988	-1,247664	-0,824197
ID9232			0,463691	0,407091	0,494715	0,167891	0,852477	0,222905	-0,571095	-1,216602	-1,002047	2,394110	-0,294149	-1,320459
ID710	HSP7C	HSP71, GRP75, TF65	-1,206980	0,098621	-0,712948	0,392157	-0,503313	1,322294	0,395700	-0,568009	-0,654031	2,366497	0,025645	-0,364008
ID989	KAP0	TXND5	0,156964	0,971065	0,493017	0,223557	0,371018	0,637389	-1,083921	-1,058284	-0,765794	2,332085	-0,830002	-0,864074
ID364	HYOU1	OXRP	0,218409	1,002231	0,624823	-0,265214	-0,162868	0,200642	-0,608399	0,122354	0,243270	2,291821	-1,367719	-1,726396
ID546917			-0,202314	-0,879010	-0,199240	-0,635668	-0,104010	1,529247	-0,497803	0,594050	-0,715729	2,245539	0,171430	-0,745106
ID195754	ITA3		0,178838	0,293174	0,184257	0,203251	1,108849	1,035137	-0,700117	-0,997203	-0,866071	2,225893	-0,698966	-1,410570
ID6662			0,080096	0,534060	-0,262500	0,940893	0,900834	0,757582	-0,897459	-1,444066	-0,597248	2,211151	-0,748809	-0,921746
ID28319	SYG	CALD1	-0,213971	-0,108382	-0,131719	0,531568	0,324053	0,237766	-0,702746	-0,319635	-0,697397	2,182322	-0,524700	-0,031580
ID462	ENPL		0,949723	0,451476	-0,160949	-0,595258	1,281255	-1,060319	0,092282	-0,478279	-0,628495	2,157434	-0,199950	-1,269563
ID25674	PLOD3		-0,251668	0,070381	0,128612	-0,567083	-0,379814	1,146307	0,627350	-0,352126	0,747311	2,135513	-1,687486	-1,083419
ID1663			-0,216159	0,282781	-0,483227	1,443308	1,053823	-0,863583	-0,321289	-0,998974	-0,260015	2,031071	0,325437	-1,485404
ID1024	CALU		-0,713702	0,509780	-0,696541	-0,226636	0,144047	2,058213	-0,196383	-0,910921	0,180561	1,994297	-1,302917	-0,341223
ID420	ACTG	ITA3	0,138769	0,538739	0,343606	0,270551	0,740577	1,255038	-0,336923	-1,293077	-0,868212	1,944290	-1,008574	-1,238712
ID408	ITA3		0,437265	0,486019	0,497623	0,635990	1,053072	0,360301	-0,481326	-1,164128	-0,974419	1,890399	-0,643678	-1,624518
ID1664			-0,525747	-0,237101	0,204760	0,663364	0,762732	1,373549	-0,970092	-0,907564	-0,241614	1,846967	-0,597489	-0,910023
ID810	PEPD	PDIA3, TBA1B	0,238360	0,370833	-0,828117	-0,007325	1,431192	-0,018746	0,709991	-0,257206	-0,591259	1,788388	-1,011201	-1,377813
ID957	F10A1	PDIA6, TXND4	0,144175	1,036939	0,746499	-0,461128	-0,319110	0,005697	0,165403	0,427001	0,471596	1,775326	-1,718343	-1,830223
ID1254	NACA	ANXA2	0,400186	1,300202	0,484183	0,889057	-0,057438	-1,410777	-0,322670	-1,592954	-0,582199	1,774048	-0,198149	-0,239976
ID55507	RCN1		-0,896373	-0,020777	-0,920388	0,181019	-0,059914	1,362277	0,364065	0,122933	1,126109	1,716327	-1,216923	-1,329273
ID53317	CALU		-0,780621	0,010411	-0,652700	-0,271944	0,214550	2,464816	-0,327865	-0,503355	-0,068515	1,681824	-0,960976	-0,385169
ID1027	CALU	ACTG	-0,366867	0,609675	-0,247683	-1,168935	-0,096374	2,225364	-0,218290	-0,207183	0,263221	1,623115	-1,376977	-0,633286
ID2068580			-0,386167	-0,059346	-0,409364	0,952249	0,352731	0,755615	-0,050502	-0,318303	-0,375446	1,586584	-0,022299	-1,629106
ID1033	TXND4	ACTG, PRS6B	-0,811263	0,276063	-0,071626	0,745945	1,460095	1,152232	-0,927162	-0,425152	-0,021793	1,535083	-1,379293	-1,149358
ID820	PDIA1		-0,607052	0,006046	-0,437879	0,155420	1,423498	0,327571	0,579819	0,001206	0,303952	1,504562	-0,726622	-2,154381
ID535	GLU2B		0,208543	1,074909	0,505648	-0,594850	-0,218291	0,147332	0,294853	0,071808	0,916847	1,474619	-1,438110	-2,074654
ID3761685			-0,548277	-0,309705	-0,551336	0,630440	0,484069	0,314284	0,262042	-1,779732	0,355422	1,472331	0,418896	-0,380353
ID630	PLOD3	IMMT, HS90B	-0,043995	0,410675	0,513233	0,377940	0,339566	-0,121865	0,849853	-0,808284	0,942295	1,420047	-1,647883	-1,876571
ID52561			-0,435668	0,526804	-0,512306	0,039518	0,101261	2,112906	-0,156124	-0,531725	0,590134	1,390282	-1,579804	-1,197707
ID1127	RCN1	SPRC	0,104889	0,549787	0,233922	0,028587	0,361906	1,427530	-0,631093	0,528165	-0,020532	1,328291	-1,667166	-1,912213
ID925	CALR	TBB2C	-0,066553	1,271407	0,193812	-0,726807	0,090306	1,707569	-0,874675	-0,346420	0,318216	1,238183	-1,529230	-0,966261
ID963			-0,966740	0,136444	-1,051399	-0,441740	0,731128	2,421832	-0,411089	-0,749825	-0,016670	1,190659	-0,630284	0,085351
ID54018	CALU		-0,511462	0,255737	-0,523592	0,211335	0,436950	2,119378	-0,109281	-0,215019	0,437185	1,182499	-1,524161	-1,463944
ID812	P4HA2	CPNE1	-0,645828	0,183679	-0,192183	1,740522	1,032006	0,906088	-0,766040	-0,712982	0,044299	1,130430	-1,192623	-1,244760
ID18275	GRP75	PDIA4	0,210159	0,622982	-0,363906	-0,118721	1,210361	0,465701	-0,089405	-1,205226	-0,792373	1,126603	-0,343207	-0,441316
ID1061	RCN1	ACTG	0,262210	1,495974	0,438542	-0,466201	0,121604	-0,052866	0,005366	-0,330013	1,099348	1,085655	-1,648423	-1,739782

Table S-4: cluster 3

ID[1]	Protein[2]	co_0 min	co_30 min	co_60 min	co_24 h	co_48 h	co_72 h	PI_0 min	PI_30 min	PI_60 min	PI_24 h	PI_48 h	PI_72 h
ID16614		0,651043	0,914792	1,282376	-0,187392	-0,629512	0,675663	-1,050405	-1,493375	0,491329	1,079539	-0,277278	-1,186895
ID10719	GRP78	-0,448500	0,373683	-0,386014	-0,528403	0,452391	-0,646824	0,810364	-2,089344	1,637155	0,985292	0,208655	-0,122132
ID278067	SPRC	-0,044829	0,717364	-0,390960	0,639890	0,502577	1,286908	-0,070908	-1,093669	0,924987	0,945366	-1,550788	-1,629598
ID574883		-0,720039	-0,417199	-0,190551	1,540755	0,307235	0,598262	-0,378390	-0,457386	1,073101	0,943373	-1,708600	-0,354719
ID2350362		-0,470983	0,184551	-0,316627	-0,382250	0,450873	2,828357	-0,629224	-0,336791	-0,482908	0,620806	-1,077996	-0,232606
ID2352301		-0,411922	0,813246	-0,009026	-0,202669	0,579633	2,357463	-0,591028	0,009074	-0,824739	0,587007	-1,334022	-0,826265

Table S-4: cluster 4

Cluster 4 ID[1]	Protein[2]		Z-score[3] co_0 min	co_30 min	co_60 min	co_24 h	co_48 h	co_72 h	PI_0 min	PI_30 min	PI_60 min	PI_24 h	PI_48 h	PI_72 h
ID589630			0,146398	0,147199	0,145347	0,178095	0,178232	0,182979	0,128322	0,112356	0,130739	-6,557567	0,145394	0,144332
ID1298			0,029488	0,184245	0,231592	0,712976	0,780460	0,536694	-0,055250	-0,443824	-0,054792	-5,923574	-0,167049	-0,273643
ID36428	U2AF2		0,205332	0,206978	0,207511	0,213912	0,209873	0,224758	0,210476	0,213517	0,209125	-4,639083	0,211578	0,206482
ID624	CALD1	IMMT	0,209230	0,210980	0,209903	0,220025	0,219612	0,223345	0,209796	0,196862	0,209167	-4,639024	0,203826	0,206765
ID855			0,236048	0,364620	0,436508	0,420355	0,267114	0,193618	-0,079260	-0,138733	0,173160	-3,882646	-0,044152	0,112044
ID811	U2AF2		0,255227	0,254841	0,256009	0,265266	0,259466	0,259823	0,256014	0,262834	0,258668	-3,788726	0,255616	0,257781
ID1393	GSTO1		0,260320	0,266571	0,264575	0,261984	0,263463	0,263348	0,243678	0,254362	0,253259	-3,788671	0,253021	0,256921
ID1592			0,253209	0,269993	0,252175	0,269675	0,261590	0,273783	0,244089	0,253621	0,258015	-3,788588	0,254022	0,251267
ID511			0,265909	0,266521	0,269258	0,267551	0,272991	0,273838	0,251753	0,239276	0,248254	-3,788452	0,243141	0,242848
ID27152			0,267803	0,268113	0,277163	0,254323	0,266104	0,272255	0,259840	0,242921	0,256071	-3,788427	0,240710	0,236017
ID401			0,262490	0,267909	0,265444	0,275722	0,276667	0,275863	0,246656	0,238522	0,233044	-3,788337	0,249081	0,249857
ID402			0,264500	0,268533	0,265294	0,276617	0,276993	0,275745	0,243657	0,235261	0,236292	-3,788313	0,248629	0,249715
ID361			0,263905	0,267887	0,271537	0,273391	0,272495	0,273674	0,252098	0,241612	0,239646	-3,788294	0,241404	0,243571
ID403			0,266860	0,271247	0,266987	0,279953	0,277738	0,275291	0,242510	0,234160	0,230679	-3,788199	0,247061	0,248663
ID466049			0,258538	0,264690	0,264283	0,279075	0,281256	0,286472	0,251026	0,237314	0,227940	-3,788129	0,244771	0,245732
ID26959	CTNA1		0,262092	0,266840	0,272124	0,286441	0,281429	0,283669	0,240012	0,236131	0,233130	-3,787972	0,233155	0,245955
ID377			0,265790	0,273005	0,273750	0,285077	0,287246	0,287848	0,234875	0,238372	0,218699	-3,787262	0,241310	0,234475
ID1443	PNPO		0,300777	0,414699	0,349929	0,482065	0,381834	0,402358	-0,348154	-0,107122	0,014522	-3,755445	-0,035048	0,021863
ID2288948			0,300081	0,298500	0,307637	0,255486	0,274882	0,268417	0,302110	0,249361	0,298584	-3,557192	0,234247	-1,712987
ID1146	RFC2		0,168273	0,174705	0,177609	0,171225	0,169122	0,175166	0,137355	0,123428	0,140251	-3,460720	0,151030	0,142196
ID1032	PA2G4	SAHH, ILEU	0,172181	0,174101	0,174056	0,182649	0,181433	0,177317	0,120523	0,118398	0,133602	-3,458275	0,142693	0,152186
ID2275539			0,160206	0,165738	0,167740	0,186188	0,175445	0,177005	0,126210	0,125128	0,147705	-3,447567	0,154146	0,138273
ID934	SYDC	HNRH1, PTN1, BLMH	0,159201	0,167763	0,164251	0,166117	0,164437	0,171902	0,141399	0,134832	0,135755	-3,359335	0,133240	0,140770
ID1692			0,150451	0,154532	0,152227	0,167587	0,167541	0,164071	0,131420	0,129508	0,138181	-3,310519	0,147698	0,152043
ID894	AL1B1		0,143955	0,149752	0,150494	0,168910	0,162412	0,163592	0,132772	0,122413	0,138274	-3,287451	0,153314	0,157839
ID1087	GALK1	ILEU, DNJBB, ROAA	0,148970	0,155982	0,153691	0,148456	0,145207	0,148211	0,152191	0,150074	0,154391	-3,269274	0,140168	0,137295
ID1352	ECH1	PSDE	0,148522	0,151510	0,153397	0,142229	0,152181	0,161475	0,153190	0,150384	0,155323	-3,238619	0,131267	0,119831
ID1067	ACTG		0,286858	0,292436	-0,200308	-0,275295	1,406065	-0,429870	0,181735	0,215451	0,235270	-3,222783	0,092247	0,612497
ID488759	CALD1		0,147086	0,151249	0,149815	0,170436	0,174098	0,164829	0,121944	0,115058	0,133605	-3,153470	0,120066	0,128551
ID2212082	CHP1		-0,041872	0,381423	-0,028152	0,967046	0,845228	1,042474	0,347794	-0,257843	-0,056175	-3,144641	-0,099648	-0,741794
ID509563			0,233083	0,201689	0,202743	0,244917	0,213777	0,200246	0,207277	0,245470	0,214148	-3,115284	0,175948	0,197164
ID1453	HSPB1	ECHM	0,225784	0,374684	0,281711	0,661885	0,515781	0,524655	-0,204401	-0,467389	0,027855	-3,097728	0,233747	0,148984
ID565	CSDE1		0,209127	0,220076	0,211649	0,243542	0,231150	0,220585	0,194738	0,191136	0,200158	-3,084546	0,198467	0,192781
ID501			0,221330	0,222759	0,227561	0,218514	0,224287	0,220086	0,200484	0,184953	0,201990	-3,071415	0,191492	0,190106
ID1367	RFA2		0,211203	0,213618	0,215427	0,207509	0,212467	0,215607	0,204400	0,203405	0,206804	-3,070063	0,207321	0,204786
ID479360	CAP2		0,204846	0,208202	0,213218	0,207107	0,209724	0,209922	0,212931	0,210692	0,218417	-3,066307	0,201015	0,203656
ID31086			0,211708	0,217605	0,214873	0,226824	0,217679	0,209392	0,191833	0,195492	0,200996	-3,060818	0,194671	0,214540
ID31063	MYH13	SEPT15, ACTY, OAT	0,212329	0,216842	0,217102	0,222654	0,225403	0,226243	0,184763	0,190314	0,194428	-3,057112	0,198906	0,203851
ID1166	GIPC1		0,209134	0,215558	0,214771	0,220221	0,214827	0,214909	0,194363	0,190040	0,197994	-3,056763	0,208043	0,212712
ID1492	HSPB1	PIMT	0,211020	0,215605	0,213573	0,217801	0,216747	0,220908	0,192552	0,190592	0,195315	-3,046869	0,204327	0,206712
ID1590			0,171422	0,192952	0,754918	-0,188780	0,002780	-0,451483	0,482346	0,609961	0,611916	-2,949516	-0,377449	0,403555
ID538			0,030362	0,390289	0,167577	0,634783	1,044813	1,343076	-0,186696	-0,390119	-0,164527	-2,938031	-0,503045	-0,162990
ID35204			0,012995	0,222827	0,023963	0,594949	0,485818	0,519876	-0,175065	-0,093974	0,143533	-2,927669	0,050221	0,410610
ID1763762			0,341870	0,353708	0,333715	0,363343	-0,421746	0,346248	0,322216	0,344701	0,332940	-2,831155	-0,444745	0,331672
ID922	RHG01	HNRH2	0,344021	0,465519	0,512201	0,804599	0,641063	0,723426	-0,293212	-0,519295	-0,372753	-2,768734	-0,193107	-0,035910
ID307			0,361878	0,362486	0,356196	0,387941	0,391525	0,372202	0,378299	0,345897	-1,672952	-2,686385	0,350575	0,321665
ID452			0,376241	0,465829	0,384934	0,681779	0,690266	0,729884	-0,509328	-0,463994	-0,574401	-2,670374	0,065274	0,156297
ID453			0,357882	0,373962	0,306993	0,624969	0,575984	0,562943	-0,177490	-0,101533	-0,746814	-2,656084	0,090118	0,125048
ID1809464			0,188120	0,238160	0,242700	0,301477	0,255369	0,315323	#DIV/0!	0,174098	#DIV/0!	-2,627440	0,204863	0,259875
ID1608			-0,218537	0,812453	0,538652	0,684495	0,458220	0,786404	0,461292	-0,744698	-0,519910	-2,624954	-0,117197	-0,172457
ID1272	PP1A		-0,240157	0,325276	-0,646098	1,538982	0,542824	0,844618	-0,350758	-0,119882	0,190439	-2,609066	-0,133734	0,005289
ID586647	DC1L2	TCPB	0,121632	0,205744	0,174018	0,623902	0,579525	0,449215	-0,083601	-0,117864	-0,095535	-2,573170	0,022108	0,050734
ID523641			0,375580	0,350349	0,451704	0,064100	-0,004422	0,039146	0,097871	0,171311	0,209525	-2,554334	0,196562	-0,035977
ID1707			-0,550318	-0,448473	-0,186534	1,002700	0,959150	1,571801	-0,358237	-0,379261	-0,383711	-2,506478	0,537878	0,114863
ID686	HSP7C		-0,077704	0,048720	-0,728300	0,447910	2,104866	-0,010912	0,386658	-0,554945	-0,414890	-2,459952	0,089274	0,554286
ID3763800			0,027539	0,073285	0,125294	0,458828	1,097515	1,006439	0,014702	0,105884	0,137503	-2,449652	-0,092020	-1,117730
ID765	LMNA		-0,819569	-0,605425	-0,467034	0,868381	0,641257	1,570935	0,726393	0,436459	0,586981	-2,384491	-0,606680	-0,543331

Table S-4: cluster 4

ID[1]	Protein[2]		co_0 min	co_30 min	co_60 min	co_24 h	co_48 h	co_72 h	Pl_0 min	Pl_30 min	Pl_60 min	Pl_24 h	Pl_48 h	Pl_72 h
ID1270	TALDO		-0,345028	-0,100219	-0,260001	0,908604	0,502811	0,657758	-0,042447	-0,209289	-0,230031	-2,312391	0,507725	0,344409
ID433	VINC		0,165767	0,172968	0,169040	0,176089	0,180284	0,172318	0,136912	0,133050	0,134557	-2,309766	0,143156	0,148185
ID1093	SCMC1	ILEU	0,167039	0,173106	0,173533	0,180272	0,181783	0,180951	0,118729	0,114756	0,135091	-2,304787	0,152264	0,151065
ID33115			0,166096	0,171981	0,177781	0,165395	0,169530	0,154971	0,158594	0,136277	0,168571	-2,302476	0,136349	0,121311
ID2070460			0,165610	0,174162	0,164350	0,209481	0,197373	0,268579	0,154300	0,099033	0,113355	-2,281235	0,081725	0,082958
ID1473	ECHM		0,067829	0,282142	0,213130	0,467229	0,373995	0,506890	-0,457776	-0,372613	-0,192360	-2,275627	0,490077	0,328176
ID1461			-0,307825	-0,046527	-0,154253	0,698756	0,402395	1,346790	-0,499379	-0,145799	-0,062674	-2,261542	0,385607	0,079066
ID1510	PRDX3		0,169554	0,178383	0,177473	0,178998	0,180057	0,183245	0,114432	0,108668	0,119308	-2,260810	0,139820	0,145669
ID1572	SFRS3		0,159366	0,168879	0,158305	0,179482	0,168547	0,168835	0,169480	0,151937	0,149346	-2,243535	0,114943	0,093533
ID36784			0,178545	0,185609	0,179715	0,199118	0,196333	0,192050	0,117341	0,092065	0,025255	-2,220036	0,148069	0,150926
ID1041	MP2K2		0,156541	0,162633	0,162732	0,161399	0,164678	0,163278	0,136391	0,133474	0,142798	-2,214973	0,137338	0,139968
ID771	TCPG		0,146235	0,151799	0,149115	0,162524	0,159677	0,163274	0,140923	0,143923	0,143097	-2,213837	0,147474	0,152338
ID778	DPYL2	LKHA4	0,147841	0,157606	0,150182	0,174405	0,166988	0,162779	0,134497	0,130366	0,134726	-2,212907	0,148173	0,152117
ID490428	LMNA	AGM1	0,131799	0,133084	0,133478	0,165271	0,164103	0,156847	0,159755	0,151795	0,151648	-2,211412	0,153992	0,156788
ID590			0,152904	0,158616	0,154656	0,177596	0,185025	0,182477	0,129453	0,110648	0,137116	-2,210876	0,135431	0,134235
ID837	TRXR1		0,150503	0,159792	0,157445	0,157237	0,148002	0,143375	0,138063	0,157479	0,147636	-2,210708	0,148221	0,150278
ID463195			0,149798	0,156204	0,151602	0,172742	0,189129	0,183036	0,130831	0,123469	0,127403	-2,210398	0,134363	0,139224
ID1139	ILEU	SCMC1, ROAA	0,154214	0,159062	0,161119	0,161668	0,156818	0,169155	0,148766	0,129464	0,131959	-2,207671	0,141663	0,141865
ID1338			0,158681	0,124456	0,157170	0,097586	0,077888	0,088415	0,200515	0,205878	0,202310	-2,206345	0,185878	0,155982
ID558	P3H3		0,144871	0,154578	0,145871	0,159421	0,166397	0,171754	0,146743	0,140062	0,143038	-2,199221	0,135928	0,140752
ID473357			0,150813	0,155655	0,158044	0,152221	0,142054	0,142033	0,132427	0,150613	0,146303	-2,198383	0,158544	0,160080
ID893	DD19A	TBG1, PPP5	0,154233	0,157651	0,160915	0,165583	0,160636	0,157919	0,133425	0,131356	0,137283	-2,192707	0,139439	0,146091
ID1652			0,141833	0,144428	0,145057	0,176988	0,165600	0,159061	0,130387	0,127166	0,134880	-2,186194	0,154878	0,159368
ID1408	GSTO1	ERP29	0,147006	0,154295	0,150359	0,155764	0,156414	0,158765	0,146772	0,144187	0,153906	-2,185431	0,138297	0,133308
ID1031	VAT1		0,158489	0,160548	0,165293	0,164489	0,158318	0,153979	0,143418	0,130341	0,136875	-2,184446	0,131106	0,135479
ID2995227	SFRS1		-0,033852	0,196700	0,150364	0,655706	1,768863	0,066541	-0,042691	-0,235629	-0,108085	-2,179931	0,286067	-1,069037
ID471051	HNRH1	TIF1B	0,155677	0,162170	0,157949	0,164456	0,156489	0,158531	0,143848	0,138029	0,137015	-2,178135	0,127748	0,131687
ID1514			0,152930	0,154956	0,158655	0,121136	0,139693	0,142228	0,147452	0,154528	0,157830	-2,173219	0,158709	0,141800
ID902	DD19A	MPPA	0,148796	0,155888	0,152385	0,169953	0,166270	0,166169	0,137780	0,134687	0,140667	-2,171033	0,126070	0,129610
ID849	TRXR1		0,153336	0,155094	0,160664	0,157951	0,153116	0,149580	0,133186	0,132547	0,141828	-2,166789	0,141499	0,146291
ID464690			0,141681	0,144068	0,141137	0,179866	0,178929	0,191896	0,118803	0,110978	0,115855	-2,162229	0,143489	0,154970
ID2194613			0,178966	0,191355	0,186027	0,197583	0,187129	0,168516	0,138583	0,110422	0,141232	-2,160216	0,047507	0,072843
ID1116	UBCP1		0,152858	0,157671	0,158844	0,164309	0,164564	0,163183	0,118405	0,116076	0,128342	-2,158615	0,148502	0,146208
ID1137	GALK1	ILEU, DNJBB, ROAA	0,142932	0,151804	0,146330	0,160381	0,157801	0,166252	0,153424	0,141035	0,136488	-2,152450	0,126731	0,131160
ID1535			0,150673	0,160283	0,151650	0,175357	0,177280	0,177826	0,100309	0,109939	0,126799	-2,148224	0,143501	0,137550
ID1064	ACTG	ACTB	0,157505	-0,136266	-0,742481	-0,666455	2,023743	-0,885419	0,429750	0,475315	0,500597	-2,139658	-0,101648	0,550103
ID467907	AGM1		0,134997	0,139436	0,137649	0,160161	0,138815	0,166667	0,138960	0,146809	0,148201	-2,129076	0,142984	0,142128
ID1776	OAT		0,156567	0,156309	0,155230	0,163395	0,151093	0,127411	0,136849	0,134898	0,137910	-2,125901	0,135973	0,138790
ID639			0,136729	0,144512	0,138403	0,173785	0,161737	0,159869	0,124358	0,124979	0,137548	-2,108918	0,131987	0,147781
ID703	SCFD1		0,139233	0,135640	0,141126	0,134113	0,128062	0,127198	0,154896	0,157759	0,153003	-2,087063	0,153694	0,140572
ID644	DPYL2	ZYX, EZRI, CALD1	0,133034	0,138274	0,140323	0,169595	0,157657	0,164485	0,120039	0,114562	0,121238	-2,077052	0,143742	0,154839
ID3591281	IF1AX	SORCN	-0,173512	-0,019157	-0,032575	0,519033	0,866487	1,034157	-0,428172	-0,373853	-0,323027	-2,072082	0,636225	-0,151545
ID1463	ERP29	TPIS, ETHE1	0,235740	0,325089	0,277091	0,350104	0,319626	0,272520	-0,235726	-0,105145	-0,050116	-2,048703	0,090953	0,056390
ID1464			-0,054236	-0,132419	0,560570	0,603609	-0,164862	-0,447045	-0,561921	0,822891	0,120662	-2,012302	-0,312943	1,074920
ID1980208			0,672384	0,016361	0,193388	0,007142	-0,121183	0,397717	0,596611	0,335360	0,420753	-1,966710	0,196256	-1,239756
ID1800806			0,337677	0,337213	0,338296	0,348214	0,356852	0,335988	-0,536905	0,371892	0,335190	-1,947469	-0,860518	0,244290
ID152055	CATD	CLIC1	-0,149694	0,396069	0,389972	-1,424322	1,144783	1,957356	-0,424791	-0,269706	0,368915	-1,934149	-0,214171	-0,323800
ID1723	CT077		-0,654957	-0,273663	-0,492622	0,996593	1,164817	0,916005	-0,334328	-0,549268	-0,041988	-1,924734	0,399962	0,312999
ID2379			0,191607	0,493203	0,512445	0,422136	0,577087	-0,293026	-0,819892	-0,786549	-0,806266	-1,812110	0,708497	1,159840
ID2340727			0,190053	-0,165039	0,588655	0,376050	-0,786456	-0,489159	-0,277704	1,158603	0,702733	-1,698201	-0,029477	0,005391
ID553	HS90A		1,093562	0,840329	-0,078226	-0,212631	0,633924	-0,599567	0,002018	-1,273287	-0,256328	-1,065097	0,461532	0,187496
ID258601			-0,424205	-0,087470	-0,899105	0,745106	-0,128866	1,414409	-0,093066	0,663888	0,597729	-0,989063	-1,317539	0,270916
ID4106	HS90B	HS90A	0,061879	-0,133240	-0,698407	-0,735994	0,919539	-0,736954	0,644641	0,021036	0,439163	-0,881173	0,210335	0,668881
ID3270147	CBX5	CATB	-0,060143	0,657171	-0,021796	-1,536594	-0,931686	1,051922	0,592973	-0,458557	-0,511358	-0,661423	1,323830	0,390306

Table S-4: cluster 5

Cluster 5 ID)1	Protein)2		Z-score)3 co_0 min	co_30 min	co_60 min	co_24 h	co_48 h	co_72 h	PI_0 min	PI_30 min	PI_60 min	PI_24 h	PI_48 h	PI_72 h
ID146603			0,234821	0,268989	0,107065	0,646779	0,592468	0,391407	-0,310274	-2,842435	-0,520993	0,736283	0,423044	0,456917
ID1804168			0,221427	0,231039	0,226198	0,218491	0,196811	0,207457	0,365080	-2,756645	#DIV/0!	0,241235	0,234331	0,259534
ID90190	SET		0,286358	0,062995	0,493796	0,608336	0,787264	0,947516	-0,843133	-2,485886	-0,901856	-0,067513	0,604970	0,490273
ID1677			0,205423	0,213447	0,195210	0,213462	0,199660	0,205670	0,185467	-2,311546	0,166299	0,279186	0,260052	0,257467
ID48368	BASP		0,228438	0,251258	0,224974	0,245880	0,247898	0,258269	0,156938	-2,296510	0,114525	0,197075	0,214722	0,205802
ID233300			-0,376617	0,005956	-0,521989	1,156967	0,883969	0,755430	-0,400017	-2,219903	-0,945878	0,713701	0,531806	0,595001
ID1404	SPEE	CATD	-0,821359	-0,612491	-0,229764	0,476610	1,085822	0,804603	-1,018401	-2,144533	0,131547	1,263666	0,362512	1,017704
ID386066			-0,325321	-0,057291	0,242484	1,243759	0,757064	1,227065	-0,617041	-2,111281	-1,178452	1,161323	-0,057603	0,005626
ID2549343			0,088696	0,054446	0,634300	0,162245	0,862299	0,821375	-0,519168	-2,094292	-0,419587	-0,691752	0,385302	0,543197
ID10923	HS90A		0,337621	0,526198	0,000329	1,440148	1,073386	0,820524	-0,845123	-2,047555	-1,399528	0,229517	-0,248418	0,170281
ID2742902			-0,341142	-0,076760	0,275580	0,417644	0,179285	0,902153	-1,077296	-2,046174	-0,818402	1,703897	0,736719	0,570471
ID1142	SET		0,121232	0,189693	0,387370	1,218260	0,768001	1,397259	-0,988551	-1,997570	-1,374388	-0,146719	0,257973	0,130760
ID3841	HS90A		-0,548004	-0,494635	0,225453	0,770283	0,749122	1,250953	-0,894346	-1,977226	-1,121747	1,027300	0,726195	0,543478
ID1173	FSTL1	ANXA2	-0,732285	-0,049285	-1,187828	1,246477	1,047304	1,134899	-0,065332	-1,975314	0,596668	0,986044	-0,401880	-0,352957
ID89291			-0,129232	0,294976	-0,417862	1,441904	0,736940	0,864932	0,227567	-1,971541	-0,663209	0,076208	0,029150	-0,470783
ID389942			0,093299	0,466944	-0,530919	0,854359	0,786219	0,817039	-0,702473	-1,961687	-1,346641	0,785615	0,296984	0,637665
ID1190			-0,060223	-0,034196	0,052607	1,253151	0,425041	0,002986	0,716603	-1,959125	-1,550996	0,385365	0,265626	0,599503
ID57056			-0,238142	0,283824	-0,171740	1,301278	1,067062	0,742250	-1,186458	-1,958084	-0,757281	0,413008	0,137516	0,470018
ID546	EF2		-0,320868	-0,144583	-0,029512	0,937514	1,060274	1,575375	-0,795362	-1,956123	-1,290022	0,005957	0,434557	0,524283
ID1547	CBX1		-0,432417	-0,194371	-0,205180	1,244852	1,029219	1,023474	-0,676446	-1,945718	-1,317593	0,364310	0,488482	0,712465
ID1328	PCNA	CX026, AN32E	-0,281109	-0,229518	-0,258030	0,759023	1,296887	1,424970	-1,120369	-1,942786	-0,916543	0,667627	0,633655	0,133100
ID5273			-0,082460	-0,098595	-0,066306	1,228325	0,792906	1,423245	-0,936174	-1,916970	-1,276222	-0,060813	0,436824	0,541035
ID790	HNRPK		-0,160878	0,680650	0,301753	1,672874	0,757675	0,026408	-0,318402	-1,912673	-1,586356	1,019081	0,117165	-0,342527
ID1550			-0,432417	0,078052	-0,250593	1,185587	0,822918	1,251179	-1,177127	-1,890927	-0,780239	0,107881	0,428770	0,683886
ID3313934	PSME2	1433E	0,154853	0,001490	0,381210	0,287121	0,236800	0,385152	-1,298462	-1,889138	-1,203947	1,981372	0,711487	0,747405
ID1009			-0,315352	0,003195	0,106809	1,392257	1,214958	1,301720	-0,654448	-1,866186	-0,554967	-0,036691	-0,072320	-0,528148
ID495			0,045378	0,303564	0,241911	1,113442	0,830859	1,173354	-1,255109	-1,849110	-1,513991	-0,164210	0,409635	0,623224
ID1780	TBCA		0,189177	0,744558	0,168435	1,041233	0,880095	0,917352	-1,832841	-1,846870	-0,982127	0,370354	0,164659	0,278563
ID3255127	ACTG		0,583582	0,331105	0,366239	1,071951	-0,419648	0,379185	-0,294368	-1,841335	-1,135333	1,498984	-0,612290	0,446674
ID847	TCPE	HNRPK	-0,028377	0,133484	0,160857	1,262144	1,036981	1,753760	-0,749663	-1,815392	-1,218112	-0,283625	-0,250823	-0,072141
ID983	DNJA2	APMAP, SNX6	-0,162963	-0,025076	-0,135095	1,025175	0,896893	0,882880	-0,290798	-1,807260	-0,538575	0,072739	0,045234	0,055031
ID804	TCPE		-0,489265	-0,047403	-0,175007	1,244160	1,063179	1,399943	-0,760200	-1,801370	-1,476300	0,416214	0,307821	0,422280
ID1364	NUDT5	ANXA5, SFRS2	0,007503	0,453868	0,031979	1,194550	1,244790	1,141484	-1,550911	-1,800189	-1,048472	0,311639	-0,039368	0,131036
ID1411			-0,477461	-0,305698	-0,031210	0,526372	1,352811	1,812887	-1,110710	-1,797460	-0,904743	0,423255	0,327404	0,290366
ID31044			-0,527256	-0,157067	-0,448401	1,118190	0,759859	1,095636	-0,593998	-1,796497	-0,855461	-0,429063	0,876764	0,742760
ID3464756			0,284883	0,287810	0,286953	0,276118	0,278591	0,257404	0,243749	-1,785782	-0,753346	0,233855	0,243687	0,268894
ID14100	CH60		-0,435057	0,437880	0,096595	1,464714	0,483329	1,016629	-1,216399	-1,783851	-0,973335	0,772633	0,270968	0,059052
ID1695	STMN1		-0,340860	-0,049459	-0,321945	1,068701	1,220309	1,892692	-1,124322	-1,782436	-0,618390	-0,328425	0,233214	0,068813
ID1577			-0,585548	-0,376512	-0,613093	1,412489	0,408675	0,726023	-0,080495	-1,775502	-0,898185	1,372143	0,453351	0,299691
ID2294			-0,183453	0,576763	-0,596104	1,550594	1,018411	0,811238	-0,799823	-1,767338	-0,784673	-0,327465	-0,010088	0,430072
ID82135			-0,253101	0,108193	-0,030749	1,091440	1,263611	0,550191	-1,522363	-1,763384	-1,150439	0,308337	0,611630	0,863719
ID2784333			0,162585	0,357474	-0,101819	1,002523	0,762692	0,488029	-1,009008	-1,755042	-0,969404	1,805742	0,007252	-0,299589
ID30752	SODC		0,225632	0,221576	0,170584	1,508264	1,183380	1,080033	-1,604520	-1,719448	-0,934368	0,168705	-0,121612	-0,136049
ID542	TERA		0,002774	0,397836	0,055618	1,167861	1,233293	1,485634	-1,010032	-1,714049	-1,294609	-0,793073	0,088427	0,182052
ID617	IF4B	NEBU	0,309642	0,491616	0,026831	1,522175	1,083524	0,818745	-1,306902	-1,698233	-1,445552	0,193271	-0,156854	0,210057
ID1651	SODC		0,052509	0,503965	-0,071159	1,021912	0,705180	1,888416	-1,621685	-1,697765	-0,724076	-0,222924	-0,039822	0,149717
ID66993			-0,643712	-0,236866	-0,369721	1,210436	0,871797	0,787768	-1,290562	-1,690272	-0,922137	0,755787	0,860765	0,855664
ID1476	PSA5		0,103303	0,290209	0,245061	0,937660	1,231339	1,258956	-1,215144	-1,682566	-1,652119	0,072487	-0,018925	0,447861
ID209100	ACTG	ACTB	0,188636	0,178987	0,194551	0,175588	0,166909	0,176839	0,101995	-1,679778	0,078610	0,163994	0,147871	0,146795
ID4489			-0,095226	0,055977	-0,028082	0,998807	1,078462	1,327671	-1,477180	-1,676828	-1,317043	0,455866	0,270619	0,520925
ID2575			-0,666368	0,231312	0,094012	1,180595	1,329909	0,905765	-1,338585	-1,668038	-0,751825	0,736304	0,364281	-0,233286
ID68693			-0,295461	0,140688	0,066162	1,316962	1,422552	-0,027421	-1,054269	-1,648643	-0,922960	0,977029	-0,045957	0,315575
ID3104	TBB2C	TBB6, TBB2A, SMCE1	0,096564	0,172672	0,201782	1,251778	1,035154	1,296323	-1,683558	-1,629089	-1,152021	-0,211023	0,268325	0,300337
ID315424			0,096950	-0,299960	-0,062776	1,014181	1,443992	1,385974	-0,561891	-1,623125	-0,908653	-0,632581	0,043324	-0,053580
ID59353	EF1B		-0,727680	-0,420469	-0,639449	1,367733	1,213612	1,011310	-1,075847	-1,620896	-0,813299	0,578198	0,522043	0,749294
ID261244			0,155084	0,176020	0,156300	0,160909	0,217931	0,182228	0,113414	-1,618739	0,101676	0,122617	0,128042	0,135174
ID405373	TCPE	FKBP4, CPNE1	-0,030104	0,611583	0,358398	1,121949	0,716803	1,480800	-1,250899	-1,614606	-1,529357	-0,190733	-0,014233	0,292715

Table S-4: cluster 5

ID)[1]	Protein)[2]		co_0 min	co_30 min	co_60 min	co_24 h	co_48 h	co_72 h	PI_0 min	PI_30 min	PI_60 min	PI_24 h	PI_48 h	PI_72 h
ID1224	NPM		-0,033842	0,819917	-0,256164	1,453914	0,213905	-1,022810	-0,464310	-1,611248	-0,682116	0,015895	0,802313	0,768522
ID1299			0,063840	0,000598	0,125135	0,336693	0,372565	0,252482	0,073600	-1,609078	0,033768	0,100961	0,217328	0,057348
ID1414			-0,208350	-0,023100	0,413490	0,174010	1,123051	1,997994	-1,283189	-1,605846	-0,072397	0,898549	-0,815024	-0,374552
ID1226			0,103602	0,758176	0,156749	1,642823	0,459261	0,484637	-1,236143	-1,605041	-1,288129	1,104823	-0,163740	-0,140812
ID2140633			0,178394	0,169342	0,179592	0,200245	0,199740	0,197819	0,138370	-1,572773	0,158447	0,023156	0,147201	-0,013744
ID1736			-0,436570	0,292141	-0,018453	0,935627	0,913168	0,798689	-1,255118	-1,564424	-1,393775	0,225237	0,508111	1,051677
ID245554			-0,006840	0,477190	0,048910	1,514023	0,226973	-0,023627	-1,341669	-1,563998	-0,725302	-0,149033	0,933543	0,742802
ID1143	SPB6		0,161642	0,298308	0,283125	0,720388	0,919611	1,256077	-1,268449	-1,557694	-1,247228	1,649322	-0,567159	-0,235613
ID1136			0,214275	0,219776	0,786242	0,714830	0,825113	1,146755	-1,985196	-1,554267	-1,130984	0,083303	0,196686	0,504294
ID16864	PRDX4		-0,894419	-0,491936	-0,627797	1,338592	0,638544	1,159552	-0,782022	-1,549229	-0,575016	1,094284	0,399879	0,563137
ID566208			-0,176858	-0,121828	-0,053698	1,271606	1,268065	1,294508	-0,740220	-1,548908	-1,227064	-0,581761	0,299439	0,171279
ID1521			0,191647	-0,030312	-0,043920	0,993486	0,946305	0,725360	-1,675673	-1,530557	-0,862231	0,482974	0,488908	0,434757
ID60178			-0,868703	-0,441384	-0,974499	1,679392	0,795000	1,165161	-0,626088	-1,515784	-0,871061	0,798924	0,369527	0,689247
ID1621			0,183868	0,385360	0,152254	1,449231	0,948792	0,722802	-1,472603	-1,512573	-1,022592	1,073333	-0,620733	-0,018806
ID87491	TPM2		0,164378	-0,301413	0,295089	1,258656	1,035996	0,409668	-1,568214	-1,511649	-0,833805	0,901643	-0,114348	0,489411
ID652			-0,561791	-0,393246	-0,663021	1,357138	0,561937	0,850378	-0,949710	-1,510051	-0,881949	1,559658	0,058718	0,961855
ID2314456			0,338987	0,375757	0,177239	0,035057	0,555902	0,355247	-0,424558	-1,504643	-1,920860	0,975574	0,526515	0,753677
ID2977290			0,332298	0,347422	0,307319	0,336573	0,313572	0,318571	-1,501389	-1,497905	0,263650	0,293488	0,284272	0,275501
ID821	UBP14	IMA2	0,107756	0,180917	0,419041	1,077479	1,003179	0,971836	-1,768078	-1,495118	-1,201319	0,173630	0,225213	0,348871
ID1394			-0,244810	-0,076016	-0,015145	0,637479	0,698661	0,988253	-1,835278	-1,493162	-1,028807	1,703174	0,513571	0,577874
ID1540			-0,650211	-0,501525	-0,591076	1,067165	0,630893	0,878676	-0,269273	-1,489866	-1,590833	0,530699	1,109473	1,008552
ID913	K2C8	PSMD5	-0,783269	-0,843086	-0,517513	1,933865	0,309299	-0,317325	-0,151845	-1,488629	-0,288927	0,254103	0,989693	0,967159
ID362785			-0,203823	0,035513	0,310195	1,362446	0,709903	0,892657	-1,068942	-1,478516	-1,357394	0,839983	0,043836	0,124138
ID1452			-0,536625	0,034195	0,733182	1,138853	0,937636	1,338100	-1,524986	-1,477434	-0,815399	0,258334	-0,099339	0,078068
ID1716			-0,700365	-0,050289	-0,367670	0,919523	1,081172	1,438100	-0,742795	-1,477112	-0,845269	-0,730936	0,069332	1,223576
ID91144			-0,091425	0,141315	-0,125835	0,966835	0,974119	1,867086	-1,352373	-1,474513	-1,152909	-0,130821	0,064092	0,281724
ID680	HSP7C	HSP71, LMNB2	0,226560	0,314302	-0,064546	1,428395	1,064156	1,051889	-0,408527	-1,469283	-1,811522	-0,448067	0,161686	-0,157059
ID145564			0,225162	-0,131594	0,442521	1,500602	0,723535	1,257961	-0,754271	-1,460209	-1,771428	-0,099748	-0,196906	0,239437
ID182136			-0,781459	0,498260	0,720229	0,949957	1,023838	0,807065	-0,444542	-1,451260	-0,932685	-0,358058	-0,435781	0,314922
ID1562			-0,168603	0,317794	0,170916	0,731164	0,628423	1,633882	-1,110987	-1,450127	-1,242496	0,995139	0,146078	-0,402398
ID1200	NPM		0,250826	0,528463	0,232507	1,189299	0,995580	-0,136030	-0,328187	-1,448349	-2,126473	0,346045	0,061796	0,521036
ID317853			-0,155643	-0,559721	-0,371352	0,466939	1,298598	1,903704	-0,527558	-1,442357	-0,754012	0,021454	0,018260	0,107051
ID2437			-1,112307	-0,709250	-0,610472	1,039943	1,050664	1,103711	-0,280800	-1,441800	0,609878	-0,179155	0,229792	0,255008
ID124470			-0,116889	-0,034767	-0,509105	0,850512	1,053140	1,979935	-1,192508	-1,426660	-0,861744	0,013044	0,269160	-0,020857
ID1602			-0,587438	0,359590	-0,397286	1,248728	0,765109	1,189944	-0,798660	-1,416431	-0,853087	-1,038128	0,890733	0,377396
ID682	VINC		-0,205984	-0,021652	-0,340153	1,035126	0,941935	1,259964	-1,677386	-1,393740	-1,278071	0,599504	0,382690	0,847642
ID1259	DDAH1	CAZA1, LDHB, RLA0	0,083361	0,185413	0,468854	0,664836	0,582363	0,920245	-1,324614	-1,381815	-1,672279	0,977024	0,119174	0,621694
ID1656	IF1AX		-0,129960	0,100224	-0,300773	1,726003	1,201217	0,906173	-1,424956	-1,381197	-1,009605	0,256484	0,174374	-0,053863
ID216132			0,137298	0,112546	0,793824	0,021242	0,619426	1,412785	-1,600393	-1,380877	-1,027847	-0,883377	0,715478	0,859052
ID1131	TMOD3		-0,403775	-0,009528	-0,593617	1,599067	1,440639	1,461233	-0,834293	-1,369602	-1,111698	0,405361	-0,254009	-0,228438
ID186642			-0,284226	-0,143858	-0,571425	0,417528	0,646494	1,984111	-0,370392	-1,343534	-0,971137	0,767026	-0,058433	0,119602
ID1395			-0,481919	-0,294551	-0,081004	1,065872	1,331276	1,088707	-1,669431	-1,339533	-1,193495	0,499843	0,688794	0,510402
ID341707	APT		0,090506	0,080545	0,198735	1,495642	0,623222	0,541736	-1,921274	-1,307869	-1,013437	0,304053	0,475982	0,508174
ID636			-0,384570	-0,033422	-0,339436	1,853031	1,011751	1,420239	-1,117131	-1,304895	-0,584599	0,571571	-0,837907	-0,111739
ID567			0,096506	0,503444	0,034729	1,130088	0,553816	1,248211	-2,114218	-1,302635	-0,782772	0,600797	-0,353478	0,535712
ID65305			0,034381	0,237841	-0,163505	0,965480	1,156395	1,350587	-1,477784	-1,286767	-1,029257	-1,007944	0,305573	0,663013
ID1129			0,114874	0,410716	0,009403	1,584044	0,812104	1,343440	-1,451072	-1,285953	-1,234079	0,022157	-0,209000	-0,111096
ID1181			0,136023	-0,036033	0,397136	1,597692	0,961530	1,225308	-1,668360	-1,278947	-0,943035	-0,259716	-0,488560	0,292034
ID1666			-0,756285	-0,685634	-0,902441	1,093813	1,092681	1,704729	-1,121059	-1,278832	-0,636140	0,575302	0,543498	0,514192
ID1047	ADRM1	ACTG, TMOD3, ATPB	-0,211057	0,211657	0,140805	1,637557	1,259509	0,854971	-0,834743	-1,251990	-1,636340	0,224351	-0,251316	-0,087316
ID303936			-0,223863	-0,536181	-0,107018	0,238073	0,522203	1,153796	-1,479573	-1,246776	-1,162225	1,507054	1,041600	0,669672
ID1557			-0,155729	0,561521	-0,246365	1,047230	0,323062	0,536374	0,210562	-1,244750	-0,828321	0,805505	-0,223807	-0,583905
ID16669	TXNL5		-0,102679	0,150387	-0,109480	0,114105	1,218550	0,512629	-1,472283	-1,240447	-0,834238	-0,688552	0,505197	1,774674
ID314			0,112488	0,356558	-0,140483	1,350699	1,181594	0,621375	-1,281422	-1,231269	-1,347016	1,523149	-0,333596	-0,431290
ID1931678			-0,573738	-0,426221	-0,308535	0,055644	0,941953	0,787053	-1,008859	-1,228360	-0,985711	0,080251	0,401401	2,285184
ID2055531			0,042722	0,282496	-0,243479	1,584533	0,600887	0,614759	-0,646571	-1,212930	-1,111002	1,185084	0,003198	-0,803426
ID1911469			0,024511	0,502803	-0,006542	0,312170	0,446067	-0,764080	-1,757712	-1,197760	-0,749686	0,964519	1,195783	1,271056
ID327583	DC1I2		-0,029918	0,588863	-0,104544	0,765229	1,245867	1,592474	-0,693369	-1,185897	-1,152825	0,837129	-0,725514	-0,928212
ID2329037			-0,118773	0,427572	-0,179414	0,574232	0,733365	2,323280	-1,019613	-1,183825	-0,804338	0,618010	-1,018267	-0,197725

Table S-4: cluster 5

ID)1	Protein)2		co_0 min	co_30 min	co_60 min	co_24 h	co_48 h	co_72 h	PI_0 min	PI_30 min	PI_60 min	PI_24 h	PI_48 h	PI_72 h
ID1655			-0,613441	-0,615289	-0,795470	0,792359	1,186782	0,463429	-1,137011	-1,179878	-0,996753	1,888281	0,941338	0,537722
ID154183			-0,234971	0,336209	0,102596	0,790295	1,023440	1,322782	-1,981698	-1,174021	-1,229992	-0,108378	0,616218	0,510426
ID623	HS90A		-0,473376	-0,199970	-0,340537	1,459740	1,239518	1,780192	-1,056516	-1,170314	-1,151149	-0,192573	0,054861	0,001980
ID204237	M6PBP	IF35, HNRPF	-0,632968	0,189420	0,270925	1,467488	1,008132	0,341305	-1,009719	-1,167393	-1,383365	1,821842	-0,448234	-0,001972
ID344430			-0,708225	-0,633359	-0,705527	0,465540	1,241819	0,961712	-1,412168	-1,162292	-0,505294	1,617286	0,534510	0,710321
ID57837			0,136661	0,160616	0,120706	0,577928	0,911501	1,513029	-1,348815	-1,139130	-1,081307	-0,866043	0,211709	0,586635
ID3763670			0,549104	-0,563309	-0,021626	0,616768	0,594249	0,617903	-0,561407	-1,136019	-1,688927	0,567353	0,582322	0,585426
ID2051821			-0,052741	0,197788	-0,293917	1,443855	1,057743	0,701090	-1,153751	-1,128024	-1,311818	1,593387	-0,081722	-0,573543
ID674			-0,505558	-0,367647	-0,260630	0,877956	0,510069	0,895645	-1,071473	-1,108124	-0,831324	2,448517	-0,156247	0,180945
ID20028	NEBU		0,059538	0,016780	-0,061665	1,524981	0,930093	0,715079	-1,184794	-1,096712	-1,510422	-0,038828	0,461395	0,174847
ID1820			-0,713827	-0,461932	-0,571040	0,937422	1,226008	-0,022442	-1,145449	-1,087054	-0,892714	0,328120	1,277566	1,207372
ID5884			0,185626	0,067062	0,129149	0,432422	0,743011	1,438901	-1,401977	-1,086338	-1,050064	-0,753500	0,331378	0,775954
ID440482			-0,551149	-0,306037	-0,263997	0,365856	0,453958	0,703507	-0,957853	-1,083874	-0,959384	-0,746997	1,560597	1,598626
ID870	TBA1B	VIME, TBB3	-0,368620	-0,396521	-0,191453	0,946113	1,040560	1,722525	-1,674419	-1,083517	-1,082661	0,779641	0,313733	0,189530
ID1053	ACTG	M6PBP	-0,405215	-0,695573	-0,479801	1,091488	1,295966	1,899800	-0,826184	-1,081086	-1,192939	0,219344	0,049022	0,180014
ID309937	GRAP1	GANAB	0,224287	0,232476	0,214901	0,255641	0,239642	0,238414	-1,038064	-1,075816	0,123315	0,206479	0,211682	0,218663
ID663	MEPD	THOP1	-0,075076	0,430906	-0,134892	1,437767	1,566366	1,154161	-1,415967	-1,074375	-1,281858	-0,558671	-0,140691	-0,047338
ID1998321			-0,528771	-0,451520	-0,625081	1,435269	1,195944	1,742662	-0,440451	-1,072336	-0,456071	-1,092382	0,070755	-0,051112
ID1821	THIO	RFA3, LEG1, COX5A	-0,239074	0,125063	-0,098817	-0,400469	0,664673	-0,165817	-1,512435	-1,065732	-0,897227	1,234760	0,592185	2,071580
ID1759			-0,033313	0,608729	0,347992	1,198956	0,598957	0,858668	-0,905736	-1,034028	-1,570201	0,240821	-0,290098	0,039459
ID1993029			-0,376246	-0,119949	-0,152603	0,261282	3,245711	0,972896	-0,535353	-1,028502	-0,636848	1,071859	#DIV/0!	#DIV/0!
ID1960958			-0,178754	-0,028375	-0,133445	0,884430	0,747345	-0,006064	-1,154435	-1,001040	-0,507103	1,254558	0,458024	-0,021503
ID1964508			-0,794245	-0,502357	-0,647224	0,728297	0,983546	-0,281556	-0,643555	-0,990297	-0,964140	0,265313	0,678861	2,233684
ID637	EZRI		-0,031799	0,132462	0,064577	1,592357	1,059741	0,275186	-1,578226	-0,988411	-0,826667	1,751660	-0,565824	-0,447141
ID3759858			-0,482852	-0,004423	-0,759488	0,945639	1,375913	1,205323	-0,727488	-0,984287	-0,612601	1,113163	-0,513998	-0,405110
ID470			-0,622960	-0,170415	-0,848823	0,839007	0,979150	1,683414	-1,069267	-0,982383	-1,019106	0,495130	0,549634	0,290400
ID3763000	CHM4B	RPB3	-0,636697	-0,256289	-1,029827	1,395953	1,798614	0,949104	-0,130808	-0,976958	-1,046921	0,174391	0,165435	-0,362400
ID426			0,052499	-0,279406	-0,997927	1,350838	0,444045	1,181143	-0,089925	-0,922483	-0,800835	-1,281172	0,765226	0,257704
ID485827			-0,222546	-0,060912	-0,766287	1,458899	0,962298	0,853154	-1,305300	-0,913444	-1,369354	0,524995	0,319228	0,650517
ID3763682			-0,384613	0,217988	-0,976627	0,636121	0,089553	0,952247	-0,553711	-0,896807	-1,572024	1,774165	0,615679	0,541570
ID341			-0,086614	0,020449	-0,225332	0,853415	0,442117	1,818745	-0,696321	-0,890178	-0,979698	0,402466	-0,249982	-0,308451
ID2846419	GRP78		-0,287099	-0,864439	-0,131072	0,609634	1,024727	1,471017	-0,170200	-0,884211	-1,360816	0,701555	0,148467	-0,082175
ID1045			0,061955	0,253869	0,041936	0,551957	0,283459	1,489943	-1,445545	-0,853179	-0,783877	-0,095546	0,529913	-0,058772
ID16655	PHP14		-0,115072	0,188273	-0,087215	-0,037931	1,292633	0,824696	-1,231228	-0,853073	-0,708120	-1,294454	0,119321	1,578556
ID1645	NDKA	BT3L4	-0,176925	0,314087	0,225099	0,922754	1,322614	1,626950	-1,225491	-0,835193	-1,729147	-0,230029	-0,153817	-0,118410
ID113114			0,069689	0,466280	-0,303724	1,094137	1,138036	1,298633	-1,939402	-0,832770	-1,276959	0,251074	-0,043945	0,141720
ID464	HNRPU	HNRH1	-0,358461	0,014134	-0,438501	0,853848	1,051281	1,366452	-1,183828	-0,818802	-1,459281	-0,390312	0,701462	0,564431
ID157378			-0,505487	0,005376	-0,783309	2,235782	1,279778	-0,164655	0,160614	-0,767892	-0,520819	0,185143	-0,584894	-0,493353
ID1630	MTND	SSRD	0,159428	0,625662	-0,217628	1,085942	0,898341	0,928440	-1,664182	-0,757019	-0,207066	-0,346270	-0,118744	-0,473471
ID2998	TBB2C	TFG	-1,115619	-0,943873	-0,894070	1,298743	0,700077	1,477991	-1,011027	-0,747814	-0,767309	1,180147	0,493219	0,624572
ID16938	PSB4		-0,663416	-0,467852	-0,718850	1,499183	0,736134	0,767489	-0,532291	-0,747376	-0,920044	1,465474	0,038264	-0,090345
ID13655	IF4A1	NDRG3, RBBP7, PDIA6	-1,225765	-0,708701	-0,707401	0,994118	1,150387	2,198200	-0,395096	-0,720578	0,050611	-0,350546	-0,346511	-0,026356
ID1619			-0,495690	-0,884505	0,379708	1,875551	0,766660	0,330497	-0,316402	-0,708898	-0,010101	-1,063306	-0,690199	0,550859
ID1197			-0,967000	-0,510951	-0,526847	1,828956	0,968129	1,666647	-0,403355	-0,699456	-0,965804	-0,923338	0,040251	0,261933
ID1074	ACTG		-1,158985	-1,038484	-0,530069	1,166100	1,210721	1,620671	-0,770388	-0,691274	-1,096488	0,673118	0,093525	0,689834
ID463			-0,298758	-0,123182	-0,446548	0,798287	0,408688	1,097463	-1,311579	-0,687618	-1,395798	0,062328	1,046208	0,866091
ID1996662			-0,555937	-0,162987	-0,257529	1,305267	#DIV/0!	2,168148	-0,616745	-0,666774	-0,919131	1,053015	#DIV/0!	#DIV/0!
ID1686	STMN1		-0,367223	0,544511	-0,464570	1,188114	0,621691	-0,480788	-2,027602	-0,657748	-0,413870	1,988515	0,238248	0,327850
ID2048120			-0,488726	-0,331640	-0,616788	0,135051	2,204571	0,857076	-0,518101	-0,656817	-0,425439	1,041922	-0,194277	-0,292348
ID58577			0,315508	0,319603	0,293264	0,337705	0,334662	0,326769	-2,395393	-0,649156	0,233265	0,315441	0,318622	0,328569
ID505	GRAP1	GANAB	-0,442542	-0,385091	-0,582228	1,334684	0,979065	1,993595	-0,812348	-0,634858	-1,148269	-0,275293	-0,302686	0,207146
ID3761869			-0,631513	-0,505670	-0,879076	1,446417	0,682802	1,004886	-0,515889	-0,633207	-0,348695	1,033540	#DIV/0!	0,410395
ID1991205			-0,345110	-0,497428	-0,684016	1,140946	0,148892	0,662846	-0,800371	-0,629790	-0,591902	-0,283715	#DIV/0!	2,411626
ID4808			-0,180833	-0,619308	-1,276922	1,848954	0,708733	0,821281	-1,102189	-0,618629	-0,206166	0,570357	0,396815	-0,199502
ID338842			-0,807313	-0,293137	-0,533709	1,355266	1,223549	0,632369	-0,470263	-0,595363	-1,069493	0,428290	1,069069	-0,832194
ID2315592			-0,971724	0,093437	-0,375904	2,016580	0,681456	0,293104	0,112812	-0,575521	-0,638748	-0,572287	-0,390322	0,184046
ID1693	TXD12	STMN1	-0,878681	0,556865	-0,773994	1,460419	0,797348	0,039947	-1,345459	-0,569373	0,182203	1,130180	-0,316521	-0,000389
ID196961			-0,408235	-0,416953	-0,348588	1,453684	1,121575	1,958495	-1,079680	-0,534253	-1,242020	-0,594108	-0,231288	0,172843
ID1954			-0,283429	-0,248924	-0,224955	-0,204704	#DIV/0!	2,237610	-0,650536	-0,508330	-0,484667	0,017609	#DIV/0!	0,472972

Table S-4: cluster 5

ID[1]	Protein[2]		co_0 min	co_30 min	co_60 min	co_24 h	co_48 h	co_72 h	PI_0 min	PI_30 min	PI_60 min	PI_24 h	PI_48 h	PI_72 h
ID425876			-0,470365	-0,007321	-1,084767	1,727698	0,622813	0,326883	-1,041472	-0,457944	-0,125974	-0,176268	0,336998	0,305651
ID907	PSMD5	TXND5	-0,918868	-0,974301	0,034454	1,022432	0,801782	2,154774	-0,088658	-0,380679	-0,570100	0,317105	-0,837889	-0,480773
ID1486			-0,245677	-0,103785	0,024636	0,469187	0,862954	-0,037087	-1,231085	-0,374641	-0,689384	-1,470662	1,184434	1,243444
ID500			-0,164010	-0,436651	-0,544148	1,118988	1,214300	1,866192	-1,320422	-0,332608	-1,049863	0,050328	-0,431739	0,042215
ID1048			-0,311490	0,321994	-0,793107	1,324491	-0,274283	1,254796	-0,907455	-0,320459	0,010654	-0,950876	0,292959	0,115057
ID2016883			-0,614902	0,375373	-0,969286	0,406373	0,310004	2,275466	-0,500912	-0,298166	-0,223067	-0,315262	0,184376	-0,708812
ID27793			-0,568556	-0,512300	-0,659649	1,253714	1,696520	1,083789	-1,150925	-0,296242	-0,967343	-1,167454	0,345343	0,651239
ID3763684			-0,297658	0,154697	-0,322911	0,535872	1,017503	2,288139	-1,511091	-0,193041	-0,995532	0,128376	-0,359139	-0,413122
ID438739			-0,536521	0,527806	-1,187986	0,702840	-0,032248	1,899150	-1,137490	-0,169684	-0,324661	-0,159916	0,158014	0,220718
ID3761658			0,381906	0,181682	-0,428724	1,490185	1,077886	0,894560	-1,826630	-0,165269	-1,217361	-0,102092	-0,208439	-0,103226
ID3661699	ANXA1	DCPS	-1,175541	-0,465176	-0,704449	1,916693	1,002978	1,463139	0,111551	-0,115151	-0,952392	0,339989	-0,533250	-0,803395
ID1684	STMN1	PTN1	-0,915084	-0,039414	-1,073307	1,289569	0,961000	0,625612	-2,204423	-0,062905	-0,128480	1,384134	0,232090	0,277240
ID1782			-0,484882	-0,534304	-0,298989	-0,101505	1,294182	1,000390	-1,259979	-0,048690	-1,153547	0,913006	0,068330	0,834240
ID2318312			-0,142282	0,117246	-1,014335	0,356768	0,814315	1,866161	-1,407963	-0,013432	-0,115474	-0,152566	-0,281769	-0,064809
ID305			0,139389	0,130898	0,155550	0,217262	0,277977	0,207905	0,115694	0,115857	-2,160516	0,137283	0,153926	0,166906
ID2617667			-0,659090	-0,461336	-0,244336	0,724801	1,980422	0,459268	-1,024529	0,138024	-0,800146	0,313923	-0,403887	0,055367
ID183880			0,292865	0,296926	0,302210	0,288018	0,291134	0,289186	-0,842138	0,142397	-1,796350	0,258622	0,270114	0,271671
ID1467	CPNS1		-1,141619	-0,162479	0,234890	1,362281	1,154275	0,798371	-2,049002	0,263703	-0,878831	0,527766	-0,178895	0,201481
ID2912567			0,156946	0,175552	0,170336	0,172497	0,173159	0,161285	-1,249988	#DIV/0!	#DIV/0!	0,168834	0,175997	0,189008

Table S-4: cluster 6

Cluster 6 ID[1]	Protein[2]		Z-score[3] co_0 min	co_30 min	co_60 min	co_24 h	co_48 h	co_72 h	Pl_0 min	Pl_30 min	Pl_60 min	Pl_24 h	Pl_48 h	Pl_72 h
ID232052			0,177923	0,169317	0,201046	0,166393	0,125181	0,142631	0,159560	-1,643747	0,070164	0,184162	0,158458	0,134952
ID1785181			0,321560	0,320422	0,321036	0,334212	0,327466	0,327587	0,287335	-1,479481	0,298902	0,239568	-0,590704	-0,648010
ID613			0,232215	0,235387	0,234433	0,250937	0,245832	0,239320	0,144160	-1,417680	0,189101	0,191899	0,137944	-0,989993
ID1660	SSRD		0,454554	0,720451	0,899359	-0,629561	0,149751	-0,257891	0,044025	-1,179862	0,435935	-0,198745	-0,344657	-0,143046
ID1705			0,600389	0,980464	0,843559	0,409812	0,335579	-1,196145	-0,366406	-1,133027	-0,361486	-0,061107	1,581843	-1,648753
ID1780185			0,239510	0,240565	0,240339	0,242229	0,258342	0,256046	0,211799	-1,075116	0,206555	0,124863	-1,024667	0,147669
ID1543			0,263439	-0,990895	0,172144	0,277326	0,295980	0,296551	0,183515	-1,073352	0,143695	0,145133	0,181387	0,141360
ID1618	CBX3		0,232135	0,247815	0,237546	0,248852	0,212074	0,255079	-1,001749	-1,025244	0,178494	0,151850	0,194981	0,159147
ID1564			0,214692	0,211360	0,301936	0,231973	0,233923	0,287687	0,133463	-1,008937	0,179953	0,147730	0,149096	-1,045943
ID1819877			0,241842	0,218926	0,216670	0,225544	0,202629	0,210115	0,211178	-0,998113	0,230975	0,215629	-1,029397	0,168370
ID1785527			0,230723	0,231530	0,231850	0,247148	0,247912	0,241957	0,199440	-0,989045	0,213699	0,022117	0,214347	-1,086149
ID1225	NPM	EF1D	-0,326522	-0,561186	0,119095	1,440664	-0,010086	-1,554846	0,837948	-0,904178	0,184751	0,785969	-0,185327	0,370211
ID3085623			0,901266	1,063323	0,993527	0,295416	0,220826	-0,536268	-0,698837	-0,857053	-0,320850	0,177794	0,200782	-1,395477
ID1657			1,331529	0,989409	0,239167	0,108978	0,159248	0,065423	-0,729109	-0,808145	-0,436839	-0,314979	0,507237	-1,190663
ID2110071			0,277891	0,283416	0,277564	0,301174	0,290248	0,295195	-0,776248	-0,783573	0,228894	0,220159	-0,782813	0,223133
ID1582			0,607445	0,568800	-0,313954	-0,393057	-0,903899	0,269977	-0,405127	-0,760260	-0,265769	-0,170166	0,616335	1,107135
ID1787194			0,268746	0,269665	0,269259	0,284099	0,281419	0,281993	0,253257	-0,757207	0,255078	0,194261	0,232725	-1,784730
ID2970570			-0,102428	0,156230	-0,114767	0,045038	0,723876	0,326052	0,619287	-0,618064	-1,128500	-0,143751	0,584561	-0,383472
ID1363	C1QBP		0,212075	1,359461	0,879377	-0,110284	-0,518612	-1,193202	-0,240447	-0,606542	1,782673	-0,527210	-0,688464	-0,480627
ID1837103			0,403905	0,022169	0,509670	-0,576590	-0,484390	-1,877453	0,490232	-0,531266	0,093416	0,495469	0,423651	1,155054
ID1563			0,915075	0,525933	1,211148	-0,100356	-0,014687	0,009565	-0,265476	-0,500979	-0,383142	-0,400245	-0,303087	-0,793811
ID1209	NPM		-0,429526	0,568407	-0,096088	0,928836	0,771247	-0,964509	1,301729	-0,488119	-1,513679	-0,026839	-0,632304	0,574135
ID1265			-0,282654	0,590726	-0,424907	0,982088	-0,092118	0,530458	0,911298	-0,460706	-0,915029	-1,033179	-0,213770	0,149500
ID2298466	ERP29		0,028866	0,189903	0,071678	1,409942	0,237358	-0,831381	-0,318835	-0,458806	-0,420324	0,113471	-0,532606	0,539102
ID3184881			0,884173	1,274161	1,035239	-0,001580	-1,775753	-0,748351	-0,675604	-0,339917	-0,375858	0,043059	1,031441	-0,340245
ID2588249	ANXA2		0,597310	1,222507	-0,025040	0,147886	0,195698	0,647906	-1,284759	-0,259745	0,075112	-0,694441	-0,551518	-0,244525
ID2000013	TCTP		0,279419	0,604487	0,804987	1,020296	-0,368492	-1,656056	-0,653580	-0,111503	-0,190780	1,113938	0,099631	-0,663862
ID1819121			0,398432	0,071901	0,323549	-0,209676	-1,570946	-0,212359	0,332534	-0,091606	0,577271	2,096721	-0,491285	-0,700354
ID1676			0,099597	0,734210	1,335952	-0,744898	-0,165032	-0,385844	0,320410	-0,072621	0,179359	-0,272660	-0,336638	-0,760000
ID1864524	ID1405		0,743065	0,268087	0,915578	-1,140100	0,093461	0,818705	-0,524073	-0,028501	-0,504057	0,317549	-0,310745	-0,569581
ID2300514			0,364332	0,503722	0,347735	-0,388593	-0,746600	-1,848438	-0,013175	-0,012838	0,108222	0,584408	0,715132	0,532195
ID3056834	PRDX4		0,809024	1,127921	0,896927	-0,391522	-0,203885	-2,186996	-1,208121	-0,006182	-0,054850	0,917799	0,187099	0,342234
ID522			0,189099	0,192066	0,193164	0,211642	0,233703	0,202581	0,129183	0,050843	0,143773	-0,055556	0,134655	-1,626333
ID3435858	QCR1		0,156119	0,161757	0,140800	0,150347	-2,093173	0,128385	0,126712	0,059234	0,103060	0,112105	0,299155	0,160233
ID642			0,167827	0,170712	0,169578	0,185608	0,180358	0,177894	0,106674	0,080008	0,124872	0,125397	0,107411	-1,564988
ID306			0,138267	0,124190	0,123558	0,216713	0,238022	0,195968	0,153583	0,098056	-2,859515	0,157268	0,160732	0,170545
ID569671			0,450070	0,851529	0,421003	0,657845	0,272090	-1,096932	0,291619	0,100156	0,351477	-0,892515	-0,145103	-1,484368
ID9000			1,509801	1,450733	1,450778	-0,221177	-0,710737	-0,038755	-0,414462	0,100503	-0,683984	-1,683185	-0,962856	-0,217455
ID2033833			0,122838	0,090184	0,104180	0,167151	0,127803	-1,533064	0,161991	0,101769	0,154806	0,225607	0,179657	0,153481
ID2006762	CALU		0,183733	0,199550	0,175367	0,184744	0,264432	0,202503	0,131204	0,106448	0,141396	0,090421	0,004576	-2,037536
ID2204207			0,164232	0,186378	0,179409	-1,370596	0,260532	0,165619	0,129659	0,111574	0,127747	0,093669	0,315041	0,091833
ID3763337			0,208699	0,198654	0,207957	0,203814	0,163188	0,188998	0,174779	0,117870	0,154274	0,076597	-1,701339	0,025659
ID315			0,173581	0,174663	0,150247	0,204985	0,222056	0,216093	0,185804	0,122738	0,035419	0,127862	0,141388	-1,526956
ID1266			0,736975	1,694932	0,539823	-0,150002	-0,445835	-0,687398	0,294760	0,134543	-0,485029	-0,708932	-0,609715	-0,491355
ID1792282			0,162208	0,165944	0,165861	0,177117	0,179721	0,170114	0,124213	0,136979	0,138234	0,111935	0,101759	-2,141468
ID1788919			0,163497	0,167242	0,166771	0,182952	0,180050	0,177914	0,132192	0,139268	0,142423	0,053337	0,108486	-1,600797
ID1228			0,764898	0,876263	0,914077	-0,501836	-0,863510	-1,300381	0,886385	0,147873	0,218135	-0,395018	-0,600699	-0,244942
ID1816422			0,205598	0,166982	0,152104	0,173737	0,147921	0,150748	0,147770	0,178206	0,186747	0,181604	-1,572373	0,066859
ID244116	DPOD2		0,235343	0,241087	0,234280	0,255423	0,259895	0,262231	0,210537	0,184045	0,170298	-1,562495	-1,055679	0,174412
ID1987569			0,104863	0,080558	0,122687	-2,076626	0,172321	0,118826	0,147995	0,185905	0,222109	0,116098	0,131768	0,183364
ID24906			0,259638	0,262884	0,260425	0,276088	0,269112	0,258219	0,146444	0,192299	-1,016907	0,150440	0,242094	-1,341619
ID2596060			0,375272	1,379010	0,065808	0,033488	-0,828059	-1,526795	0,799930	0,193627	0,202730	-0,105346	-0,232126	-0,383876
ID1866181			0,292298	0,213022	0,228259	0,159229	0,291315	0,183399	0,117647	0,219967	0,155838	0,089951	0,044883	-1,843427
ID2728784			0,118110	0,028171	0,123886	0,119171	0,088563	-1,559543	0,242182	0,225229	0,230972	0,156586	0,147416	0,118402
ID246907			0,204214	0,194934	0,201673	-2,235262	0,190637	0,185915	0,205035	0,237124	0,190336	0,232950	0,234994	0,215686
ID1814733			0,031924	-0,085102	-0,011261	0,332842	-0,326725	0,028422	0,197612	0,241068	0,613754	1,420355	-0,678212	-1,409588
ID2142601			0,226370	-1,016402	0,192510	0,199642	0,124908	-1,011580	0,295899	0,252540	0,285586	0,235056	0,178333	0,095901

Table S-4: cluster 6

ID)[1]	Protein)[2]		co_0 min	co_30 min	co_60 min	co_24 h	co_48 h	co_72 h	PI_0 min	PI_30 min	PI_60 min	PI_24 h	PI_48 h	PI_72 h
ID3040913			0,753864	0,712502	1,314248	0,431628	-0,079138	-2,416702	0,395085	0,254275	0,283590	-0,378370	-0,794707	-0,570869
ID1313			0,269022	0,274326	0,271772	-1,038598	0,271677	0,274388	0,273923	0,284442	0,285647	0,236719	-0,784585	-0,819203
ID338			0,281330	0,286437	0,266512	0,306628	-0,618197	#DIV/0!	0,274854	0,292850	0,291764	-1,037010	0,230018	-0,692784
ID2094921			0,354500	0,593593	0,333224	0,138284	-0,345830	-1,628573	0,577581	0,303231	0,799029	0,394419	-0,101658	-1,319196
ID360270	ACTG	ACTB	1,072821	0,972601	1,446015	0,463309	0,066091	-0,395625	-1,281505	0,319539	-1,425702	0,466645	-0,783761	-0,803767
ID2447768			0,587076	0,307201	0,094573	-1,155578	-0,450954	1,970987	-0,577301	0,345571	-0,537315	-0,492092	-0,195973	-0,019219
ID1712			0,586357	1,141883	1,071931	0,286704	0,190969	-0,952283	-0,168881	0,347506	0,214998	-0,761995	-1,178761	-0,968928
ID2102478			0,257804	0,439468	0,392120	-1,455589	0,434225	0,182738	-0,110959	0,359732	0,865696	0,527843	-1,137682	-0,623435
ID1556			0,536652	1,064015	1,449597	-0,512212	-0,551026	-0,193889	0,537465	0,364854	0,247104	-1,348723	-1,042192	-0,888826
ID2108161			0,311175	0,313953	0,312736	-1,383941	0,314980	0,314213	0,301464	0,365731	0,303406	0,295137	-0,879622	-0,852669
ID1472			0,769019	1,153929	1,140232	0,138645	-0,867921	-1,460751	0,316149	0,381201	0,439001	0,731564	-1,049350	-1,508828
ID1398			0,191253	0,497479	0,549937	-1,751959	0,423624	0,950405	-0,674809	0,447810	-0,554733	-0,171183	0,308294	-0,258912
ID1700			-0,080796	-0,146134	-0,103117	-0,142172	-0,010027	-0,160430	-0,832252	0,451847	-0,403896	0,840662	0,437103	0,359377
ID75108			0,120472	1,108755	0,804502	0,129196	-0,392188	-0,961860	-0,140214	0,473511	0,669106	-0,919414	-0,789916	-0,331804
ID1135			0,445480	0,299759	1,120503	-0,085598	-0,063570	0,315901	-0,258379	0,474761	0,556626	-1,292011	-1,118675	-0,717798
ID1193			-0,425872	-0,149617	-0,607475	0,247747	0,134114	-0,202793	1,464995	0,479883	-0,438413	-0,900816	0,192739	0,220247
ID2093028			0,090793	0,211905	0,012296	-0,008110	-0,222936	-1,742673	0,581064	0,482427	1,367237	-0,329029	0,337048	-0,862277
ID2305694			-0,261342	1,474095	0,381184	0,371004	0,229555	-0,129428	-0,168937	0,507680	-0,798991	-0,339258	-0,330474	-1,019902
ID707			-0,112042	0,606786	0,282151	1,647626	0,516515	-1,484677	-0,030226	0,523410	0,309406	-0,708145	-0,650773	-1,077069
ID229688			-0,085347	1,170354	-0,101412	0,402330	-0,774346	-1,315947	1,014706	0,652583	-0,804651	-0,021064	0,138532	-0,281004
ID1574			0,410180	0,674523	1,959455	-1,054710	-0,581758	-0,621294	0,217297	0,663396	0,619424	-1,149529	-0,679511	-0,254992
ID1028			1,469621	0,626926	0,599170	-1,018546	-0,512931	-1,376906	0,693638	0,771941	0,672024	-0,466401	-0,282199	-1,292938
ID331			0,096967	-0,613781	0,029335	0,277815	0,349236	-0,989733	0,334954	0,796610	0,492800	-0,807678	1,168558	-1,337004
ID1339			0,447453	0,217752	0,211158	0,213729	0,091287	0,112017	-0,121998	0,815823	-0,364088	-1,265780	-0,365239	-0,308559
ID2096810			0,403516	0,367724	0,233359	0,189785	0,122716	-1,633221	0,328724	0,849303	0,421699	0,722536	-0,543968	-1,281539
ID2439918			0,027837	0,046550	0,072674	0,064432	0,191014	-0,125467	-0,236302	1,162986	-0,434850	-0,180885	-0,301387	-0,331824
ID3763685			0,234102	0,062842	0,111190	0,374921	0,342619	-0,403992	-0,869825	1,189885	-0,686743	-0,213312	-0,021342	-0,173674
ID1871260			0,152067	-0,137078	0,713416	0,698646	-0,148592	-0,961356	-0,099697	1,207755	-0,018683	-0,411292	0,349862	-1,447871
ID1340			-0,037142	0,002643	-0,086031	-0,271873	0,012915	-0,305770	0,074650	1,246293	-0,359290	0,288975	-0,368056	-0,125071
ID3019322			0,029473	0,202600	0,446690	-0,091854	-0,077915	0,489046	-0,491633	1,263988	0,073095	-1,285097	-0,320694	-0,558974
ID1499			-0,053353	0,102482	0,381250	-0,059597	-0,052320	-0,305947	-0,048547	1,641922	0,133019	-0,673222	-0,433683	-0,800309
ID105671	GRP78	ENPL	0,954330	0,868652	1,220776	-0,828463	-0,879091	-0,694866	-0,503617	1,751938	-0,530508	0,018452	-1,120678	-0,252313

Table S-4: cluster 7

Cluster 7 ID[1]	Protein[2]		Z-score[3] co_0 min	co_30 min	co_60 min	co_24 h	co_48 h	co_72 h	PI_0 min	PI_30 min	PI_60 min	PI_24 h	PI_48 h	PI_72 h
ID493	HNRL2		0,627728	0,703022	0,709499	1,051606	0,667518	0,378256	-0,478784	-2,343315	-1,210590	-0,662781	0,216786	0,175360
ID305540	HNRPC	STRAP	0,668119	0,658948	0,995748	0,683949	0,601975	-0,302383	-0,844230	-2,213401	-1,095287	0,953825	-0,079991	0,211185
ID1366			0,403784	0,644011	0,441850	0,767723	0,878839	0,792502	-0,860805	-2,192453	-1,129715	-1,024480	0,337603	0,685020
ID2964442	HNRPC		0,714575	0,691123	1,026404	0,957198	0,599491	-0,256590	-1,004996	-2,191490	-1,112125	0,145956	0,149088	0,317855
ID3931	HS90A		1,024181	0,985751	0,848049	0,614785	0,304624	0,021352	-1,038179	-2,149873	-1,356116	0,155700	0,574698	0,053955
ID1534			0,654871	0,525784	1,350971	0,465167	0,962918	0,590981	-0,447849	-2,123196	-0,300962	-0,412845	-0,528941	-0,840111
ID1267	SGTA		0,684014	0,842713	0,866538	0,804632	0,589302	0,644716	-1,176807	-2,095750	-1,440975	0,103965	0,058123	0,145520
ID1518			0,715873	0,614652	1,760789	0,495398	-0,089602	-0,298576	-0,559900	-2,075203	-0,631953	-0,194394	-0,099076	0,313393
ID929	NP1L1	SYAP1	0,472365	0,908349	0,671950	1,187082	0,683102	0,366999	-1,551640	-2,068966	-0,947594	0,326643	-0,204499	0,237870
ID1017			0,874991	0,561922	1,421464	0,309534	0,234028	0,662265	-0,941201	-2,057528	-0,676999	0,567751	-0,380464	-0,433826
ID3392731	CNDP2	PEPD	0,676176	0,802531	0,851123	0,941277	0,585051	1,219672	-1,008906	-2,041969	-0,989358	-0,538440	-0,311511	-0,320254
ID104582	ENPL		0,355662	1,279399	0,819268	0,324126	0,447755	1,013914	-0,619556	-2,038112	-0,057001	0,663431	-0,820457	-1,202571
ID936	BASP		0,197063	0,835250	0,254955	0,992720	1,434359	0,920827	-1,050429	-2,031439	-1,084655	-0,380370	-0,119374	-0,063999
ID1099	CSN4	MPI, BPNT1	0,545964	0,896894	1,077212	0,951028	0,913250	0,422605	-0,649553	-2,017944	-0,807850	0,579053	-0,933634	-0,832262
ID1382	EF1B		0,192779	0,858346	0,126755	1,696783	0,947324	0,189147	-0,784411	-1,980088	-1,264170	0,626152	-0,443032	-0,009047
ID1019	IF4A1	ACTG, HNRPF, NUDC	0,722866	0,880364	1,095093	1,248401	0,663950	-0,219396	-1,146375	-1,978949	-1,035553	0,630541	-0,244824	-0,458482
ID926	NP1L1		0,622636	1,025689	0,746665	1,087216	0,898214	0,413886	-1,374695	-1,971618	-0,974813	0,135878	-0,472674	-0,102414
ID974	SYDC		0,327720	0,641134	0,539564	1,697156	1,000461	0,170879	-1,355442	-1,946861	-0,928316	-0,216328	0,001487	0,014464
ID878	FKBP4	HMCS1, HNRPK	0,545080	1,024110	0,688823	1,526364	0,707379	0,380660	-1,089902	-1,929021	-1,229707	-0,049106	-0,362822	-0,224135
ID985			0,259764	0,544212	0,701409	1,180323	1,352812	0,846764	-0,879162	-1,917728	-1,299028	-0,778152	-0,072198	-0,133554
ID3228910	SAKS1	SAE1	0,608298	0,648070	0,595225	0,666571	0,308735	0,250144	-1,711521	-1,916799	-1,056833	1,096174	0,201360	0,584621
ID1312	OTUB1		0,374256	0,401683	0,567452	1,143921	1,351355	0,932233	-1,456146	-1,912874	-0,902731	-0,497394	-0,129062	0,002958
ID2917416			0,432862	0,475883	0,586910	0,874668	0,766063	0,570529	-1,356976	-1,891774	-1,270426	-0,616295	0,642018	0,632466
ID566	PSMD2		0,497470	0,587671	0,706933	1,263641	0,813619	0,842948	-1,284138	-1,882671	-1,326437	-0,077873	-0,341248	0,180615
ID1152	ARSA1		0,782641	0,800777	1,154849	0,669051	0,924582	0,910718	-1,315410	-1,875645	-0,846006	-0,733209	-0,282271	-0,373379
ID1025			1,041879	1,000348	1,329365	0,701124	0,615921	-0,495790	-0,817559	-1,859036	-0,387832	-0,220370	-0,353727	-0,609415
ID2924537			0,360222	0,363995	1,070155	1,161163	0,710657	-0,310996	-1,257134	-1,854951	-1,389343	0,471145	0,173812	0,619061
ID459	RCN1		0,381057	0,569151	0,350353	1,218240	1,057459	1,145217	-0,982958	-1,848300	-1,284378	-0,853021	0,023739	0,010186
ID262545			0,621239	1,048365	1,162080	0,429555	1,157172	0,348698	-0,677928	-1,847171	-0,865339	-0,465999	-0,459447	-0,567724
ID788	HNRPK		0,075089	0,703616	0,450114	1,031482	0,803292	1,416353	-0,439242	-1,833113	-1,775180	-0,171142	0,016573	-0,320627
ID1631			0,191276	0,861387	-0,053322	1,459567	1,276342	0,733182	-1,381814	-1,830800	-0,499555	-0,763232	-0,207169	0,023329
ID1214	SUGT1		0,682407	1,001028	0,627177	1,299882	0,706970	0,484406	-1,207820	-1,828141	-1,391382	0,289520	-0,388494	-0,203172
ID2816455	HS90A		0,803786	0,863436	0,926851	0,906379	1,075052	-0,502975	-1,089294	-1,821270	-0,862607	-0,098226	-0,194436	-0,031251
ID1241	PP2AB	CT077	0,225754	0,725088	0,801043	1,633877	0,964487	0,655992	-1,030579	-1,819979	-1,115046	-0,408451	-0,476846	-0,257453
ID1203	HNRPC		0,403104	0,509031	0,595016	1,139930	0,520255	1,173812	-1,372046	-1,819241	-1,448193	0,355436	-0,204843	0,236599
ID2830369			0,949802	1,385808	0,912228	1,112177	0,533363	-0,239419	-0,948673	-1,817473	-0,874212	-0,109445	-0,577342	-0,354175
ID1304	PCNA		0,364435	0,555473	0,501602	0,988399	1,370028	1,318715	-1,188003	-1,815887	-0,876117	-0,421183	-0,209810	-0,692947
ID2818369	P3H1		0,787239	0,994265	1,114915	0,956263	0,828269	0,089965	-1,261429	-1,812472	-1,134296	0,057576	-0,323789	-0,282111
ID871	FKBP4		0,432793	0,912608	0,634281	1,173978	0,800662	0,921422	-1,500427	-1,806698	-1,242953	-0,112524	-0,202620	-0,038653
ID1153	TRA2B	CSN4	0,497241	0,840377	0,735820	1,001033	0,905369	0,875792	-1,652277	-1,800788	-1,127542	-0,176847	-0,093363	-0,049026
ID1269	TXNL1	ANXA2	0,634081	0,950541	0,421969	1,717879	0,863283	0,106911	-1,288316	-1,794368	-0,879878	0,227145	-0,362792	-0,539669
ID476			0,367204	0,610984	0,511194	1,353082	0,878006	0,833309	-1,087894	-1,793670	-1,533736	-0,374133	-0,077616	0,219736
ID3231778			0,476983	0,679976	0,651047	1,290426	0,824801	1,055594	-1,065018	-1,789489	-1,293169	-0,622350	-0,415555	0,051166
ID1784	PEA15		0,625336	0,861371	0,533631	0,978086	0,949504	0,965896	-1,864526	-1,783864	-0,629667	-0,306107	-0,239287	-0,166899
ID279386	SET		0,070006	0,312854	0,485676	1,379692	1,036754	1,164922	-1,156300	-1,777229	-1,351971	-0,132239	0,042234	-0,107458
ID997	PRS7		0,459247	0,809162	0,668149	1,551122	0,945984	0,856654	-0,742596	-1,774782	-1,115672	-0,423596	-0,762550	-0,577021
ID1797353			0,556658	0,707205	0,931521	0,863104	0,763842	0,517632	-0,735039	-1,764279	-0,340087	-0,381244	-0,678447	-0,536178
ID13083	TXND5	SH3G1	0,711830	0,920347	0,707922	0,801653	0,995502	0,984473	-1,599898	-1,759992	-0,732267	-0,536233	-0,354860	-0,272535
ID435	DREB		0,510588	0,767868	0,492399	1,459542	0,968520	0,915906	-0,761574	-1,757421	-1,210581	-0,948114	-0,355457	-0,318704
ID17270	CSN4		0,690768	0,889335	0,998869	0,750340	0,834652	1,041366	-1,090252	-1,754594	-1,297446	-0,738121	-0,540730	0,031284
ID1158			0,583231	0,776802	0,951242	1,017028	0,642416	0,346886	-1,490478	-1,748366	-1,402270	-0,003719	0,110052	0,216249
ID428	ICAL	DREB	0,428824	0,675681	0,173273	1,474877	1,067572	1,078532	-0,925412	-1,746144	-1,182892	-0,765031	-0,295604	-0,174934
ID1523			0,316662	0,493219	1,014868	0,946682	0,119008	-1,090249	-0,704773	-1,744737	-0,981414	1,976607	0,272442	-0,124163
ID379418	MYG1	SHLB1	0,478961	0,935828	0,516016	1,182352	1,012436	0,971670	-1,189042	-1,744534	-1,185933	-0,847235	-0,290615	-0,051712
ID1392	PSME2		0,639896	0,548117	0,980971	0,597345	1,155444	1,302591	-1,293837	-1,744285	-1,089857	-0,465757	-0,299898	-0,447170
ID1105			0,598138	0,792909	0,631579	0,758244	0,934879	1,030974	-1,676505	-1,742497	-1,006593	-0,696830	0,028458	0,173037
ID445851	DNJA2	APMAP, SNX6	0,137967	0,647943	0,389054	1,305846	0,657249	0,853509	-0,486057	-1,734380	-1,036682	-0,081738	-0,284970	-0,388175

Table S-4: cluster 7

ID[1]	Protein[2]		co_0 min	co_30 min	co_60 min	co_24 h	co_48 h	co_72 h	PI_0 min	PI_30 min	PI_60 min	PI_24 h	PI_48 h	PI_72 h
ID357613	SAE1	ACTG, TMOD3	0,754072	0,852076	0,883150	1,154350	0,960031	0,829986	-0,666346	-1,727219	-1,322963	-0,588424	-0,584982	-0,690838
ID818	UBP14		0,758152	0,870088	0,899250	1,012440	0,848810	0,774664	-1,479367	-1,726890	-1,221585	-0,339268	-0,418688	-0,062425
ID1701	STMN1	1433S	0,095998	0,668823	0,579117	1,604853	1,285645	0,727654	-1,072386	-1,725161	-0,843658	-0,301687	-0,883980	-0,210640
ID1412	PA1B2	PSME1, DDAH2	0,318106	0,787583	0,683044	1,175495	1,218882	1,134520	-1,182849	-1,724245	-0,459757	-0,699339	-0,841508	-0,584767
ID1268	CHM4B	RPB3	0,240095	0,947644	0,520337	1,374477	1,222893	0,569214	-1,169769	-1,721924	-1,245963	0,145441	-0,269764	-0,576321
ID1157	SET		0,695495	1,035701	0,623141	1,253798	0,997339	0,869187	-0,951440	-1,716331	-0,870657	-0,466843	-0,707331	-0,878769
ID485	HS105		0,398600	0,702727	0,452257	1,390898	1,020177	0,732700	-1,193529	-1,714648	-1,562906	-0,439798	-0,068172	0,171744
ID949	RUVB2	NEMO	0,510984	1,000116	1,103144	1,122212	0,786104	0,906778	-0,460924	-1,703116	-0,776372	-0,700109	-1,063806	-0,900039
ID1769	COTL1		0,766211	1,150046	1,015078	1,035116	0,426369	0,372531	-1,721511	-1,698420	-0,899980	0,077526	-0,625040	0,121456
ID402687	TBA1B		0,685154	0,776043	0,855367	1,001366	0,929069	1,174159	-0,838207	-1,696437	-0,628786	-0,478129	-1,220912	-0,678219
ID1042	SAHH		0,399717	0,479982	0,380349	1,134306	1,121091	1,162889	-1,661951	-1,695306	-0,975736	-0,567167	-0,222685	0,302719
ID136093			0,344319	0,378383	0,428265	1,235412	0,822111	1,178317	-1,107851	-1,693582	-1,548496	-0,087217	-0,404302	0,432836
ID575	HS90A		0,322791	0,590197	-0,095027	1,540257	1,280614	0,633036	-1,181988	-1,690259	-1,295069	0,144565	-0,314973	0,101997
ID559	HS90A	DP13A, SRC8	0,745476	1,103248	0,764141	1,227777	1,208645	0,240000	-0,856982	-1,689152	-1,023877	-0,423827	-0,724722	-0,676684
ID51068			0,320851	0,883295	0,447891	0,961366	1,541309	0,860128	-1,247197	-1,686401	-0,739740	-0,203083	-0,381669	-0,807521
ID545	UBP5		0,449417	0,635266	0,457547	1,082312	1,099259	0,954487	-1,618066	-1,684291	-1,220152	-0,388752	0,024996	0,110791
ID576			0,575424	0,503152	0,393329	1,373615	1,007105	0,565206	-1,452621	-1,684182	-1,039319	0,117703	-0,291251	-0,038735
ID2005865			0,641942	1,050369	0,545802	0,162945	1,365128	0,548730	-1,280813	-1,683264	-0,029115	-0,914828	-0,614627	-0,020976
ID1494	NDUS3	HSPB1, PSB4	0,345819	0,859753	1,028823	1,505266	0,866165	0,596221	-0,917295	-1,681734	-1,260443	-0,271394	-0,595003	-0,544026
ID798	TCPE	PNCB, TCPQ, HDAC2	0,510229	0,784062	0,595826	1,437272	1,070769	0,676611	-1,307180	-1,678261	-1,318183	-0,183459	-0,455418	-0,178133
ID469	UBA1	TIF1B	0,618020	0,671381	0,584918	1,031357	1,220648	1,054576	-0,885958	-1,677660	-1,393676	-1,104558	-0,280790	-0,114398
ID622	GSPT1	HS90A, ERF3B	0,607663	0,749844	0,561847	1,420910	1,016923	0,723101	-1,326602	-1,672407	-1,266812	-0,633305	-0,332016	-0,007473
ID130985			0,557544	0,509946	0,661668	1,135271	1,068430	0,938106	-0,854947	-1,669280	-1,441932	-0,339739	-0,277275	-0,372726
ID1077	AHSA1	ACTG, CSN4	0,642896	0,912737	1,036461	1,342840	0,562773	0,437827	-1,668260	-1,665237	-0,874590	0,237452	-0,544917	-0,360620
ID168476			0,006482	1,009724	0,958398	1,176938	1,028355	0,660516	-0,953313	-1,663269	-0,998679	-0,366782	-0,869559	-0,080506
ID257339			0,508031	0,950818	0,737736	0,899481	1,225040	0,713487	-1,223138	-1,660567	-0,845362	-0,057702	-0,473366	-0,788884
ID809	ULA1		0,638858	0,567883	0,846302	1,010342	1,079452	1,009655	-1,127387	-1,656953	-1,492611	-0,671336	-0,296238	-0,075801
ID1669			0,540639	0,911932	1,257733	0,630714	0,997717	0,082959	-1,837060	-1,656283	-0,697069	0,288056	-0,229819	-0,217504
ID1772			0,642930	1,370469	0,996422	0,987940	0,183343	-0,053173	-1,224799	-1,648506	-1,094091	0,699015	-0,377346	-0,307450
ID484			0,476188	0,878256	0,487872	1,268899	0,831724	1,118821	-1,074992	-1,643239	-1,503889	-0,561347	-0,351789	-0,066841
ID941	CD2B2		0,570502	1,077779	0,711406	0,866684	1,264231	0,841678	-0,872314	-1,641959	-0,600298	-0,316613	-0,951349	-1,028899
ID1399	3HIDH	PSME1, CDV3	0,742539	0,855008	1,039132	0,368676	1,098965	1,324662	-1,237516	-1,641109	-0,783404	-0,663458	-0,538927	-0,730433
ID1000			0,257903	0,701229	0,269702	1,459484	1,407768	0,440453	-1,511779	-1,639631	-0,933117	-0,262538	-0,241235	-0,013875
ID25129			0,191977	0,737930	-0,110965	1,889248	1,134521	-0,203242	-1,101895	-1,638624	-0,478558	-0,075809	-0,257952	-0,105583
ID2286			0,117067	0,759780	0,697370	1,457241	0,808292	0,509066	-0,879167	-1,638304	-0,643083	0,298587	-0,736328	-0,675875
ID571	HS90A		0,857132	1,219287	0,924669	1,064507	0,994116	-0,040819	-0,914801	-1,637035	-0,985072	-0,284924	-0,460931	-0,807361
ID486	HS105		0,420962	0,706453	0,425241	1,523945	1,028784	0,661780	-1,255072	-1,630076	-1,519802	-0,359468	-0,186859	0,094246
ID1174	IDH3A		0,754144	1,322734	0,643621	1,237645	0,175788	0,976385	-0,906133	-1,628994	-1,194918	-1,101711	-0,224323	-0,329668
ID1234	TALDO	PP1B, KPYM	0,724647	1,087263	0,910962	0,848168	0,660772	0,624779	-1,602732	-1,627301	-1,269414	-0,322719	-0,325457	0,210352
ID1359			0,426048	0,411247	0,894205	0,385377	1,043650	1,207187	-1,530715	-1,620764	-1,027220	-0,402047	0,075841	0,036678
ID935	RD23A		0,252109	0,726269	0,419046	0,997883	1,608214	0,999143	-1,378500	-1,619155	-0,913736	-0,443692	-0,281577	-0,476927
ID1402	PSB7		0,418528	1,060886	0,948404	1,565751	0,742449	0,528724	-0,950138	-1,618481	-1,126437	-0,765004	-0,747825	-0,248108
ID1106	CAPG		0,342492	1,009323	0,705538	0,945956	1,010081	1,349036	-1,172933	-1,617635	-0,820637	-0,964792	-0,736548	-0,291079
ID1167	GLRX3	TXNL2, SNAG, CPIN1	0,591179	0,884556	0,641792	1,292210	0,896487	0,931426	-1,495727	-1,616788	-1,119052	-0,554342	-0,443447	-0,146878
ID477	HS105	GPIA1	0,299375	0,575272	0,414852	1,616015	1,080227	0,809848	-1,085031	-1,606009	-1,528182	-0,218610	-0,371615	-0,040796
ID490	HSP74		0,319345	0,671047	0,313364	1,140084	1,027538	1,161472	-1,388603	-1,604501	-1,452359	-0,651445	-0,011695	0,312892
ID1161	STRAP		0,685239	0,824772	0,995858	1,329553	0,865156	0,593826	-1,460590	-1,600304	-1,087773	-0,586492	-0,498264	-0,207605
ID1227	ACTG		0,693354	0,995778	0,830317	1,346224	0,016035	1,125423	-1,104468	-1,598828	-1,112070	-0,327934	-0,432217	-0,513598
ID172969	PSA3	TPD54	0,411117	0,769076	0,641599	1,112480	0,916809	1,132387	-1,392467	-1,586881	-1,410764	-0,549246	-0,338977	0,157554
ID879	HNRPK	TBL1R, HMCS1, TBA3	0,467912	0,745880	0,494826	1,016355	1,057089	1,540503	-1,234375	-1,585841	-0,739700	-0,609342	-0,824100	-0,481544
ID793	TCPA		0,509824	0,909062	0,597757	1,408988	1,015335	0,573268	-1,459707	-1,582601	-1,319709	-0,496170	-0,279352	-0,000738
ID1196	IF2A		0,551415	0,926903	0,854586	1,467490	0,869614	0,780475	-1,043556	-1,579205	-1,050558	-0,741401	-0,775403	-0,445710
ID176254	PDIA6	F10A1, PABP2, PRS6B	0,448488	1,214205	0,648548	0,919924	1,009795	1,039563	-1,216586	-1,576322	-1,085798	-0,302023	-0,605933	-0,569367
ID149908			0,178507	0,749966	0,134293	1,265036	0,887474	1,371909	-1,456351	-1,575265	-0,907433	-0,436881	-0,348394	0,027918
ID1465	IF6		0,378340	1,011514	0,226969	1,751426	0,688340	0,244935	-1,573401	-1,570124	-0,869485	-0,430923	-0,409428	0,444106
ID1258	NACA		0,359951	0,740493	1,014484	0,708657	0,216510	-0,348677	-0,612803	-1,567537	-1,065584	1,691518	-0,195130	-0,519003
ID2926520			0,434202	0,548275	0,982091	0,704512	-0,189514	-0,711992	-0,167206	-1,566016	-1,686839	0,421277	-0,018102	1,354630
ID1430	1433Z		0,473485	0,638927	1,075699	0,838151	0,942268	1,289309	-1,548059	-1,564505	-0,785948	-0,529985	-0,631184	-0,330656
ID1355	MARE1	EFHD2	0,592040	1,130689	0,969452	1,230576	0,744012	0,504565	-1,697985	-1,542941	-0,758866	-0,002114	-0,621796	-0,548161

Table S-4: cluster 7

ID)1	Protein)2		co_0 min	co_30 min	co_60 min	co_24 h	co_48 h	co_72 h	PI_0 min	PI_30 min	PI_60 min	PI_24 h	PI_48 h	PI_72 h
ID1125			0,700702	0,562436	1,345976	1,089259	0,596651	0,809839	-0,667243	-1,541475	-1,260666	-1,204386	-0,491102	-0,241088
ID954	PSMD5	SH3G1	0,938625	0,622875	1,366572	0,775534	1,019775	0,655459	-0,761049	-1,541367	-0,553371	-1,471959	-0,738804	-0,680278
ID1002	PRS6A		0,433778	0,994169	0,617109	1,402992	0,950406	0,772917	-0,836732	-1,537794	-1,465768	0,184258	-0,742212	-0,727059
ID23159			0,468823	0,600590	0,410332	1,272952	1,392393	0,369576	-0,272498	-1,529581	-1,458467	0,581681	-0,605568	-1,084812
ID1165	CNN3		-0,030656	0,306818	0,283095	1,678113	1,393133	0,792327	-0,644561	-1,526549	-1,639065	-0,137276	-0,458817	-0,050881
ID131133			0,397142	0,440605	0,474424	1,190070	1,281585	1,336707	-0,904126	-1,525341	-1,344724	-0,391165	-0,417043	-0,635926
ID283779			0,439315	0,716263	0,595784	1,161291	0,799694	1,373211	-0,625863	-1,524779	-0,747110	-0,197741	-1,245037	-0,794463
ID890	TBA1B		0,429447	0,573079	0,557983	0,764732	1,151352	1,542956	-1,417302	-1,522854	-1,026679	-0,859645	-0,529244	0,121265
ID534785	PP1A		0,697427	1,047093	1,041974	0,971123	0,625824	0,872165	-1,632956	-1,514793	-1,025132	-0,593023	-0,438856	-0,199102
ID508	MSH2	GANAB	0,578279	0,499826	0,567799	1,150298	1,294283	1,235048	-1,124347	-1,512322	-1,260302	-0,781578	-0,343880	-0,498499
ID302775	DCTN2	PABP2, PRS6A	0,670011	0,920489	0,844380	1,317667	0,847518	0,800625	-1,224270	-1,512185	-1,233921	-0,236275	-0,684676	-0,568434
ID1501	TCTP	CBX1	0,683735	1,515529	0,862262	1,460095	0,430989	-0,376412	-0,903024	-1,510187	-0,588898	0,467813	-0,962858	-0,962091
ID1541	PRDX2	GSTP1	0,623370	0,829641	0,680439	0,811610	0,889636	1,103667	-1,531834	-1,499943	-1,609637	-0,336738	-0,042635	-0,001760
ID1536	SFRS3		0,540825	0,654682	0,792415	0,729543	1,195783	0,930829	-1,888990	-1,498388	-0,965431	0,101730	-0,307075	-0,260491
ID4295	P3H1		0,792578	1,230483	1,256644	0,735421	0,573148	0,272571	-0,914838	-1,497832	-0,982986	0,633423	-0,962690	-0,977566
ID940	GDIB		0,571290	0,660771	0,729531	1,151725	1,136931	0,973254	-1,557996	-1,497025	-1,116230	-0,631590	-0,402120	-0,176437
ID3760248			0,649736	0,651985	0,792329	1,150725	1,007557	0,856943	-1,627340	-1,494042	-1,276670	-0,373103	-0,249222	-0,182172
ID1360	MARE1	TBCB, ANXA5	0,572519	1,091482	0,878147	1,040400	0,803976	0,960526	-1,697128	-1,492993	-0,804844	-0,237372	-0,647992	-0,526063
ID2788379			0,317131	0,413271	0,208081	1,384203	1,328738	1,304494	-1,002010	-1,492874	-1,333337	-0,303745	-0,406595	-0,493293
ID491	HSP74		0,625955	0,987357	0,589018	1,283566	1,055868	0,630285	-1,341924	-1,492661	-1,360425	-0,858526	-0,321111	-0,012032
ID1513	GDIR1	LGUL	0,478386	0,894908	0,732590	1,110929	0,977630	1,042264	-1,783106	-1,492374	-0,805833	-0,275722	-0,462250	-0,486353
ID1283			0,435741	0,972378	0,392196	1,236593	1,076926	0,786404	-1,351974	-1,490944	-1,032876	-0,339460	-0,141938	-0,627911
ID1519	PSD10		0,347966	0,792468	0,674642	1,078236	0,978140	0,940780	-1,794779	-1,490526	-0,891860	0,020012	-0,437640	-0,212437
ID1185	IF32	NPM	0,072169	0,881022	0,233656	1,967594	0,590366	0,180806	0,065826	-1,489738	-1,688282	0,103866	-0,800263	-0,091056
ID1409	PSME2	CLIC4	0,596694	0,628256	0,964203	0,883562	1,064185	1,039902	-1,730853	-1,489542	-1,109362	-0,467730	-0,394889	-0,101358
ID1568			0,624237	0,843850	0,766425	0,878012	0,632942	0,609773	-2,075217	-1,483979	-0,720939	-0,562974	-0,297098	0,644225
ID165075			-0,292476	1,084889	0,301605	1,150833	1,174689	0,396067	-1,437998	-1,481245	-0,880716	0,240394	-0,392942	0,196999
ID1005	RINI		0,502942	0,653271	0,548346	1,188442	1,198254	0,877037	-1,786439	-1,479971	-0,981373	-0,780161	-0,244319	0,108931
ID16834			0,171634	0,526788	0,535919	1,265750	1,004838	1,102185	-1,487425	-1,479640	-1,013506	-0,453045	-0,127206	-0,159554
ID550	UBP5	P3H1	0,759769	0,974594	0,809857	1,095984	0,952131	0,852655	-1,435300	-1,476889	-1,123291	-0,814230	-0,414556	-0,384280
ID466	UBE1	MVP, UBA1	0,624949	0,753311	0,582314	1,266232	1,135110	0,927741	-1,141162	-1,476433	-1,284689	-1,146225	-0,346723	-0,180982
ID573	HS90A		0,559572	1,549033	0,404105	1,216387	0,516584	-0,168961	-0,859216	-1,475406	-0,714799	-0,074698	-0,637198	-0,334076
ID1101			0,765858	0,858677	0,914225	0,874117	1,012889	0,930296	-1,422577	-1,473631	-1,201909	-1,023674	-0,308998	-0,181192
ID1419	TIF1B	1433G	0,731239	0,931062	1,208893	0,730983	0,904303	0,954577	-1,497076	-1,469783	-0,873276	-0,462301	-0,688053	-0,586143
ID1243	PPP6	LDHB, PDXK	1,055931	0,926759	1,431911	0,541892	0,609454	0,692270	-1,553557	-1,467421	-0,936359	-0,499963	-0,674625	-0,251285
ID1632	NDKA	COMD1, SODC	0,571859	0,842178	0,762620	1,323650	0,650985	0,463894	-1,987942	-1,466556	-1,034405	0,319127	-0,427210	0,061580
ID1124			-0,353721	1,454359	0,521731	0,960475	0,354296	0,674346	-1,283626	-1,461955	-0,726661	-0,281400	-0,291780	0,363584
ID1154	CNN3	IDH3A	0,736554	1,251095	0,755502	1,323704	0,598699	0,761148	-1,030951	-1,455672	-1,083673	-1,351775	-0,447644	-0,394931
ID958	CDC37	PRS6B, DCTN2, PDIA6	0,630479	1,000963	0,941831	1,210531	0,928449	0,852458	-1,173131	-1,454815	-1,124977	-0,717316	-0,689146	-0,584654
ID1372	TPM3	TPM4, TPM1, TPM2	0,755669	1,017263	1,042898	0,842881	1,036623	0,748091	-0,974370	-1,453969	-0,778215	-1,509015	-0,659008	-0,446102
ID4476			0,871185	1,131889	0,817965	1,071593	0,876479	0,707290	-1,275189	-1,452978	-1,069460	-0,597403	-0,636545	-0,594175
ID1429	1433T	1433Z	0,701933	0,759051	1,041584	0,950664	0,987414	1,063824	-1,503745	-1,430113	-0,898821	-0,670979	-0,699377	-0,469180
ID859	TBA1B	TBA1C, VIME, TBA3	0,792958	0,559568	0,515293	0,956610	1,080950	1,435560	-1,406289	-1,426547	-1,001938	-0,573669	-0,593313	-0,482600
ID1151			0,799401	1,715774	0,674815	0,387233	0,168161	0,701779	-0,659526	-1,421335	-0,220123	0,066914	-0,754221	-1,442143
ID1083	SPS1		0,713669	1,220812	0,967862	0,944558	0,626987	1,008567	-1,311742	-1,417263	-1,180040	-0,685910	-0,626138	-0,432839
ID1571			0,171864	0,752648	0,171480	1,514389	1,281079	1,147936	-1,295883	-1,411510	-0,952450	-0,544754	-0,427182	-0,543806
ID1505			0,681706	1,042354	0,985919	1,001600	0,823279	0,627370	-1,898638	-1,410496	-0,793911	-0,077094	-0,607998	-0,393364
ID574	HS90A	HS90B	0,705849	1,546983	0,802266	0,812340	0,567688	0,104656	-1,469146	-1,409309	-0,981515	-0,155464	-0,463414	-0,099798
ID956	SNX6	HNRH1	0,410388	0,863024	0,537451	1,221748	1,161872	1,115474	-1,321135	-1,402771	-1,133472	-1,043375	-0,512594	-0,157454
ID408351	SHLB2	AL9A1	0,360133	0,750083	0,486143	1,104932	0,755928	1,393015	-1,364993	-1,402236	-0,835881	-0,240630	-0,786771	-0,279879
ID960	GSHB		0,595033	1,106384	0,857891	0,782259	0,905873	1,098722	-1,574625	-1,392294	-1,174895	-0,717186	-0,408530	-0,257929
ID1168	CNN3		0,048243	0,507649	0,218965	1,883351	1,187684	0,887557	-0,863934	-1,392257	-1,390965	-0,437178	-0,590415	-0,167995
ID1425	1433G		0,872272	1,101224	1,261592	0,885532	0,827452	0,578039	-1,320411	-1,391482	-1,005976	-0,610824	-0,835845	-0,514281
ID1240	IF31	ANXA2, HDGF, TPM2	0,981907	1,264442	1,194078	0,872742	0,464824	0,685742	-1,329738	-1,391396	-1,050267	-0,430816	-0,648024	-0,721199
ID21718	HS90A		0,474253	0,716728	0,488029	1,525784	0,671262	0,675858	-1,266016	-1,380281	-1,496653	0,122713	-0,230373	-0,270626
ID1449	1433B	1433G, TIF1B	0,946532	1,109824	1,251995	0,536748	0,614169	1,064472	-1,209438	-1,377428	-0,509046	-0,658608	-0,884537	-1,049336
ID1321	OTUB1		0,918455	1,224811	1,297707	0,510122	0,938980	-0,279991	-1,300835	-1,375188	-0,783220	-0,295786	-0,296220	-0,632781
ID1300	IPYR		0,399281	0,949485	0,516783	0,841377	0,711401	1,500168	-1,921647	-1,368231	-0,677411	-0,764025	-0,277994	-0,100194
ID395	LPPRC		0,480377	0,681538	0,660868	1,276794	1,229224	1,058927	-0,709279	-1,367795	-0,965692	-0,149620	-1,234397	-0,998351

Table S-4: cluster 7

ID)1	Protein)2		co_0 min	co_30 min	co_60 min	co_24 h	co_48 h	co_72 h	PI_0 min	PI_30 min	PI_60 min	PI_24 h	PI_48 h	PI_72 h
ID1030	PP1R7	IF4A1	0,630265	0,926245	0,794258	0,903414	1,116783	1,054256	-1,484200	-1,358641	-1,220530	-0,772142	-0,396565	-0,386177
ID643	FKB10	DC1I2	0,714990	1,437985	0,817208	0,946789	0,434523	0,726984	-1,356224	-1,357544	-1,223833	0,156207	-0,558181	-0,699853
ID177384	PR56A	TXND5	0,660731	1,176653	1,117196	1,008724	0,835972	0,441342	-1,104616	-1,345514	-1,406025	-0,234917	-0,557073	-0,651202
ID1438	1433S		0,332834	0,629829	0,434521	1,287889	1,312445	1,277428	-1,516540	-1,344526	-0,832241	-0,456996	-0,594350	-0,644543
ID1709			0,420424	0,771759	0,292764	1,312541	1,221561	1,277301	-1,416946	-1,342507	-0,729382	-0,382282	-0,823394	-0,697409
ID1641	COF1		0,792181	0,853551	0,712947	1,114161	0,997202	1,022810	-1,375781	-1,342169	-0,717526	-0,380057	-1,001731	-0,770603
ID83022			0,990778	1,143249	1,271976	0,473423	0,506057	1,025734	-0,975610	-1,340858	-0,690288	-0,572505	-0,982456	-0,992626
ID15652	ENOG		0,236551	0,625219	0,528796	0,983870	1,091307	1,179281	-1,863743	-1,332877	-1,320372	-0,169967	0,033201	-0,033759
ID518	ACTN4	ERO1A	0,698424	0,821214	0,852061	1,268140	1,191806	0,734137	-0,976948	-1,329658	-1,107674	-1,169283	-0,828008	-0,446532
ID1140	PSD13		1,042932	0,853816	1,543177	0,476588	0,668751	0,847727	-0,419176	-1,322695	-0,949237	-1,146037	-1,105196	-0,777161
ID1291	IPYR	GBB1, GBB2	0,503708	0,957403	0,520901	1,345844	0,934988	0,715800	-1,957815	-1,318690	-0,871017	-0,014513	-0,585243	-0,234993
ID2944561			0,745801	1,168888	0,413288	1,137240	0,234901	-0,794058	-1,359235	-1,315657	-0,512724	0,368551	0,640770	-0,635628
ID1204	CAZA1	NPM	0,133029	0,915468	0,590962	1,798237	0,628452	0,951012	-1,044875	-1,313270	-1,167323	-0,225476	-0,957305	-0,365279
ID1070	ACTB		0,859210	1,029842	1,260074	1,403655	0,026037	0,059429	-1,222364	-1,313165	-0,894087	-0,697453	-0,248702	-0,436839
ID3762259			0,830580	0,679926	0,646677	1,039069	0,983321	0,733567	-1,684704	-1,310388	-1,277963	0,388323	-0,564155	-0,367173
ID507	ACTN4	HS105	0,834549	0,832774	0,959275	1,188260	1,211560	0,572606	-0,995744	-1,304174	-1,069723	-1,157231	-0,835627	-0,525833
ID1962730	LEG1		0,155300	0,711952	0,480398	0,992216	1,240642	0,711006	-1,180489	-1,303802	-1,177407	-1,436548	-0,543329	0,990925
ID1378	CLIC1		0,701203	0,917069	1,000174	0,995244	0,844781	0,728771	-1,954147	-1,302321	-0,977520	-0,228069	-0,564541	-0,217661
ID1231	HDGF		0,954990	1,145871	0,713828	1,137481	0,429601	0,329527	-1,618900	-1,295485	-1,175649	-0,627865	-0,097327	-0,053039
ID638	MSH2		0,223588	0,618508	0,272998	1,038234	0,346045	1,223210	-1,099945	-1,294628	-0,983166	-0,385667	0,633364	-0,688958
ID3150	TBB3	TBB2C	0,471498	0,794169	0,481182	1,254175	0,767744	0,773413	-1,900008	-1,280155	-1,376787	0,523567	0,007215	-0,385121
ID1457			0,415516	0,706394	0,972624	0,875712	0,910355	1,446035	-1,517528	-1,279981	-0,947543	-0,900093	-0,643531	-0,262984
ID875	TBB2C	TBB3, TBB5	0,707634	0,581108	0,356686	1,228907	1,356474	0,821144	-1,622612	-1,276776	-0,918997	-0,930073	-0,162378	-0,373637
ID201269	UQCR1	HNRPK, RUVB2	1,036706	0,892535	1,198351	0,673065	0,679899	0,610417	-1,537333	-1,261227	-1,193691	-0,267144	-0,517695	-0,380669
ID2838327			0,578486	1,479355	0,700211	1,054744	0,509886	-0,044534	-1,285120	-1,260883	-1,050915	-0,114690	-0,427444	-0,167769
ID300154			0,530294	1,411869	0,478112	1,183898	1,198329	0,520331	-1,204605	-1,260771	-1,054766	-0,720622	-0,632432	-0,629793
ID2382844	PPM1G		0,631204	0,503867	0,678005	1,100176	1,323879	0,981071	-1,207914	-1,256171	-0,899655	-0,062802	-0,484923	-1,322436
ID1223			0,908389	0,994342	0,893918	0,783569	-0,338900	0,403335	-1,050051	-1,227112	-1,360075	0,751165	-1,072425	0,501636
ID3597623			0,137759	0,644878	0,638069	1,183860	0,816688	0,639522	-1,982044	-1,197787	-1,144830	0,491532	-0,516352	0,411589
ID1735			-0,169483	0,386651	1,044190	0,905869	1,393605	0,628696	-1,394363	-1,196609	-1,326548	-0,935261	0,032897	0,396541
ID409	MATR3		0,293290	0,690198	0,448186	1,033655	0,962531	1,548516	-0,754012	-1,195712	-1,231729	0,474060	-1,016761	-1,133707
ID3762560			0,876715	1,096740	0,865051	1,197829	0,788878	0,294520	-0,371203	-1,183300	-0,976732	-0,200178	-1,669685	-0,768679
ID1516	GSTP1		0,433259	0,831234	0,773702	0,922838	0,965002	1,311901	-1,919695	-1,179452	-0,870008	-0,609505	-0,556230	-0,255424
ID1403	1433E		0,855320	1,026177	1,175679	0,620846	0,775629	1,115995	-1,049192	-1,177895	-0,511947	-0,944336	-1,077337	-1,045024
ID2723			0,730716	1,339014	1,074830	0,845970	0,547221	0,560220	-1,958155	-1,176270	-0,850255	-0,294627	-0,712773	-0,179547
ID1703	HSB11		0,166985	0,868734	0,547181	1,235401	0,826666	0,589216	-2,043954	-1,167377	-0,965775	0,230592	-0,329921	0,099900
ID1351	ANXA3	CAPZB, IPYR	0,735185	1,086186	1,000412	1,127337	1,027293	0,701077	-1,210231	-1,160229	-1,093428	-1,049402	-0,873122	-0,553428
ID1001	ENOG		0,420184	0,820743	0,628295	0,959232	1,161099	1,290815	-1,703034	-1,155745	-0,943120	-1,063128	-0,393526	-0,287596
ID1121	SPSY	ARSA1	0,872182	0,929789	1,321113	0,888462	0,842009	0,652447	-1,632613	-1,149680	-0,832968	-0,953669	-0,756873	-0,418615
ID1233			0,917858	1,297584	0,851944	1,361447	0,612908	0,054542	-1,138106	-1,143688	-0,631506	0,046775	-1,122849	-1,095216
ID959	KAP0	TXND5	0,986582	1,379051	1,537371	0,445136	0,383416	0,366675	-0,606174	-1,139793	-0,625448	-0,877790	-0,981255	-1,087219
ID1281			0,655202	0,883699	0,594658	0,742389	0,170788	0,854152	0,397524	-1,130636	-2,101630	0,148412	-0,444632	-0,732822
ID777	LKHA4		0,776295	0,652675	1,385246	0,735656	0,614273	0,832084	-1,776791	-1,129540	-1,256116	0,127489	-0,231901	-0,697499
ID1495	GRB2		0,173809	0,988286	0,378281	1,802545	0,880035	0,481831	-1,629723	-1,129509	-0,510179	0,274906	-0,738796	-0,902760
ID1308	LZTL1	SFRS1, SPEE	0,787997	1,036546	0,857400	1,479394	0,703764	0,605238	-1,585743	-1,121027	-0,893628	-0,641395	-0,826689	-0,562206
ID4596	GANAB		0,525286	0,801116	0,109905	1,173325	0,985526	1,321381	-1,607320	-1,116980	-1,319601	-0,518480	-0,464263	-0,019514
ID306795	SET	HDGF	0,548646	0,856676	0,787185	0,961714	1,288352	0,489800	-1,347174	-1,101208	-0,993312	-1,629628	0,040649	-0,309105
ID955	PSMD5	SH3G1	0,513454	0,863044	0,583833	1,244629	1,141611	0,881659	-1,651125	-1,096621	-0,007284	-0,985708	-0,661953	-1,071965
ID1316	PP4C	EF1D	0,859238	1,912981	0,696257	0,234587	0,690649	0,160225	-0,699432	-1,092325	-0,961739	0,191593	-1,024010	-0,920126
ID942	DCTN2	PABP2	0,684428	1,308093	1,078625	0,805108	0,772706	0,931667	-0,955515	-1,081735	-1,199374	-0,453896	-1,032591	-0,970990
ID137923			0,784597	0,418916	0,311076	0,031629	0,387031	1,866654	-0,450184	-1,066515	-1,604230	0,457248	-0,500089	-0,521821
ID1401	CPNS1	GDIR	0,805812	1,165754	1,021372	0,989827	0,739869	0,871743	-1,488503	-1,056812	-0,456614	-0,602739	-1,148962	-0,991433
ID1479	CI032		0,269605	0,419802	0,852381	1,008474	1,149470	1,186972	-1,908427	-1,056789	-0,994786	-0,356537	-0,772661	0,113362
ID1446	RANG	PSA3	0,676570	1,137508	0,693911	1,054936	0,909413	1,184596	-1,348284	-1,049857	-0,640118	-0,896714	-1,047291	-0,898848
ID1022	M6PBP	IF35, HNRPF	0,439597	0,737466	0,933654	1,376174	1,059575	0,930444	-1,541882	-1,038444	-0,806205	-0,409671	-1,056138	-0,726988
ID1195			0,856455	1,146402	1,367857	1,309097	0,389678	-0,483040	-0,459562	-1,035851	-1,514588	-0,401748	-0,938508	-0,336631
ID2031934			0,363758	0,976740	0,490764	0,607710	0,892371	1,449185	-1,614779	-1,018251	-1,049913	-0,252713	-0,824580	-0,083470
ID1575			0,161306	0,609731	0,514382	1,263744	0,241001	-0,548963	-1,165979	-1,015595	-0,422481	0,853864	0,286680	-0,564226
ID1458	IDI1	PSB10, EXOS4	0,570710	0,911426	0,960480	0,496778	1,170369	1,278319	-1,505936	-1,004080	-1,010784	-0,547814	-0,660565	-0,795858

Table S-4: cluster 7

ID[)1]	Protein[)2]		co_0 min	co_30 min	co_60 min	co_24 h	co_48 h	co_72 h	PI_0 min	PI_30 min	PI_60 min	PI_24 h	PI_48 h	PI_72 h
ID1396	PSME3	GSTO1	0,250385	1,086590	0,816521	0,679953	0,989131	1,113645	-2,114869	-0,999765	-0,874763	-0,773979	-0,402036	0,035691
ID296837			0,219539	0,690997	0,965621	0,534652	0,406520	1,132395	-1,187390	-0,996942	-1,039007	0,495651	-0,875614	-0,222508
ID1488			0,079917	0,586662	0,554260	1,222581	0,683979	0,955063	-1,947054	-0,990390	-1,196783	-0,747430	0,187881	0,424456
ID169714	AIBP	CI032	0,786260	0,793888	1,234500	0,559409	1,060526	0,999436	-1,646350	-0,985984	-0,929390	-0,725855	-0,894262	-0,433643
ID3296127	GMFB		0,638681	1,461468	1,125269	0,138809	-0,184209	0,600485	-1,156454	-0,985715	-0,748460	-2,014700	-0,272813	0,893964
ID3111980	TBB2C		1,138625	1,194283	0,966624	0,771578	0,657216	0,859498	-1,511567	-0,980576	-1,027745	-0,610266	-0,765424	-0,844812
ID3068832			-0,015033	1,126307	1,025228	1,315749	-0,093936	0,632160	-0,990516	-0,971586	-1,035676	-0,232834	-0,504944	-0,313128
ID1697	IF5A1		0,848347	1,270024	1,146201	1,091211	0,710967	0,410645	-1,496390	-0,965848	-0,862170	-1,062667	-0,957686	-0,398302
ID167310			0,704177	1,312766	0,903796	1,158227	0,900611	0,473526	-1,607333	-0,935137	-0,595754	-0,957735	-0,892208	-0,704370
ID110384			0,788058	1,126071	0,940075	1,476438	0,397574	-0,370332	-0,648201	-0,929934	-0,588954	-0,341589	-0,401484	-1,533118
ID2001699	CPNS1		0,318280	0,530515	1,052886	0,648193	0,451389	1,538943	-1,612552	-0,920024	-1,392564	-0,445354	-0,424698	0,143647
ID129210			0,400237	0,895921	0,179583	0,964080	0,959327	0,865202	-1,583519	-0,907343	-1,403508	1,035887	-0,137107	-1,009789
ID1138			0,958722	0,954611	1,830480	0,408732	0,292580	0,154749	-1,805869	-0,904838	-0,924115	-0,687332	-0,387662	-0,061891
ID1169	DJB11	ROAA	0,484653	1,018632	1,136189	1,310380	0,773651	0,500767	-1,142587	-0,890153	-1,339169	-0,196428	-1,046127	-0,708023
ID1497	PRDX4		0,455505	0,819550	0,800975	0,543607	0,486816	0,614206	-2,522994	-0,874155	-1,068920	0,225718	0,179381	0,396742
ID2304779			-0,043893	0,136748	0,666244	1,304924	0,947253	0,308304	-1,633069	-0,857480	-0,055400	-0,162103	0,288641	-0,940695
ID1545			0,288966	0,587678	0,313395	0,981333	0,777544	0,701019	-0,727722	-0,849211	-0,355504	0,308713	-0,281560	-1,667473
ID178566			1,226079	1,451093	1,245827	0,424864	0,282635	-0,259418	-1,174125	-0,841687	-0,818614	-1,090213	-0,419049	-0,299944
ID1506			0,682185	1,153230	0,944344	1,133782	0,573920	0,304716	-2,063530	-0,827627	-0,599199	-1,165662	-0,554485	0,126911
ID1296	PPP6	PGP, CAZA2	1,129462	1,042558	1,478936	0,477795	0,514002	0,754204	-1,219094	-0,771179	-0,638484	-0,810453	-1,256937	-0,903423
ID876	TBAK		1,161065	1,108755	1,236093	0,721632	0,658240	0,724326	-1,204533	-0,749454	-1,058832	-0,677595	-1,067477	-1,021620
ID1386	ALBU		0,766090	1,093510	1,164468	0,997640	0,860493	0,634781	-1,602475	-0,745004	-0,738696	-0,926571	-0,771471	-0,964407
ID1249	PPP6		0,295277	1,646407	1,073879	0,335957	0,127417	0,581054	-1,756682	-0,741076	-0,909891	-0,244408	-0,560357	0,091320
ID2030105			0,342517	1,200913	0,420787	0,955347	1,063516	0,932534	-1,740036	-0,704089	-1,090831	-0,196137	-1,026062	-0,207493
ID1057			0,292406	0,845701	0,668650	0,320530	0,127911	0,951578	-1,306892	-0,698539	-1,512227	-0,297033	0,921863	-0,388205
ID3711681			0,393701	0,886694	0,413526	2,237851	0,993546	-0,658526	-0,572857	-0,675221	-0,980233	-0,432003	-1,191682	-0,522796
ID17458	TCPA	CPNE2	0,572664	1,007025	0,845805	1,155214	0,935325	0,293017	-1,533505	-0,673742	-0,549506	0,154098	-0,341737	-1,826134
ID1007			-0,034988	0,835926	0,436845	1,790684	1,010699	0,263483	-0,969405	-0,631784	-1,493426	-0,055175	-0,823361	-0,343292
ID1006			0,061495	1,006828	0,904747	1,204832	0,866008	0,030036	-0,836051	-0,624470	-1,236616	-0,486593	-0,343407	-0,668458
ID17391	PAK2		0,043609	0,179084	0,746877	1,129213	1,100052	-0,526543	-1,009900	-0,566497	-0,393200	-0,322503	-0,061059	-0,399759
ID1526	UBE2K	RPE, CBX5, GSTP1	0,552664	0,726505	0,770851	0,987724	0,613896	0,801215	-2,281455	-0,536179	-0,430820	0,598250	-0,674042	-0,979047
ID371745	COR1B	PLST	0,855291	0,580312	1,077552	0,041166	-0,527375	1,204666	-1,934622	-0,521748	-0,922554	-0,906866	0,970702	-0,143242
ID158651			0,879209	1,134480	0,924621	0,665062	0,892267	0,261396	-0,632759	-0,519082	-1,539497	-0,718405	-0,547741	-0,979153
ID844	PPP5	RHG01	0,456013	0,758649	0,947290	0,736003	0,300297	0,319382	-1,263791	-0,497248	-0,738020	0,154515	-0,689591	-0,444870
ID3395583			0,207031	0,019664	0,459020	0,788903	1,357422	0,376805	-1,412978	-0,478693	-1,068225	1,020748	-0,850459	-0,164049
ID829			-0,184064	0,655026	0,799922	0,074728	-0,173310	1,211333	-1,636140	-0,467173	-0,677059	-0,444902	1,032436	-0,302021
ID1130			0,549760	0,440830	1,603315	0,508175	0,716474	-0,255407	-0,283644	-0,458882	-1,412723	-1,103883	-0,474192	-0,105796
ID421481			1,222874	0,752967	1,876617	-0,824346	0,390736	0,861341	-0,971275	-0,416255	-0,729908	-0,575946	-0,859384	-0,871408
ID2019749			0,584401	0,722622	0,724147	0,699542	0,664836	0,264683	-2,024043	-0,376996	-1,691140	0,478290	0,389621	-0,316390
ID1052	SET	ADRM1, MYL3	0,987818	1,538866	1,098522	0,326743	0,466006	0,269192	-1,048864	-0,286931	-1,073257	-1,224536	-0,721351	-0,638343
ID221306			-0,043605	1,068324	0,836477	0,718102	0,613717	0,436684	-0,940820	-0,205232	-1,361160	0,984965	-0,881719	-0,979491
ID2003479	AN32A		0,564800	0,710597	0,715973	0,540314	1,151373	-0,313346	-2,164612	-0,201734	-0,624876	0,739745	-0,495219	-0,438080
ID3763726			0,370185	0,564617	0,594277	0,978055	1,005211	0,576056	-1,952831	-0,015523	-0,082042	-0,613527	-0,025475	-1,552385
ID92002	SET	SPSY, HSP7C, RSSA	0,724245	1,491220	0,993944	0,267507	0,862193	-0,044036	-2,007865	-0,012659	-1,177182	-0,546326	-0,086270	-0,601352

Table S-4: cluster 8

Cluster 8			Z-score)3											
**ID)1**	**Protein)2**		**co_0 min**	**co_30 min**	**co_60 min**	**co_24 h**	**co_48 h**	**co_72 h**	**PI_0 min**	**PI_30 min**	**PI_60 min**	**PI_24 h**	**PI_48 h**	**PI_72 h**
ID610	EFG1		-0,029935	0,108801	0,033984	1,500188	1,175257	1,185965	-0,510628	-0,968351	-0,850104	-2,858073	-0,196024	-0,020116
ID352			0,695941	0,583865	0,428216	0,888344	0,885530	0,921341	-0,086414	-0,937436	-0,645562	-2,769655	-0,445094	-0,211489
ID2083630			0,357327	0,533213	0,435209	0,884278	0,963232	1,268485	-0,169661	-0,656479	-0,489788	-2,722218	-0,395032	-0,689119
ID2072345			0,222592	0,326335	0,224143	0,836998	0,756584	1,530892	0,007117	-0,797118	-0,652741	-2,663314	-0,159203	-0,298113
ID2081733			0,331826	0,447741	0,389042	0,859900	0,889998	1,471298	-0,027940	-0,754724	-0,423645	-2,656278	-0,440855	-0,750431
ID349			0,216482	0,439768	0,297166	0,972028	0,858517	1,328814	0,061414	-0,720097	-0,617172	-2,611886	-0,389424	-0,488584
ID584	GELS	MX1, HS90A	0,108919	0,535924	0,341208	0,939251	1,159421	1,457379	-0,599026	-0,577056	-0,623625	-2,602270	-0,285012	-0,505680
ID351			0,533847	0,550971	0,347574	0,971801	0,989479	1,201803	-0,121880	-0,792819	-0,648743	-2,596320	-0,775632	-0,309160
ID508072	SODC		0,379676	0,553569	0,331171	0,755282	0,612954	0,726363	-0,611445	-0,337398	-0,235587	-2,545740	-0,371668	-0,530047
ID354	SF3B2		0,329550	0,536025	0,505805	1,096202	0,914769	1,171158	-0,476664	-0,998196	-0,928133	-2,519254	-0,110849	-0,150229
ID460138	GANAB		0,308182	0,570639	0,388766	0,999550	1,046960	1,367759	0,009579	-0,646268	-0,864041	-2,502857	-0,794557	-0,509425
ID1175			0,488482	1,163011	0,846315	0,995879	0,585339	0,826619	-0,735695	-0,360985	-0,766024	-2,387844	-0,736305	-0,515753
ID242682			0,284643	0,643446	0,391419	1,164674	0,990941	1,198426	-0,211155	-0,934079	-0,755455	-2,379330	-0,514707	-0,473654
ID240305			0,392240	0,683715	0,413592	1,235212	1,033443	1,078813	-0,291330	-0,915831	-0,783582	-2,366730	-0,589151	-0,482073
ID2042668			0,174380	0,350313	-0,035329	0,525941	0,668058	1,861282	-0,240103	-0,706590	-0,200525	-2,344656	-0,193483	-0,445454
ID1511	PARK7		0,431869	0,701888	0,548798	0,756158	0,697122	0,739903	-0,946713	-0,963054	-0,669325	-2,248547	-0,096546	-0,075828
ID3261240			-0,229031	0,289135	0,113731	0,892182	1,242248	1,567371	-0,535471	-0,361954	-0,260172	-2,242306	-0,430307	-0,606002
ID339			0,286982	0,675852	0,352117	1,003927	1,394390	0,713947	0,002502	-0,414798	-0,888495	-2,225046	-0,554522	-0,903118
ID355	SF3B2		0,435750	0,718846	0,641971	1,226683	1,017301	1,065827	-0,558159	-1,076574	-0,943917	-2,219966	-0,363354	-0,499399
ID888	TCPB	RUVB1	0,620835	1,043889	0,752012	1,035976	0,738231	0,574368	-0,775628	-0,896950	-0,755523	-2,211508	-0,455009	-0,223570
ID2221628			0,416742	0,630554	0,530841	0,915504	0,949784	0,991984	-0,647234	-0,632908	-0,523709	-2,178740	-0,209246	-0,788256
ID240147			0,435209	0,684898	0,395819	1,217251	0,939918	1,254172	-0,239064	-0,898013	-0,788215	-2,160083	-0,813324	-0,568589
ID2085530			0,254323	0,420134	0,374360	0,955482	0,836620	1,485026	-0,061949	-1,305942	-0,534978	-2,137177	-0,133125	-0,687067
ID760	TCPG		0,395289	0,964069	0,553602	1,271556	0,844478	0,810017	-0,791215	-0,826593	-0,880887	-2,061465	-0,485218	-0,308998
ID585	MD1L1	GELS	0,273043	0,753390	0,584148	1,312731	1,276017	1,045104	-0,804600	-0,833422	-0,841353	-2,060776	-0,616022	-0,603452
ID371			0,298760	0,180884	0,250149	1,145010	1,008382	1,461813	-0,225172	-0,877478	-0,723159	-2,059485	-0,127525	-0,847050
ID2040831			0,145524	0,475463	-0,106150	0,826671	0,729054	1,483488	-0,277715	-1,318032	-0,267729	-2,026861	0,308017	-0,478445
ID908	SYHC		0,469766	0,988585	0,748156	1,178118	0,981297	0,915938	-0,806784	-0,330611	-0,557581	-2,023354	-1,300981	-0,768387
ID1481			0,191338	0,493000	0,266254	0,929366	0,676390	0,957908	-0,851676	-1,837148	-0,321525	-1,988349	0,601169	0,386185
ID114983			0,256204	1,143798	0,119380	0,827347	0,231606	1,298814	-1,447354	-0,328885	-0,312034	-1,970174	-0,175144	-0,136102
ID350			0,294856	0,666799	0,287776	1,068373	1,128801	1,528021	-0,261832	-1,180142	-0,933897	-1,965739	-0,751489	-0,372960
ID479	TIF1B	HNRPU	0,414180	0,447553	0,399349	1,364024	1,325004	0,886416	-0,829297	-1,184036	-1,006669	-1,954605	-0,265604	-0,084967
ID457	HNRL2		0,383301	0,376652	0,377085	0,916834	1,320028	1,414149	0,196207	-1,218100	-1,136287	-1,948261	-0,612462	-0,556210
ID840	TBA1B	TBAK, TBA3, VIME	1,316597	0,897181	0,616765	-0,007913	1,331824	0,440339	0,167204	-0,742545	-1,084315	-1,928440	-0,838017	-0,650789
ID5909			0,154414	0,127491	0,355921	0,876085	1,191744	1,697564	0,241909	-1,346774	-1,031018	-1,909380	-0,472807	-0,362495
ID51803			0,197229	0,515290	0,119463	0,894666	1,239447	1,137208	-0,796598	-1,603931	-0,897093	-1,903683	0,114507	0,507574
ID440			0,440641	0,417281	0,352281	0,989250	1,075801	1,224040	-0,732321	-0,755794	-1,359460	-1,897169	-0,214769	-0,014072
ID388			0,422338	0,553488	0,409664	0,939087	1,063488	1,384194	-0,046101	-0,713090	-1,097926	-1,894598	-0,756478	-0,737715
ID1586	RBM8A		-0,275552	0,812449	0,120262	1,341455	1,191143	1,439821	-0,202014	-0,431188	-0,900353	-1,852725	-0,658425	-1,048054
ID411894			0,036657	1,041249	0,376920	1,128533	0,120689	1,285999	0,413036	-1,141945	-1,216445	-1,852661	-0,704511	0,049313
ID584346	PRDX3	HSPB1	0,493077	0,666300	0,618728	0,567673	0,638932	0,762455	-1,405874	-0,956409	-0,655934	-1,847641	0,052012	0,142860
ID2308652			0,419935	0,289815	0,557853	1,044613	1,564227	1,151648	-0,208563	-0,780529	-0,832983	-1,815629	-0,808500	-1,035795
ID313877			0,255977	0,250312	0,110183	0,815213	1,154185	1,426074	-0,863624	-1,174217	-0,970237	-1,787665	0,035398	0,301485
ID356			0,508058	0,797209	0,412454	1,097823	1,210246	1,349619	-0,580855	-0,980466	-0,944968	-1,752299	-0,965284	-0,589612
ID13335	DPOD2		-0,409059	-0,441970	-0,499844	0,989758	1,554702	1,841358	-0,377871	-0,660732	0,094214	-1,744786	-0,422086	-0,359880
ID458	HNRL2		0,504191	0,537110	0,415044	1,090850	1,239605	1,248872	-0,015022	-1,443895	-1,194259	-1,731691	-0,532757	-0,550970
ID37898			0,640412	0,712498	0,628228	0,990896	0,932718	0,875145	-0,945467	-0,874016	-1,356161	-1,715797	-0,188262	-0,129144
ID3130260			0,289140	0,738698	0,247568	1,454197	1,350532	1,028194	-0,444653	-0,890073	-0,837516	-1,709275	-1,072118	-0,582013
ID80360			-0,232350	0,390501	0,880543	0,655071	1,045052	1,189198	-0,876233	-0,555924	-0,337314	-1,672967	-0,675254	-0,228563
ID2105992			0,582738	0,670454	0,489859	1,004528	0,925067	1,303714	-0,175865	-0,823938	-0,786158	-1,667733	-1,095945	-0,843656
ID2906459	SET		0,587792	1,053920	1,181457	0,976898	0,509058	0,538802	-0,894622	-0,431469	-1,082585	-1,645020	-0,529687	-0,675800
ID309			0,290112	0,613938	0,321694	1,242160	1,335038	1,282958	-0,447689	-1,415603	-0,894696	-1,631640	-0,671896	-0,432288
ID8247	ACTN4		0,376440	0,522867	0,392524	1,246934	1,493530	1,094795	-0,416783	-1,319141	-1,188494	-1,620622	-0,479408	-0,507797
ID393893			0,587868	0,785838	0,606803	0,880213	0,896125	1,346027	-0,150287	-0,670922	-1,006116	-1,617137	-1,311120	-0,751577
ID488	TIF1B		0,602428	0,879891	0,668974	0,924235	1,183969	0,927525	-0,162550	-1,457597	-1,450393	-1,616903	-0,348519	-0,555285
ID1183	BIEA	TALDO	0,081477	0,823701	0,516483	1,298451	1,033412	0,858253	-0,912366	-0,209182	-0,721516	-1,614788	-0,987590	-0,570033
ID276677	PSMD4		-0,191686	0,706897	-0,060192	1,331270	1,594808	0,700737	-0,336932	-0,884683	-0,975762	-1,601103	-0,574016	-0,109615

Table S-4: cluster 8

ID)1	Protein)2		co_0 min	co_30 min	co_60 min	co_24 h	co_48 h	co_72 h	Pl_0 min	Pl_30 min	Pl_60 min	Pl_24 h	Pl_48 h	Pl_72 h
ID1491	UCHL1		0,288051	1,286182	0,368445	1,460352	0,675942	0,696822	-1,376696	-0,639049	-0,392231	-1,599602	-1,050727	-0,117389
ID1081			0,935664	0,879072	1,300729	0,613041	0,214876	1,131938	-0,640048	-0,361973	-1,035650	-1,593327	-1,047093	-0,795560
ID1913428	ELOB		-0,300182	-0,206354	-0,129656	0,851847	1,185993	1,581945	-0,568358	-0,677066	-0,558422	-1,570868	-0,023136	0,021541
ID2044505			0,276816	0,268245	0,049302	0,639937	0,879928	1,792113	-0,527505	-0,971103	-0,555227	-1,566187	-0,233959	-0,443906
ID478	UBA1	TIF1B	0,571239	0,714024	0,620434	0,948849	1,248902	1,253194	-0,799543	-1,373498	-1,228123	-1,561052	-0,447043	-0,337647
ID835	TBA1B	TBAK, TBA3, IFIT3	0,819179	0,613713	0,335175	0,664581	2,095913	-0,039621	-0,615671	-0,872913	-0,392092	-1,550750	-0,436795	-1,008406
ID885	SYWC		0,552775	0,688660	0,719280	0,758106	0,976833	1,382507	-1,489518	-1,252294	-0,876138	-1,546849	-0,437578	0,137503
ID64437	EF1B		0,475473	1,158563	0,338168	1,219152	1,040437	0,812263	-0,726204	-1,169135	-0,065771	-1,544966	-0,842147	-1,082074
ID353686			0,631174	0,885265	0,530495	0,742093	0,549341	1,249476	-1,180944	-1,270570	-0,519331	-1,543151	-0,068644	-0,390993
ID323167			0,146509	0,109664	0,444481	1,040522	1,428547	1,186225	0,420914	-0,378166	-0,994604	-1,537714	-1,046912	-1,203894
ID1522	PFD3		0,424588	0,908959	0,635754	1,222168	0,856119	0,513479	-1,126391	-0,911576	-0,945464	-1,533962	-0,323813	-0,486842
ID447	HNRPU		0,063157	0,356897	0,089480	1,164532	1,182086	1,813450	-0,525430	-1,090385	-1,258853	-1,532027	-0,301060	-0,344853
ID483			0,401484	0,646617	0,176219	1,400498	1,138787	1,303299	-0,287929	-1,402990	-1,152469	-1,530360	-0,712885	-0,362861
ID431	VINC		0,503460	0,727191	0,614557	1,131117	1,205434	1,175034	-0,243618	-1,155213	-1,181642	-1,521715	-0,787395	-0,847639
ID540509	MVP		0,364098	0,389815	0,434082	1,292535	1,466056	1,233461	-0,739583	-1,022730	-1,129424	-1,519723	-0,424679	-0,723840
ID197182	MVP	UBA1	0,552895	0,620964	0,566874	0,851529	1,213350	1,402974	-0,890930	-1,318863	-1,280774	-1,511908	-0,385892	-0,198197
ID410	DPOD2		0,544664	0,732278	0,462199	1,170225	1,089667	1,213036	-0,345561	-1,053371	-1,465030	-1,510660	-0,873665	-0,341449
ID88373	HDGF		0,675227	1,112460	0,861538	1,016175	0,743634	1,161693	-1,246495	-1,051009	-0,819084	-1,492958	-0,559607	-0,774813
ID1793947			0,185083	0,523060	0,605553	1,088761	0,756067	0,816863	-1,045341	0,074262	-0,180886	-1,488396	-0,665213	-0,852895
ID2068313			0,274080	0,253300	0,087363	1,137847	1,271966	1,664710	-0,294232	-1,440716	-1,062739	-1,485044	-0,400115	-0,377682
ID72360	MLRN		0,195809	0,664703	1,054933	1,050367	0,784857	0,277889	-1,768330	0,075883	-0,015021	-1,471627	-0,746765	-0,470603
ID918	HNRH1	CNDP2, SNX6	0,230313	0,777827	0,589018	1,283018	1,058720	1,139220	0,120318	-0,891558	-1,435563	-1,466730	-0,970854	-0,800411
ID114043			0,618004	1,153142	0,298896	0,812926	0,714737	1,279830	-1,413354	-1,103385	-0,999415	-1,458725	-0,234294	-0,033042
ID5526	HYOU1		0,546856	0,724212	0,336864	1,093148	1,330978	0,929990	-0,936908	-1,648937	-1,089141	-1,457952	-0,143690	-0,049907
ID3763666			1,158441	1,091339	1,146050	0,507029	0,650319	0,910862	-0,575609	-0,830799	-0,877595	-1,456123	-1,259299	-0,828647
ID381	DYNA		0,565490	0,587786	0,509121	0,993719	1,351996	1,285561	-0,780332	-1,435264	-1,105477	-1,456035	-0,274465	-0,606107
ID2062951			0,314053	0,464334	0,250882	1,292845	1,104505	1,526949	-0,456990	-0,955107	-0,704969	-1,445512	-0,903685	-0,848682
ID951	BLMHLA	PA2G4	0,713995	1,135547	0,987182	1,004419	0,716095	1,042235	-1,260235	-0,821740	-1,141569	-1,442187	-0,758515	-0,535776
ID29521	HNRH1	HNRH2	0,286086	0,835160	0,603626	1,312710	1,055541	1,214303	-0,265220	-0,999069	-1,304518	-1,436623	-0,924961	-0,736190
ID651	ACPH		0,679909	1,240860	0,920252	0,696760	0,828660	0,983787	-1,392961	-0,035382	-0,590821	-1,434110	-1,043981	-1,211502
ID373664			0,255053	0,647220	0,638463	1,052629	1,062960	1,194629	-0,739215	-0,361320	-0,377948	-1,432134	-1,461982	-0,836388
ID3760270	KU86		0,758584	0,773231	0,806901	1,416983	1,081459	0,685429	-0,776207	-1,198616	-1,059165	-1,417784	-0,534208	-0,891052
ID441	HNRL2		0,488887	0,523480	0,354551	1,215342	1,333460	1,184615	-0,131271	-1,540165	-1,210869	-1,413913	-0,500739	-0,656856
ID2039001			-0,164438	0,391123	-0,341831	1,140706	0,473691	1,555089	-0,221143	-1,727951	-0,325162	-1,398728	0,675753	-0,406790
ID529			0,087795	0,291710	-0,010711	1,231183	1,386554	1,433793	-0,965937	-1,372670	-0,983641	-1,397446	-0,156477	0,106485
ID142473			0,362939	0,620521	-0,141267	1,243516	1,208635	1,137628	-0,884366	-0,207401	-0,953534	-1,396582	-0,097347	-1,241887
ID1689	STMN1		0,474214	0,848951	0,366437	0,974885	1,071143	1,631224	-0,800136	-1,245109	-0,335290	-1,393226	-0,920701	-1,020699
ID1194	HDGF		0,631456	1,027803	0,729204	0,851293	0,935540	1,349727	-1,339072	-1,074012	-0,922464	-1,381607	-0,562538	-0,590731
ID1375	RFA2		0,343607	0,693564	0,684059	1,362673	1,188453	1,225097	-0,872265	-1,135768	-0,631149	-1,381303	-0,985507	-0,836785
ID3542	UBA5		-0,049013	0,334679	0,293686	1,606500	1,410718	1,072247	-0,692880	-1,091480	-0,966230	-1,363379	-0,408793	-0,486899
ID557	SRC8		0,689325	0,999286	0,676022	0,948831	1,031918	1,142104	-0,839462	-1,602976	-0,934513	-1,355008	-0,717364	-0,376916
ID1084	TADBP	PPME1	0,589525	0,784577	0,795964	0,813976	1,101804	1,201421	-0,909589	-1,736106	-0,733760	-1,353721	-0,840074	-0,052448
ID11367			-0,094993	0,410677	0,043439	0,736357	1,489962	1,771229	-0,250856	-0,437499	-0,078125	-1,347757	-1,217063	-1,362310
ID2737054			0,612389	0,577324	0,668221	0,925344	-0,141805	1,012994	0,282288	-1,314808	-0,587925	-1,339386	-0,297885	-0,731595
ID650	DC1I2		0,544076	0,831076	0,492427	1,394361	1,099498	1,123055	-0,577831	-0,766712	-0,757655	-1,338757	-1,282147	-1,096081
ID953	ENOG	RBBP7	-0,060483	0,739740	0,020794	1,759279	1,335875	1,064026	-0,463778	-0,562344	-0,585341	-1,334264	-0,897072	-1,349999
ID540	TERA		0,417371	0,577187	0,529697	1,536489	1,476236	0,642974	-0,836834	-1,324666	-1,158224	-1,328995	-0,506364	-0,357120
ID3761675			0,468643	0,598627	0,156642	1,387992	1,228046	0,681599	-0,992533	0,260293	-0,829719	-1,325745	-0,937459	-1,027822
ID23217			0,421064	0,625418	0,360208	1,412143	1,084107	1,337291	-0,844743	-1,426980	-1,089831	-1,325161	-0,568925	-0,315881
ID3348523	DCUP		0,398081	0,857437	0,691929	1,296166	0,444177	1,395197	-1,182114	-1,517188	-1,011467	-1,324397	-0,230861	-0,148057
ID629	KU86	HS90A	0,577087	0,719237	0,682173	1,077500	1,383680	0,967312	-0,776839	-0,857600	-1,060041	-1,324086	-0,646156	-1,073288
ID939	AL9A1		0,455772	0,688648	0,514566	1,110833	1,139882	1,412242	-1,374320	-1,278906	-0,779634	-1,318791	-0,531473	-0,368516
ID442	DDB1		-0,290462	-0,424843	0,306616	1,431961	1,494664	1,379656	-0,019469	-0,148382	-0,618372	-1,318504	-0,964905	-1,157585
ID5403	HYOU1		0,393545	0,543840	0,243871	1,020212	1,355245	1,067597	-0,912007	-1,701422	-1,198156	-1,318204	-0,031781	0,207709
ID432	VINC		0,149751	0,473204	0,271436	1,272419	1,353504	1,320952	-1,048708	-1,360240	-1,148423	-1,310060	-0,193084	-0,108266
ID460	MVP	SYAC, UBA1	0,610075	0,717432	0,646403	1,012267	1,186612	1,171628	-1,205922	-1,392975	-1,204021	-1,309808	-0,411673	-0,147470
ID1229			-0,152184	0,275968	-0,521535	1,974391	1,262162	0,190461	-0,058509	-0,685093	-0,366538	-1,297011	-0,601469	-0,344898
ID9875	TERA		0,573738	0,911297	0,529487	1,248258	1,159142	1,013244	-0,838805	-1,397550	-1,243686	-1,288921	-0,641984	-0,346447
ID1319	DNJC9		0,376901	1,269045	0,889724	1,276219	0,706575	0,813525	-1,354255	-0,512847	-1,148989	-1,287147	-0,657085	-0,693453

Table S-4: cluster 8

ID)1	Protein)2		co_0 min	co_30 min	co_60 min	co_24 h	co_48 h	co_72 h	Pl_0 min	Pl_30 min	Pl_60 min	Pl_24 h	Pl_48 h	Pl_72 h
ID2028269			0,127405	0,983162	0,116627	1,350944	0,647804	1,400465	-1,655956	-0,227938	-0,753518	-1,284966	-0,789634	-0,235637
ID1128	EI2BL	CAPG, ROAA	0,444777	0,893644	0,515782	1,040382	1,096439	1,262710	-1,360539	-1,444477	-0,859920	-1,281318	-0,563443	-0,064367
ID965	ARP3		0,603560	0,707871	0,683130	1,352064	1,188726	1,009372	-0,990442	-1,403139	-0,835852	-1,271016	-0,826420	-0,535607
ID475	ICAL		0,270969	0,498940	0,141484	1,336966	1,068207	1,405636	-1,030653	-1,595871	-0,975885	-1,267205	-0,233813	0,064424
ID519			0,600738	0,880660	0,400898	1,271280	0,860172	1,341592	-0,500729	-0,999459	-1,376646	-1,256310	-0,800411	-0,735862
ID970	ODO2		0,141332	0,484250	0,407888	1,571358	0,920028	1,499216	-1,189549	-1,039152	-0,690358	-1,237898	-0,843343	-0,333246
ID554833	UBE1		0,615613	0,678701	0,659260	1,262033	1,200254	0,890814	-0,803061	-1,469282	-1,309709	-1,235427	-0,436493	-0,361559
ID4923			0,005540	0,381833	-0,381534	1,349373	1,419923	1,137217	0,134407	-1,438640	-1,087720	-1,228175	-0,106538	-0,492729
ID391	HNRPU		0,381947	0,518161	0,609963	1,173902	1,409664	1,154084	-0,293887	-1,229181	-0,517539	-1,227025	-1,398453	-0,888390
ID1244	PSDE		0,425747	0,816034	0,699110	0,745484	0,594178	0,581349	-0,368177	-0,831136	-0,574151	-1,220208	-0,610595	-0,562685
ID2310	TEBP		0,643075	0,445204	1,107684	0,854595	0,567594	1,294346	-0,994736	-0,992231	-0,961878	-1,206788	-0,447902	-0,610661
ID593			-0,595937	-0,148283	-0,385206	1,255113	1,003646	1,578471	-0,491682	-0,729051	-0,178405	-1,200926	-0,016243	-0,391727
ID7894	MVP	TIF1B, MVP	0,026143	0,264708	0,243266	1,075193	1,254143	1,714447	-1,093122	-1,455296	-1,067370	-1,199614	-0,177334	0,114933
ID1078	EIF3G	ARP3, TADBP, ACTB	0,246424	0,646652	0,557777	1,817074	1,042559	0,835897	-0,697614	-0,972224	-0,559987	-1,198223	-1,356425	-0,661467
ID972	SHLB2		0,307089	0,619724	0,356951	1,118164	1,352581	1,455777	-1,097090	-1,441127	-0,795259	-1,196895	-0,491560	-0,487579
ID901	CNDP2	PEPD	0,730139	0,884580	0,878465	0,973854	1,089784	1,132794	-0,927624	-1,038541	-0,651770	-1,195415	-1,189683	-0,985437
ID311244			0,612535	0,419838	0,688508	1,062270	0,245464	1,503594	-0,775305	-1,715278	-1,140038	-1,192015	-0,001991	-0,005585
ID1374	TPM3	TPM4, TPM2, TPM3L	0,310184	0,811978	0,222804	1,233690	1,251631	1,477923	-0,651795	-1,293475	-0,985454	-1,188399	-0,958409	-0,527778
ID937	SCRN1		0,387143	0,707369	0,355752	1,112070	1,234363	1,256496	-1,432108	-1,467936	-0,990704	-1,184686	-0,301832	0,027902
ID1252	PP2AA	ACTG, ANXA2	-0,130412	0,715574	1,316118	1,473177	0,104633	1,155272	-1,296880	-0,334210	-0,347644	-1,171951	-1,269296	-0,507371
ID427	ICAL		0,302944	0,476905	-0,042490	1,291150	1,247604	1,516173	-1,060443	-1,473378	-0,926656	-1,168403	-0,128023	-0,327483
ID1936868	MRLC2		-0,237253	-0,165551	-0,179144	0,875128	0,236732	2,292996	-0,038194	-0,234047	-0,244601	-1,159231	-1,074631	-0,362012
ID909	CNDP2	HNRH1	0,637771	0,849061	0,843507	1,138774	1,209419	1,025307	-0,893336	-0,980557	-1,012284	-1,131175	-1,145638	-0,823642
ID294218			0,545426	0,796614	0,676707	1,017583	1,773520	0,101004	-1,114060	-0,770555	-0,048322	-1,126736	-1,135004	-0,997860
ID948			-0,672543	0,398691	-0,240848	1,983199	1,540190	0,847123	-0,196885	-0,813897	-1,021665	-1,117500	-0,283273	-0,701967
ID525			0,166148	0,315933	0,183525	1,297410	1,186062	1,555152	-1,033857	-1,230844	-1,217476	-1,111048	-0,152975	-0,235793
ID576672			-0,272321	-0,670218	0,123535	2,001767	1,906191	-0,170010	-0,403827	-0,741988	-0,688549	-1,105583	-0,109733	-0,145660
ID921	SEPT8	HNRH1	0,412383	0,623693	0,245026	1,198719	1,362055	1,365457	-0,628042	-1,207151	-1,130477	-1,103227	-0,920458	-0,493787
ID412768			0,280014	0,635193	0,420391	1,099218	0,739393	1,610309	-0,161029	-1,672441	-1,545596	-1,103040	-0,356035	-0,222138
ID971	HNRH1	GDIB, 2ABA	0,407302	0,649640	0,664960	1,345561	1,103313	1,274512	-1,109936	-1,266513	-1,134327	-1,093937	-0,628206	-0,485852
ID1062			0,680286	0,579410	1,196072	0,677815	0,530268	0,898151	-0,562855	0,003982	-1,990175	-1,087287	-0,528894	-0,668595
ID430	ICAL		0,715060	0,699858	0,179784	1,419376	1,115540	1,048311	-0,778792	-1,386622	-1,009353	-1,084337	-0,247775	-0,942133
ID1182			0,722101	0,154430	0,976618	1,007659	1,299050	1,304726	-0,804849	-1,052216	-1,108066	-1,081469	-1,065040	-0,623311
ID796	TCPQ	HNRPK	0,325803	0,798020	0,620678	1,081117	0,994288	1,049314	0,368494	-1,417770	-1,790258	-1,080043	-0,503567	-0,716086
ID458579	PUR4		0,374719	0,611753	0,478146	1,557782	1,219153	1,047190	-0,746227	-1,486362	-0,794639	-1,074412	-0,768691	-0,687015
ID449534			0,364768	0,680432	0,432472	1,054670	1,078978	1,394468	-0,647070	-0,932577	-0,674049	-1,064951	-1,392902	-0,560474
ID1594			0,617746	0,749909	0,594174	1,244059	1,200734	1,085282	-1,231349	-1,544448	-0,585696	-1,059162	-0,656730	-0,679311
ID1058	SUCB1	IF34, ACTB, ACTG	0,401258	0,698808	0,746733	1,450154	1,163463	0,905730	-0,874069	-1,103232	-0,281078	-1,055441	-1,263467	-1,052720
ID380			0,264195	0,230896	0,255735	1,276664	1,380718	1,344189	-0,892689	-1,556523	-1,217688	-1,055288	-0,017104	-0,276927
ID487	MVP		0,247902	0,395314	0,384228	1,027433	1,300814	1,511559	-0,962524	-1,278558	-1,580278	-1,046769	-0,330835	0,070022
ID1211	HDGF		0,613304	1,112945	0,662680	1,546346	0,722024	0,855998	-0,853785	-1,052225	-0,756414	-1,035645	-0,715793	-1,358346
ID2113599			0,169747	0,506637	0,182988	0,895504	1,122563	1,810450	-0,553945	-0,810070	-1,618075	-1,025099	-0,684213	-0,252761
ID17135	CAZA2		0,417258	1,194711	0,912746	1,365234	1,139662	0,342487	-0,834335	-0,881762	-0,680993	-1,021578	-1,096218	-1,112605
ID213992	ANXA5		0,559258	0,954889	0,650092	1,162292	1,002677	1,221014	-1,520412	-0,899463	-0,590088	-1,006980	-0,762819	-1,022205
ID938			-0,044823	0,193521	0,359234	1,683674	1,890605	0,103129	-0,744019	-0,660924	-1,108056	-1,001201	-0,304245	-0,617195
ID3670	KAP2	IF4A1	-0,653728	-0,241161	-0,668621	1,617581	1,728055	1,371855	-0,461223	-0,870892	-0,748291	-0,988970	-0,183486	-0,148362
ID1802	PROF2	TR112	0,189941	0,559608	0,417640	0,637476	1,704643	0,780178	-1,289740	-1,246753	-0,832709	-0,986231	-0,575606	0,394994
ID348			0,065872	0,171752	0,194669	1,237438	0,877999	1,615402	0,188174	-0,714234	-0,965609	-0,957916	-0,507514	-1,445513
ID528			0,347577	0,701577	0,491785	1,492836	1,330268	0,901458	-0,682337	-1,062586	-1,339707	-0,956335	-0,772981	-0,690639
ID1060	PPME1		0,495391	0,699502	1,043821	1,448461	1,013313	0,828921	-0,687795	-1,295670	-0,679957	-0,953010	-1,243013	-0,908215
ID973	TBG1	MPPB	0,198868	0,460594	0,259547	1,582925	1,067803	1,253420	-1,078369	-1,437618	-1,213687	-0,949335	-0,224702	-0,156779
ID496907	CAPG		0,343902	0,641916	0,708894	1,234859	0,985345	1,497309	-1,291077	-1,175409	-1,074704	-0,944003	-0,471895	-0,691138
ID17878			0,487464	0,545753	0,365613	1,835534	1,314468	0,077888	-1,026040	-0,563535	-0,959838	-0,939032	-0,799665	-0,573368
ID1730			-0,129158	0,216891	-0,195130	0,568867	1,105314	2,159486	-1,064615	-1,431881	-0,529447	-0,924411	-0,051986	0,044968
ID2008587			0,211412	0,520254	-0,035103	0,190853	1,615849	0,920896	-0,557361	-0,406135	-0,131265	-0,922999	-0,750008	-0,834290
ID377849	SYHC	VATB2, PDIA3, RUVB2	-0,077546	0,332694	0,112091	1,156441	1,277968	1,982130	-0,631022	-1,020027	-0,550501	-0,917585	-1,143229	-0,750810
ID56308	TPM2		0,256294	0,770882	0,378597	1,107177	1,007642	1,431337	-1,178935	-1,336841	-1,183145	-0,906786	-0,866304	0,293386
ID517	ACTN4		0,298389	0,355656	0,550035	1,263951	1,295333	1,437738	-1,031048	-1,164380	-1,359214	-0,903397	-0,710789	-0,258123
ID446	HNRPU	PPCE	0,613666	0,718811	0,537216	0,996005	0,994566	1,205964	-0,396678	-1,709364	-0,956830	-0,901356	-0,799198	-0,528141

Table S-4: cluster 8

ID)1	Protein)2		co_0 min	co_30 min	co_60 min	co_24 h	co_48 h	co_72 h	PI_0 min	PI_30 min	PI_60 min	PI_24 h	PI_48 h	PI_72 h
ID1610	TEBP		0,746089	0,224446	0,551306	0,953093	0,387915	1,827266	-0,977524	-0,905823	-0,685462	-0,900121	-0,750845	-0,695371
ID2974603			0,078594	0,629801	-0,108608	1,089182	0,999884	1,614820	-1,312059	-1,133069	-1,115882	-0,873093	0,098558	-0,186401
ID2106278			0,532574	0,694839	0,518630	1,141909	0,896575	1,016590	-0,129314	-1,747325	-0,173930	-0,871579	-1,459182	-0,637682
ID125371			-0,356310	-0,073433	-0,621225	1,232367	1,803447	0,836629	0,304702	-1,308171	-1,048377	-0,870142	0,471249	-0,588271
ID144537	NUBP1		0,598046	0,900355	0,565859	1,599105	1,019194	0,712479	-1,225038	-0,859134	-0,314132	-0,853204	-1,246222	-1,110609
ID1544			0,354117	0,600544	0,194322	1,052847	0,782319	0,981146	-0,713585	-0,941827	-0,463487	-0,848397	-0,619228	-0,590870
ID1210	TPM2		0,227555	0,963005	0,233490	1,684537	1,123214	0,393909	0,057322	-1,262744	-0,624649	-0,844312	-1,432318	-0,730087
ID85707			0,434417	0,859381	0,594219	0,925659	1,223357	1,225260	-0,773187	-1,090068	-0,667158	-0,824707	-1,006821	-1,106529
ID1596	BID	RBM8A	0,259878	0,794515	0,253711	1,721525	1,081518	0,566120	0,178271	-1,056033	-0,892635	-0,819746	-0,415466	-1,876595
ID618	KU86	HS90A	0,151320	0,326578	0,280036	1,253258	1,408255	1,433536	-0,904597	-1,487822	-1,349394	-0,790372	-0,112449	-0,405941
ID2190755			0,304723	0,482400	0,441128	1,058762	1,785973	0,821546	-0,998938	-0,646049	-0,640251	-0,790234	-1,235151	-0,781468
ID1369	TPM4		0,227588	0,603991	0,355814	1,384440	1,366123	1,298336	-0,849099	-1,494358	-1,116736	-0,774788	-0,742978	-0,452031
ID398			0,356167	0,558296	0,235092	1,111509	1,382665	1,419780	-1,029694	-1,455602	-1,294807	-0,767488	-0,319200	-0,388589
ID822	HSP7C	AINX	0,070982	0,680415	0,253378	1,456491	1,253110	0,870246	-0,167232	-1,429191	-1,706300	-0,760553	-0,660658	-0,050825
ID8503			-0,266523	-0,130752	0,140598	1,463366	1,221563	1,796391	-0,839152	-0,955209	-1,413530	-0,750444	-0,425369	-0,028551
ID520	NUCL		0,282947	0,286309	0,234609	1,159032	1,238644	1,514769	-0,945384	-1,607696	-1,296661	-0,725030	-0,076597	-0,246200
ID25932			0,581620	0,574915	0,501683	1,209853	1,101845	0,767174	-0,755009	-1,401366	-0,863858	-0,718023	-0,277508	-0,900831
ID2023391			0,622593	1,189999	0,476824	1,075897	0,727224	1,262409	-0,889116	-0,917907	-0,357968	-0,699985	-1,257876	-1,407091
ID126195	ICAL		0,325769	0,509950	0,087460	0,972248	1,140105	1,712379	-1,391612	-1,560363	-0,920038	-0,695119	-0,127303	-0,227256
ID119751			0,484811	0,736574	0,026011	1,031012	1,017214	1,669433	-1,264355	-0,784226	-1,486207	-0,688046	-0,605050	-0,309182
ID115953			-0,270426	0,756762	-1,158786	0,970237	0,774825	1,366148	-1,274630	-0,135820	0,100320	-0,678836	-0,259488	-0,360015
ID1354	C1QBP		-0,285745	0,683244	-0,430197	1,748955	1,094729	1,433577	-0,053943	-1,017334	-0,507256	-0,673147	-1,149500	-1,011672
ID328907			0,185030	0,792360	-1,302818	1,070265	0,536051	1,805408	-0,467644	-0,576140	-0,713950	-0,672291	-0,776435	-0,047908
ID405	NASP		0,164529	0,545116	0,054625	0,838123	1,127412	1,874707	-0,702378	-1,661492	-1,120262	-0,671715	0,120012	-0,736605
ID574393			0,527383	0,651847	0,419773	1,232001	1,036275	1,402078	-1,070249	-1,182343	-1,135137	-0,669525	-0,759382	-0,620102
ID524			0,099051	0,445433	0,298316	1,371374	1,172558	1,416220	-1,129406	-0,715720	-1,434155	-0,660782	-0,664712	-0,363373
ID387435	FKB10		0,132709	0,279091	0,335575	0,832993	0,713869	2,129766	-0,296093	-1,620981	-1,139659	-0,642505	-0,561567	-0,323825
ID1431			-0,448068	0,690792	0,137880	0,613658	1,114184	1,787188	-1,638816	0,337728	-0,131928	-0,627474	-1,275595	-0,716419
ID320497			0,397896	0,613398	0,178386	0,871667	1,170050	1,747136	-0,969928	-0,896244	-1,377221	-0,626506	-0,334003	-0,931258
ID1440	CAPZB		0,216656	0,899283	0,630700	0,949267	1,069443	1,462392	-1,030983	-0,949288	-0,429903	-0,577353	-1,122116	-1,262436
ID1391	1433E	CLIC1	0,046165	0,489811	0,522993	1,361240	1,128350	1,428686	-1,462361	-1,177708	-0,746726	-0,575330	-0,747619	-0,411333
ID139104			-0,241272	0,142022	0,398260	0,406216	0,790110	2,395130	-0,394071	-1,140715	-0,464890	-0,536957	-0,445415	-1,042658
ID24119			-0,779854	-0,396576	-0,455329	1,197910	1,175604	2,335582	-0,699375	-0,320377	-0,586699	-0,523468	-0,352811	-0,725475
ID2010414	BCCIP	ACTG	-0,641219	0,439088	-0,614799	0,513112	1,237499	1,619187	-0,510615	-0,488588	-0,415474	-0,514190	0,163067	-0,915618
ID797	HNRPK	HSP7C	-0,074571	0,531416	0,344178	0,834285	0,630825	1,605107	0,977650	-0,968374	-1,894782	-0,508786	-0,511189	-1,092957
ID1437			0,198971	0,714319	0,229677	1,384490	1,072796	1,293964	-1,048653	-0,984796	-0,507916	-0,477719	-0,396862	-1,597700
ID414			0,362240	0,579685	0,403184	1,232664	1,048707	1,564172	-0,552315	-1,341614	-1,425799	-0,470755	-0,886907	-0,630950
ID2555205			0,082093	0,047732	0,665699	0,668810	1,466805	1,007514	-0,392245	-1,772659	-0,515023	-0,465754	-0,255854	-0,653556
ID457100			-0,096013	0,191074	0,120297	1,349385	1,289746	1,731126	-0,397403	-1,445854	-1,050725	-0,462370	-0,780668	-0,564188
ID527			0,046955	0,156755	-0,153676	0,655661	0,888673	2,233427	-1,304383	-0,405547	-1,314935	-0,430871	-0,565713	0,085937
ID315205			0,227115	-0,234485	-0,124887	1,317685	1,718172	1,166425	-0,446481	-1,600932	-1,076631	-0,422909	-0,256945	-0,371853
ID318	GRP78		0,391332	0,462790	0,340049	0,556680	1,008081	0,588954	-0,206489	-0,223605	-1,988848	-0,390767	0,084222	-0,581464
ID523			0,450950	0,600392	0,146786	1,279210	0,753690	1,609864	-0,703695	-0,477704	-1,246040	-0,360836	-1,121685	-1,021140
ID2064822			0,286333	0,418424	0,277458	1,423711	1,199879	1,235126	-0,815490	-1,304527	-1,095308	-0,358456	-0,804897	-0,551867
ID2057395			0,223741	0,371501	-0,038222	1,217900	0,990641	1,915281	-0,973093	-1,196061	-1,292903	-0,102936	-0,597978	-0,543606
ID415	HNRPU	PPCE	0,588493	0,883102	0,559196	0,929917	0,963427	1,288345	-0,361477	-1,124051	-0,991696	-0,048157	-1,386411	-1,312727
ID2060826			0,147502	0,364909	-0,093586	1,217166	0,702410	2,028366	-0,901495	-1,102633	-1,159534	0,142296	-0,638233	-0,671594
ID3102815	PR56A	TXND5	0,116802	0,851947	0,532020	0,993326	0,909546	1,446888	-0,425384	-0,904307	-1,203067	0,305197	-1,238640	-1,308030
ID393	ITA3		0,057761	0,390252	0,286114	0,817389	1,269717	1,715709	-0,423703	-1,205763	-0,764021	0,489936	-1,374622	-1,136286

Table S-4: cluster 9

Cluster 9			Z-score[3]											
ID[1]	Protein[2]		co_0 min	co_30 min	co_60 min	co_24 h	co_48 h	co_72 h	PI_0 min	PI_30 min	PI_60 min	PI_24 h	PI_48 h	PI_72 h
ID1814025			0,203703	0,201255	0,204382	0,225913	0,210850	0,204686	0,334077	0,289999	0,263926	0,258387	-4,356112	0,157513
ID1085			0,320688	0,357658	0,318090	0,356199	0,396200	0,354456	0,258678	0,303439	0,287416	0,222944	-3,157672	0,207226
ID319			0,270639	0,270842	0,263794	0,296324	0,292839	0,286613	0,247041	0,240335	0,215910	0,246918	-2,775427	0,205903
ID2198480	PPT1		0,360100	0,363383	0,364629	0,376973	0,423782	0,376582	0,350954	0,344562	0,338607	-1,764074	-2,721816	-1,231214
ID2218268			0,399885	0,405152	0,399872	0,373349	0,436887	-0,379136	0,376898	0,381603	0,386514	0,187112	-2,642773	-1,254997
ID2201032			0,377422	0,380568	0,379068	0,396400	0,431073	0,463781	0,358485	0,340158	0,355667	0,303436	-2,610218	-2,610218
ID2302886			0,232420	0,397025	0,372845	0,775513	0,546006	0,390243	-0,206518	-0,173963	-0,006784	-0,170737	-2,583712	0,384978
ID2592168			0,404942	0,241521	0,366601	0,392213	0,389410	0,442359	#DIV/0!	0,465251	0,422266	0,359537	-2,501038	-1,622815
ID1293	ANXA3	IPYR	0,157072	0,708105	0,677622	0,742527	0,510471	0,688679	0,277921	0,234563	0,300150	-0,198917	-2,360006	-1,787917
ID2117397	VINC		0,545907	0,567223	0,534176	0,890964	0,631422	0,871009	0,017459	-0,097992	0,163835	-0,544833	-2,333220	-1,382159
ID1763758			0,227886	0,227592	0,223820	0,263757	0,249396	0,243443	0,200201	0,165176	0,185911	0,195747	-2,270912	0,136920
ID2024619			0,213453	0,493498	0,298398	1,013943	1,183497	0,442597	-0,067734	-0,419384	0,270675	-0,212424	-2,168991	-1,100635
ID3022983			0,578075	0,739627	0,653142	0,154690	0,131099	0,192007	0,687525	0,644748	0,670350	-0,436238	-2,153046	-1,971039
ID418384	SCMC1	TADBP, PPME1, ACTG	0,382015	0,655522	0,974584	-0,169563	0,672090	0,952335	0,352018	-0,361498	0,770358	-0,699598	-2,133976	-1,569187
ID2296834			0,469117	0,471661	0,465403	0,468825	-0,175615	0,453402	0,470525	0,467018	0,469463	-2,125250	-2,125250	-0,495897
ID1315	TPM1		0,213619	0,614411	0,167339	0,715703	1,089796	1,337892	0,345891	-0,173979	0,034796	-1,118065	-2,100416	-1,406503
ID1864541			0,321260	0,434698	0,493967	0,599630	0,826608	0,749165	-0,313292	0,381869	0,225017	-0,523955	-2,097170	-1,228787
ID981	K1C17		-0,021957	0,579922	0,487048	1,907608	0,564946	0,103109	0,173850	0,109633	-0,336750	-0,203323	-2,040128	-1,374789
ID1765003			0,371465	0,368444	0,368460	0,352805	0,387690	0,348764	0,361676	0,340181	0,355279	0,326353	-2,022814	-2,759584
ID447263	HNRH1	CNDP2	0,416786	0,719401	0,475497	0,115983	0,631498	0,190652	0,247273	0,993913	0,655786	-1,505993	-2,007593	-1,309701
ID1755	MYL6		-0,034976	0,770527	0,132220	1,374092	0,849919	1,013838	-0,323531	-0,122346	0,367006	-0,599545	-1,980356	-1,596734
ID1248	RLA0		0,304413	0,905049	1,002414	1,375797	0,612803	0,580570	-0,461269	-0,660549	-0,607786	0,079680	-1,965655	-1,145547
ID1086	K1C18	ACTB	0,310449	0,518882	0,172197	0,776749	0,364927	0,621085	0,964689	0,535806	0,473812	-1,679779	-1,964136	-1,514626
ID2214019			0,467441	0,485346	0,476253	0,519090	0,514827	0,496820	#DIV/0!	0,461129	0,610305	-1,182066	-1,963754	-0,187194
ID1036			0,308763	0,435132	1,400554	0,358006	0,795714	0,546957	-0,333726	-0,491778	0,086678	0,637479	-1,931930	-1,652477
ID2027812			0,503391	0,538429	0,499223	0,569985	0,569736	0,536513	-1,297984	0,487094	-0,126852	0,491284	-1,890288	-0,102191
ID2669	MLRM		0,058649	0,770543	0,445653	1,421361	0,849595	0,460689	-0,575300	0,581670	0,030886	-1,372878	-1,866131	-1,147957
ID1847793			-0,203143	0,081082	-0,243749	1,228021	1,498744	1,102139	-0,336232	0,142269	0,192422	-0,150952	-1,864717	-1,483620
ID950	SHLB2	AL9A1	0,073890	0,794612	0,789380	1,411865	0,189308	-0,425743	0,509815	0,628910	0,138867	-1,642350	-1,862904	-1,016237
ID295167	COPE		0,704084	0,986981	1,181319	0,399651	-0,069735	0,240437	0,306874	-0,128254	0,680650	-0,783900	-1,855716	-1,858366
ID1647	MLRM		0,258681	0,724780	0,671081	0,879422	0,406604	0,414916	-0,020422	0,969524	0,335032	-1,517155	-1,850370	-1,651382
ID923	HNRH1	CNDP2	0,144614	0,595815	0,538385	0,725320	0,832722	1,332252	0,457147	0,066177	-0,245261	-1,620595	-1,844333	-1,387393
ID1008	ACTG	ACTB	0,522735	0,518253	1,204789	0,894275	-0,220837	1,476111	-0,323176	-0,129466	-0,058578	-0,804896	-1,829131	-1,451303
ID1080	ACTG	K1C18, ACTB	0,998326	1,182627	1,231544	0,782324	0,356936	0,397626	-0,160554	-0,097225	-0,619992	-1,195567	-1,807075	-1,367862
ID206082			0,592306	0,470851	0,964561	0,649564	0,504336	1,119343	-0,001703	-1,468293	0,390499	-0,999668	-1,773239	-0,698475
ID496	GANAB		0,534604	0,790292	0,788530	0,432712	0,622629	1,245481	-0,123077	-0,120685	0,261905	-1,053465	-1,765175	-1,877117
ID169541			0,337529	0,639935	0,285000	1,151482	0,442730	0,168752	0,693300	0,055837	-0,123207	0,518078	-1,762614	-2,277304
ID210320			1,142546	1,423348	1,371116	0,562134	-0,096459	0,352109	-0,214188	-0,507166	-0,710479	-0,473367	-1,755008	-1,212928
ID1982157			0,925109	1,075947	0,924004	0,552258	0,310086	0,140928	0,073313	0,167662	0,366817	-1,123902	-1,743744	-1,949453
ID15367			0,396009	0,695482	0,586388	0,556458	-0,039118	0,488331	0,450651	0,529271	0,338422	-1,145258	-1,736899	-1,406050
ID1215	SEC13		0,550533	0,662426	0,769879	0,738737	-0,146813	0,286555	-0,024824	1,148507	0,564428	-1,378954	-1,733441	-1,781769
ID1325	WDR61	ANXA5	0,555475	1,067640	0,740250	0,676365	0,458307	0,371126	0,254749	0,367194	0,090537	-1,075157	-1,732255	-2,043020
ID422947	GSTO1	PSB7	0,460646	0,901167	0,854948	1,783279	0,649984	0,369617	-0,384245	-0,818573	-0,409659	-0,443381	-1,727792	-1,346835
ID1740	MYL6	RLA2	-0,165834	0,996262	0,078658	1,833408	0,743925	0,292474	-0,134953	-0,947789	-0,109887	0,682999	-1,727246	-1,371268
ID1953484	MLRN		-0,132383	-0,170514	0,274103	1,593232	0,722714	0,057266	0,020132	0,796781	0,423213	-0,643913	-1,704035	-1,397574
ID1327			0,545657	0,605883	-0,627684	0,593831	0,607890	0,584405	0,569710	0,546225	0,594006	-0,219178	-1,694063	-1,694063
ID3543802			0,534197	1,325420	1,124974	1,447755	0,137631	-0,490372	-0,005396	-0,212822	-0,583515	-0,301080	-1,679382	-1,372681
ID2215889			0,186164	0,590660	0,654421	0,907250	0,503834	1,098481	0,265393	0,023511	-0,273337	-1,288707	-1,677939	-1,311907
ID55610	TPM1	TPM2	-0,046709	0,345425	0,027928	1,076473	1,498934	1,528462	0,108872	-0,624707	-0,265986	-1,105091	-1,667863	-1,152010
ID365	HYOU1	OXRP	0,440023	1,212874	0,711141	0,845017	0,589278	0,168417	-0,769710	-0,176613	-0,156520	0,955368	-1,666250	-1,914184
ID993	GDIB	SAHH, IDH3A	0,631078	0,991643	1,362553	1,053495	0,532904	0,692762	-0,660338	-0,613143	-0,519434	-1,158296	-1,647293	-0,955507
ID1750			0,853277	0,804043	1,468983	0,113032	0,550155	0,616330	-0,078063	-0,650868	-0,085459	-0,374765	-1,646415	-1,663941
ID3046761			0,334972	0,580667	0,949622	1,035667	0,647582	-0,451947	-0,244439	0,373123	0,448212	-0,021257	-1,645968	-2,011547
ID1758			0,504389	0,982339	1,169678	0,519105	0,081309	-0,024331	-0,229617	1,387491	-0,361220	-1,269977	-1,644338	-1,432320
ID1164			0,487866	1,083450	0,774405	0,616093	0,706515	0,888860	-1,001534	-0,246085	-0,510281	0,609743	-1,640757	-1,615839
ID1260			0,323275	1,064115	1,329013	0,666802	0,387634	0,692308	0,355150	-0,156107	-0,649688	-1,391370	-1,626130	-1,342845
ID509	GANAB		0,119560	0,396455	0,275446	0,266747	0,509115	2,349272	-0,108047	-0,082827	-0,145328	-0,597948	-1,617064	-1,514868

Table S-4: cluster 9

ID[1]	Protein[2]		co_0 min	co_30 min	co_60 min	co_24 h	co_48 h	co_72 h	PI_0 min	PI_30 min	PI_60 min	PI_24 h	PI_48 h	PI_72 h
ID1603			0,402124	0,404582	0,403205	0,426998	0,441804	0,422658	0,378472	0,369489	0,383158	-0,596151	-1,615053	-2,484846
ID906	TBA1C	PTRF, TBA4A, TBB5	0,523970	0,540462	1,057750	1,176802	0,520499	0,076129	-0,095943	0,791726	-0,571726	-1,249741	-1,578102	-1,504261
ID1538			-0,133313	1,163237	-0,100537	0,734959	0,310561	0,974473	0,757656	-0,607327	0,636581	-0,598008	-1,560076	-1,727707
ID499	GANAB	IMMT	0,677694	0,819925	1,141919	0,156665	0,513788	1,272129	0,119311	-0,711659	0,160230	-1,328805	-1,554757	-1,598641
ID2021577			0,547581	0,971165	0,688553	0,782974	0,537959	0,757700	-0,848743	-0,613574	-0,197614	0,658393	-1,533655	-1,586141
ID1255	ODPB		0,279179	0,986021	0,817413	1,121324	1,065541	0,960797	-0,274518	-0,577440	-0,455887	-1,187281	-1,526673	-1,505296
ID364919			0,949040	1,362730	0,744586	0,277180	0,323844	0,533454	-0,589722	0,757429	-0,451066	-0,798031	-1,506078	-1,802874
ID13616	TPP1	HNRPF	0,080016	0,401470	0,540250	1,460397	0,736452	0,976543	0,459487	-0,363705	-0,609881	-0,409118	-1,504492	-1,869699
ID435879	DNJC9	PSME3	1,065305	1,504905	1,634960	0,426011	-0,313280	-0,818843	-0,312029	-0,080784	0,032818	-0,392457	-1,502014	-1,342707
ID1301			0,394203	0,451800	0,476191	0,531355	0,671515	0,652293	0,329971	0,267098	0,316574	-0,498969	-1,498351	-2,218423
ID1672	HSB11		-0,050644	0,678060	0,304098	1,459077	0,814983	0,082099	0,146735	-0,780040	0,816470	-0,184648	-1,495729	-1,836624
ID964	IF2B	ARP3, QCR1, PDIA3	0,640717	0,805065	0,463229	0,593003	0,873003	0,763183	0,595183	-0,169567	-0,042305	-1,950038	-1,495110	-1,563872
ID900	K2C8		0,690293	0,534133	1,051506	0,420811	-0,101614	-0,161829	1,343080	-0,099138	0,587431	-1,367408	-1,489283	-1,749835
ID16755			0,889803	1,005758	1,223077	-0,607023	-0,026970	0,341478	-0,007497	0,028769	0,895906	-0,488165	-1,488468	-1,888709
ID2087348			0,597112	0,866543	0,408678	0,999736	0,983557	0,265448	0,053356	0,106669	-0,213883	-2,217834	-1,485779	-0,918062
ID2990527			0,339455	0,349394	0,328915	0,360301	0,346651	0,315922	0,347485	0,360297	0,340899	0,136734	-1,485090	-2,044608
ID1305	SNAA	SFRS1	0,575356	0,777261	0,764633	1,089565	1,312789	0,729657	-0,627072	-0,564585	-0,105030	-1,233630	-1,480920	-1,546431
ID1186	PP1B	NAGK	0,319213	1,354673	0,978391	1,662245	0,102191	-0,219074	-0,299579	0,030600	-1,013504	-1,009815	-1,469297	-0,688498
ID3352574			1,095244	1,543132	0,943683	0,335787	0,105228	0,405011	0,084626	-0,412646	-0,663428	-1,291725	-1,464235	-1,003608
ID1762			-0,039426	1,048137	0,300496	1,068117	0,459611	0,250170	0,795348	0,252742	0,039782	-1,418778	-1,462296	-1,648598
ID29100	TADBP		0,594689	0,920987	1,040451	0,744019	1,053472	0,987125	-0,942840	-0,310152	-0,568324	-1,451313	-1,462036	-0,968905
ID1720			0,062005	0,133322	0,211002	0,258889	0,772270	1,279819	-0,461246	0,688331	0,220619	-0,606147	-1,458073	-1,252327
ID1763803			0,525635	0,567735	0,544341	0,778798	0,771745	0,687394	0,007836	-0,629727	0,107162	-1,750644	-1,455047	-0,592890
ID2292906			0,426583	0,655943	0,413271	1,687879	0,579427	-0,011192	-0,565815	-0,256637	-0,425491	0,701637	-1,455025	-1,575172
ID1156	RSSA	SET	0,771280	1,357380	0,458837	0,366789	0,431851	1,275980	-0,395708	-0,643242	-0,050374	-0,344372	-1,443710	-1,870804
ID631	IMMT	PLOD3, THOP1	0,100986	0,270395	0,457641	0,434122	0,819040	0,836758	1,077243	-0,544888	0,922860	-1,729779	-1,440198	-1,636623
ID510	GANAB	IMMT	0,768848	1,047871	1,027292	0,387616	0,396892	1,018476	-0,223002	-0,048367	-0,128408	-2,124918	-1,439443	-1,214086
ID3760549	SF3A3		0,506288	0,840578	0,852445	0,730968	0,789118	0,814732	0,591889	-0,033930	-1,285415	-1,211107	-1,428432	-1,469912
ID93790	ENOG		0,065545	0,415404	-0,022165	1,836706	1,320652	0,838954	-0,176352	-0,403937	-0,333925	-0,901239	-1,426887	-1,438065
ID1295			0,100777	0,784129	-0,363283	0,858967	0,632591	0,922519	0,170717	-0,143142	-0,001705	-0,220779	-1,422830	-1,373156
ID978	APMAP	SNX6	-0,337115	-0,136660	0,889902	0,727952	0,881764	0,393184	0,745091	-0,853591	1,156787	-0,517940	-1,420820	-1,658038
ID1589	SORCN		0,002757	0,683856	0,109926	1,231825	0,790864	0,847236	0,531499	0,360864	-0,116944	-2,127903	-1,419545	-1,426412
ID3499167			0,494639	0,495709	0,498620	0,516702	0,510425	0,532390	0,502493	0,505150	0,504001	-1,975798	-1,407710	-1,404376
ID1303			0,805777	1,370975	1,353720	0,918640	0,296386	0,022984	-1,162680	-0,402028	-0,535175	-0,915664	-1,403020	-0,578831
ID1358	UBA1	3HIDH	0,503595	0,780528	0,911156	0,796509	1,183279	1,033505	-0,675076	-0,445783	-0,064064	-1,538115	-1,388692	-1,481371
ID112515			0,327748	0,627397	0,326851	1,303470	0,682131	0,095673	0,406068	0,557750	0,006300	-1,734693	-1,381498	-1,650870
ID1098	RSSA		0,705869	1,151122	1,543160	0,859525	0,015413	-0,941148	0,002917	-0,265809	-0,526946	0,570663	-1,378655	-1,593443
ID99319	PDIA1		0,330368	1,082891	0,457787	0,455375	0,219198	-0,157043	0,110367	0,770677	0,445265	-0,201063	-1,364501	-2,199586
ID2287478	SFRS3		0,517251	0,849496	0,710980	1,058419	0,665566	0,463994	-0,231659	-0,125310	0,113258	-2,164863	-1,360927	-1,578636
ID1665			1,172847	1,021581	2,090236	-0,347214	-0,396532	-0,753586	0,024748	0,007293	-0,025817	-0,335267	-1,355559	-1,186546
ID207376	KCRB		0,378800	0,888570	1,104791	0,665975	0,169896	0,603721	0,440726	0,179414	-0,423051	-0,424205	-1,345524	-2,345163
ID3763691	GRP75		0,653061	1,005094	0,620382	0,664319	1,156113	-0,232541	-0,054019	-0,516519	-0,301897	-0,266454	-1,342439	-1,451712
ID1282	ODPB	CSN6, GBB2, GBB1	0,106345	0,441550	0,237916	0,990436	1,245931	1,181816	-0,029730	0,187670	-0,116526	-2,194984	-1,340672	-1,258500
ID1553	STAM1	PSB9	0,935391	1,423655	1,897757	-0,150326	-0,010578	-0,037746	-0,019569	-0,500708	-0,356960	-0,706307	-1,338805	-1,312381
ID904	TBA1B	TBB5	0,610056	0,541858	1,321549	0,417665	-0,007252	-0,505975	0,145263	1,384522	-1,182790	-0,894717	-1,336551	-0,717306
ID659	NDUS1		-0,047979	0,321174	-0,122100	0,477592	0,951521	1,103180	0,556569	0,617144	0,650986	-1,675225	-1,335883	-1,915786
ID709	LMNB1		0,195715	-0,509975	0,637984	1,049838	0,462621	0,993493	0,764015	0,096787	0,411212	-0,611874	-1,335321	-2,307464
ID8700	ACTN4	MVP	1,408630	1,188732	1,234259	-0,476571	-0,718109	0,287540	0,137322	0,489422	-0,099263	-1,993768	-1,325472	-0,631165
ID461679			0,666837	0,642322	1,282839	0,359737	0,637753	1,482310	-0,202423	-1,291579	-0,456035	-0,622768	-1,322035	-1,332649
ID568037			0,396007	0,628180	0,408782	0,969900	1,310265	-0,573730	0,413596	0,234598	-0,235819	-1,256387	-1,320595	-1,288893
ID191574	FKB10		0,888769	1,301301	1,158426	-0,021813	0,580403	0,789844	-0,429138	-1,348559	-0,060703	-1,296515	-1,306820	-0,579322
ID99730	RD23B	PDIA1	0,305605	0,333149	0,411375	0,159896	0,716521	0,196582	0,765430	0,429427	0,234547	0,442098	-1,303487	-2,580619
ID1026			-0,381788	0,273174	-0,247437	0,947049	0,967410	0,771192	0,342687	0,750932	0,689744	-1,074157	-1,292613	-2,014731
ID1980598			#DIV/0!	#DIV/0!	#DIV/0!	0,467425	0,472021	0,481242	0,447806	0,479672	0,466637	-0,440260	-1,291162	-1,516236
ID83942			0,990476	1,500950	1,179582	0,697699	0,328366	0,130716	-0,694862	-0,366539	-0,017199	-1,476496	-1,289594	-1,352225
ID1717			0,448004	1,058169	0,731411	0,836310	0,461475	-0,015994	-0,304318	0,597679	0,103609	-1,969962	-1,288583	-1,150291
ID791	CPNE1		0,135793	0,366669	0,282795	1,104294	0,839845	1,159380	0,170657	0,355926	0,036094	-2,252913	-1,286003	-1,475764
ID1277	SAE1	EF1D	0,915158	1,659125	0,933593	0,438775	0,221938	0,477341	0,073692	-1,087137	-0,847201	-0,172648	-1,278133	-1,377664
ID1432	NNMT		0,983396	1,026689	1,338016	0,728121	0,917160	0,413151	-1,021699	-0,533896	-0,318732	-1,050031	-1,272858	-1,471825

Table S-4: cluster 9

ID)1	Protein)2		co_0 min	co_30 min	co_60 min	co_24 h	co_48 h	co_72 h	PI_0 min	PI_30 min	PI_60 min	PI_24 h	PI_48 h	PI_72 h
ID990	IF4A1	HNRPF, ACTG	0,722227	1,225113	1,249401	0,901437	0,720870	0,254010	0,027360	-0,933506	-0,596831	-0,784233	-1,253737	-1,728170
ID516			0,298704	0,585066	-0,311377	0,906483	0,522988	1,667125	-0,488245	-0,009743	-0,888111	0,050926	-1,238284	-1,082801
ID1383			0,784441	1,403496	1,173225	1,161162	0,297692	0,128789	-0,888752	-0,672309	0,087284	-1,163108	-1,225159	-1,377536
ID1533			0,827484	0,972094	1,268743	0,218134	0,710040	0,479521	-0,896268	-0,852181	0,187798	-1,046609	-1,217943	-0,912465
ID2290995			0,522878	0,538463	0,368101	0,619171	0,390537	0,656429	0,120967	0,099659	0,065720	-0,016873	-1,208773	-2,160497
ID1286			-0,110960	0,532119	0,369239	1,095144	0,659068	0,996136	1,158700	-0,457715	-1,104074	-0,007283	-1,208650	-1,923545
ID218682			1,038052	1,185134	1,262611	0,437297	0,231632	0,490707	-0,494730	-0,963984	0,316662	-1,338406	-1,205592	-1,293985
ID436	DDB1	OXRP	0,325466	0,239047	0,791924	1,426127	1,354908	0,697229	-0,065010	-0,367078	-0,577516	-1,486253	-1,202722	-1,507686
ID62715			-0,103120	0,662704	-0,411101	0,445848	0,527279	1,621932	0,582355	0,307142	0,181390	-2,292954	-1,201331	-0,893383
ID1617			0,757593	0,939155	1,038620	0,148886	0,238291	0,184192	-0,317906	0,262834	0,449361	-2,021547	-1,200417	-0,984448
ID932	RBBP4		-0,244932	0,410281	-0,185123	1,302256	1,018093	0,949854	0,721727	0,165127	0,158405	-1,828748	-1,194641	-1,729486
ID1569	APT		0,986210	1,434757	1,542104	0,789044	0,162945	-0,001793	-1,146245	-0,586393	-0,352411	-0,725155	-1,165951	-1,118401
ID16882			-0,635608	0,164642	0,025210	0,614326	0,591339	1,783025	-0,314696	-0,106433	1,227172	-0,578160	-1,164241	-1,751116
ID1205	NAGK		-0,104182	0,659793	0,171036	2,162580	0,827735	-0,027635	0,692969	-0,344062	-0,954170	-1,495484	-1,160629	-0,801822
ID1628	SKP1		0,013364	0,876158	0,806324	0,448849	0,886685	1,118112	-0,549045	-0,684269	0,130326	0,060195	-1,145707	-1,945942
ID3763676			0,028741	0,456070	-0,095680	1,089680	0,600458	0,169662	0,254154	0,528838	0,850162	-0,743348	-1,145244	-2,179328
ID1201			-0,418857	0,698341	0,299122	2,166613	-0,055977	-0,219632	1,124736	-0,314969	-1,350784	-0,300827	-1,137780	-0,565193
ID1278	EF1D		0,685437	1,475483	0,651813	0,814635	0,484690	-0,331415	0,027838	-1,643800	-0,269304	0,989340	-1,134877	-1,502505
ID577	IMMT	PLOD3, HS90B	0,374205	1,168922	0,943155	0,520386	0,846137	0,738637	0,035196	-0,907544	0,190111	-2,086568	-1,118208	-1,226072
ID504850			0,582918	1,118519	0,821622	0,540231	1,097991	0,653662	-1,051918	-0,450580	-0,634133	-0,225648	-1,105674	-1,403401
ID1434			-0,596345	0,190391	-0,027333	-0,233597	0,521596	2,448240	-0,690201	0,816085	0,164367	-1,041014	-1,105205	-0,707237
ID1115	THOC3		0,565644	1,426208	0,799617	0,641368	0,081739	1,197143	-0,639008	-0,279715	-0,177047	-2,048025	-1,102867	-0,977064
ID1565	SFRS3		0,304614	0,538818	0,557653	0,687955	1,385823	0,833143	0,439102	-0,188363	-0,694976	-1,451825	-1,102696	-1,672205
ID1189			-0,221219	0,918269	0,093021	1,740484	0,399402	-0,854460	1,132675	0,027048	-1,581053	-0,249904	-1,093432	-0,373305
ID1576	TCTP		0,367330	1,013096	0,590196	1,448955	0,937849	0,600620	-0,059493	-0,735229	-0,470267	-1,502532	-1,093243	-1,472916
ID1435	ECHM		0,112954	0,544296	0,361106	0,824944	1,028705	1,122480	-0,307343	0,020474	0,519456	-2,298023	-1,070340	-1,433214
ID3058865	PSB6		0,797445	1,135269	0,839379	-0,030867	0,246659	0,889819	0,462419	-0,076403	-0,111896	-2,045283	-1,067545	-1,550317
ID347052			0,115150	0,404319	0,684568	0,194313	0,311578	2,007740	-1,940156	0,115611	0,169664	-0,037433	-1,061352	-0,973361
ID163898	CBX1		-0,299275	0,330837	-0,396816	1,744608	0,938052	1,065988	1,054388	-0,992144	-0,722957	-0,304773	-1,035592	-1,458509
ID3275754			0,221515	0,426128	0,404637	0,463537	0,308786	0,756654	0,823187	0,589629	0,310218	-2,147798	-1,012586	-1,680858
ID1318	CLIC4	PPIE	-0,888078	0,360356	-0,626176	1,365408	0,869439	1,104330	0,569203	0,005982	0,318323	-1,334095	-1,003472	-1,074744
ID1746	PFD5		0,631061	0,835949	0,807494	1,189296	0,790154	0,409774	-0,720842	-0,779488	-0,687151	-0,269659	-0,997355	-1,276647
ID1379			0,467678	0,614640	0,728846	0,870007	1,240174	1,203522	-0,600888	-0,440764	-0,207448	-1,573429	-0,994659	-1,701036
ID564	P3H2	KU86, HS90A	0,091835	0,143014	0,379518	1,079489	0,800649	0,748208	-0,154683	-1,107227	0,464369	-0,593559	-0,983846	-1,016157
ID1331	PP1A	DHC3	0,737412	1,319128	1,413684	0,051639	0,313029	0,400976	-1,188796	0,482333	-0,248744	-1,806391	-0,978791	-0,947077
ID607	THOP1		0,088406	0,388576	0,618796	0,396655	1,098866	1,466493	0,421328	-1,322509	0,386711	-1,525771	-0,978177	-1,420817
ID75950			0,208817	0,376611	0,157652	1,114270	0,593113	1,137179	-0,584288	-0,299050	0,168259	-1,354779	-0,961179	-0,895298
ID2214			-0,713926	-0,607805	-0,459380	1,947623	0,285170	1,018889	-0,281739	0,455383	0,532292	-0,471923	-0,948668	-0,873895
ID943	GSHB	K2C8, DDX39	0,829103	1,113689	1,430881	0,505105	0,651620	0,496188	0,210453	-0,925921	-0,799851	-1,780082	-0,943958	-1,232248
ID156308			0,108996	0,276763	0,647812	2,005320	0,936946	0,277047	-0,323226	-0,432924	0,055232	-1,981947	-0,943543	-1,121963
ID319168	OXRP		0,566579	1,200630	0,642806	1,268103	0,489378	0,070593	-0,672604	-0,893659	-0,247379	0,612610	-0,943231	-1,940673
ID2115505			0,214269	0,160426	0,702190	0,117438	-0,140075	2,268698	0,138404	-0,141886	0,011513	-1,704473	-0,925327	-1,127295
ID586			0,591024	0,687241	0,768981	0,078119	-1,557171	1,671751	-0,462963	-0,140923	0,137120	-0,022780	-0,893310	-0,862785
ID1100			0,329355	0,397339	0,476458	0,769246	0,559108	0,622537	1,241061	-0,389572	-0,835502	-2,066660	-0,866144	-0,753891
ID320752			-0,020733	0,063873	0,173332	1,445284	0,966598	0,534400	0,983176	-0,328052	-0,645662	-1,676870	-0,858111	-1,056453
ID1470			0,285600	0,029802	0,639564	0,490664	0,558469	0,227828	0,254761	0,579605	0,609243	-0,913236	-0,833614	-2,156995
ID543746			0,164444	0,090732	0,510754	1,212462	0,993346	0,592792	1,165949	-0,552305	-0,898455	-1,672482	-0,824790	-1,200568
ID1593			1,060567	1,718480	0,847065	-0,401312	-0,133705	1,053549	-0,134122	-0,862378	0,048855	-1,101501	-0,799371	-1,571502
ID1580			0,435058	1,393208	1,142566	0,577199	0,318366	-0,598879	-0,532335	-0,668645	0,465286	-0,237283	-0,780783	-1,573079
ID30544			0,022740	-0,835441	0,715173	0,865988	-0,224293	1,707015	0,431554	-0,320639	0,644536	-1,488772	-0,734927	-1,155127
ID1604	RBM8A		0,835600	1,072341	0,741295	0,647843	0,465958	0,413252	0,305503	-0,305106	-0,263363	-1,203886	-0,722818	-2,287589
ID1219			0,821260	0,973001	1,083799	1,468664	0,572071	-0,944486	-0,952913	-0,161098	-0,507445	0,017098	-0,680962	-1,684716
ID109442			0,551996	1,171522	0,499095	1,533532	-0,223247	-0,267053	-0,178787	-1,384734	-0,155796	0,472249	-0,653073	-1,247642
ID1420	HSPB1		-0,046062	1,069898	0,964007	0,596484	-0,142203	0,534524	-0,714877	0,226657	1,043778	-2,203517	-0,602628	-1,276942
ID1451			-0,016425	0,063066	0,236653	0,677229	0,636979	0,739215	-0,007295	0,575532	0,388133	-1,036735	-0,235066	-2,280468
ID1648			0,014123	0,205347	0,314770	0,609452	0,812918	0,641903	-0,361359	-0,140386	-0,155674	0,015694	0,214248	-2,167112
ID3369954	TADBP		0,153528	0,164627	0,166272	0,190939	0,297248	0,210832	0,129403	0,129355	0,157714	0,105904	#DIV/0!	-5,912055

Table S-4: cluster 10

Cluster 10			Z-score)3											
ID)1	Protein)2		co_0 min	co_30 min	co_60 min	co_24 h	co_48 h	co_72 h	PI_0 min	PI_30 min	PI_60 min	PI_24 h	PI_48 h	PI_72 h
ID1702	CALM		0,157510	0,134613	0,828075	-0,582884	-0,577647	-0,521979	-0,425319	2,715986	0,534607	-0,552765	-0,971456	-0,876932
ID1236			1,287332	#DIV/0!	2,083228	-0,454255	-0,131170	-0,558200	1,383504	2,685806	#DIV/0!	-0,355789	-0,671831	-0,298477
ID1455	HSPB1		-0,056111	0,016933	1,091443	-0,163013	-0,411623	-1,237110	-0,478496	2,329246	0,847265	-1,218861	-0,597816	-0,426571
ID3403522			-0,501480	-0,449666	-0,360383	0,904721	-0,630644	-0,667678	0,476129	2,051322	0,624310	-0,406046	-0,605729	-0,536369
ID1439			-0,839344	-0,260922	-0,042003	-0,375647	0,000641	0,240868	0,017585	2,023112	0,372989	1,535803	-1,108576	-1,180554
ID17374	PDIA3	PEPD	-0,131525	-1,189889	-0,160990	-0,589669	-0,421263	-0,391990	1,115207	1,990347	1,235387	0,349005	-0,565333	-1,152037
ID1719			-0,803240	-0,558279	-0,372213	-0,149699	-0,361309	0,224960	0,480551	1,987482	1,016044	-0,582106	-0,304139	-0,723580
ID1623			1,103758	0,955359	-0,188075	-0,140478	0,265321	-0,384722	0,939240	1,970704	0,440036	-1,544431	-0,659690	-0,686364
ID135817	ID135817		-0,330522	-0,487159	-0,001990	0,375441	-0,128048	-1,118895	0,513415	1,968149	0,799182	-0,379454	-0,408303	-0,896680
ID1460	PSB7		-0,321373	0,254481	0,513092	-0,359390	-0,344612	0,268417	0,434099	1,866997	1,202938	-0,437831	-1,425303	-1,760973
ID185509			-0,849319	-0,394283	-0,812409	0,189385	0,110257	0,539914	0,995117	1,864715	1,174663	-0,583661	-1,093468	-1,286826
ID2997219			0,168616	0,168792	0,122328	0,400079	-0,830108	-0,700139	0,630094	1,834932	0,988950	-0,596633	-0,862125	-1,473944
ID2158110			-0,810138	0,096711	-0,946290	-0,423407	-0,363088	-0,533158	0,816843	1,815975	1,933672	-0,411309	-0,445949	-0,832690
ID94683	RD23B		-0,780754	-0,591743	-0,473386	-0,469345	-0,196469	0,608091	0,850242	1,809573	0,959411	1,022461	-0,830957	-1,651510
ID1739			-0,111792	0,175973	-0,115037	0,338399	-0,156004	-0,092056	0,328864	1,788495	0,764063	-1,799511	-0,544678	-1,026595
ID3761682			0,074064	-0,923854	-0,483250	-0,752221	-1,075355	0,320029	0,881458	1,728635	1,487507	-0,426447	-0,024662	-0,912515
ID1825144	RCN2		0,002110	0,505334	0,173631	0,049639	0,068357	-0,739305	0,662931	1,659312	0,736350	-0,718536	-0,867446	-1,712011
ID732			-0,505650	-0,794966	-0,644169	-0,253238	-0,159054	-0,013710	0,519134	1,630947	0,933816	0,421975	0,510643	-1,540234
ID469498	PRS4	SYWC	-0,218102	-0,450420	0,162238	-0,597034	-0,690707	-0,363816	1,261165	1,625976	1,636697	-0,259927	-1,135849	-1,035202
ID1600			0,297361	0,482734	0,874434	0,244433	-0,348696	-0,905416	0,376053	1,616168	1,066310	-1,161007	-1,650019	-1,182608
ID217409	HEXB		-0,190395	0,381494	0,219892	-0,360722	-0,041282	0,384302	0,891387	1,592361	1,090250	-0,784352	-1,728947	-1,650076
ID1188	DDAH1	EIF3I, NPM	-0,251258	-0,166544	-0,090931	0,453060	-0,465150	-0,457839	1,585366	1,586118	0,860628	-1,420237	-1,534045	-0,454229
ID731			-0,281342	-0,809714	-0,572809	-0,212602	0,175085	-0,214430	0,686841	1,558166	0,733407	0,522631	0,397865	-1,852440
ID343050			0,273198	0,980871	0,446363	-0,559415	-0,611522	-0,801807	1,380355	1,535608	1,394132	-0,945109	-0,650162	-0,658983
ID1520			-0,918371	-0,471383	-0,673027	-0,515882	-0,577184	-0,122283	1,033488	1,511483	2,225812	-0,245346	-0,630189	-0,678453
ID706			0,040060	0,060635	0,361502	0,338630	-0,134500	-1,870986	0,684932	1,505623	0,934934	-0,393668	-0,385737	-1,239843
ID1721			0,792207	1,036431	0,375224	-0,567175	-0,776211	-1,158314	0,567929	1,501014	0,448470	-0,757366	-0,463863	-0,636724
ID2209869			-0,203041	-0,119799	-0,163852	-0,218282	-0,126322	-1,178350	1,486998	1,494456	1,210223	0,140670	-0,735767	-1,551765
ID1410			0,478204	0,651591	1,049038	-0,097683	-0,121956	-0,052124	0,365868	1,480805	0,515614	-1,012210	-1,635300	-1,874901
ID996			-1,435769	1,289590	-0,804186	0,315181	1,046001	-0,640167	-0,081746	1,473314	0,571089	-0,061137	-0,464151	-1,223305
ID975	VIME		-1,312529	1,022692	-0,613990	0,253015	0,012957	-2,009505	0,757739	1,439540	0,770331	0,526402	-0,008849	-0,706200
ID1341	COPE	ANXA5	0,415496	0,331453	0,369127	-0,250555	-0,362088	0,038719	1,183418	1,430534	0,800325	-1,046844	-1,587389	-1,583908
ID1034	ACTG	K1C18	-0,251704	-0,096446	0,178076	-0,392606	-0,499070	0,120770	1,621890	1,427121	1,332625	-0,999873	-1,187522	-1,503230
ID3762817			0,055508	0,038298	0,553286	-0,503915	-0,652067	-0,719014	0,430408	1,392615	1,084505	-0,312649	0,149344	-1,594482
ID1343	ANXA4		0,143288	0,495900	0,669357	0,015167	-0,144738	-0,606528	0,339999	1,367700	0,772337	0,924557	-1,817322	-1,928578
ID3765751			0,645731	0,122321	0,717628	-0,025642	-0,523762	-0,014913	0,918394	1,352686	0,213112	-0,742042	-1,798209	-1,050813
ID3170569			-1,117523	-0,849618	-0,585246	-1,045266	-0,040401	0,805559	1,518232	1,339932	1,244483	-0,431118	-0,930968	-0,015845
ID1471	PRDX4		-0,523321	0,489099	-0,120045	0,535541	-0,477110	-0,949690	-0,185880	1,322370	1,156007	1,890735	-1,147580	-1,517442
ID1609			0,084788	0,318874	0,427135	-0,789457	-0,287102	-0,569919	1,253805	1,309367	1,453905	-0,292360	-1,375027	-1,607100
ID1468	PHB	HSPB1	-0,593959	-0,433732	-0,451607	0,617942	0,413374	0,873652	0,817668	1,291238	1,119093	-0,555665	-1,338510	-1,898412
ID1035	ACTG	K1C18, CHD9	0,539429	0,754342	0,833610	0,009434	-0,431370	-0,370919	1,057880	1,251298	0,533990	-1,358846	-1,536057	-1,622503
ID1477			-0,415022	0,304636	-0,076204	0,465938	-0,120679	-0,141787	1,304542	1,244340	1,035775	-0,938253	-1,234212	-1,663637
ID327			0,037574	-0,214188	0,019725	0,609199	0,088723	-0,895421	0,836299	1,243185	0,572438	-0,813946	-0,060491	-1,626583
ID987			0,400414	0,495411	0,777049	0,478799	-0,374706	-0,429899	-1,161567	1,236964	1,124778	-1,011724	-0,695319	-1,093132
ID927	K1C17	ATPB, PDIA6	-0,475962	0,089898	-0,268757	-0,476994	-0,722890	-0,370121	1,246716	1,233793	1,832655	0,683077	-1,128603	-1,472043
ID578			-0,604014	-0,659341	-0,375158	-0,672411	-0,212929	-0,211972	1,361689	1,233681	1,900119	-0,523555	-0,512660	-0,854337
ID281947			0,292510	0,601636	0,337968	-0,587651	-0,923398	-0,757399	0,140695	1,233297	1,258964	-0,103739	-0,630727	-0,888090
ID2074209			-0,137824	-0,364271	0,089750	-0,233138	-0,102454	-0,734746	0,721040	1,196421	1,103732	0,110745	0,112457	-1,734026
ID1638	SSRD		0,093357	0,167527	0,269350	0,304223	0,223074	-0,937635	0,331138	1,149448	1,922059	-1,592053	-1,424380	-0,904121
ID230824	PDIA1		-0,912280	-0,702580	0,533004	-0,860565	0,541610	0,516353	0,205129	1,128813	1,690719	-0,400893	-0,990260	-0,849274
ID2722863			-0,472185	-1,214789	-0,471351	0,598480	-0,428096	0,566322	1,237645	1,072191	1,324372	-1,417952	-0,232033	-0,917092
ID986	K1C18	ACTB, NUP43	0,081056	0,226858	0,153469	-0,072471	0,036247	-0,234819	1,706640	1,067853	1,063904	-1,590734	-1,272994	-1,562691
ID3259231			-0,653458	0,002306	0,251427	0,700972	-0,195330	0,130653	0,996740	1,062418	0,820086	-2,333088	-0,775866	-0,590130
ID2753			0,845033	0,865028	0,963633	-0,875525	-0,856357	-0,586757	1,024870	1,030821	0,622444	-1,779611	-1,028074	-0,670406
ID1530	CBX5	CATB	0,060731	0,171949	0,118529	0,329819	0,282466	0,552259	0,707122	1,025583	1,164186	-1,891033	-1,377992	-1,616378
ID12547	HDAC1	HNRPK, CH60	0,303971	-0,152900	0,967879	0,619315	0,182253	-0,792720	1,446130	0,999456	-0,070560	-1,209002	-0,999325	-1,596747
ID3557801			-0,490614	0,405221	-0,343992	0,824876	1,060556	-0,747677	0,572058	0,999249	0,704364	-0,500364	-0,836509	-1,772259

Table S-4: cluster 10

ID)1	Protein)2		co_0 min	co_30 min	co_60 min	co_24 h	co_48 h	co_72 h	PI_0 min	PI_30 min	PI_60 min	PI_24 h	PI_48 h	PI_72 h
ID3668269			0,706499	0,800281	0,652664	-0,632478	-0,498297	-0,146333	1,173449	0,981949	0,954024	-1,344003	-1,288072	-1,695683
ID2880272			-1,036841	-0,664549	-0,988830	-0,560556	-0,053896	0,747383	1,534561	0,976970	1,312403	0,341002	-0,429403	-1,092994
ID1532			0,325214	0,061471	0,683157	-0,206406	-0,295513	0,814498	0,399189	0,975676	0,591704	-2,276522	-0,982324	-0,659274
ID712	LMNB1		-0,479115	0,282523	-0,400867	-0,192916	0,280936	1,506102	1,112496	0,967887	0,677919	-1,863307	-0,744386	-1,613098
ID831	CALR		0,086930	0,663377	0,291461	-1,309118	-0,816110	-0,012308	0,951498	0,961302	1,393437	0,350379	-0,834171	-1,639081
ID399832	PAK2	PDIA3	-0,173925	-0,038464	0,246458	-0,227103	-0,541182	-0,104586	0,488882	0,932503	2,053092	-0,153376	-0,601820	-1,918823
ID3497145			0,461893	0,526643	0,629586	0,021290	0,166807	0,025417	0,767047	0,911445	0,878776	-0,958009	-1,187063	-2,483334
ID977	TXND5	HNRPF, TBAK	-0,210851	0,410556	0,011337	-0,083715	-0,230310	0,137574	1,204845	0,902372	1,168211	0,598222	-1,814227	-1,944459
ID10303			1,059583	0,771689	0,855203	-0,309569	-0,453540	-0,717769	0,198997	0,888043	0,312228	-0,729569	-0,929206	-1,128481
ID2091139			0,259856	0,367000	0,152359	0,126043	-0,113038	-2,058319	0,607370	0,824484	1,182506	-0,373298	-0,180946	-0,887341
ID865	PDIA3	TBA1B	0,521315	0,817726	0,626803	-0,237747	-0,173002	0,223940	0,666505	0,814613	0,861039	-0,383361	-1,990412	-1,843259
ID1096			0,751317	0,414983	1,225448	-0,072669	-0,603373	-0,195367	0,633656	0,800985	0,900089	-1,377086	-1,182135	-1,640119
ID992	TXND5	ACL6A	0,688591	0,733958	0,838559	0,140107	0,002850	-0,726757	0,896924	0,799758	0,915035	-1,187526	-1,646185	-1,752196
ID1046	ARP3	TADBP	-1,128150	-0,103282	-0,386337	0,354964	0,513328	1,588774	1,214154	0,793572	0,479798	-0,788560	-1,389921	-1,345482
ID3763073			-0,307942	-0,434587	-0,735759	-0,023152	0,610436	0,390901	0,786651	0,783581	0,651649	-0,937843	0,127334	-1,145728
ID708	LMNB1		-0,737554	-0,015215	-0,937724	-0,374227	0,543716	1,283374	1,424599	0,778317	1,076935	-0,964819	-0,721278	-1,597330
ID1334	ANXA5		0,294356	0,400788	1,050840	-0,942836	-0,652523	-0,168895	1,148410	0,755010	1,378277	-0,366134	-1,416468	-1,572357
ID1524	NDUV2		0,069704	0,838279	0,418526	-0,355541	0,117333	0,125545	0,522903	0,754999	1,277657	-0,536071	-1,422201	-1,945152
ID268600			-1,011274	-0,551655	-0,820201	-0,333340	-0,778166	0,761796	1,145377	0,754412	1,629075	0,582991	-0,611319	-0,621947
ID198671	ERO1A		0,568565	0,871376	1,232953	-0,585815	-0,335889	-0,244540	0,512161	0,747597	0,819206	-0,157738	-1,621587	-1,845724
ID2150207			-0,699980	-0,677476	-0,792493	0,367827	-1,183086	1,048113	0,852492	0,734231	1,189392	-0,192900	0,039362	-0,733706
ID733			-0,140594	0,201393	-1,035011	0,851607	-0,465825	0,520072	0,685972	0,706751	0,799967	-1,518572	-0,238463	-0,746939
ID1013	UQCR1		-0,080704	0,379075	0,396924	0,576865	0,269940	-0,239930	1,023026	0,650953	1,338859	-1,983653	-1,765739	-1,061530
ID1537			0,692963	0,641482	0,159562	-0,131591	-0,196642	0,307261	0,796205	0,639299	0,692234	-1,764804	-0,595022	-1,642256
ID1484			-1,162699	-0,038839	-0,302237	1,265053	0,431830	0,203627	0,932840	0,627242	0,730555	0,426245	-1,035908	-1,971147
ID451640	PDIA3		0,419326	-1,389954	0,219401	-0,777408	-0,614237	0,355686	1,505771	0,615331	0,607421	0,943100	-0,625896	-1,022766
ID2152149			-0,881169	-0,106615	-0,842636	0,318123	0,116427	0,303747	0,855123	0,596437	1,364973	-0,408019	-0,242529	-1,175866
ID1983973			0,104991	0,588575	0,294443	-0,608067	-0,125938	0,522040	0,490448	0,570414	0,939244	-0,737543	-0,778141	-1,444852
ID692	FKBP9		-0,540464	-0,249069	-0,724166	0,231583	-0,410871	1,513742	0,749377	0,505934	0,937428	0,051925	-0,741370	-1,311070
ID1059			-0,308212	0,045138	-0,019104	-0,114349	0,848376	1,025457	0,287460	0,483424	0,899750	0,331570	-1,475722	-1,920896
ID1289			-0,115221	0,598629	0,456638	0,537440	-0,185078	0,248783	1,113596	0,439352	0,097804	0,656756	-1,602807	-2,081703
ID11611			-0,506549	0,025162	-0,996692	-0,397709	0,634788	1,633315	0,983111	0,437852	1,227031	-0,971220	-0,829094	-1,482800
ID2089255			0,386295	0,696663	0,375032	0,448909	0,469865	-1,626973	0,551001	0,435387	0,863386	-0,542837	-0,765251	-1,427186
ID884	K2C8	UAP56, IF2B, GSHB	0,363690	0,446195	0,167198	0,069237	0,509083	-0,033124	1,776435	0,412810	0,478026	-1,964778	-1,086535	-1,629432
ID1985723			-0,396047	-0,130840	0,343119	0,870371	0,244978	0,330772	0,949247	0,372038	0,637428	-1,718912	-1,037312	-0,894570
ID324496			0,056714	-0,421131	0,319501	0,601732	0,932837	0,001011	1,438393	0,299389	0,402035	-1,616072	-1,038723	-1,379704
ID1570			0,651226	0,056402	0,239706	0,259987	-0,692456	-0,455066	0,915368	0,296842	0,599808	-1,347551	0,429664	-1,290816
ID13892	ACL6A	IF4A1, HNRPF	-0,352054	0,025964	-0,280611	0,898001	0,720094	0,501374	1,382754	0,235367	0,939961	-1,577699	-1,151340	-1,736236
ID1560	ATP5H	CBX5	-0,716806	0,136294	-0,410641	-0,475421	-0,542017	-0,421009	1,567107	0,161192	1,844288	1,414903	-0,991851	-1,212314
ID537545	LMNB1	TKT	-0,876789	-0,275346	-1,523173	0,048582	0,550050	1,640678	1,228052	0,053259	1,137451	-0,487577	-0,477052	-1,140030
ID366771	GRP78		-0,362374	-0,046841	-0,859911	-0,151318	0,318794	1,942003	1,283304	0,022978	0,896262	-1,264311	-0,827054	-1,267609
ID1601			0,149155	0,255659	0,119875	0,459666	0,507155	0,392231	#DIV/0!	-0,066389	1,614028	-0,210813	-1,159845	-1,433451
ID2666660	SPRC		0,320017	0,849330	0,297831	-1,464509	-0,567122	1,457197	0,412302	-0,067052	1,267135	-0,032689	-1,496218	-0,984394
ID141431			-0,403803	-0,675833	-0,318596	0,404100	0,384913	0,362108	-0,014010	-0,548777	2,321688	-0,336386	-0,511507	-0,391420

Table S-5: Summary results from network-analysis using Ingenuity Pathway Analysis (IPA) for each single cluster.
Spot-lists of MALDI-TOF-MS identified proteins from statistical-analyses of each cluster were analysed.
Resulting networks, molecular and cellular functions, as well as “Top Tox Lists” and the respective scores are displayed.
The same categories of “Top Tox Lists” or “molecular and cellular functions” were coloured equally.

Table S-5

	rank	Associated Network Functions	score
**cluster 1**	1	Post-translational modification, cancer, cell morphology	50
	2	Post-translational modification, protein folding, inflammatory desease	36
	3	Cell death, gene expression, DNA replication, recombination and repair	25
	4	Genetic disorder, metabolic disease, cellular development	21
	5	Endocrine system development and function, small molecule biochemistry, lipid metabolism	2
**cluster 2**	1	Post-translational modification, protein folding, cell function and maintainance	56
	2	Cancer, cellular development, tumor morphology	18
	3	Cell cycle, cancer, cardiovascular disease	18
	4	Cell cycle, hair and skin development and function, embryonic development	1
	5	n.d.	n.d.
**cluster 3**	1	Post-translational modification, protein folding, cancer	61
	2	Cellular compromise, inflammatory response, lipid metabolism	27
	3	Dermatological disease and conditions, genetic disorder, cell-to-cell signalling and interaction	24
	4	Development disorder, amino acid metabolism, small molecule biochemistry	2
	5	n.d.	n.d.
**cluster 4**	1	Cellular assembly and organization, post-translational modification, protein folding	58
	2	Cellular function and maintainance, cellular development, hematological system development and function	27
	3	Cancer, cellular development, cellular growth and proliferation	26
	4	Cell morphology, cellular compromise, connective tissue development	22
	5	Cellular assembly and organization, protein synthesis, DNA replication, recombination and repair	11
**cluster 5**	1	Cancer, reproductive disease, cardivascular disease	63
	2	Cellular assembly and organization, genetic disorder, neurological disease	39
	3	Post-translational modification, protein folding, cell morphology	31
	4	Drug metabolism, endocrine system development and function, lipid metabolism	22
	5	Cellular assembly and organization, cellular movement, cellular development	13

Table S-5

	rank	Associated Network Functions	score
**cluster 6**	1	Protein degradation, cell-to-cell signalling and interaction, hematological system development and function	37
	2	Molecular transport, small molecule biochemistry, infectious disease	11
	3	Reproductive system disease, genetic disorder, skeletal and muscular disorders	2
	4	n.d.	n.d.
	5	n.d.	n.d.
**cluster 7**	1	Post-translational modification, protein folding, cellular compromise	58
	2	Cellular assembly and organization, genetic disorder, skeletal and muscular disorder	47
	3	Cancer, reproductive system disease, cardiovascular disease	46
	4	Cardiovascular system development and function, genetic disorder, metabolic disease	45
	5	Molecular transport, nucleic acid metabolism, small molecule biochemistry	42
**cluster 8**	1	Genetic disorder, skeletal and muscular disorders, Cellular assembly and organization	55
	2	Cell death, RNA post-translational modification	54
	3	Cellular function and maintainance, neurological disease, cell death	42
	4	Cellular assembly and organization, cell death, cellular function and maintainance	24
	5	Cell morphology, cellular compromise, drug metabolism	21
**cluster 9**	1	Cancer, reproductive system disease, cardiovascular disease	55
	2	Cell death, genetic disorder, neurological disease	50
	3	Lipid metabolism, post-translational modification, small molecule biochemistry	37
	4	Cellular development, drug metabolism, lipid metabolism	25
	5	Cellular development, cellular growth and proliferation, cell death	20
**cluster 10**	1	Cellular assembly and organization, hair and skin development and function, hepatic system development and function	68
	2	Cell morphology, cellular movement, organ development	32
	3	Neurological disease, organismal injury and abnormalities, small molecule biochemistry	26
	4	Cellular assembly and organization, molecular transport, RNA trafficking	2
	5	n.d.	n.d.

Table S-5

	rank	Top Tox lists	p-value / ratio
**cluster 1**	1	Mechanism of gene regulation by peroxisome proliferators via PPAR alpha	4.31E-04 / 4/95 (0.042)
	2	PPAR alpha/RXR alpha activation	3.32E-03 / 4/165 (0.024)
	3	Nrf2 mediated oxidative stress response	7.14E-03 / 4/205 (0.02)
	4	Liver necrosis / cell death	1.84E-02 / 3/150 (0.02)
	5	Decrease permeability transition of mitochondria and mitochondrial membrane	1.85E-02 / 1/5 (0.2)
**cluster 2**	1	Decrease permeability transition of mitochondria and mitochondrial membrane	1.36E-02 / 1/5 (0.2)
	2	Hypoxia inducible factor signalling	1.56E-02 / 2/70 (0.029)
	3	Nrf2 mediated oxidative stress response	1.83E-02 / 3/205 (0.015)
	4	Increase Bradycardia	2.17E-02 / 1/8 (0.125)
	5	Liver necrosis / cell death	6.32E-02 / 2/150 (0.013)
**cluster 3**	1	PPAR alpha/RXR alpha activation	1.88E-03 / 4/165 (0.024)
	2	Mechanism of gene regulation by peroxisome proliferators via PPAR alpha	3.46E-03 / 3/95 (0.032)
	3	cell cycle: G2/M DNA damage checkpoint regulation	5.57E-03 / 2/35 (0.057)
	4	Decrease permeability transition of mitochondria and mitochondrial membrane	1.59E-02 / 1/5 (0.2)
	5	PXR/RXR activation	2.00E-02 / 2/68 (0.029)
**cluster 4**	1	Nrf2 mediated oxidative stress response	6.33E-07 / 9/205 (0.044)
	2	Aryl hydrocarbon receptor signalling	9.17E-05 / 6/151 (0.04)
	3	Hypoxia inducible factor signalling	4.79E-03 / 3/70 (0.043)
	4	Mechanism of gene regulation by peroxisome proliferators via PPAR alpha	1.11E-02 / 3/95 (0.032)
	5	Fatty acid metabolism	2.49E-02 / 3/129 (0.023)
**cluster 5**	1	Nrf2 mediated oxidative stress response	2.66E-05 / 6/205 (0.039)
	2	Liver necrosis / cell death	1.17E-02 / 4/150 (0.027)
	3	Decrease permeability transition of mitochondria and mitochondrial membrane	2.90E-02 / 1/5 (0.2)
	4	Increase transmembrane potential of mitochondria and mitochondrial membrane	3.47E-02 / 2/50 (0.04)
	5	Oxidative stress	4.41E-02 / 2/57 (0.035)

Table S-5

	rank	Top Tox lists	p-value / ratio
**cluster 6**	1	Nrf2 mediated oxidative stress response	2.07E-03 / 3/205 (0.015)
	2	Decrease permeability transition of mitochondria and mitochondrial membrane	6.31E-03 / 1/5 (0.2)
	3	Oxidative stress	6.98E-02 / 1/57 (0.018)
	4	Mitochondrial dysfunction	1.47E-01 / 1/125 (0.008)
	5	Cardiac hypertrophy	2.70E-01 / 1/246 (0.004)
**cluster 7**	1	Nrf2 mediated oxidative stress response	1.55E-04 / 11/205 (0.054)
	2	cell cycle: G2/M DNA damage checkpoint regulation	1.41E-03 / 4/35 (0.114)
	3	Oxidative stress	8.39E-03 / 4/57 (0.07)
	4	Mechanism of gene regulation by peroxisome proliferators via PPAR alpha	1.09E-02 / 5/95 (0.053)
	5	Aryl hydrocarbon receptor signalling	1.99E-02 / 6/151 (0.04)
**cluster 8**	1	Nrf2 mediated oxidative stress response	4.95E-03 / 6/205 (0.029)
	2	Increase transmembrane potential of mitochondria and mitochondrial membrane	6.61E-03 / 3/50 (0.06)
	3	Aryl hydrocarbon receptor signalling	2.88E-02 / 4/151 (0.026)
	4	Decrease permeability transition of mitochondria and mitochondrial membrane	3.77E-02 / 1/5 (0.2)
	5	Oxidative stress	7.07E-02 / 2/57 (0.035)
**cluster 9**	1	Hypoxia-inducible factor signalling	1.40E-03 / 4/70 (0.057)
	2	Nrf2 mediated oxidative stress response	3.02E-03 / 6/205 (0.029)
	3	Aryl hydrocarbon receptor signalling	3.99E-03 / 5/151 (0.033)
	4	PPAR alpha/RXR alpha activation	1.07E-01 / 3/165 (0.018)
	5	Glutathione depletion - phase II reactions	1.36E-01 / 1/21 (0.048)
**cluster 10**	1	Liver necrosis / cell death	1.67E-02 / 3/150 (0.02)
	2	Decrease permeability transition of mitochondria and mitochondrial membrane	1.79E-02 / 1/5 (0.2)
	3	Oxidative stress	1.79E-02 / 2/57 (0.035)
	4	Hypoxia-inducible factor signalling	2.63E-02 / 2/70 (0.029)
	5	Increase Bradycardia	2.84E-02 / 1/8 (0.125)

Table S-5

	rank	Molecular and cellular functions	p-value / molecules
**cluster 1**	1	cell death	2.62E-06 - 4.74E-02 / 28
	2	post-translational modification	1.04E-05 - 2.94E-02 / 12
	3	protein folding	1.04E-05 - 3.37E-03 / 5
	4	cellular movement	2.25E-04 - 4.74E-02 / 19
	5	cellular growth and proliferation	3.18E-04 - 4.74E-02
**cluster 2**	1	post-translational modification	5.83E-08 - 1.53E-02 / 10
	2	protein folding	5.83E-08 - 1.05E-06 / 6
	3	cellular function and maintainance	5.91E-05 - 4.91E-02 / 6
	4	protein degradation	7.24E-05 - 7.24E-05 / 2
	5	protein synthesis	1.88E-04 - 3.95E-02 / 8
**cluster 3**	1	post-translational modification	1.06E-10 - 5.00E-02 / 19
	2	protein folding	1.06E-10 - 2.53E-02 / 8
	3	amino acid metabolism	3.06E-07 - 2.19E-02 / 6
	4	small molecule biochemistry	3.06E-07 - 4.39E-02 / 17
	5	cell to cell signalling and interaction	4.16E-06 - 5.00E-02 / 16
**cluster 4**	1	post-translational modification	1.94E-06 - 3.83E-02 / 14
	2	protein folding	1.94E-06 - 6.41E-04 / 6
	3	cell morphology	1.09E-04 - 4.78E-02 / 20
	4	cell death	1.18E-04 - 4.91E-02 / 28
	5	cell to cell signalling and interaction	2.31E-02 - 4.29E-02 / 6
**cluster 5**	1	cellular growth and proliferation	1.62E-10 - 3.47E-02 / 42
	2	post-translational modification	1.53E-08 - 3.47E-02 / 15
	3	protein folding	1.53E-08 - 1.17E-02 / 9
	4	cell death	2.50E-06 - 3.47E-02 / 37
	5	protein synthesis	1.17E-05 - 3.14E-02 / 15

Table S-5

	rank	Molecular and cellular functions	p-value / molecules
**cluster 6**	1	protein degradation	1.51E-05 - 1.51E-05 / 2
	2	cell death	1.25E-04 - 4.31E-02 / 6
	3	gene expression	1.37E-04 - 4.71E-02 / 6
	4	protein synthesis	1.37E-04 - 2.68E-02 / 4
	5	cellular development	1.25E-03 - 4.22E-02 / 9
**cluster 7**	1	cellular growth and proliferation	1.02E-13 - 3.84E-02 / 73
	2	cell death	1.04E-09 - 4.06E-02 / 88
	3	post-translational modification	9.69E-09 - 3.66E-02 / 38
	4	protein folding	9.69E-09 - 1.41E-02 / 11
	5	cellular movement	2.08E-05 - 2.79E-02 / 34
**cluster 8**	1	cellular growth and proliferation	3.62E-09 - 3.08E-02 / 55
	2	cell death	3.42E-07 - 3.46E-02 / 44
	3	cellular function and maintainance	2.18E-06 - 3.77E-02 / 22
	4	cellular movement	1.07E-05 - 2.78E-02 / 28
	5	cellular assembly and organization	2.58E-05 - 3.77E-02 / 34
**cluster 9**	1	cellular growth and proliferation	2.82E-05 - 3.42E-02 / 30
	2	cellular assembly and organization	4.76E-05 - 4.75E-02 / 21
	3	cell death	7.01E-05 - 4.75E-02 / 38
	4	cell morphology	1.42E-04 - 4.89E-02 / 15
	5	amino acid metabolism	4.69E-04 - 4.09E-02 / 4
**cluster 10**	1	cellular growth and proliferation	2.44E-07 - 3.19E-02 / 22
	2	post-translational modification	3.18E-07 - 2.84E-02 / 11
	3	protein folding	3.18E-07 - 2.84E-02 / 6
	4	cell death	6.99E-07 - 4.77E-02 / 25
	5	cellular assembly and organization	1.27E-05 - 4.92E-02 / 17

Table S-6: Up-regulated proteins (fold-changes ≥1.5) in response to short-term and long-term cultivation after CAP treatment or both. Protein annotation name)[1], function)[2], highest fold-change)[3] and a (presumed) functional description)[4] is given. These proteins can serve as marker proteins.

Table S-6

Short term induced

Protein)1	Function)2	Fold changes)3	Functional description)4
CALM	Calmodulin-1	3,171241	mediates the control of a large number of enzymes, ion channels, aquaporins and other proteins through calcium-binding
NSAP	Heterogeneous nuclear ribonucleoprotein Q	2,667590	implicated in mRNA processing mechanisms. Component of the CRD-mediated complex that promotes MYC mRNA stability
TCPA	T-complex protein 1 subunit alpha	2,113189	molecular chaperone; assists the folding of proteins upon ATP hydrolysis; as part of the BBS/CCT complex may play a role in the assembly of BBSome, a complex involved in ciliogenesis regulating transports vesicles to the cilia
LMNB1	Lamin-B1	2,107199	components of the nuclear lamina, a fibrous layer on the nucleoplasmic side of the inner nuclear membrane, which is thought to provide a framework for the nuclear envelope and may also interact with chromatin
VIME	Vimentin	2,020745	vimentins are class-III intermediate filaments found in various non-epithelial cells, especially mesenchymal cells; attached to the nucleus, endoplasmic reticulum, and mitochondria, either laterally or terminally; major cytoskeletal component of mesenchymal cells. Because of this, vimentin is often used as a marker of mesenchymally-derived cells or cells undergoing an epithelial-to-mesenchymal transition (EMT) during both normal development and metastatic progression.
HSPB1	Heat shock protein beta-1	1,906802	small heat shock protein which functions as a molecular chaperone probably maintaining denatured proteins in a folding-competent state. Plays a role in stress resistance and actin organization
IMA7	Importin subunit alpha-7	1,800911	functions in nuclear protein import as an adapter protein for nuclear receptor KPNB1
FSTL1	Follistatin-related protein 1	1,778680	may modulate the action of some growth factors on cell proliferation and differentiation
KBP	KIF1-binding protein	1,660694	required for organization of axonal microtubules, and axonal outgrowth and maintenance during peripheral and central nervous system development
K2C8	Keratin, type II cytoskeletal 8	1,660645	plays a role in keratin filament assembly
LMNA	Prelamin-A/C	1,623751	components of the nuclear lamina, a fibrous layer on the nucleoplasmic side of the inner nuclear membrane, which is thought to provide a framework for the nuclear envelope and may also interact with chromatin
PRS4	26S proteasome regulatory subunit 4	1,596090	component of the 26S proteasome, a multiprotein complex involved in the ATP-dependent degradation of ubiquitinated proteins; this complex plays a key role in the maintenance of protein homeostasis by removing misfolded or damaged proteins, which could impair cellular functions, and by removing proteins whose functions are no longer required; therefore, the proteasome participates in numerous cellular processes, including cell cycle progression, apoptosis, or DNA damage repair
MX1	Interferon-induced GTP-binding protein Mx1	1,556947	interferon-induced dynamin-like GTPase with antiviral activity against a wide range of RNA viruses and some DNA viruses; enhances ER stress-mediated cell death after influenza virus infection. May regulate the calcium channel activity of TRPCs
FKBP9	Peptidyl-prolyl cis-trans isomerase FKBP9	1,555623	PPIases accelerate the folding of proteins during protein synthesis
MARCS	Myristoylated alanine-rich C-kinase substrate	1,536069	MARCKS is the most prominent cellular substrate for protein kinase C; binds calmodulin, actin, and synapsin; filamentous (F) actin cross-linking protein
CH60	60 kDa heat shock protein, mitochondrial	1,529091	chaperonin implicated in mitochondrial protein import and macromolecular assembly. Together with Hsp10, facilitates the correct folding of imported proteins; may also prevent misfolding and promote the refolding and proper assembly of unfolded polypeptides generated under stress conditions in the mitochondrial matrix
P4HA1	Prolyl 4-hydroxylase subunit alpha-1	1,525163	catalyzes the post-translational formation of 4-hydroxyproline in -Xaa-Pro-Gly- sequences in collagens and other proteins
K1C17	Keratin, type I cytoskeletal 17	1,502958	involved in tissue repair; acts as a promoter of epithelial proliferation by acting a regulator of immune response in skin; positive regulation of cell growth

Table S-6

Long term induced

Protein)1	Function)2	Fold changes)3	Functional description)4
QCR1	Cytochrome b-c1 complex subunit 1, mitochondrial	11,943578	this is a component of the ubiquinol-cytochrome c reductase complex (complex III or cytochrome b-c1 complex), which is part of the mitochondrial respiratory chain.
CALD1	Caldesmon	6,007415	actin- and myosin-binding protein implicated in the regulation of actomyosin interactions in smooth muscle and nonmuscle cells (could act as a bridge between myosin and actin filaments)
DP13B	DCC-interacting protein 13-beta	4,096890	required for the regulation of cell proliferation in response to extracellular signals mediated by an early endosomal compartment
SCFD1	Sec1 family domain-containing protein 1	3,165289	plays a role in SNARE-pin assembly and Golgi-to-ER retrograde transport via its interaction with COG4. Involved in vesicular transport between the endoplasmic reticulum and the Golgi
TXND5	Thioredoxin domain-containing protein 5	2,695739	possesses thioredoxin activity. Has been shown to reduce insulin disulfide bonds; complements protein disulfide-isomerase deficiency in yeast
SYG	Glycine--tRNA ligase	2,686072	catalyzes the ligation of glycine to the 3'-end of its cognate tRNA. Also produces diadenosine tetraphosphate (Ap4A), a universal pleiotropic signaling molecule needed for cell regulation pathways, by direct condensation of 2 ATPs
COR1B	Coronin-1B	2,381682	regulates leading edge dynamics and cell motility in fibroblasts; may be involved in cytokinesis and signal transduction
ERP29	Endoplasmic reticulum resident protein 29	2,181211	plays an important role in the processing of secretory proteins within the endoplasmic reticulum (ER), possibly by participating in the folding of proteins in the ER
RCN1	Reticulocalbin-1	2,177496	may regulate calcium-dependent activities in the endoplasmic reticulum lumen or post-ER compartment
PRDX4		2,154153	thiol-specific peroxidase that catalyzes the reduction of hydrogen peroxide and organic hydroperoxides to water and alcohols, respectively; plays a role in cell protection against oxidative stress by detoxifying peroxides and as sensor of hydrogen peroxide-mediated signaling events
EZRI	Ezrin	2,135899	probably involved in connections of major cytoskeletal structures to the plasma membrane. In epithelial cells, required for the formation of microvilli and membrane ruffles on the apical pole. Along with PLEKHG6, required for normal macropinocytosis
TXNL5	Thioredoxin domain-containing protein 17	2,130670	disulfide reductase. May participate in various redox reactions through the reversible oxidation of its active center dithiol to a disulfide and catalyze dithiol-disulfide exchange reactions. Has peroxidase activity and may contribute to the elimination of cellular hydrogen peroxide
NPM	Nucleophosmin	2,098274	involved in diverse cellular processes such as ribosome biogenesis, centrosome duplication, protein chaperoning, histone assembly, cell proliferation, and regulation of tumor suppressors p53/TP53 and ARF
STML2	Stomatin-like protein 2, mitochondrial	2,049966	mitochondrial protein that probably regulates the biogenesis and the activity of mitochondria; through regulation of the mitochondrial function may play a role into several biological processes including cell migration, cell proliferation, T-cell activation, calcium homeostasis and cellular response to stress
THIO	Thioredoxin	1,980648	participates in various redox reactions through the reversible oxidation of its active center dithiol to a disulfide and catalyzes dithiol-disulfide exchange reactions; plays a role in the reversible S-nitrosylation of cysteine residues in target proteins, and thereby contributes to the response to intracellular nitric oxide
MAOX	NADP-dependent malic enzyme	1,970692	glycolytic enzyme ???
HYOU1	Hypoxia up-regulated protein 1	1,908457	has a pivotal role in cytoprotective cellular mechanisms triggered by oxygen deprivation; may play a role as a molecular chaperone and participate in protein folding
SF3B2	Splicing factor 3B subunit 2	1,878931	involved in pre-mRNA splicing as a component of the splicing factor SF3B complex
PSME2	Proteasome activator complex subunit 2	1,821719	implicated in immunoproteasome assembly and required for efficient antigen processing
GELS	Gelsolin	1,814354	calcium-regulated, actin-modulating protein that binds to the plus (or barbed) ends of actin monomers or filaments, preventing monomer exchange (end-blocking or capping); can promote the assembly of monomers into filaments (nucleation) as well as sever filaments already formed; plays a role in ciliogenesis
PHP14	14 kDa phosphohistidine phosphatase	1,771815	peptidyl-histidine dephosphorylation; positive regulation of cell motility; regulation of actin cytoskeleton reorganization
LG3BP	Galectin-3-binding protein	1,715210	promotes integrin-mediated cell adhesion. May stimulate host defense against viruses and tumor cells
CBX5	Chromobox protein homolog 5	1,637215	component of heterochromatin that recognizes and binds histone H3 tails methylated at 'Lys-9' (H3K9me), leading to epigenetic repression
PDIA6	Protein disulfide-isomerase A6	1,624036	may function as a chaperone that inhibits aggregation of misfolded proteins
ATPB	ATP synthase subunit beta, mitochondrial	1,552042	mitochondrial membrane ATP synthase (F1F0 ATP synthase or Complex V) produces ATP from ADP in the presence of a proton gradient across the membrane
TSNAX	Translin-associated protein X	1,518427	acts in combination with TSN as an endonuclease involved in the activation of the RNA-induced silencing complex
PSME1	Proteasome activator complex subunit 1	1,515996	implicated in immunoproteasome assembly and required for efficient antigen processing

Table S-6

Short & Long term induced

Protein[1]	Function[2]	Fold changes[3]	Functional description[4]
ARP3	ARP3 actin-related protein 3	55,374106	regulation of actin polymerization; role in ciliogenesis
DCTN1	dynactin 1	7,157172	key role in dynein-mediated retrograde transport of vesicles and organelles along microtubules; regulation of microtubule stability by promoting microtubule formation
GRP78 (BIP)	heat shock 70kDa protein 5 (glucose-regulated protein, 78kDa)	5,797061	endoplasmic reticulum chaperone that plays a key role in protein folding and quality control in the endoplasmic reticulum lumen
UBQL1	ubiquilin 1	4,268907	important role in the regulation of different protein degradation mechanisms and pathways including ubiquitin-proteasome system (UPS); attenuating the induction of UPR-inducible genes
2AAB	protein phosphatase 2, regulatory subunit A, beta	4,190876	positive regulation of extrinsic apoptotic signaling pathway in absence of ligand
RD23B	UV excision repair protein RAD23 homolog B	3,880998	involved in global genome nucleotide excision repair (GG-NER) by acting as component of the XPC complex; the XPC complex recognizes a wide spectrum of damaged DNA characterized by distortions of the DNA helix such as single-stranded loops, mismatched bubbles or single-stranded overhangs
HNRPK	heterogeneous nuclear ribonucleoprotein K	3,874541	plays an important role in p53/TP53 response to DNA damage;
MCM6	DNA replication licensing factor MCM6	3,850676	acts as component of the MCM2-7 complex (MCM complex) which is the putative replicative helicase essential for 'once per cell cycle' DNA replication initiation and elongation in eukaryotic cells
TM1L2	TOM1-like protein 2	3,834643	probable role in protein transport; may regulate growth factor-induced mitogenic signaling
DPYL2	dihydropyrimidinase-related protein 2	3,795573	plays a role in neuronal development and polarity, as well as in axon growth and guidance, neuronal growth cone collapse and cell migration; microtubule binding
HSP71	Heat shock 70 kDa protein 1A	3,713209	plays a pivotal role in the protein quality control system, ensuring the correct folding of proteins, the re-folding of misfolded proteins and controlling the targeting of proteins for subsequent degradation
CALR	calreticulin	3,664755	Calcium-binding chaperone that promotes folding, oligomeric assembly and quality control in the endoplasmic reticulum (ER) via the calreticulin/calnexin cycle
ENOA	enolase 1, (alpha)	3,540622	multifunctional enzyme that, as well as its role in glycolysis, plays a part in various processes such as growth control, hypoxia tolerance and allergic responses
TRXR1	thioredoxin reductase 1	3,520093	isoform 1 may possess glutaredoxin activity as well as thioredoxin reductase activity and induces actin and tubulin polymerization, leading to formation of cell membrane protrusions
ALDOA	Fructose-bisphosphate aldolase A	3,495068	plays a key role in glycolysis and gluconeogenesis. In addition, may also function as scaffolding protein
ANXA5	annexin A5	3,495068	ability to bind to phosphatidylserine, a marker of apoptosis when it is on the outer leaflet of the plasma membrane
K1C9	keratin, type I cytoskeletal 9	3,191425	plays a role in keratin filament assembly
STABP	STAM binding protein	3,182033	Zinc metalloprotease that specifically cleaves 'Lys-63'-linked polyubiquitin chains; plays a role in signal transduction for cell growth and MYC induction mediated by IL-2 and GM-CSF; endosomal localization of STAMBP is required for efficient EGFR degradation but not for its internalization
NP1L4	nucleosome assembly protein 1-like 4	3,145248	acts as histone chaperone in nucleosome assembly
PDIA1	prolyl 4-hydroxylase, beta polypeptide	3,065948	multifunctional protein catalyzes the formation, breakage and rearrangement of disulfide bonds; at the cell surface, seems to act as a reductase that cleaves disulfide bonds of proteins attached to the cell; at high concentrations, functions as a chaperone that inhibits aggregation of misfolded proteins
TBA3	tubulin alpha-1A chain	3,051881	major constituent of microtubules
PDIA4	protein disulfide isomerase family A, memnber 4	2,982396	peptide disulfide oxidoreductase activity; response to endoplasmic reticulum stress
GDIA	GDP dissociation inhibitor 1	2,928630	controls Rho proteins homeostasis
NP1L1	nicotinamide N-methyltransferase	2,811353	may be involved in modulating chromatin formation and contribute to regulation of cell proliferation
ACTG	actin, gamma 1	2,790079	component of the cytoskeleton and as mediators of internal cell motility
RANB3	RAN binding protein 3	2,757964	G1/S transition of mitotic cell cycle; acts as a cofactor for XPO1/CRM1-mediated nuclear export; negative regulator of TGF-beta signaling through interaction with the R-SMAD proteins, SMAD2 and SMAD3, and mediating their nuclear export
SYK	lysyl-tRNA ligase	2,642445	when secreted, acts as a signaling molecule that induces immune response through the activation of monocyte/macrophages
HS90A	heat shock protein 90kDa alpha (cytosolic), class A member 1	2,620103	molecular chaperone that promotes the maturation, structural maintenance and proper regulation of specific target proteins involved for instance in cell cycle control and signal transduction
SDC10	peptidyl-prolyl cis-trans isomerase CWC27 homolog	2,571389	as part of the spliceosome, plays a role in pre-mRNA splicing; probable inactive PPIase with no peptidyl-prolyl cis-trans isomerase activity
RHG01	Rho GTPase activating protein 1	2,569763	GTPase activator for the Rho, Rac and Cdc42 proteins, converting them to the putatively inactive GDP-bound state
TRFL	lactotransferrin	2,540804	iron binding transport proteins which can bind two Fe^{3+} ions in association with the binding of an anion, usually bicarbonate; has antimicrobial activity, which depends on the extracellular cation concentration
NUCB1	nucleobindin 1	2,123962	major calcium-binding protein of the Golgi; may have a role in calcium homeostasis

Table S-6

Protein)1	Function)2	Fold changes)3	Functional description)4
PDIA3	protein disulfide-isomerase A3	2,106542	catalyzes the rearrangement of -S-S- bonds in proteins; positive regulation of extrinsic apoptotic signaling pathway; protein folding in endoplasmic reticulum; response to endoplasmic reticulum stress
ENPL	endoplasmin	2,025110	molecular chaperone that functions in the processing and transport of secreted proteins; actin rod assembly; negative regulation of apoptotic process; protein folding in endoplasmic reticulum; response to endoplasmic reticulum stress; response to hypoxia; ubiquitin-dependent ERAD pathway
CATB	cathepsin B	1,924969	thiol protease which is believed to participate in intracellular degradation and turnover of proteins; epithelial cell differentiation; regulation of apoptotic process
ACTB	actin, beta	1,897699	component of the cytoskeleton and as mediators of internal cell motility
CRK	adapter molecule crk	1,871735	isoform Crk-II: Regulates cell adhesion, spreading and migration; involved in phagocytosis of apoptotic cells and cell motility via its interaction with DOCK1 and DOCK4
SSRD	translocon-associated protein subunit delta	1,742402	TRAP proteins are part of a complex whose function is to bind calcium to the ER membrane and thereby regulate the retention of ER resident proteins
